# Catalytic Enantioselective Dearomatizing [2,3]-Wittig Rearrangements Allow Divergent [2,3]‑, [1,2]‑, and Sommelet–Hauser-Type Products

**DOI:** 10.1021/jacs.6c04473

**Published:** 2026-05-25

**Authors:** Tengfei Kang, Alister S. Goodfellow, Kevin Kasten, David B. Cordes, Aidan P. McKay, Michael Bühl, Andrew D. Smith

**Affiliations:** † EaStCHEM, School of Chemistry, 7486 University of St Andrews, North Haugh, St Andrews, KY16 9ST, U.K.; ‡ Key Laboratory of Applied Surface and Colloid Chemistry, Ministry of Education and School of Chemistry and Chemical Engineering, Shaanxi Normal University, Xi’an 710062 China

## Abstract

The development of catalytic enantioselective dearomatizing [2,3]-rearrangement processes involving the C=C bond of a pendant (hetero)­aromatic substituent remains a recognized significant challenge, primarily due to the energetic penalty associated with the loss of aromaticity in this process. The most common strategy in this area relies upon rearrangement from an onium ylide intermediate, followed by rearomatization, to generate Sommelet–Hauser-type products. Only limited enantioselective variants have been disclosed to date. In this paper, the enantioselective dearomatizing [2,3]-Wittig rearrangement of oxindole-substituted heteroaryl ethers employing a chiral bifunctional iminophosphorane (BIMP) catalyst is demonstrated. Solvent-dependent reactivity is observed, with 1,4-dioxane allowing preferential access to highly enantioenriched [2,3]-Wittig rearrangement products (up to 94:6 dr, 98:2 er). In mesitylene, the formation of enantioenriched [1,2]-Wittig products (up to 97:3 er) is favored via a cascade process consisting of initial [2,3]-rearrangement followed by an enantioretentive fragmentation/recombination event (equivalent to a [1,3]-rearrangement). Sommelet–Hauser-type products (up to 98:2 er) can be accessed by enantioselective [2,3]-Wittig rearrangement followed by selective tautomerization upon treatment with acid. Together, these processes deliver divergent access to heteroaryl-substituted 3-hydroxyoxindole derivatives in high yields and excellent stereoselectivities. Computational DFT studies provide insights into the origin of stereocontrol in the dearomatizing [2,3]-rearrangement event that underpins the observed stereoselectivity.

## Graphical abstract

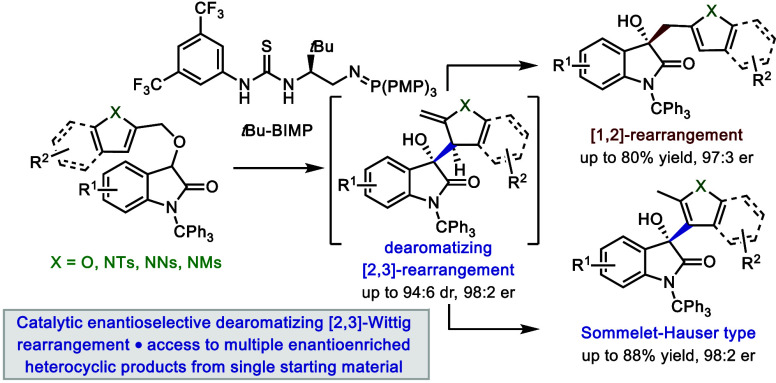

## Introduction

Sigmatropic rearrangements are recognized as one of the most important types of carbon–carbon and carbon–heteroatom bond-forming processes in organic synthesis. Consequently, these reactions have been broadly utilized for the synthesis of complex molecules due to their high reliability and atom economy.^1–6^ Sigmatropic rearrangement processes that incorporate reactivity upon the C=C double bond within an aromatic substituent result in dearomatization, with subsequent rearomatization by tautomerization being generally facile. These processes are widely recognized as challenging due to the significant energetic cost associated with the loss of aromaticity.^7–11^ A number of well-established dearomatizing [3,3]-sigmatropic rearrangements that belong to this class include Cope^12–16^ and Claisen variants,^17–25^ as well as the Fischer indole synthesis.^26–30^ Dearomatizing [2,3]-rearrangements from onium ylide intermediates have also attracted significant attention as archetypally represented by the Sommelet–Hauser reaction and related variants.^31^ These processes are commonly carried out through metal-catalyzed decomposition of a diazo compound to give an electrophilic carbenoid,^32^ which upon nucleophilic addition, generates the onium ylide *in situ* that can undergo dearomatizing [2,3]-rearrangement followed by tautomerization to generate the rearomatized product, with [1,2]-rearrangement products also possible (Figure 1A).^33^ This strategy has been applied to the generation and subsequent rearrangement of sulfonium,^11,34–39^ selenonium,^39^ ammonium,^40–43^ and oxonium ylides.^44^ Stereocontrol in these processes traditionally relies upon the use of stoichiometric chiral auxiliaries^45^ or chirality transfer from an enantioenriched substrate.^40,46–50^ Catalytic enantioselective dearomatizing [2,3]-processes are rare. The current state-of-the-art in this area builds on the Rh­(II)-catalyzed thia-Sommelet–Hauser rearrangement demonstrated by Wang et al. in 2008,^34^ with a catalytic enantioselective variant first realized by Feng et al. using a Ni­(II)/*N*,*N*-dioxide ligand system, delivering enantioenriched sulfide products in up to 93:7 er (Figure 1B).^32,36^ Subsequent work by Wang and Li using a Cu­(I)/chiral bisoxazoline complex, promoted the enantioselective thia-Sommelet–Hauser reaction, giving products in up to 96:4 er.^37^

**Figure 1:**
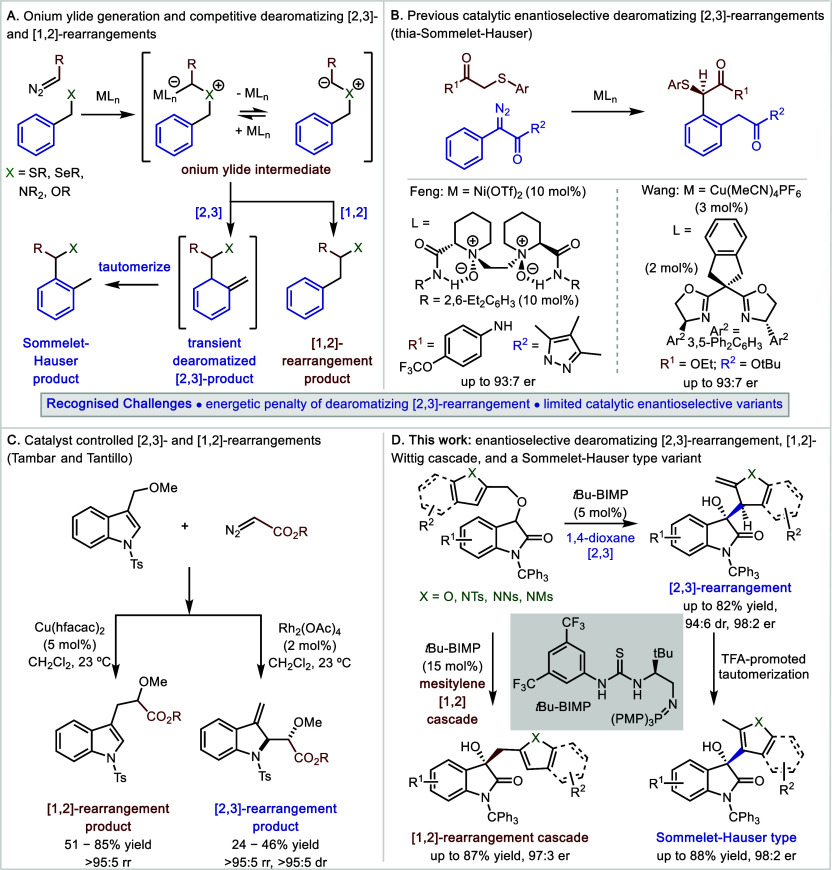
Summary of dearomatizing [2,3]- and [1,2]-sigmatropic rearrangements onto the C=C bond of a (hetero)­aromatic system. **A.** Onium ylide generation and competitive [2,3]- and [1,2]-rearrangement processes. **B.** Previous catalytic enantioselective thia-Sommelet–Hauser variants. **C.** Previously disclosed catalyst-controlled [2,3]- and [1,2]-rearrangements. **D.** This work: enantioselective dearomatizing [2,3]-rearrangements allow access to divergent products (PMP = 4-MeOC_6_H_4_).

In certain circumstances, [1,2]-rearrangement processes can compete with dearomatizing [2,3]-rearrangements of onium ylides, with recent advances describing selective control over these reaction manifolds.^44,51,52^ For example, both Pan et al.^51^ and Koenigs et al.^52^ have reported solvent- and substrate-controlled [1,2]- and [2,3]-rearrangements of sulfonium ylides, while Tambar, Tantillo, et al. documented catalyst-controlled regiodivergent [1,2]- and [2,3]-rearrangements of indole-based onium ylides using Cu and Rh, respectively (Figure 1C).^44^ Although these approaches elegantly demonstrate catalytic control over the formation of [1,2]- and [2,3]-rearrangements involving (hetero)­aromatic substituents, only racemic [1,2]-products have been generated to date.

Processes arising from the treatment of neutral allylic ethers under basic reaction conditions typically generate product mixtures arising from the thermally allowed concerted [2,3]-Wittig rearrangement, alongside minor quantities of [1,2]-Wittig rearrangement products arising from a nonconcerted pathway. To date, only limited reports have detailed dearomatizing [2,3]-Wittig rearrangements from neutral allylic or propargylic ether starting materials, rather than the *in situ* formation of an onium ylide, with no catalytic enantioselective variants reported.^53–57^ In this context, the development of catalytic enantioselective protocols that would *selectively* allow the formation of dearomatized [2,3]-products, as well as the corresponding [1,2]-product or Sommelet–Hauser-type products from a single starting material, was considered. The proposed process builds upon our recent work that described the use of versatile chiral bifunctional iminophosphorane (BIMP) catalysts, elegantly introduced by the Dixon group^58,59^ and now widely exploited in enantioselective catalysis,^60–74^ to deliver [1,2]-Wittig rearrangement products from oxindole-derived allylic ethers.^75^ Mechanistic studies indicate that this process proceeds via a cascade sequence, with initial enantioselective [2,3]-sigmatropic rearrangement,^76–87^ followed by an enantioretentive anionic fragmentation process (equivalent to a [1,3]-rearrangement), rather than the commonly accepted mechanism involving diradical formation and recombination. Building upon this work, it was proposed that BIMP catalysts could promote an enantioselective dearomatizing [2,3]-Wittig rearrangement onto the C=C bond of a pendant heteroaryl substituent (Figure 1D). Subsequent enantioretentive [1,3]-ionic fragmentation/recombination would generate the [1,2]-product in a cascade process. Alternatively, selective rearomatization of the [2,3]-Wittig products would lead to Sommelet–Hauser-type products. If feasible, this process would allow access to three distinct enantioenriched products from a single starting material and offer a novel strategy for the divergent synthesis of valuable nitrogen heterocyclic products.^8–92^ In this paper, the successful realization of this strategy is delineated. Solvent-controlled, BIMP-catalyzed enantioselective access to the products of [2,3]- and [1,2]-rearrangements of indole-based oxindole-derived ethers is demonstrated, alongside selective tautomerization of the [2,3]-products, giving diverse 3-hydroxyoxindoles in high yields with excellent stereoselectivities.

## Results and Discussion

### Initial Optimization: Solvent-Controlled Divergent Reactivity

Initial studies considered oxindole ether **1** as a model substrate for reaction optimization, as previous work demonstrated the requirement of an *N*-trityl substituent for optimal product enantioselectivity (Table 1).^75^ An *N*-tosyl indole substituent was chosen for the dearomatizing [2,3]-rearrangement, as the introduction of heteroatoms onto an aromatic ring is known to dramatically decrease the energetic cost of dearomatization,^93–103^ while the indole structural motif is also common in many natural products and medicinally relevant compounds.^104–106^ Treatment of ether **1** with *t*Bu-BIMP **2** (20 mol %) in ethereal solvents at 30 °C showed a preference for the [2,3]-rearrangement product **3** over the [1,2]-product **4**. For example, using THF and 1,4-dioxane gave the [2,3]-rearrangement product in 60% and 80% yield, respectively, with good stereoselectivity (83:17 dr, 96:4 and 98:2 er, Table 1, entries 1 and 2). Further optimization using 1,4-dioxane with 5 mol % of *t*Bu-BIMP **2** gave [2,3]-rearrangement product **3** in 76% isolated yield (83:17 dr, 98:2 er, entry 3). Consistent with our previous work that demonstrated the interconversion of [2,3]-products to [1,2]-products is preferred upon treatment with base in low-polarity aromatic solvents, treatment of ether **1** in toluene with *t*Bu-BIMP **2** (20 mol %) at rt gave preferentially [1,2]-rearrangement product **4** in 71% yield (93:7 er). Two other products were observed, with the rearomatized Sommelet–Hauser type product **5** (arising from [2,3]-rearrangement and tautomerization) formed in 10% yield. Isatin derivative **6** (11%) was also formed, presumably generated by oxy-Cope rearrangement of the initially generated [2,3]-product **3**, followed by rearomatization. This process can be likened to the widely studied Cope rearrangement–tautomerization observed in isoprenoid biosynthesis^15,107–122^ but was not optimized herein (Table 1, entry 4). Further work showed that the use of trifluorotoluene and chlorobenzene showed inferior results. For example, trifluorotoluene gave only 59% conversion to a mixture of all products **36** due to poor starting material solubility at rt, giving [1,2]-rearrangement product **4** in 28% yield (97:3 er, entry 5). In chlorobenzene, a range of products **3**–**6** was again observed, with **4** formed in 55% yield (96:4 er, entry 6). Conducting the reaction in mesitylene at 30 °C led to suppression of both side products **5** and **6**, giving [1,2]-rearrangement product **4** in 80% yield (95:5 er, entry 7). Further optimization showed that the catalyst loading could be reduced while maintaining product yield and stereoselectivity. In mesitylene, using 15 mol % of **2** delivered [1,2]-rearrangement product **4** in 72% yield (96:4 er, entry 8). The absolute (*S*)-configuration of the [1,2]-rearrangement product **4** was unambiguously determined through X-ray crystallographic analysis of product **7** that was formed by N-detritylation of **4** (see Supporting Information for further details).

**Table 1:**
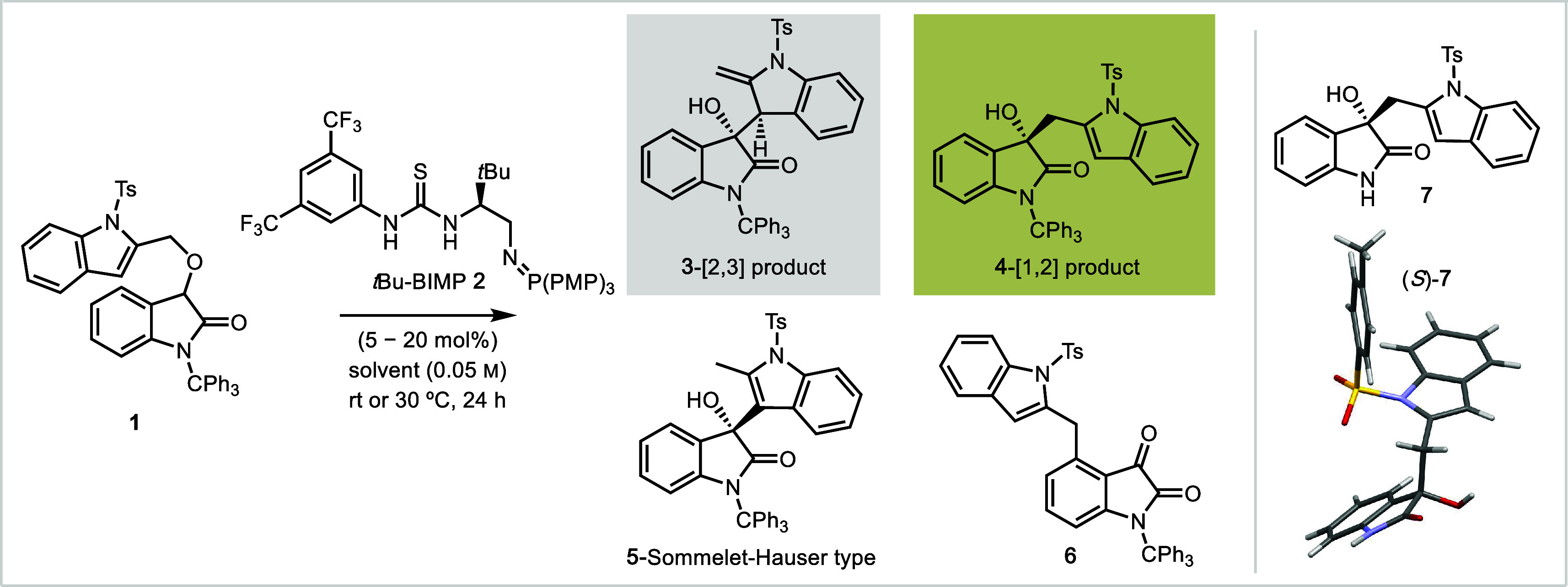
Optimization Process

				product ratio (%)^a^				3		4
Entry	solvent	2 (mol %)	conversion (%)	3	4	5	6	dr^a^	er^b^	er^b^
1^c^	THF	20	>95	60	30	<5	<5	83:17	96:4	97:3
2^c^	1,4-dioxane	20	>95	80	10	<5	<5	83:17	98:2	-
**3** ^c^,^d^	1,4-dioxane	5	92	84 (76)	7	<5	<5	83:17	98:2	-
**4**	toluene	20	>95	0	71	10	11	-	-	93:7
**5**	trifluorotoluene	20	59	10	28	8	8	80:20	-	97:3
**6**	chlorobenzene	20	95	6	55	8	10	67:33	-	96:4
7^c^	mesitylene	20	>95	0	80	7	5	-	-	95:5
8^c^,^d^	mesitylene	15	>95	0	80 (72)	8	7	-	-	96:4

a Product ratio and conversion were determined by ^1^H NMR analysis using 1,3,5-trimethoxybenzene as an internal standard.

b Determined by HPLC analysis on a chiral stationary phase.

c At 30 °C.

d Isolated yield is shown in brackets.

### Scope and Limitations

#### Enantioselective Dearomatizing [2,3]-Wittig Rearrangement

With optimized conditions developed, the ability to generate selectively and isolate a series of dearomatized [2,3]-Wittig rearrangement products was probed (Figure 2). Using *t*Bu-BIMP **2** (5 mol %) in 1,4-dioxane, a variety of halogenated (4-Cl-, 6-Cl-, and 6-Br-), electron-withdrawing (5-O_2_N-) and electron-donating (5-Me- and 5-MeO-) substituents on the oxindole were tolerated, affording **8** to **13** in high yields (72–81%), with good to excellent diastereocontrol (86:14–94:6 dr) and uniformly excellent enantiocontrol (97:3–98:2 er). Notably, rearrangement of substrates incorporating a halogen or 5-O_2_N substituent was complete within short reaction times (generally 2 min to 1 h). These containing electron-donating (notably 5-MeO) substituents generally required higher catalyst loading and extended reaction times to reach completion. Substituent variation on the indole undergoing dearomatization was also tolerated, with 5-Br-, 5-Me-, and 6-Me variants **14–** **16** generated in good yields (73–82%) and enantiocontrol (97:3–98:2 er). The relative and absolute (3*R*,3′*S*)-configuration within the preferred diastereoisomer **15** was unambiguously proven by X-ray crystallographic analysis, with all others assigned by analogy.

**Figure 2:**
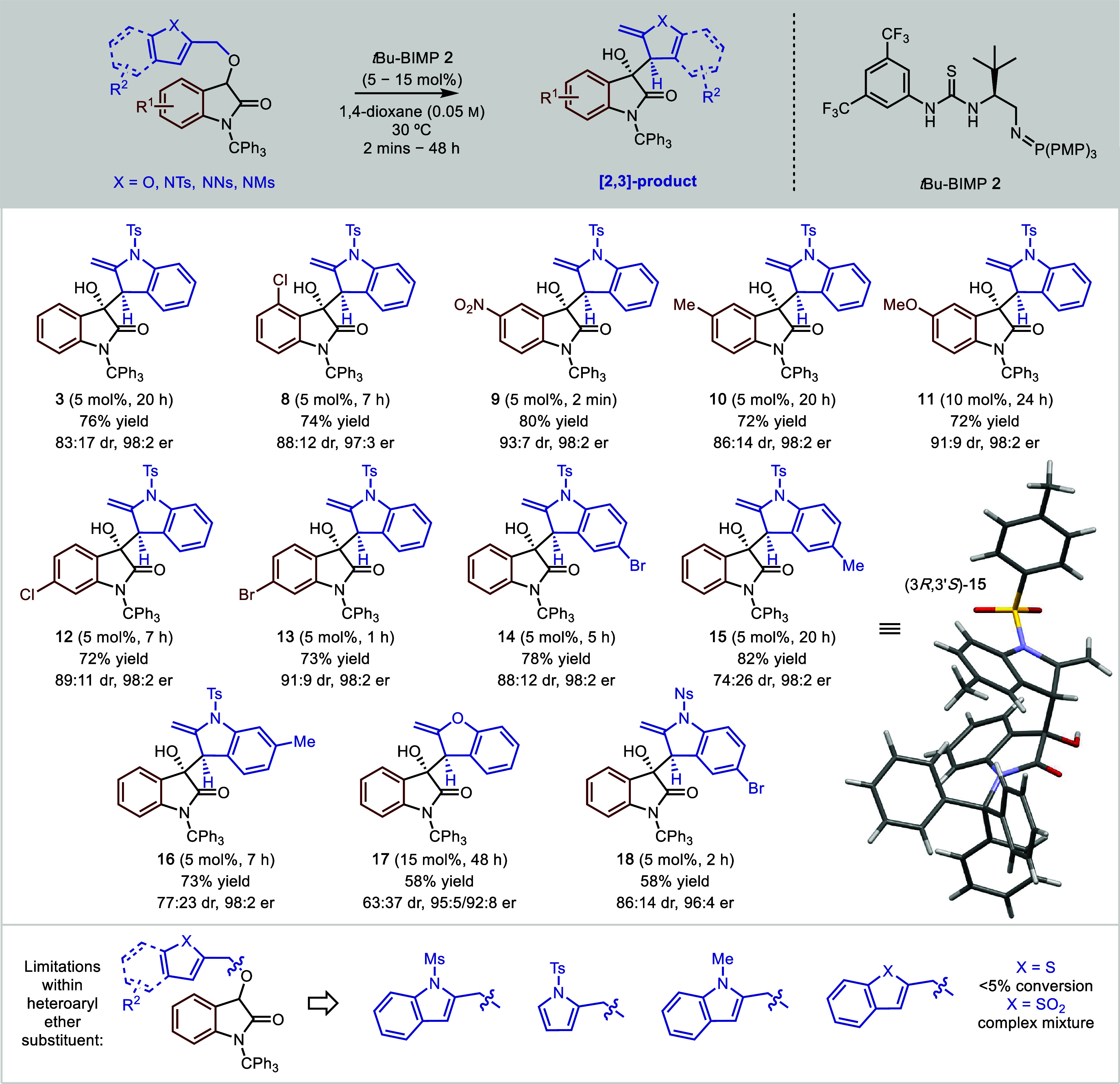
Substrate scope and limitations of the [2,3]-rearrangements. Ts = *p*-O_2_S–C_6_H_4_–Me, Ns = *p*-O_2_S–C_6_H_4_–NO_2_. Reactions performed on a 0.10 mmol scale. Yields are isolated yields. Enantiomeric ratios were determined by HPLC analysis on a chiral stationary phase.

Application to a benzofuran-derived substrate was successful, giving **17** (58% yield, 63:37 dr, and 95:5/92:8 er). Variation of the N-indole substituent showed that N-Ns substitution was tolerated, giving **18** (58% yield, 86:14 dr, and 96:4 er). The moderate isolated yields of **17** and **18** reflect competitive formation of Sommelet–Hauser-type and [1,2]-rearrangement products. Limitations of this methodology indicated that the corresponding N-Ms, as well as N-Ts-2-pyrrole and N-Ts-2-methylindole substrates, gave a mixture of products, which we attribute to competitive onward reactivity to afford the [1,2]-products (see Figure 3). Further limitations under these conditions showed that *N*-methylindole, benzo­[*b*]­thiophene, and benzo­[*b*]­thiophene 1,1-dioxide-derived substrates showed low (less than 10%) conversion to product.

**Figure 3:**
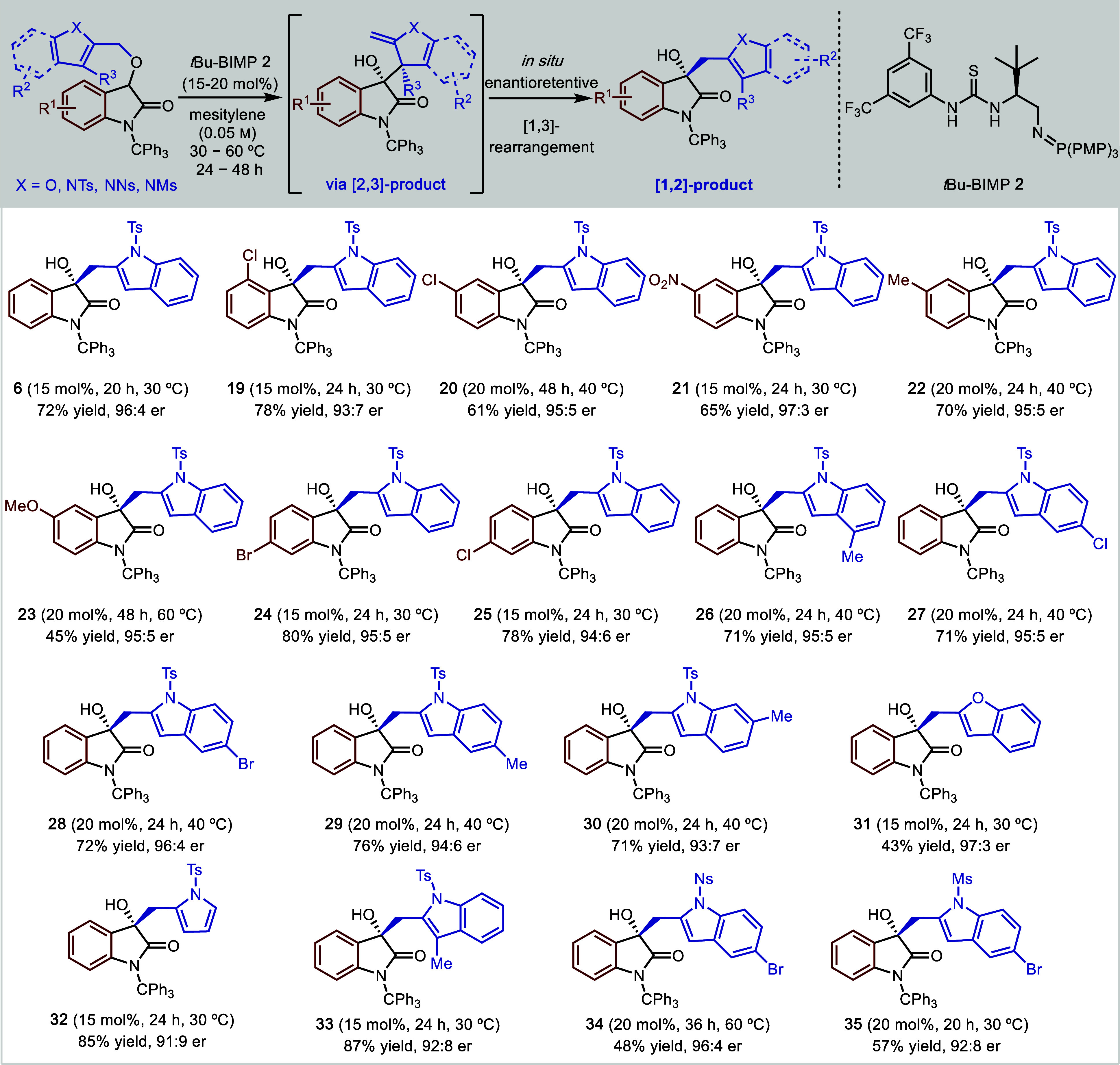
Substrate scope and limitations of the [1,2]-rearrangement cascade. Ts = *p*-O_2_S–C_6_H_4_–Me, Ns = *p*-O_2_S–C_6_H_4_–NO_2_. Reactions performed on a 0.10 mmol scale. Yields are isolated yields. Enantiomeric ratios were determined by HPLC analysis on a chiral stationary phase.

### Enantioselective [1,2]-Wittig Rearrangement Cascade

The generality of the approach to selectively prepare [1,2]-Wittig rearrangement products through a cascade process via [2,3]-rearrangement and in situ enantioretentive [1,3]-shift was next probed (Figure 3). Variation of the oxindole heteroaromatic substituent and other aromatic heterocycles was tested. Oxindole substrates with substituents at various positions worked efficiently, including halogen (4-Cl-, 5-Cl-, 6-Br-, and 6-Cl-), electron-donating (5-Me-) and electron-withdrawing substituents (5-O_2_N-), delivering products **19**–**22** and **24**–**25** in 61% to 80% yield and 93:7–97:3 er. A strong electron-donating 5-OMe-substituent reduced product yield, but high enantioselectivity was observed (**23,** 45% yield, 95:5 er), with the low yield attributed to the increased proportion of side product derived from the oxy-Cope rearrangement. The substrate scope with respect to substituent variation on the indole moiety was also examined. Halogens (5-Cl and 5-Br) and methyl substitution on the indole ring were tolerated, giving products **26**–**30** in high yields (71–76%) and enantioselectivity (93:7–96:4 er). Alternative heteroaromatic substituents were also investigated, with 2-benzofuran, 2-pyrrole, and 2-methylindole-substituted substrates giving **31** to **33** in moderate to high yields (43–87%) and high enantioselectivity (91:9–97:3 er). N-Ns and N-Ms substitution was also tolerated, giving **34** in 96:4 er and **35** in 92:8 er, respectively. The reduced yield for products **31** and **35** is attributed to the competitive formation of Sommelet–Hauser type products under the reaction conditions. Consistent with the limitations of the [2,3]-Wittig rearrangement, benzo­[*b*]­thiophene and benzo­[*b*]­thiophene 1,1-dioxide-derived substrates either only returned starting material or gave complex mixtures under the reaction conditions.

### Enantioselective Sommelet–Hauser Product Formation

Although isolable, the dearomatized-[2,3]-Wittig rearrangement products are relatively prone to rearomatization upon treatment under basic or acidic conditions, or upon storage (see Supporting Information for details). For example, control experiments indicate that treatment of dearomatized-[2,3]-Wittig product **3** with DBU led to a 70:30 mixture of Sommelet–Hauser type product **5** (98:2 er) and [1,2]-product **4** (91:9 er). The use of strongly acidic conditions (TsOH or TFA) allowed controlled rearomatization of **3** to give **5** in excellent yield and enantioselectivity. This was optimized (see Supporting Information) to show that **3** (83:17 dr, 98:2 er) could be selectively tautomerized to give Sommelet–Hauser type product **4** in the presence of TFA with retention of enantioselectivity (80% yield, 97:3 er). Based on this finding, a one-pot cascade process to generate Sommelet–Hauser type products via BIMP-catalyzed [2,3]-Wittig rearrangement, followed by tautomerization via treatment with TFA, was developed, affording direct access to a range of highly enantioenriched 3-indoline-3-hydroxyoxindoles (Figure 4). This process was tolerant of a variety of different halogenated (5-Cl-, 6-Cl-, and 6-Br-), electron-withdrawing (5-O_2_N-) and electron-donating (5-Me- and 5-MeO-) substituents on the oxindole skeleton, affording **36** to **41** in high yields (61–88%) and excellent enantiocontrol (95:5 er–98:2 er). The absolute (*R*)-configuration within **39** was proven by crystallographic analysis, consistent with rearomatization proceeding with retention of configuration at C(3) from the corresponding [2,3]-product. The substrate scope with respect to substituent variation on the indole moiety indicated that 5-Cl-, 5-Br-, 5-Me-, and 6-Me positions were tolerated, giving the rearomatized [2,3]-Wittig products **42**–**45** in high yields (71–76%) and enantioselectivity (97:3–98:2 er). Application to a benzofuran-derived substrate gave **46** in 50% yield (94:6 er), while N-Ns substitution gave **47** in 72% yield (96:4 er). While N-tritylation of 7-chloroisatin was unsuccessful, the *N*-benzyl analogue was prepared and tested, giving 7-Cl **48** in 81% yield (90:10 er). Treatment of isolated N-Ns protected product **47** (96:4 er) with PhSH and K_2_CO_3_ promoted N-Ns deprotection to generate **49** in 80% yield and 96:4 er.

**Figure 4:**
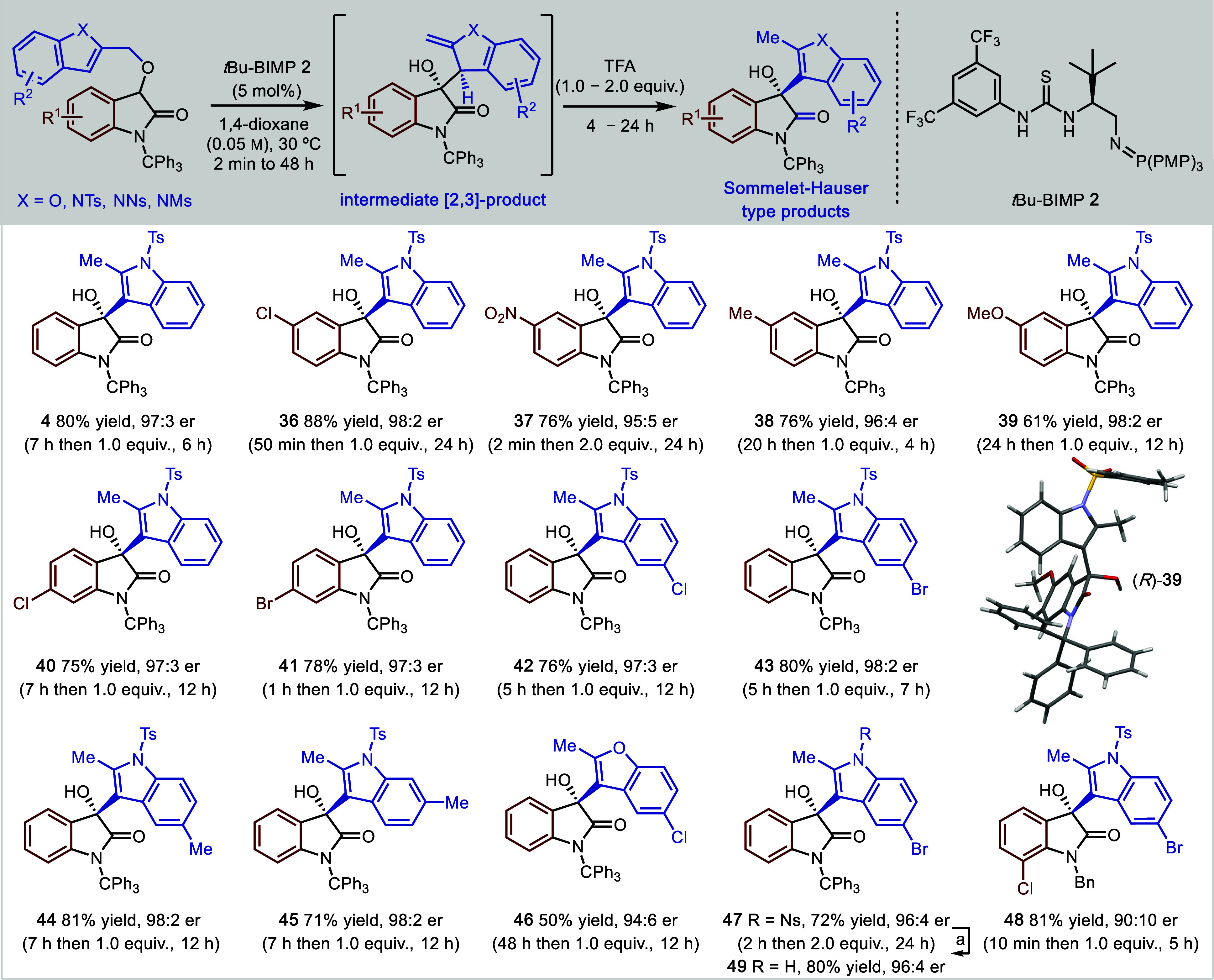
Scope of one-pot [2,3]-rearrangement/tautomerization protocol. Ts = *p*-SO_2_–C_6_H_4_–Me, Ns = *p*-SO_2_–C_6_H_4_–NO_2_. Reactions performed on a 0.10 mmol scale. Yields are isolated yields. Enantiomeric ratios were determined by HPLC analysis on a chiral stationary phase. a. PhSH (3 equiv), K_2_CO_3_ (5 equiv), MeCN, rt, 16 h.

### Computational Analysis of Dearomatizing [2,3]-Wittig Rearrangement

On the basis of our previous work^75^ and DFT calculations, the proposed mechanistic scheme for the observed divergent processes is illustrated in Figure 5. In either 1,4-dioxane or mesitylene, it is assumed that the initial association of BIMP to the substrate and subsequent deprotonation leads to an ether–BIMP complex **I** that undergoes a stereodetermining dearomatizing [2,3]-Wittig rearrangement process to give **II**, which after proton transfer and catalyst release, generates the isolable [2,3]-rearrangement product **3**. This product can subsequently be tautomerized to generate the Sommelet–Hauser-type product **5** under acidic conditions. Alternatively, in mesitylene and under the basic catalytic reaction conditions, deprotonation of **3** by *t*Bu-BIMP is followed by a formal [1,3]-rearrangement (that proceeds via ionic fragmentation/recombination) with retention of configuration to generate the [1,2]-rearrangement product **4**.^123–128^

**Figure 5:**
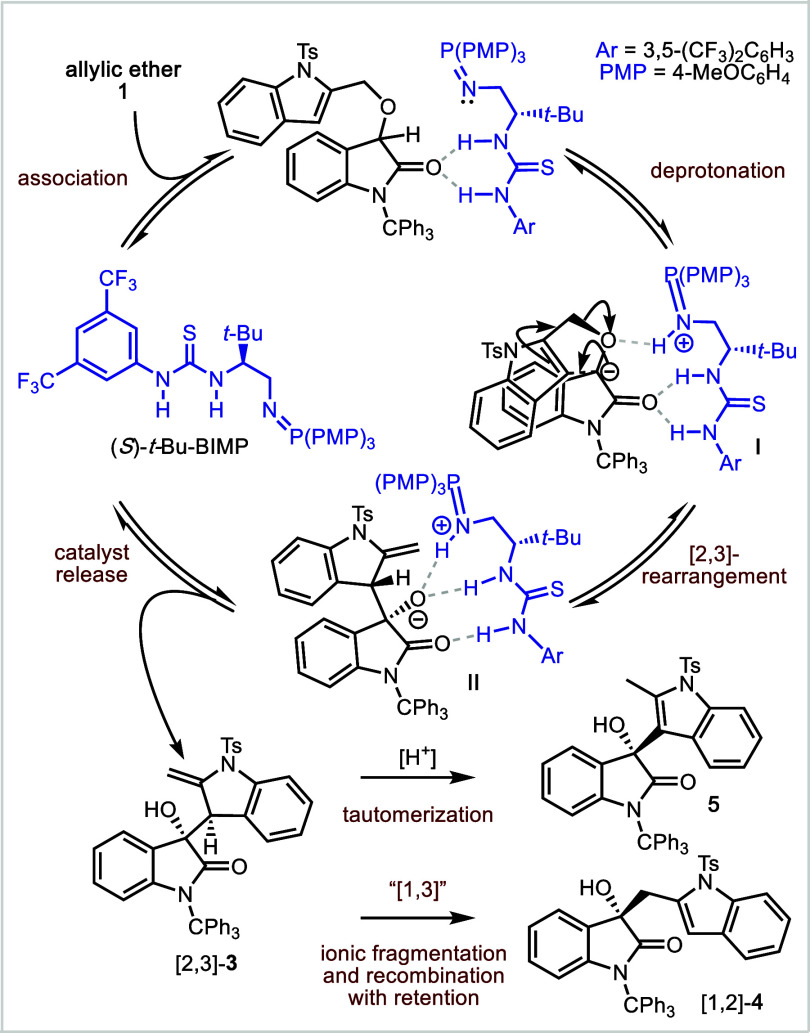
Outline of the catalytic cycle for the generation of **3** from **1** and subsequent *in situ* derivatization to give **4** or **5**.

Fundamental to the observed product enantioselectivity in these possible reaction outcomes is the role of the BIMP catalyst within the dearomatizing [2,3]-rearrangement process. This process has been investigated using DFT (Figure 6A). As the size and flexibility within this catalytic system present a significant conformational challenge to probe computationally, an alternative approach to that developed by Grayson and coworkers in our previous computational work was considered.^75^ In this approach, the conformational flexibility of the [2,3]-transition state (TS) geometry for the formation of the 4 possible diastereoisomers was considered. Constrained meta-dynamics using CREST^129^ with the semiempirical GFN2-xTB^130^ were performed, and each conformer was fully optimized to a transition state with GFN2-xTB. Refinement of electronic energies through M06-2X/def2-SVP single-point calculations allowed for the selection of 20 conformers (5 of each diastereoisomer) to study at the full DFT level (see Supporting Information for more details). Using the level of M06-2X_CPCM(THF)_/def2-TZVP//M06-2X_CPCM(THF)_/def2-SVP^131–133^ with ORCA 5.0.4 ^134^ (which has performed well in previous computational studies of similar organocatalytic reactions^72,135–137^ ) the favored transition state leads to the experimentally observed (3*R*,3′*S*)-diastereoisomer, proceeding through a “loose” transition state primarily associated with the breaking of the ether C–O bond.^138^ This transition state is favored by ΔΔ^‡^ *G*_298_ = 1.7 kcal/mol, in good agreement with the experimentally observed diastereoselectivity (Figure 6B).

**Figure 6:**
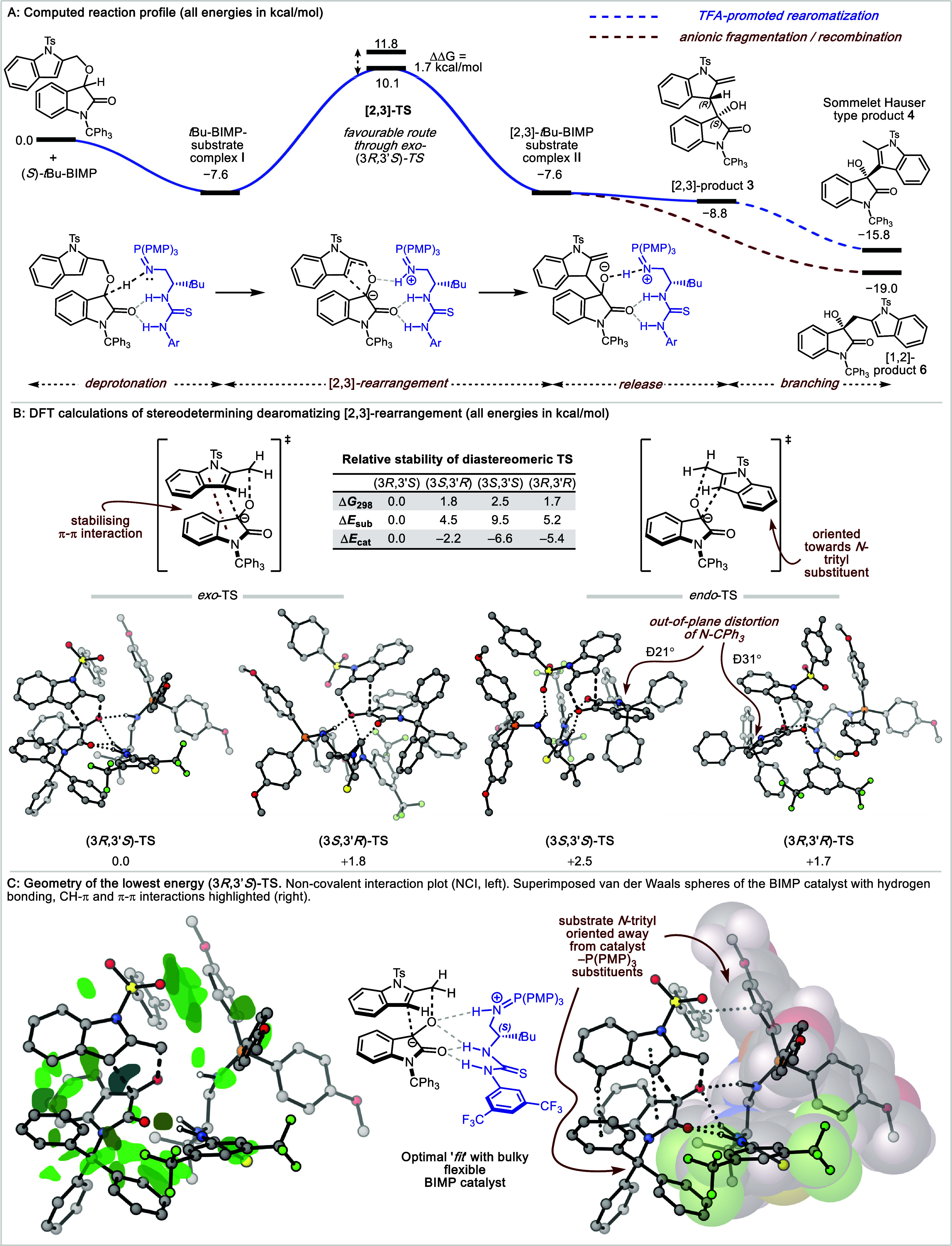
Computational investigation of the dearomatizing [2,3]-sigmatropic rearrangement. **A.** Computed reaction profile at the M06-2X_CPCM(THF)_/def2-TZVP//M06-2X_CPCM(THF)_/def2-SVP level of theory. **B.** *Exo*- and *endo*-transition states, with the most stable computed conformation for each diastereoisomer. Relative Gibbs free energies (Δ*G*_298_) and reorganization energies of the separated substrate (Δ*E*_sub_) and catalyst (Δ*E*_cat_) in kcal/mol. **C.** Lowest energy (3*R*,3′*S*)-transition state with noncovalent interaction plot (left), with attractive electrostatic (blue) and van der Waals (green) interactions highlighted; superimposed van der Waals spheres of the catalyst and interactions (right). Images were generated using xyzrender.^139^

Deprotonation of the ether substrate by the BIMP organocatalyst generates the resultant anion-BIMP complex (Δ*G* = −7.6 kcal/mol) that exhibits multiple hydrogen-bonding interactions from the thiourea and the conjugate acid of the iminophosphorane Brønsted base. Within this complex, the large steric bulk associated with the substrate (*N*-trityl substituent) and the catalyst (-P­(PMP)_3_ group) are the main structural motifs that enforce the geometry of the subsequent transition state structures (Figure 6C, right). Within the favored (3*R*,3′*S*)-TS, these sterically imposing substituents are oriented as far apart as possible to minimize steric interactions. The rearrangement proceeds through a formal *exo*-transition state, which is stabilized by intramolecular CH−π and π–π interactions, leading to a lower-energy arrangement of the substrate compared to the *endo*-TS (Figure 6B, 6C).^139^ Complementary to this stabilization, the *endo*-TS is further destabilized due to the enforced proximity of the indole moiety toward the *N*-trityl protecting group of the oxindole, leading to an out-of-plane distortion (*Đ*) of the *N*–CPh_3_ bond by up to 30° (Figure 6B). The energy of the substrate is significantly stabilized in the geometry of the (3*R*,3′*S*)-TS by 9.5 and 5.2 kcal/mol compared to the diastereomeric (3*S*,3′*S*)- and (3*R*,3′*R*)-TS, respectively. With a stabilized *exo*-TS generating the (3*R*,3′*S*)- and (3*S*,3′*R*)-products, the enantiocontrol arises due to the preferential fit with the flexible (*S*)-*t*Bu-BIMP organocatalyst. The noncovalent interaction (NCI) plot (Figure 6C, left) for the favored (3*R*,3′*S*)-TS shows widespread dispersion interactions between the fragments, dominated by attractive electrostatic (blue surfaces) and van der Waals interactions (green surfaces). The geometric fit of the two components is challenging to quantify. According to distortion–interaction analysis using the Activation Strain Model,^140^ the (3*S*,3′*R*)-TS has a less stable conformation of the substrate by 4.5 kcal/mol and a catalyst stabilization of 2.2 kcal/mol, leading to an overall destabilization of 2.3 kcal/mol (relative to the lowest (3*R*,3′*S*)-TS), indicating additional strain in the TS (see Section 2.4 in the Supporting Information for further details).

Within the proposed mechanism, the substrate undergoes a dearomatizing [2,3]-Wittig rearrangement with a computed barrier height of 17.7 kcal/mol to generate the initially formed [2,3]-product **3** (Δ*G* = −8.8 kcal/mol). Dependent upon the subsequent reaction conditions, the [2,3]-Wittig product can be converted to either the [1,2]-product (Δ*G* = −19.0 kcal/mol) or the Sommelet–Hauser type product (Δ*G* = −15.8 kcal/mol).^141^ Based on previous computational work by Grayson et al.,^75^ the [1,3]-rearrangement to form the formal [1,2]-product was assumed to proceed via anionic fragmentation and recombination with retention of configuration and was not computed here. Similarly, the barrier to proton transfer that leads to rearomatization and the Sommelet–Hauser type product was also not calculated.

## Conclusions

This paper describes the catalytic enantioselective dearomatizing [2,3]-Wittig rearrangement of oxindole-derived heteroaromatic ethers, employing a chiral bifunctional iminophosphorane (BIMP) catalyst. Solvent-dependent selectivity is observed, with 1,4-dioxane facilitating the dearomatizing [2,3]-rearrangement. In mesitylene, the reaction predominantly favors the [1,2]-rearrangement product via a cascade process consisting of an initial [2,3]-rearrangement followed by enantioretentive formal [1,3]-rearrangement (via fragmentation and recombination) with retention of configuration. A one-pot [2,3]-rearrangement/tautomerization process was also established, affording divergent synthesis of 3-hydroxyoxindoles in good yields (up to 88%) and high stereoselectivities (up to 94:6 dr and 98:2 er). In support of the proposed reaction mechanism, computational DFT studies provide a rationale for the observed stereoselectivity within the key dearomatizing [2,3]-Wittig rearrangement. Further studies concerning the generality and selectivity of related [1,2]- and dearomatizing [2,3] rearrangements are currently ongoing within this laboratory.

## Supplementary Material





## Data Availability

The data underpinning this manuscript is available from the University of St Andrews Research Portal, Pure ID: 328179032, “BIMP-Catalyzed Enantioselective Dearomatizing [2,3]-Wittig Rearrangements of Heteroaryl Ethers Allows Divergent [2,3]-, [1,2]- and Sommelet-Hauser-Type Reactions” doi: 10.17630/25dda982-fbc2-42f2-9f75-c310ad3344ff.

## Notes

Optimized Cartestian coordinates from DFT calculations (XYZ)
Experimental procedures, characterization data, NMR spectra, HPLC traces, DFT calculations, as well as X-ray crystallographic data for **7**, **15**, and **39** (PDF)
