# Supplementary material for: Catalytic Enantioselective Dearomatizing [2,3]-Wittig Rearrangements Allow Divergent [2,3]‑, [1,2]‑, and Sommelet–Hauser-Type Products
Source: J Am Chem Soc. 2026 May 25;148(22):22990–3002. doi: 10.1021/jacs.6c04473 (PMC13266707; doi:10.1021/jacs.6c04473)
Supplement: Supplementary file 2 [file ja6c04473_si_002.pdf]

Supporting Information

Catalytic Enantioselective Dearomatizing [2,3]-Wittig Rearrangements Allow Divergent [2,3]-, [1,2]- and Sommelet-Hauser-Type Products

Tengfei Kang,[1,2*] Alister Goodfellow,[1] Kevin Kasten,[1] David B. Cordes,[1] Aidan P. Mckay,[1] Michael Bühl,[1*] Andrew D. Smith[1*]

[1]EaStCHEM, School of Chemistry, University of St Andrews, North Haugh, St Andrews, KY16 9ST, UK.

[2] Key Laboratory of Applied Surface and Colloid Chemistry, Ministry of Education and School of Chemistry and Chemical Engineering, Shaanxi Normal University, Xi'an, 710062 China

*e-mail: tfkang@snnu.edu.cn; mb105@st-andrews.ac.uk; ads10@st-andrews.ac.uk

Contents

1 General Information 3
1.1 Materials and Methods 3
1.2 General Procedures 5
1.2.1 Preparation of heteroaryl ethers 5
1.2.2 Preparation of oxindole substituted heteroaryl ethers 6
1.2.3 BIMP-catalyzed rearrangement reaction. 7
2 Computational Studies 8
2.1 Computational Details 8
2.2 Computational Discussion 11
2.3 IRC Traces 11
2.4 Conformational Analysis 12
2.5 Strain Analysis 14
3. Control experiments of [2,3]-rearrangement product 3. 18
4 Derivatization of rearrangement products. 18
5. Characterisation of compounds 19
6. X-ray diffraction data 74
7. References 76
8. HPLC data of chiral compounds 79
9. NMR spectral data for compounds 125
10. Computational raw data 283

1 General Information

1.1 Materials and Methods

Reactions involving moisture sensitive reagents were carried out in flame-dried glassware under a nitrogen atmosphere using standard vacuum line techniques. Anhydrous solvents (THF, CH_2Cl_2, Et_2O and toluene) were obtained from an anhydrous solvent system (purified using an alumina column, Mbraun SPS-800). Petrol is defined as petroleum ether 40–60 °C. All other solvents and commercial reagents were used as received without further purification unless otherwise stated.

Room temperature (rt) refers to 15–20 °C. Temperatures of 0 °C, and –78 °C were obtained using ice/water and CO_2(s)/acetone baths, respectively. Temperatures of 0 °C to –78 °C for overnight reactions were obtained using an immersion cooler (HAAKE EK 90). Unless otherwise stated, reactions involving heating were performed using DrySyn blocks and a contact thermocouple.

Under reduced pressure or 'in vacuo' refers to the use of either a Büchi Rotavapor R200 with a Büchi V-491 heating bath and Büchi V-800 vacuum controller, a Büchi Rotavapor R-210 with a Büchi V-491 heating bath and Büchi V-850 vacuum controller, a Heidolph Laborota 4001 with vacuum controller, an IKA RV10 rotary evaporator with a IKA HB10 heating bath and ILMVAC vacuum controller, or an IKA RV10 rotary evaporator with a IKA HB10 heating bath and Vacuubrand CVC3000 vacuum controller. Rotary evaporator condensers are fitted to Julabo FL601 Recirculating Coolers filled with ethylene glycol and set to –6 °C.

Analytical thin layer chromatography (TLC) was performed on pre-coated aluminium plates (Kieselgel 60 F254 silica). TLC visualisation was carried out with ultraviolet light (254 nm) and/or staining with either aqueous $KMnO_4$ solution, ethanolic phosphomolybdic acid, or ethanolic Vanillin solution followed by heating. Flash column chromatography was performed in glass columns fitted with porosity 3 sintered discs over Kieselgel 60 silica using the solvent system stated. Automated chromatography was performed on Biotage® Sfär™ Silica HC D or Biotage® Sfär™ Silica D columns. Melting points were recorded on an Electrothermal 9100 melting point apparatus. Optical rotations were measured on a PerkinElmer Precisly/Model-341 polarimeter operating at the sodium D line with a 100 mm path cell at rt.

Infrared spectra were recorded on a Shimadzu IRAffinity-1 Fourier transform IR spectrophotometer fitted with a Specac Quest ATR accessory (diamond puck). Spectra were

recorded of either thin films or solids, with characteristic absorption wavenumbers (ν_{max}) reported in cm^{-1}.

HPLC analyses were obtained on either a Shimadzu HPLC consisting of a DGU-20A5 degassing unit, LC-20AT liquid chromatography pump, SIL-20AHT autosampler, CMB-20A communications bus module, SPD-M20A diode array detector and a CTO20A column oven or a Shimadzu HPLC consisting of a DGU-20A5R degassing unit, LC-20AD liquid chromatography pump, SIL-20AHT autosampler, SPD-20A UV/Vis detector and a CTO-20A column oven. Separation was achieved using either DAICEL S2 CHIRALPAK OD-H column or DAICEL CHIRALPAK AD-H, IB, IC columns using the method stated. HPLC traces of enantiomerically enriched compounds were compared with authentic racemic spectra. Racemic compounds were synthesized under analogous reaction conditions using DBU or Cs_2CO_3 as base.

^{1}H, ^{13}C, ^{19}F nuclear magnetic resonance (NMR) spectra were acquired on either a Bruker Avance 300 (^{1}H 300 MHz; ^{13}C 75 MHz; ^{19}F 282 MHz), Bruker Avance II 400 (^{1}H 400 MHz; ^{13}C 101 MHz; ^{19}F 376 MHz) or a Bruker Avance II 500 (^{1}H 500 MHz, ^{13}C 126 MHz, ^{19}F 470 MHz), spectrometer at ambient temperature in the deuterated solvent stated. All chemical shifts are quoted in parts per million (ppm) and referenced to the residual solvent peak. All coupling constants, *J*, are quoted in Hz. Multiplicities are indicated by: s (singlet), d (doublet), t (triplet), q (quartet), dd (doublet of doublets), dt (doublet of triplets), dq (doublet of quartets), td (triplet of doublets), ddd (doublet of doublet of doublets), ddt (doublet of doublet of triplets) and m (multiplet). The abbreviation Ar is used to denote aromatic, Ph to denote phenyl, Bn to denote benzyl, br to denote broad and app to denote apparent. NMR peak assignments were confirmed using 2D ^{1}H correlated spectroscopy (COSY), 2D ^{1}H–^{13}C heteronuclear multiple-bond correlation spectroscopy (HMBC), and 2D ^{1}H–^{13}C heteronuclear single quantum coherence (HSQC) where necessary. For diastereomers, not all minor signals have been resolved.

Mass spectrometry (*m*/*z*) data were acquired by either electrospray ionisation (ESI), electron impact (EI), or matrix-assisted laser desorption/ionisation with no matrix (MALDI (no matrix)) at the University of St Andrews Mass Spectrometry Facility.

1.2 General Procedures

1.2.1 Preparation of heteroaryl ethers

General Procedure A:

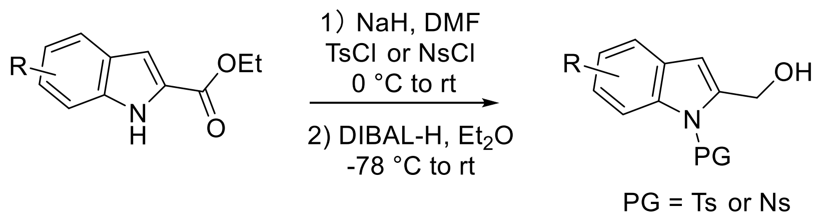

Step I: To a stirred solution of the appropriate ethyl 1*H*-indole-2-carboxylate (1.0 equiv.) was added sodium hydride (60% dispersion in mineral oil, 1.5 equiv.) slowly in dry DMF (0.5 M) at 0 °C under N_2. The mixture was stirred at this temperature for 30 min before TsCl or NsCl (1.5 equiv.) was added during a period of 5 min, and the reaction mixture was allowed to warm to rt and stirred overnight. After completion was determined by TLC, the mixture was quenched with saturated NH_4Cl solution and extracted with ethyl acetate (×3). The combined extracts were washed sequentially with 1 m HCl (×2), H_2O (×3), and brine (×2). The organic layer was dried over $MgSO_4$ and concentrated under reduced pressure to afford the crude product which was then either crystallized or purified by flash column chromatography (PE:EA = 7:1 to 3:1) to give the target product.

Step II: To a solution of the appropriate protected 1*H*-indole-2-carboxylate (1.0 equiv.) obtained above in anhydrous CH_2Cl_2 (0.25 M) was added DIBAL-H (1.0 M in hexanes, 2.5 equiv.) dropwise while stirring at −78 °C. The mixture was stirred for 3 h, then allowed to warm to rt. Saturated sodium potassium tartrate solution was added and the mixture was stirred for 2 h. The phases were separated, and the aqueous phase was extracted with CH_2Cl_2 (×2). The combined organic extracts were washed with brine, dried over $MgSO_4$, and concentrated *in vacuo*. The residue was purified by flash column chromatography, unless otherwise specified, to give the corresponding alcohols.

General Procedure B:

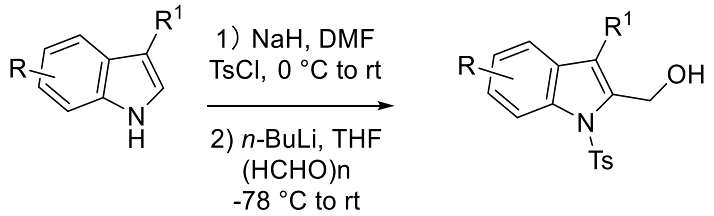

Step I: To a stirred solution of the appropriate substituted indole (1.0 equiv.) was added sodium hydride (60% dispersion in mineral oil, 1.5 equiv.) slowly in dry DMF (0.5 M) at 0 °C

under N_2. The mixture was stirred at this temperature for 30 min before TsCl (1.5 equiv.) was added during a period of 5 min, and the reaction mixture was allowed to warm to rt and stirred overnight. After completion was determined by TLC, the mixture was quenched with saturated NH_4Cl solution and extracted with ethyl acetate (×3). The combined extracts were washed sequentially with 1.0 M HCl (×2), H_2O (×3), and brine (×2). The organic layer was dried over $MgSO_4$ and concentrated under reduced pressure to afford the crude product which was then either crystallized or purified by flash column chromatography (PE:EA = 10:1 to 5:1) to give the target product.

Step II: To a solution of the appropriate substituted *N*-tosyl protected indole (1.0 equiv.) obtained above in anhydrous THF (0.5 M) was added *n*-BuLi (2.5 M in hexanes, 1.1 equiv.) dropwise while stirring at −78 °C. The mixture was stirred for 0.5 h at the same temperature and then $(HCHO)_n$ (3.0 equiv.) was added at once and further stirring with warming to room temperature over 1 h, and then stirred at rt until completion of the reaction (determined by TLC). Saturated NH_4Cl solution was added to quench the reaction, and ethyl acetate was added. The phases were separated, and the aqueous phase was extracted with CH_2Cl_2 (×2). The combined organic extracts were washed with brine, dried over $MgSO_4$, and concentrated *in vacuo*. The residue was purified by flash column chromatography (PE:EA = 5:1 to 3:1), unless otherwise specified, to give the corresponding alcohols.

1.2.2 Preparation of oxindole substituted heteroaryl ethers

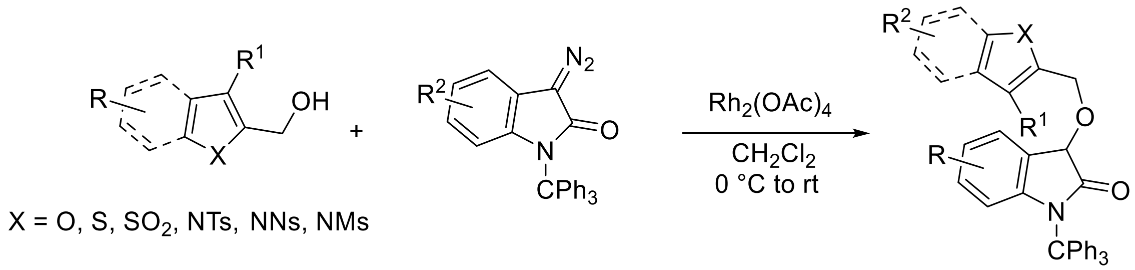

General Procedure C:

To a stirred solution of appropriate heteroaryl alcohol (1.0 equiv.) in CH_2Cl_2 (0.05 M) was added $Rh_2(OAc)_4$ (0.005 equiv.) at 0 °C. The appropriate diazo compound (1.1 equiv.) was added at once, the reaction mixture was allowed to warm to rt and stirred for 1-4 h. After the consumption of the starting material was determined by TLC, the mixture was concentrated under reduced pressure to give the crude product, which was further purified by flash column chromatography (eluent: Hexane/EtOAc = 5:1 to 4:1) to give the final product.

1.2.3 BIMP-catalyzed rearrangement reaction.

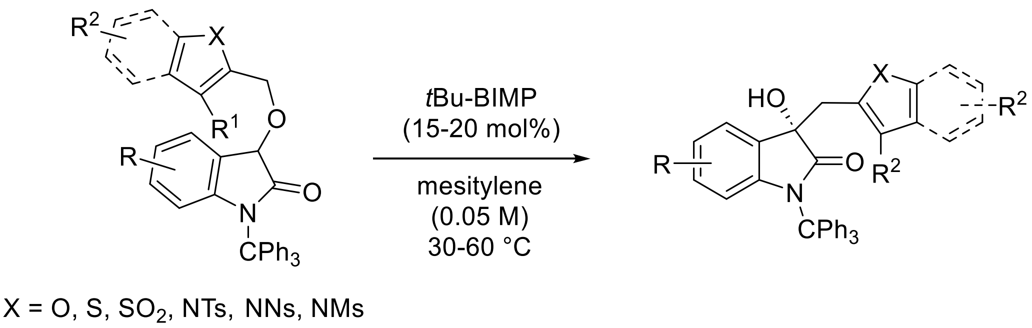

General Procedure D:

The appropriate ether substrate (0.1 mmol) and *t*Bu-BIMP (0.015-0.020 mmol) were added to a flame-dried Schlenk tube and the tube was flushed with N_2 three times. Mesitylene (0.05 M) was added through the septum under a positive pressure of N_2. The reaction was stirred at 30-60 °C until completion as indicated by TLC analysis. The reaction mixture was concentrated under reduced pressure to give the crude product, which was purified by flash column chromatography (eluent: hexane/EtOAc = 4:1 to 3:1).

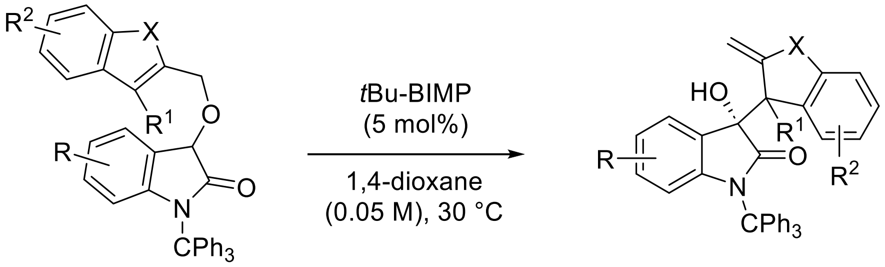

General Procedure E:

The appropriate ether substrate (0.1 mmol) and *t*Bu-BIMP (0.005 mmol) were added to a flame-dried Schlenk tube and the tube was flushed with N_2 three times. 1,4-Dioxane (0.05 M) was added through the septum under a positive pressure of N_2. The reaction was stirred at 30 °C until completion as indicated by TLC analysis. The reaction mixture was concentrated under reduced pressure to give the crude product, which was purified by flash column chromatography (eluent: hexane/acetone = 5:1 to 3:1). *Note: the temperature of the water bath for rotary evaporator should be kept as low as possible (below 30 °C) due to the instability of the [2,3]-rearrangement products.*

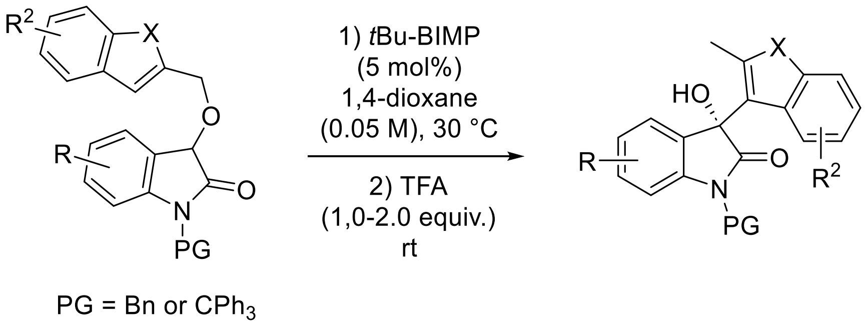

General Procedure F:

The appropriate ether substrate (0.1 mmol) and *t*Bu-BIMP (0.005 mmol) were added to a flame-dried Schlenk tube and the tube was flushed with N_2 three times. 1,4-Dioxane (0.05 M) was added through the septum under a positive pressure of N_2. The reaction was stirred at 30 °C until completion as indicated by TLC analysis, then TFA (1.0-2.0 equiv.) was added at once and stirred another 12-24 h until completion. The reaction mixture was concentrated under reduced pressure to give the crude product, which was purified by flash column chromatography (eluent: hexane/acetone = 5:1 to 3:1).

Note: all the racemic products were prepared employing the same procedures as enantioenriched products with racemic catalyst used instead.

2 Computational Studies

2.1 Computational Details

DFT calculations were performed using ORCA 5.0.4[1]; geometries were fully optimised with M06-2X/def2-SVP.[2,3] Implicit solvation was also included with the CPCM solvation model[4] employing the parameters of THF. Convergence criteria were tightened with 'TightSCF' and 'TightOpt' and a finer grid 'DefGrid3' was used due to the sensitivity of the Minnesota functionals towards the grid size.[5–7] The nature of minima and transition states were verified by the computation of harmonic frequencies at the same level of theory. Single-point energies were evaluated with the same M06-2X functional and a larger def2-TZVP basis, including implicit solvation. Thermochemistry was evaluated at 1 atm and 298.15 K using thermodynamic corrections from the level of geometry optimisation, in combination with energetics obtained from single-point calculations.

Conformational flexibility for minima was considered with a meta-dynamics conformational search with CREST 2.12[8] using GFN2-xTB 6.2[9]. An energy window ('ewin') of 8 kcal/mol was used and implicit solvation was also included through the analytical linearised Poisson-

Boltzmann (ALPB) model[10] with the parameters of THF. The lowest GFN2-xTB energy structure was then optimised fully with DFT (while this may bias structures with overstabilised intramolecular interactions, especially π-π contacts, the minima are not crucial for the accurate prediction of stereocontrol).

Conformational flexibility for the key [2,3]-transition state was extensively considered to ensure that the computed selectivity was an accurate representation of the experimental selectivity. Initially, the [2,3]-transition state was located for the isolated anionic system (without BIMP) for both endo- and exo-transition states before the full *t*-Bu BIMP catalyst was added to the system to create 4 diastereomeric geometries.

A meta-dynamics conformational search with CREST 2.12[8] using GFN2-xTB 6.2[9] was performed for each of the 4 systems with an energy window ('ewin') of 10 kcal/mol. An ellipsoidal potential was included through the 'NCI' option, to ensure the two fragments did not fully dissociate from the non-covalent ensemble. Solvation was also included through the (ALPB) model[10] with the parameters of THF. Atoms involved in (and connected to) the [2,3]-transition state (Scheme **S1C**) were constrained in the geometry of the (isolated) TS with a force constant of 0.5 to ensure that the respective energy rankings are not too heavily influenced by changes in the bonding of the substrate during the meta-dynamics run. All conformers were then subject to a GFN2-xTB transition state search interfaced through ORCA 5.0.4[11] with tightened convergence criteria using keywords 'TightSCF' and 'TightOpt' and implicit solvation was included with ALPB(THF). A frequency calculation was performed at this level of theory to verify the location of a TS with one single imaginary mode.[*]

The single imaginary mode was analysed by displacement of the TS mode in both the 'forward' and 'reverse' directions using the *pyQRC 1.03* tool by Robert Paton[12] with a displacement amplitude of '0.5'. The RMSD of the core atoms involved in the [2,3]-rearrangement (Scheme **S1C**) was calculated between the two displaced geometries and where this significantly different from zero (>0.05), the TS was kept for subsequent single point analysis. This removed any conformers that had not optimised to the desired [2,3]-transition state.

Subsequent DFT single-point calculations were then performed on *all* relevant GFN2-xTB transition states to improve the energetic description of the system. These were performed with

[*] small imaginary modes were eliminated with displacement of the geometry along the 2nd imaginary mode using *pyQRC 1.03*[12] with an amplitude of '0.2' and convergence criteria was tightened with 'VeryTightOpt'

M06-2X/def2-SVP[2,3] using Gaussian16, C.01.[13] Implicit solvation was included through the SMD[14] solvent model with the parameters of THF and the default "UltraFine" grid.†

Following this energetic analysis of the conformers, the 5 lowest conformers of each diastereomer were taken forward to perform full DFT transition state calculations through ORCA as described above.

Energies were extracted using *GoodVibes 3.2*[15,‡] and quasi-rigid-rotor entropies were evaluated using the Grimme method with a frequency cutoff of 100 cm^{-1}, with any small imaginary modes included in the partition functions by changing the sign of the wavenumber.

Additional Martin, Hay, Pratt empirical entropic corrections were included (S_{MHP} = 3.38 kcal/mol per particle, evaluated at 302 atm to mimic bulk THF)[16] to account for the restriction by the solvent on translational and rotational degrees of freedom.

Visualisation of structures was performed using CYLview20[17], GaussView6.1.1[13] and PyMOL[18], non-covalent interactions were computed using NCIPlot 4.0.[19]

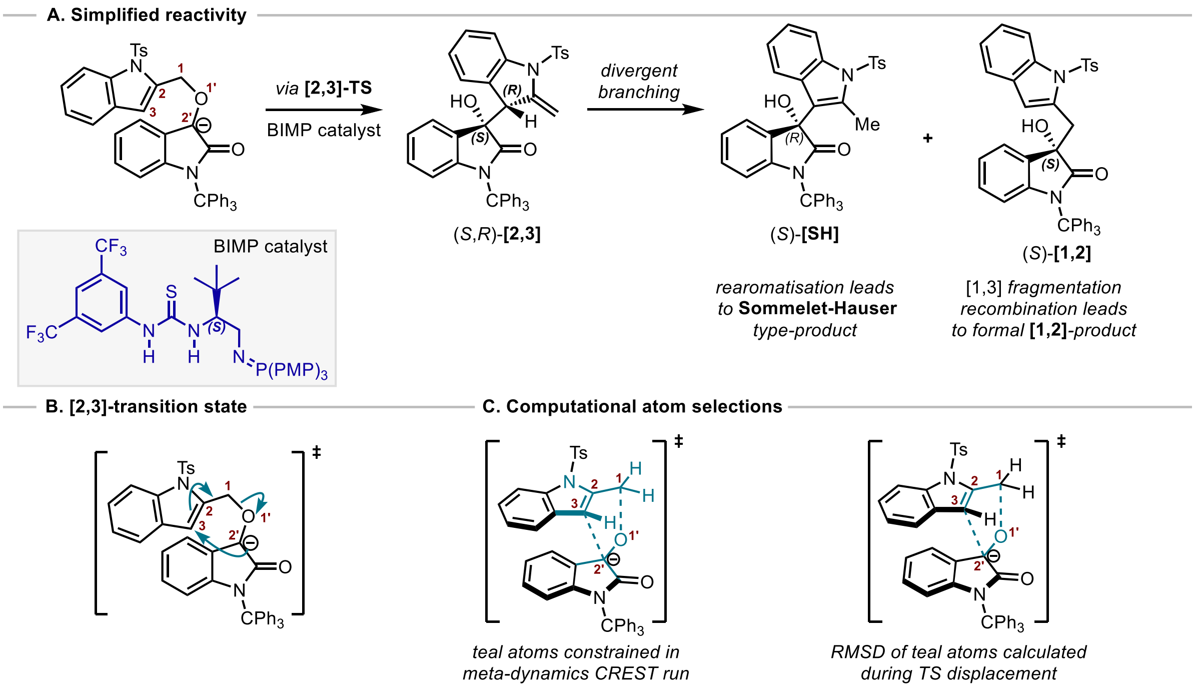

Scheme S1 A: Reactivity scheme. B: [2,3]-transition state. C: Atom selections for constraints and RMSD calculations.

† Single-point calculations were performed with Gaussian before moving to ORCA for methodological reasons due to the size of the system.

‡ Implementation was added for the parsing of ORCA outputs.

2.2 Computational Discussion

Computationally, truncating the BIMP catalyst $(PMP)_3$ and the substrate trityl groups would save resources; however, these groups are likely to be important steric bulk that help to set the relative orientations of the substrate and catalyst.

There were many small imaginary modes that were challenging to remove, associated with the tosyl methyl group and more general phonon vibrations of the large non-covalent complex. Tosyl groups are known to have very small barriers to methyl rotation and truncation to $-SO_2Ph$ would have eliminated this problem. These were removed where possible with tightened optimisation criteria ('VeryTightOpt') and displacement along the unwanted imaginary mode; however, in many cases after reoptimisation and tightened convergence criteria the small imaginary modes could not be removed and due to the expense of the optimisation and frequency calculations, these were not resolved.

The energy associated with such a rotation of *p*-Me groups is very low[20–22] and will have a negligible impact on the electronic energies at the transition state. However, the presence of an additional imaginary mode would reduce the degrees of freedom to $3N-8$, introducing variation in the computed thermochemistry. Both Gaussian and ORCA jobs ignore the presence of additional imaginary modes and compute the thermochemistry using only the real modes; however, this can lead to an inconsistent number of vibrational modes included if some conformers have additional imaginary modes.

The *GoodVibes* program has been used in this work to include *quasi-harmonic* approximations from Grimme[23,§] with inversion of the small imaginary modes in an attempt to improve upon the poor description of low-energy vibrational modes from the rigid-rotor harmonic oscillator and reduce dependence on the computed wavenumber of these small imaginary modes.

2.3 IRC Traces

Following the work of Singleton[25] on competitive [2,3]- and [1,2]-rearrangements of ammonium ylides, we locate a [2,3]-transition state that is primarily associated with the cleavage of the C–O ether bond, analogous to the C–N cleavage, with a 'loose' C–C interaction, transpiring from tracing the intrinsic reaction coordinate (IRC, **Figure S1**). This mode connects the reactant to the [2,3]-product and involves an early C–O cleavage, followed by a later C–C bond formation process.

§ This quasi-rigid rotor approximation is also included by default in ORCA.

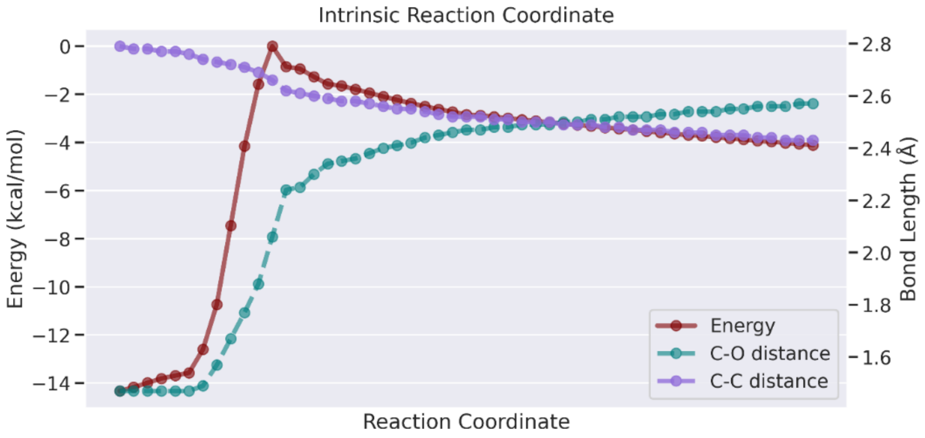

Figure S1 IRC path of (*S*,*R*)_0070, the most stable transition state.

2.4 Conformational Analysis

The energetics of the different levels of theory give an indication of the relative accuracies in terms of diastereomer prediction, though this is a challenging target looking at small energetic differences and semi-empirical methods may not be expected to perform well. The GFN2-xTB energies do not give an accurate reflection of the most stable diastereomer; however, the description improves with the inclusion of thermodynamic corrections to obtain the free energies. DFT single-point calculations further improve the description and identify the correct major diastereomer.

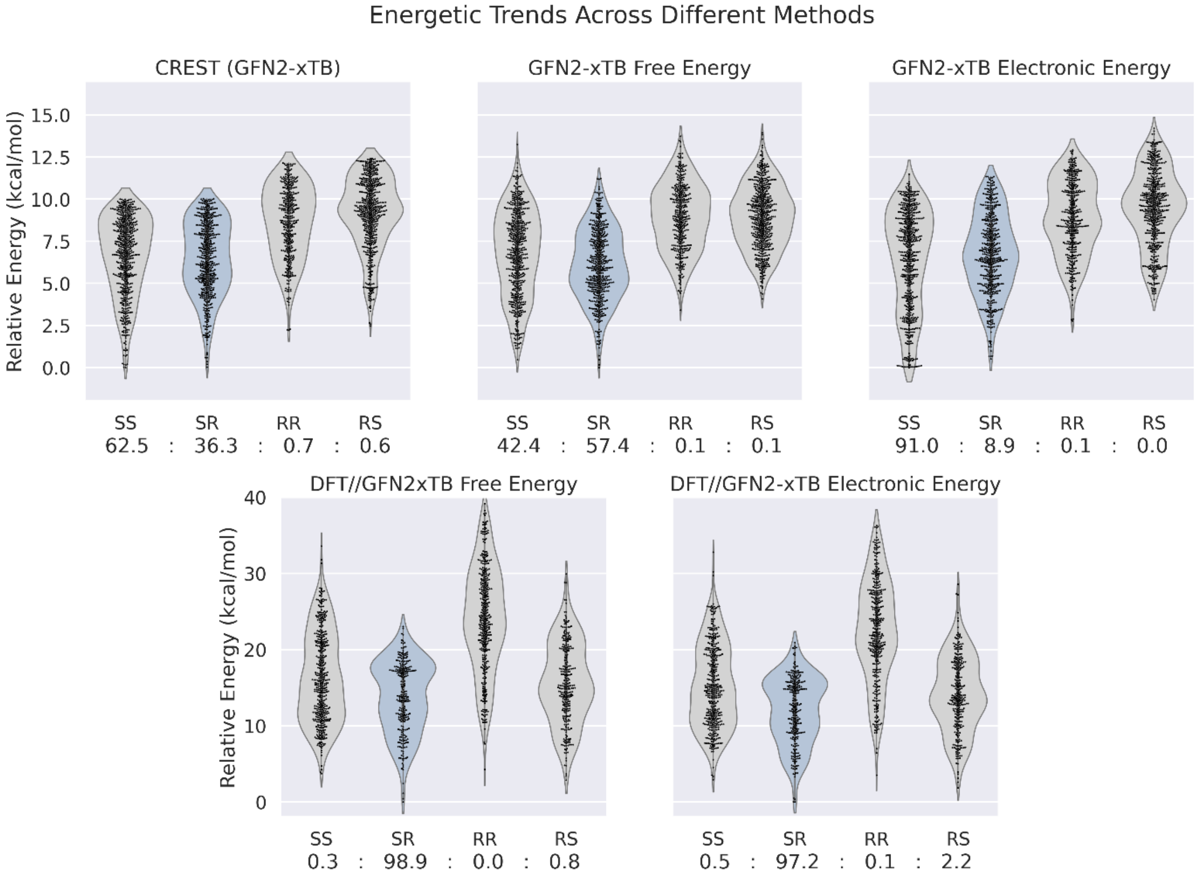

Figure S2 Comparison of the distribution of relative energies of the transition states across the conformational ensemble including Boltzmann populations for each diastereomer averaged over the whole ensemble. Individual energies represented by dots, with the violin plot indicating the distribution across the range of energies. The most stable diastereomer (absolute configuration from the crystal structure) is highlighted in blue.

This method of conformational pruning based on the single-point energies eliminates the poor energetic description of non-covalent interactions, for example, the overstabilisation of π-π interactions by GFN2-xTB.[26] However, the method relies on broadly accurate GFN2-xTB geometries, that are sufficiently similar to the DFT geometries such that the potential energy surface (PES) is not significantly distorted relative to the DFT PES.

The DFT optimised geometries were compared to the GFN2-xTB optimised geometries, and there was some RMSD change overall, with the most deviations arising from changes to the key transition state region (**Figure S3**). This has already been identified by Daniel Singleton to be a challenge across different DFT methodologies due to the "loose" nature of the transition states.[25] GFN2-xTB significantly underestimates the length of the C–C bond, with >15% deviation in this bond length. Contrastingly, GFN2-xTB overestimates the length of the C–O bond, with <10% deviation. This indicates that the transition-state is calculated to be significantly *later* with GFN2-xTB relative to DFT and is more "product-like".

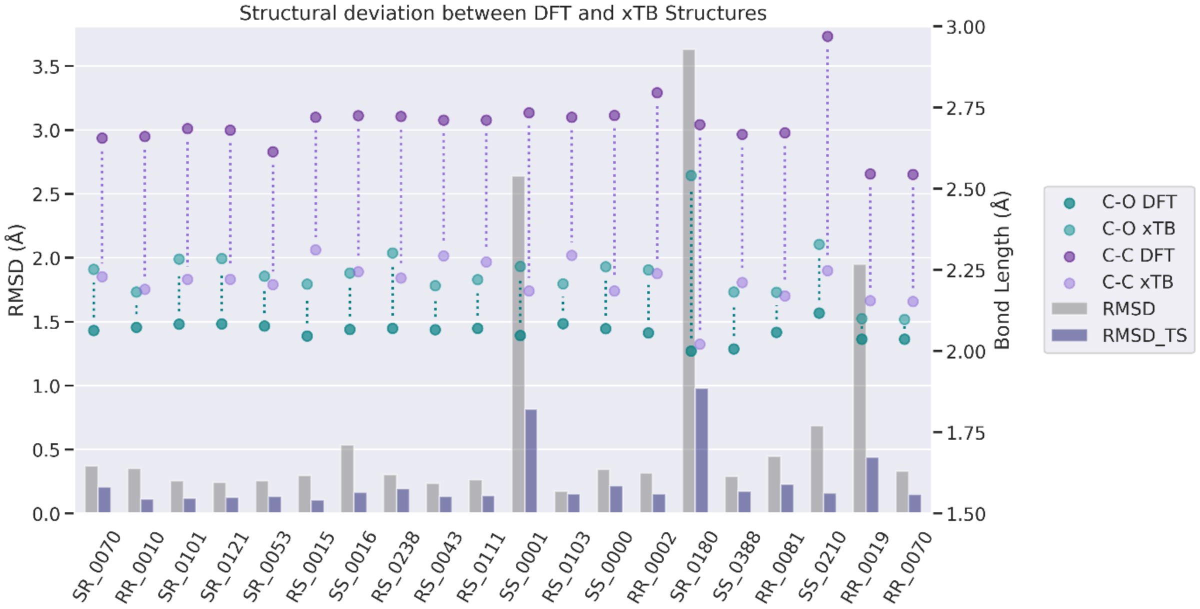

Figure S3 RMSD and key bond distances across the different conformers studied with DFT. RMSD calculated over all atoms, RMSD_TS only over the five atoms involved in the TS. Labels correspond to the energetic ranking of structures as output from CREST and the structures are ranked by DFT electronic energies (**Figure S4** top left).

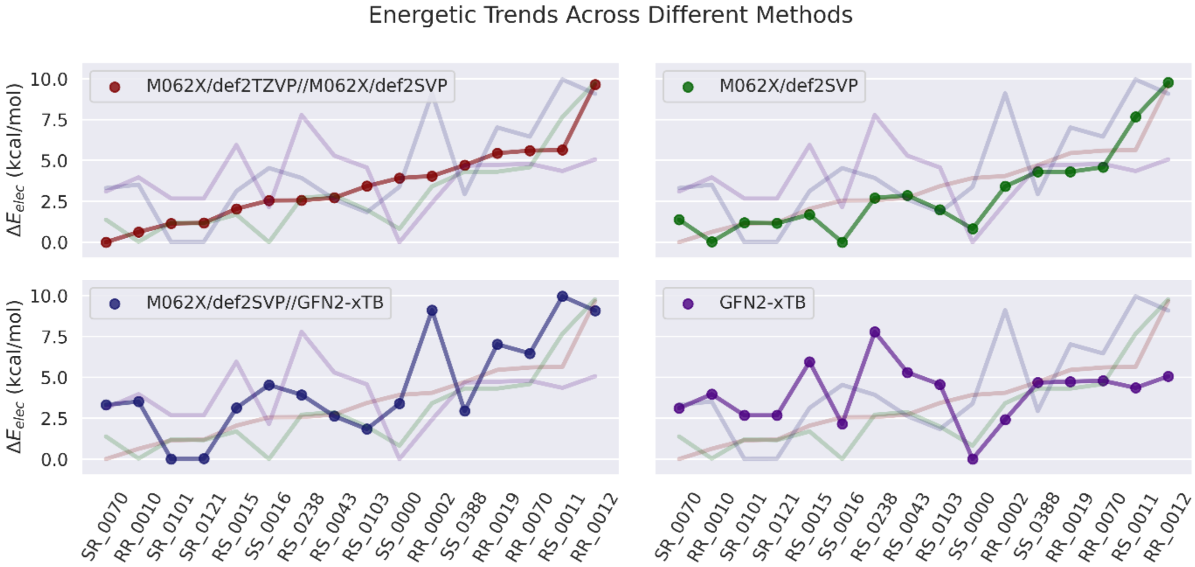

Figure S4 Comparison of electronic energies across the DFT optimised structures relative to the most stable at each level of theory).

The DFT electronic energies from optimisation would lead to the wrong diastereomer prediction, with subsequent single-point calculations identifying a transition state that leads to the experimentally observed product.

2.5 Strain Analysis

To gain insight into the selectivity, we have considered the relative strain and interaction of the system by comparing the gas-phase energy of both the substrate and the BIMP catalyst in the

geometry of the TS. This follows the activation-strain model whereby interaction and reorganisation energies are calculated by fragmenting the substrate and BIMP organocatalyst.[27] Single-point energies of the TS and each fragment (in the geometry of the TS) were computed in the gas-phase to prevent artificial stabilisation due to changes in the solvent accessible surface areas compared to the TS. Due to the changes in charges on going from the fully relaxed reactants to the corresponding fragments of the [2,3]-TS, the relative energies of the fragmented components were compared across conformers to examine the relative stabilities of each component rather than comparing to an optimised minimum (relative to the most stable TS).

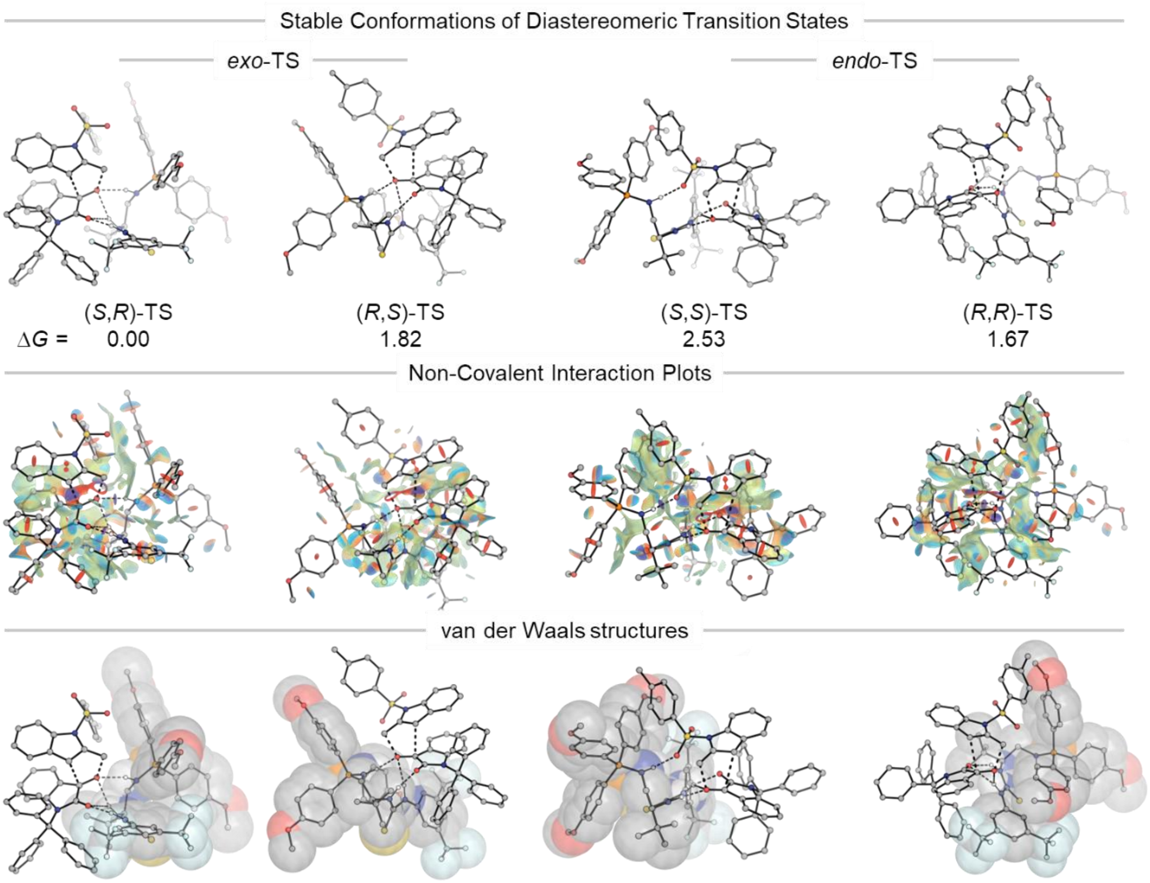

Figure S5 Most stable conformations of each of the four diastereomeric TSs.

The geometry of the substrate in the enantiomeric (*S*,*R*)- and (*R*,*S*)-TS are significantly favoured relative to the (*S*,*S*)- and (*R*,*R*)-TS (**Figure S6**, middle). These proceed through formally an *exo*-TS which is more stable than the *endo*-TS. The *exo*-TS is stabilised by intramolecular π–π interactions of the substrate which can be observed from non-covalent interaction (NCI) plots of the stable diastereomers (**Figure S5**). Additionally, the *endo*-TS has the aromatic ether unit orientated towards the trityl group, leading to a destabilising strain in

the substrate, observable from puckering of the indole N–CPh_3 bond out-of-plane (up to 30°, **Figure S5**).

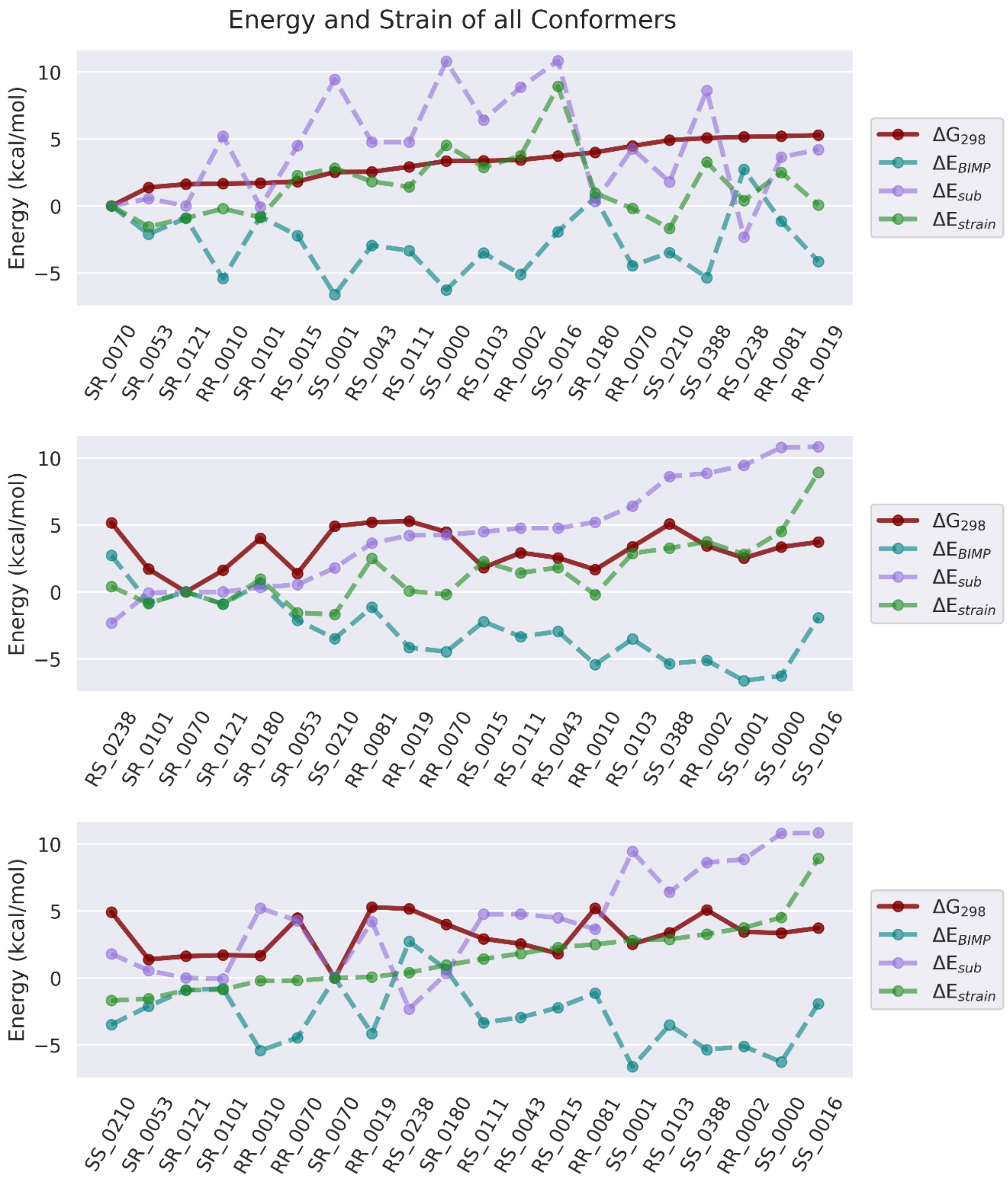

Figure S6 Relative energies across all DFT conformations. Structures are ranked by relative free energy (ΔG), relative energy of substrate fragment (ΔE_{sub}) and relative strain energy ($\Delta E_{strain} = \Delta E_{sub} + \Delta E_{BIMP}$) from top to bottom.

The *exo*- preference appears to drive the formation of both the (*S*,*R*)- and (*R*,*S*)-products through stabilisation of the substrate; however, the BIMP catalyst is less energetically stable in

this conformation, with the lowest energy structures coming from the (*S*,*S*)- and (*R*,*R*)-TS (**Figure S6**).

By comparing the *exo*-TS structures, it appears that the enantiocontrol comes from the different '*fit*' of the substrate and catalyst. Geometrically, the different '*fit*' can be rationalised from the different conformations dominant across the diastereoisomers. In the (*S*,*R*)-TS, the catalyst *t*-Bu group fits '*below*' the oxindole ring of the substrate, whereas in the (*R*,*S*)-TS, the *t*-Bu group is orientated away from the substrate (**Figure S5** top).

The (*R*,*S*)-conformations have a geometry that appears to be either optimised to stabilise the organocatalyst or the substrate at the expense of the other component, leading to an increase in overall strain on the system relative to the low-energy (*S*,*R*)-conformations (**Figure S7**).

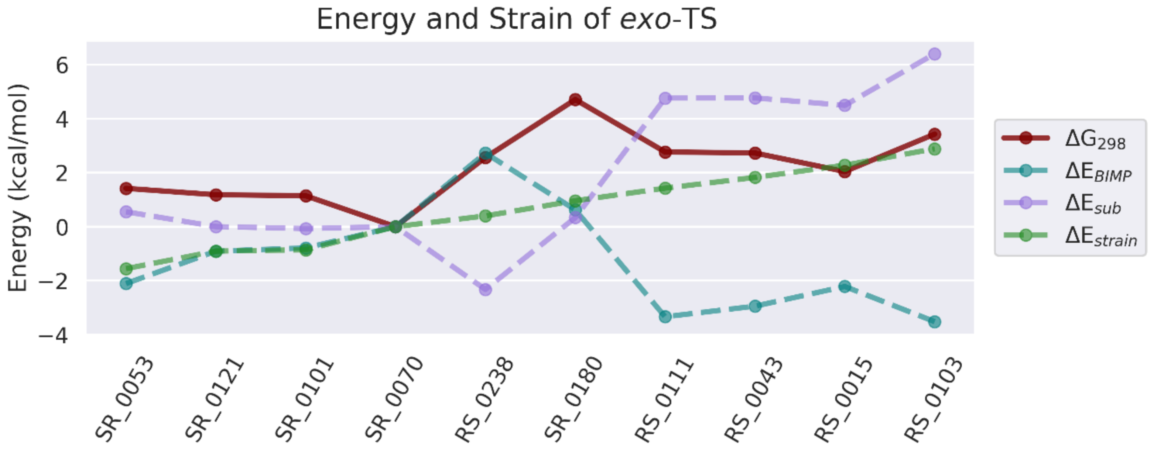

Figure S7 Relative energies across the different conformations of the *exo*-TS. Structures are ranked by relative strain energy ($\Delta E_{strain} = \Delta E_{sub} + \Delta E_{BIMP}$).

Attempts to relate the stabilities of the TSs to the strengths of the hydrogen bonds, involving the thiourea HB donors and the conjugate acid of the iminophosphorane Brønsted base, assessed through the NH bond lengths were inconclusive.

3. Control experiments of [2,3]-rearrangement product 3.

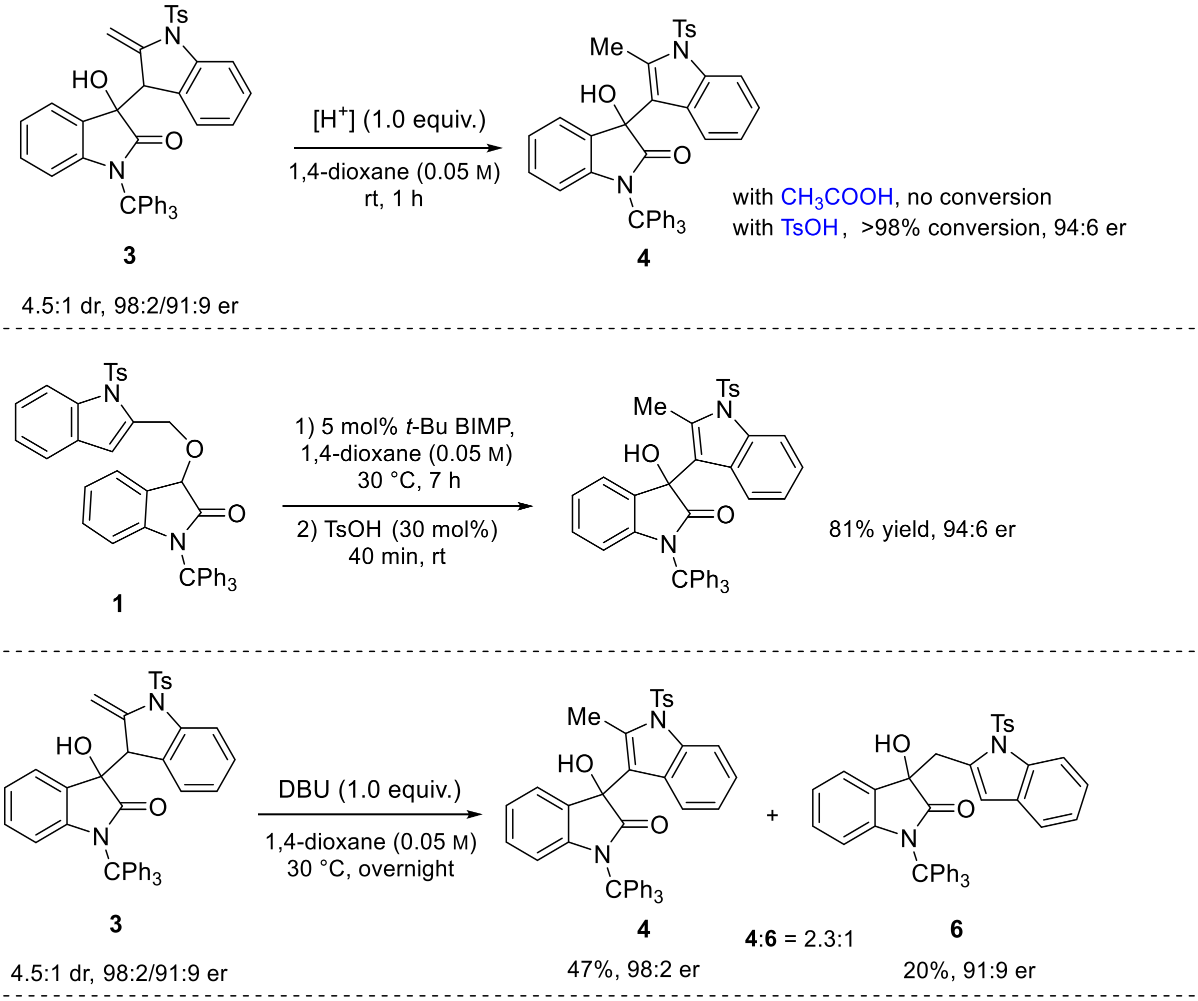

Figure S8 Control experiments

4 Derivatization of rearrangement products.

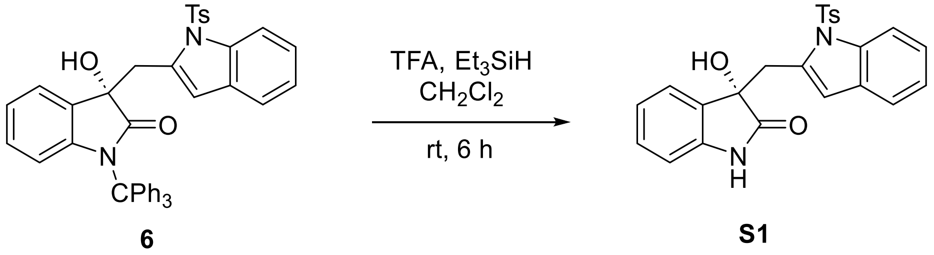

General Procedure G:

To a stirred solution of [1,2]-rearrangement product **6** (0.10 mmol) in CH_2Cl_2 (2 mL) at rt was added Et_3SiH (0.5 mmol) and TFA (1.0 mL). The reaction mixture was stirred at rt for 6 h. After completion was determined by TLC analysis, excess TFA was removed under reduced pressure and the mixture was redissolved in EtOAc (10 mL) and neutralized by sat. aq.

$NaHCO_3$. The mixture was extracted with EtOAc (×4). The combined organic extracts were dried over $MgSO_4$, filtered, and concentrated under reuduced pressure. Further purification by flash column chromatography (eluent: hexane/EtOAc = 3:1 to 1:1) gave the final product.

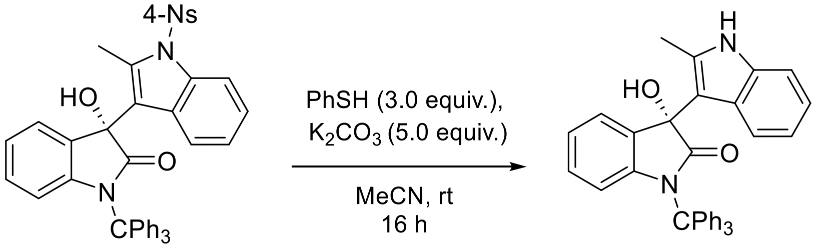

49 96:4 er — **51** 80% yield, 96:4 er

General Procedure H:

To a stirred solution of [2,3]-rearrangement product **3** (0.10 mmol) in MeCN (5 mL) at rt was added PhSH (0.3 mmol) and K_2CO_3 (0.5 mmol). The reaction mixture was stirred at rt for 16 h. After completion was determined by TLC analysis, the mixture was redissolved in EtOAc (20 mL) and water (10 mL). The mixture was extracted with EtOAc (×4) and the combined organic extracts were dried over $MgSO_4$, filtered, and concentrated under reuduced pressure. Further purification by flash column chromatography (eluent: hexane/EtOAc = 3:1 to 1:1) gave the final product.

5. Characterisation of compounds

(*S*)-3-Hydroxy-3-[(1-tosyl-1H-indol-2-yl)methyl]indolin-2-one (S1)

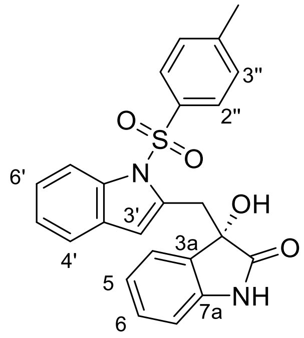

Following **General procedure G**, (*S*)-3-hydroxy-3-[(1-tosyl-1*H*-indol-2-yl)methyl]-1-tritylindolin-2-one (67.4 mg, 0.1 mmol), $SiEt_3H$ (0.5 mmol) and TFA (1.0 mL) in CH_2Cl_2 (2.0 mL) at rt for 6 h gave a crude product, which was purified by flash column chromatography (eluent: hexane/EtOAc = 3:1 to 1:1) to afford the product (38.9 mg, 90%) as a colourless amorphous solid. **IR** ν_{max} (film) 1716 (C=O), 1471, 1450, 1361, 1172 (S=O), 1147, 1089; **$[\alpha]_D^{20}$** = 60.8 (*c* 0.25 in $CHCl_3$); **Chiral HPLC analysis**, Chiralpak IA (80:20 hexane: ^{i}PrOH, flow rate 1 ml·min^{-1}, 211 nm, 30 °C) tR (*S*)-**S1**: 20.3 min, tR (*R*)-**S1**: 22.9 min, 96:4 er; **^{1}H NMR (500 MHz, $CDCl_3$)** δ **2.29** (3H, s, C*H*$_3$), **3.36** (1H, d, *J* 15.1, C*H*^{A}H^B), **3.98** (1H, d, *J* 15.4, CH^A*H*^{B}), **4.21** (1H, s, O*H*), **6.48** (1H, s, ArC(3`)*H*), **6.88** (1H, d, *J* 7.7, ArC(7)*H*), **6.91–6.99** (2H, m, ArC(4,5)*H*), **7.12** (2H, d, *J* 7.8, ArC(3``,5``)*H*), **7.22** (2H, dd, *J* 10.6, 4.9, ArC(6,5`)*H*), **7.28** (1H, t, *J* 7.8, ArC(6`)H), **7.42** (1H, d, *J* 7.8, ArC(4`)*H*), **7.56** (2H, d, *J* 7.4, ArC(2``,6``)*H*), **8.15** (1H, d, *J* 8.3, ArC(7`)*H*), **8.19** (1H, d, *J* 8.1, N*H*); **^{13}C{^{1}H} NMR (126 MHz,**

CDCl₃) δ$_C$: **21.7** (*C*H$_3$), **36.3** (*C*H$_2$), **76.1** (*C*(3)), **110.5** (*C*(7)H), **114.2** (Ar*C*(3`)H), **115.5** (Ar*C*(7`)H), **120.8** (Ar*C*(4`)H), **122.9** (Ar*C*(5)H), **124.0** (Ar*C*(5`)H), **124.7** (Ar*C*(6`)H), **125.5** (Ar*C*(4)H), **126.4** (Ar*C*(2``,6``)H), **129.7** (Ar*C*(3a)), **129.7** (Ar*C*(3a`)), **129.9** (Ar*C*(6)H), **130.0** (Ar*C*(3``,5``)H), **134.3** (Ar*C*(2`)), **135.4** (Ar*C*(1``)), **137.5** (Ar*C*(7a`)), **140.3** (Ar*C*(7a)), **145.1** (Ar*C*(4``)), **179.8** (*C*=O). **HRMS (ESI$^+$)** $C_{24}H_{20}N_2O_4SNa$ [M+Na]$^+$ found 455.1052, requires 455.1036 (–3.51 ppm).

Ethyl 5-chloro-1-tosyl-1*H*-indole-2-carboxylate (S2)

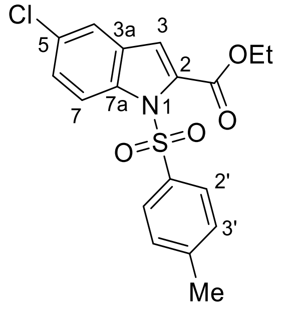

Following **General Procedure A**, **step I**, ethyl 5-chloro-1*H*-indole-2-carboxylate (2.23 g, 10.0 mmol), sodium hydride (60% dispersion in mineral oil, 0.60 g, 15.0 mmol), and tosyl chloride (2.85 g, 15.0 mmol) in DMF (20.0 mL) were stirred overnight to give, after purification by column chromatography (eluent: Hexane/EtOAc = 7:1 to 3:1), the title compound (3.24 g, 86%) as a pale-yellow solid. **mp** 59–61 °C; **IR** ν$_{max}$ (film) 1728 (C=O), 1695, 1541, 1440, 1359, 1192, 1163 (S=O), 1126; **^{1}H NMR (500 MHz, CDCl$_3$)** δ **1.40** (3H, t, *J* 7.2, CH$_2$C*H*$_3$), **2.37** (3H, s, C*H*$_3$Ar), **4.41** (2H, q, *J* 6.8, C*H*$_2$CH$_3$), **7.05** (1H, d, *J* 0.8, C(3)*H*), **7.25–7.29** (2H, m, ArC(3`,5`)*H*), **7.36** (1H, dd, *J* 9.2, 2.4, ArC(6)*H*), **7.52** (1H, d, *J* 2.0, ArC(4)*H*), **7.85–7.93** (2H, m, ArC(2`,6`)*H*), **8.04** (1H, dt, *J* 9.2, 0.8, ArC(7)*H*); **^{13}C NMR (126 MHz, CDCl$_3$)** δ **14.2** (CH$_2$*C*H$_3$), **21.7** (*C*H$_3$Ar), **62.3** (*C*H$_2$CH$_3$), **115.2** (*C*(3)H), **116.6** (Ar*C*(7)H), **121.9** (Ar*C*(4)H), **127.2** (Ar*C*(6)H), **127.5** (Ar*C*(2`,6`)H), **129.5** (Ar*C*(5)), **129.8** (Ar*C*(3`,5`)H), **129.9** (*C*(3a)), **133.2** (*C*(2)), **135.4** (Ar*C*(1`)), **136.4** (*C*(7a)), **145.4** (Ar*C*(4`)), **161.1** (*C*=O); **HRMS (ESI$^+$)** $C_{18}H_{16}{}^{35}ClNO_4SNa$ [M+Na]$^+$ found 400.0376, requires 400.0381 (–1.29 ppm).

Ethyl 5-bromo-1-tosyl-1*H*-indole-2-carboxylate (S3)

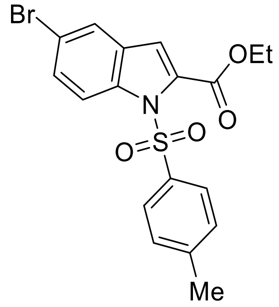

Following **General Procedure A**, **step I**, ethyl 5-bromo-1*H*-indole-2-carboxylate (2.67 g, 10.0 mmol), sodium hydride (60% dispersion in mineral oil, 0.60 g, 15.0 mmol), and tosyl chloride (2.85 g, 15.0 mmol) in DMF (20.0 mL) were stirred overnight to give, after purification by column chromatography (eluent: Hexane/EtOAc = 7:1 to 3:1), the title compound (3.79 g, 90%) as a pale-yellow solid with ^{1}H and ^{13}C spectroscopic data in accordance with the literature;[28] **^{1}H NMR (500 MHz, CDCl$_3$)** δ **1.40** (3H, t, *J* 7.2), **2.38** (3H, s), **4.41** (2H, q, *J* 6.8), **7.05** (1H, d, *J* 0.8), **7.26–7.29** (2H, m), **7.50** (1H, dd, *J* 9.0, 2.0), **7.69** (1H, d, *J* 1.9), **7.86–7.92** (2H, m), **7.99** (1H, dt, *J* 9.2, 0.8, ArC(7)*H*); **^{13}C NMR (101 MHz, CDCl$_3$)** δ **14.2**, **21.8**, **62.3**, **115.1**, **116.9**, **117.5**, **125.0**, **127.5**, **129.8**, **129.9**, **130.0**, **133.1**, **135.4**, **136.7**, **145.4**, **161.1**.

Ethyl 5-methyl-1-tosyl-1*H*-indole-2-carboxylate (S4)

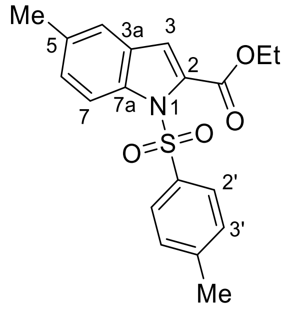

Following **General Procedure A**, **step I**, ethyl 5-chloro-1*H*-indole-2-carboxylate (2.03 g, 10.0 mmol), sodium hydride (60% dispersion in mineral oil, 0.60 g, 15.0 mmol), and tosyl chloride (2.85 g, 15.0 mmol) in DMF (20.0 mL) were stirred overnight to give, after purification by column chromatography (eluent: Hexane/EtOAc = 7:1 to 3:1), the title compound (1.43 g, 40%) as a colorless oil. **IR** ν_{max} (film) 1728 (C=O), 1548, 1359 (S=O), 1340, 1197, 1172, 1147, 1122, 1093; **^{1}H NMR (500 MHz, $CDCl_3$)** δ **1.39** (3H, t, *J* 7.2, CH_2CH_3), **2.36** (3H, s, SO_2ArCH_3), **2.40** (3H, s, $ArCH_3$),**4.40** (2H, q, *J* 6.8, CH_2CH_3), **7.08** (1H, d, *J* 0.8, C(3)*H*), **7.21–7.25** (3H, m, ArC(3`,5`)*H*, ArC(6)*H*), **7.30–7.34** (1H, m, ArC(4)*H*), **7.85–7.90** (2H, m, ArC(2`,6`)*H*), **7.98** (1H, d, *J* 8.6, ArC(7)*H*); **^{13}C NMR (126 MHz, $CDCl_3$)** δ **14.3** (CH_2CH_3), **21.3** ($ArCH_3$), **21.7** (SO_2ArCH_3), **62.0** (CH_2CH_3), **115.2** (Ar*C*(7)H), **116.8** (*C*(3)H), **122.2** (Ar*C*(4)H), **127.4** (Ar*C*(2`,6`)H), **128.6** (Ar*C*(6)H), **128.6** (Ar*C*(3a)), **129.6** (Ar*C*(3`,5`)H), **133.9** (Ar*C*(5)), **133.0** (*C*(2)), **135.7** (Ar*C*(1`)), **136.6** (*C*(7a)), **144.9** (Ar*C*(4`)), **161.6** (*C*=O); **HRMS (ESI$^+$)** $C_{19}H_{19}NO_4SNa$ $[M+Na]^+$ found 380.0923, requires 380.0927 (–1.01 ppm).

Ethyl 5-bromo-1-[(4-nitrophenyl)sulfonyl]-1*H*-indole-2-carboxylate (S5)

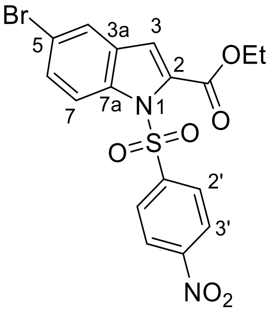

Following **General Procedure A**, **step I**, ethyl 5-bromo-1*H*-indole-2-carboxylate (1.34 g, 5.0 mmol), sodium hydride (60% dispersion in mineral oil, 0.30 g, 7.5 mmol), and 4-nitrobenzenesulfonyl chloride (1.65 g, 15.0 mmol) in DMF (10.0 mL) were stirred overnight to give, after purification by column chromatography (eluent: Hexane/EtOAc = 7:1 to 3:1), the title compound (1.81 g, 80%) as a pale-yellow solid. **IR** ν_{max} (film) 1722 (C=O), 1533 (NO_2), 1371, 1340, 1188, 1168 (S=O), 1126, 1089; **^{1}H NMR (400 MHz, $CDCl_3$)** δ **1.39** (3H, t, *J* 7.2, CH_2CH_3), **4.37** (2H, q, *J* 7.1, CH_2CH_3), **7.17** (1H, d, *J* 0.8, C(3)*H*), **7.58** (1H, dd, *J* 9.0, 2.0, ArC(6)*H*), **7.75** (1H, d, *J* 1.8, ArC(4)*H*), **8.04** (1H, d, *J* 9.0, ArC(7)*H*), **8.18–8.26** (2H, m, ArC(2`,6`)*H*), **8.31–8.39** (2H, m, ArC(3`,5`)*H*); **^{13}C NMR (101 MHz, $CDCl_3$)** δ **14.3** (CH_2CH_3), **62.5** (CH_2CH_3), **116.6** (*C*(3)H), **116.7** (Ar*C*(7)H), **118.2** (Ar*C*(5)Br), **124.4** (Ar*C*(3`,5`)H), **125.5** (Ar*C*(3)H), **128.9** (Ar*C*(2`,6`)H), **129.8** (Ar*C*(3a)), **130.7** (Ar*C*(6)H), **132.6** (*C*(2)), **137.1** (Ar*C*(7a)), **144.3** (Ar*C*(1`)), **150.8** (Ar*C*(4`)NO_2), **160.5** (*C*=O); **HRMS (ESI$^+$)** $C_{17}H_{13}{}^{79}BrN_2O_6SNa$ $[M+Na]^+$ found 474.9564, requires 474.9570 (–1.26 ppm).

(4-Methyl-1-tosyl-1*H*-indol-2-yl)methanol (S6)

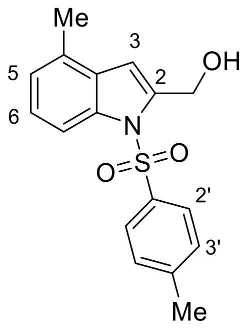

Following **General Procedure B**, 4-methyl-1*H*-indole (1.31 g, 10.0 mmol), sodium hydride (60% dispersion in mineral oil, 0.60 g, 15.0 mmol), and tosyl chloride (2.85 g, 15.0 mmol) in DMF (20.0 mL) were stirred overnight, the obtained tosyl protected indole was treated with *n*-BuLi (1.1 equiv.) and $(HCHO)_n$ (3.0 equiv.) to give, after purification by column chromatography (eluent: Hexane/EtOAc = 5:1 to 3:1), the title compound (1.89 g, 60% (2 steps)) as a pale-yellow oil. **IR** ν_{max} (film) 3275 (OH); **^{1}H NMR (500 MHz, $CDCl_3$)** δ **2.33** (3H, s, ArC(4`)C$H_3$), **2.44** (3H, s, ArC(4)C$H_3$), **3.08** (1H, brs, O*H*), **4.92** (2H, s, C$H_2$OH), **6.68** (1H, s, C(3)*H*), **7.02** (1H, d, *J* 7.3, ArC(5)*H*), **7.17–7.23** (3H, m, ArC(6,3`,5`)*H*), **7.71** (2H, d, *J* 8.3, ArC(2`,6`)*H*), **7.88** (1H, d, *J* 8.5, ArC(7)*H*). **$^{13}$C NMR (126 MHz, $CDCl_3$)** δ **18.5** (ArC(4)*C*H$_3$), **21.7** (ArC(4`)CH_3), **58.8** (*C*H$_2$), **109.8** (*C*(3)H), **112.0** (Ar*C*(7)H), **124.3** (Ar*C*(5)H), **125.2** (Ar*C*(6)H), **126.6** (Ar*C*(2`,6`)H), **128.8** (Ar*C*(4)H), **130.1** (Ar*C*(3`,5`)H), **130.9** (Ar*C*(3a)), **135.9** (Ar*C*(1`)), **137.0** (*C*(2)H), **139.7** (ArC(7a)), **145.2** (Ar*C*(4`)); **HRMS (ESI$^+$)** $C_{17}H_{17}NO_3SNa$ $[M+Na]^+$ found 338.0824, requires 338.0821 (0.78 ppm).

(5-Chloro-1-tosyl-1*H*-indol-2-yl)methanol (S7)

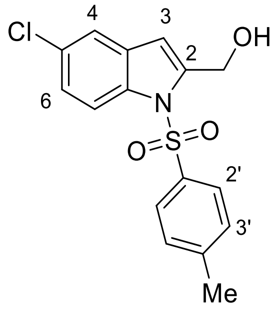

Following **General Procedure A**, **step II**, ethyl 5-chloro-1*H*-indole-2-carboxylate (2.23 g, 10.0 mmol), sodium hydride (60% dispersion in mineral oil, 0.60 g, 15.0 mmol), and tosyl chloride (2.85 g, 15.0 mmol) in DMF (20.0 mL) were stirred overnight, the obtained tosyl protected indole was treated with DIBAL-H (2.5 equiv.) to give, after purification by column chromatography (eluent: Hexane/EtOAc = 5:1 to 3:1), the title compound (2.50 g, 75% (2 steps)) as a colourless oil. **IR** ν_{max} (film) 3277 (OH); **^{1}H NMR (400 MHz, $CDCl_3$)** δ **2.34** (3H, s, CH_3Ar), **2.79** (1H, br, O*H*), **4.89** (2H, s, CH_2OH), **6.58** (1H, s, C(3)*H*), **7.18–7.23** (2H, m, ArC(3`,5`)*H*), **7.23–7.26** (1H, m, ArC(6)*H*), **7.44** (1H, d, *J* 2.1, ArC(4)*H*), **7.66–7.72** (2H, m, ArC(2`,6`)*H*), **7.94–8.01** (1H, d, *J* 8.8, ArC(7)*H*); **^{13}C NMR (101 MHz, $CDCl_3$)** δ **21.7** (Ar*C*H$_3$), **58.6** (*C*H$_2$), **110.5** (*C*(3)H), **115.5** (Ar*C*(7)H), **120.9** (Ar*C*(4)H), **125.3** (Ar*C*(6)H), **126.5** (Ar*C*(2`,6`)H), **129.6** (Ar*C*(5)Cl), **130.2** (Ar*C*(3`,5`)H), **130.5** (Ar*C*(3a)), **135.3** (*C*(2)H), **135.4** (Ar*C*(1`)), **141.7** (ArC(7a)), **145.6** (Ar*C*(4`)); **HRMS (ESI$^+$)** $C_{16}H_{14}{}^{35}ClNO_3SNa$ $[M+Na]^+$ found 358.0275, requires 358.0276 (–0.17 ppm).

(5-Bromo-1-tosyl-1*H*-indol-2-yl)methanol (S8)

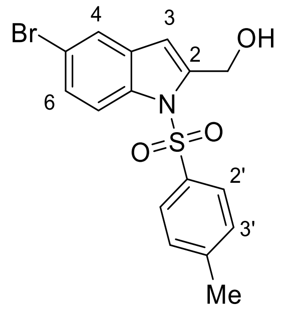

Following **General Procedure A**, **step II**, ethyl 5-bromo-1*H*-indole-2-carboxylate (2.67 g, 10.0 mmol), sodium hydride (60% dispersion in mineral oil, 0.60 g, 15.0 mmol), and tosyl chloride (2.85 g, 15.0 mmol) in DMF (20.0 mL) were stirred overnight, the obtained tosyl protected indole was treated with DIBAL-H (2.5 equiv.) to give, after purification by column chromatography (eluent: Hexane/EtOAc = 5:1 to 3:1), the title compound (2.92 g, 77% (2 steps)) as a pale-yellow oily solid. **IR** ν_{max} (film) 3290 (OH); **^{1}H NMR (500 MHz, $CDCl_3$)** δ **2.34** (3H, s, CH_3Ar), **3.06** (1H, t, *J* 7.3, O*H*), **4.90** (2H, d, *J* 7.3, CH_2OH), **6.57** (1H, d, *J* 0.5, C(3)*H*), **7.19–7.24** (2H, m, ArC(3`,5`)*H*), **7.38** (1H, dd, *J* 8.9, 2.0, ArC(6)*H*), **7.59** (1H, d, *J* 1.9, ArC(4)*H*), **7.66–7.70** (2H, m, ArC(2`,6`)*H*), **7.92** (1H, d, *J* 8.9, ArC(7)*H*); **^{13}C NMR (126 MHz, $CDCl_3$)** δ **21.7** (ArCH_3), **58.6** (CH_2), **110.2** (*C*(3)H), **115.9** (Ar*C*(7)H), **117.3** (Ar*C*(5)Br), **123.9** (Ar*C*(4)H), **126.5** (Ar*C*(2`,6`)H), **127.9** (Ar*C*(6)H), **130.2** (Ar*C*(3`,5`)H), **131.0** (Ar*C*(3a)), **135.5** (Ar*C*(1`)), **135.9** (*C*(2)H), **141.7** (ArC(7a)), **145.6** (Ar*C*(4`)); **HRMS (ESI$^+$)** $C_{16}H_{14}{}^{79}BrNO_3SNa$ $[M+Na]^+$ found 401.9764, requires 401.9770 (–1.49 ppm).

(5-Methyl-1-tosyl-1*H*-indol-2-yl)methanol (S9)

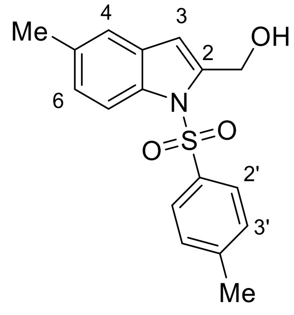

Following **General Procedure A**, **step II**, ethyl 5-methyl-1*H*-indole-2-carboxylate (2.03 g, 10.0 mmol), sodium hydride (60% dispersion in mineral oil, 0.60 g, 15.0 mmol), and tosyl chloride (2.85 g, 15.0 mmol) in DMF (20.0 mL) were stirred overnight, the obtained tosyl protected indole was treated with DIBAL-H (2.5 equiv.) to give, after purification by column chromatography (eluent: Hexane/EtOAc = 5:1 to 3:1), the title compound (0.95 g, 30% (2 steps)) as a colourless oil. **IR** ν_{max} (film) 3419 (OH); **^{1}H NMR (500 MHz, $CDCl_3$)** δ **2.32** (3H, s, CH_3ArSO_2), **2.39** (3H, s, CH_3C(5)Ar), **3.16** (1H, t, *J* 7.3, O*H*), **4.89** (2H, d, *J* 7.2, CH_2OH), **6.56** (1H, d, *J* 0.5, C(3)*H*), **7.11** (1H, dd, *J* 8.6, 1.8, ArC(6)*H*), **7.18** (2H, d, *J* 8.3, ArC(3`,5`)*H*), **7.25** (1H, s, ArC(4)*H*), **7.69** (2H, d, *J* 8.4, ArC(2`,6`)*H*), **7.93** (1H, d, *J* 8.9, ArC(7)*H*); **^{13}C NMR (126 MHz, $CDCl_3$)** δ **21.3** (CH_3C(5)Ar), **21.6** (CH_3ArSO_2), **58.7** (CH_2), **111.2** (*C*(3)H), **114.2** (Ar*C*(7)H), **121.2** (Ar*C*(4)H), **126.5** (Ar*C*(6)H), **126.5** (Ar*C*(2`,6`)H), **129.5** (Ar*C*(3a)), **130.2** (Ar*C*(3`,5`)H), **133.5** (Ar*C*(5)Me), **135.4** (*C*(2)H), **135.8** (Ar*C*(1`)), **140.4** (ArC(7a)), **145.1** (Ar*C*(4`)); **HRMS (ESI$^+$)** $C_{17}H_{17}NO_3SNa$ $[M+Na]^+$ found 338.0815, requires 338.0821 (–1.88 ppm).

(6-Methyl-1-tosyl-1*H*-indol-2-yl)methanol (S10)

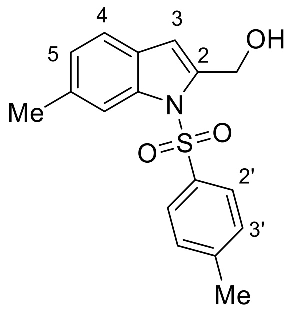

Following **General Procedure B**, 6-methyl-1*H*-indole (1.31 g, 10.0 mmol), sodium hydride (60% dispersion in mineral oil, 0.60 g, 15.0 mmol), and tosyl chloride (2.85 g, 15.0 mmol) in DMF (20.0 mL) were stirred overnight, the obtained tosyl protected indole was treated with *n*-BuLi (1.1 equiv.) and $(HCHO)_n$ (3.0 equiv.) to give, after purification by column chromatography (eluent: Hexane/EtOAc = 5:1 to 3:1), the title compound (2.25 g, 71% (2 steps)) as a pale-yellow oil. **IR** ν_{max} (film) 3527 (OH), 1361, 1163 (S=O), 1141, 1053; **^{1}H NMR (400 MHz, $CDCl_3$)** δ **2.34** (3H, s, CH_3ArSO_2), **2.46** (3H, s, $CH_3C(6)Ar$), **3.14** (1H, t, *J* 7.3, O*H*), **4.87** (2H, d, *J* 7.3, CH_2OH), **6.58** (1H, s, C(3)*H*), **7.05** (1H, d, *J* 7.9, ArC(5)*H*), **7.20** (2H, d, *J* 8.1, ArC(3`,5`)*H*), **7.35** (1H, d, *J* 7.9, ArC(4)*H*), **7.70** (2H, d, *J* 8.4, ArC(2`,6`)*H*), **7.87** (1H, s, ArC(7)*H*); **^{13}C NMR (101 MHz, $CDCl_3$)** δ **21.7** (CH_3ArSO_2), **22.2** ($CH_3C(6)Ar$), **58.7** (CH_2), **111.4** (*C*(3)H), **114.6** (Ar*C*(7)H), **120.9** (Ar*C*(4)H), **125.4** (Ar*C*(5)H), **126.5** (Ar*C*(2`,6`)H), **126.9** (Ar*C*(3a)), **130.1** (Ar*C*(3`,5`)H), **135.3** (Ar*C*(6)Me), **135.9** (Ar*C*(1`)), **137.6** (*C*(2)H), **139.6** (ArC(7a)), **145.2** (Ar*C*(4`)); **HRMS (ESI$^+$)** $C_{17}H_{17}NO_3SNa$ $[M+Na]^+$ found 338.0816, requires 338.0821 (–1.58 ppm).

(5-Bromo-1-[(4-nitrophenyl)sulfonyl]-1*H*-indol-2-yl)methanol (S11)

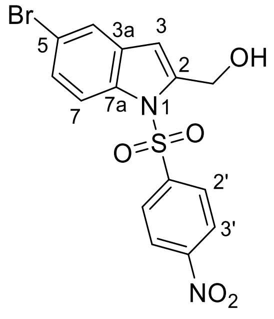

Following **General Procedure A**, **step II**, ethyl 5-bromo-1*H*-indole-2-carboxylate (1.34 g, 5.0 mmol), sodium hydride (60% dispersion in mineral oil, 0.30 g, 7.5 mmol), and 4-nitrobenzenesulfonyl chloride (1.65 g, 7.5 mmol) in DMF (10.0 mL) were stirred overnight, the obtained 4-nitrophenyl sulfonyl protected indole was treated with DIBAL-H (2.5 equiv.) to give, after purification by column chromatography (eluent: Hexane/EtOAc = 5:1 to 3:1), the title compound (0.98 g, 48% (2 steps)) as a yellow solid. **IR** ν_{max} (film) 1544 (NO_2), 1442, 1379, 1350, 1220, 1170 (S=O), 1147, 1087; **^{1}H NMR (500 MHz, Acetone-*d*$_6$)** δ **4.64** (1H, t, *J* 6.0, O*H*), **4.99–5.05** (2H, m, CH_2OH), **6.80–6.88** (1H, m, ArC(3)*H*), **7.46** (1H, dd, *J* 8.8, 2.1 ArC(6)*H*), **7.74** (1H, d, *J* 2.0, ArC(4)*H*), **8.05** (1H, d, *J* 8.9, ArC(7)*H*), **8.27–8.33** (2H, m, ArC(2`,6`)*H*), **8.35–8.42** (2H, m, ArC(3`,5`)*H*); **^{13}C NMR (126 MHz, Acetone-*d*$_6$)** δ **58.6** (CH_2OH), **110.5** (*C*(3)H), **116.7** (Ar*C*(7)H), **117.9** (Ar*C*(5)Br), **124.6** (Ar*C*(4)H), **125.6** (Ar*C*(3`,5`)H), **128.3** (Ar*C*(6)H), **129.4** (Ar*C*(2`,6`)H), **132.6** (*C*(2)), **136.4** (Ar*C*(3a)), **144.0** (Ar*C*(1`)), **144.4** (Ar*C*(7a)), **152.0** (Ar*C*(4`)NO_2), **HRMS (ESI$^+$)** $C_{15}H_{11}{}^{79}BrN_2O_5SNa$ $[M+Na]^+$ found 432.9459, requires 432.9464 (–1.21 ppm).

The following alochols (**S12**[29], **S13**[30], **S14**[31], **S15**[32], **S16**[33], **S17**[34], **S18**[35]) were prepared according to literature procedures.

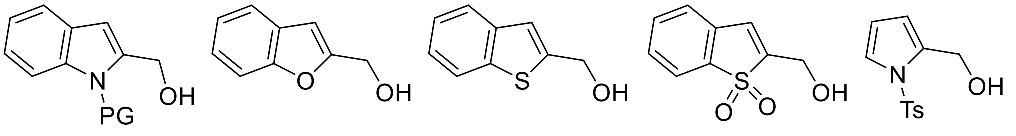

PG = Ts, **S12**
PG = Ms, **S13**
PG = Me, **S14**

S15 **S16** **S17** **S18**

3-[(1-Tosyl-1*H*-indol-2-yl)methoxy]-1-tritylindolin-2-one (S19)

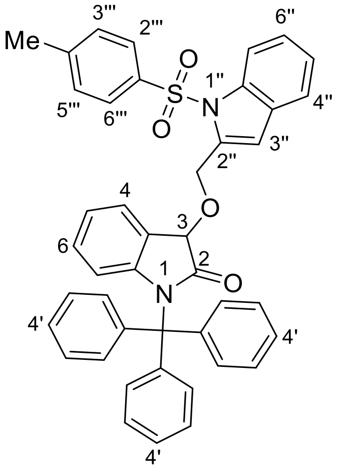

Following **General procedure C**, (1-tosyl-1*H*-indol-2-yl)methanol (0.602 g, 2.0 mmol), 3-diazo-1-tritylindolin-2-one (0.882 g, 2.2 mmol) and $Rh_2(OAc)_4$ (4.41 mg, 0.01 mmol) in CH_2Cl_2 (40 mL, 0.05 M) was added at 0 °C to rt was stirred for 2 h, to give after purification by column chromatography (eluent: Hexane/EtOAc = 5:1 to 3:1), the title compound (0.944 g, 70%) as a pale-yellow solid. **mp** 198–200 °C; **IR** ν_{max} (film) 1741 (C=O), 1610 (C=C), 1367 (S=O), 1170 (S=O), 1124 (C-O); **^{1}H NMR (400 MHz, CDCl$_3$)** δ **2.29** (3H, s, C*H*$_3$), **5.10–5.19** (2H, m, C(3)*H*O, C*H*^{A}H^B), **5.34** (1H, dd, *J* 13.2, 0.8, CH^A*H*^{B}), **6.25** (1H, dd, *J* 8.0, 1.2, ArC(7)*H*), **6.74** (1H, dd, *J* 1.2, C(3``)*H*), **6.85** (1H, dd, *J* 7.6, 1.6, ArC(5)*H*), **6.87–6.93** (1H, m, ArC(6)*H*), **7.03–7.10** (2H, m, ArC(3```,5```)*H*), **7.18–7.24** (4H, m, ArC(4`)*H*, ArC(5``)H), **7.24–7.30** (8H, m, ArC(3`,5`)*H*, ArC(4)*H*, ArC(6``)H), **7.44–7.50** (7H, m, ArC(2`,6`)*H*, ArC(4``)*H*), **7.70** (2H, d, *J* 8.4, C(2```,6```)*H*), **8.06** (1H, dd, *J* 8.4, 1.2, C(7``)*H*); **$^{13}$C{$^1$H} NMR (101 MHz, CDCl$_3$)** $\delta_C$: **21.7** (*C*H$_3$), **65.1** (*C*H$_2$), **74.5** (N*C*Ph$_3$), **76.0** (*C*(3)HO), **112.3** (*C*(3``)H), **114.7** (Ar*C*(7``)H), **116.0** (Ar*C*(7)H), **121.2** (Ar*C*(4``)H), **122.4** (Ar*C*(5)H), **123.6** (Ar*C*(5``)H), **124.9** (Ar*C*(6``)H), **125.1** (Ar*C*(4)H), **125.6** (Ar*C*(3a)), **126.9** (Ar*C*(2```,6```)H), **127.1** (Ar*C*(4`)H), **127.9** (Ar*C*(3`,5`)H), **128.4** (Ar*C*(6)H), **129.2** (Ar*C*(3a``)), **129.4** (Ar*C*(2`,6`)H), **129.8** (Ar*C*(3```,5```)H), **135.8** (Ar*C*(1```)), **137.2** (Ar*C*(7a``)), **137.3** (Ar*C*(2``)), **142.0** (Ar*C*(1`)), **144.1** (Ar*C*(7a)), **144.8** (Ar*C*(4```)), **176.6** (*C*=O); **HRMS (ESI$^+$)** $C_{43}H_{34}N_2O_4SNa$ [M+Na]$^+$ found 697.2128, requires 697.2131 (–0.45 ppm).

4-Chloro-3-[(1-tosyl-1*H*-indol-2-yl)methoxy]-1-tritylindolin-2-one (S20)

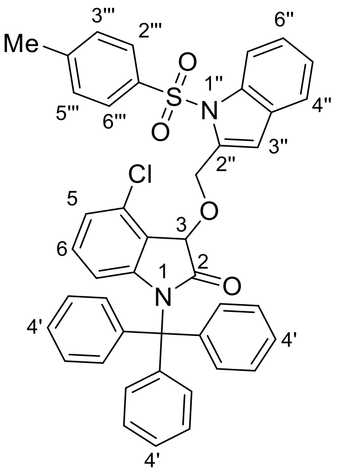

Following **General procedure C**, (1-tosyl-1*H*-indol-2-yl)methanol (0.602 g, 2.0 mmol), 4-chloro-3-diazo-1-tritylindolin-2-one (0.957 g, 2.2 mmol) and $Rh_2(OAc)_4$ (4.41 mg, 0.01 mmol) in CH_2Cl_2 (40 mL, 0.05 M) was added at 0 °C to rt was stirred for 3 h, to give after purification by column chromatography (eluent: Hexane/EtOAc = 5:1 to 3:1), the title compound (0.960 g, 68%) as a pale-yellow amorphous solid. **IR** ν_{max} (film) 1728 (C=O), 1608 (C=C), 1448, 1369, 1336 (S=O), 1174 (S=O), 1143 (C-O); **^{1}H NMR (500 MHz, $CDCl_3$)** δ **2.29** (3H, s, C*H*$_3$), **5.06** (1H, m, C(3)*H*O), **5.17** (1H, d, *J* 13.5, C*H*^{A}H^B), **5.36** (1H, d, *J* 13.4, C*H*^{A}H^B), **6.17** (1H, dd, *J* 6.0, 3.0, ArC(7)*H*), **6.82** (1H, s, C(3``)*H*), **6.85–6.91** (2H, m, ArC(5,6)*H*), **7.06** (2H, d, *J* 8.0, ArC(3```,5```)*H*), **7.18–7.32** (11H, m, ArC(3`,4`,5`,5``,6``)*H*), **7.43–7.50** (7H, m, ArC(3`,5`,4``)*H*), **7.68** (2H, d, *J* 8.0, ArC(2```,6```)*H*), **8.10** (1H, d, *J* 8.4, ArC(7``)*H*); **$^{13}$C{$^1$H} NMR (126 MHz, $CDCl_3$)** $\delta_C$: **21.7** (*C*H$_3$), **64.9** (*C*H$_2$), **74.9** (N*C*Ph$_3$), **75.0** (*C*(3)HO), **111.5** (*C*(3``)H), **114.5** (Ar*C*(7)H), **114.9** (Ar*C*(7``)H), **121.1** (Ar*C*(4``)H), **123.1** (Ar*C*(4)Cl), **123.2** (Ar*C*(5)H), **123.6** (Ar*C*(5``)H), **124.6** (Ar*C*(6``)H), **126.9** (Ar*C*(2```,6```)H), **127.2** (Ar*C*(4`)H), **127.9** (Ar*C*(3`,5`)H), **129.4** (Ar*C*(2`,6`)*H*), **129.6** (Ar*C*(3a``)), **129.8** (Ar*C*(3```,5```)H), **129.9** (Ar*C*(6)H), **132.3** (Ar*C*(3a)), **135.9** (Ar*C*(1```)), **137.1** (Ar*C*(7a``)), **137.2** (Ar*C*(2``)), **141.8** (Ar*C*(1`)), **144.7** (Ar*C*(4```)), **146.2** (Ar*C*(7a)), **174.4** (*C*=O); **HRMS (ESI$^+$)** $C_{43}H_{32}{}^{35}ClN_2O_4SNa$ [M+Na]$^+$ found 731.1718, requires 731.1742 (–3.25 ppm).

5-Chloro-3-((1-tosyl-1*H*-indol-2-yl)methoxy)-1-tritylindolin-2-one (S21)

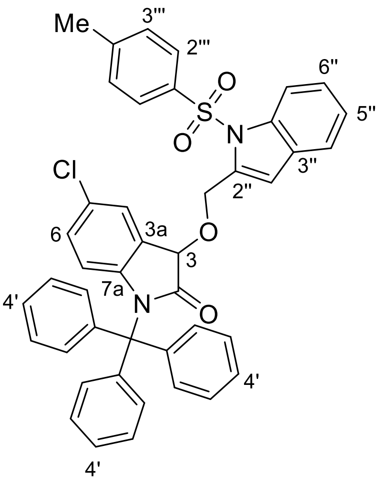

Following **General procedure C**, (1-tosyl-1*H*-indol-2-yl)methanol (0.602 g, 2.0 mmol), 5-chloro-3-diazo-1-tritylindolin-2-one (0.957 g, 2.2 mmol) and $Rh_2(OAc)_4$ (4.41 mg, 0.01 mmol) in CH_2Cl_2 (40 mL, 0.05 M) was added at 0 °C to rt was stirred for 2 h, to give after purification by column chromatography (eluent: Hexane/EtOAc = 5:1 to 3:1), the title compound (0.807 g, 57%) as a pale-yellow solid. **mp** 103–105 °C; **IR** ν_{max} (film) 1732 (C=O), 1469, 1448, 1367, 1172 (S=O), 1120 (C-O), 1089; **^{1}H NMR (500 MHz, $CDCl_3$)** δ **2.30** (3H, s, C*H*$_3$), **5.12** (1H, m, C(3)*H*O), **5.18** (1H, d, *J* 12.7, C*H*^{A}H^B), **5.40** (1H, d, *J* 12.7, C*H*^{A}H^B), **6.17** (1H, d, *J* 8.8, ArC(7)*H*), **6.73** (1H, s, C(3``)*H*), **6.85** (1H, dd, *J* 8.7, 1.9, ArC(6)*H*), **7.08** (1H, dd, *J* 2.4, 1.1, ArC(4)*H*), **7.09** (2H, d, *J* 8.2, ArC(3```,5```)*H*), **7.20–7.25** (4H, m, ArC(4`,5``)*H*), **7.26–7.32** (7H, m, ArC(3`,5`)*H*, ArC(6``)H), **7.43–7.50** (7H, m, ArC(2`,6`)*H*, ArC(4``)*H*), **7.70** (2H, d, *J* 8.4, ArC(2```,6```)*H*), **8.01–8.11** (1H, m, ArC(7``)*H*). **^{13}C{^{1}H} NMR (126 MHz, $CDCl_3$)** δ_C: **21.7** (*C*H$_3$), **65.4** (*C*H$_2$), **74.6** (N*C*Ph$_3$), **75.7**

(*C*(3)HO), **112.5** (*C*(3``)H), **114.7** (Ar*C*(7``)H), **116.9** (Ar*C*(7)H), **121.3** (Ar*C*(4``)H), **123.7** (Ar*C*(5``)H), **125.1** (Ar*C*(6``)H), **125.3** (Ar*C*(4)H), **126.8** (Ar*C*(2```,6```)H), **127.2** (Ar*C*(4`)H), **127.4** (Ar*C*(3a)), **128.0** (Ar*C*(3`,5`)H), **128.0** (Ar*C*(5)Cl), **128.3** (Ar*C*(6)H), **129.0** (Ar*C*(3a``)), **129.4** (Ar*C*(2`,6`)*H*), **129.9** (Ar*C*(3```,5```)H), **136.0** (Ar*C*(1```)), **136.9** (Ar*C*(7a``)), **137.2** (Ar*C*(2``)), **141.7** (Ar*C*(1`)), **142.5** (Ar*C*(7a)), **144.9** (Ar*C*(4```)), **176.3** (*C*=O); **HRMS (ESI$^+$)** $C_{43}H_{32}{}^{35}ClN_2O_4SNa$ $[M+Na]^+$ found 731.1728, requires 731.1742 (–1.88 ppm).

5-Nitro-3-[(1-tosyl-1*H*-indol-2-yl)methoxy]-1-tritylindolin-2-one (S22)

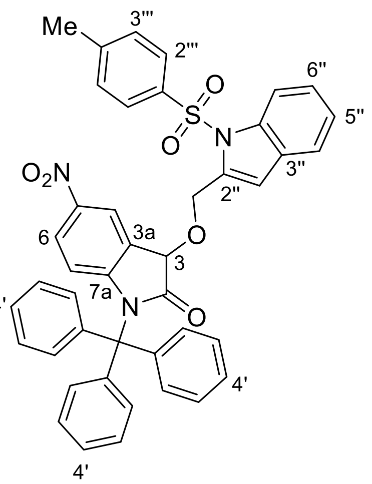

Following **General procedure C**, (1-tosyl-1*H*-indol-2-yl)methanol (0.602 g, 2.0 mmol), 3-diazo-5-nitro-1-tritylindolin-2-one (0.981 g, 2.2 mmol) and $Rh_2(OAc)_4$ (4.41 mg, 0.01 mmol) in CH_2Cl_2 (40 mL, 0.05 M) was added at 0 °C to rt was stirred for 2 h, to give after purification by column chromatography (eluent: Hexane/EtOAc = 5:1 to 3:1), the title compound (0.998 g, 70%) as a pale-yellow amorphous solid. **IR** ν_{max} (film) 1742 (C=O), 1610 (C=C), 1448, 1336 (S=O), 1172 (S=O), 1089, 1072; **^{1}H NMR (500 MHz, $CDCl_3$)** δ **2.27** (3H, s, C*H*$_3$), **5.20** (1H, d, *J* 0.9, C(3)*H*O), **5.23** (1H, d, *J* 12.7, C*H*^{A}H^B), **5.47** (1H, d, *J* 12.7, C*H*^{A}H^B), **6.35** (1H, d, *J* 9.1, ArC(7)*H*), **6.71** (1H, s, C(3``)*H*), **7.07** (2H, d, *J* 8.2, ArC(3```,5```)*H*), **7.21** (1H, td, *J* 7.5, 1.0, ArC(5``)*H*), **7.24–7.28** (3H, m, ArC(4`)*H*), **7.28–7.33** (7H, m, ArC(3`,5`)*H*, ArC(6``)H), **7.42–7.49** (7H, m, ArC(2`,6`)*H*, ArC(4``)*H*), **7.68–7.74** (2H, d, *J* 8.4, ArC(2```,6```)*H*), **7.79** (1H, dd, *J* 9.0, 2.3, ArC(6)*H*), **7.88–7.95** (1H, m, ArC(4)*H*), **8.04** (1H, dd, *J* 8.4, 1.0, ArC(7``)*H*); **$^{13}$C{$^1$H} NMR (126 MHz, $CDCl_3$)** $\delta_C$: **21.7** (*C*H$_3$), **65.9** (*C*H$_2$), **75.2** (*C*(3)HO), **112.6** (*C*(3``)H), **114.7** (Ar*C*(7``)H), **115.5** (Ar*C*(7)H), **120.6** (Ar*C*(4)H), **121.3** (Ar*C*(4``)H), **123.7** (Ar*C*(5``)H), **124.7** (Ar*C*(6)H), **125.3** (Ar*C*(6``)H), **126.6** (Ar*C*(3a)), **126.8** (Ar*C*(2```,6```)H), **127.5** (Ar*C*(4`)H), **128.2** (Ar*C*(3`,5`)H), **128.8** (Ar*C*(3a``)), **129.3** (Ar*C*(2`,6`)*H*), **129.9** (Ar*C*(3```,5```)H), **135.9** (Ar*C*(1```)), **136.5** (Ar*C*(7a``)), **137.2** (Ar*C*(2``)), **141.2** (Ar*C*(1`)), **142.9** (Ar*C*(5)NO$_2$), **149.7** (Ar*C*(7a)), **145.1** (Ar*C*(4```)), **176.6** (*C*=O); **HRMS (ESI$^+$)** $C_{43}H_{33}N_3O_6SNa$ $[M+Na]^+$ found 742.1962, requires 742.1982 (–2.73 ppm).

5-Methyl-3-[(1-tosyl-1*H*-indol-2-yl)methoxy]-1-tritylindolin-2-one (S23)

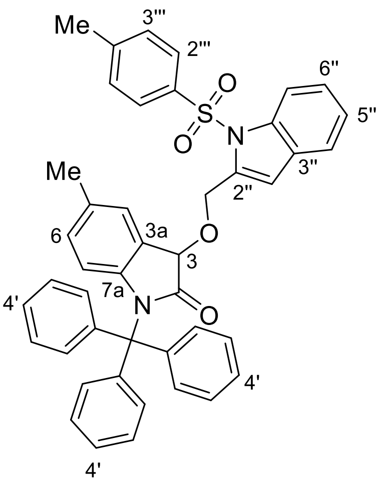

Following **General procedure C**, (1-tosyl-1*H*-indol-2-yl)methanol (0.602 g, 2.0 mmol), 3-diazo-5-methyl-1-tritylindolin-2-one (0.913 g, 2.2 mmol) and $Rh_2(OAc)_4$ (4.41 mg, 0.01 mmol) in CH_2Cl_2 (40 mL, 0.05 M) was added at 0 °C to rt was stirred for 3 h, to give after purification by column chromatography (eluent: Hexane/EtOAc = 5:1 to 3:1), the title compound (0.729 g, 53%) as a pale-yellow solid. **mp** 185–187 °C; **IR** ν_{max} (film) 1732 (C=O), 1487, 1367 (S=O), 1172 (S=O), 1128 (C-O), 1087; **^{1}H NMR (500 MHz, $CDCl_3$)** δ **2.10** (3H, s, ArC(5)C*H*$_3$), **2.30** (3H, s, ArC(4```)C*H*$_3$), **5.13** (1H, m, C(3)*H*O), **5.16** (1H, d, *J* 12.9, CH^AH^B), **5.36** (1H, d, *J* 13.0, CH^AH^B), **6.13** (1H, d, *J* 8.3, ArC(7)*H*), **6.69** (1H, d, *J* 8.2, ArC(6)*H*), **6.72** (1H, s, C(3``)*H*), **7.01** (1H, s, ArC(4)*H*), **7.07** (2H, d, *J* 8.1, ArC(3```,5```)*H*), **7.18–7.24** (4H, m, ArC(4`,5``)*H*), **7.25–7.31** (7H, m, ArC(3`,5`)*H*, ArC(6``)H), **7.46** (1H, d, *J* 7.8, ArC(4``)*H*), **7.48–7.56** (6H, m, ArC(2`,6`)*H*), **7.73** (2H, d, *J* 8.5, ArC(2```,6```)*H*), **8.07** (1H, d, *J* 8.4, ArC(7``)*H*); **^{13}C{^{1}H} NMR (126 MHz, $CDCl_3$)** δ_C: **20.7** (ArC(5)*C*H$_3$), **21.7** (ArC(4```)*C*H$_3$), **65.1** (*C*H$_2$), **74.4** (N*C*Ph$_3$), **76.2** (*C*(3)HO), **112.2** (*C*(3``)H), **114.6** (Ar*C*(7``)H), **115.7** (Ar*C*(7)H), **121.2** (Ar*C*(4``)H), **123.6** (Ar*C*(5``)H), **124.9** (Ar*C*(6``)H), **125.7** (Ar*C*(3a)), **125.8** (Ar*C*(4)H), **126.9** (Ar*C*(2```,6```)H), **127.0** (Ar*C*(4`)H), **127.8** (Ar*C*(3`,5`)H), **128.8** (Ar*C*(6)H), **129.2** (Ar*C*(3a``)), **129.4** (Ar*C*(2`,6`)*H*), **129.8** (Ar*C*(3```,5```)H), **131.9** (Ar*C*(5)Me), **135.9** (Ar*C*(1```)), **137.1** (Ar*C*(7a``)), **137.4** (Ar*C*(2``)), **141.5** (Ar*C*(1`)), **142.1** (Ar*C*(7a)), **144.7** (Ar*C*(4```)), **176.6** (*C*=O); **HRMS (ESI$^+$)** $C_{44}H_{36}N_2O_4SNa$ [M+Na]$^+$ found 711.2284, requires 711.2288 (–0.54 ppm).

5-Methoxy-3-[(1-tosyl-1*H*-indol-2-yl)methoxy]-1-tritylindolin-2-one (S24)

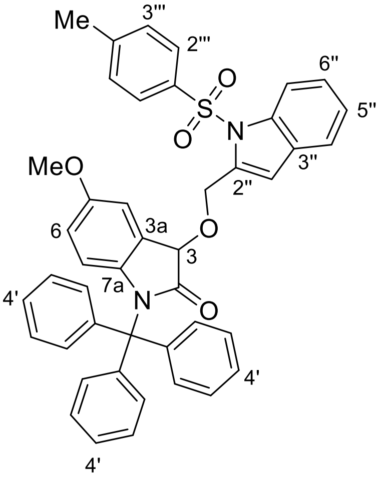

Following **General procedure C**, (1-tosyl-1*H*-indol-2-yl)methanol (0.602 g, 2.0 mmol), 3-diazo-5-methyl-1-tritylindolin-2-one (0.948 g, 2.2 mmol) and $Rh_2(OAc)_4$ (4.41 mg, 0.01 mmol) in CH_2Cl_2 (40 mL, 0.05 M) was added at 0 °C to rt was stirred for 4 h, to give after purification by column chromatography (eluent: Hexane/EtOAc = 5:1 to 3:1), the title compound (1.056 g, 75%) as a pale-red amorphous solid. **IR** ν_{max} (film) 1722 (C=O), 1485, 1448, 1369 (S=O), 1263 (C-O), 1174 (S=O), 1089; **^{1}H NMR (500 MHz, $CDCl_3$)** δ **2.27** (3H, s, ArC(4```)C*H*$_3$), **3.52** (3H, s, ArC(5)OC*H*$_3$), **5.09** (1H, d, *J* 12.8, CH^AH^B), **5.12** (1H, m, C(3)*H*O), **5.33** (1H, d, *J* 12.8, CH^AH^B), **6.15** (1H, d, *J* 8.9, ArC(7)*H*), **6.42** (1H, dd, *J* 8.9, 2.8, ArC(6)*H*), **6.71** (1H, s, C(3``)*H*), **6.75–6.83** (1H, m, ArC(4)*H*), **7.04** (2H, d, *J* 8.0, ArC(3```,5```)*H*), **7.18–7.25** (4H, m, ArC(4`,5``)*H*), **7.25–7.31** (7H, m, ArC(3`,5`)*H*, ArC(6``)H), **7.42–7.55** (6H, m, ArC(2`,6`,4``)*H*), **7.70** (2H, d, *J* 8.0,

ArC(2```,6```)*H*), **8.06** (1H, d, *J* 8.4, ArC(7``)*H*); **^{13}C{^{1}H} NMR (126 MHz, CDCl$_3$)** δ_C: **21.6** (ArC(4```)*C*H$_3$), **55.4** (ArC(4```)*C*H$_3$), **65.2** (*C*H$_2$), **74.5** (N*C*Ph$_3$), **76.4** (*C*(3)HO), **110.8** (Ar*C*(4)H), **112.3** (*C*(3``)H), **114.7** (Ar*C*(6)H), **116.7** (Ar*C*(7)H), **121.2** (Ar*C*(4``)H), **123.6** (Ar*C*(5``)H), **124.9** (Ar*C*(6``)H), **126.8** (Ar*C*(3a)), **126.9** (Ar*C*(2```,6```)H), **127.0** (Ar*C*(4`)H), **127.8** (Ar*C*(3`,5`)H), **129.2** (Ar*C*(3a``)), **129.4** (Ar*C*(2`,6`)*H*), **129.8** (Ar*C*(3```,5```)H), **135.8** (Ar*C*(1```)), **137.1** (Ar*C*(7a``)), **137.2** (Ar*C*(7a)), **137.3** (Ar*C*(2``)), **142.1** (Ar*C*(1`)), **144.8** (Ar*C*(4```)), **155.4** (Ar*C*(5)OMe). **176.5** (*C*=O); **HRMS (ESI$^+$)** $C_{44}H_{36}N_2O_5SNa$ [M+Na]$^+$ found 727.2226, requires 727.2238 (–1.65 ppm).

6-Chloro-3-[(1-tosyl-1*H*-indol-2-yl)methoxy]-1-tritylindolin-2-one (S25)

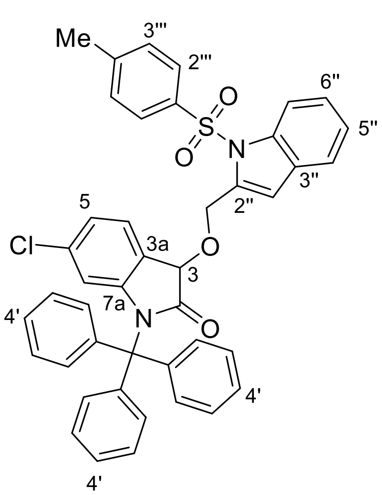

Following **General procedure C**, (1-tosyl-1*H*-indol-2-yl)methanol (0.602 g, 2.0 mmol), 6-chloro-3-diazo-1-tritylindolin-2-one (0.957 g, 2.2 mmol) and $Rh_2(OAc)_4$ (4.41 mg, 0.01 mmol) in CH_2Cl_2 (40 mL, 0.05 M) was added at 0 °C to rt was stirred for 2 h, to give after purification by column chromatography (eluent: Hexane/EtOAc = 5:1 to 3:1), the title compound (0.878 g, 62%) as a pale-yellow solid. **mp** 110–112 °C; **IR** ν_{max} (film) 1732 (C=O), 1608 (C=C), 1448, 1369 (S=O), 1174 (S=O), 1151, 1089; **^{1}H NMR (400 MHz, CDCl$_3$)** δ **2.30** (3H, s, C*H*$_3$), **5.07–5.11** (1H, m, C(3)*H*O), **5.14** (1H, d, *J* 12.5, C*H*^{A}H^B), **5.33** (1H, d, *J* 13.0, C*H*^{A}H^B), **6.20** (1H, d, *J* 1.8, ArC(7)*H*), **6.69–6.74** (1H, m, C(3``)*H*), **6.81** (1H, dd, *J* 7.9, 1.8, ArC(5)*H*), **7.04–7.09** (2H, m, ArC(3```,5```)*H*), **7.14** (1H, dd, *J* 8.0, 1.1, ArC(4)*H*), **7.18–7.26** (3H, m, ArC*H*), **7.26–7.34** (8H, m, ArC(3`,5`)*H*, ArC(6``)H, ArC*H*), **7.44–7.51** (7H, m, ArC(2`,6`)*H*, ArC(4``)*H*), **7.62–7.74** (2H, m, ArC(2```,6```)*H*), **8.07** (1H, dd, *J* 8.4, 0.9, ArC(7``)*H*); **$^{13}$C{$^{1}$H} NMR (101 MHz, CDCl$_3$)** $\delta_C$: **21.6** (*C*H$_3$), **65.3** (*C*H$_2$), **74.7** (N*C*Ph$_3$), **75.5** (*C*(3)HO), **112.5** (*C*(3``)H), **114.6** (Ar*C*(7``)H), **116.3** (Ar*C*(7)H), **121.2** (Ar*C*(4``)H), **122.4** (Ar*C*(5)H), **123.7** (Ar*C*(5``)H), **124.0** (Ar*C*(3a)), **125.0** (Ar*C*(6``)H), **126.0** (Ar*C*(4)H), **126.8** (Ar*C*(2```,6```)H), **127.3** (Ar*C*(4`)H), **128.0** (Ar*C*(3`,5`)H), **129.1** (Ar*C*(3a``)), **129.3** (Ar*C*(2`,6`)*H*), **129.8** (Ar*C*(3```,5```)H), **134.1** (Ar*C*(6)Cl), **135.8** (Ar*C*(1```)), **137.0** (Ar*C*(7a``)), **137.2** (Ar*C*(2``)), **141.6** (Ar*C*(1`)), **144.9** (Ar*C*(4```)), **145.2** (Ar*C*(7a)), **176.5** (*C*=O); **HRMS (ESI$^+$)** $C_{43}H_{32}{}^{35}ClN_2O_4SNa$ [M+Na]$^+$ found 731.1737, requires 731.1742 (–0.65 ppm).

6-Bromo-3-[(1-tosyl-1*H*-indol-2-yl)methoxy]-1-tritylindolin-2-one (S26)

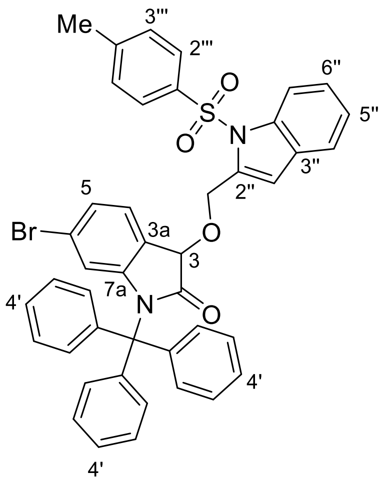

Following **General procedure C**, (1-tosyl-1*H*-indol-2-yl)methanol (0.602 g, 2.0 mmol), 6-bromo-3-diazo-1-tritylindolin-2-one (1.054 g, 2.2 mmol) and $Rh_2(OAc)_4$ (4.41 mg, 0.01 mmol) in CH_2Cl_2 (40 mL, 0.05 M) was added at 0 °C to rt was stirred for 2 h, to give after purification by column chromatography (eluent: Hexane/EtOAc = 5:1 to 3:1), the title compound (1.10 g, 73%) as a pale-yellow amorphous solid. **IR** ν_{max} (film) 1732 (C=O), 1606 (C=C), 1448, 1371 (S=O), 1172 (S=O), 1118, 1089; **^{1}H NMR (500 MHz, $CDCl_3$)** δ **2.30** (3H, s, C*H*3), **5.06** (1H, s, C(3)*H*O), **5.12** (1H, d, *J* 12.8, C*H^A*H^B), **5.31** (1H, d, *J* 12.8, C*H^A*H^B), **6.32** (1H, d, *J* 1.6, ArC(7)*H*), **6.71** (1H, s, C(3``)*H*), **6.96** (1H, dd, *J* 7.9, 1.6, ArC(5)*H*), **7.04–7.09** (3H, m, ArC(3```,4,5```)*H*), **7.18–7.26** (4H, m, ArC(4`,5``)*H*), **7.27–7.32** (7H, m, ArC(3`,5`,6``)*H*), **7.43–7.47** (7H, m, ArC(2`,6`,4``)*H*), **7.67** (2H, d, *J* 8.4, ArC(2```,6```)*H*), **8.06** (1H, d, *J* 8.4, ArC(7``)*H*); **$^{13}$C{$^1$H} NMR (126 MHz, $CDCl_3$)** $\delta_C$: **21.7** (*C*H3), **65.3** (*C*H2), **74.8** (N*C*Ph3), **75.5** (*C*(3)HO), **112.5** (*C*(3``)H), **114.7** (Ar*C*(7``)H), **119.0** (Ar*C*(7)H), **121.2** (Ar*C*(4``)H), **122.1** (Ar*C*(6)Br), **123.7** (Ar*C*(5``)H), **124.6** (Ar*C*(3a)), **125.0** (Ar*C*(6``)H), **125.3** (Ar*C*(5)H), **126.3** (Ar*C*(4)H), **126.8** (Ar*C*(2```,6```)H), **127.3** (Ar*C*(4`)H), **128.0** (Ar*C*(3`,5`)H), **129.1** (Ar*C*(3a``)), **129.3** (Ar*C*(2`,6`)*H*), **129.8** (Ar*C*(3```,5```)H), **135.8** (Ar*C*(1```)), **137.0** (Ar*C*(2``)), **137.2** (Ar*C*(7a``)), **141.6** (Ar*C*(1`)), **144.9** (Ar*C*(4```)), **145.3** (Ar*C*(7a)), **176.4** (*C*=O); **HRMS (ESI$^+$)** $C_{43}H_{33}{}^{79}BrN_2O_4SNa$ $[M+Na]^+$ found 775.1229, requires 775.1237 (–0.98 ppm).

1-Benzyl-7-chloro-3-[(1-tosyl-1*H*-indol-2-yl)methoxy]indolin-2-one (S27)

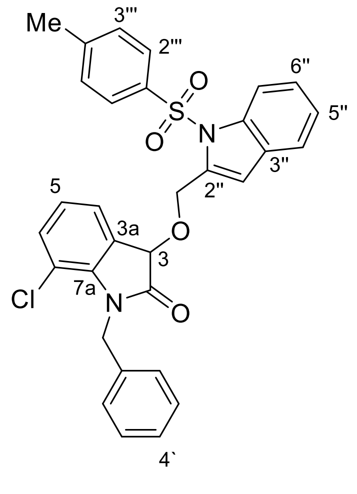

Following **General procedure C**, (1-tosyl-1*H*-indol-2-yl)methanol (0.602 g, 2.0 mmol), 1-benzyl-7-chloro-3-diazoindolin-2-one (0.623 g, 2.2 mmol) and $Rh_2(OAc)_4$ (4.41 mg, 0.01 mmol) in CH_2Cl_2 (40 mL, 0.05 M) was added at 0 °C to rt was stirred for 2 h, to give after purification by column chromatography (eluent: Hexane/EtOAc = 5:1 to 3:1), the title compound (0.43 g, 39%) as a pale-yellow amorphous solid. **IR** ν_{max} (film) 1724 (C=O), 1610 (C=C), 1450, 1367 (S=O), 1172 (S=O), 1130, 1089; **^{1}H NMR (500 MHz, $CDCl_3$)** δ **2.31** (3H, s, C*H*3), **5.13** (1H, s, C(3)*H*O), **5.28–5.34** (2H, m, OC*H^A*H^B and NC*H^A*H^BPh), **5.35–5.41** (1H, m, NCH^A*H^B*Ph), **5.46–5.52** (1H, m, OCH^A*H^B*), **6.83** (1H, s, C(3``)*H*), **6.92** (1H, dd, *J* 8.2, 7.3, ArC(5)*H*), **7.11–7.16** (2H, m, ArC(3```,5```)*H*), **7.18** (1H, dt, *J* 8.2, 1.1, ArC(6)*H*), **7.20–7.26** (2H, m, ArC(4`,5``)*H*), **7.27–7.34** (6H, m, ArC(4,2`,3`,5`,6`,6``)*H*), **7.49** (1H, d, *J* 7.7, ArC(4``)*H*), **7.73–7.80** (2H, m, ArC(2```,6```)*H*), **8.08** (1H, dd, *J* 8.4, 0.9, ArC(7``)*H*); **$^{13}$C{$^1$H} NMR (126 MHz, $CDCl_3$)** $\delta_C$: **21.7** (*C*H3), **44.8** (N*C*H2Ph), **65.3** (O*C*H2), **75.0** (*C*(3)HO), **112.7** (*C*(3``)H), **114.7** (Ar*C*(7``)H), **115.7** (Ar*C*(7)Cl), **121.3** (Ar*C*(4``)H), **123.7** (Ar*C*(5``)H), **124.0**

(Ar*C*(5``)H), **124.8** (Ar*C*(4)H), **125.1** (Ar*C*(4`)H), **126.6** (Ar*C*(2`,6`)H), **126.9** (Ar*C*(2```,6```)H), **127.4** (Ar*C*(6``)H), **128.0** (Ar*C*(3a)), **128.8** (Ar*C*(3`,5`)H), **129.1** (Ar*C*(3a``)), **129.9** (Ar*C*(3```,5```)H), **132.6** (Ar*C*(6)H), **135.8** (Ar*C*(1```)), **136.6** (Ar*C*(2``)), **137.2** (Ar*C*(1`,7a``)), **139.5** (Ar*C*(7a)), **145.0** (Ar*C*(4```)CH_3), **175.4** (*C*=O); **HRMS (ESI$^+$)** $C_{31}H_{25}{}^{35}ClN_2O_4SNa$ $[M+Na]^+$ found 579.1121, requires 579.1116 (0.90 ppm).

3-[(4-Methyl-1-tosyl-1*H*-indol-2-yl)methoxy]-1-tritylindolin-2-one (S28)

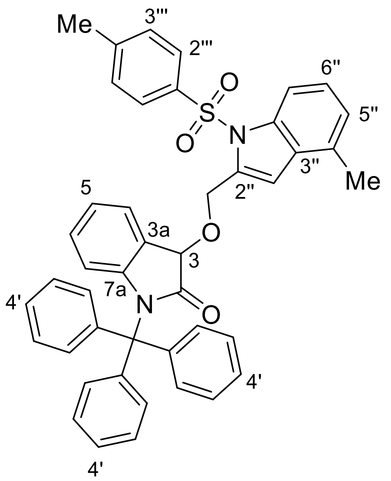

Following **General procedure C**, (4-methyl-1-tosyl-1*H*-indol-2-yl)methanol (0.631 g, 2.0 mmol), 3-diazo-1-tritylindolin-2-one (0.882 g, 2.2 mmol) and $Rh_2(OAc)_4$ (4.41 mg, 0.01 mmol) in CH_2Cl_2 (40 mL, 0.05 M) was added at 0 °C to rt was stirred for 2 h, to give after purification by column chromatography (eluent: Hexane/EtOAc = 5:1 to 3:1), the title compound (1.06 g, 77%) as a amorphous pale-yellow solid. **IR** ν_{max} (film) 1724 (C=O), 1597 (C=C), 1448, 1367 (S=O), 1178 (S=O), 1163, 1095; **^{1}H NMR (500 MHz, $CDCl_3$)** δ **2.29** (3H, s, ArC(4```)C*H*$_3$), **2.44** (3H, s, ArC(4``)C*H*$_3$), **5.13–5.20** (2H, m, C(3)*H*O, C*H*$^A$H$^B$), **5.34** (1H, d, *J* 12.8, CH$^A$*H*$^B$), **6.27** (1H, d, *J* 8.1, ArC(7)*H*), **6.79** (1H, s, C(3``)*H*), **6.85** (1H, td, *J* 7.5, 1.1, ArC(5)*H*), **6.91** (1H, td, *J* 7.5, 1.1, ArC(6)*H*), **7.00** (1H, d, *J* 7.3, ArC(5``)*H*), **7.07** (1H, d, *J* 7.3, ArC(3```,5```)*H*), **7.18** (1H, t, *J* 8.0, ArC(6``)*H*), **7.18–7.25** (3H, m, ArC(4`)*H*), **7.24–7.31** (7H, m, ArC(4,3`,5`)*H*), **7.46–7.53** (6H, m, ArC(2`,6`)*H*), **7.72** (2H, d, *J* 8.4, ArC(2```,6```)*H*), **7.90** (1H, d, *J* 8.4, ArC(7``)*H*); **^{13}C{^{1}H} NMR (126 MHz, $CDCl_3$)** δ$_C$: **18.5** (ArC(5)*C*H$_3$), **21.6** (ArC(4```)*C*H$_3$), **65.1** (*C*H$_2$), **74.6** (N*C*Ph$_3$), **76.0** (*C*(3)HO), **110.7** (*C*(3``)H), **112.2** (Ar*C*(7``)H), **116.0** (Ar*C*(7)H), **122.4** (Ar*C*(5)H), **124.0** (Ar*C*(5``)H), **125.0** (Ar*C*(6``)H), **125.2** (Ar*C*(4)H), **125.7** (Ar*C*(3a)), **127.0** (Ar*C*(2```,6```)H), **127.1** (Ar*C*(4`)H), **127.9** (Ar*C*(3`,5`)H), **128.4** (Ar*C*(6)H), **128.9** (Ar*C*(4``)CH_3), **129.5** (Ar*C*(2`,6`)*H*), **129.8** (Ar*C*(3```,5```)H), **130.7** (Ar*C*(3a``)), **136.0** (Ar*C*(1```)), **136.7** (Ar*C*(2``)), **137.0** (Ar*C*(7a``)), **141.5** (Ar*C*(1`)), **144.1** (Ar*C*(7a)), **144.7** (Ar*C*(4```)), **176.6** (*C*=O); **HRMS (ESI$^+$)** $C_{44}H_{36}N_2O_4SNa$ $[M+Na]^+$ found 711.2295, requires 711.2288 (0.98 ppm).

3-[(5-Chloro-1-tosyl-1*H*-indol-2-yl)methoxy]-1-tritylindolin-2-one (S29)

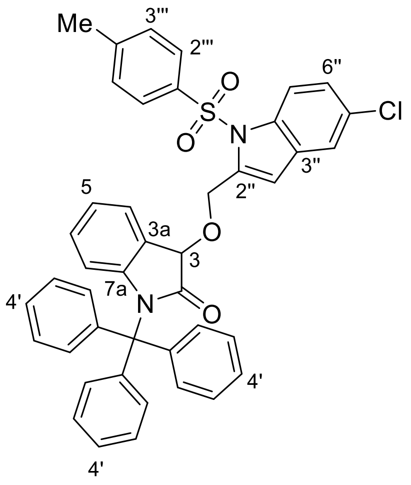

Following **General procedure C**, (5-chloro-1-tosyl-1*H*-indol-2-yl)methanol (0.670 g, 2.0 mmol), 3-diazo-1-tritylindolin-2-one (0.882 g, 2.2 mmol) and $Rh_2(OAc)_4$ (4.41 mg, 0.01 mmol) in CH_2Cl_2 (40 mL, 0.05 M) was added at 0 °C to rt was stirred for 2 h, to give after purification by column chromatography (eluent: Hexane/EtOAc = 5:1 to 3:1), the title compound (0.72 g, 51%) as a pale-yellow amorphous solid. **IR** ν_{max} (film) 1736 (C=O), 1595 (C=C), 1446, 1371 (S=O), 1168 (S=O), 1124, 1089; **^{1}H NMR (500 MHz, CDCl$_3$)** δ **2.30** (3H, s, C*H*$_3$), **5.11** (1H, d, *J* 13.1, CH^A*H*^{B}), **5.14** (1H, s, C(3)*H*O), **5.29** (1H, d, *J* 13.1, CH^A*H*^{B}), **6.27** (1H, d, *J* 8.0, ArC(7)*H*), **6.67** (1H, s, C(3``)*H*), **6.86** (1H, t, *J* 7.0, ArC(5)*H*), **6.91** (1H, t, *J* 7.0, ArC(6)*H*), **7.07** (2H, d, *J* 8.1, ArC(3```,5```)*H*), **7.19–7.31** (11H, m, ArC(4,3`,4`,5`,6``)*H*), **7.42** (1H, d, *J* 2.1, ArC(4``)*H*), **7.48** (6H, d, *J* 7.4, ArC(2`,6`)*H*), **7.67** (1H, d, *J* 8.4, ArC(2```,6```)*H*), **7.99** (1H, d, *J* 8.9, C(7``)*H*); **$^{13}$C{$^1$H} NMR (126 MHz, CDCl$_3$)** $\delta_C$: **21.7** (Ar*C*H$_3$), **64.9** (*C*H$_2$), **74.6** (N*C*Ph$_3$), **76.2** (*C*(3)HO), **111.2** (*C*(3``)H), **115.7** (Ar*C*(7``)H), **116.1** (Ar*C*(7)H), **120.7** (Ar*C*(4``)H), **122.4** (Ar*C*(5)H), **125.0** (Ar*C*(6``)H), **125.0** (Ar*C*(4)H), **125.4** (Ar*C*(3a)), **126.9** (Ar*C*(2```,6```)H), **127.1** (Ar*C*(4`)H), **127.9** (Ar*C*(3`,5`)H), **128.5** (Ar*C*(6)H), **129.4** (Ar*C*(2`,6`)H), **129.9** (Ar*C*(3```,5```)H), **130.5** (Ar*C*(3a``)), **135.4** (Ar*C*(2``)), **135.5** (Ar*C*(1```)),**138.8** (Ar*C*(7a``)), **142.0** (Ar*C*(1`)), **144.1** (Ar*C*(7a)), **145.1** (Ar*C*(4```)), **176.5** (*C*=O); **HRMS (ESI$^+$)** $C_{43}H_{32}{}^{35}ClN_2O_4SNa$ [M+Na]$^+$ found 731.1737, requires 731.1737 (0 ppm).

3-[(5-Bromo-1-tosyl-1*H*-indol-2-yl)methoxy]-1-tritylindolin-2-one (S30)

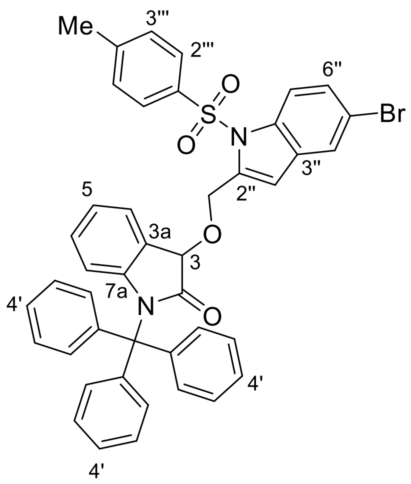

Following **General procedure C**, (5-bromo-1-tosyl-1*H*-indol-2-yl)methanol (0.758 g, 2.0 mmol), 3-diazo-1-tritylindolin-2-one (0.882 g, 2.2 mmol) and $Rh_2(OAc)_4$ (4.41 mg, 0.01 mmol) in CH_2Cl_2 (40 mL, 0.05 M) was added at 0 °C to rt was stirred for 2 h, to give after purification by column chromatography (eluent: Hexane/EtOAc = 5:1 to 3:1), the title compound (1.022 g, 68%) as a pale-yellow solid. **mp** 109–111 °C; **IR** ν_{max} (film) 1736 (C=O), 1595 (C=C), 1446, 1371 (S=O), 1305, 1167 (S=O), 1124 (C-O), 1087; **^{1}H NMR (500 MHz, CDCl$_3$)** δ **2.31** (3H, s, C*H*$_3$), **5.07–5.19** (2H, m, C(3)*H*O, C*H*^{A}H^B), **5.29** (1H, d, *J* 13.2, CH^A*H*^{B}), **6.28** (1H, d, *J* 8.0, ArC(7)*H*), **6.68** (1H, s, C(3``)*H*), **6.87** (1H, t, *J* 7.3, ArC(5)*H*), **6.92** (1H, t, *J* 7.4, ArC(6)*H*), **7.07** (2H, d, *J* 8.1, ArC(3```,5```)*H*), **7.19–7.24** (3H, m, ArC(4`)*H*), **7.24–7.31** (7H, m, ArC(3`,5`)*H*, ArC(4)*H*), **7.37** (1H, dd, *J* 8.9, 2.0, ArC(6``)*H*), **7.49** (6H, d, *J* 7.6, ArC(2`,6`)*H*), **7.59** (1H, d, *J* 2.0,

ArC(4``)*H*), **7.67** (1H, d, *J* 8.4, ArC(2```,6```)*H*), **7.95** (1H, d, *J* 8.9, C(7``)*H*); **$^{13}$C{$^{1}$H} NMR (126 MHz, $CDCl_3$)** $\delta_C$: **21.7** (Ar*C*H$_3$), **64.8** (*C*H$_2$), **74.6** (N*C*Ph$_3$), **76.1** (*C*(3)HO), **110.9** (*C*(3``)H), **116.1** (Ar*C*(7``)H), **116.1** (Ar*C*(7)H), **117.1** (Ar*C*(5``)Br), **122.4** (Ar*C*(5)H), **123.7** (Ar*C*(4``)H), **125.1** (Ar*C*(4)H), **125.5** (Ar*C*(3a)), **126.9** (Ar*C*(2```,6```)H), **127.1** (Ar*C*(4`)H), **127.7** (Ar*C*(6``)H), **127.9** (Ar*C*(3`,5`)H), **128.5** (Ar*C*(6)H), **129.4** (Ar*C*(2`,6`)H), **129.9** (Ar*C*(3```,5```)H), **131.1** (Ar*C*(3a``)), **135.6** (Ar*C*(1```)), **135.9** (Ar*C*(2``)), **138.8** (Ar*C*(7a``)), **142.1** (Ar*C*(1`)), **144.2** (Ar*C*(7a)), **145.1** (Ar*C*(4```)), **176.4** (*C*=O); **HRMS (ESI$^+$)** $C_{43}H_{33}{}^{79}BrN_2O_4SNa$ [M+Na]$^+$ found 775.1223, requires 775.1237 (–1.76 ppm).

3-[(5-Methyl-1-tosyl-1*H*-indol-2-yl)methoxy]-1-tritylindolin-2-one (S31)

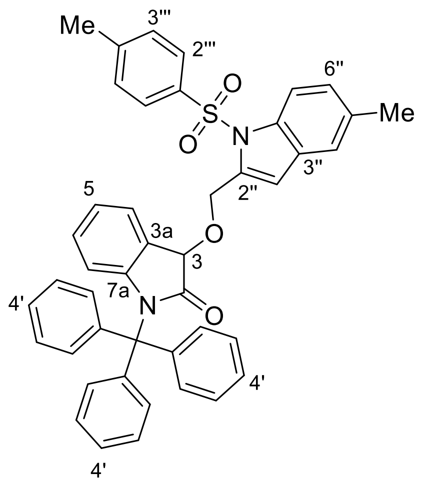

Following **General procedure C**, (5-methyl-1-tosyl-1*H*-indol-2-yl)methanol (0.631 g, 2.0 mmol), 3-diazo-1-tritylindolin-2-one (0.882 g, 2.2 mmol) and $Rh_2(OAc)_4$ (4.41 mg, 0.01 mmol) in CH_2Cl_2 (40 mL, 0.05 M) was added at 0 °C to rt was stirred for 2 h, to give after purification by column chromatography (eluent: Hexane/EtOAc = 5:1 to 3:1), the title compound (0.922 g, 67%) as a pale-yellow solid. **mp** 175–177 °C; **IR** ν_{max} (film) 1741 (C=O), 1595 (C=C), 1465, 1367 (S=O), 1180, 1165 (S=O), 1118, 1085; **^{1}H NMR (500 MHz, $CDCl_3$)** δ **2.29** (3H, s, ArC(4```)C*H*$_3$), **2.39** (3H, s, ArC(5``)C*H*$_3$), **5.09–5.18** (2H, m, C(3)*H*O, C*H*$^A$H$^B$), **5.29–5.37** (1H, m, CH$^A$*H*$^B$), **6.26** (1H, d, *J* 8.0, ArC(7)*H*), **6.68** (1H, s, ArC(3``)*H*), **6.85** (1H, td, *J* 7.5, 1.1, ArC(5)*H*), **6.90** (1H, td, *J* 7.8, 1.7, ArC(6)*H*), **7.06** (2H, d, *J* 8.1, ArC(3```,5```)*H*), **7.10** (1H, dd, *J* 8.6, 1.8, ArC(6``)*H*), **7.19–7.24** (3H, m, ArC(4`)*H*), **7.24–7.31** (8H, m, ArC(4,3`,5`,4``)*H*), **7.45–7.52** (6H, m, ArC(2`,6`)*H*), **7.69** (2H, d, *J* 8.4, ArC(2```,6```)*H*), **7.95** (1H, d, *J* 8.6, ArC(7``)*H*); **$^{13}$C{$^{1}$H} NMR (126 MHz, $CDCl_3$)** $\delta_C$: **21.3** (ArC(5``)*C*H$_3$), **21.6** (ArC(4```)*C*H$_3$), **65.1** (*C*H$_2$), **74.6** (N*C*Ph$_3$), **76.0** (*C*(3)HO), **112.2** (*C*(3``)H), **114.4** (Ar*C*(7``)H), **116.0** (Ar*C*(7)H), **121.1** (Ar*C*(4``)H), **122.4** (Ar*C*(5)H), **125.2** (Ar*C*(4)H), **125.7** (Ar*C*(3a)), **126.3** (Ar*C*(6``)H), **126.9** (Ar*C*(2```,6```)H), **127.1** (Ar*C*(4`)H), **127.8** (Ar*C*(3`,5`)H), **128.4** (Ar*C*(6)H), **129.5** (Ar*C*(2`,6`)*H*), **129.6** (Ar*C*(3a``)), **129.8** (Ar*C*(3```,5```)H), **133.2** (Ar*C*(5``)Me), **135.5** (Ar*C*(7a``)), **136.0** (Ar*C*(1```)), **137.4** (Ar*C*(2``)), **142.1** (Ar*C*(1`)), **144.1** (Ar*C*(7a)), **144.6** (Ar*C*(4```)), **176.6** (*C*=O); **HRMS (ESI$^+$)** $C_{44}H_{36}N_2O_4SNa$ [M+Na]$^+$ found 711.2278, requires 711.2288 (–1.41 ppm).

3-[(6-Methyl-1-tosyl-1*H*-indol-2-yl)methoxy]-1-tritylindolin-2-one (S32)

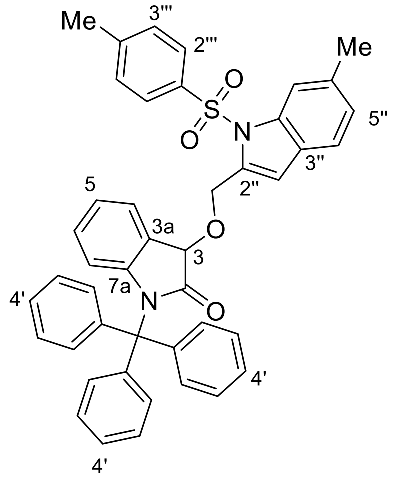

Following **General procedure C**, (6-methyl-1-tosyl-1*H*-indol-2-yl)methanol (0.630 g, 2.0 mmol), 3-diazo-1-tritylindolin-2-one (0.882 g, 2.2 mmol) and $Rh_2(OAc)_4$ (4.41 mg, 0.01 mmol) in CH_2Cl_2 (40 mL, 0.05 M) was added at 0 °C to rt was stirred for 2 h, to give after purification by column chromatography (eluent: Hexane/EtOAc = 5:1 to 3:1), the title compound (1.04 g, 75%) as a pale-yellow amorphous solid. **IR** ν_{max} (film) 1724 (C=O), 1597 (C=C), 1448, 1367 (S=O), 1170 (S=O), 1089; **^{1}H NMR (500 MHz, CDCl$_3$)** δ **2.30** (3H, s, ArC(4```)C*H*$_3$), **2.46** (3H, s, ArC(6``)C*H*$_3$), **5.05–5.17** (2H, m, C(3)*H*O, C*H*$^A$H$^B$), **5.31** (1H, d, *J* 12.9, CH$^A$*H*$^B$), **6.25** (1H, d, *J* 8.0, ArC(7)*H*), **6.70** (1H, s, ArC(3``)*H*), **6.84** (1H, td, *J* 7.9, 1.6, ArC(5)*H*), **6.89** (1H, td, *J* 7.9, 1.6, ArC(6)*H*), **7.01–7.05** (1H, m, ArC*H*), **7.07** (2H, d, *J* 8.2, ArC(3```,5```)*H*), **7.19–7.24** (4H, m, ArC(4,4`)*H*), **7.24–7.30** (6H, m, ArC(3`,5`)*H*), **7.34** (1H, d, *J* 7.9, ArC*H*), **7.45–7.51** (6H, m, ArC(2`,6`)*H*), **7.70** (2H, d, *J* 8.4, ArC(2```,6```)*H*), **7.89** (1H, s, ArC(7``)*H*); **$^{13}$C{$^1$H} NMR (126 MHz, CDCl$_3$)** δ$_C$: **18.5** (ArC(5``)CH$_3$), **21.6** (ArC(4```)*C*H$_3$), **65.1** (*C*H$_2$), **74.6** (N*C*Ph$_3$), **76.0** (*C*(3)HO), **110.7** (*C*(3``)H), **112.2** (Ar*C*(7``)H), **116.0** (Ar*C*(7)H), **122.4** (Ar*C*(5)H), **124.0** (Ar*C*H), **125.0** (Ar*C*(4)H), **125.2** (Ar*C*), **125.7** (Ar*C*(3a)), **127.0** (Ar*C*(2```,6```)H), **127.1** (Ar*C*(4`)H), **127.9** (Ar*C*(3`,5`)H), **128.4** (Ar*C*(6)H), **128.9** (Ar*C*(3a``)), **129.5** (Ar*C*(2`,6`)*H*), **129.8** (Ar*C*(3```,5```)H), **130.7** (Ar*C*(6``)Me), **136.0** (Ar*C*(7a``)), **136.7** (Ar*C*(1```)), **137.0** (Ar*C*(2``)), **142.1** (Ar*C*(1`)), **144.1** (Ar*C*(7a)), **144.7** (Ar*C*(4```)), **176.6** (*C*=O); **HRMS (ESI$^+$)** $C_{44}H_{36}N_2O_4SNa$ $[M+Na]^+$ found 711.2294, requires 711.2288 (0.84 ppm).

3-(Benzofuran-2-ylmethoxy)-1-tritylindolin-2-one (S33)

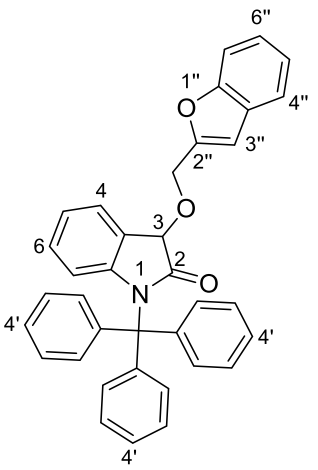

Following **General procedure C**, (3-methyl-1-tosyl-1*H*-indol-2-yl)methanol (0.296 g, 2.0 mmol), 3-diazo-1-tritylindolin-2-one (0.882 g, 2.2 mmol) and $Rh_2(OAc)_4$ (4.41 mg, 0.01 mmol) in CH_2Cl_2 (40 mL, 0.05 M) was added at 0 °C to rt was stirred for 3 h, to give after purification by column chromatography (eluent: Hexane/EtOAc = 5:1 to 3:1), the title compound (0.625 g, 60%) as a pale-orange solid. **mp** 173–175 °C; **IR** ν_{max} (film) 1728 (C=O), 1608 (C=C), 1463, 1448, 1182, 1103 (C-O); **^{1}H NMR (400 MHz, CDCl$_3$)** δ **4.87–4.98** (2H, m, C*H*$_2$), **5.09** (1H, d, *J* 0.8, C(3)*H*), **6.22–6.29** (1H, m, ArC(7)*H*), **6.70** (1H, d, *J* 0.8, C(3``)*H*), **6.87–6.93** (2H, m, ArC(5,6)*H*), **7.20–7.25** (4H, m, ArC(4`)*H*, ArC(5``)H), **7.25–7.32** (8H, m, ArC(3`,5`)*H*, ArC(4)*H*, ArC(6``)H), **7.45–7.52** (7H, m, ArC(2`,6`)*H*, ArC(4``)*H*), **7.52–7.57** (1H, m, ArC(7``)*H*); **$^{13}$C{$^1$H} NMR (101 MHz, CDCl$_3$)** δ$_C$: **63.8** (*C*H$_2$), **74.6** (N*C*Ph$_3$), **74.8** (*C*(3)HO), **106.75** (*C*(3``)H), **111.5** (Ar*C*(4``)H), **116.1** (Ar*C*(7)H), **121.3** (Ar*C*(7``)H), **122.5** (Ar*C*(5)H), **122.9**

(Ar*C*(5``)H), **124.6** (Ar*C*(6``)H), **124.8** (Ar*C*(4)H), **125.7** (Ar*C*(3a)), **127.1** (Ar*C*(4`)H), **127.9** (Ar*C*(3`,5`)H), **128.1** (Ar*C*(3a``)), **128.5** (Ar*C*(6)H), **129.4** (Ar*C*(2`,6`)H), **142.0** (Ar*C*(1`)), **144.0** (Ar*C*(7a)), **153.8** (*C*(2``)), **155.5** (Ar*C*(7a``)), **176.8** (*C*=O); **HRMS (ESI$^+$)** $C_{36}H_{27}NO_3Na$ $[M+Na]^+$ found 544.1873, requires 544.1883 (–1.78 ppm).

3-[(1-Tosyl-1*H*-pyrrol-2-yl)methoxy]-1-tritylindolin-2-one (S34)

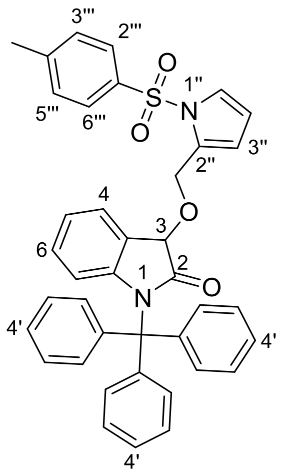

Following **General procedure C**, (1-tosyl-1*H*-pyrrol-2-yl)methanol (0.502 g, 2.0 mmol), 3-diazo-1-tritylindolin-2-one (0.882 g, 2.2 mmol) and $Rh_2(OAc)_4$ (4.41 mg, 0.01 mmol) in CH_2Cl_2 (40 mL, 0.05 M) was added at 0 °C to rt was stirred for 3 h, to give after purification by column chromatography (eluent: Hexane/EtOAc = 5:1 to 3:1), the title compound (0.786 g, 63%) as a pale-yellow solid. **mp** 70–72 °C; **IR** ν_{max} (film) 1728 (C=O), 1608 (C=C), 1595 (C=C), 1463, 1448, 1367 (S=O), 1174 (S=O), 1147 (C-O); **^{1}H NMR (500 MHz, CDCl$_3$)** δ **2.32** (3H, s, C*H*$_3$), **4.82** (1H, d, *J* 12.3, C*H*^{A}H^B), **4.95** (1H, s, C(3)*H*), **5.15** (1H, d, *J* 12.3, CH^A*H*^{B}), **6.17–6.25** (2H, m, ArC(7,4``)*H*), **6.32** (1H, dd, *J* 3.4, 1.8, ArC(3``)*H*), **6.79** (1H, td, *J* 7.5, 1.0, ArC(5)*H*), **6.86** (1H, td, *J* 7.9, 1.6, ArC(6)*H*), **6.98** (1H, dt, *J* 7.2, 1.3, ArC(4)*H*), **7.08–7.12** (2H, m, ArC(3```,5```)*H*), **7.18–7.22** (3H, m, ArC(4`)*H*), **7.29** (1H, dd, *J* 3.3, 1.8, ArC(5``)*H*), **7.24–7.28** (6H, m, ArC(3`,5`)*H*), **7.44–7.49** (6H, m, ArC(2`,6`)*H*), **7.66–7.72** (2H, m, ArC(2```,6```)*H*); **^{13}C{^{1}H} NMR (126 MHz, CDCl$_3$)** δ_C: **21.7** (*C*H$_3$), **63.6** (*C*H$_2$), **74.4** (N*C*Ph$_3$), **75.4** (*C*(3)HO), **111.6** (*C*(4``)H), **115.9** (Ar*C*(7)H), **117.0** (Ar*C*(3``)H), **122.3** (Ar*C*(5)H), **124.2** (Ar*C*(5``)H), **125.1** (Ar*C*(4)H), **125.9** (Ar*C*(3a)), **127.0** (Ar*C*(4`)H), **127.3** (Ar*C*(2```,6```)H), **127.8** (Ar*C*(3`,5`)H), **128.2** (Ar*C*(6)H), **129.4** (Ar*C*(2`,6`)H), **129.9** (Ar*C*(3```,5```)H), **131.2**(Ar*C*(2``)), **136.4** (Ar*C*(1```)), **142.1** (Ar*C*(1`)), **143.9** (Ar*C*(7a)), **144.8** (Ar*C*(4```)), **176.7** (*C*=O); **HRMS (ESI$^+$)** $C_{39}H_{32}N_2O_4SNa$ $[M+Na]^+$ found 647.1963, requires 647.1975 (–1.85 ppm).

3-[(3-Methyl-1-tosyl-1*H*-indol-2-yl)methoxy]-1-tritylindolin-2-one (S35)

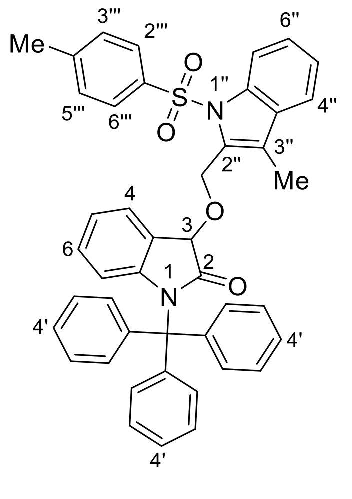

Following **General procedure C**, (3-methyl-1-tosyl-1*H*-indol-2-yl)methanol (0.630 g, 2.0 mmol), 3-diazo-1-tritylindolin-2-one (0.882 g, 2.2 mmol) and $Rh_2(OAc)_4$ (4.41 mg, 0.01 mmol) in CH_2Cl_2 (40 mL, 0.05 M) was added at 0 °C to rt was stirred for 2 h, to give after purification by column chromatography (eluent: Hexane/EtOAc = 5:1 to 3:1), the title compound (1.10 g, 80%) as a pale-yellow solid. **mp** 110–112 °C; **IR** ν_{max} (film) 1726 (C=O), 1595 (C=C), 1463, 1448, 1367 (S=O), 1174 (S=O), 1149 (C-O); **^{1}H NMR (400 MHz, $CDCl_3$)** δ **2.24** (3H, s, ArC(3``)CH_3), **2.29** (3H, s, Ar(C(4```)CH_3), **4.99** (1H, d, *J* 11.5, CH^AH^B), **5.11** (1H, s, C(3)*H*O), **5.43** (1H, d, *J* 11.5, CH^AH^B), **6.24** (1H, dd, *J* 7.5, 1.5, ArC(7)*H*), **6.85–6.94** (2H, m, ArC(5,6)*H*), **7.08** (2H, d, *J* 8.0, ArC(3```,5```)*H*), **7.19–7.34** (12H, m, ArC(4)*H*, ArC(5``,6``)H, ArC(3`,5`)*H*, ArC(4`)H), **7.43** (1H, d, ArC(4``)*H*), **7.49–7.56** (6H, m, ArC(2`,6`)*H*), **7.76–7.83** (6H, m, ArC(2```,6```)*H*), **8.09** (1H, d, *J* 8.3, C(7``)*H*); **$^{13}$C{$^1$H} NMR (101 MHz, $CDCl_3$)** $\delta_C$: **9.1** (ArC(3``)*C*H$_3$), **21.7** (Ar(C(4```)C*H*$_3$), **61.6** (*C*H$_2$), **74.4** (N*C*Ph$_3$), **75.9** (*C*(3)HO), **115.0** (Ar*C*(7``)H), **115.7** (Ar*C*(7)H), **119.5** (Ar*C*(4``)H), **122.0** (Ar*C*(3``)H), **122.4** (Ar*C*(5)H), **123.4** (Ar*C*(5``)H), **125.1** (Ar*C*(4)H), **125.5** (Ar*C*(6``)H), **125.9** (Ar*C*(3a)), **127.0** (Ar*C*(2```,6```)H), **127.0** (Ar*C*(4`)H), **127.8** (Ar*C*(3`,5`)H), **128.4** (Ar*C*(6)H), **129.3** (Ar*C*(2`,6`)H), **129.6** (Ar*C*(3```,5```)H), **130.7** (Ar*C*(3a``)), **132.2** (Ar*C*(2``)), **136.0** (Ar*C*(1```)), **136.6** (Ar*C*(7a``)), **142.2** (Ar*C*(1`)), **144.0** (Ar*C*(7a)), **144.5** (Ar*C*(4```)), **177.0** (*C*=O); **HRMS (ESI$^+$)** $C_{44}H_{36}N_2O_4SNa$ [M+Na]$^+$ found 711.2289, requires 711.2288 (0.12 ppm).

3-(Benzo[b]thiophen-2-ylmethoxy)-1-tritylindolin-2-one (S36)

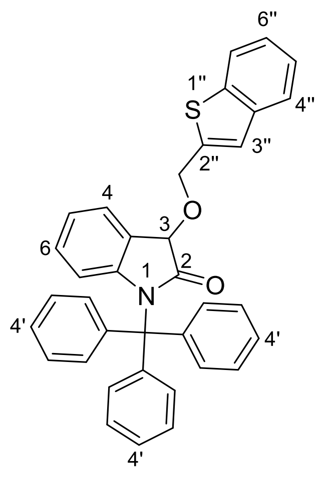

Following **General procedure C**, benzo[b]thiophen-2-ylmethanol (0.328 g, 2.0 mmol), 3-diazo-1-tritylindolin-2-one (0.882 g, 2.2 mmol) and $Rh_2(OAc)_4$ (4.41 mg, 0.01 mmol) in CH_2Cl_2 (40 mL, 0.05 M) was added at 0 °C to rt was stirred for 3 h, to give after purification by column chromatography (eluent: Hexane/EtOAc = 5:1 to 3:1), the title compound (0.430 g, 40%) as a pale-yellow amorphous solid. **IR** ν_{max} (film) 1716 (C=O), 1600 (C=C), 1463, 1448, 1305, 1099 (C-O); **^{1}H NMR (500 MHz, $CDCl_3$)** δ **5.02–5.07** (2H, m, C$H^A$$H^B$, C(3)*H*), **5.15** (1H, dd, *J* 12.5, 1.0, C(3)*H*), **6.25** (1H, dd, *J* 7.5, 1.5, ArC(7)*H*), **6.88–6.95** (2H, m, ArC(5,6)*H*), **7.20–7.23** (3H, m, ArC(4`)*H*), **7.24–7.27** (6H, m, ArC(3`,5`)H), **7.28–7.29** (1H, m, ArC(3``)H), **7.32–7.37** (3H, m, ArC(4)*H*, ArC(6``)*H*, ArC(5``)*H*), **7.45–7.49** (6H, m, ArC(2`,6`)*H*), **7.72–7.75** (1H, m, ArC(4``)*H*), **7.82–7.85** (1H, m, ArC(7``)*H*); **^{13}C{^{1}H} NMR (126 MHz, $CDCl_3$)** δ_C: **66.6** (*C*H$_2$), **74.1** (N*C*Ph$_3$), **74.6** (*C*(3)HO), **116.1** (Ar*C*(7)H), **122.5**

(Ar*C*(5)H), **122.6** (Ar*C*(5``)H), **123.8** (*C*(3``)H), **123.8** (*C*(4``)H), **124.4** (Ar*C*(5``)H), **124.5** (Ar*C*(6``)H), **124.9** (Ar*C*(4)H), **125.8** (Ar*C*(3a)), **127.1** (Ar*C*(4`)H), **127.9** (Ar*C*(3`,5`)H), **128.5** (Ar*C*(6)H), **129.4** (Ar*C*(2`,6`)H), **142.0** (Ar*C*(1`)), **139.5** (*C*(3a``)), **140.6** (*C*(7a``)), **141.6** (*C*(2``)), **144.1** (Ar*C*(7a)), **177.0** (*C*=O); **HRMS (ESI$^+$)** $C_{36}H_{27}NSO_2Na$ $[M+Na]^+$ found 560.1652, requires 560.1655 (–0.55 ppm).

3-[(1,1-Dioxidobenzo[b]thiophen-2-yl)methoxy]-1-tritylindolin-2-one (S37)

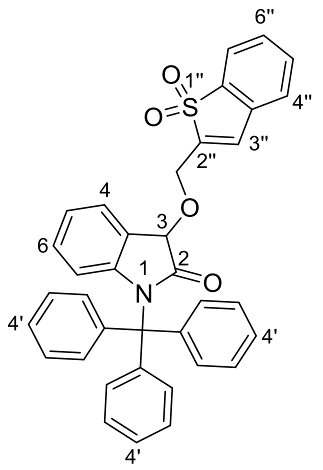

Following **General procedure C**, 2-(hydroxymethyl)benzo[b]thiophene 1,1-dioxide (0.392 g, 2.0 mmol), 3-diazo-1-tritylindolin-2-one (0.882 g, 2.2 mmol) and $Rh_2(OAc)_4$ (4.41 mg, 0.01 mmol) in CH_2Cl_2 (40 mL, 0.05 M) was added at 0 °C to rt was stirred for 3 h, to give after purification by column chromatography (eluent: Hexane/EtOAc = 5:1 to 3:1), the title compound (0.774 g, 68%) as a pale-yellow solid. **mp** 134–136 °C; **IR** ν_{max} (film) 1726 (C=O), 1600 (C=C), 1463, 1446, 1300 (S=O), 1149 (S=O), 1114 (C-O); **^{1}H NMR (500 MHz, CDCl$_3$)** δ **4.76–4.90** (2H, m, C*H*$_2$), **5.10** (1H, s, C(3)HO), **6.25** (1H, dd, *J* 8.0, 1.5, ArC(7)*H*), **6.92** (1H, td, *J* 8.0, 1.5, ArC(6)*H*), **6.96** (1H, td, *J* 8.0, 1.0, ArC(5)*H*), **7.13** (1H, q, *J* 1.0, C(3``)*H*), **7.19–7.23** (3H, m, ArC(4`)*H*), **7.24–7.29** (6H, m, ArC(3`,5`)*H*), **7.31–7.35** (1H, m, ArC(4)*H*), **7.43–7.47** (6H, m, ArC(2`,6`)*H*), **7.47–7.51** (2H, m, ArC(4``,5``)*H*), **7.54** (1H, td, *J* 8.0, 1.5, C(6``)*H*), **7.69–7.74** (1H, d, *J* 8.0, ArC(7``)*H*); **$^{13}$C{$^1$H} NMR (126 MHz, CDCl$_3$)** $\delta_C$: **61.6** (*C*H$_2$), **74.7** (N*C*Ph$_3$), **75.2** (*C*(3)HO), **116.1** (Ar*C*(7)H), **121.6** (Ar*C*(7``)H), **122.9** (Ar*C*(5)H), **125.1** (Ar*C*(3a)), **125.3** (Ar*C*(4)H), **125.3** (Ar*C*(4``)H), **127.1** (Ar*C*(4`)H), **127.9** (Ar*C*(3`,5`)H), **128.8** (Ar*C*(6)H), **129.4** (Ar*C*(2`,6`)H, *C*(3``)H), **130.4** (Ar*C*(5``)H), **130.8** (Ar*C*(3a``)), **133.8** (Ar*C*(6``)), **137.6** (Ar*C*(7a``)), **141.0** (Ar*C*(2``)), **141.9** (Ar*C*(1`)), **144.1** (Ar*C*(7a)), **176.6** (*C*=O); **HRMS (ESI$^+$)** $C_{36}H_{27}NO_4SNa$ $[M+Na]^+$ found 592.1546, requires 592.1553 (–1.16 ppm).

3-{[5-Bromo-1-((4-nitrophenyl)sulfonyl)-1*H*-indol-2-yl]methoxy}-1-tritylindolin-2-one (S38)

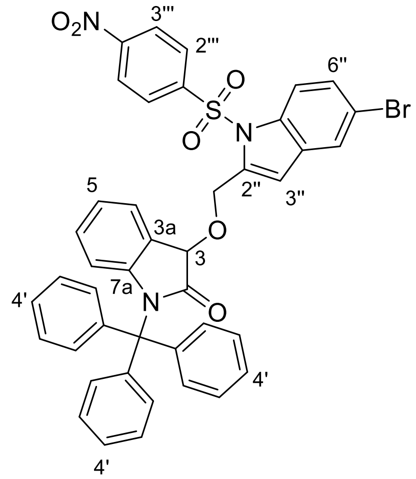

Following **General procedure C**, (5-bromo-1-[(4-nitrophenyl)sulfonyl]-1*H*-indol-2-yl)methanol (0.820 g, 2.0 mmol), 3-diazo-1-tritylindolin-2-one (0.882 g, 2.2 mmol) and $Rh_2(OAc)_4$ (4.41 mg, 0.01 mmol) in CH_2Cl_2 (40 mL, 0.05 M) was added at 0 °C to rt was stirred for 2 h, to give after purification by column chromatography (eluent: Hexane/EtOAc = 5:1 to 3:1), the title compound (0.642 g, 41%) as a yellow solid. **IR** ν_{max} (film) 1728 (C=O), 1606 (C=C), 1531 (NO_2), 1446, 1346, 1305, 1176 (S=O), 1151 (C-O), 1087; **^{1}H NMR (500 MHz, $CDCl_3$)** δ **5.01** (1H, d, *J* 12.8, C*H*^{A}H^B), **5.07** (1H, s, C(3)*H*O), **5.38** (1H, d, *J* 12.8, CH^A*H*^{B}), **6.32** (1H, d, *J* 8.2, ArC(7)*H*), **6.69** (1H, s, C(3``)*H*), **6.80** (1H, t, *J* 7.5, ArC(5)*H*), **6.93** (1H, t, *J* 7.9, ArC(6)*H*), **7.09** (1H, d, *J* 7.4, ArC(4)*H*), **7.18–7.24** (3H, m, ArC(4`)*H*), **7.25–7.30** (6H, m, ArC(3`,5`)*H*), **7.42** (1H, d, *J* 8.9, ArC(6``)*H*), **7.49** (2H, d, *J* 7.8, ArC(2``,6``)*H*), **7.63** (1H, d, *J* 1.9, ArC(4``)*H*), **7.85–7.91** (4H, m, ArC(2```,3```,5```,6```)*H*), **7.95** (1H, d, *J* 8.9, C(7``)*H*); **$^{13}$C{$^1$H} NMR (126 MHz, $CDCl_3$)** $\delta_C$: **64.3** (O*C*$H_2$), **74.6** (N*C*$Ph_3$), **76.0** (*C*(3)HO), **112.2** (*C*(3``)H), **115.8** (Ar*C*(7``)H), **116.3** (Ar*C*(7)H), **117.8** (Ar*C*(5``)Br), **122.4** (Ar*C*(5)H), **124.2** (Ar*C*(4``)H), **124.3** (Ar*C*(3```,5```)H), **124.9** (Ar*C*(4)H), **125.2** (Ar*C*(3a)), **127.2** (Ar*C*(4`)H), **127.9** (Ar*C*(3`,5`)H), **128.4** (Ar*C*(2```,6```)H), **128.8** (Ar*C*(6)H), **129.3** (Ar*C*(2`,6`)H), **131.0** (Ar*C*(3a``)), **135.7** (Ar*C*(2``)), **138.5** (Ar*C*(7a``)), **141.9** (Ar*C*(1`)), **143.5** (Ar*C*(1`)SO_2), **144.1** (Ar*C*(7a)), **150.5** (Ar*C*(4```)NO_2), **176.2** (*C*=O); **HRMS (ESI$^+$)** $C_{42}H_{30}{}^{79}BrN_3O_6SNa$ $[M+Na]^+$ found 806.0921, requires 806.0931 (–1.23 ppm).

3-{[1-(Methylsulfonyl)-1*H*-indol-2-yl]methoxy}-1-tritylindolin-2-one (S39)

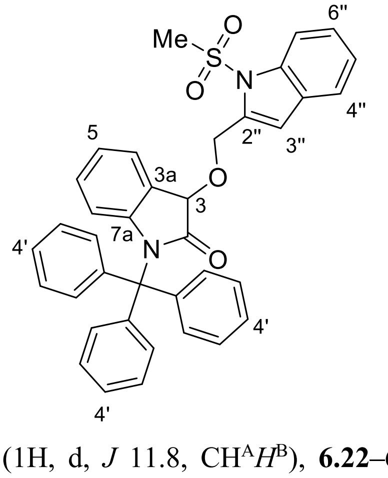

Following **General procedure C**, [1-(methylsulfonyl)-1*H*-indol-2-yl]methanol (0.450 g, 2.0 mmol), 3-diazo-1-tritylindolin-2-one (0.882 g, 2.2 mmol) and $Rh_2(OAc)_4$ (4.41 mg, 0.01 mmol) in CH_2Cl_2 (40 mL, 0.05 M) was added at 0 °C to rt was stirred for 2 h, to give after purification by column chromatography (eluent: Hexane/EtOAc = 6:1 to 3:1), the title compound (0.813 g, 68%) as an amorphous yellow solid. **IR** ν_{max} (film) 1726 (C=O), 1608 (C=C), 1463, 1363 (S=O), 1263, 1171 (S=O), 1153 (C-O), 1058; **^{1}H NMR (500 MHz, $CDCl_3$)** δ **3.21** (3H, s, SO_2CH_3), **5.00–5.09** (2H, m, C(3)*H* and C*H*^{A}H^B), **5.31** (1H, d, *J* 11.8, CH^A*H*^{B}), **6.22–6.28** (1H, m, ArC(7)*H*), **6.73** (1H, s, C(3``)*H*), **6.88–6.99** (2H, m, ArC(5,6)*H*), **7.18–7.23** (3H, m, ArC(4`)*H*), **7.23–7.30** (7H, m, ArC(3`,5`,5``)*H*, **7.32–7.37** (1H, m, ArC(6``)*H*, **7.39–7.42** (1H, m, ArC(4)*H*), **7.45** (6H, d, *J* 7.6, ArC(2`,6`)*H*), **7.55** (1H, d, *J* 7.7, ArC(4``)*H*), **8.05** (1H, d, *J* 8.4, ArC(7``)*H*); **^{13}C{^{1}H} NMR (126 MHz, $CDCl_3$)** δ_C: **41.4** (*C*H_3), **64.9**

(OCH_2), **74.5** ($NCPh_3$), **75.9** (*C*(3)HO), **112.5** (*C*(3``)H), **114.3** (Ar*C*(7``)H), **116.2** (Ar*C*(7)H), **121.5** (Ar*C*(4``)H), **122.4** (Ar*C*(5)H), **123.7** (Ar*C*(5``)H), **125.1** (Ar*C*(4)H), **125.3** (Ar*C*(6``)H), **125.6** (Ar*C*(3a)), **127.1** (Ar*C*(4`)H), **127.9** (Ar*C*(3`,5`)H), **128.7** (Ar*C*(6)H), **128.8** (Ar*C*(3a``)), **129.4** (Ar*C*(2`,6`)H), **136.4** (Ar*C*(2``)), **137.3** (Ar*C*(7a``)), **142.0** (Ar*C*(1`)), **144.2** (Ar*C*(7a)), **176.1** (*C*=O); **HRMS (ESI$^+$)** $C_{37}H_{30}N_2O_4SNa$ $[M+Na]^+$ found 621.1833, requires 621.1818 (2.38 ppm).

3-[(1-Methyl-1H-indol-2-yl)methoxy]-1-tritylindolin-2-on (S40)

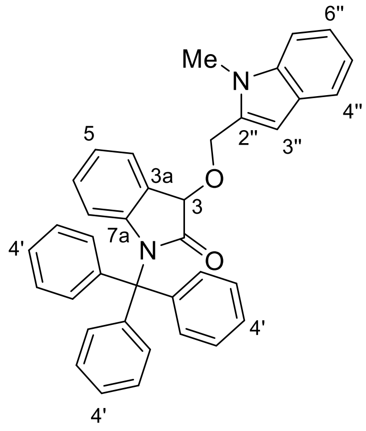

Following **General procedure C**, (1-methyl-1H-indol-3-yl)methanol (0.322 g, 2.0 mmol), 3-diazo-1-tritylindolin-2-one (0.882 g, 2.2 mmol) and $Rh_2(OAc)_4$ (4.41 mg, 0.01 mmol) in CH_2Cl_2 (40 mL, 0.05 M) was added at 0 °C to rt was stirred for 2 h, to give after purification by column chromatography (eluent: Hexane/EtOAc = 10:1 to 5:1), the title compound (0.214 g, 20%) as a amorphous yellow solid. **IR** ν_{max} (film) 1724 (C=O), 1608 (C=C), 1463, 1448, 1338, 1263, 1101 (C-O), 1080; **^{1}H NMR (500 MHz, $CDCl_3$)** δ **3.81** (3H, s, CH_3), **4.82** (1H, d, *J* 12.1, CH^AH^B), **4.94** (1H, d, *J* 12.1, CH^AH^B), **5.04** (1H, s, C(3)*H*O), **6.21–6.28** (1H, m, ArC(7)*H*), **6.51** (1H, s, C(3``)*H*), **6.88–6.94** (2H, m, ArC(5,6)*H*), **7.09** (1H, t, *J* 7.4, ArC(6``)*H*), **7.16** (1H, dd, *J* 5.2, 3.6, ArC(4)*H*), **7.19–7.24** (4H, m, ArC(4`,5``)*H*), **7.24–7.29** (6H, m, ArC(3`,5`)*H*), **7.33** (1H, d, *J* 8.3, ArC(4``)*H*), **7.48** (6H, d, *J* 7.5, 1.8, ArC(2`,6`)*H*), **7.58** (1H, d, *J* 7.9, ArC(7``)*H*); **$^{13}C\{^1H\}$ NMR (126 MHz, $CDCl_3$)** $\delta_C$: **30.2** ($CH_3$), **63.0** ($OCH_2$), **74.0** (*C*(3)HO), **74.6** ($NCPh_3$), **103.8** (*C*(3``)H), **109.4** (Ar*C*(4``)H), **116.1** (Ar*C*(7)H), **119.6** (Ar*C*(6``)H), **121.0** (Ar*C*(7``)H), **122.2** (Ar*C*(5``)H), **122.5** (Ar*C*(5)H), **124.7** (Ar*C*(4)H), **125.9** (Ar*C*(3a)), **127.1** (Ar*C*(4`)H), **127.3** (Ar*C*(3a``)), **127.9** (Ar*C*(3`,5`)H), **128.5** (Ar*C*(6)H), **129.3** (Ar*C*(2`,6`)H), **135.3** (Ar*C*(2``)), **138.4** (Ar*C*(7a``)), **142.1** (Ar*C*(1`)), **144.1** (Ar*C*(7a)), **177.0** (*C*=O); **HRMS (ESI$^+$)** $C_{37}H_{30}N_2O_2Na$ $[M+Na]^+$ found 557.2210, requires 557.2199 (1.87 ppm).

4-[(1-Tosyl-1*H*-indol-2-yl)methyl]-1-tritylindoline-2,3-dione (5)

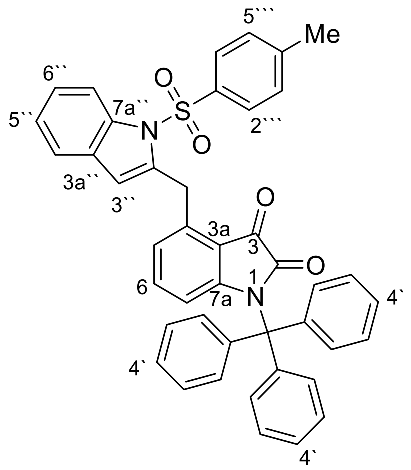

Yellow amorphous solid. **IR** ν_{max} (film) 1732 (C(3)=O), 1593 (C(2)=O), 1490, 1448, 1369 (S=O), 1172 (S=O), 1147, 1089; **^{1}H NMR (400 MHz, $CDCl_3$)** δ **2.32** (3H, s, ArC(4```)C*H*$_3$), **4.76** (2H, s, ArC(4)C*H*$_2$), **6.22, (**1H, s, ArC(3``)*H*), **6.31** (1H, d, *J* 8.2, ArC(7)*H*), **6.79** (1H, d, *J* 7.8, ArC(5)*H*), **7.06** (1H, t, *J* 8.0, ArC(6)*H*), **7.18–7.22** (2H, m, ArC(5``,3```,5```)*H*), **7.23–7.32** (10H, m, ArC(3`,4`,5`,6``)*H*), **7.37** (1H, d, *J* 7.3, ArC(4``)*H*), **7.43–7.49** (6H, m, ArC(2`,6`)*H*), **7.73–7.78** (2H, m, ArC(2```,6```)*H*), **8.15** (1H, dd, *J* 8.3,1.0, ArC(7``)*H*); **^{13}C{^{1}H} NMR (101 MHz, $CDCl_3$)** δ_C: **21.7** (ArC(4```)*C*H$_3$), **31.0** (ArC(4)*C*H$_2$), **75.5** (N*C*Ph$_3$), **111.5** (Ar*C*(3``)H), **115.0** (Ar*C*(7``)H), **116.1** (Ar*C*(7)H), **116.7** (Ar*C*(3a)), **120.6** (Ar*C*(4``)H), **123.8** (Ar*C*(5``)H), **124.5** (Ar*C*(6``)H), **124.7** (Ar*C*(5)H), **126.8** (Ar*C*(2```,6```)H), **127.5** (Ar*C*(4`)H), **128.1** (Ar*C*(3`,5`)H), **129.4** (Ar*C*(2`,6`)H), **129.6** (Ar*C*(3a``)H), **130.0** (Ar*C*(3```,5```)H), **135.6** (Ar*C*(1```)), **136.5** (Ar*C*(6)H), **137.3** (Ar*C*(2``)), **138.2** (Ar*C*(7a``)), **140.8** (Ar*C*(4)CH$_2$), **141.3** (Ar*C*(1`)), **145.1** (Ar*C*(4```)), **152.6** (Ar*C*(7a)), **159.3** (*C*(2)=O); **183.5** (*C*(3)=O); **HRMS (ESI$^+$)** $C_{43}H_{32}N_2O_4SNa$ $[M+Na]^+$ found 695.1975, requires 695.1994 (–0.27 ppm).

(3*R*,3'*S*)-3'-Hydroxy-2-methylene-1-tosyl-1'-trityl-[3,3'-biindolin]-2'-one (3)

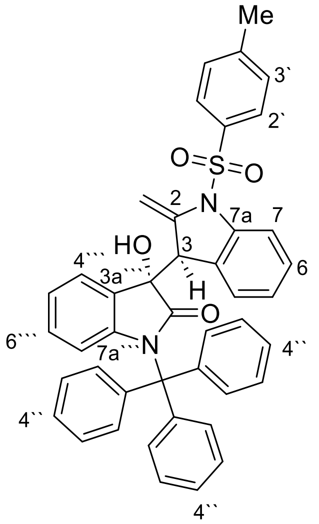

Following **General procedure E**, 3-[(1-tosyl-1*H*-indol-2-yl)methoxy]-1-tritylindolin-2-one (67.4 mg, 0.1 mmol) and *t*Bu-BIMP 3.7 mg, 0.005 mmol) in 1,4-dioxane (2.0 mL, 0.05 M) at 30 °C for 20 h gave a crude [2,3]-rearrangement product which was purified by flash column chromatography (eluent: hexane/acetone = 4:1 to 3:1) to afford the product (51.2 mg, 76%) as a colourless amorphous solid. **IR** ν_{max} (film) 1714 (C=O), 1599 (C=C), 1462, 1361 (S=O), 1170 (S=O), 1089; **$[\alpha]_D^{20}$**= –109.6 (*c* 0.25 in $CHCl_3$); **Chiral HPLC analysis**, Chiralpak ID (80:20 hexane:^{i}PrOH, flow rate 1 ml·min^{-1}, 211 nm, 30 °C) major diastereomer: tR (3*S*,3'*R*)-**3**: 19.0 min, tR (3*R*,3'*R*)-**3**: 38.8 min, 2:98 er; minor diastereomer tR (3*S*,3'*S*)-**3**: 23.0 min, tR (3*R*,3'*S*)-**3**: 27.1 min, 1:99 er; **^{1}H NMR (400 MHz, CD_2Cl_2)** δ **2.38** (3H, s, ArC(4`)C*H*$_3$ (major and minor)), **2.98** (0.81H, s, O*H* (major)), **3.34** (0.18H, s, O*H* (minor)), **4.24** (1H, s, C(3)*H* (major and minor)),)), **4.34** (0.81H, t, *J* 1.9, CC*H*^{A}H^B (major)), **4.86** (0.18H, t, *J* 1.8, CC*H*^{A}H^B (minor)), **5.52** (0.17H, t, *J* 1.8, CCH^A*H*^{B} (minor)), **5.65** (0.82H, t, *J* 1.9, CCH^A*H*^{B} (major)), **6.22–6.26** (0.18H, m, ArC(7```)*H* (minor)), **6.27–6.30** (0.82H, m, ArC(7```)*H* (major)), **6.86** (0.19H, t, *J* 7.4, ArC*H* (minor)), **6.89–6.99** (2.74H, m, ArC*H* (major and minor)), **7.06** (0.22H, td, *J* 7.5, ArC*H* (minor)), **7.18–7.31** (14.12H, m, ArC*H* (major and minor)), **7.41–7.47** (6H, m, ArC(2``,6``)*H* (major and minor)), **7.70–7.73** (0.34H, m, ArC(2`, 6`)*H* (minor)), **7.73–**

7.77 (1.63H, m, ArC(2`, 6`)*H* (major)), **7.81** (1H, d, *J* 8.3, ArC(7)*H* (major)), **7.90** (0.18H, d, *J* 8.3, ArC(7)*H* (minor)); **^{13}C{^{1}H} NMR (101 MHz, CD_2Cl_2)** δ_C: **21.9** (ArC(4`)*C*$H_3$ (major)), **31.2** (ArC(4`)*C*H_3 (minor)), **53.6** (*C*(3)H (major)), **75.5** (N*C*Ph_3 (minor)), **75.6** (N*C*Ph_3 (major)), **76.8** (*C*-OH (minor)), **77.5** (*C*-OH (major)), **97.0** (C=*C*H_2 (minor)), **97.3** (C=*C*H_2 (major)), **114.4** (Ar*C*(7)H (minor)), **114.6** (Ar*C*(7)H (major)), **116.7** (Ar*C*(7```)H (major)), **116.9** (Ar*C*(7)H (minor)), **123.2** (Ar*C*(5```)H (minor)), **123.2** (Ar*C*(5```)H (major)), **123.9** (Ar*C*H (minor)), **124.3** (Ar*C*H (major and minor)), **124.4** (Ar*C*H (major)), **126.3** (Ar*C*H (major)), **126.7** (Ar*C*(3a) (major)), **126.7** (Ar*C*(3a) (minor)), **127.1** (Ar*C*H), **127.4** (Ar*C*(4``)H (minor)), **127.4** (Ar*C*(4``)H (major)), **127.7** (Ar*C*(2`,6`)H (minor)), **127.8** (Ar*C*(2`,6`)H (major)), **128.2** (Ar*C*(3``,5``)H (minor)), **128.2** (Ar*C*(3``,5`)H (major)), **129.0** (Ar*C*(3a) (major)), **129.0** (Ar*C*(6```)H (major)), **129.1** (Ar*C*(6```)H (minor)), **129.6** (Ar*C*H (minor)), **129.7** (Ar*C*(2``,6``)H (minor)), **129.8** (Ar*C*(2``,6``)H (major)), **130.1** (Ar*C*(3`,5`)H (minor)), **130.2** (Ar*C*(3`,5`)H (major)), **134.9** (Ar*C*(1`) (major)), **135.4** (Ar*C*(1`) (minor)), **142.3** (Ar*C*(1``)H (minor)), **142.4** (Ar*C*(1``)H (major)), **143.2** (*C*(2) (major)), **143.9** (Ar*C*(7a) (minor)), **144.0** (Ar*C*(7a) (major)), **144.2** (Ar*C*(minor)), **144.4** (Ar*C*(7a```) (minor)), **144.5** (Ar*C*(7a```) (major)), **145.4** (Ar*C*(4`) (minor)), **145.7** (Ar*C*(4`) (major)), **177.6** ((*C*=O) (major)), **178.1** ((*C*=O) (minor)); **HRMS (ESI$^+$)** $C_{43}H_{34}N_2O_4SNa$ [M+Na]$^+$ found 697.2130, requires 697.2131 (–0.26 ppm).

(3*R*,3'*S*)-4'-Chloro-3'-hydroxy-2-methylene-1-tosyl-1'-trityl-[3,3'-biindolin]-2'-one (8)

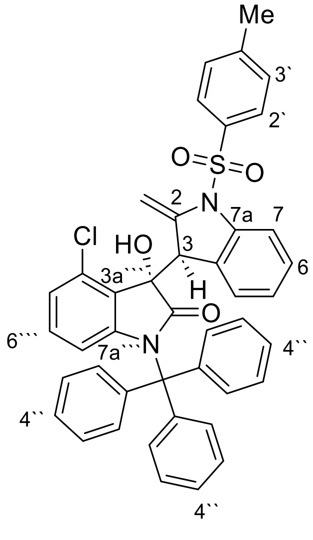

Following **General procedure E**, 4-chloro-3-[(1-tosyl-1*H*-indol-2-yl)methoxy]-1-tritylindolin-2-one (70.8 mg, 0.1 mmol) and *t*Bu-BIMP 3.7 mg, 0.005 mmol) in 1,4-dioxane (2.0 mL, 0.05 M) at 30 °C for 7 h gave a crude [2,3]-rearrangement product which was purified by flash column chromatography (eluent: hexane/acetone = 4:1 to 3:1) to afford the product (52.4 mg, 74%) as a colourless amorphous solid. **IR** ν_{max} (film) 1728 (C=O), 1599 (C=C), 1446, 1356, 1263, 1166 (S=O), 1143, 1085; **$[\alpha]_D^{20}$** = –66.0 (*c* 0.25 in $CHCl_3$); **Chiral HPLC analysis**, Chiralpak IC (90:10 hexane:^{i}PrOH, flow rate 1 ml·min^{-1}, 211 nm, 30 °C) major diastereomer: tR (3*S*,3'*R*)-**8**: 5.3 min, tR (3*R*,3'*S*)-**8**: 6.3 min, 3:97 er; minor diastereomer tR (3*R*,3'*R*)-**8**: 10.9 min, tR (3*S*,3'*S*)-**8**: 15.9 min, 13:87 er; **^{1}H NMR (500 MHz, CD_2Cl_2)** δ **2.37** (2.69H, s, ArC(4`)C*H*$_3$ (major)), **2.39** (0.26H, s, ArC(4`)C*H*$_3$ (minor)), **3.03** (0.88H, s, O*H* (major)), **3.65** (0.13H, s, O*H* (minor)), **3.92** (0.88H, t, *J* 1.9, C=C(2)H^AH^B (major)), **4.81** (0.13H, s, C(3)*H* (minor)), **4.84** (0.87H, s, C(3)*H* (major)), **5.03** (0.13H, t, *J* 1.5, C=C(2)H^AH^B (minor)), **5.60** (0.13H, t, *J* 1.8, C=C(2)H^AH^B (minor)), **5.66** (0.87H, t, *J* 1.9, C=C(2)H^AH^B (major)), **6.19** (0.13H, d, *J* 8.2, ArC(4)*H* (minor)), **6.29** (0.13H, d, *J* 7.5, ArC(7```)*H* (minor)), **6.39** (0.88H, d, *J* 8.0, ArC(7```)*H* (major)), **6.90–7.05** (2.94H, m, ArC(5```,6```)*H* (major and minor), ArCH (major and minor)), **7.11** (0.99H, t, *J* 7.5, ArC(5)*H* (major and minor)), **7.17–7.23** (3.73H, m, ArC(4``)*H* (major and minor) and ArC*H*), **7.24–7.32** (7.30H, m, ArC(3``,5``,3`,5`)*H* (major and

minor) and ArC*H*), **7.38** (0.84H, t, *J* 7.9, ArC(6)*H* (major)), **7.42** (0.28H, t, *J* 8.1, ArC(6)*H* (minor), **7.48** (5.09H, t, *J* 7.7, ArC(2``,6``)*H* (major)), **7.55** (0.99H, d, *J* 7.5, ArC(4)*H* (major and minor)), **7.76** (1.74H, d, *J* 8.1, ArC(2`,6`)*H* (major)), **7.79** (0.26H, d, *J* 8.6, ArC(2`,6`)*H* (minor)), **7.94** (0.88H, d, *J* 8.3, ArC(7)*H* (major)), **8.02** (0.13H, d, *J* 8.3, ArC(7)*H* (minor)); **^{13}C{^{1}H} NMR (126 MHz, CD_2Cl_2)** δ_C: **21.9** (ArC(4`)*C*H_3 (major and minor)), **51.1** (*C*(3)H (major)), **51.3** (*C*(3)H (minor)), **76.0** (N*C*Ph_3 (major)), **76.2** (N*C*Ph_3 (minor)), **76.8** (*C*(3```)OH (minor)), **78.7** (*C*(3```)OH (major)), **96.6** (C=*C*H_2 (major)), **97.1** (C=*C*H_2 (minor)), **114.6** (Ar*C*(7)H (minor)), **114.7** (Ar*C*(7)H (major)), **115.3** (Ar*C*(7```)H (major and minor)), **123.9** (Ar*C*(5```)H (minor)), **124.1** (Ar*C*(5```)H (major)), **124.3** (Ar*C*(5)H (minor)), **124.5** (Ar*C*(5)H (major)), **125.8** (Ar*C*(4```)Cl (major)), **125.9** (Ar*C*(4)H (minor)), **126.0** (Ar*C*(4```)Cl (minor)), **126.7** (Ar*C*(4)H (major)), **126.9** (Ar*C*(3a) (major)), **127.3** (Ar*C*(4``)H (minor)), **127.4** (Ar*C*(4``)H (major)), **127.9** (Ar*C*(2`,6`)H (major)), **128.2** (Ar*C*(3``,5``)H (minor)), **128.3** (Ar*C*(4``)H (major)), **129.7** (Ar*C*(6)H (minor)), **129.8** (Ar*C*(6)H (major)), **130.1** (Ar*C*(6```)H (major)), **130.1** (Ar*C*(3`,5`)H (major)), **130.2** (Ar*C*(3`,5`)H (minor)), **130.3** (Ar*C*(6```)H (minor)), **131.1** (Ar*C*(3a```) (major)), **131.4** (Ar*C*(3a```) (minor)), **134.3** (Ar*C*(1`) (major)), **135.1** (Ar*C*(1`) (minor)), **142.0** (Ar*C*(1``) (minor)), **142.1** (Ar*C*(1``) (major)), **143.4** (Ar*C*(7a) (major)), **143.5** (Ar*C*(7a) (minor)), **143.8** (Ar*C*(2) (major)), **144.3** (Ar*C*(2) (minor)), **145.6** (Ar*C*(4`) (minor)), **145.9** (Ar*C*(4`) (major)), **146.0** (Ar*C*(7a```) (minor)), **146.3** (Ar*C*(7a```) (major)), **176.6** (*C*=O (major)), **177.1** (*C*=O (minor)); **HRMS (ESI$^+$)** $C_{43}H_{33}{}^{35}ClN_2O_4SNa$ [M+Na]$^+$ found 731.1734, requires 731.1734 (0 ppm).

(3*R*,3'*S*)-3'-Hydroxy-2-methylene-5'-nitro-1-tosyl-1'-trityl-[3,3'-biindolin]-2'-one (9)

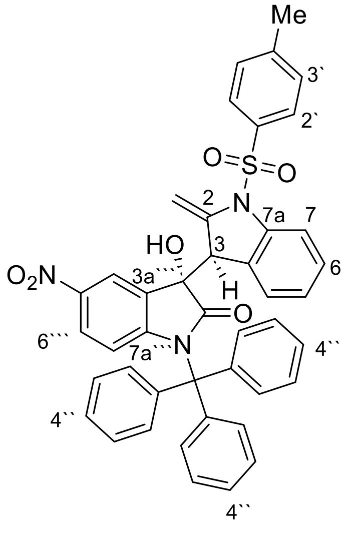

Following **General procedure E**, 5-nitro-3-[(1-tosyl-1*H*-indol-2-yl)methoxy]-1-tritylindolin-2-one (71.9 mg, 0.1 mmol) and *t*Bu-BIMP 3.7 mg, 0.005 mmol) in 1,4-dioxane (2.0 mL, 0.05 M) at 30 °C for 2 min gave a crude [2,3]-rearrangement product which was purified by flash column chromatography (eluent: hexane/acetone = 4:1 to 3:1) to afford the product (57.5 mg, 80%) as a colourless amorphous solid. **IR** ν_{max} (film) 1732 (C=O), 1599 (C=C), 1462, 1355, 1337, 1167 (S=O), 1087; **$[\alpha]_D^{20}$** = –67.2 (*c* 0.25 in $CHCl_3$) **Chiral HPLC analysis**, Chiralpak IA (85:15 hexane:^{i}PrOH, flow rate 1 ml·min^{-1}, 211 nm, 30 °C) major diastereomer: tR (3*S*,3'*R*)-**9**: 23.6 min, tR (3*R*,3'*S*)-**9**: 26.0 min, 2:98 er; minor diastereomer tR (3*S*,3'*S*)-**9**: 31.1 min, tR (3*R*,3'*R*)-**9**: 40.2 min, 99:1 er; **^{1}H NMR (500 MHz, CD_2Cl_2)** δ **2.38** (3H, s, C*H*$_3$), **3.19** (1H, s, O*H*), **4.28** (2H, s, C=CH^AH^B, ArC(3)*H*), **5.69** (1H, s, C=CH^AH^B), **6.44** (1H, d, *J* 9.1, ArC(7```)*H*), **7.02** (1H, t, *J* 7.5, ArC(5)*H*), **7.22–7.34** (13H, m, ArC(3``, 4``,5``,4,6,3`,5`)*H*), **7.38–7.47** (6H, m, ArC(2``, 6``)*H*), **7.75** (2H, d, *J* 8.0, ArC(2`,6`)*H*), **7.83** (2H, d, *J* 8.6, ArC(6```,7)*H*), **8.33** (1H, s, ArC(4```)*H*); **^{13}C{^{1}H} NMR (126 MHz, CD_2Cl_2)** δ_C: **21.9** (*C*H_3), **53.9** (*C*(3)H), **76.5** (N*C*Ph_3), **77.2** (*C*(3```)OH), **97.5** (*C*=CH_2), **114.7** (Ar*C*(7)H), **116.5** (Ar*C*(7```)H), **119.8** (Ar*C*(4```)H), **124.6**

(Ar*C*(5)H), **125.3** (Ar*C*(6```)H), **126.1** (Ar*C*(3a)), **126.4** (Ar*C*(6)H), **127.7** (Ar*C*(2`,6`)H), **127.8** (Ar*C*(4``)H), **128.5** (Ar*C*(3``,5``)H), **129.6** (Ar*C*(2``,6``)H), **130.1** (Ar*C*(4)H, Ar*C*(3a```)), **130.2** (Ar*C*(3`,5`)H), **134.5** (Ar*C*(1`)), **141.5** (Ar*C*(1``)), **143.2** (Ar*C*(7a)), **143.5** (*C*(2)), **143.6** (Ar*C*(5```)NO_2), **145.9** (Ar*C*(4`)), **150.2** (Ar*C*(7a```)), **177.6** (*C*=O); **HRMS (ESI$^+$)** $C_{43}H_{33}N_3O_6SNa$ $[M+Na]^+$ found 742.1977, requires 742.1982 (–0.71 ppm).

(3*R*,3'*S*)-3'-Hydroxy-5'-methyl-2-methylene-1-tosyl-1'-trityl-[3,3'-biindolin]-2'-one (10)

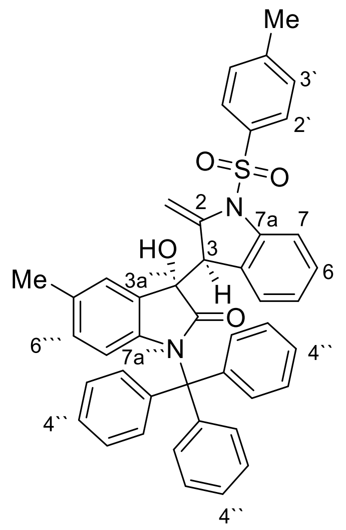

Following **General procedure E**, 5-methyl-3-[(1-tosyl-1*H*-indol-2-yl)methoxy]-1-tritylindolin-2-one (71.9 mg, 0.1 mmol) and *t*Bu-BIMP 3.7 mg, 0.005 mmol) in 1,4-dioxane (2.0 mL, 0.05 M) at 30 °C for 20 h gave a crude [2,3]-rearrangement product which was purified by flash column chromatography (eluent: hexane/acetone = 4:1 to 3:1) to afford the product (57.5 mg, 80%) as a colourless amorphous solid. **IR** ν_{max} (film) 1724 (C=O), 1597 (C=C), 1475, 1448, 1359, 1168 (S=O), 1153, 1132. $[\alpha]_D^{20}$ = –86.4 (*c* 0.25 in $CHCl_3$); **Chiral HPLC analysis**, Chiralpak ID (80:20 hexane:^{i}PrOH, flow rate 1 ml·min^{-1}, 211 nm, 30 °C) major diastereomer: tR (3*S*,3'*R*)-**10**: 17.6 min, tR (3*S*,3'*S*)-**10**: 26.4 min, 2:98 er; minor diastereomer tR (3*R*,3'*S*)-**10**: 22.9 min, tR (3*R*,3'*R*)-**10**: 43.0 min, 97:3 er; **^{1}H NMR (500 MHz, CD_2Cl_2)** δ **2.13** (0.46H, s, ArC(5```)C*H*$_3$ (minor)), **2.25** (2.56H, s, ArC(5```)C*H*$_3$ (major)), **2.38** (3H, s, ArC(4`)C*H*$_3$ (major and minor)), **2.98** (0.85H, s, O*H* (major)), **3.34** (0.13H, s, O*H* (minor)), **4.21** (1H, sa, ArC(3)*H*), **4.35** (0.86H, s, C=CH^AH^B (major)), **4.87** (0.14H, s, C=CH^AH^B (minor)), **5.53** (0.14H, s, C=CH^AH^B (minor)), **5.63** (0.86H, s, C=CH^AH^B (major)), **6.12** (0.14H, d, *J* 8.3, ArC(7```)*H* (minor)), **6.16** (0.85H, d, *J* 8.4, ArC(7```)*H* (major)), **6.73** (1H, d, *J* 8.2, ArC(6```)*H* (major and minor)), **6.98** (0.86H, t, *J* 7.5, ArC(5)*H* (major)), **7.07** (0.19H, t, *J* 7.5, ArC(5)*H* (minor)), **7.15–7.26** (5.34H, m, ArC(4``,4,6)*H* (major and minor)), **7.24–7.31** (8.85H, m, ArC(3``,5``,3,5)*H* (major and minor), ArC(2``,6``)*H* (minor)), **7.33** (1H, s, ArC(4```)*H* (major and minor)), **7.41–7.46** (5.14H, m, ArC(2``, 6``)*H* (major)), **7.71** (0.28H, d, *J* 8.2, ArC(2```,6```)*H* (minor)), **7.76** (1.69H, d, *J* 8.3, ArC(2`,6`)*H* (major)), **7.78** (0.83H, d, *J* 8.8, ArC(7)*H* (major)), **7.89** (0.14H, d, *J* 8.8, ArC(7)*H* (minor)); **^{13}C{^{1}H} NMR (126 MHz, CD_2Cl_2)** δ_C: **21.0** (ArC(5```)*C*H$_3$ (major and minor)), **21.9** (ArC(4`)*C*H$_3$ (major and minor), **53.7** (*C*(3)H (major and minor), **75.4** (N*C*Ph$_3$ (minor)), **75.6** (N*C*Ph$_3$) (minor)), **76.9** (*C*(3```)OH (minor)), **77.6** (*C*(3```)OH (major)), **96.9** (C(2)=*C*H$_2$ (minor)), **97.1** (C(2)=*C*H$_2$ (major)), **114.3** (Ar*C*(7)H (minor)), **114.5** (Ar*C*(7)H (major)), **116.4** (Ar*C*(7```)H (major)), **116.6** (Ar*C*(7```)H (minor)), **123.9** (Ar*C*(5)H (minor)), **124.3** (Ar*C*(5)H (major)), **125.0** (Ar*C*(4```)H (major and minor)), **126.3** (Ar*C*H (major)), **126.7** (Ar*C*H (minor)), **127.1** (Ar*C*(3a) (major)), **127.3** (Ar*C*(4``)H (minor)), **127.4** (Ar*C*(4``)H (major)), **127.7** (Ar*C*(2`,6`)H (major and minor)), **128.1** (Ar*C*(3``,5``)H (minor)), **128.3** (Ar*C*(3``,5``)H (major)), **128.8** (Ar*C*(3a```) (major)), **129.4** (Ar*C*(6```)H (major)), **129.5** (Ar*C*(6```)H (minor)), **129.6** (Ar*C*H (major)), **129.7** (Ar*C*(2``,6``)H (minor)), **129.8**

(Ar*C*(2``,6``)H (major)), **130.2** (Ar*C*(3`,5`)H (major and minor)), **132.9** (Ar*C*(5```)CH_3 (minor)), **133.1** (Ar*C*(5```)CH_3 (major)), **135.0** (Ar*C*(1`) (major)), **135.5** (Ar*C*(1`) (minor)), **141.8** (Ar*C*(7a```) (minor)), **141.9** (Ar*C*(7a```) (major)), **142.4** (Ar*C*(1``) (major and minor)), **143.2** (Ar*C*(7a) (major)), **143.9** (Ar*C*(7a) (minor)), **144.0** (*C*(2) (major)), **144.2** (*C*(2) (minor)), **145.4** (Ar*C*(4`) (minor)), **145.7** (Ar*C*(4`) (major)), **177.6** ((*C*=O) (major)), **178.1** ((*C*=O) (minor)); **HRMS (ESI$^+$)** $C_{44}H_{36}N_2O_4SNa$ $[M+Na]^+$ found 711.2275, requires 711.2288 (–1.83 ppm).

(3*R*,3'*S*)-3'-Hydroxy-5'-methoxy-2-methylene-1-tosyl-1'-trityl-[3,3'-biindolin]-2'-one (11)

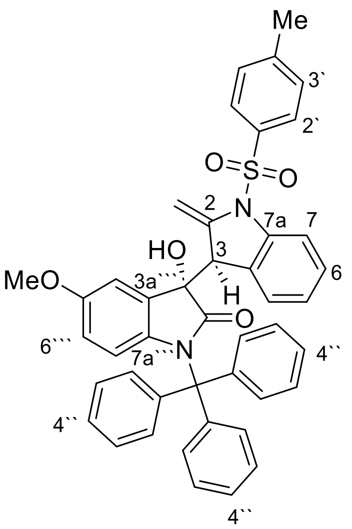

Following **General procedure E**, 5-methoxy-3-[(1-tosyl-1*H*-indol-2-yl)methoxy]-1-tritylindolin-2-one (70.4 mg, 0.1 mmol) and *t*Bu-BIMP 7.4 mg, 0.010 mmol) in 1,4-dioxane (2.0 mL, 0.05 M) at 30 °C for 24 h gave a crude [2,3]-rearrangement product which was purified by flash column chromatography (eluent: hexane/acetone = 4:1 to 3:1) to afford the product (45.8 mg, 65%) as a pale-yellow amorphous solid. **IR** ν_{max} (film) 1724 (C=O), 1597 (C=C), 1485, 1265, 1170 (S=O), 1089; **$[\alpha]_D^{20}$** = –107.2 (*c* 0.25 in $CHCl_3$); **Chiral HPLC analysis**, Chiralpak IA (85:15 hexane:^{i}PrOH, flow rate 1 ml·min^{-1}, 211 nm, 30 °C) major diastereomer: tR (3*R*,3'*S*)-**11**: 32.0 min, tR (3*S*,3'*R*)-**11**: 41.8 min, 98:2 er; **^{1}H NMR (500 MHz, CD_2Cl_2)** δ **2.34** (0.33H, s, ArC(4`)C*H*$_3$ (minor)), **2.37** (2.80H, s, ArC(4`)C*H*$_3$ (major)), **2.97** (0.90H, s, O*H* (major)), **3.21** (0.10H, s, O*H* (minor)), **3.50** (0.29H, s , OC*H*$_3$ (minor)), **3.74** (2.72H, s , OC*H*$_3$ (majaor)), **4.19** (0.90H, s, C=C*H*^{A}H^B (major)), **4.21** (0.10H, s, C=C*H*^{A}H^B (minor)), **4.40** (0.90H, t, *J* 1.8, C(3)*H* (major)), **4.84** (0.10H, t, *J* 1.7, C(3)*H* (minor)), **5.53** (0.10H, t, *J* 1.8, C=CH^A*H*^{B} (minor)), **5.65** (0.90H, t, *J* 1.8, C=C(2)H^A*H*^{B} (major)), **6.15** (0.90H, d, *J* 9.0, ArC(7```)*H* (major)), **6.23** (0.10H, d, *J* 8.9, ArC(7```)*H* (minor)), **6.44** (0.90H, dd, *J* 9.0, 2.8, ArC(6```)*H* (major)), **6.52** (0.10H, dd, *J* 9.0, 2.8, ArC(6```)*H* (minor)), **6.68** (0.10H, d, *J* 2.8, ArC(4```)*H* (minor)), **6.93** (0.10H, d, *J* 7.5, ArC(5)*H* (minor), **6.97** (0.91H, td, *J* 7.5, 1.0, ArC(5)*H* (major), **7.08** (0.90H, d, *J* 2.8, ArC(4```)*H* (major)), **7.17–7.25** (5.18H, m, ArC(4``,4)*H* (major and minor) and ArC*H*), **7.24–7.28** (8.26H, m, ArC(3``,5``,6,3`,5`)*H* (major and minor) and ArC*H*), **7.37–7.44** (5.40H, m, ArC(2``,6``)*H* (major)), **7.46–7.50** (0.60H, m, ArC(2``,6``)*H* (minor)), **7.60** (0.20H, d, *J* 8.4, ArC(2`,6`)*H* (minor)), **7.75** (1.79H, d, *J* 8.4, ArC(2`,6`)*H* (major)), **7.79** (0.90H, d, *J* 8.3, ArC(7)*H* (major)), **7.86** (0.10H, d, *J* 8.2, ArC(7)*H* (minor)); **^{13}C{^{1}H} NMR (126 MHz, CD_2Cl_2)** δ_C: **21.9** (ArC(4`)*C*H_3 (major and minor)), **53.6** (*C*(3)H (major and minor)), **56.0** (O*C*H_3 (minor)), **56.1** (O*C*H_3 (major)), **75.6** (N*C*Ph_3 (major)), **76.9** (*C*(3```)OH (minor)), **77.8** (*C*(3```)OH (major)), **96.9** (C=*C*H_2 (minor)), **97.2** (C=*C*H_2 (major)), **109.9** (Ar*C*(4```)H (major)), **111.7** (Ar*C*(4```)H (minor)), **114.1** (Ar*C*(6```)H (minor)), **114.3** (Ar*C*(7)H (minor)), **114.4** (Ar*C*(7)H (major)), **114.6** (Ar*C*(6```)H (major)), **117.5** (Ar*C*(7```)H (major)), **117.6** (Ar*C*(7```)H (minor)), **124.3** (Ar*C*(5)H (major)), **124.4** (Ar*C*(5)H (minor)), **126.3** (Ar*C*(4`)H (major)), **126.8** (Ar*C*), **126.8** (Ar*C*(2```,6```)H (minor)), **127.0** (Ar*C*(3a)

(major)), **127.4** (Ar*C*(4``)H (major)), **127.5** (Ar*C*(4``)H (minor)), **127.5** (Ar*C*(2`,6`)H (minor)), **127.7** (Ar*C*(2`,6`)H (major)), **128.1** (Ar*C*(3``,5``)H (minor)), **128.2** (Ar*C*(3``,5``)H (major)), **129.5** (Ar*C*H), **129.6** (Ar*C*H), **129.8** (Ar*C*(2``,6``) (major)), **130.2** (Ar*C*(3`,5`)H (major)), **130.5** (Ar*C*(3`,5`)H (minor)), **134.8** (Ar*C*(1`) (major)), **137.3** (Ar*C*(7a```) (minor)), **137.4** (Ar*C*(7a```) (major)), **142.4** (Ar*C*(1``) (major and minor)), **143.2** (Ar*C*(7a) (major)), **143.9** (Ar*C*(2) (major)), **145.7** (Ar*C*(4`) (major)), **145.8** (Ar*C*(4`) (minor)), **156.3** (Ar*C*(5```)OCH_3 (major)), **156.3** (Ar*C*(5```)OCH_3 (minor)), **177.1** (*C*=O (minor)), **177.5** (*C*=O (major)); **HRMS (ESI$^+$)** $C_{44}H_{36}N_2O_5SNa$ $[M+Na]^+$ found 727.2232, requires 727.2237 (–0.71 ppm).

(3*R*,3'*S*)-6'-Chloro-3'-hydroxy-2-methylene-1-tosyl-1'-trityl-[3,3'-biindolin]-2'-one (12)

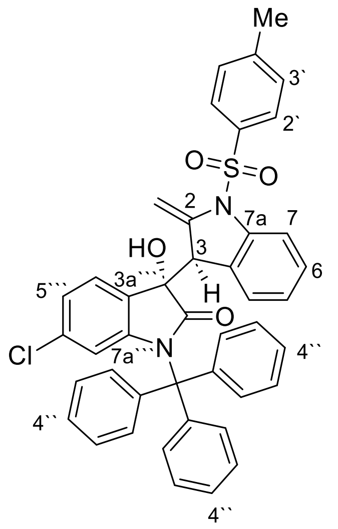

Following **General procedure E**, 6-chloro-3-[(1-tosyl-1*H*-indol-2-yl)methoxy]-1-tritylindolin-2-one (70.8 mg, 0.1 mmol) and *t*Bu-BIMP 3.7 mg, 0.005 mmol) in 1,4-dioxane (2.0 mL, 0.05 M) at 30 °C for 7 h gave a crude [2,3]-rearrangement product which was purified by flash column chromatography (eluent: hexane/acetone = 4:1 to 3:1) to afford the product (51.0 mg, 72%) as a colourless amorphous solid. **IR** ν_{max} (film) 1732 (C=O), 1600 (C=O), 1473, 1448, 1359 (S=O), 1170 (S=O), 1112, 1078; $[\alpha]_D^{20}$ = –96.4 (*c* 0.25 in $CHCl_3$); **Chiral HPLC analysis**, Chiralpak ID (90:10 hexane:^{i}PrOH, flow rate 1 ml·min^{-1}, 211 nm, 30 °C) major diastereomer: tR (3*S*,3'*R*)-**12**: 12.4 min, tR (3*R*,3'*S*)-**12**: 18.0 min, 4:96 er; **^{1}H NMR (500 MHz, CD_2Cl_2)** δ **2.38** (3H, s, ArC(4`)CH_3 (major and minor)), **3.08** (0.89H, s, O*H* (major)), **3.41** (0.11H, s, O*H* (minor)), **4.20** (0.88H, s, C(3)*H* (major)), **4.21** (0.12H, s, C(3)*H* (minor)), **4.44** (0.88H, s, C=CH^AH^B (major)), **4.79** (0.12H, s, C=CH^AH^B (minor)), **5.51** (0.11H, t, *J* 1.9, C=CH^AH^B (minor)), **5.67** (0.89H, t, *J* 1.9, C=CH^AH^B (major)), **6.18** (0.12H, d, *J* 1.8, ArC(7```)*H* (minor)), **6.21** (0.88H, d, *J* 1.8, ArC(7```)*H* (major)), **6.88** (0.25H, dd, *J* 8.0, 1.8, ArC(5```)*H* (minor)), **6.93** (0.92H, dd, *J* 8.0, 1.8, ArC(5```)*H* (major)), **6.99** (0.87H, dd, *J* 7.6, 1.0, ArC(5)*H* (major)), **7.03** (0.19H, d, *J* 8.0, ArC(4```,5)*H* (minor)), **7.14** (0.90H, d, *J* 7.5, ArC(4```)*H* (major)), **7.24–7.32** (12.64H, m, ArC(3``,4``,5``,6,3`,5`)*H* (major and minor) and ArC*H*), **7.37** (0.96H, d, *J* 8.0, ArC(4```)*H* (major)), **7.39–7.44** (5.45H, m, ArC(2``,6``)*H* (major)), **7.59** (0.44H, d, *J* 8.4, ArC(2``,6``)*H* (minor)), **7.69** (0.25H, d, *J* 8.4, ArC(2`,6`)*H* (minor)), **7.75** (1.80H, d, *J* 8.4, ArC(2`,6`)*H* (major)), **7.80** (0.89H, d, *J* 8.3, ArC(7)*H* (major)), **7.88** (0.11H, d, *J* 8.3, ArC(7)*H* (minor)); **^{13}C{^{1}H} NMR (126 MHz, CD_2Cl_2)** δ_C: **21.9** (ArC(4`)CH_3 (major and minor)), **53.4** (*C*(3)H (major and minor)), **75.0** (N*C*Ph_3 (minor)), **75.9** (N*C*Ph_3 (major)), **76.1** (*C*(3```)OH (minor)), **77.1** (*C*(3```)OH (major)), **97.0** (C=*C*H_2 (minor)), **97.5** (C=*C*H_2 (major)), **114.4** (Ar*C*(7)H (minor)), **114.6** (Ar*C*(7)H (minor)), **117.0** (Ar*C*(7```)H (major)), **117.1** (Ar*C*(7```)H (major)), **123.2** (Ar*C*(5```)H (major)), **123.3** (Ar*C*(5```)H (minor)), **124.4** (Ar*C*(5)H (major)), **124.5** (Ar*C*(5)H (minor)), **125.2** (Ar*C*(4```)H (major)), **126.1** (Ar*C*(4)H (major)), **126.2** (Ar*C*H (minor)), **126.6** (Ar*C*(3a) (major)), **126.8** (Ar*C*(2`,6`)H (minor)), **127.3** (Ar*C*(3a```) (major)),

127.7 (Ar*C*(4``)H (major and minor)), **127.7** (Ar*C*(2`,6`)H (major)), **128.3** (Ar*C*(3``,5``)H (minor)), **128.3** (Ar*C*(3``,5``)H (major)), **128.5** (Ar*C*H), **129.5** (Ar*C*H), **129.7** (Ar*C*(2``,6``) (minor)), **129.7** (Ar*C*(2``,6``) (major)), **130.2** (Ar*C*(3`,5`)H (major)), **130.5** (Ar*C*(3`,5`)H (minor)), **134.6** (Ar*C*(6```)Cl (major)), **137.3** (Ar*C*(1`) (majjor)), **134.8** (Ar*C* (minor)), **135.0** (Ar*C* (minor)), **141.8** (Ar*C*(1``) (major)), **141.9** (Ar*C*(1``) (minor)), **143.2** (Ar*C*(7a) (major)), **143.6** (Ar*C*(7a) (minor)), **143.7** (Ar*C*(2) (major)), **144.1** (Ar*C*(2) (minor)), **145.5** (Ar*C*(7a```) (minor)), **145.6** (Ar*C*(7a```) (major)), **145.8** (Ar*C*(4`) (major)), **145.9** (Ar*C*(4`) (minor)), **177.0** (*C*=O (minor)), **177.5** (*C*=O (major)). **HRMS (ESI$^+$)** $C_{43}H_{33}{}^{35}ClN_2O_4SNa$ $[M+Na]^+$ found 731.1743, requires 731.1742 (0.17 ppm).

(3*R*,3'*S*)-6'-Bromo-3'-hydroxy-2-methylene-1-tosyl-1'-trityl-[3,3'-biindolin]-2'-one (13)

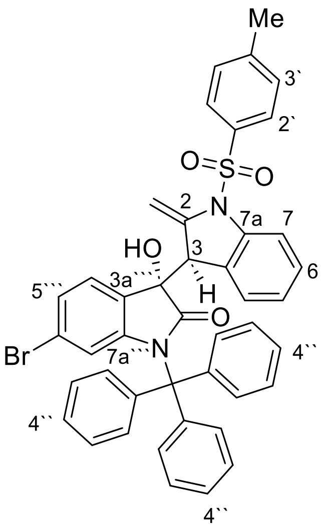

Following **General procedure E**, 6-bromo-3-[(1-tosyl-1*H*-indol-2-yl)methoxy]-1-tritylindolin-2-one (75.2 mg, 0.1 mmol) and *t*Bu-BIMP 3.7 mg, 0.005 mmol) in 1,4-dioxane (2.0 mL, 0.05 M) at 30 °C for 1 h gave a crude [2,3]-rearrangement product which was purified by flash column chromatography (eluent: hexane/acetone = 4:1 to 3:1) to afford the product (54.9 mg, 73%) as a colourless amorphous solid. **IR** ν_{max} (film) 1732 (C=O), 1601 (C=C), 1473, 1448, 1359, 1168 (C-O), 1118, 1087; $[\alpha]_D^{20}$ = –117.2 (*c* 0.25 in $CHCl_3$); **Chiral HPLC analysis**, Chiralpak IA (85:15 hexane:^{i}PrOH, flow rate 1 ml·min^{-1}, 211 nm, 30 °C) major diastereomer: tR (3*S*,3'*R*)-**13**: 19.6 min, tR (3*R*,3'*S*)-**13**: 25.9 min, 3:97 er; **^{1}H NMR (500 MHz, CD_2Cl_2)** δ **2.38** (3H, s, ArC(4`)C*H*$_3$ (major and minor)), **3.00** (0.88H, s, O*H* (major)), **3.32** (0.12H, s, O*H* (minor)), **4.20** (0.10H, s, C(3)*H* (major and minor)), **4.44** (0.92H, s, C=C*H*^{A}H^B (major)), **4.79** (0.08H, s, C=C*H*^{A}H^B (minor)), **5.50** (0.09H, t, *J* 1.9, C=C(2) H^A*H*^{B} (minor)), **5.67** (0.92H, t, *J* 1.9, C=C(2)H^A*H*^{B} (major)), **6.34** (0.91H, d, *J* 1.9, ArC(7```)*H* (major)), **6.38** (0.08H, d, *J* 1.8, ArC(7```)*H* (minor)), **6.95** (1H, t, *J* 7.5, ArC(5)*H* (major and minor)), **7.08** (1H, dd, *J* 8.0, 1.7, ArC(5```)*H* (major)), **7.14** (0.98H, d, *J* 7.6, ArC(4)*H* (major)), **7.16–7.18** (0.47H, m, ArC*H* (minor) and ArC*H*), **7.22–7.32** (13H, m, ArC(4```,3``,4``,5``,6,3`,5`)*H* (major and minor) and ArC*H*), **7.40** (5.54H, d, *J* 7.5, ArC(2``,6``)*H* (major)), **7.46–7.52** (0.40H, m, ArC(2``,6``)*H* (minor)), **7.68** (0.16H, d, *J* 7.8, ArC(2`,6`)*H* (minor)), **7.74** (1.81H, d, *J* 8.1, ArC(2`,6`)*H* (major)), **7.79** (0.92H, d, *J* 8.4, ArC(7)*H* (major)), **7.88** (0.08H, d, *J* 8.4, ArC(7)*H* (minor)); **^{13}C{^{1}H} NMR (126 MHz, CD_2Cl_2)** δ_C: **21.9** (ArC(4`)*C*H$_3$ (major and minor)), **53.4** (*C*(3)H (major and minor)), **75.9** (N*C*Ph$_3$ (major)), **77.1** (*C*(3```)OH (major)), **97.1** (C=*C*H$_2$ (minor)), **97.5** (C=*C*H$_2$ (major)), **114.5** (Ar*C*(7)H (minor)), **114.7** (Ar*C*(7)H (major)), **119.6** (Ar*C*(7```)H (minor)), **119.8** (Ar*C*(7```)H (major)), **122.7** (Ar*C*(6```)Br (major)), **122.9** (Ar*C*(6```)Br (minor)), **124.5** (Ar*C*(5)H (major and minor)), **125.5** (Ar*C*(4```)H (major)), **126.2** (Ar*C*(5```,4)H (major)), **126.6** (Ar*C*(3a) (major)), **127.7** (Ar*C*(4``)H (major and minor)), **127.8** (Ar*C*(2`,6`)H (major and minor)), **128.3** (Ar*C*(3``,5``) (minor)), **128.4** (Ar*C*(3``,5``) (major)), **128.5** (Ar*C*H (major)), **128.6**

(Ar*C*H (minor)), **129.5** (Ar*C*H), **129.8** (Ar*C*(2`,6`)H), **130.2** (Ar*C*(3`,5`)H (major)), **130.5** (Ar*C*(3`,5`)H (minor)), **134.8** (Ar*C*(1`) (major)), **141.8** (Ar*C*(1``) (major and minor)), **143.2** (Ar*C*(7a) (major)), **143.8** (Ar*C*(2) (major)), **145.7** (Ar*C*(7a```) (major)), **145.8** (Ar*C*(4`) (major)), **177.4** (*C*=O (major)). **HRMS (ESI$^+$)** $C_{43}H_{33}{}^{79}BrN_2O_4SNa$ [M+Na]$^+$ found 775.1232, requires 775.1237 (–0.65 ppm).

(3*R*,3'*S*)-5-Bromo-3'-hydroxy-2-methylene-1-tosyl-1'-trityl-[3,3'-biindolin]-2'-one (14)

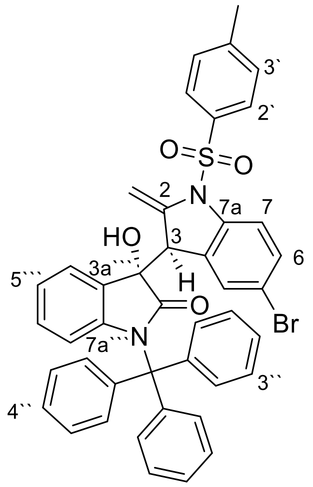

Following **General procedure E**, 3-[(5-bromo-1-tosyl-1*H*-indol-2-yl)methoxy]-1-tritylindolin-2-one (75.2 mg, 0.1 mmol) and *t*Bu-BIMP 3.7 mg, 0.005 mmol) in 1,4-dioxane (2.0 mL, 0.05 M) at 30 °C for 5 h gave a crude [2,3]-rearrangement product which was purified by flash column chromatography (eluent: hexane/acetone = 4:1 to 3:1) to afford the product (58.7 mg, 78%) as a colourless amorphous solid. **IR** ν_{max} (film) 1716 (C=O), 1606 (C=C), 1595, 1463, 1448, 1361 , 1170 (S=O), 1089; $[\alpha]_D^{20}$ = –47.6 (*c* 0.25 in $CHCl_3$); **Chiral HPLC analysis**, Chiralpak ID (80:20 hexane:^{i}PrOH, flow rate 1 ml·min^{-1}, 211 nm, 30 °C) major diastereomer: tR (3*S*,3'*S*)-**14**: 15.5 min, tR (3*S*,3'*R*)-**14**: 19.7 min, tR (3*R*,3'*R*)-**14**: 27.1 min, tR (3*R*,3'*S*)-**14**: 35.9 min, 96:4 er; **^{1}H NMR (500 MHz, CDCl$_3$)** δ **2.39** (3H, s, C*H*$_3$ (major and minor)), **2.90** (0.89H, s, O*H* (major), **3.32** (0.12H, s, O*H* (minor), **4.20** (1H, s, C(3)*H* (major and minor)), **4.31** (0.87H, s, C=C*H*^{A}H^B (major)), **4.87** (0.12H, s, C=C*H*^{A}H^B (minor)), **5.51** (0.12H, s, C=CH^A*H*^{B} (minor)), **5.61** (0.90H, s, C=CH^A*H*^{B} (major) (major and minor)), **6.28–6.30** (0.13H, m, ArC*H* (minor)), **6.32** (1H, d, *J* 8.1, ArC(7```)*H*), **6.93** (1H, t, *J* 7.9, ArC(6```)*H* (major and minor)), **6.99** (1H, t, *J* 7.4, ArC(5```)*H* (major and minor)), **7.17** (0.81H, d, *J* 7.8, ArC*H*), **7.20–7.23** (3.29H, m, ArC*H* (major and minor)), **7.25–7.30** (7.71H, m, ArC*H* (major and minor)), **7.39–7.47** (7.83H, m, ArC(2``,6``)*H* (major and minor) and ArC*H*), **7.57** (1H, s, ArC*H*), **7.68** (0.24H, d, *J* 8.1, ArC(2`,6`)*H* (minor)), **7.72** (2.72H, d, *J* 8.3, ArC(7,2`,6`)*H* (major)), **7.81** (0.13H, d, *J* 8.8, ArC(7)*H* (minor)); **^{13}C{^{1}H} NMR (126 MHz, CDCl$_3$)** δ_C **21.9** (*C*H$_3$ (major and minor)), **53.6** (*C*(3)H (major and minor)), **75.9** (N*C*Ph$_3$ (major and minor)), **77.7** (*C*(3```)OH (major and minor)), **97.4** (C=*C*H$_2$ (minor)), **97.5** (C=*C*H$_2$ (major)), **115.8** (Ar*C*(7)H (minor)), **116.0** (Ar*C*(7)H (major)), **117.0** (Ar*C*(7```)H (major and minor)), **123.4** (Ar*C*(5```)H (major and minor)), **124.1** (Ar*C*(4```)H (minor)), **124.4** (Ar*C*(4```)H (major)), **127.5** (Ar*C*(4``)H (major and minor)), **127.8** (Ar*C*(2`,6`)H (major and minor)), **128.3** (Ar*C*(3``,5``)H (major and minor)), **128.6** (Ar*C*(3a```) (major and minor)), **129.2** (Ar*C*(6```) (major and minor)), **129.4** (Ar*C*(3a) (major and minor)), **129.5** (Ar*C*H), **129.6** (Ar*C*H), **129.8** (Ar*C*(2``,6``)H (major)), **130.3** (Ar*C*(3`,5`)H (major and minor)), **132.6** (Ar*C*H (major)), **132.9** (Ar*C*H (minor)), **134.5** (Ar*C*(1`) (major and minor)), **142.2** (Ar*C*(1``) (major and minor)), **142.5** (Ar*C*(7a) (major and minor)), **143.4** (Ar*C*(2) (minor)), **143.5** (Ar*C*(2) (major)), **144.5** (Ar*C*(7a```) (major and minor)), **145.7** (Ar*C*(4`) (minor)), **146.0** (Ar*C*(4`) (major)), **177.7** (*C*=O (major and minor)); **HRMS (ESI$^+$)** $C_{43}H_{33}{}^{79}BrN_2O_4SNa$ [M+Na]$^+$ found 775.1246, requires 775.1237 (1.16 ppm).

(3*R*,3'*S*)-3'-Hydroxy-5-methyl-2-methylene-1-tosyl-1'-trityl-[3,3'-biindolin]-2'-one (15)

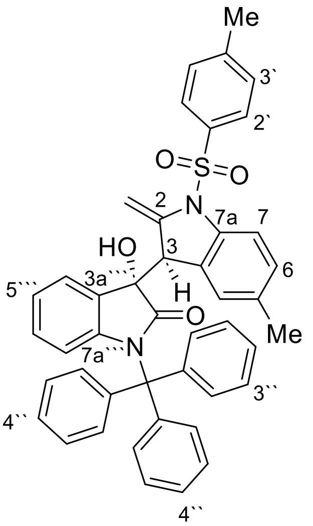

Following **General procedure E**, 3-[(5-methyl-1-tosyl-1*H*-indol-2-yl)methoxy]-1-tritylindolin-2-one (68.8 mg, 0.1 mmol) and *t*Bu-BIMP 3.7 mg, 0.005 mmol) in 1,4-dioxane (2.0 mL, 0.05 M) at 30 °C for 20 h gave a crude [2,3]-rearrangement product which was purified by flash column chromatography (eluent: hexane/acetone = 4:1 to 3:1) to afford the product (56.4 mg, 82%) as a colourless amorphous solid. **IR** ν_{max} (film) 1724 (C=O), 1597 (C=C), 1489, 1359, 1263, 1168 (S=O), 1132; $[\alpha]_D^{20}$ = –39.6 (*c* 0.25 in CHCl₃); **Chiral HPLC analysis**, Chiralpak ID (80:20 hexane:^{i}PrOH, flow rate 1 ml·min^{-1}, 211 nm, 30 °C) minor diastereomer: tR (3*S*,3'*S*)-**15**: 21.1 min, tR (3*S*,3'*R*)-**15**: 44.7 min, 97:3 er; major diastereomer tR (3*R*,3'*R*)-**15**: 25.0 min, tR (3*R*,3'*S*)-**15**: 66.0 min, 98:2 er; **^{1}H NMR (500 MHz, CD_2Cl_2)** δ **2.26** (0.77H, s, ArC(5)CH_3 (minor)), **2.28** (2.20H, s, ArC(5)CH_3 (major)), **2.38** (3H, s, ArC(4`)CH_3 (major and minor)), **2.97** (0.75H, s, O*H* (major)), **3.36** (0.24H, s, O*H* (minor)), **4.17** (0.25H, s, C(3)*H* (minor)), **4.18** (0.75H, s, C(3)*H* (major)), **4.29** (0.75H, s, C=CH^AH^B (major)), **4.86** (0.26H, s, C=CH^AH^B (minor)), **5.53** (0.25H, t, *J* 1.8, C=CH^AH^B (minor)), **5.61** (0.75H, t, *J* 1.8, C=CH^AH^B (major)), **6.25** (0.24H, d, *J* 8.1, ArC(7```)*H* (minor)), **6.30** (0.76H, d, *J* 8.1, ArC(7```)*H* (major)), **6.40** (0.24H, s, ArC*H* (minor)), **6.90–6.94** (1H, m, ArC(6```)*H* (major and minor)), **6.94–7.01** (1H, m, ArC(5```)*H* (major and minor)), **7.12–7.18** (2.50H, m, ArC*H* (major and minor)), **7.19–7.24** (6H, m, ArC(4``,3,5)*H* (major and minor), ArC*H*), **7.25–7.29** (6H, m, ArC(3``,5``)*H* (major and minor)), **7.39–7.43** (4.54H, m, ArC(2``,6``)*H* (major)), **7.46** (0.75H, dd, *J* 7.4, 1.5, ArC(4```)*H* (major)), **7.71** (1.22H, d, *J* 8.4, ArC(7)*H* (major) and ArC(2`,6`)H (minor)), **7.74** (1.48H, d, *J* 8.4, ArC(2`,6`)H (major)), **7.79** (0.24H, d, *J* 8.4, ArC(7)H (minor)); **^{13}C{^{1}H} NMR (126 MHz, CD_2Cl_2)** δ_C: **21.2** (ArC(5```)*C*H₃ (major)), **21.9** (ArC(4`)*C*H₃ (major), **54.0** (*C*(3)H (major and minor), **75.5** (N*C*Ph₃ (minor)), **75.8** (N*C*Ph₃) (major)), **76.6** (*C*(3```)OH (minor)), **77.7** (*C*(3```)OH (major)), **96.8** (C=*C*H₂ (minor)), **97.0** (C=*C*H₂ (major)), **114.2** (Ar*C*(7)H (minor)), **114.3** (Ar*C*(7)H (major)), **116.7** (Ar*C*(7```)H (minor)), **116.9** (Ar*C*(7```)H (major)), **123.2** (Ar*C*(5```)H (major)), **124.2** (Ar*C*(4```)H (minor)), **124.4** (Ar*C*(4```)H (major)), **126.6** (Ar*C*(5)CH₃ (minor)), **126.9** (Ar*C*(4)H (major)), **127.0** (Ar*C*(5)CH₃ (major)), **127.1** (Ar*C*(4) (minor)), **127.3** (Ar*C*(4``)H (minor)), **127.4** (Ar*C*(4``)H (major)), **127.8** (Ar*C*(2`,6`)H (major and minor)), **128.2** (Ar*C*(3``,5``)H (major and minor)), **128.9** (Ar*C*(3a```) (major)), **129.0** (Ar*C*(6```)H (major)), **129.1** (Ar*C*(6```)H (minor)), **129.6** (Ar*C*H (major)), **129.8** (Ar*C*(2``,6``)H (major and minor)), **130.1** (Ar*C*(3`,5`)H (major)), **130.3** (Ar*C*H), **130.5** (Ar*C*H (minor)), **133.7** (Ar*C*(3a) (minor)), **134.0** (Ar*C*(3a) (major)), **134.8** (Ar*C*(1`) (major)), **135.3** (Ar*C*(1`) (minor)), **141.0** (Ar*C*(7a) (major)), **141.9** (Ar*C*(7a) (minor)), **142.4** (Ar*C*(1``) (major and minor)), **144.2** (Ar*C*(2) (major and minor)), **144.4** (Ar*C*(7a```) (minor)), **144.5** (*C*(7a```) (major)), **145.3** (*C*(4`) (minor)), **145.6**

(Ar*C*(4`) (major)), **177.9** ((*C*=O) (major)), **178.1** ((*C*=O) (minor)); **HRMS (ESI$^+$)** $C_{44}H_{36}N_2O_4SNa$ $[M+Na]^+$ found 711.2267, requires 711.2288 (–2.95 ppm).

(3*R*,3'*S*)-3'-Hydroxy-6-methyl-2-methylene-1-tosyl-1'-trityl-[3,3'-biindolin]-2'-one (16)

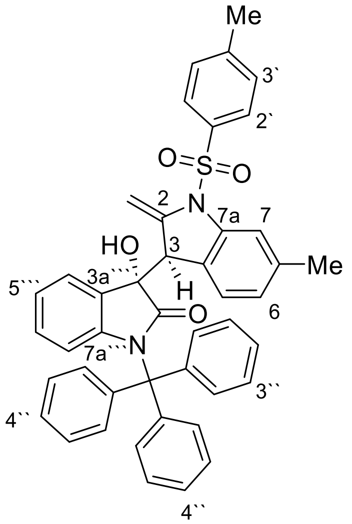

Following **General procedure E**, 3-[(6-methyl-1-tosyl-1*H*-indol-2-yl)methoxy]-1-tritylindolin-2-one (68.8 mg, 0.1 mmol) and *t*Bu-BIMP 3.7 mg, 0.005 mmol) in 1,4-dioxane (2.0 mL, 0.05 M) at 30 °C for 7 h gave a crude [2,3]-rearrangement product which was purified by flash column chromatography (eluent: hexane/acetone = 4:1 to 3:1) to afford the product (50.2 mg, 73%) as a colourless amorphous solid. **IR** ν_{max} (film) 1724 (C=O), 1608 (C=C), 1597, 1465, 1358 (S=O), 1170 (S=O), 1107, 1087; $[\alpha]_D^{20}$ = –49.2 (*c* 0.25 in $CHCl_3$); **Chiral HPLC analysis**, Chiralpak IA (85:15 hexane:^{i}PrOH, flow rate 1 ml·min^{-1}, 211 nm, 30 °C) major diastereomer: tR (3*S*,3'*R*)-**16**: 16.9 min, tR (3*R*,3'*S*)-**16**: 28.6 min, 2:98 er; minor diastereomer tR (3*S*,3'*S*)-**16**: 34.3 min, tR (3*R*,3'*R*)-**16**: 61.1 min, 98:2 er; **^{1}H NMR (500 MHz, CD_2Cl_2)** δ **2.39** (4.54H, s, ArC(4`)C*H*$_3$ and ArC(6)C*H*$_3$ (major)), **2.43** (4.54H, s, ArC(4`)C*H*$_3$ and ArC(6)C*H*$_3$ (minor)), **2.98** (0.76H, s, O*H* (major)), **3.36** (0.23H, s, O*H* (minor)), **4.18** (1H, s, C(3)*H* (major and minor)), **4.32** (0.75H, s, C=C*H*^{A}H^B (major)), **4.85** (0.24H, s, C=C*H*^{A}H^B (minor)), **5.50** (0.25H, s, C=CH^A*H*^{B} (minor)), **5.61** (0.75H, s, C=CH^A*H*^{B} (major)), **6.24** (0.26H, d, *J* 9.8, ArC(7```)*H* (minor)), **6.28** (0.74H, d, *J* 8.1, ArC(7```)*H* (major)), **6.77–7.01** (3H, m, ArC(5```,6```,4)*H* (major and minor)), **7.08–7.36** (13H, m, ArC(4```,3``,4``,5``,5,3`,5`)*H* (major and minor)), **7.34–7.54** (5.84H, m, ArC(2``,6``)*H* (major and minor)), **7.59** (0.25H, s, ArC(7)*H* (minor)), **7.66** (0.76H, s, ArC(7)*H* (major)), **7.70–7.80** (2H, m, ArC(2`,6`)*H* (major and minor)); **^{13}C{^{1}H} NMR (126 MHz, CD_2Cl_2)** δ_C: **21.9** (ArC(4`)*C*H$_3$ (major and minor)), **22.3** (ArC(6)*C*H$_3$ (major)), **22.4** (ArC(6)*C*H$_3$ (minor)), **53.3** (*C*(3)H (major)), **75.4** (N*C*Ph$_3$ (minor)), **75.6** (N*C*Ph$_3$ (major)), **76.7** (*C*(3```)OH (major)), **77.5** (*C*(3```)OH (major)), **96.9** (C=*C*H$_2$ (minor)), **97.1** (C=*C*H$_2$ (major)), **115.0** (Ar*C*(7)H (minor)), **115.1** (Ar*C*(7)H (major)), **116.7** (Ar*C*(7```)H (major)), **116.8** (Ar*C*(7```)H (minor)), **123.2** (Ar*C*(5```)H (major and minor)), **123.6** (Ar*C*(3a) (minor)), **124.0** (Ar*C*(3a) (major)), **124.2** (Ar*C*(4```)H (minor)), **124.3** (Ar*C*(4```)H (major)), **124.7** (Ar*C*(4)H (minor)), **125.1** (Ar*C*(4)H (major)), **125.9** (Ar*C*(5)H (major)), **126.1** (Ar*C*(5)H (minor)), **127.3** (Ar*C*(4``)H (minor)), **127.4** (Ar*C*(4``)H (major)), **127.7** (Ar*C*(2`,6`)H (major and minor)), **128.1** (Ar*C*(3``,5``)H (minor)), **128.2** (Ar*C*(3``,5``)H (major)), **128.9** (Ar*C*(6```)H (major)), **129.0** (Ar*C*(6```)H (minor)), **129.6** (Ar*C*(2``,6``)H (minor)), **129.8** (Ar*C*(2``,6``)H (major)), **130.1** (Ar*C*(3`,5`)H (major)), **134.9** (Ar*C*(1`) (major)), **135.4** (Ar*C*(1`) (minor)), **139.9** (Ar*C*(6)CH$_3$ (major)), **140.5** (Ar*C*(6)CH$_3$ (minor)), **142.3** (Ar*C*(1``) (major)), **143.4** (Ar*C*(7a) (major)), **144.3** (Ar*C*(7a```) (major)), **144.4** (Ar*C*(2`) (major)), **145.4** (Ar*C*(4`) (minor)), **145.6** (Ar*C*(4`) (major)), **177.7** (*C*=O

(major)), **178.1** (*C*=O (minor)); **HRMS (ESI$^+$)** $C_{44}H_{36}N_2O_4SNa$ [M+Na]$^+$ found 711.2267, requires 711.2288 (–2.95 ppm).

(3*S*,3'*S*)-3-Hydroxy-3-(2-methylene-2,3-dihydrobenzofuran-3-yl)-1-tritylindolin-2-one (17)

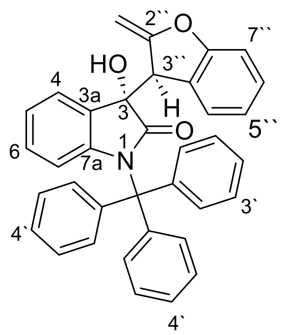

Following **General procedure E**, 3-(benzofuran-2-ylmethoxy)-1-tritylindolin-2-one (52.1 mg, 0.1 mmol) and *t*Bu-BIMP 11.1 mg, 0.015 mmol) in 1,4-dioxane (2.0 mL, 0.05 M) at 30 °C for 48 h gave a crude [2,3]-rearrangement product which was purified by flash column chromatography (eluent: hexane/acetone = 4:1 to 3:1) to afford the product (30.2 mg, 58%) as a colourless amorphous solid. **IR** ν_{max} (film) 1728 (C=O), 1608 (C=C), 1477, 1462, 1319, 1111. **$[\alpha]_D^{20}$**= –4.0 (*c* 0.25 in $CHCl_3$) **Chiral HPLC analysis**, Chiralpak ID (90:10 hexane:^{i}PrOH, flow rate 1 ml·min^{-1}, 211 nm, 30 °C) major diastereomer: tR (3*S*,3'*S*)-**17**: 8.3 min, tR (3*R*,3'*R*)-**17**: 9.0 min, 95:5 er; minor diastereomer tR (3*S*,3'*R*)-**17**: 9.7 min, tR (3*R*,3'*S*)-**17**: 11.6 min, 92:8 er; **^{1}H NMR (400 MHz, $CDCl_3$)** δ **3.03** (0.62H, s, O*H* (major)), **3.41** (0.38H, s, O*H* (minor)), **4.16** (0.62H, m, CCH^AH^B (major)) ,**4.47** (0.38H, m, C(3``)*H* (minor)), **4.48** (0.62H, t, *J* 2.1, C(3``)*H* (major)), **4.56** (0.38H, m, CCH^AH^B (minor)), **4.84–4.92** (1H, m, CCH^AH^B (major and minor)), **6.20** (0.62H, d, *J* 8.1, ArC(7)*H* (major)), **6.23–6.29** (0.38H, m, ArC(7)*H* (minor)), **6.65–6.74** (0.38H, m, ArC*H* (minor)), **6.79–6.91** (2H, m, ArC*H* (major and minor)), **6.89–7.04** (3H, m, ArC*H* (major and minor)), **7.13–7.18** (6H, m, ArC*H* (major and minor)), **7.19–7.25** (6H, m, ArC*H* (major and minor)), **7.32** (0.61H, td, *J* 7.9, 1.4, ArC*H* (minor)), **7.37–7.42** (3.39H, m, ArC*H* (major and minor)), **7.43–7.48** (0.39H, m, ArC*H* (minor)), **7.50** (0.63H, dd, *J* 7.4, 1.5, ArC*H* (major)); **^{13}C{^{1}H} NMR (101 MHz, $CDCl_3$)** δ_C: **51.9** (*C*(3``)H (major)), **52.1** (*C*(3``)H (minor)), **75.1** (N*C*Ph$_3$ (minor)), **75.2** (N*C*Ph$_3$ (major)), **75.7** (*C*(3)OH (minor)), **76.6** (*C*(3)OH (major)), **89.4** (C*C*H$_2$ (major)), **89.6** (C*C*H$_2$ (minor)), **109.4** (Ar*C*H (major)), **109.8** (Ar*C*H (minor)), **116.3** (Ar*C*(7)H (major)), **116.5** (Ar*C*(7)H (minor)), **121.8** (Ar*C*(7``)H (minor)), **122.1** (Ar*C*(7``)H (major)), **122.9** (Ar*C*(5``)H (major)), **123.0** (Ar*C*(5``)H (minor)), **123.6** (Ar*C*H (major)), **123.7** (Ar*C*H (minor)), **124.1** (Ar*C*(3a``) (major)), **124.1** (Ar*C*(3a``) (minor)), **125.5** (Ar*C*H (major)), **125.9** (Ar*C*H (minor)), **127.0** (Ar*C*(4`)H (minor)), **127.1** (Ar*C*(4`)H (major)), **127.7** (Ar*C*(3`,5`)H (minor)), **127.8** (Ar*C*(3`,5`)H (major)), **127.9** (Ar*C*H (major)), **128.1** (Ar*C*H (minor)), **128.1** (Ar*C*(3a) (major)), **128.4** (Ar*C*(3a) (minor)), **128.7** (Ar*C*H (major)), **128.9** (Ar*C*H (minor)), **129.2** (Ar*C*(2`,6`)H (minor)), **129.3** (Ar*C*H (minor)), **129.5** (Ar*C*(2`,6`)H (major)), **130.0** (Ar*C*H (major)), **141.6** (Ar*C*(1`) (minor)), **141.7** (Ar*C*(1`) (major)), **144.0** (Ar*C*(7a) (major)), **144.1** (Ar*C*(7a) (minor)), **158.1** (Ar*C*(major)), **159.0** (Ar*C*(minor)), **159.4** (Ar*C*(7a``) (major)), **159.8** (Ar*C*(7a``) (minor)), **177.4** (*C*=O (major)), **177.6** (*C*=O (minor)); **HRMS (ESI$^+$)** $C_{36}H_{27}NO_3Na$ [M+Na]$^+$ found 544.1878, requires 544.1883 (–0.97 ppm).

(3*R*,3'*S*)-5-Bromo-3'-hydroxy-2-methylene-1-[(4-nitrophenyl)sulfonyl]-1'-trityl-[3,3'-biindolin]-2'-one (18)

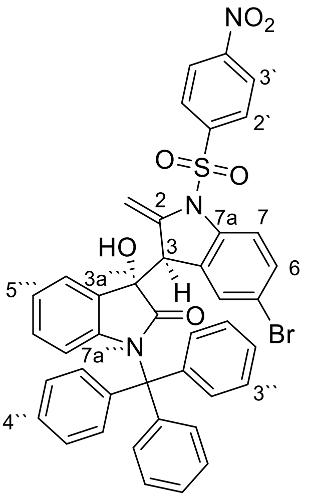

Following **General procedure E**, 3-{[5-bromo-1-((4-nitrophenyl)sulfonyl)-1*H*-indol-2-yl]methoxy}-1-tritylindolin-2-one (78.3 mg, 0.1 mmol) and *t*Bu-BIMP 3.7 mg, 0.005 mmol) in 1,4-dioxane (2.0 mL, 0.05 M) at 30 °C for 2 h gave a crude [2,3]-rearrangement product which was purified by flash column chromatography (eluent: hexane/acetone = 4:1 to 3:1) to afford the product (45.4 mg, 58%) as a yellow amorphous solid. **IR** ν_{max} (film) 1712 (C=O), 1604 (C=C), 1529 (NO_2), 1463, 1346, 1170 (S=O), 1112, 1085; $[\alpha]_D^{20}$ = –36.0 (*c* 0.25 in $CHCl_3$); **Chiral HPLC analysis**, Chiralpak AD-H (85:15 hexane:^{i}PrOH, flow rate 1 ml·min^{-1}, 211 nm, 30 °C) major diastereomer: tR (3*R*,3'*S*)-**18**: 43.3 min, tR (3*S*,3'*R*)-**18**: 50.8 min, 96:4 er; **^{1}H NMR (500 MHz, $CDCl_3$)** δ **2.85** (1H, s, O*H*), **4.17** (1H, s, C(3)*H*), **4.62** (1H, s, C=CH^AH^B), **5.66** (1H, t, *J* 2.1, C=CH^AH^B), **6.24** (1H, d, *J* 8.2, ArC(7```)*H*), **6.88** (1H, td, *J* 7.9, 1.5, ArC(6```)*H*), **6.96** (1H, td, *J* 7.5, 1.0, ArC(5```)*H*), **7.19–7.23** (3H, m, ArC(4``)*H*), **7.24–7.28** (6H, m, ArC(3``,5``)*H*), **7.32–7.37** (6H, m, ArC(2``,6``)*H*), **7.42** (1H, dd, *J* 8.8, 2.1, ArC(6)*H*), **7.48** (1H, dd, *J* 7.4, 1.4, ArC(4```)*H*), **7.52** (1H, d, *J* 2.1, ArC(4)*H*), **7.67** (1H, d, *J* 8.8, ArC(7)*H*), **7.96–8.04** (2H, m, ArC(2`,6`)*H*), **8.28–8.35** (2H, m, ArC(3`,5`)*H*); **$^{13}C\{^1H\}$ NMR (126 MHz, $CDCl_3$)** δ_C **53.1** (*C*(3)H), **75.7** (N*C*Ph_3), **77.3** (*C*(3```)OH), **98.4** (C=*C*H_2), **115.6** (Ar*C*(7)H), **117.0** (Ar*C*(7```)H), **117.9** (Ar*C*(5)Br), **123.2** (Ar*C*(5```)H), **124.1** (Ar*C*(4```)H), **124.5** (Ar*C*(3`,5`)H), **127.1** (Ar*C*(3a```)), **127.3** (Ar*C*(4``)H), **127.9** (Ar*C*(3``,5``)H), **128.6** (Ar*C*(2`,6`)H), **128.7** (Ar*C*(3a)), **129.1** (Ar*C*(6```)H), **129.3** (Ar*C*(4)H), **129.6** (Ar*C*(2``,6``)H), **132.5** (Ar*C*(6)H), **141.2** (Ar*C*(1`)), **141.4** (Ar*C*(1``)), **142.4** (Ar*C*(2)), **142.9** (Ar*C*(7a)), **144.2** (Ar*C*(7a```)), **151.0** (Ar*C*(4`)), **177.4** (*C*=O); **HRMS (ESI$^+$)** $C_{42}H_{30}BrN_3O_6SNa$ [M+Na]$^+$ found 806.0928, requires 806.0931 (–0.36 ppm).

(*S*)-3-Hydroxy-3-[(1-tosyl-1H-indol-2-yl)methyl]-1-tritylindolin-2-one (6)

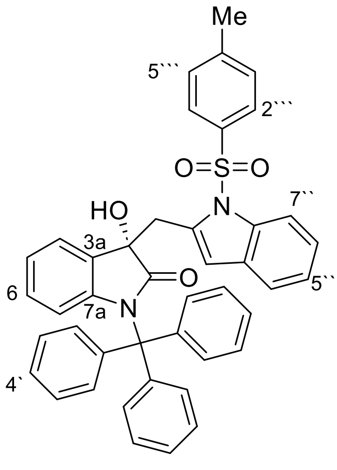

Following **General procedure D**, 3-[(1-tosyl-1*H*-indol-2-yl)methoxy]-1-tritylindolin-2-one (67.4 mg, 0.1 mmol) and *t*Bu-BIMP 11.1 mg, 0.015 mmol) in mesitylene (2.0 mL, 0.05 M) at 30 °C for 24 h gave a crude [1,2]-rearrangement product which was purified by flash column chromatography (eluent: hexane/acetone = 4:1 to 3:1) to afford the product (45.4 mg, 72%) as a colourless amorphous solid. **IR** ν_{max} (film) 1732 (C=O), 1597 (C=C), 1448, 1369 (S=O), 1174 (S=O), 1149 (C-O), 1089; **$[\alpha]_D^{20}$** = –22.4 (*c* 0.25 in $CHCl_3$); **Chiral HPLC analysis**, Chiralpak OD-H (90:10 hexane:^{i}PrOH, flow rate 1 ml·min^{-1}, 211 nm, 30 °C) tR (*R*)-**6**: 13.3 min, tR (*S*)-**6**: 16.6 min, 4:96 er; **^{1}H NMR (500 MHz, $CDCl_3$)** δ **2.30** (3H, s, CH_3), **2.44** (1H, s, O*H*), **3.68** (1H, d, *J* 15.0, CH^AH^B), **4.01** (1H, d, *J* 15.0, C$H^A$$H^B$), **6.27** (1H, dd, *J* 7.0, 1.5, ArC(7)*H*), **6.42** (1H, s, C(3``)*H*), **6.84–6.91** (2H, m, ArC(5,6)*H*), **7.08** (1H, dd, *J* 7.0, 2.0, ArC(4)*H*), **7.12** (2H, d, *J* 8.5, ArC(3```,5```)*H*), **7.17–7.25** (10H, m, ArC(3`, 4`, 5`)*H*, ArC(5``)H), **7.30**, (1H, t, *J* 8.5, ArC(6``)H), **7.36–7.42** (7H, m, ArC(2`, 6`)*H*, ArC(4``)*H*), **7.54** (2H, d, *J* 8.0, ArC(2```,6```)*H*), **8.16** (1H, d, *J* 8.5, ArC(7``)*H*); **$^{13}$C{$^1$H} NMR (126 MHz, $CDCl_3$)** $\delta_C$: **21.7** (*C*$H_3$), **36.9** (*C*$H_2$), **74.7** (N*C*$Ph_3$), **75.8** (*C*(3)), **114.1** (*C*(3``)H), **115.8** (Ar*C*(7``)H), **116.3** (Ar*C*(7)H), **120.7** (Ar*C*(4``)H), **122.5** (Ar*C*(5)H), **124.1** (Ar*C*(5``)H), **124.4** (Ar*C*(4)H), **124.7** (Ar*C*(6``)H), **126.4** (Ar*C*(2```,6```)H), **127.0** (Ar*C*(4`)H), **127.8** (Ar*C*(3`,5`)H), **128.3** (Ar*C*(6)H), **129.4** (Ar*C*(3a)), **129.5** (Ar*C*(2`,6`)H), **129.8** (Ar*C*(3```,5```)H), **129.9** (Ar*C*(3a``)H), **134.8** (Ar*C*(2``)), **135.5** (Ar*C*(1```)), **137.5** (Ar*C*(7a``)), **141.9** (Ar*C*(1`)), **143.1** (Ar*C*(7a)), **144.9** (Ar*C*(4```)), **179.5** (*C*=O); **HRMS (ESI$^+$)** $C_{43}H_{34}N_2O_4SNa$ $[M+Na]^+$ found 697.2128, requires 697.2125 (–0.95 ppm).

(*S*)-4-Chloro-3-hydroxy-3-[(1-tosyl-1*H*-indol-2-yl)methyl]-1-tritylindolin-2-one (19)

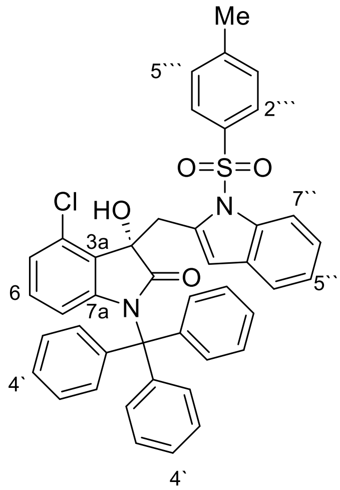

Following **General procedure D**, 4-chloro-3-[(1-tosyl-1*H*-indol-2-yl)methoxy]-1-tritylindolin-2-one (70.8 mg, 0.1 mmol) and *t*Bu-BIMP 11.1 mg, 0.015 mmol) in mesitylene (2.0 mL, 0.05 M) at 30 °C for 24 h gave a crude [1,2]-rearrangement product which was purified by flash column chromatography (eluent: hexane/acetone = 4:1 to 3:1) to afford the product (55.2 mg, 78%) as a colourless amorphous solid. **IR** ν_{max} (film) 1724 (C=O), 1600 (C=C), 1448, 1369 (S=O), 1247, 1147 (S=O), 1085; **$[\alpha]_D^{20}$** = –26.4 (*c* 0.25 in $CHCl_3$); **Chiral HPLC analysis**, Chiralpak OD-H (95:5 hexane:^{i}PrOH, flow rate 1 ml·min^{-1}, 211 nm, 30 °C) tR (*R*)-**19**: 18.4 min, tR (*S*)-**19**: 24.0 min, 7:93 er; **^{1}H NMR (500 MHz, $CDCl_3$)** δ **2.30** (3H, s, CH_3), **3.21** (1H, s, O*H*), **4.19** (1H, q, *J* 14.5, CH_2), **6.15–6.25** (2H, m, ArC(7,3``)*H*), **6.83** (1H, t, *J* 8.2, ArC(6)*H*), **6.92** (1H, d, *J* 8.1, ArC(5)*H*), **7.11–7.17** (11H, m,

ArC(3`,4`,5`,3```,5```)*H*), **7.20** (1H, t, *J* 7.2, ArC(5``)*H*), **7.23–7.34** (8H, m, ArC(2`,6`,4``,6``)*H*), **7.56** (2H, d, *J* 8.4, ArC(2```,6```)*H*), **8.18** (1H, d, *J* 8.3, ArC(7``)*H*); **$^{13}$C{$^{1}$H} NMR (126 MHz, CDCl$_3$)** $\delta_C$: **21.7** (*C*H$_3$), **35.0** (*C*H$_2$), **75.3** (N*C*Ph$_3$), **76.7** (*C*(3)), **114.0** (*C*(3``)H), **115.1** (Ar*C*(7)H), **116.1** (Ar*C*(7``)H), **120.7** (Ar*C*(4``)H), **123.8** (Ar*C*(5)H), **124.1** (Ar*C*(5``)H), **124.8** (Ar*C*(6``)H), **126.5** (Ar*C*(2```,6```)H), **126.5** (Ar*C*(4)Cl), **127.1** (Ar*C*(4`)H), **127.8** (Ar*C*(3`,5`)H), **129.3** (Ar*C*(6)H), **129.4** (Ar*C*(2`,6`)H), **129.8** (Ar*C*(3```,5```)H), **130.2** (Ar*C*(3a``)), **131.0** (Ar*C*(3a)), **134.5** (Ar*C*(2``)), **135.5** (Ar*C*(1```)), **137.8** (Ar*C*(7a``)), **141.5** (Ar*C*(1`)), **145.4** (Ar*C*(7a)), **144.8** (Ar*C*(4```)), **178.1** (*C*=O); **HRMS (ESI$^+$)** $C_{43}H_{32}{}^{35}ClN_2O_4SNa$ [M+Na]$^+$ found 731.1738, requires 731.1742 (–0.52 ppm).

(*S*)-5-Chloro-3-hydroxy-3-[(1-tosyl-1*H*-indol-2-yl)methyl]-1-tritylindolin-2-one (20)

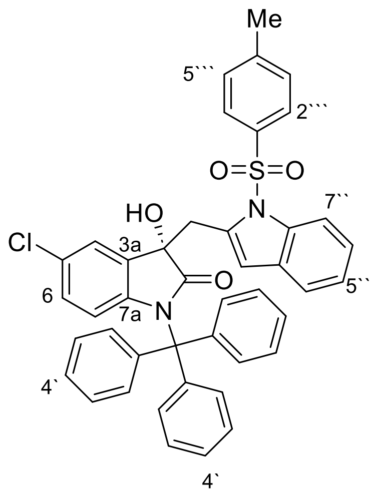

Following **General procedure D**, 5-chloro-3-[(1-tosyl-1*H*-indol-2-yl)methoxy]-1-tritylindolin-2-one (70.8 mg, 0.1 mmol) and *t*Bu-BIMP 13.7 mg, 0.020 mmol) in mesitylene (2.0 mL, 0.05 M) at 40 °C for 48 h gave a crude [1,2]-rearrangement product which was purified by flash column chromatography (eluent: hexane/acetone = 4:1 to 3:1) to afford the product (43.2 mg, 61%) as a colourless amorphous solid. **IR** ν_{max} (film) 1732 (C=O), 1595, 1448, 1367, 1263, 1170 (S=O), 1149, 1089; **$[\alpha]_D^{20}$** = 44.0 (*c* 0.25 in CHCl$_3$); **Chiral HPLC analysis**, Chiralpak IA (80:20 hexane:^{i}PrOH, flow rate 1 ml·min^{-1}, 211 nm, 30 °C) tR (*R*)-**20**: 23.9 min, tR (*S*)-**20**: 37.2 min, 5:95 er; **^{1}H NMR (500 MHz, CDCl$_3$)** δ **2.31** (3H, s, C*H*$_3$), **3.48** (1H, s, O*H*), **3.63** (1H, d, *J* 14.8, C*H*^{A}H^B), **4.00** (1H, d, *J* 14.8, CH^A*H*^{B}), **6.18** (1H, d, *J* 8.7, ArC(7)*H*), **6.44** (1H, s, C(3``)*H*), **6.85** (1H, dd, *J* 14.8, 2.3, ArC(6)*H*), **7.05** (1H, dd, *J* 2.4, ArC(4)*H*), **7.13** (2H, d, *J* 8.1, ArC(3```,5```)*H*), **7.17–7.25** (10H, m, ArC(3`, 4`, 5`)*H*, ArC(5``)H), **7.28–7.32**, (1H, m, ArC(4``)H), **7.32–7.37** (6H, m, ArC(2`, 6`)*H*), **7.40** (1H, d, *J* 7.6, ArC(6``)*H*), **7.54** (2H, d, *J* 8.4, ArC(2```,6```)*H*), **8.16** (1H, d, *J* 8.5, ArC(7``)*H*); **$^{13}$C{$^1$H} NMR (126 MHz, CDCl$_3$)** $\delta_C$: **21.7** (*C*H$_3$), **37.1** (*C*H$_2$), **74.9** (N*C*Ph$_3$), **75.7** (*C*(3)), **114.3** (*C*(3``)H), **115.8** (Ar*C*(7``)H), **117.3** (Ar*C*(7)H), **120.7** (Ar*C*(4``)H), **124.2** (Ar*C*(5``)H), **124.8** (Ar*C*(4)H), **124.9** (Ar*C*(6``)H), **126.4** (Ar*C*(2```,6```)H), **127.2** (Ar*C*(4`)H), **127.9** (Ar*C*(3`,5`)H), **128.1** (Ar*C*(5)Cl), **128.3** (Ar*C*(6)H), **129.4** (Ar*C*(2`,6`)H), **129.7** (Ar*C*(3a``)), **129.9** (Ar*C*(3```,5```)H), **131.1** (Ar*C*(3a)), **134.1** (Ar*C*(2``)), **135.4** (Ar*C*(1```)), **137.6** (Ar*C*(7a``)), **141.5** (Ar*C*(1`)), **141.7** (Ar*C*(7a)), **145.0** (Ar*C*(4```)), **179.0** (*C*=O); **HRMS (ESI$^+$)** $C_{43}H_{32}{}^{35}ClN_2O_4SNa$ [M+Na]$^+$ found 731.1733, requires 731.1742 (–1.20 ppm).

(*S*)-3-Hydroxy-5-nitro-3-[(1-tosyl-1*H*-indol-2-yl)methyl]-1-tritylindolin-2-one (21)

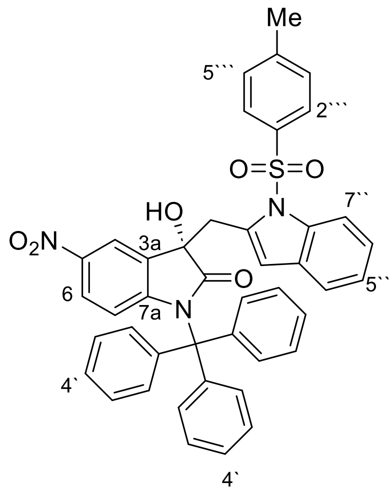

Following **General procedure D**, 5-nitro-3-[(1-tosyl-1*H*-indol-2-yl)methoxy]-1-tritylindolin-2-one (71.9 mg, 0.1 mmol) and *t*Bu-BIMP 11.1 mg, 0.015 mmol) in mesitylene (2.0 mL, 0.05 M) at 30 °C for 24 h gave a crude [1,2]-rearrangement product which was purified by flash column chromatography (eluent: hexane/acetone = 4:1 to 3:1) to afford the product (46.7 mg, 65%) as a colourless amorphous solid. **IR** ν_{max} (film) 1732 (C=O), 1610 (C=C), 1448, 1338 (S=O), 1271, 1172 (S=O), 1149, 1089; $[\alpha]_D^{20}$ = 86.8 (*c* 0.25 in $CHCl_3$); **Chiral HPLC analysis**, Chiralpak OD-H (95:5 hexane:^{i}PrOH, flow rate 1 ml·min^{-1}, 211 nm, 30 °C) tR (*R*)-**21**: 44.7 min, tR (*S*)-**21**: 55.3 min, 3:97 er; ^{1}**H NMR (500 MHz, CDCl$_3$)** δ **2.31** (3H, s, C*H*$_3$), **3.51** (1H, brs, O*H*), **3.71** (1H, d, *J* 14.7, C*H*^{A}H^B), **4.05** (1H, d, *J* 14.7, CH^A*H*^{B}), **6.37** (1H, dd, *J* 7.0, 1.5, ArC(7)*H*), **6.48** (1H, s, C(3``)*H*), **7.13** (2H, d, *J* 8.0, ArC(3```,5```)*H*), **7.18–7.25** (10H, m, ArC(3`,4`,5`,5``)*H*, **7.29–7.36** (7H, m, ArC(2`,6`,6``)*H*), **7.41** (1H, d, *J* 7.7, ArC(4``)*H*)), **7.52** (2H, d, *J* 8.0, ArC(2```,6```)*H*), **7.81** (1H, dd, *J* 9.1, 2.5, ArC(6)H), **7.95** (1H, d, *J* 2.5, ArC(4)*H*), **8.13** (1H, d, *J* 8.4, ArC(7``)*H*); $^{13}$**C{$^1$H} NMR (126 MHz, CDCl$_3$)** δ$_C$: **21.7** (*C*H$_3$), **37.2** (*C*H$_2$), **75.4** (*C*(3)), **75.6** (N*C*Ph$_3$), **114.8** (*C*(3``)H), **115.8** (Ar*C*(7``)H), **115.9** (Ar*C*(7)H), **120.3** (Ar*C*(4)H), **120.9** (Ar*C*(4``)H), **124.4** (Ar*C*(5``)H), **124.7** (Ar*C*(6)H), **125.2** (Ar*C*(5``)H), **126.4** (Ar*C*(2```,6```)H), **127.5** (Ar*C*(4`)H), **128.1** (Ar*C*(3`,5`)H), **129.4** (Ar*C*(2`,6`)H), **129.7** (Ar*C*(3a``)), **129.9** (Ar*C*(3```,5```)H), **130.4** (Ar*C*(3a)), **133.6** (Ar*C*(2``)), **135.4** (Ar*C*(1```)), **137.8** (Ar*C*(7a``)), **141.1** (Ar*C*(1`)), **143.0** (Ar*C*(7a)), **149.0** (Ar*C*(5)NO$_2$), **145.1** (Ar*C*(4```)), **179.5** (*C*=O); **HRMS (ESI$^+$)** $C_{43}H_{33}N_3O_6SNa$ [M+Na]$^+$ found 742.1980, requires 742.1982 (–0.31 ppm).

(*S*)-3-Hydroxy-5-methyl-3-[(1-tosyl-1*H*-indol-2-yl)methyl]-1-tritylindolin-2-one (22)

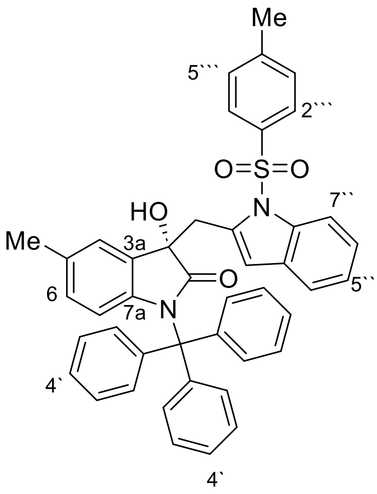

Following **General procedure D**, 5-methyl-3-[(1-tosyl-1*H*-indol-2-yl)methoxy]-1-tritylindolin-2-one (68.8 mg, 0.1 mmol) and *t*Bu-BIMP 13.7 mg, 0.020 mmol) in mesitylene (2.0 mL, 0.05 M) at 40 °C for 24 h gave a crude [1,2]-rearrangement product which was purified by flash column chromatography (eluent: hexane/acetone = 4:1 to 3:1) to afford the product (48.1 mg, 70%) as a colourless amorphous solid. **IR** ν_{max} (film) 1726 (C=O), 1595 (C=C), 1489, 1448, 1365, 1172 (S=O), 1149, 1089; $[\alpha]_D^{20}$ = 33.2 (*c* 0.25 in $CHCl_3$); **Chiral HPLC analysis**, Chiralpak IA (80:20 hexane:^{i}PrOH, flow rate 1 ml·min^{-1}, 211 nm, 30 °C) tR (*R*)-**22**: 25.7 min, tR (*S*)-**22**: 34.7 min, 5:95 er; ^{1}**H NMR (500 MHz, CDCl$_3$)** δ **2.14** (3H, s, ArC(5)C*H*$_3$), **2.30** (3H, s, ArC(4```)C*H*$_3$), **3.34**

(1H, s, O*H*), **3.69** (1H, d, *J* 14.8, C$H^{A}H^{B}$), **3.99** (1H, d, *J* 14.8, C$H^{A}H^{B}$), **6.14** (1H, d, *J* 8.3 ArC(7)*H*), **6.40** (1H, s, C(3``)*H*), **6.63–6.74** (1H, m, ArC(6)*H*), **6.87–6.95** (1H, m, ArC(4)*H*), **7.12** (2H, d, *J* 8.2, ArC(3```,5```)*H*), **7.16–7.24** (10H, m, ArC(3`, 4`, 5`)*H*, ArC(5``)H), **7.29** (1H, td, *J* 8.5, 7.3, 1.4, ArC(6``)*H*), **7.35–7.42** (7H, m, ArC(2`, 6`)*H*, ArC(4``)*H*), **7.54** (2H, d, *J* 8.4, ArC(2```,6```)*H*), **8.08–8.21** (1H, m, ArC(7``)*H*); **^{13}C{^{1}H} NMR (126 MHz, CDCl$_3$)** δ_C: **20.8** (ArC(5)*C*H_3), **21.6** (ArC(4```)*C*H_3), **37.0** (*C*H_2), **74.7** (N*C*Ph$_3$), **75.9** (*C*(3)), **113.9** (*C*(3``)H), **115.7** (Ar*C*(7``)H), **116.1** (Ar*C*(7)H), **120.6** (Ar*C*(4``)H), **124.0** (Ar*C*(5``)H), **124.6** (Ar*C*(6``)H), **125.1** (Ar*C*(4)H), **126.4** (Ar*C*(2```,6```)H), **127.0** (Ar*C*(4`)H), **127.7** (Ar*C*(3`,5`)H), **128.4** (Ar*C*(6)H), **129.4** (Ar*C*(3a)), **129.5** (Ar*C*(2`,6`)H), **129.8** (Ar*C*(3```,5```)H), **129.9** (Ar*C*(3a``)), **132.1** (Ar*C*(5)Me), **134.9** (Ar*C*(2``)), **135.7** (Ar*C*(1```)), **137.6** (Ar*C*(7a``)), **140.7** (Ar*C*(7a)), **142.0** (Ar*C*(1`)), **144.8** (Ar*C*(4```)), **179.4** (*C*=O); **HRMS (ESI$^+$)** $C_{44}H_{36}N_2O_4SNa$ [M+Na]$^+$ found 711.2289, requires 711.2288 (0.11 ppm).

(*S*)-3-Hydroxy-5-methoxy-3-[(1-tosyl-1*H*-indol-2-yl)methyl]-1-tritylindolin-2-one (23)

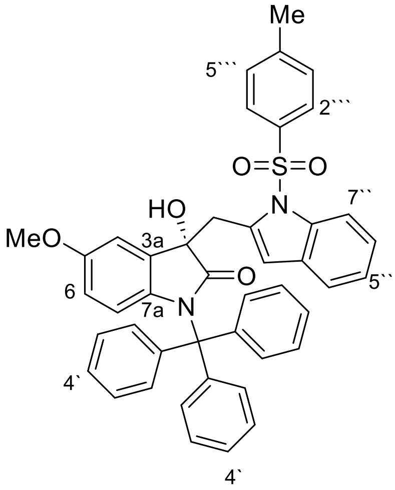

Following **General procedure D**, 5-methoxy-3-[(1-tosyl-1*H*-indol-2-yl)methoxy]-1-tritylindolin-2-one (70.4 mg, 0.1 mmol) and *t*Bu-BIMP 13.7 mg, 0.020 mmol) in mesitylene (2.0 mL, 0.05 M) at 60 °C for 48 h gave a crude [1,2]-rearrangement product which was purified by flash column chromatography (eluent: hexane/acetone = 4:1 to 3:1) to afford the product (31.7 mg, 45%) as a yellow amorphous solid. **IR** ν_{max} (film) 1714 (C=O), 1597 (C=C), 1485, 1448, 1367 (S=O), 1188, 1172 (S=O), 1147, 1089; **$[\alpha]_D^{20}$** = 40.0 (*c* 0.25 in CHCl$_3$); **Chiral HPLC analysis**, Chiralpak IA (80:20 hexane:^{i}PrOH, flow rate 1 ml·min^{-1}, 211 nm, 30 °C) tR (*S*)-**23**: 19.6 min, tR (*R*)-**23**: 49.3 min, 95:5 er; **^{1}H NMR (500 MHz, CDCl$_3$)** δ **2.30** (3H, s, ArC(4```)C*H*$_3$), **3.25–3.48** (1H, m, O*H*), **3.52** (3H, s, OC*H*$_3$), **3.63** (1H, d, *J* 14.7, C$H^{A}H^{B}$), **4.00** (1H, d, *J* 14.8, C$H^{A}H^{B}$), **6.13** (1H, d, *J* 8.9, ArC(7)*H*), **6.42** (1H, dd, *J* 8.9, 2.8, ArC(6)*H*), **6.44** (1H, s, C(3``)*H*), **6.62** (1H, d, *J* 2.8, ArC(4)*H*), **7.12** (2H, d, *J* 8.1, ArC(3```,5```)*H*), **7.16–7.23** (10H, m, ArC(3`, 4`, 5`)*H*, ArC(5``)H), **7.27–7.31** (1H, m, ArC(6``)*H*), **7.33–7.40** (7H, m, ArC(2`, 6`)*H*, ArC(4``)*H*), **7.53** (2H, d, *J* 8.4, ArC(2```,6```)*H*), **8.14** (1H, d, *J* 8.4, ArC(7``)*H*); **^{13}C{^{1}H} NMR (126 MHz, CDCl$_3$)** δ_C: **21.7** (ArC(4```)*C*H_3), **37.0** (*C*H_2), **55.5** (O*C*H_3), **74.7** (N*C*Ph$_3$), **76.1** (*C*(3)), **109.9** (Ar*C*(4)H), **114.3** (*C*(3``)H), **114.6** (Ar*C*(6)H), **115.8** (Ar*C*(7``)H), **117.1** (Ar*C*(7)H), **120.6** (Ar*C*(4``)H), **124.1** (Ar*C*(5``)H), **124.7** (Ar*C*(6``)H), **126.4** (Ar*C*(2```,6```)H), **127.1** (Ar*C*(4`)H), **127.8** (Ar*C*(3`,5`)H), **129.5** (Ar*C*(2`,6`)H), **129.9** (Ar*C*(3```,5```,3a``)H), **130.3** (Ar*C*(3a)), **134.8** (Ar*C*(2``)), **135.5** (Ar*C*(1```)), **136.2** (Ar*C*(7a)), **137.6** (Ar*C*(7a``)), **141.9** (Ar*C*(1`)), **144.9** (Ar*C*(4```)), **155.4** (ArC(5)O*C*H_3), **179.3** (*C*=O); **HRMS (ESI$^+$)** $C_{44}H_{36}N_2O_5SNa$ [M+Na]$^+$ found 727.2216, requires 727.2237 (–2.91 ppm).

(*S*)-6-Chloro-3-hydroxy-3-[(1-tosyl-1*H*-indol-2-yl)methyl]-1-tritylindolin-2-one (24)

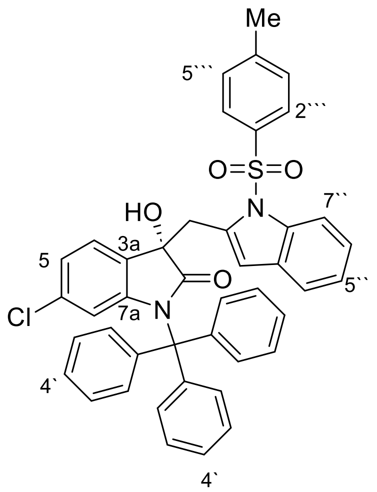

Following **General procedure D**, 6-chloro-3-[(1-tosyl-1*H*-indol-2-yl)methoxy]-1-tritylindolin-2-one (70.8 mg, 0.1 mmol) and *t*Bu-BIMP 11.1 mg, 0.015 mmol) in mesitylene (2.0 mL, 0.05 M) at 30 °C for 24 h gave a crude [1,2]-rearrangement product which was purified by flash column chromatography (eluent: hexane/acetone = 4:1 to 3:1) to afford the product (56.6 mg, 80%) as a colourless amorphous solid. **IR** ν_{max} (film) 1732 (C=O), 1608, 1473, 1448, 1369 (S=O), 1174 (S=O), 1151, 1089; $[\alpha]_D^{20}$ = 19.2 (*c* 0.25 in $CHCl_3$); **Chiral HPLC analysis**, Chiralpak IB (95:5 hexane:^{i}PrOH, flow rate 1 ml·min^{-1}, 211 nm, 30 °C) tR (*R*)-**24**: 25.8 min, tR (*S*)-**24**: 31.9 min, 5:95 er; **^{1}H NMR (500 MHz, $CDCl_3$)** δ **2.31** (3H, s, C*H*$_3$), **3.51** (1H, s, O*H*), **3.57** (1H, d, *J* 14.8, C*H*^{A}H^B), **3.99** (1H, d, *J* 14.8, CH^A*H*^{B}), **6.21** (1H, d, *J* 1.8, ArC(7)*H*), **6.43** (1H, s, C(3``)*H*), **6.82** (1H, dd, *J* 8.0, 1.8, ArC(5)*H*), **6.94** (1H, d, *J* 8.0, ArC(4)*H*), **7.12** (2H, d, *J* 8.2, ArC(3```,5```)*H*), **7.19–7.25** (10H, m, ArC(3`,4`,5`,5``)*H*), **7.29–7.33** (1H, m, ArC(6``)*H*), **7.34–7.38** (6H, m, ArC(2`,6`)*H*), **7.38–7.41** (1H, m, ArC(4``)*H*), **7.52** (2H, d, *J* 8.4, ArC(2```,6```)*H*), **8.15** (1H, d, *J* 8.4, ArC(7``)*H*); **$^{13}$C{$^1$H} NMR (126 MHz, $CDCl_3$)** $\delta_C$: **21.7** (*C*H$_3$), **36.9** (*C*H$_2$), **74.9** (N*C*Ph$_3$), **75.4** (*C*(3)OH), **114.3** (*C*(3``)H), **115.8** (Ar*C*(7``)H), **116.6** (Ar*C*(7)H), **120.7** (Ar*C*(4``)H), **122.5** (Ar*C*(5)H), **124.2** (Ar*C*(5``)H), **124.9** (Ar*C*(6``)H), **125.4** (Ar*C*(4)H), **126.4** (Ar*C*(2```,6```)H), **127.3** (Ar*C*(4`)H), **127.7** (Ar*C*(3a)), **127.9** (Ar*C*(3`,5`)H), **129.4** (Ar*C*(2`,6`)*H*), **129.7** (Ar*C*(3a``)), **129.9** (Ar*C*(3```,5```)H), **134.0** (Ar*C*(6)Cl), **134.3** (Ar*C*(2``)), **135.4** (Ar*C*(1```)), **137.6** (Ar*C*(7a``)), **141.4** (Ar*C*(1`)), **144.3** (Ar*C*(4```)), **145.0** (Ar*C*(7a)), **179.3** (*C*=O); **HRMS (ESI$^+$)** $C_{43}H_{33}{}^{35}ClN_2O_4SNa$ [M+Na]$^+$ found 731.1740, requires 731.1742 (–0.27 ppm).

(*S*)-6-Bromo-3-hydroxy-3-[(1-tosyl-1H-indol-2-yl)methyl]-1-tritylindolin-2-one (25)

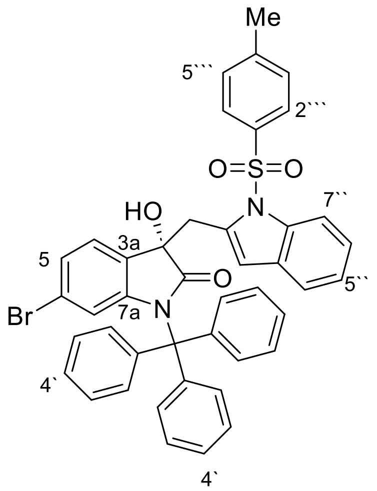

Following **General procedure D**, 6-bromo-3-[(1-tosyl-1*H*-indol-2-yl)methoxy]-1-tritylindolin-2-one (75.2 mg, 0.1 mmol) and *t*Bu-BIMP 11.1 mg, 0.015 mmol) in mesitylene (2.0 mL, 0.05 M) at 30 °C for 24 h gave a crude [1,2]-rearrangement product which was purified by flash column chromatography (eluent: hexane/acetone = 4:1 to 3:1) to afford the product (58.7 mg, 78%) as a colourless amorphous solid. **IR** ν_{max} (film) 1732 (C=O); $[\alpha]_D^{20}$ = 25.2 (*c* 0.25 in $CHCl_3$); **Chiral HPLC analysis**, Chiralpak IB (95:5 hexane:^{i}PrOH, flow rate 1 ml·min^{-1}, 211 nm, 30 °C) tR (*R*)-**25**: 17.2 min, tR (*S*)-**25**: 38.5 min, 6:94 er; **^{1}H NMR (500 MHz, $CDCl_3$)** δ **2.31** (3H, s, C*H*$_3$), **3.51** (1H, s, O*H*), **3.56** (1H, d, *J* 14.8, C*H*^{A}H^B), **3.99** (1H, d, *J* 14.8, CH^A*H*^{B}), **6.35** (1H, d, *J* 1.7, ArC(7)*H*), **6.43** (1H, s, C(3``)*H*), **6.87** (1H, d, *J* 7.9, ArC(4)*H*), **6.98** (1H, d, *J* 8.0, ArC(5)*H*), **7.12** (2H, d, *J* 8.1,

ArC(3```,5```)*H*), **7.19–7.26** (10H, m, ArC(3`,4`,5`,5``)*H*), **7.29–7.33** (1H, m, ArC(6``)*H*), **7.33–7.39** (6H, m, ArC(2`,6`)*H*), **7.40** (1H, d, *J* 7.7, ArC(4``)*H*), **7.52** (2H, d, *J* 8.1, ArC(2```,6```)*H*), **8.15** (1H, d, *J* 8.4, ArC(7``)*H*); **$^{13}$C{$^1$H} NMR (126 MHz, $CDCl_3$)** $\delta_C$: **21.7** ($CH_3$), **36.8** ($CH_2$), **75.0** (N*C*$Ph_3$), **75.5** (*C*(3)OH), **114.3** (*C*(3``)H), **115.8** (Ar*C*(7``)H), **119.4** (Ar*C*(7)H), **120.7** (Ar*C*(4``)H), **122.1** (Ar*C*(6)Br), **124.2** (Ar*C*(5``)H), **124.9** (Ar*C*(6``)H), **125.4** (Ar*C*(5)H), **125.7** (Ar*C*(4)H), **126.4** (Ar*C*(2```,6```)H), **127.3** (Ar*C*(4`)H), **127.9** (Ar*C*(3`,5`)H), **128.3** (Ar*C*(3a)), **129.4** (Ar*C*(2`,6`)*H*), **129.7** (Ar*C*(3a``)), **129.9** (Ar*C*(3```,5```)H), **134.3** (Ar*C*(2``)), **135.4** (Ar*C*(1```)), **137.6** (Ar*C*(7a``)), **141.4** (Ar*C*(1`)), **144.4** (Ar*C*(7a)), **145.0** (Ar*C*(4```)), **179.2** (*C*=O); **HRMS (ESI$^+$)** $C_{43}H_{33}{}^{79}BrN_2O_4SNa$ [M+Na]$^+$ found 775.1231, requires 775.1237 (–0.73 ppm).

(*S*)-3-Hydroxy-3-[(4-methyl-1-tosyl-1*H*-indol-2-yl)methyl]-1-tritylindolin-2-one (26)

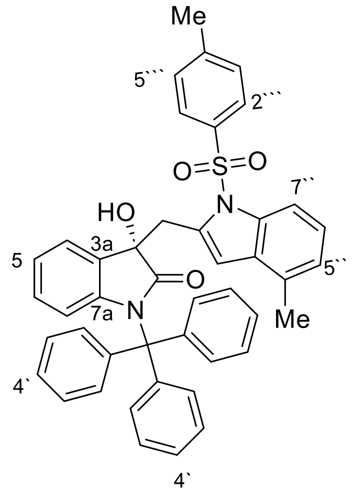

Following **General procedure D**, 3-[(4-methyl-1-tosyl-1*H*-indol-2-yl)methoxy]-1-tritylindolin-2-one (68.8 mg, 0.1 mmol) and *t*Bu-BIMP 14.7 mg, 0.020 mmol) in mesitylene (2.0 mL, 0.05 M) at 40 °C for 24 h gave a crude [1,2]-rearrangement product which was purified by flash column chromatography (eluent: hexane/acetone = 4:1 to 3:1) to afford the product (52.3 mg, 76%) as a colourless amorphous solid. **IR** ν_{max} (film) 1730 (C=O), 1597 (C=C), 1489, 1448, 1367 (S=O), 1178 (S=O), 1149, 1093; **$[\alpha]_D^{20}$** = 6.0 (*c* 0.25 in $CHCl_3$); **Chiral HPLC analysis**, Chiralpak OD-H (90:10 hexane:^{i}PrOH, flow rate 1 ml·min^{-1}, 211 nm, 30 °C) tR (*R*)-**26**: 12.0 min, tR (*S*)-**26**: 15.1 min, 5:95 er; **^{1}H NMR (500 MHz, $CDCl_3$)** δ **2.30** (3H, s, Ar(C4```)C*H*$_3$), **2.34** (3H, s, ArC(4``)C*H*$_3$), **3.40** (1H, s, O*H*), **3.68** (1H, d, *J* 14.6, C$H^A H^B$), **4.02** (1H, d, *J* 14.6, C$H^A H^B$), **6.20–6.29** (1H, m, ArC(7)*H*), **6.49** (1H, s, ArC(3``)*H*), **6.84–6.91** (2H, m, ArC(5,6)*H*), **7.02** (1H, d, *J* 7.3, ArC(5``)*H*), **7.09–7.14** (3H, m, ArC(4,3```,5```)*H*), **7.15–7.23** (10H, m, ArC(3`,4`,5`,6``)*H*), **7.31–7.39** (6H, m, ArC(2`,6`)*H*), **7.54** (2H, d, *J* 8.1, ArC(2```,6```)*H*), **7.97** (1H, d, *J* 8.4, ArC(7``)*H*); **$^{13}$C{$^1$H} NMR (126 MHz, $CDCl_3$)** $\delta_C$ **18.5** (ArC(4``)*C*H$_3$), **21.7** (Ar(C4```)*C*H$_3$), **37.1** (*C*H$_2$), **74.8** (N*C*Ph$_3$), **76.0** (*C*(3)OH), **112.7** (*C*(3``)H), **113.3** (Ar*C*(7``)H), **116.4** (Ar*C*(7)H), **122.4** (Ar*C*(5)H), **124.5** (Ar*C*(5``)H), **124.5** (Ar*C*(4)H), **124.8** (Ar*C*(6``)H), **126.5** (Ar*C*(2```,6```)H), **127.1** (Ar*C*(4`)H), **127.8** (Ar*C*(3`,5`)H), **128.3** (Ar*C*(6)H), **129.4** (Ar*C*(3a)), **129.4** (Ar*C*(3a``)), **129.5** (Ar*C*(2`,6`)H), **129.8** (Ar*C*(3```,5```)H), **130.1** (Ar*C*(4``)Me), **134.0** (Ar*C*(2``)), **135.6** (Ar*C*(1```)), **137.4** (Ar*C*(7a``)), **141.9** (Ar*C*(1`)), **143.2** (Ar*C*(7a)), **144.8** (Ar*C*(4```)), **179.6** (*C*=O); **HRMS (ESI$^+$)** $C_{44}H_{36}N_2O_4SNa$ [M+Na]$^+$ found 711.2277, requires 711.2288 (–1.55 ppm).

(*S*)-3-[(5-Chloro-1-tosyl-1*H*-indol-2-yl)methyl]-3-hydroxy-1-tritylindolin-2-one (27)

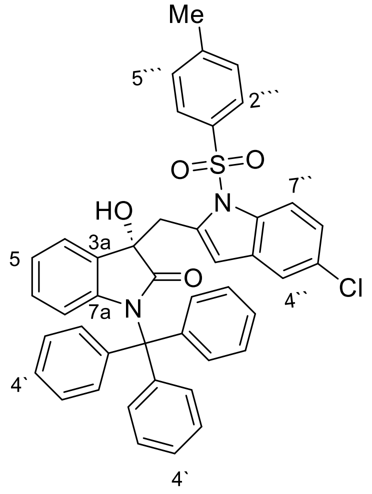

Following **General procedure D**, 3-[(5-chloro-1-tosyl-1*H*-indol-2-yl)methoxy]-1-tritylindolin-2-one (70.8 mg, 0.1 mmol) and *t*Bu-BIMP 14.7 mg, 0.020 mmol) in mesitylene (2.0 mL, 0.05 M) at 40 °C for 24 h gave a crude [1,2]-rearrangement product which was purified by flash column chromatography (eluent: hexane/acetone = 4:1 to 3:1) to afford the product (50.3 mg, 71%) as a colourless amorphous solid. **IR** ν_{max} (film) 1728 (C=O), 1597 (C=C), 1448, 1367 (S=O), 1165 (S=O), 1153, 1089; $[\alpha]_D^{20}$ = –7.6 (*c* 0.25 in $CHCl_3$); **Chiral HPLC analysis**, Chiralpak OD-H (90:10 hexane:^{i}PrOH, flow rate 1 ml·min^{-1}, 211 nm, 30 °C) tR (*R*)-**27**: 13.0 min, tR (*S*)-**27**: 16.7 min, 5:95 er; **^{1}H NMR (500 MHz, CDCl$_3$)** δ **2.32** (3H, s, Ar(C4```)C*H*$_3$), **3.36** (1H, brs, O*H*), **3.67** (1H, d, *J* 14.9, C*H*^{A}H^B), **3.95** (1H, d, *J* 14.9, CH^A*H*^{B}), **6.26–6.30** (1H, m, ArC(7)*H*), **6.32** (1H, s, C(3``)*H*), **6.83–6.92** (2H, m, ArC(5,6)*H*), **7.02–7.06** (1H, m, ArC(4)*H*), **7.14** (2H, d, *J* 8.2, ArC(3```,5```)*H*), **7.18–7.24** (9H, m, ArC(3`,4`,5`)*H*), **7.25** (1H, d, *J* 2.2, ArC(6``)*H*), **7.33** (1H, d, *J* 2.1, ArC(4``)*H*), **7.35–7.42** (6H, m, ArC(2`,6`)*H*), **7.52** (2H, d, *J* 8.4, ArC(2```,6```)*H*), **8.07** (1H, d, *J* 8.9, ArC(7``)*H*); **$^{13}$C{$^1$H} NMR (126 MHz, CDCl$_3$)** δ$_C$: **21.7** (*C*H$_3$), **36.9** (*C*H$_2$), **74.7** (N*C*Ph$_3$), **75.6** (*C*(3)), **113.1** (*C*(3``)H), **116.4** (Ar*C*(7)H), **116.8** (Ar*C*(7``)H), **120.3** (Ar*C*(4``)H), **122.6** (Ar*C*(5)H), **124.2** (Ar*C*(4)H), **124.8** (Ar*C*(6``)H), **126.4** (Ar*C*(2```,6```)H), **127.1** (Ar*C*(4`)H), **127.8** (Ar*C*(3`,5`)H), **128.4** (Ar*C*(6)H), **129.3** (Ar*C*(3a)), **129.5** (Ar*C*(2`,6`)H), **129.8** (Ar*C*(3a``)), **130.0** (Ar*C*(3```,5```)H), **131.1** (*C*(2``)), **135.3** (Ar*C*(1```)), **135.8** (Ar*C*(5``)Cl), **136.4** (Ar*C*(7a``)), **141.8** (Ar*C*(1`)), **143.1** (Ar*C*(7a)), **145.2** (Ar*C*(4```)), **179.3** (*C*=O); **HRMS (ESI$^+$)** $C_{43}H_{33}{}^{35}ClN_2O_4SNa$ [M+Na]$^+$ found 731.1742, requires 731.1742 (0.03 ppm).

(*S*)-3-[(5-Bromo-1-tosyl-1*H*-indol-2-yl)methyl]-3-hydroxy-1-tritylindolin-2-one (28)

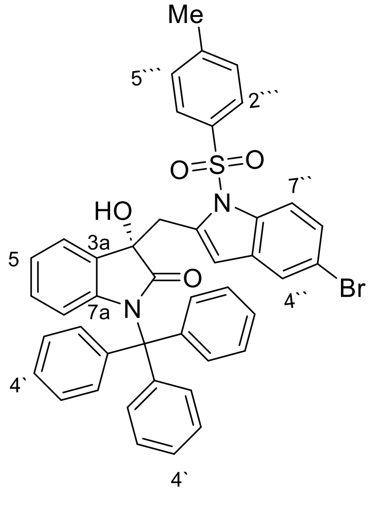

Following **General procedure D**, 3-[(5-bromo-1-tosyl-1*H*-indol-2-yl)methoxy]-1-tritylindolin-2-one (75.2 mg, 0.1 mmol) and *t*Bu-BIMP 14.7 mg, 0.020 mmol) in mesitylene (2.0 mL, 0.05 M) at 40 °C for 24 h gave a crude [1,2]-rearrangement product which was purified by flash column chromatography (eluent: hexane/acetone = 4:1 to 3:1) to afford the product (54.1 mg, 72%) as a colourless amorphous solid. **IR** ν_{max} (film) 1732 (C=O), 1597 (C=C), 1444, 1369 (S=O), 1188, 1166 (S=O), 1089; $[\alpha]_D^{20}$ = –9.6 (*c* 0.25 in $CHCl_3$); Chiralpak IA (80:20 hexane:^{i}PrOH, flow rate 1 ml·min^{-1}, 211 nm, 30 °C) tR (*S*)-**28**: 35.0 min, tR (*R*)-**28**: 44.2 min, 96:4 er; **^{1}H NMR (500 MHz, CDCl$_3$)** δ **2.32** (3H, s, C*H*$_3$), **3.34** (1H, s, O*H*), **3.69** (1H, d, *J* 14.8, C*H*^{A}H^B), **3.94** (1H, d, *J* 14.9, CH^A*H*^{B}), **6.28** (1H, d, *J* 7.6, ArC(7)*H*), **6.30** (1H, s,

C(3``)*H*), **6.83–6.93** (2H, m, ArC(5,6)*H*), **7.02–7.06** (1H, m, ArC(4)*H*), **7.14** (2H, d, *J* 8.2, ArC(3```,5```)*H*), **7.17–7.24** (9H, m, ArC(3`,4`,5`)*H*), **7.36–7.43** (7H, m, ArC(2`,6`,6``)*H*), **7.49** (1H, d, *J* 2.0, ArC(4``)*H*), **7.52** (2H, d, *J* 8.3, ArC(2```,6```)*H*), **8.02** (1H, d, *J* 8.9, ArC(7``)*H*); **$^{13}$C{$^1$H} NMR (126 MHz, $CDCl_3$)** $\delta_C$: **21.7** ($CH_3$), **36.8** ($CH_2$), **74.8** (N*C*$Ph_3$), **75.7** (*C*(3)), **112.9** (*C*(3``)H), **116.5** (Ar*C*(7)H), **117.1** (Ar*C*(7``)H), **117.5** (Ar*C*(5``)Br), **122.6** (Ar*C*(5)H), **123.4** (Ar*C*(4``)H), **124.2** (Ar*C*(4)H), **126.4** (Ar*C*(2```,6```)H), **127.1** (Ar*C*(4`)H), **127.5** (Ar*C*(6``)H), **127.8** (Ar*C*(3`,5`)H), **128.5** (Ar*C*(6)H), **129.3** (Ar*C*(3a)), **129.5** (Ar*C*(2`,6`)H), **130.0** (Ar*C*(3```,5```)H), **131.6** (*C*(2``)), **135.4** (Ar*C*(1```)), **136.2** (Ar*C*(3a``)), **136.3** (Ar*C*(7a``)), **141.9** (Ar*C*(1`)), **143.2** (Ar*C*(7a)), **145.2** (Ar*C*(4```)), **179.3** (*C*=O); **HRMS (ESI$^+$)** $C_{43}H_{33}{}^{79}BrN_2O_4SNa$ [M+Na]$^+$ found 775.1231, requires 775.1237 (–0.73 ppm).

(*S*)-3-Hydroxy-3-[(5-methyl-1-tosyl-1H-indol-2-yl)methyl]-1-tritylindolin-2-one (29)

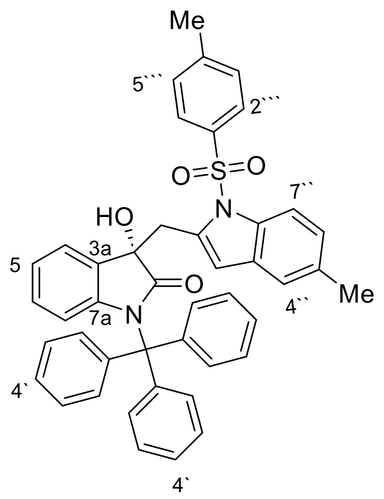

Following **General procedure D**, 3-[(5-methyl-1-tosyl-1*H*-indol-2-yl)methoxy]-1-tritylindolin-2-one (68.8 mg, 0.1 mmol) and *t*Bu-BIMP 14.7 mg, 0.020 mmol) in mesitylene (2.0 mL, 0.05 M) at 40 °C for 24 h gave a crude [1,2]-rearrangement product which was purified by flash column chromatography (eluent: hexane/acetone = 4:1 to 3:1) to afford the product (52.3 mg, 76%) as a colourless amorphous solid. **IR** ν_{max} (film) 1728 (C=O), 1610 (C=C), 1463, 1365 (S=O), 1178, 1157 (S=O), 1089; **$[\alpha]_D^{20}$** = 6.0 (*c* 0.25 in $CHCl_3$); **Chiral HPLC analysis**, Chiralpak OD-H (90:10 hexane:^{i}PrOH, flow rate 1 ml·min^{-1}, 211 nm, 30 °C) tR (*R*)-**29**: 12.0 min, tR (*S*)-**29**: 16.0 min, 6:94 er; **^{1}H NMR (500 MHz, $CDCl_3$)** δ **2.30** (3H, s, Ar(C4```)CH_3), **2.39** (3H, s, ArC(5``)C$H_3$), **3.45** (1H, s, O*H*), **3.62** (1H, d, *J* 14.8, C$H^AH^B$), **3.98** (1H, d, *J* 14.8, C$H^AH^B$), **6.26** (1H, dd, *J* 7.3, 1.7, ArC(7)*H*), **6.34** (1H, s, C(3``)*H*), **6.82–6.92** (2H, m, ArC(5,6)*H*), **7.03–7.07** (1H, m, ArC(4)*H*), **7.08–7.14** (3H, m, ArC(6``,3```,5```)*H*), **7.16** (1H, s, ArC(4``)*H*), **7.17–7.24** (9H, m, ArC(3`,4`,5`)*H*), **7.35–7.42** (6H, m, ArC(2`,6`)*H*), **7.53** (2H, d, *J* 8.4, ArC(2```,6```)*H*), **8.02** (1H, d, *J* 8.5, ArC(7``)*H*); **$^{13}$C{$^1$H} NMR (126 MHz, $CDCl_3$)** $\delta_C$: **21.4** (ArC(5``)*C*H_3), **21.7** (Ar(C4```)*C*H_3), **37.0** (CH_2), **74.7** (N*C*Ph_3), **75.8** (*C*(3)), **114.1** (*C*(3``)H), **115.5** (Ar*C*(7``)H), **116.3** (Ar*C*(7)H), **120.6** (Ar*C*(4``)H), **122.4** (Ar*C*(5)H), **124.4** (Ar*C*(4)H), **126.1** (Ar*C*(6``)H), **126.4** (Ar*C*(2```,6```)H), **127.0** (Ar*C*(4`)H), **127.8** (Ar*C*(3`,5`)H), **128.3** (Ar*C*(6)H), **129.4** (Ar*C*(3a)), **129.5** (Ar*C*(2`,6`)H), **129.8** (Ar*C*(3```,5```)H), **130.1** (Ar*C*(3a``)), **133.6** (Ar*C*(5``)Me), **134.7** (Ar*C*(2``)), **135.5** (Ar*C*(1```)), **135.8** (Ar*C*(7a``)), **141.9** (Ar*C*(1`)), **143.1** (Ar*C*(7a)), **144.8** (Ar*C*(4```)), **179.5** (*C*=O); **HRMS (ESI$^+$)** $C_{44}H_{36}N_2O_4SNa$ [M+Na]$^+$ found 711.2290, requires 711.2288 (0.28 ppm).

(*S*)-3-Hydroxy-3-[(6-methyl-1-tosyl-1*H*-indol-2-yl)methyl]-1-tritylindolin-2-one (30)

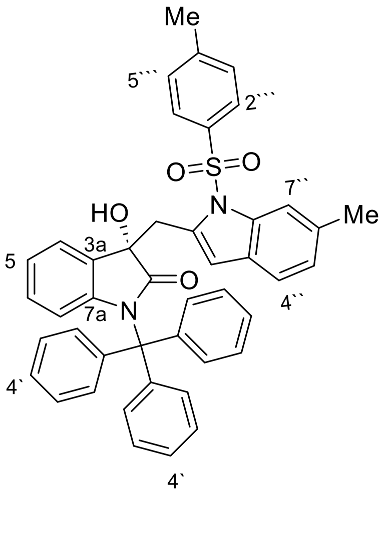

Following **General procedure D**, 3-[(6-methyl-1-tosyl-1*H*-indol-2-yl)methoxy]-1-tritylindolin-2-one (68.8 mg, 0.1 mmol) and *t*Bu-BIMP 14.7 mg, 0.020 mmol) in mesitylene (2.0 mL, 0.05 M) at 40 °C for 24 h gave a crude [1,2]-rearrangement product which was purified by flash column chromatography (eluent: hexane/acetone = 4:1 to 3:1) to afford the product (54.1 mg, 74%) as a colourless amorphous solid. **IR** ν_{max} (film) 1728 (C=O), 1610 (C=C), 1465, 1448, 1263, 1168 (S=O), 1132, 1089; **$[\alpha]_D^{20}$** = 4.0 (*c* 0.25 in $CHCl_3$); **Chiral HPLC analysis**, Chiralpak OD-H (90:10 hexane:^{i}PrOH, flow rate 1 ml·min^{-1}, 211 nm, 30 °C) tR (*R*)-**30**: 12.0 min, tR (*S*)-**30**: 15.2 min, 7:93 er; **^{1}H NMR (500 MHz, $CDCl_3$)** δ **2.31** (3H, s, Ar(C4```)C*H*$_3$), **2.49** (3H, s, ArC(6``)C*H*$_3$), **3.49** (1H, s, O*H*), **3.62** (1H, d, *J* 14.8, C*H*$^A$H$^B$), **3.97** (1H, d, *J* 14.8, CH$^A$*H*$^B$), **6.27** (1H, d, *J* 7.6, ArC(7)*H*), **6.36** (1H, s, ArC(3``)*H*), **6.82–6.92** (2H, m, ArC(5,6)*H*), **7.02–7.08** (2H, m, ArC(4, 5``)*H*), **7.13** (2H, d, *J* 8.2, ArC(3```,5```)*H*), **7.17–7.24** (9H, m, ArC(3`,4`,5`)*H*), **7.25–7.28** (1H, app d, *J* 8.0, ArC(4``)*H*), **7.36–7.42** (6H, m, ArC(2```,6```)*H*), **7.54** (2H, d, *J* 8.2, ArC(2```,6```)*H*), **7.98** (1H, s, ArC(4``)*H*); **^{13}C{^{1}H} NMR (126 MHz, $CDCl_3$)** δ_C **21.7** (Ar(C4```)*C*H$_3$), **22.2** (ArC(6``)*C*H$_3$), **37.0** (*C*H$_2$), **74.6** (N*C*Ph$_3$), **75.8** (*C*(3)OH), **114.1** (*C*(3``)H), **115.9** (Ar*C*(7``)H), **116.3** (Ar*C*(7)H), **120.2** (Ar*C*(4``)H), **122.4** (Ar*C*(5)H), **124.4** (Ar*C*(4)H), **125.5** (Ar*C*(5``)H), **126.4** (Ar*C*(2```,6```)H), **127.0** (Ar*C*(4`)H), **127.6** (Ar*C*(3a``)), **127.8** (Ar*C*(3`,5`)H), **128.3** (Ar*C*(6)H), **129.4** (Ar*C*(3a)), **129.5** (Ar*C*(2`,6`)H), **129.8** (Ar*C*(3```,5```)H), **133.9** (Ar*C*(2``)), **134.7** (Ar*C*(6``)Me), **135.6** (Ar*C*(1```)), **138.0** (Ar*C*(7a``)), **141.9** (Ar*C*(1`)), **143.1** (Ar*C*(7a)), **144.8** (Ar*C*(4```)), **179.6** (*C*=O); **HRMS (ESI$^+$)** $C_{44}H_{36}N_2O_4SNa$ [M+Na]$^+$ found 711.2277, requires 711.2288 (–1.55 ppm).

(*S*)-3-(Benzofuran-2-ylmethyl)-3-hydroxy-1-tritylindolin-2-one (31)

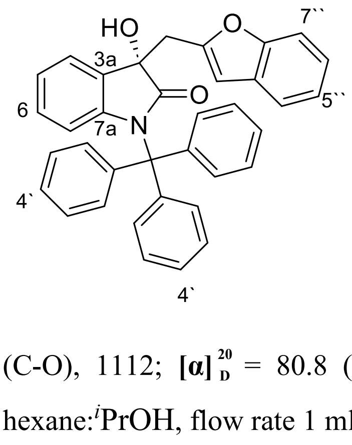

Following **General procedure D**, 3-(benzofuran-2-ylmethoxy)-1-tritylindolin-2-one (52.1 mg, 0.1 mmol) and *t*Bu-BIMP 11.1 mg, 0.015 mmol) in mesitylene (2.0 mL, 0.05 M) at 30 °C for 24 h gave a crude [1,2]-rearrangement product which was purified by flash column chromatography (eluent: hexane/acetone = 4:1 to 3:1) to afford the product (22.4 mg, 43%) as a colourless amorphous solid. **IR** ν_{max} (film) 1712 (C=O), 1610 (C=C), 1481, 1448, 1319, 1263, 1251, 1186 (C-O), 1112; **$[\alpha]_D^{20}$** = 80.8 (*c* 0.25 in $CHCl_3$); **Chiral HPLC analysis**, Chiralpak OD-H (90:10 hexane:^{i}PrOH, flow rate 1 ml·min^{-1}, 211 nm, 30 °C) tR (*R*)-**31**: 9.5 min, tR (*S*)-**31**: 15.8 min, 3.5:96.5 er; **^{1}H NMR (400 MHz, $CDCl_3$)** δ **2.95** (1H, s, O*H*), **3.54** (2H, s, C*H*$_2$), **6.15** (1H, d, *J* 8.2, ArC(7)*H*), **6.28** (1H, s, ArC(3``)*H*), **6.86** (1H, t, *J* 7.9, ArC(6)*H*)), **6.99** (1H, t, *J* 7.5, ArC(5)*H*), **7.03–7.10** (6H, m,

ArC(3`,5`)*H*), **7.11–7.15** (4H, m, ArC(4`)*H*), **7.15–7.20** (6H, m, ArC(2`,6`)*H*), **7.21–7.25** (2H, m, ArC(5``,6``)*H*), **7.32–7.38** (1H, m, ArC(7``)*H*), **7.40** (1H, d, *J* 7.4, ArC(4)*H*), **7.47–7.53** (1H, m, ArC(4``)*H*); **^{13}C{^{1}H} NMR (101 MHz, $CDCl_3$)** δ_C: **39.0** (CH_2), **74.8** (N*C*Ph_3), **75.8** (*C*(3)OH), **105.8** (*C*(3``)H), **111.2** (Ar*C*(7``)H), **116.5** (Ar*C*(7)H), **120.9** (Ar*C*(4``)H), **122.8** (Ar*C*(5)H), **122.9** (Ar*C*(5``)H), **123.4** (Ar*C*(4)H), **124.2** (Ar*C*(6``)H), **127.0** (Ar*C*(4`)H), **127.7** (Ar*C*(3`,5`)H), **128.5** (Ar*C*(6)H), **128.6** (Ar*C*(3a, 3a``)), **129.4** (Ar*C*(2`,6`)H), **141.6** (Ar*C*(1`)), **143.4** (Ar*C*(7a)), **152.3** (*C*(2``)), **154.8** (Ar*C*(7a``)), **179.4** (*C*=O); **HRMS (ESI$^+$)** $C_{36}H_{27}NO_3Na$ $[M+Na]^+$ found 544.1873, requires 544.1883 (–1.78 ppm).

(*S*)-3-Hydroxy-3-[(1-tosyl-1*H*-pyrrol-2-yl)methyl]-1-tritylindolin-2-one (32)

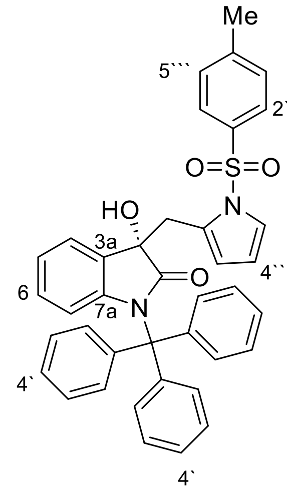

Following **General procedure D**, 3-[(1-tosyl-1*H*-pyrrol-2-yl)methoxy]-1-tritylindolin-2-one (62.4 mg, 0.1 mmol) and *t*Bu-BIMP 11.1 mg, 0.015 mmol) in mesitylene (2.0 mL, 0.05 M) at 30 °C for 24 h gave a crude [1,2]-rearrangement product which was purified by flash column chromatography (eluent: hexane/acetone = 4:1 to 3:1) to afford the product (53.0 mg, 85%) as a colourless amorphous solid. **IR** ν_{max} (film) 1726 (C=O), 1610 (C=C), 1448, 1361 (S=O), 1172 (S=O), 1145, 1089; **$[\alpha]_D^{20}$** = –46.4 (*c* 0.25 in $CHCl_3$); **Chiral HPLC analysis**, Chiralpak OD-H (90:10 hexane:^{i}PrOH, flow rate 1 ml·min^{-1}, 211 nm, 30 °C) tR (*R*)-**32**: 10.7 min, tR (*S*)-**32**: 13.5 min, 9:91 er; **^{1}H NMR (500 MHz, $CDCl_3$)** δ **2.39** (3H, s, CH_3), **3.29–3.41** (2H, m, O*H*, C$H^A$$H^B$), **3.53** (1H, d, *J* 15.1, C$H^A$$H^B$), **5.99** (1H, dd, *J* 3.4, 1.7, ArC(3``)*H*), **6.22** (1H, t, *J* 3.3, ArC(4``)*H*), **6.28** (1H, d, *J* 8.0, ArC(7)*H*), **6.84** (1H, t, *J* 7.5, ArC(5)*H*), **6.88** (1H, td, *J* 7.8, 1.7, ArC(6)*H*), **6.92–6.96** (1H, m, ArC(4)*H*), **7.19–7.23** (4H, m, ArC(4,4`)*H*), **7.30** (1H, dd, *J* 3.4, 1.7, ArC(5``)*H*), **7.23–7.28** (8H, m, ArC(3`,3```,5`,5```)*H*), **7.40–7.45** (6H, m, ArC(2`,6`)*H*), **7.59** (2H, d, *J* 8.3, ArC(2```,6```)*H*); **^{13}C{^{1}H} NMR (126 MHz, $CDCl_3$)** δ_C: **21.8** (CH_3), **35.4** (CH_2), **74.5** (N*C*Ph_3), **75.4** (*C*(3)OH), **112.3** (*C*(4``)H), **116.2** (Ar*C*(7)H), **116.7** (Ar*C*(3``)H), **122.4** (Ar*C*(5)H), **123.7** (Ar*C*(5``)H), **124.2** (Ar*C*(4)H), **126.6** (Ar*C*(2```,6```)H), **127.1** (Ar*C*(4`)H), **127.8** (Ar*C*(3`,5`)H), **128.2** (Ar*C*(6)H), **128.3** (Ar*C*(2``)H), **129.6** (Ar*C*(3a)), **129.5** (Ar*C*(2`,6`)H), **130.1** (Ar*C*(3```,5```)H), **136.5** (Ar*C*(1```)), **142.0** (Ar*C*(1`)), **143.0** (Ar*C*(7a)), **145.1** (Ar*C*(4```)), **179.5** (*C*=O); **HRMS (ESI$^+$)** $C_{39}H_{32}N_2O_4SNa$ $[M+Na]^+$ found 647.1964, requires 647.1975 (–1.70 ppm).

(*S*)-3-Hydroxy-3-[(3-methyl-1-tosyl-1H-indol-2-yl)methyl]-1-tritylindolin-2-one (33)

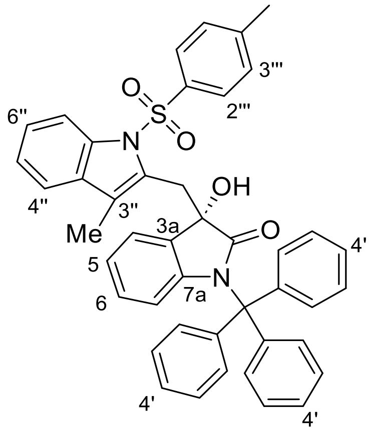

Following **General procedure D**, 3-[(3-methyl-1-tosyl-1*H*-indol-2-yl)methoxy]-1-tritylindolin-2-one (68.8 mg, 0.1 mmol) and *t*Bu-BIMP 11.1 mg, 0.015 mmol) in mesitylene (2.0 mL, 0.05 M) at 30 °C for 24 h gave a crude [1,2]-rearrangement product which was purified by flash column chromatography (eluent: hexane/acetone = 4:1 to 3:1) to afford the product (59.9 mg, 87%) as a colourless amorphous solid. **IR** ν_{max} (film) 1730 (C=O), 1597 (C=C), 1448, 1365, 1186, 1168 (S=O), 1151, 1136; $[\alpha]_D^{20}$ = –4.4 (*c* 0.25 in $CHCl_3$); **Chiral HPLC analysis**, Chiralpak IB (95:5 hexane:^{i}PrOH, flow rate 1 ml·min^{-1}, 211 nm, 30 °C) tR (*S*)-**32**: 20.1 min, tR (*R*)-**33**: 32.3 min, 92:8 er; **^{1}H NMR (500 MHz, $CDCl_3$)** δ **1.67** (3H, s, C*H*$_3$C(3``)Ar), **2.29** (3H, s, C*H*$_3$ArSO$_2$), **3.31** (1H, s, C*H*^{A}H^B), **3.97** (1H, d, *J* 15.1, CH^A*H*^{B}), **4.13** (1H, s, O*H*), **6.28** (1H, d, *J* 8.2, ArC(7)*H*), **6.60** (1H, d, *J* 6.9, ArC(4)*H*), **6.71** (1H, t, *J* 7.4, ArC(5)*H*), **6.85** (1H, td, *J* 7.9, 1.5, ArC(6)*H*), **7.11** (2H, d, *J* 8.1, ArC(3```,5```)*H*), **7.19–7.23** (3H, m, ArC(4`)*H*), **7.24–7.29** (7H, m, ArC(3`,5`,5```)*H*), **7.32–7.36** (2H, m, ArC(4```, 6```)*H*), **7.44–7.48** (6H, m, ArC(2`, 6`)*H*), **7.52** (2H, d, *J* 8.5, ArC(2```,6```)*H*), **8.20–8.26** (1H, m, ArC(7``)*H*); **$^{13}$C{$^1$H} NMR (126 MHz, $CDCl_3$)** $\delta_C$: **9.5** (*C*H$_3$C(3``)Ar), **21.7** (*C*H$_3$ArSO$_2$), **34.8** (*C*H$_2$), **74.7** (N*C*Ph$_3$), **76.5** (*C*(3)), **116.1** (Ar*C*(7``)H), **116.2** (Ar*C*(7)H), **119.0** (Ar*C*(4``)H), **122.3** (Ar*C*(5)H), **122.3** (Ar*C*(3``)), **124.0** (Ar*C*(5``)H), **124.5** (Ar*C*(4)H), **125.1** (Ar*C*(6``)H), **126.5** (Ar*C*(2```,6```)H), **127.1** (Ar*C*(4`)H), **127.8** (Ar*C*(3`,5`)H), **128.0** (Ar*C*(6)H), **129.4** (Ar*C*(3a)), **129.5** (Ar*C*(2`,6`)H), **129.8** (Ar*C*(3```,5```)H), **130.3** (Ar*C*(3a``)), **131.6** (Ar*C*(2``)), **135.1** (Ar*C*(1```)), **137.2** (Ar*C*(7a``)), **142.0** (Ar*C*(1`)), **142.8** (Ar*C*(7a)), **144.8** (Ar*C*(4```)), **179.8** (*C*=O); **HRMS (ESI$^+$)** $C_{44}H_{36}N_2O_4SNa$ [M+Na]$^+$ found 711.2291, requires 711.2288 (0.42 ppm).

(*S*)-3-{[5-bromo-1-((4-nitrophenyl)sulfonyl)-1*H*-indol-2-yl]methyl}-3-hydroxy-1-tritylindolin-2-one (34)

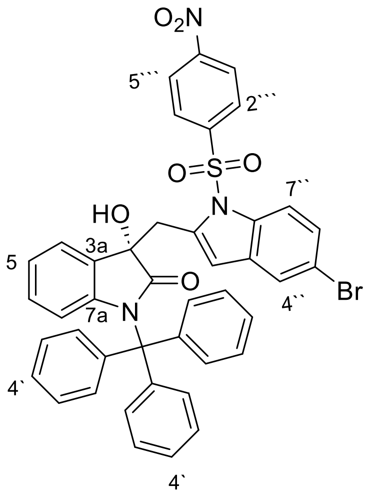

Following **General procedure D**, 3-{[5-bromo-1-((4-nitrophenyl)sulfonyl)-1*H*-indol-2-yl]methoxy}-1-tritylindolin-2-one (78.3 mg, 0.1 mmol) and *t*Bu-BIMP 14.7 mg, 0.020 mmol) in mesitylene (2.0 mL, 0.05 M) at 60 °C for 36 h gave a crude [1,2]-rearrangement product which was purified by flash column chromatography (eluent: hexane/acetone = 4:1 to 3:1) to afford the product (37.6 mg, 48%) as a colourless amorphous solid. **IR** ν_{max} (film) 1726 (C=O), 1606 (C=C), 1531 (NO_2), 1465, 1346, 1178 (S=O), 1087; $[\alpha]_D^{20}$ = 11.2 (*c* 0.25 in $CHCl_3$); **Chiral HPLC analysis**, Chiralpak IA (70:30 hexane:^{i}PrOH, flow rate 2 ml·min^{-1}, 211 nm, 30 °C) tR (*R*)-**34**: 14.7 min, tR (*S*)-**34**: 29.5 min, 4:96 er; **^{1}H NMR (400 MHz, $CDCl_3$)** δ **3.17** (1H, s,

O*H*), **3.70** (1H, d, *J* 15.0, C$H^A$$H^B$), **3.91** (1H, d, *J* 15.0, C$H^A$$H^B$), **6.30** (1H, d, *J* 7.7, ArC(7)*H*), **6.34** (1H, s, ArC(3``)*H*), **6.83–6.96** (2H, m, ArC(5,6)*H*), **7.06** (1H, dd, *J* 7.2, 1.7, ArC(4)*H*), **7.16–7.26** (9H, m, ArC(3`,4`,5`)*H*), **7.32–7.39** (6H, m, ArC(2`,6`)*H*), **7.41** (1H, dd, *J* 8.9, 2.0, ArC(6``)*H*), **7.49** (1H, d, *J* 2.0, ArC(4``)*H*), **7.74–7.82** (2H, m, ArC(2```,6```)*H*), **7.96** (1H, d, *J* 8.9, ArC(7``)*H*), **8.11–8.19** (2H, m, ArC(3```,5```)*H*); **^{13}C{^{1}H} NMR (101 MHz, CDCl$_3$)** δ_C **36.8** (*C*H$_2$), **74.9** (N*C*Ph$_3$), **75.5** (*C*(3)OH), **114.0** (*C*(3``)H), **116.7** (Ar*C*(7)H), **116.9** (Ar*C*(7``)H), **118.4** (Ar*C*(3a``)), **122.7** (Ar*C*(5)H), **123.9** (Ar*C*(4``)H), **124.1** (Ar*C*(4)H), **124.6** (Ar*C*(3```,5```)H), **127.2** (Ar*C*(4`)H), **127.8** (Ar*C*(2```,6```)H), **127.9** (Ar*C*(3`,5`)H), **128.2** (Ar*C*(6``)H), **128.7** (Ar*C*(6)H), **129.0** (Ar*C*(3a)), **129.4** (Ar*C*(2`,6`)H), **131.7** (Ar*C*(5``)Br), **135.9** (Ar*C*(7a``)), **136.2** (Ar*C*(2``)), **141.7** (Ar*C*(1`)), **143.1** (Ar*C*(1```)), **143.2** (Ar*C*(7a)), **150.7** (Ar*C*(4```)), **179.9** (*C*=O); **HRMS (ESI$^+$)** $C_{42}H_{30}BrN_3O_6SNa$ [M+Na]$^+$ found 806.0925, requires 806.0931 (–0.73 ppm).

(*S*)-3-Hydroxy-3-{[1-(methylsulfonyl)-1*H*-indol-2-yl]methyl}-1-tritylindolin-2-one (35)

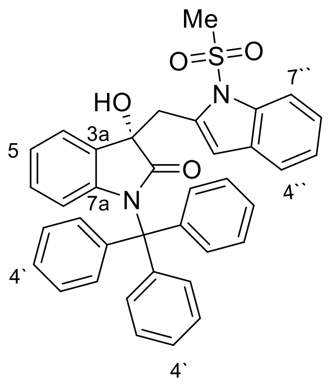

Following **General procedure D**, 3-[(1-(methylsulfonyl)-1*H*-indol-2-yl)methoxy]-1-tritylindolin-2-one (59.8 mg, 0.1 mmol) and *t*Bu-BIMP 14.7 mg, 0.020 mmol) in mesitylene (2.0 mL, 0.05 M) at 30 °C for 20 h gave a crude [1,2]-rearrangement product which was purified by flash column chromatography (eluent: hexane/acetone = 4:1 to 3:1) to afford the product (34.1 mg, 57%) as a colourless amorphous solid. **IR** ν_{max} (film) 1716 (C=O), 1608 (C=C), 1448, 1359, 1168 (S=O), 1147; $[\alpha]_D^{20}$ = −20.4 (*c* 0.25 in CHCl$_3$); **Chiral HPLC analysis**, Chiralpak IA (70:30 hexane:^{i}PrOH, flow rate 1 ml·min^{-1}, 211 nm, 30 °C) tR (*S*)-**35**: 11.3 min, tR (*R*)-**35**: 13.1 min, 92:8 er; **^{1}H NMR (500 MHz, CDCl$_3$)** δ **2.89** (3H, s, C*H*$_3$), **3.26** (1H, s, O*H*), **3.72** (1H, d, *J* 15.0, C$H^A$$H^B$), **3.92** (1H, d, *J* 15.0, C$H^A$$H^B$), **6.21–6.27** (1H, m, ArC(7)*H*), **6.43** (1H, s, ArC(3``)*H*), **6.87–6.92** (2H, m, ArC(5,6)*H*), **7.13** (1H, dd, *J* 5.7, 3.2, ArC(4)*H*), **7.17–7.22** (9H, m, ArC(3`,4`,5`)*H*), **7.27–7.33** (2H, m, ArC(5``,6``)*H*), **7.33–7.37** (6H, m, ArC(2`,6`)*H*), **7.50** (1H, d, *J* 7.6, ArC(4``)*H*), **7.97** (1H, d, *J* 8.2, ArC(7``)*H*); **$^{13}$C{$^1$H} NMR (126 MHz, CDCl$_3$)** $\delta_C$ **36.1** (*C*H$_2$), **40.8** (SO$_2$*C*H$_3$), **74.7** (N*C*Ph$_3$), **75.6** (*C*(3)OH), **112.7** (*C*(3``)H), **114.6** (Ar*C*(7``)H), **116.5** (Ar*C*(7)H), **121.0** (Ar*C*(4``)H), **122.6** (Ar*C*(5)H), **124.0** (Ar*C*(5``)H), **124.3** (Ar*C*(4)H), **124.8** (Ar*C*(6``)H), **127.1** (Ar*C*(4`)H), **127.8** (Ar*C*(3`,5`)H), **128.5** (Ar*C*(6)H), **129.4** (Ar*C*(7)H), **129.6** (Ar*C*(2`,6`,3a``)H), **134.7** (Ar*C*(2``)), **136.9** (Ar*C*(7a``)), **141.7** (Ar*C*(1`)), **143.4** (Ar*C*(7a)), **179.4** (*C*=O); **HRMS (ESI$^+$)** $C_{37}H_{30}N_3O_4SNa$ [M+Na]$^+$ found 621.1826, requires 621.1818 (1.29 ppm).

(*R*)-3-Hydroxy-3-(2-methyl-1-tosyl-1*H*-indol-3-yl)-1-tritylindolin-2-one (4)

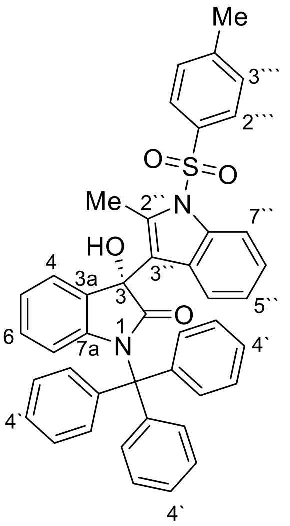

Following **General procedure F**, 3-[(1-tosyl-1*H*-indol-2-yl)methoxy]-1-tritylindolin-2-one (67.4 mg, 0.1 mmol) and *t*Bu-BIMP (3.7 mg, 0.005 mmol) in 1,4-dioxane (2.0 mL, 0.05 M) at 30 °C for 7 h gave a crude [2,3]-rearrangement product, which was treated with TFA (0.10 mmol) for another 6 h and purified by flash column chromatography (eluent: hexane/acetone = 5:1 to 3:1) to afford the product (53.9 mg, 80%) as a colourless amorphous solid. **IR** ν_{max} (film) 1732 (C=O), 1597 (C=C), 1448, 1367 (S=O), 1174 (S=O), 1153, 1089; $[\alpha]_D^{20}$ = –129.2 (*c* 0.25 in $CHCl_3$); **Chiral HPLC analysis**, Chiralpak OD-H (90:10 hexane:^{i}PrOH, flow rate 1 ml·min^{-1}, 211 nm, 30 °C) tR (*S*)-**4**: 7.4 min, tR (*R*)-**4**: 10.4 min, 3:97 er; **IR** ν_{max} (film) 1732 (C=O); **IR** ν_{max} (film) 1732 (C=O); **^{1}H NMR (500 MHz, $CDCl_3$)** δ **2.34** (3H, s, C*H*$_3$ArSO$_2$), **2.40** (3H, s, C*H*$_3$C(2``)NSO$_2$), **3.12** (1H, s, O*H*), **6.33** (1H, d, *J* 8.2, ArC(7)*H*), **6.87** (1H, td, *J* 7.5, 1.0, ArC(5)*H*), **6.93–7.07** (3H, m, ArC(4,6,5``)*H*), **7.16** (1H, dd, *J* 7.4, 1.5, ArC(4``)*H*), **7.17–7.20** (3H, m, ArC(3```,5```,6``)*H*), **7.20–7.26** (9H, m, ArC(3`, 4`, 5`)*H*), **7.45–7.52** (6H, m, ArC(2`, 6`)*H*), **7.56–7.60** (2H, m, ArC(2```,6```)*H*), **8.17** (1H, dd, *J* 8.5, 1.0, ArC(7``)*H*); **$^{13}$C{$^1$H} NMR (126 MHz, $CDCl_3$)** δ$_C$: **14.1** (*C*H$_3$C(2``)NSO$_2$), **21.7** (*C*H$_3$ArSO$_2$), **74.5** (N*C*Ph$_3$), **76.3** (*C*(3)), **114.6** (Ar*C*(7``)H), **116.3** (Ar*C*(7)H), **118.3** (Ar*C*(3``)), **120.5** (Ar*C*(4)H), **123.0** (Ar*C*(5)H), **123.4** (Ar*C*(5``)H), **124.0** (Ar*C*(6``)H), **125.0** (Ar*C*(4``)H), **126.5** (Ar*C*(2```,6```)H), **127.1** (Ar*C*(4`)H), **127.9** (Ar*C*(3`,5`)H), **128.4** (Ar*C*(3a``)), **129.0** (Ar*C*(6)H), **129.2** (Ar*C*(2`,6`)H), **130.0** (Ar*C*(3```,5```)H), **130.9** (Ar*C*(3a)), **135.5** (Ar*C*(2``)), **136.2** (Ar*C*(1```)), **136.5** (Ar*C*(7a``)), **141.8** (Ar*C*(1`)), **143.2** (Ar*C*(7a)), **144.9** (Ar*C*(4```)), **177.0** (*C*=O); **HRMS (ESI$^+$)** $C_{43}H_{34}N_2O_4SNa$ [M+Na]$^+$ found 697.2114, requires 697.2131 (–2.44 ppm).

(*R*)-5-Chloro-3-hydroxy-3-(2-methyl-1-tosyl-1*H*-indol-3-yl)-1-tritylindolin-2-one (36)

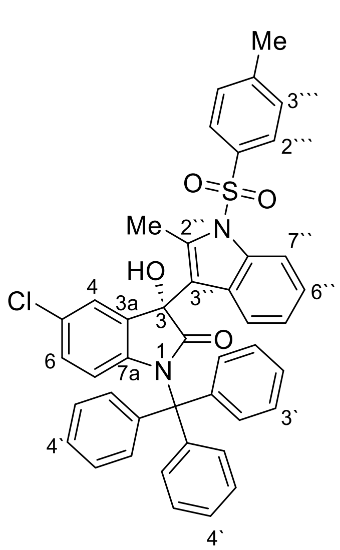

Following **General procedure F**, 5-chloro-3-[(1-tosyl-1*H*-indol-2-yl)methoxy]-1-tritylindolin-2-one (70.8 mg, 0.1 mmol) and *t*Bu-BIMP (3.7 mg, 0.005 mmol) in 1,4-dioxane (2.0 mL, 0.05 M) at 30 °C for 50 min gave a crude [2,3]-rearrangement product, which was treated with TFA (0.10 mmol) for another 24 h and purified by flash column chromatography (eluent: hexane/acetone = 5:1 to 3:1) to afford the product (62.3 mg, 88%) as a colourless amorphous solid. **IR** ν_{max} (film) 1736 (C=O), 1595 (C=C), 1448, 1367, 1174 (S=O), 1118, 1087; $[\alpha]_D^{20}$ = –136.0 (*c* 0.25 in $CHCl_3$); **Chiral HPLC analysis**, Chiralpak OD-H (95:5 hexane:^{i}PrOH, flow rate 1 ml·min^{-1}, 211 nm, 30 °C) tR (*S*)-**36**: 10.1 min, tR (*R*)-**36**: 13.3 min, 2:98 er; **^{1}H NMR (400 MHz, $CDCl_3$)** δ **2.34** (3H, s, ArC(4```)C*H*$_3$), δ **2.37** (3H, s, C*H*$_3$C(2``)NSO$_2$), **3.13** (1H, s, O*H*), **6.24** (1H, d, *J* 8.7,

ArC(7)*H*), **6.93** (1H, dd, *J* 8.7, 2.3, ArC(6)*H*), **6.96–7.10** (3H, m, ArC(4,4``,5``)*H*), **7.16–7.31** (12H, m, ArC(3`,4`,5`,6``,5``,3```,5```)*H*), **7.42–7.50** (6H, m, ArC(2`, 6`)*H*), **7.57** (2H, d, *J* 8.4, ArC(2```,6```)*H*), **8.18** (1H, d, *J* 8.5, ArC(7``)*H*); **$^{13}$C{$^{1}$H} NMR (101 MHz, CDCl$_3$)** $\delta_C$: **14.2** (*C*H$_3$C(2``)NSO$_2$), **21.7** (ArC(4```)*C*H$_3$), **74.6** (N*C*Ph$_3$), **76.2** (*C*(3)), **114.8** (Ar*C*(7``)H), **117.3** (Ar*C*(7)H), **117.8** (Ar*C*(3``)), **120.2** (Ar*C*(4``)H), **123.6** (Ar*C*(5``)H), **124.2** (Ar*C*(6``)H), **125.2** (Ar*C*(4)H), **126.5** (Ar*C*(2```,6```)H), **127.3** (Ar*C*(4`)H), **128.0** (Ar*C*(3`,5`)H), **128.2** (Ar*C*(3a``)), **128.6** (Ar*C*(5``)Cl), **129.0** (Ar*C*(6)H), **129.2** (Ar*C*(2`,6`)H), **130.1** (Ar*C*(3```,5```)H), **132.5** (Ar*C*(3a)), **135.5** (Ar*C*(2``)), **136.2** (Ar*C*(1```)), **136.6** (*C*(7a``)), **141.5** (Ar*C*(1`)), **141.8** (Ar*C*(7a)), **145.1** (Ar*C*(4```)), **176.5** (*C*=O); **HRMS (ESI$^+$)** $C_{43}H_{33}{}^{35}ClN_2O_4SNa$ [M+Na]$^+$ found 731.1761, requires 731.1742 (2.63 ppm).

(*R*)-3-Hydroxy-3-(2-methyl-1-tosyl-1*H*-indol-3-yl)-5-nitro-1-tritylindolin-2-one (37)

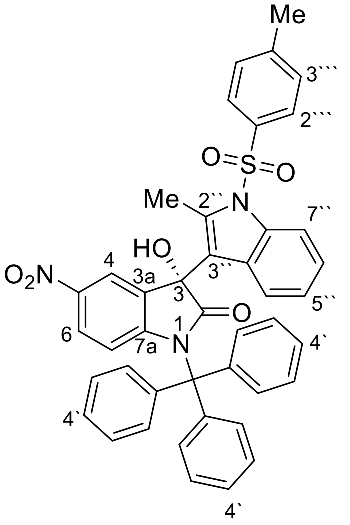

Following **General procedure F**, 5-nitro-3-[(1-tosyl-1*H*-indol-2-yl)methoxy]-1-tritylindolin-2-one (71.9 mg, 0.1 mmol) and *t*Bu-BIMP (3.7 mg, 0.005 mmol) in 1,4-dioxane (2.0 mL, 0.05 M) at 30 °C for 2 min gave a crude [2,3]-rearrangement product, which was treated with TFA (0.20 mmol) for another 24 h and purified by flash column chromatography (eluent: hexane/acetone = 5:1 to 3:1) to afford the product (54.6 mg, 76%) as a colourless amorphous solid. **IR** ν_{max} (film) 1747 (C=O), 1600 (C=C), 1517, 1332 (NO$_2$), 1174 (S=O), 1074; **$[\alpha]_D^{20}$** = –176.0 (*c* 0.25 in CHCl$_3$); **Chiral HPLC analysis**, Chiralpak IA (85:15 hexane:^{i}PrOH, flow rate 1 ml·min^{-1}, 211 nm, 30 °C) tR (*S*)-**37**: 11.7 min, tR (*R*)-**37**: 15.6 min, 5:95 er; **^{1}H NMR (500 MHz, CDCl$_3$)** δ **2.34** (3H, s, ArC(4```)C*H*$_3$), **2.39** (3H, s, C*H*$_3$C(2``)NSO$_2$), **3.25** (1H, s, O*H*), **6.43** (1H, d, *J* 9.0, ArC(7)*H*), **6.85–6.95** (1H, m, ArC(4``)*H*), **6.98** (1H, t, *J* 7.6, ArC(5``)*H*), **7.18–7.21** (2H, m, ArC(3```,5```)*H*), **7.21–7.24** (1H, m, ArC(6``)*H*), **7.26–7.31** (1H, m, ArC(3`,4`,5`)*H*), **7.44–7.50** (6H, m, ArC(2`,6`)*H*), **7.55–7.60** (2H, m, ArC(2```,6```)*H*), **7.90** (1H, dd, *J* 9.0, 2.5, ArC(6)*H*), **7.93** (1H, d, *J* 2.4, ArC(4)*H*), **8.18** (1H, d, *J* 8.5, ArC(7``)*H*); **$^{13}$C{$^{1}$H} NMR (126 MHz, CDCl$_3$)** $\delta_C$: **14.3** (*C*H$_3$C(2``)NSO$_2$), **21.7** (ArC(4```)*C*H$_3$), **75.2** (N*C*Ph$_3$), **75.7** (*C*(3)OH), **115.0** (Ar*C*(7``)H), **115.8** (Ar*C*(7)H), **116.8** (Ar*C*(3``)), **119.8** (Ar*C*(4``)H), **120.6** (Ar*C*(4)H), **123.7** (Ar*C*(5``)H), **124.5** (Ar*C*(6``)H), **125.4** (Ar*C*(6)H), **126.4** (Ar*C*(2```,6```)H), **127.6** (Ar*C*(4`)H), **127.8** (Ar*C*(3a``)), **128.3** (Ar*C*(3`,5`)H), **129.1** (Ar*C*(2`,6`)H), **130.2** (Ar*C*(3```,5```)H), **131.8** (*C*(3a)), **135.8** (*C*(2``)), **136.0** (Ar*C*(1```)), **136.6** (Ar*C*(7a``)), **141.0** (Ar*C*(1`)), **143.3** (Ar*C*(7a)), **145.3** (Ar*C*(4```)), **149.0** (Ar*C*(5)NO$_2$), **176.8** (*C*=O); **HRMS (ESI$^+$)** $C_{43}H_{33}N_3O_6SNa$ [M+Na]$^+$ found 742.1974, requires 742.1982 (–1.12 ppm).

(*R*)-3-Hydroxy-5-methyl-3-(2-methyl-1-tosyl-1*H*-indol-3-yl)-1-tritylindolin-2-one (38)

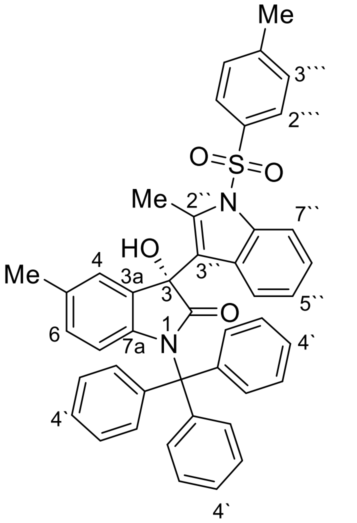

Following **General procedure F**, 5-methyl-3-[(1-tosyl-1*H*-indol-2-yl)methoxy]-1-tritylindolin-2-one (68.8 mg, 0.1 mmol) and *t*Bu-BIMP (3.7 mg, 0.005 mmol) in 1,4-dioxane (2.0 mL, 0.05 M) at 30 °C for 20 h gave a crude [2,3]-rearrangement product, which was treated with TFA (0.10 mmol) for another 4 h and purified by flash column chromatography (eluent: hexane/acetone = 5:1 to 3:1) to afford the product (52.3 mg, 76%) as a colourless amorphous solid. **IR** ν_{max} (film) 1732 (C=O), 1450, 1371 (S=O), 1238, 1174, 1043; $[\alpha]_D^{20}$ = –109.2 (*c* 0.25 in $CHCl_3$); **Chiral HPLC analysis**, Chiralpak OD-H (90:10 hexane:^{i}PrOH, flow rate 1 ml·min^{-1}, 211 nm, 30 °C) tR (*S*)-**38**: 7.0 min, tR (*R*)-**38**: 8.6 min, 4:96 er; **^{1}H NMR (500 MHz, $CDCl_3$)** δ **2.13** (3H, s, ArC(5)C*H*$_3$), **2.34** (3H, s, ArC(4```)C*H*$_3$), **2.40** (3H, s, C*H*$_3$C(2``)NSO$_2$), **3.11** (1H, s, O*H*), **6.20** (1H, d, *J* 8.4, ArC(7)*H*), **6.77** (1H, d, *J* 8.1, ArC(6)*H*), **6.95** (1H, s, ArC(4)*H*), **6.99** (1H, t, *J* 7.5, ArC(5``)*H*), **7.02–7.12** (1H, m, ArC(6``)*H*), **7.16–7.26** (12H, m, ArC(3`,4`,5`,4``,3```,5```)*H*), **7.49** (6H, d, *J* 6.9, ArC(2`,6`)*H*), **7.60** (2H, d, *J* 8.1, ArC(2```,6```)*H*), **8.17** (1H, d, *J* 8.5, ArC(7``)*H*); **$^{13}$C{$^1$H} NMR (126 MHz, $CDCl_3$)** $\delta_C$: **14.1** (*C*H$_3$C(2``)NSO$_2$), **20.8** (ArC(5)*C*H$_3$), **21.7** (ArC(4```)*C*H$_3$), **74.4** (N*C*Ph$_3$), **76.5** (*C*(3)OH), **114.6** (Ar*C*(7``)H), **116.1** (Ar*C*(7)H), **118.4** (Ar*C*(3``)), **120.5** (Ar*C*(6``)H), **123.4** (Ar*C*(5``)H), **124.0** (Ar*C*(4``)H), **125.6** (Ar*C*(4)H), **126.5** (Ar*C*(2```,6```)H), **127.1** (Ar*C*(4`)H), **127.9** (Ar*C*(3`,5`)H), **128.5** (Ar*C*(3a``)), **129.2** (Ar*C*(6)H), **129.5** (Ar*C*(6)H), **130.0** (Ar*C*(3```,5```)H), **130.8** (Ar*C*(5)Me), **132.7** (*C*(3a)), **135.4** (*C*(2``)), **136.2** (Ar*C*(1```)), **136.5** (Ar*C*(7a``)), **140.7** (Ar*C*(7a)), **141.8** (Ar*C*(1`)), **144.9** (Ar*C*(4```)), **177.0** (*C*=O); **HRMS (ESI$^+$)** $C_{44}H_{36}N_2O_4SNa$ [M+Na]$^+$ found 711.2298, requires 711.2288 (1.40 ppm).

(*R*)-3-Hydroxy-5-methoxy-3-(2-methyl-1-tosyl-1*H*-indol-3-yl)-1-tritylindolin-2-one (39)

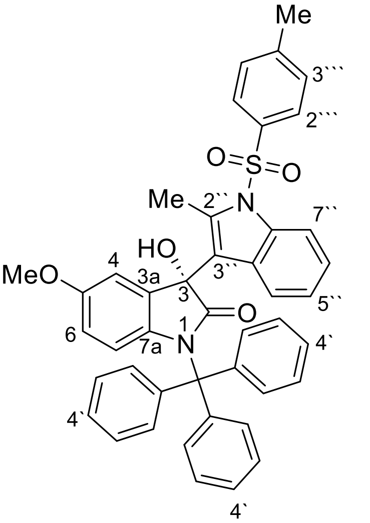

Following **General procedure F**, 5-methoxy-3-[(1-tosyl-1*H*-indol-2-yl)methoxy]-1-tritylindolin-2-one (70.4 mg, 0.1 mmol) and *t*Bu-BIMP (7.4 mg, 0.010 mmol) in 1,4-dioxane (2.0 mL, 0.05 M) at 30 °C for 24 h gave a crude [2,3]-rearrangement product, which was treated with TFA (0.10 mmol) for another 12 h and purified by flash column chromatography (eluent: hexane/acetone = 5:1 to 3:1) to afford the product (42.9 mg, 61%) as a colourless amorphous solid. **IR** ν_{max} (film) 1728 (C=O), 1595 (C=C), 1483, 1436, 1367, 1172 (S=O), 1155, 1087; $[\alpha]_D^{20}$ = –114 (*c* 0.25 in $CHCl_3$); **Chiral HPLC analysis**, Chiralpak AD-H (80:20 hexane:^{i}PrOH, flow rate 1 ml·min^{-1}, 211 nm, 30 °C) tR (*R*)-**39**: 8.7 min, tR (*S*)-**39**: 10.5 min, 98:2 er; **^{1}H NMR (500 MHz, $CDCl_3$)** δ **2.34** (3H, s, ArC(4```)C*H*$_3$), **2.41** (3H, s, C*H*$_3$C(2``)NSO$_2$), **3.60** (1H, s, C*H*$_3$OAr),

6.22 (1H, d, *J* 8.9, ArC(7)*H*), **6.50** (1H, dd, *J* 9.0, 2.8, ArC(6)*H*), **6.73** (1H, d, *J* 2.8, ArC(4)*H*), **6.99** (1H, t, *J* 7.6, ArC(5``)*H*), **7.03–7.13** (1H, m, ArC(4``)*H*), **7.17–7.26** (12H, m, ArC(3`,4`,5`,6``,3```,5```)*H*), **7.44–7.51** (6H, m, ArC(2`,6`)*H*), **7.59** (2H, d, *J* 8.0, ArC(2```,6```)*H*), **8.17** (1H, d, *J* 8.5, ArC(7``)*H*); **$^{13}$C{$^1$H} NMR (126 MHz, $CDCl_3$)** $\delta_C$: **14.1** ($CH_3C$(2``)NSO_2), **21.7** (ArC(4```)CH_3), **55.6** (CH_3OAr), **74.5** (N*C*Ph_3), **77.4** (*C*(3)OH), **111.2** (Ar*C*(4)H), **114.0** (Ar*C*(6)H), **114.6** (Ar*C*(7``)H), **117.0** (Ar*C*(7)H), **118.3** (Ar*C*(3``)), **120.5** (Ar*C*(4``)H), **123.5** (Ar*C*(5``)H), **124.0** (Ar*C*(6``)H), **126.5** (Ar*C*(2```,6```)H), **127.1** (Ar*C*(4`)H), **127.9** (Ar*C*(3`,5`)H), **128.5** (Ar*C*(3a``)), **129.3** (Ar*C*(2`,6`)H), **130.1** (Ar*C*(3```,5```)H), **132.2** (*C*(3a)), **135.5** (*C*(2``)), **136.3** (Ar*C*(7a``)), **136.6** (Ar*C*(7a``,1```)), **141.8** (Ar*C*(1`)), **145.0** (Ar*C*(4```)), **155.9** (Ar*C*(5)OCH_3), **177.0** (*C*=O); **HRMS (ESI$^+$)** $C_{44}H_{36}N_2O_5SNa$ $[M+Na]^+$ found 727.2229, requires 727.2237 (–1.12 ppm).

(*R*)-6-Chloro-3-hydroxy-3-(2-methyl-1-tosyl-1*H*-indol-3-yl)-1-tritylindolin-2-one (40)

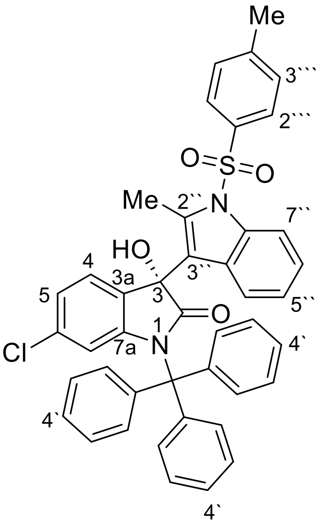

Following **General procedure F**, 6-chloro-3-[(1-tosyl-1*H*-indol-2-yl)methoxy]-1-tritylindolin-2-one (70.8 mg, 0.1 mmol) and *t*Bu-BIMP (3.7 mg, 0.005 mmol) in 1,4-dioxane (2.0 mL, 0.05 M) at 30 °C for 7 h gave a crude [2,3]-rearrangement product, which was treated with TFA (0.10 mmol) for another 12 h and purified by flash column chromatography (eluent: hexane/acetone = 5:1 to 3:1) to afford the product (53.1 mg, 75%) as a colourless amorphous solid. **IR** ν_{max} (film) 1736 (C=O), 1606 (C=C), 1448, 1367, 1263, 1174 (S=O), 1155, 1076; **$[\alpha]_D^{20}$** = –160.0 (*c* 0.25 in $CHCl_3$); **Chiral HPLC analysis**, Chiralpak OD-H (90:10 hexane:^{i}PrOH, flow rate 1 ml·min^{-1}, 211 nm, 30 °C) tR (*S*)-**40**: 7.3 min, tR (*R*)-**40**: 11.1 min, 3:97 er; **^{1}H NMR (400 MHz, $CDCl_3$)** δ **2.25–2.44** (6H, m, CH_3C(2``)NSO_2, ArC(4```)CH_3), **3.13** (1H, s, O*H*), **6.23** (1H, d, *J* 1.8, ArC(7)*H*), **6.85** (1H, dd, *J* 8.0, 1.8, ArC(5)*H*), **6.94–7.09** (3H, m, ArC(4, 4``,5``)*H*), **7.16–7.21** (3H, m, ArC(6``,3```,5```)*H*), **7.21–7.31** (9H, m, ArC(3`,4`,5`)*H*), **7.43–7.51** (6H, m, ArC(2`,6`)*H*), **7.54–7.62** (2H, m, ArC(2`,6`)*H*), **8.16** (1H, d, *J* 8.5, ArC(7``)*H*); **$^{13}$C{$^1$H} NMR (101 MHz, $CDCl_3$)** $\delta_C$: **14.1** ($CH_3C$(2``)NSO_2), **21.7** (ArC(4```)CH_3), **74.7** (N*C*Ph_3), **75.8** (*C*(3)OH), **114.7** (Ar*C*(7``)H), **116.6** (Ar*C*(7)H), **117.8** (Ar*C*(3``)H), **120.3** (Ar*C*(4``)H), **123.0** (Ar*C*(5)H), **123.5** (Ar*C*(5``)H), **124.2** (Ar*C*(6``)H), **125.8** (Ar*C*(4)H), **126.5** (Ar*C*(2```,6```)H), **127.4** (Ar*C*(4`)H), **128.1** (Ar*C*(3`,5`)H), **128.2** (Ar*C*(3a``)), **129.2** (Ar*C*(2`,6`)H), **129.2** (*C*(3a)), **130.1** (Ar*C*(3```,5```)H), **134.7** (Ar*C*(6)Cl), **135.4** (*C*(2``)), **136.2** (Ar*C*(1``)), **136.5** (Ar*C*(7a``)), **141.3** (Ar*C*(1`)), **144.4** (Ar*C*(7a)), **145.0** (Ar*C*(4```)), **176.8** (*C*=O); **HRMS (ESI$^+$)** $C_{43}H_{33}{}^{35}ClN_2O_4SNa$ $[M+Na]^+$ found 731.1771, requires 731.1742 (–3.96 ppm).

(*R*)-6-Bromo-3-hydroxy-3-(2-methyl-1-tosyl-1*H*-indol-3-yl)-1-tritylindolin-2-one (41)

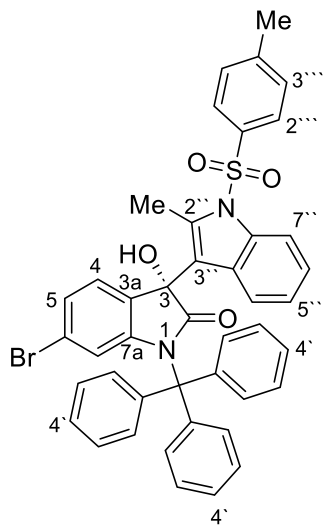

Following **General procedure F**, 6-bromo-3-[(1-tosyl-1*H*-indol-2-yl)methoxy]-1-tritylindolin-2-one (75.2 mg, 0.1 mmol) and *t*Bu-BIMP (3.7 mg, 0.005 mmol) in 1,4-dioxane (2.0 mL, 0.05 M) at 30 °C for 1 h gave a crude [2,3]-rearrangement product, which was treated with TFA (0.10 mmol) for another 12 h and purified by flash column chromatography (eluent: hexane/acetone = 5:1 to 3:1) to afford the product (58.7 mg, 78%) as a colourless amorphous solid. **IR** ν_{max} (film) 1734 (C=O), 1602 (C=C), 1448, 1367, 1174 (S=O), 1155, 1089; $[\alpha]_D^{20}$ = –145.2 (*c* 0.25 in $CHCl_3$); **Chiral HPLC analysis**, Chiralpak IB (95:5 hexane:^{i}PrOH, flow rate 1 ml·min^{-1}, 211 nm, 30 °C) tR (*S*)-**41**: 13.5 min, tR (*R*)-**41**: 15.6 min, 3:97 er; **^{1}H NMR (500 MHz, $CDCl_3$)** δ **2.25–2.42** (6H, m, CH_3C(2``)NSO_2, ArC(4```)CH_3), **3.11** (1H, s, O*H*), **6.36** (1H, s, ArC(7)*H*), **6.96–7.10** (4H, m, ArC(4,5,4``,5``)*H*), **7.14–7.22** (3H, m, ArC(6``,3```,5```)*H*), **7.22–7.31** (9H, m, ArC(3`,4`,5`)*H*), **7.44–7.50** (6H, m, ArC(2`,6`)*H*), **7.58** (2H, d, *J* 8.1, ArC(2`,6`)*H*), **8.16** (1H, d, *J* 8.5, ArC(7``)*H*); **$^{13}$C{$^1$H} NMR (101 MHz, $CDCl_3$)** $\delta_C$: **14.1** (*C*$H_3$C(2``)NSO_2), **21.7** (ArC(4```)*C*H_3), **74.7** (N*C*Ph_3), **75.9** (*C*(3)OH), **114.7** (Ar*C*(7``)H), **117.7** (Ar*C*(3``)H), **117.3** (Ar*C*(7)H), **120.3** (Ar*C*(4``)H), **122.7** (Ar*C*(6)Br), **123.5** (Ar*C*(5``)H), **124.2** (Ar*C*(6``)H), **125.9** (Ar*C*(5)H), **126.1** (Ar*C*(4)H), **126.5** (Ar*C*(2```,6```)H), **127.4** (Ar*C*(4`)H), **128.1** (Ar*C*(3`,5`)H), **128.2** (Ar*C*(3a``)), **129.2** (Ar*C*(2`,6`)H), **129.8** (*C*(3a)), **130.1** (Ar*C*(3```,5```)H), **135.4** (*C*(2``)), **136.2** (Ar*C*(1``)), **136.5** (Ar*C*(7a``)), **141.3** (Ar*C*(1`)), **144.5** (Ar*C*(7a)), **145.0** (Ar*C*(4```)), **176.6** (*C*=O); **HRMS (ESI$^+$)** $C_{43}H_{33}{}^{79}BrN_2O_4SNa$ $[M+Na]^+$ found 775.1232, requires 775.1237 (−0.60 ppm).

(*R*)-3-(5-Chloro-2-methyl-1-tosyl-1*H*-indol-3-yl)-3-hydroxy-1-tritylindolin-2-one (42)

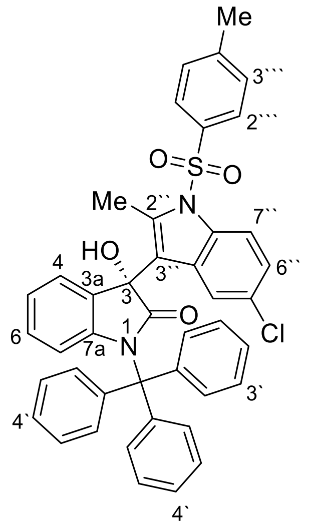

Following **General procedure F**, 3-[(5-chloro-1-tosyl-1*H*-indol-2-yl)methoxy]-1-tritylindolin-2-one (70.8 mg, 0.1 mmol) and *t*Bu-BIMP (3.7 mg, 0.005 mmol) in 1,4-dioxane (2.0 mL, 0.05 M) at 30 °C for 5 h gave a crude [2,3]-rearrangement product, which was treated with TFA (0.10 mmol) for another 12 h and purified by flash column chromatography (eluent: hexane/acetone = 5:1 to 3:1) to afford the product (53.8 mg, 76%) as a colourless amorphous solid. **IR** ν_{max} (film) 1732 (C=O), 1595 (C=C), 1463, 1448, 1367, 1168 (S=O), 1087; $[\alpha]_D^{20}$ = –96.8 (*c* 0.25 in $CHCl_3$) **Chiral HPLC analysis**, Chiralpak OD-H (90:10 hexane:^{i}PrOH, flow rate 1 ml·min^{-1}, 211 nm, 30 °C) tR (*S*)-**42**: 6.6 min, tR (*R*)-**42**: 10.8 min, 3:97 er; **^{1}H NMR (500 MHz, $CDCl_3$)** δ **2.25** (3H, s, CH_3C(2``)NSO_2), **2.36** (3H, m, ArC(4```)CH_3), **3.11** (1H, s, O*H*), **6.35** (1H, d, *J* 8.2, ArC(7)*H*), **6.92** (1H, t, *J* 7.4, ArC(5)*H*), **7.00** (1H, td, *J* 7.9, 1.5, ArC(6)*H*), **7.17** (1H, d, *J* 7.4, 1.5, ArC(4)*H*), **7.19–7.26**

(13H, m, ArC(3`,4`,5`,6``,5``,3```,5```)*H*), **7.38–7.43** (1H, m, ArC(4``)*H*), **7.42–7.48** (6H, m, ArC(2`,6`)*H*), **7.57** (2H, d, *J* 8.4, ArC(2```,6```)*H*), **8.13** (1H, d, *J* 9.0, ArC(7``)*H*); **$^{13}$C{$^1$H} NMR (126 MHz, CDCl$_3$)** $\delta_C$: **14.1** (*C*H$_3$C(2``)NSO$_2$), **21.7** (ArC(4```)*C*H$_3$), **74.7** (N*C*Ph$_3$), **76.2** (*C*(3)), **115.7** (Ar*C*(7``)H), **116.6** (Ar*C*(7)H), **117.8** (Ar*C*(3``)), **120.4** (Ar*C*(4``)H), **123.3** (Ar*C*(5)H), **124.2** (Ar*C*(6``)H), **124.8** (Ar*C*(4)H), **126.5** (Ar*C*(2```,6```)H), **127.2** (Ar*C*(4`)H), **128.0** (Ar*C*(3`,5`)H), **129.2** (Ar*C*(2`,6`)H), **129.2** (Ar*C*(6)H), **129.3** (Ar*C*(3a``)), **130.2** (Ar*C*(3```,5```)H), **130.7** (Ar*C*(3a)), **134.9** (Ar*C*(5``)Cl, Ar*C*(2``)), **135.9** (Ar*C*(1```)), **137.1** (*C*(7a``)), **141.6** (Ar*C*(1`)), **143.2** (Ar*C*(7a)), **145.3** (Ar*C*(4```)), **177.3** (*C*=O); **HRMS (ESI$^+$)** $C_{43}H_{33}{}^{35}ClN_2O_4SNa$ [M+Na]$^+$ found 731.1751, requires 731.1742 (1.26 ppm).

(*R*)-3-(5-Bromo-2-methyl-1-tosyl-1*H*-indol-3-yl)-3-hydroxy-1-tritylindolin-2-one (43)

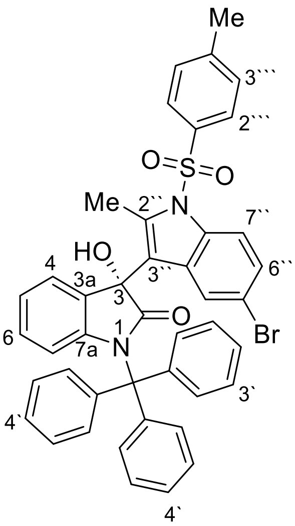

Following **General procedure F**, 3-[(5-bromo-1-tosyl-1*H*-indol-2-yl)methoxy]-1-tritylindolin-2-one (75.2 mg, 0.1 mmol) and *t*Bu-BIMP (3.7 mg, 0.005 mmol) in 1,4-dioxane (2.0 mL, 0.05 M) at 30 °C for 5 h gave a crude [2,3]-rearrangement product, which was treated with TFA (0.10 mmol) for another 7 h and purified by flash column chromatography (eluent: hexane/acetone = 5:1 to 3:1) to afford the product (60.2 mg, 80%) as a colourless amorphous solid. **IR** ν_{max} (film) 1732 (C=O), 1595 (C=C), 1462, 1373, 1263, 1174 (S=O), 1089; **$[\alpha]_D^{20}$** = –92.8 (*c* 0.25 in CHCl$_3$); **Chiral HPLC analysis**, Chiralpak OD-H (90:10 hexane:^{i}PrOH, flow rate 1 ml·min^{-1}, 211 nm, 30 °C) tR (*S*)-**43**: 7.0 min, tR (*R*)-**43**: 12.4 min, 2:98 er; **^{1}H NMR (400 MHz, CDCl$_3$)** δ **2.31** (3H, s, C*H*$_3$C(2``)NSO$_2$), **2.37** (3H, m, ArC(4```)*C*H$_3$), **2.98** (1H, s, O*H*), **6.35** (1H, d, *J* 8.1, ArC(7)*H*), **6.92** (1H, t, *J* 7.4, ArC(5)*H*), **7.00** (1H, td, *J* 7.9, 1.6, ArC(6)*H*), **7.15–7.26** (12H, m, ArC(3`,4`,5`,4,5``,3```,5```)*H*), **7.34** (1H, dd, *J* 9.0, 2.0, ArC(6``)*H*), **7.41–7.47** (6H, m, ArC(2`,6`)*H*), **7.51–7.65** (3H, m, ArC(4``,2```,6```)*H*), **8.09** (1H, d, *J* 9.0, ArC(7``)*H*); **$^{13}$C{$^1$H} NMR (101 MHz, CDCl$_3$)** $\delta_C$: **14.1** (*C*H$_3$C(2``)NSO$_2$), **21.7** (ArC(4```)*C*H$_3$), **74.8** (N*C*Ph$_3$), **76.1** (*C*(3)), **116.1** (Ar*C*(7``)H), **116.6** (Ar*C*(7)H), **117.2** (Ar*C*(5``)Br), **117.7** (Ar*C*(3``)), **123.2** (Ar*C*(5)H), **123.3** (Ar*C*(4``)H), **124.8** (Ar*C*(4)H), **126.5** (Ar*C*(2```,6```)H), **126.9** (Ar*C*(6``)H), **127.2** (Ar*C*(4`)H), **128.0** (Ar*C*(3`,5`)H), **129.2** (Ar*C*(6)H), **129.3** (Ar*C*(2`,6`)H), **130.2** (Ar*C*(3```,5```)H), **130.2** (Ar*C*(3a``)), **130.7** (Ar*C*(3a)), **135.3** (Ar*C*(2``)), **135.9** (Ar*C*(1```)), **137.1** (*C*(7a``)), **141.7** (Ar*C*(1`)), **143.2** (Ar*C*(7a)), **145.3** (Ar*C*(4```)), **177.1** (*C*=O); **HRMS (ESI$^+$)** $C_{43}H_{33}{}^{79}BrN_2O_4SNa$ [M+Na]$^+$ found 775.1218, requires 775.1237 (−2.45 ppm).

(*R*)-3-(2,5-Dimethyl-1-tosyl-1*H*-indol-3-yl)-3-hydroxy-1-tritylindolin-2-one (44)

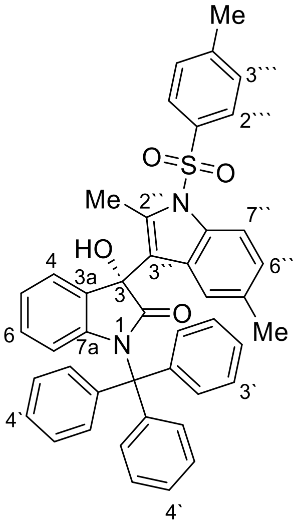

Following **General procedure F**, 3-[(5-methyl-1-tosyl-1*H*-indol-2-yl)methoxy]-1-tritylindolin-2-one (68.8 mg, 0.1 mmol) and *t*Bu-BIMP (7.4 mg, 0.010 mmol) in 1,4-dioxane (2.0 mL, 0.05 M) at 30 °C for 7 h gave a crude [2,3]-rearrangement product, which was treated with TFA (0.10 mmol) for another 12 h and purified by flash column chromatography (eluent: hexane/acetone = 5:1 to 3:1) to afford the product (55.7 mg, 81%) as a colourless amorphous solid. **IR** ν_{max} (film) 1732 (C=O), 1597 (C=C), 1462, 1369, 1238, 1172 (S=O), 1087; $[\alpha]_D^{20}$ = –105.2 (*c* 0.25 in $CHCl_3$); **Chiral HPLC analysis**, Chiralpak OD-H (90:10 hexane:^{i}PrOH flow rate 1 ml·min^{-1}, 211 nm, 30 °C) tR (*S*)-**44**: 7.4 min, tR (*R*)-**44**: 11.6 min, 2:98 er; **^{1}H NMR (500 MHz, $CDCl_3$)** δ **2.30** (3H, s, ArC(5``)*C*H$_3$), **2.32–2.46** (6H, m, ArC(4```)*C*H$_3$, C*H*$_3$C(2``)NSO$_2$), **3.09** (1H, s, O*H*), **6.34** (1H, d, *J* 8.2, ArC(7)*H*), **6.89** (1H, t, *J* 7.5, ArC(5)*H*), **6.97** (1H, td, *J* 7.9, 1.5, ArC(6)*H*), **7.06** (1H, d, *J* 8.6, ArC(6``)*H*), **7.16–7.26** (13H, m, ArC(4,3`,4`,5`,4``,5``,3```,5```)*H*), **7.46** (6H, d, ArC(2`, 6`)*H*), **7.60** (2H, d, *J* 8.1, ArC(2```,6```)*H*), **8.08** (1H, d, *J* 8.6, ArC(7``)*H*); **$^{13}$C{$^1$H} NMR (126 MHz, $CDCl_3$)** δ$_C$: **14.2** (*C*H$_3$C(2``)NSO$_2$), **21.6** (ArC(5``)*C*H$_3$), **21.7** (ArC(4```)*C*H$_3$), **74.6** (N*C*Ph$_3$), **76.3** (*C*(3)), **114.4** (Ar*C*(7``)H), **116.4** (Ar*C*(7)H), **118.2** (Ar*C*(3``)), **120.5** (Ar*C*(4``)H), **123.0** (Ar*C*(5)H), **124.9** (Ar*C*(4)H), **125.4** (Ar*C*(6``)H), **126.5** (Ar*C*(2```,6```)H), **127.2** (Ar*C*(4`)H), **127.9** (Ar*C*(3`,5`)H), **128.7** (Ar*C*(5``)CH$_3$), **128.9** (Ar*C*(6)H), **129.3** (Ar*C*(2`,6`)H), **130.0** (Ar*C*(3```,5```)H), **131.0** (Ar*C*(3a)), **132.9** (Ar*C*(3a``)), **134.8** (*C*(7a``)), **136.2** (Ar*C*(1```)), **141.8** (Ar*C*(1`)), **143.2** (Ar*C*(7a)), **144.8** (Ar*C*(4```)), **177.4** (*C*=O); **HRMS (ESI$^+$)** $C_{44}H_{36}N_2O_4SNa$ [M+Na]$^+$ found 711.2297, requires 711.2288 (–1.26 ppm).

(*R*)-3-(2,6-Dimethyl-1-tosyl-1*H*-indol-3-yl)-3-hydroxy-1-tritylindolin-2-one (45)

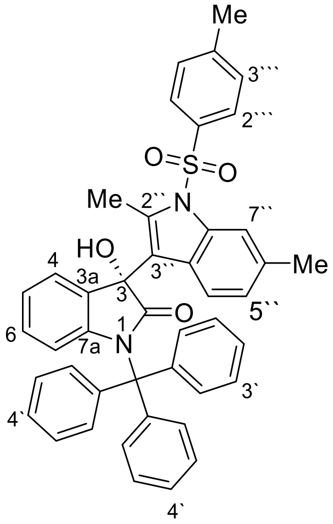

Following **General procedure F**, 3-[(6-methyl-1-tosyl-1*H*-indol-2-yl)methoxy]-1-tritylindolin-2-one (68.8 mg, 0.1 mmol) and *t*Bu-BIMP (7.4 mg, 0.010 mmol) in 1,4-dioxane (2.0 mL, 0.05 M) at 30 °C for 7 h gave a crude [2,3]-rearrangement product, which was treated with TFA (0.10 mmol) for another 12 h and purified by flash column chromatography (eluent: hexane/acetone = 5:1 to 3:1) to afford the product (48.8 mg, 71%) as a colourless amorphous solid. **IR** ν_{max} (film) 1724 (C=O), 1608 (C=C), 1450, 1357, 1170 (S=O), 1107; $[\alpha]_D^{20}$ = –129.2 (*c* 0.25 in $CHCl_3$); **Chiral HPLC analysis**, Chiralpak IA (95:5 hexane:^{i}PrOH, flow rate 1 ml·min^{-1}, 211 nm, 30 °C) tR (*R*)-**45**: 18.1 min, tR (*S*)-**45**: 24.5 min, 98:2 er; **^{1}H NMR (400 MHz, $CDCl_3$)** δ **2.35** (3H, s, ArC(4```)C*H*$_3$), **2.38** (3H, s, C*H*$_3$C(2``)NSO$_2$), **2.42** (3H, s, ArC(6``)C*H*$_3$), **3.05** (1H, s, O*H*),

6.32 (1H, d, *J* 8.1, ArC(7)*H*), **6.78–6.92** (2H, m, ArC(5,4``,5``)*H*), **6.96** (1H, td, *J* 7.9, 1.5, ArC(6)*H*), **7.15** (1H, dd, *J* 7.4, 1.5, ArC(4)*H*), **7.18–7.25** (11H, m, ArC(3`,4`,5`,3```,5```)*H*), **7.43–7.51** (6H, m, ArC(2`,6`)*H*), **7.59** (2H, d, *J* 8.4, ArC(2```,6```)*H*), **8.00** (1H, s, ArC(7``)*H*); **$^{13}$C{$^1$H} NMR (101 MHz, CDCl$_3$)** δ$_C$: **14.1** (*C*H$_3$C(2``)NSO$_2$), **21.7** (ArC(4```)*C*H$_3$), **22.0** (ArC(6``)*C*H$_3$), **74.5** (N*C*Ph$_3$), **76.3** (*C*(3)OH), **114.8** (Ar*C*(7``)H), **116.2** (Ar*C*(7)H), **118.2** (Ar*C*(3``)), **120.0** (Ar*C*(4``)H), **123.0** (Ar*C*(5)H), **124.9** (Ar*C*(5``)H), **125.0** (Ar*C*(4)H), **126.2** (Ar*C*(3a``)), **126.4** (Ar*C*(2```,6```)H), **127.1** (Ar*C*(4`)H), **127.9** (Ar*C*(3`,5`)H), **128.9** (Ar*C*(6)H), **129.3** (Ar*C*(2`,6`)H), **130.0** (Ar*C*(3```,5```)H), **131.0** (Ar*C*(3a)), **134.0** (Ar*C*(6``)CH$_3$), **134.8** (Ar*C*(2``)), **136.4** (Ar*C*(1```)), **137.0** (*C*(7a``)), **141.8** (Ar*C*(1`)), **143.2** (Ar*C*(7a)), **144.8** (Ar*C*(4```)), **177.1** (*C*=O); **HRMS (ESI$^+$)** $C_{44}H_{36}N_2O_4SNa$ [M+Na]$^+$ found 711.2278, requires 711.2288 (–1.41 ppm).

(*R*)-3-Hydroxy-3-(2-methylbenzofuran-3-yl)-1-tritylindolin-2-one (46)

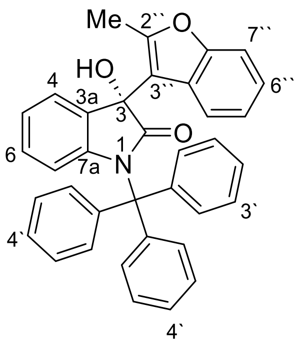

Following **General procedure F**, 3-(benzofuran-2-ylmethoxy)-1-tritylindolin-2-one (52.1 mg, 0.1 mmol) and *t*Bu-BIMP (11.1 mg, 0.015 mmol) in 1,4-dioxane (2.0 mL, 0.05 M) at 30 °C for 48 h gave a crude [2,3]-rearrangement product, which was treated with TFA (0.10 mmol) for another 12 h and purified by flash column chromatography (eluent: hexane/acetone = 5:1 to 3:1) to afford the product (26.0 mg, 50%) as a yellow amorphous solid. **IR** ν$_{max}$ (film) 1714 (C=O), 1597 (C=C), 1450, 1263, 1188; **$[\alpha]_D^{20}$** = –45.3 (*c* 0.25 in CHCl$_3$); **Chiral HPLC analysis**, Chiralpak OD-H (95:5 hexane:^{i}PrOH, flow rate 1 ml·min^{-1}, 211 nm, 30 °C) tR (*R*)-**46**: 7.3 min, tR (*S*)-**46**: 9.1 min, 94:6 er; **^{1}H NMR (500 MHz, CDCl$_3$)** δ **2.41** (3H, s, C*H*$_3$C(2), **3.08** (1H, s, O*H*), **6.37** (1H, d, *J* 8.1, ArC(7)*H*), **6.68** (1H, d, *J* 7.9, ArC(4``)*H*), **6.92–7.00** (1H, m, ArC(5,5``)*H*), **7.01** (1H, td, *J* 7.9, 1.6, ArC(6)*H*), **7.15** (1H, t, *J* 7.7, ArC(5``)*H*), **7.17–7.27** (9H, m, ArC(3`,4`,5`)*H*), **7.33** (1H, d, *J* 7.3, ArC(4)*H*), **7.35** (1H, d, *J* 8.2, ArC(7``)*H*), **7.41–7.48** (6H, m, ArC(2`,6`)*H*); **^{13}C{^{1}H} NMR (126 MHz, CDCl$_3$)** δ$_C$: **14.2** (*C*H$_3$), **74.7** (N*C*Ph$_3$), **75.2** (*C*(3)), **110.8** (Ar*C*(7``)H), **113.4** (Ar*C*(3``)), **116.4** (Ar*C*(7)H), **120.3** (Ar*C*(4``)H), **122.5** (Ar*C*(5``)H), **123.0** (Ar*C*(5)H), **123.5** (Ar*C*(6``)H), **125.0** (Ar*C*(4)H), **127.1** (Ar*C*(4`)H), **127.4** (Ar*C*(3a``)), **127.9** (Ar*C*(3`,5`)H), **129.0** (Ar*C*(6)H), **129.3** (Ar*C*(2`,6`)H), **130.9** (Ar*C*(3a)), **141.8** (Ar*C*(1`)), **143.4** (Ar*C*(7a)), **153.9** (Ar*C*(2``), ArC(7a``)), **177.9** (*C*=O); **HRMS (ESI$^+$)** $C_{36}H_{27}NO_3Na$ [M+Na]$^+$ found 544.1888, requires 544.1883 (0.91 ppm).

(*R*)-3-{5-Bromo-2-methyl-1-[(4-nitrophenyl)sulfonyl]-1*H*-indol-3-yl}-3-hydroxy-1-tritylindolin-2-one (47)

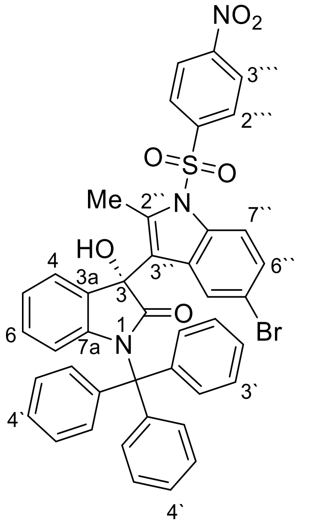

Following **General procedure F**, 3-((5-bromo-1-((4-nitrophenyl)sulfonyl)-1*H*-indol-2-yl)methoxy)-1-tritylindolin-2-one (78.3 mg, 0.1 mmol) and *t*Bu-BIMP (3.7 mg, 0.005 mmol) in 1,4-dioxane (2.0 mL, 0.05 M) at 30 °C for 2 h gave a crude [2,3]-rearrangement product, which was treated with TFA (0.20 mmol) for another 24 h and purified by flash column chromatography (eluent: hexane/acetone = 5:1 to 3:1) to afford the product (56.4 mg, 72%) as a yellow amorphous solid. **IR** ν_{max} (film) 1732 (C=O), 1606 (C=C), 1531 (NO_2), 1448, 1346, 1305, 1178 (S=O), 1087; **$[\alpha]_D^{20}$** = –79.2 (*c* 0.25 in $CHCl_3$); **Chiral HPLC analysis**, Chiralpak AD-H (80:20 hexane:^{i}PrOH, flow rate 1 ml·min^{-1}, 211 nm, 30 °C) tR (*R*)-**47**: 7.4 min, tR (*S*)-**47**: 14.0 min, 96:4 er; **^{1}H NMR (400 MHz, $CDCl_3$)** δ **2.25** (3H, s, C*H*$_3$C(2``)$NSO_2$), **3.08** (1H, s, O*H*), **6.38** (1H, d, *J* 8.1, ArC(7)*H*), **6.94** (1H, td, *J* 7.5, 1.0, ArC(5)*H*), **7.01–7.06** (1H, m, ArC(6)*H*), **7.15** (1H, dd, *J* 7.5, 1.4, ArC(4)*H*), **7.20–7.28** (9H, m, ArC(3`,4`,5`)*H*), **7.39** (1H, dd, *J* 9.0, 2.0, ArC(6``)*H*), **7.45–7.49** (6H, m, ArC(2`,6`)*H*), **7.58–7.72** (1H, m, ArC(4``)*H*), **7.81–7.86** (2H, m, ArC(2```,6```)*H*), **8.04** (1H, d, *J* 9.0, ArC(7``)*H*), **8.24–8.32** (2H, m, ArC(3```,5```)*H*); **^{13}C{^{1}H} NMR (101 MHz, $CDCl_3$)** δ_C: **14.1** (*C*H$_3$C(2``)$NSO_2$), **74.8** (N*C*Ph$_3$), **75.9** (*C*(3)), **116.0** (Ar*C*(7``)H), **116.6** (Ar*C*(7)H), **118.0** (Ar*C*(5``)Br), **119.5** (Ar*C*(3``)), **123.2** (Ar*C*(5)H), **123.8** (Ar*C*(4``)H), **124.6** (Ar*C*(4)H), **124.7** (Ar*C*(3```,5```)H), **127.2** (Ar*C*(4`)H), **127.5** (Ar*C*H), **127.6** (Ar*C*(2```,6```)H), **127.9** (Ar*C*(3`,5`)H), **129.1** (Ar*C*(2`,6`)H), **129.3** (Ar*C*(6)H), **130.3** (Ar*C*(3a)), **130.7** (Ar*C*(3a``)), **135.0** (Ar*C*(2``)), **141.5** (Ar*C*(1`)), **143.2** (Ar*C*(7a)), **143.6** (Ar*C*(1```)), **150.7** (Ar*C*(4```)), **176.6** (*C*=O); **HRMS (ESI$^+$)** $C_{42}H_{30}{}^{79}BrN_3O_6SNa$ [M+Na]$^+$ found 806.0948, requires 806.0931 (2.12 ppm).

(*R*)-1-Benzyl-7-chloro-3-hydroxy-3-(2-methyl-1-tosyl-1*H*-indol-3-yl)indolin-2-one (48)

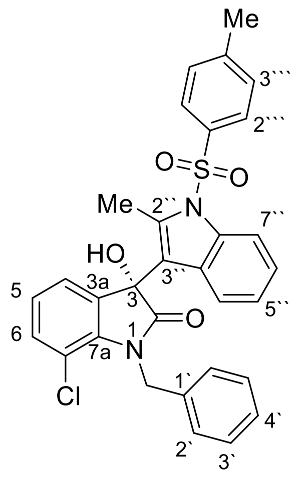

Following **General procedure F**, 1-benzyl-7-chloro-3-[(1-tosyl-1*H*-indol-2-yl)methoxy]indolin-2-one (55.6 mg, 0.1 mmol) and *t*Bu-BIMP (3.7 mg, 0.005 mmol) in 1,4-dioxane (2.0 mL, 0.05 M) at 30 °C for 10 min gave a crude [2,3]-rearrangement product, which was treated with TFA (0.10 mmol) for another 5 h and purified by flash column chromatography (eluent: hexane/acetone = 5:1 to 3:1) to afford the product (45.0 mg, 81%) as a colourless amorphous solid. **IR** ν_{max} (film) 1732 (C=O); **$[\alpha]_D^{20}$** = –224.4 (*c* 0.25 in $CHCl_3$); **Chiral HPLC analysis**, Chiralpak AD-H (80:20 hexane:^{i}PrOH, flow rate 1 ml·min^{-1}, 211 nm, 30 °C) tR (*R*)-**48**: 45.1 min, tR (*S*)-**48**: 50.6 min, 90:10 er; **^{1}H NMR (400 MHz, $CDCl_3$)** δ **2.36** (3H, s, C*H*$_3$C(2``)NSO_2), **2.57** (3H, s, ArC(4```)C*H*$_3$), **3.13** (1H, s, O*H*), **5.33** (1H, d, *J* 15.9, NC*H*^{A}H^{B}Ph), **5.52**

(1H, d, *J* 16.0, NCH^A*H*^{B}Ph), **6.93** (1H, t, *J* 7.8, ArC*H*), **7.11** (1H, t, *J* 7.7, ArC*H*), **7.19–7.25** (5H, m, ArC(3``,5``)*H* and ArC*H*), **7.27–7.35** (6H, m, ArC*H*), **7.63** (2H, d, *J* 8.4, ArC(2```,6```)*H*), **8.20** (1H, d, *J* 8.5, ArC(7``)*H*); **$^{13}$C{$^1$H} NMR (101 MHz, CDCl$_3$)** $\delta_C$: **14.1** (*C*H$_3$C(2``)NSO$_2$), **21.7** (ArC(4```)*C*H$_3$), **45.5** (N*C*H$_2$Ph), **75.6** (*C*(3)OH), **114.9** (Ar*C*(7``)H), **123.7** (Ar*C*H), **124.1** (Ar*C*H), **124.4** (Ar*C*H), **124.8** (Ar*C*H), **126.0** (Ar*C*), **126.6** (Ar*C*(2```,6````)H), **127.1** (Ar*C*(2`,6`)H), **127.7** (Ar*C*H), **128.1** (Ar*C*(3a``)), **128.8** (Ar*C*H), **130.1** (Ar*C*(3```,5```)H), **132.9** (Ar*C*), **133.4** (Ar*C*), **134.9** (Ar*C*), **136.2** (Ar*C*(1```)), **136.7** (Ar*C*), **136.7** (Ar*C*), **138.5** (Ar*C*), **145.1** (Ar*C*(4```)), **142.3** (Ar*C*), **177.1** (*C*=O); **HRMS (ESI$^+$)** $C_{43}H_{33}{}^{35}ClN_2O_4SNa$ [M+Na]$^+$ found 579.1119, requires 579.1116 (0.56 ppm).

(*R*)-3-(5-Bromo-2-methyl-1*H*-indol-3-yl)-3-hydroxy-1-tritylindolin-2-one (49)

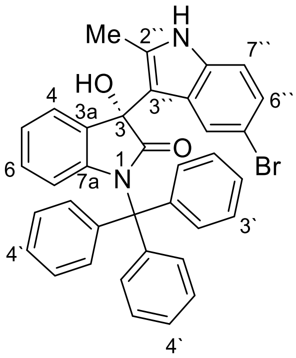

Following **General procedure H**, (3*R*,3'*S*)-5-bromo-3'-hydroxy-2-methylene-1-[(4-nitrophenyl)sulfonyl]-1'-trityl-[3,3'-biindolin]-2'-one (78.3 mg, 0.1 mmol), PhSH (0.3 mmol) and K_2CO_3 (0.5 mmol) in MeCN (5.0 mL, 0.02 M) at rt for 16 h gave a crude product, which was purified by flash column chromatography (eluent: hexane/EtOAc = 3:1 to 1:1) to afford the product (47.8 mg, 80%) as a yellow amorphous solid. **IR** ν_{max} (film) 1716 (C=O), 1606 (C=C), 1463, 1448, 1305, 1263, 1103; **$[\alpha]_D^{20}$** = –24.0 (*c* 0.25 in CHCl$_3$); **Chiral HPLC analysis**, Chiralpak IA (90:10 hexane:^{i}PrOH, flow rate 1 ml·min^{-1}, 211 nm, 30 °C) tR (*R*)-**49**: 16.0 min, tR (*S*)-**49**: 20.8 min, 96:4 er; **^{1}H NMR (500 MHz, CD$_2$Cl$_2$)** δ **2.26** (3H, s, C*H*$_3$C(2``)NSO$_2$), **3.10** (1H, s, O*H*), **6.40** (1H, d, *J* 8.1, ArC(7)*H*), **6.99** (1H, td, *J* 7.5, 1.0, ArC(5)*H*), **7.02–7.09** (2H, m, ArC(6)*H*, ArC*H*), **7.10** (1H, d, *J* 8.5, 1.4, ArC*H*), **7.16** (1H, dd, *J* 8.6, 1.9, ArC*H*), **7.17–7.27** (9H, m, ArC(3`,4`,5`)*H*), **7.31** (1H, dd, *J* 7.3, 1.6, ArC(4)*H*), **7.40–7.46** (6H, m, ArC(2`,6`)*H*), **8.23** (1H, brs, N*H*); **^{13}C{^{1}H} NMR (126 MHz, CD$_2$Cl$_2$)** δ_C: **14.0** (*C*H$_3$C(2``)NSO$_2$), **74.8** (N*C*Ph$_3$), **76.7** (*C*(3)), **109.6** (Ar*C*(3``)), **112.5** (Ar*C*H), **113.4** (Ar*C*H), **116.7** (Ar*C*(7)H), **122.6** (Ar*C*H), **123.3** (Ar*C*(5)H), **124.3** (Ar*C*H), **125.3** (Ar*C*(4)H), **127.4** (Ar*C*(4`)H), **128.3** (Ar*C*(3`,5`)H), **129.1** (Ar*C*(6)H), **129.6** (Ar*C*(2`,6`)H), **132.5** (Ar*C*(3a)), **134.2** (Ar*C*(3a``)), **136.7** (Ar*C*(2``)), **142.5** (Ar*C*(1`)), **143.5** (Ar*C*(7a)), **179.2** (*C*=O); **HRMS (ESI$^+$)** $C_{36}H_{27}{}^{79}BrN_2O_2Na$ [M+Na]$^+$ found 621.1150, requires 621.1148 (0.30 ppm).

6. X-ray diffraction data

X-ray diffraction data for all compounds were collected at either 125 or 100 K using a Rigaku MM-007HF High Brilliance RA generator/confocal optics [Cu Kα radiation (λ = 1.54187 Å)] with XtaLAB P200 diffractometer. Intensity data for all compounds analysed were collected using either CrystalClear[36] (using ω steps and accumulating area detector images spanning at least a hemisphere of reciprocal space) or CrysAlisPro[37] (using a calculated strategy), and processed (including correction for Lorentz, polarization and absorption) using CrysAlisPro. Structures were solved by dual-space methods (SHELXT[38]) and refined by full-matrix least-squares against F^2 (SHELXL-2019/3[39]). Non-hydrogen atoms were refined anisotropically, and hydrogen atoms were refined using a riding model except for OH/NH hydrogens, which were located from the difference Fourier map and refined isotropically subject to a distance restraint. In **7**, the substituent on the hydroxyoxindole ring was seen to be disordered equally over two orientations. The benzene ring of the tosyl group was constrained to ideality, and several other bond distance restraints were required in the disordered part of the molecule. All calculations were performed using the Olex2[40] interface. Selected crystallographic data are presented in Table S1. CCDC 2344436-2344438 contains the supplementary crystallographic data for this paper. These data can be obtained free of charge from The Cambridge Crystallographic Data Centre via www.ccdc.cam.ac.uk/structures.

Table S1. Selected crystallographic data.

compound number	**7**	**15**	**39**
CCDC number	**2344436**	**2344437**	**2344438**
formula	$C_{24}H_{20}N_2O_4S$	$C_{44}H_{36}N_2O_4S$	$C_{44}H_{36}N_2O_5S$
fw	432.48	688.81	704.84
crystal description	Colorless needle	Colorless plate	Colorless rod
crystal size [mm^3]	0.26×0.03×0.01	0.08×0.05×0.01	0.32×0.04×0.02
temperature [K]	125	100	100
space group	$P2_12_12$	$P2_1$	$P2_12_12_1$
a [Å]	7.26830(10)	8.91594(13)	9.79044(6)
b [Å]	31.6959(5)	9.26039(17)	16.97625(12)
c [Å]	10.8979(2)	20.9739(4)	21.38996(13)
β [°]		90.0888(14)	
vol [Å]^3	2510.61(7)	1731.71(5)	3555.12(4)
Z	4	2	4
ρ (calc) [g/cm^3]	1.144	1.321	1.317
μ [mm^{-1}]	1.387	1.214	1.217
F(000)	904	724	1480
reflections collected	29954	64082	71692
independent reflections (R_{int})	5114 (0.0435)	6995 (0.0591)	7377 (0.0574)
parameters, restraints	444, 10	466, 2	476, 1
GoF on F^2	1.083	1.062	1.053
R_1 [$I > 2\sigma(I)$]	0.0962	0.0402	0.0397
wR_2 (all data)	0.2726	0.1044	0.1031
largest diff. peak/hole [e/Å^3]	0.775, -0.304	0.178, -0.411	0.758, -0.577
Flack parameter	0.041(9)	0.003(9)	0.020(5)

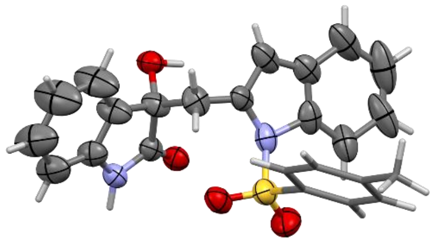

Figure S8: Thermal ellipsoid plot of the structure of **7**. Ellipsoids are drawn at the 50 % probability level, only one orientation of the disorder is shown, and the tolyl group of the tosyl is drawn as sticks only for clarity.

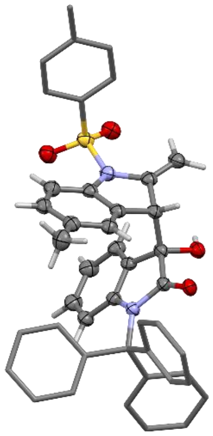

Figure S9: Thermal ellipsoid plot of the structure of **15**. Ellipsoids are drawn at the 50 % probability level, and both the trityl group and tolyl group of the tosyl are drawn as sticks only, with hydrogens omitted, for clarity.

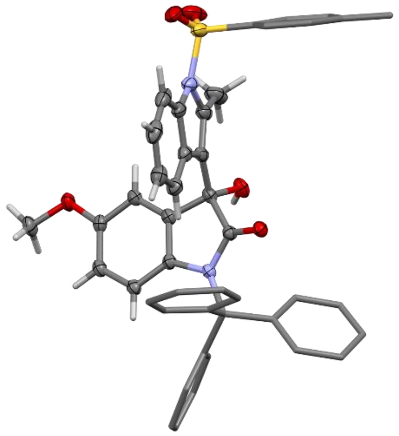

Figure S10: Thermal ellipsoid plot of the structure of **39**. Ellipsoids are drawn at the 50 % probability level, and both the trityl group and tolyl group of the tosyl are drawn as sticks only, with hydrogens omitted, for clarity.

7. References

(1) Neese, F. Software Update: The ORCA Program System—Version 5.0. *Wiley Interdiscip. Rev. Comput. Mol. Sci.* **2022**, *12*, 1–15.

(2) Zhao, Y.; Truhlar, D. G. The M06 Suite of Density Functionals for Main Group Thermochemistry, Thermochemical Kinetics, Noncovalent Interactions, Excited States, and Transition Elements: Two New Functionals and Systematic Testing of Four M06-Class Functionals and 12 Other Function. *Theor. Chem. Acc.* **2008**, *120*, 215–241.

(3) Weigend, F.; Ahlrichs, R. Balanced Basis Sets of Split Valence, Triple Zeta Valence and Quadruple Zeta Valence Quality for H to Rn: Design and Assessment of Accuracy. *Phys. Chem. Chem. Phys.* **2005**, *7*, 3297–3305.

(4) Barone, V.; Cossi, M. Quantum Calculation of Molecular Energies and Energy Gradients in Solution by a Conductor Solvent Model. *J. Phys. Chem. A* **1998**, *102*, 1995–2001.

(5) Mardirossian, N.; Head-Gordon, M. How Accurate Are the Minnesota Density Functionals for Noncovalent Interactions, Isomerization Energies, Thermochemistry, and Barrier Heights Involving Molecules Composed of Main-Group Elements? *J. Chem. Theory Comput.* **2016**, *12*, 4303–4325.

(6) Wheeler, S. E.; Houk, K. N. Integration Grid Errors for Meta-Gga-Predicted Reaction Energies: Origin of Grid Errors for the M06 Suite of Functionals. *J. Chem. Theory Comput.* **2010**, *6*, 395–404.

(7) Bootsma, A. N.; Wheeler, S. E. Popular Integration Grids Can Result in Large Errors in DFT-Computed Free Energies. *ChemRxiv* **2019**, 1–20.

(8) Pracht, P.; Bohle, F.; Grimme, S. Automated Exploration of the Low-Energy Chemical Space with Fast Quantum Chemical Methods. *Phys. Chem. Chem. Phys.* **2020**, *22*, 7169–7192.

(9) Grimme, S. Exploration of Chemical Compound, Conformer, and Reaction Space with Meta-Dynamics Simulations Based on Tight-Binding Quantum Chemical Calculations. *J. Chem. Theory Comput.* **2019**, *15*, 2847–2862.

(10) Ehlert, S.; Stahn, M.; Spicher, S.; Grimme, S. Robust and Efficient Implicit Solvation Model for Fast Semiempirical Methods. *J. Chem. Theory Comput.* **2021**, *17*, 4250–4261.

(11) Neese, F. Software Update: The ORCA Program System—Version 5.0. *WIREs Comput. Mol. Sci.* **2022**, *12*.

(12) Paton, R. S.; Luchini, G. PyQRC: Version 1.0.3. 2021.

(13) Frisch, M. J.; Trucks, G. W.; Schlegel, H. B.; Scuseria, G. E.; Robb, M. A.; Cheeseman, J. R.; Scalmani, G.; Barone, V.; Petersson, G. A.; Nakatsuji, H.; Li, X.; Caricato, M.; Marenich, A. V; Bloino, J.; Janesko, B. G.; Gomperts, R.; Mennucci, B.; Hratchian, H. P.; Ortiz, J. V; Izmaylov, A. F.; Sonnenberg, J. L.; Williams-Young, D.; Ding, F.; Lipparini, F.; Egidi, F.; Goings, J.; Peng, B.; Petrone, A.; Henderson, T.; Ranasinghe, D.; Zakrzewski, V. G.; Gao, J.; Rega, N.; Zheng, G.; Liang, W.; Hada, M.; Ehara, M.; Toyota, K.; Fukuda, R.; Hasegawa, J.; Ishida, M.; Nakajima, T.; Honda, Y.; Kitao, O.; Nakai, H.; Vreven, T.; Throssell, K.; Montgomery, Jr., J. A.; Peralta, J. E.; Ogliaro, F.; Bearpark, M. J.; Heyd, J. J.; Brothers, E. N.; Kudin, K. N.; Staroverov, V. N.; Keith, T. A.; Kobayashi, R.; Normand, J.; Raghavachari, K.; Rendell, A. P.; Burant, J. C.; Iyengar,

S. S.; Tomasi, J.; Cossi, M.; Millam, J. M.; Klene, M.; Adamo, C.; Cammi, R.; Ochterski, J. W.; Martin, R. L.; Morokuma, K.; Farkas, O.; Foresman, J. B.; Fox, D. J. *Gaussian 16, Revision C.01*; Gaussian Inc.: Wallingford CT, 2019.

(14) Marenich, A. V.; Cramer, C. J.; Truhlar, D. G. Universal Solvation Model Based on Solute Electron Density and on a Continuum Model of the Solvent Defined by the Bulk Dielectric Constant and Atomic Surface Tensions. *J. Phys. Chem. B* **2009**, *113*, 6378–6396.

(15) Luchini, G.; Alegre-Requena, J. V; Funes-Ardoiz, I.; Paton, R. S. GoodVibes: Automated Thermochemistry for Heterogeneous Computational Chemistry Data. *F1000Research (Chem. Inf. Sci.)* **2020**, *9*, 291.

(16) Martin, R. L.; Hay, P. J.; Pratt, L. R. Hydrolysis of Ferric Ion in Water and Conformational Equilibrium. *J. Phys. Chem. A* **1998**, *102* (20), 3565–3573.

(17) Legault, C. Y. CYLview20. *Univ. Sherbrooke* **2020**, www.cylview.org.

(18) The PyMOL Molecular Graphics System, Version 2.4.1. Schrödinger, LLC.

(19) Boto, R. A.; Peccati, F.; Laplaza, R.; Quan, C.; Carbone, A.; Piquemal, J. P.; Maday, Y.; Contreras-García, J. NCIPLOT4: Fast, Robust, and Quantitative Analysis of Noncovalent Interactions. *J. Chem. Theory Comput.* **2020**, *16*, 4150–4158.

(20) Ferres, L.; Stahl, W.; Kleiner, I.; Nguyen, H. V. L. The Effect of Internal Rotation in P-Methyl Anisole Studied by Microwave Spectroscopy. *J. Mol. Spectrosc.* **2018**, *343*, 44–49.

(21) Breen, P. J.; Warren, J. A.; Bernstein, E. R.; Seeman, J. I. A Study of Nonrigid Aromatic Molecules by Supersonic Molecular Jet Spectroscopy. II. Propyltoluenes. *J. Chem. Phys.* **1987**, *87*, 1927–1935.

(22) Held, A.; Selzle, H. L.; Schlag, E. W. Methyl Group Rotational Dynamics in O-, m-, and p-Xylene Cations from Pulsed Field Ionization Zero-Kinetic-Energy Spectroscopy. *J. Phys. Chem. A* **1998**, *102*, 9625–9630.

(23) Grimme, S. Supramolecular Binding Thermodynamics by Dispersion-Corrected Density Functional Theory. *Chem. - A Eur. J.* **2012**, *18*, 9955–9964.

(24) Ribeiro, R. F.; Marenich, A. V.; Cramer, C. J.; Truhlar, D. G. Use of Solution-Phase Vibrational Frequencies in Continuum Models for the Free Energy of Solvation. *J. Phys. Chem. B* **2011**, *115*, 14556–14562.

(25) Biswas, B.; Collins, S. C.; Singleton, D. A. Dynamics and a Unified Understanding of Competitive [2,3]- and [1,2]-Sigmatropic Rearrangements Based on a Study of Ammonium Ylides. *J. Am. Chem. Soc.* **2014**, *136*, 3740–3743.

(26) Iribarren, I.; Trujillo, C. Efficiency and Suitability When Exploring the Conformational Space of Phase-Transfer Catalysts. *J. Chem. Inf. Model.* **2022**, *62*, 5568–5580.

(27) Bickelhaupt, F. M.; Houk, K. N. Analyzing Reaction Rates with the Distortion/Interaction-Activation Strain Model. *Angew. Chemie Int. Ed.* **2017**, *56*, 10070–10086.

(28) Zhao, Z.; Wolkenberg, S. E.; Lu, M.; Munshi, V.; Moyer, G.; Feng, M.; Carella, A. V.; Ecto, L. T.; Gabryelski, L. J.; Lai, M.-T.; Prasad, S. G.; Yan, Y.; McGaughey, G. B.;

Miller, M. D.; Lindsley, C. W.; Hartman, G. D.; Vacca, J. P.; Williams, T. M.; *Bioorg. Med. Chem. Lett.* **2008**, *18*, 554–559.

(29) McNulty, J.; Keskar, K.; Bordón, C.; Yolken, R.; Jones-Brando, L.; *Chem. Commun.*, **2014**, *50*, 8904–8907.

(30) Wang, R.; Mo, S.; Lu, Y.; Shen, Z.; *Adv. Synth. Catal.* **2011**, *353*, 713–718.

(31) Tiziana, B.; Andrea, M.; Tullio, P.; Alessandro, P.; Simona, R.; Francesco, S.; *Chem. Eur. J.* **2009**, *15*, 94–105.

(32) Tian, Q.; Bai, J.; Chen, B.; Zhang, G.; *Org. Lett.* **2016**, *18*, 1828–1831.

(33) O'Hara, C.; Yang, C.-H.; Francis, A. J.; Newell, B. S.; Wang, H.; Resendiz, M. J. E.; *J. Org. Chem.* **2019**, *84*, 9714–9725.

(34) Carpino, L. A.; Ismail, M.; Truran, G. A.; Mansour, M. E.; Iguchi, S.; Ionescu, D.; EI-Faham, A.; Riemer, Christoph.; Warrass, Ralf.; *J. Org. Chem.* **1994**, *64*, 4324–4338.

(35) Fu, J.; Wurzer, N.; Lehner, V.; Reiser, O.; Davies, H. M. L.; *Org. Lett.* **2019**, *21*, 6102–6106.

(36) *CrystalClear-SM Exper*t v2.1. Rigaku Americas, *The Woodlands, Texas, USA*, and Rigaku Corporation, *Tokyo, Japan*, 2015.

(37) *CrysAlisPro* v1.171.42.49, v1.171.42.94a, v1.171.43.98a. Rigaku Oxford Diffraction, Rigaku Corporation, *Oxford, U.K.*, 2022-2023.

(38) Sheldrick, G. M. SHELXT – Integrated space-group and crystal structure determination. *Acta Crystallogr., Sect. A: Found. Adv.* **2015**, *71*, 3-8.

(39) Sheldrick, G. M. Crystal structure refinement with SHELXL. *Acta Crystallogr., Sect. C: Struct. Chem.* **2015**, *71*, 3-8.

(40) Dolomanov, O. V.; Bourhis, L. J.; Gildea, R. J.; Howard, J. A. K.; Puschmann, H. OLEX2: a complete structure solution, refinement and analysis program. *J. Appl. Crystallogr*. **2009**, *42*, 339-341.

8. HPLC data of chiral compounds

Chiral HPLC analysis, Chiralpak IA (80:20 hexane: ^{i}PrOH, flow rate 1 ml·min^{-1}, 211 nm, 30 °C) tR (*S*)-**S1**: 20.3 min, tR (*R*)-**S1**: 22.9 min, 96:4 er.

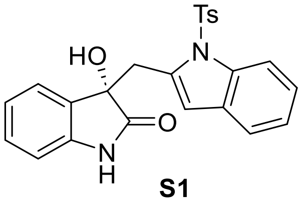

S1

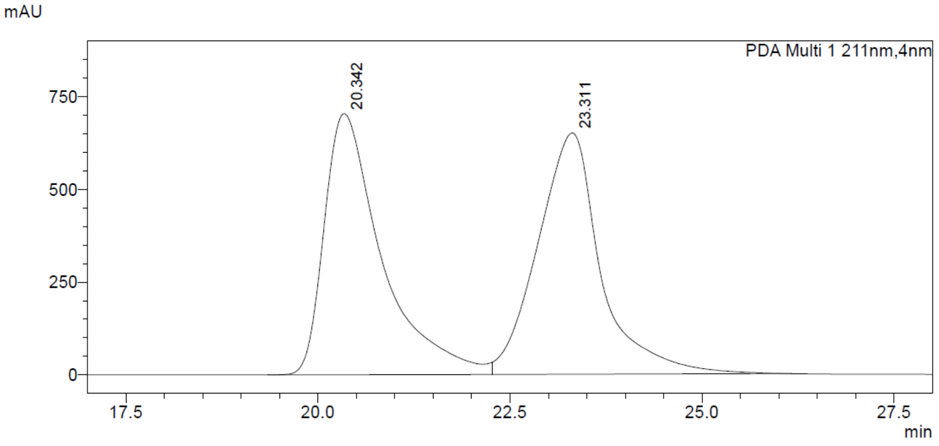

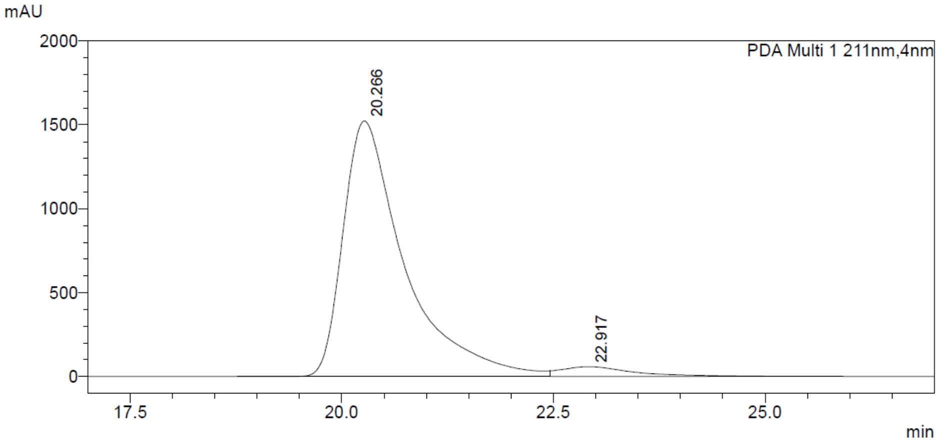

Racemic			Enantioenriched		
PDA Ch1 211nm			PDA Ch1 211nm		
Peak#	Ret. Time	Area%	Peak#	Ret. Time	Area%
1	20.342	49.750	1	20.266	95.725
2	23.311	50.250	2	22.917	4.275
Total		100.000	Total		100.000

Chiral HPLC analysis, Chiralpak ID (80:20 hexane:^{i}PrOH, flow rate 1 ml·min^{-1}, 211 nm, 30 °C) major diastereomer: tR (3*S*,3'*R*)-**3**: 19.0 min, tR (3*R*,3'*R*)-**3**: 38.8 min, 2:98 er; minor diastereomer tR (3*S*,3'*S*)-**3**: 23.0 min, tR (3*R*,3'*S*)-**3**: 27.1 min, 1:99 er.

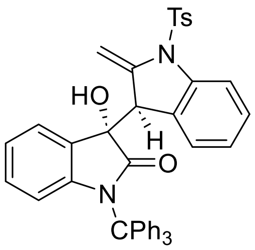

3

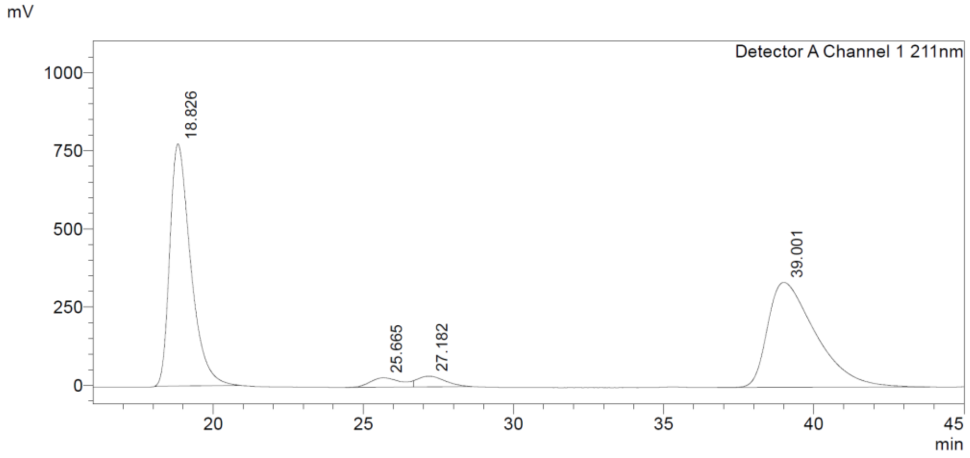

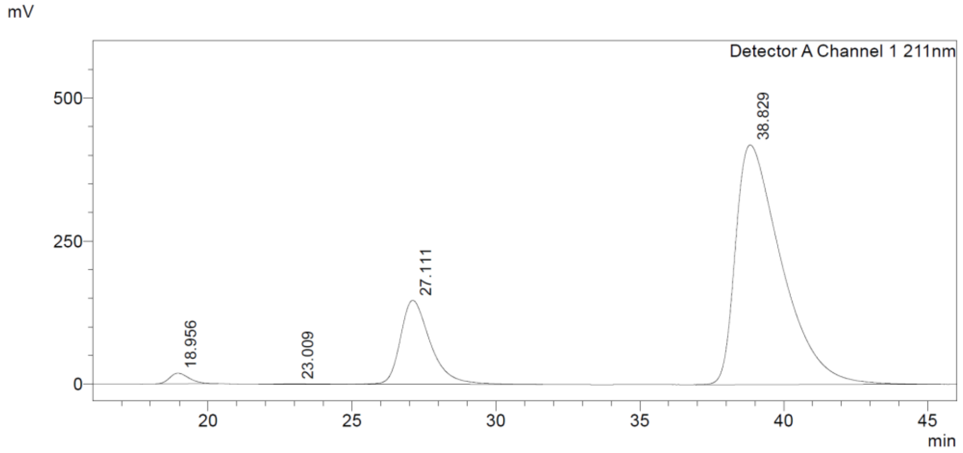

Racemic			Enantioenriched		
Detector A Channel 1 211nm			Detector A Channel 1 211nm		
Peak#	Ret. Time	Area%	Peak#	Ret. Time	Area%
1	18.826	47.297	1	18.956	1.500
2	25.665	2.755	2	23.009	0.138
3	27.182	2.748	3	27.111	18.359
4	39.001	47.200	4	38.829	80.003
Total		100.000	Total		100.000

Chiral HPLC analysis, Chiralpak IC (90:10 hexane:^{i}PrOH, flow rate 1 ml·min^{-1}, 211 nm, 30 °C) major diastereomer: tR (3*S*,3'*R*)-**8**: 5.3 min, tR (3*R*,3'*S*)-**8**: 6.3 min, 3:97 er; minor diastereomer tR (3*R*,3'*R*)-**8**: 10.9 min, tR (3*S*,3'*S*)-**8**: 15.9 min, 13:87 er.

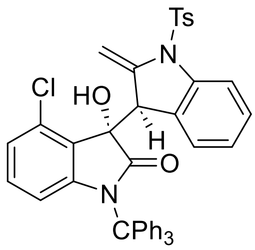

8

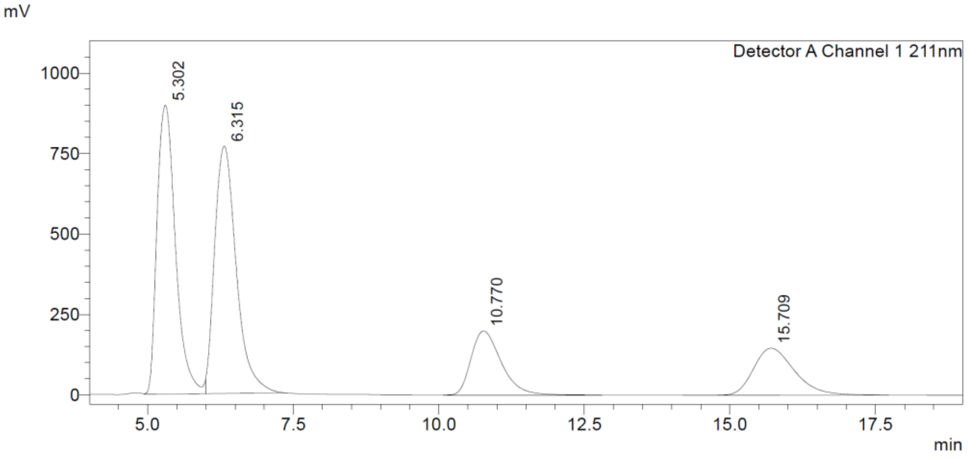

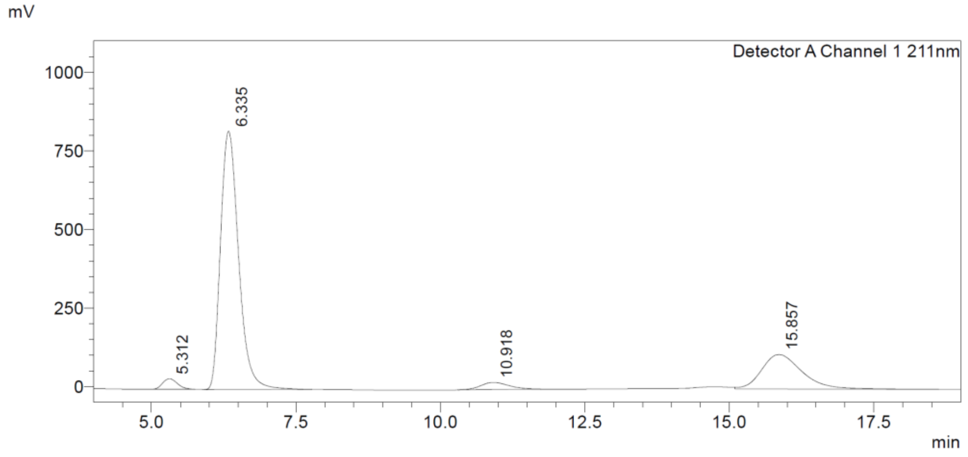

Racemic — Detector A Channel 1 211nm

Peak#	Ret. Time	Area%
1	5.302	36.707
2	6.315	36.812
3	10.770	13.287
4	15.709	13.193
Total		100.000

Enantioenriched — Detector A Channel 1 211nm

Peak#	Ret. Time	Area%
1	5.312	2.378
2	6.335	72.776
3	10.918	3.226
4	15.857	21.620
Total		100.000

Chiral HPLC analysis, Chiralpak IA (85:15 hexane:^{i}PrOH, flow rate 1 ml·min^{-1}, 211 nm, 30 °C) major diastereomer: tR (3*S*,3'*R*)-**9**: 23.6 min, tR (3*R*,3'*S*)-**9**: 26.0 min, 2:98 er; minor diastereomer tR (3*S*,3'*S*)-**9**: 31.1 min, tR (3*R*,3'*R*)-**9**: 40.2 min, 99:1 er.

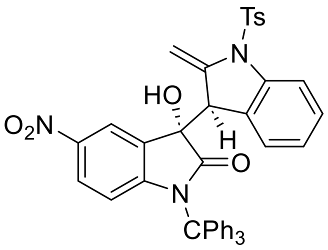

9

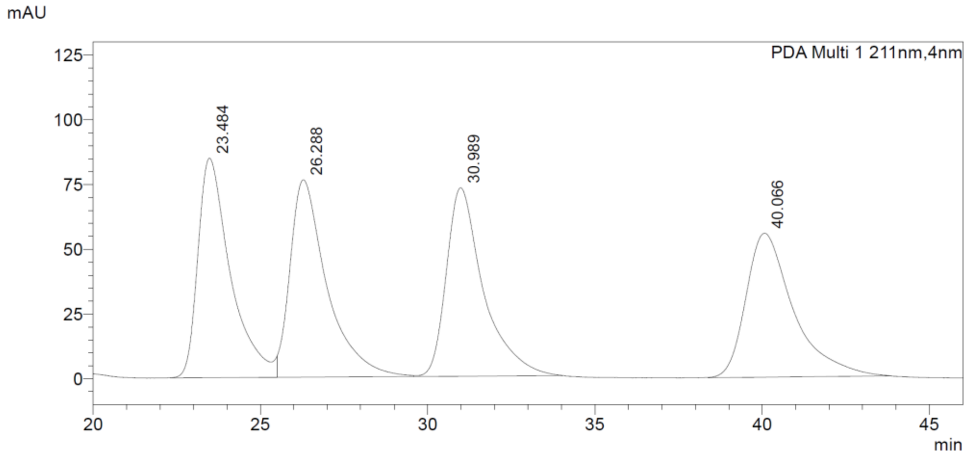

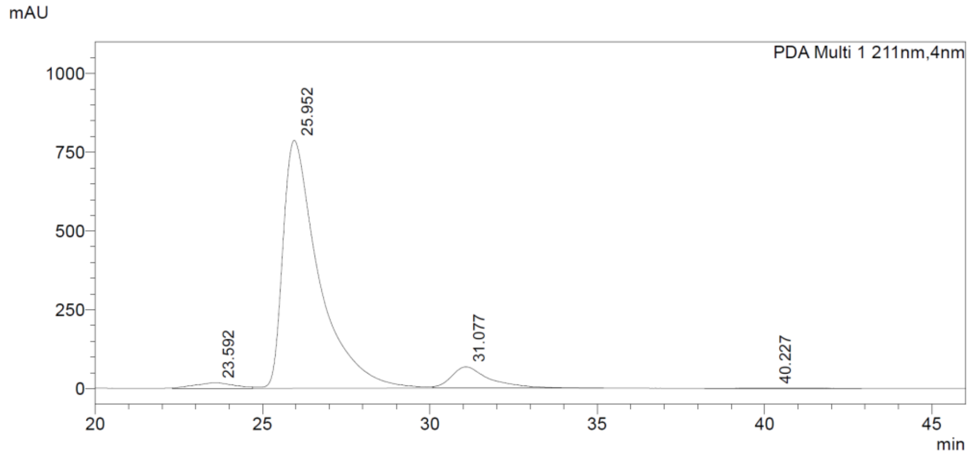

Racemic			Enantioenriched		
PDA Ch1 211nm			PDA Ch1 211nm		
Peak#	Ret. Time	Area%	Peak#	Ret. Time	Area%
1	23.484	25.366	1	23.592	2.145
2	26.288	25.652	2	25.952	89.496
3	30.989	24.728	3	31.077	8.260
4	40.066	24.254	4	40.227	0.100
Total		100.000	Total		100.000

Chiral HPLC analysis, Chiralpak ID (80:20 hexane:^{i}PrOH, flow rate 1 ml·min^{-1}, 211 nm, 30 °C) major diastereomer: tR (3*S*,3'*R*)-**10**: 17.6 min, tR (3*S*,3'*S*)-**10**: 26.4 min, 2:98 er; minor diastereomer tR (3*R*,3'*S*)-**10**: 22.9 min, tR (3*R*,3'*R*)-**10**: 43.0 min, 97:3 er.

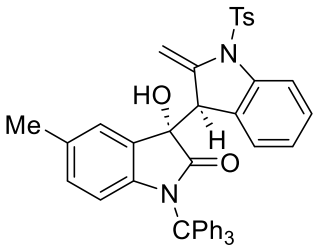

10

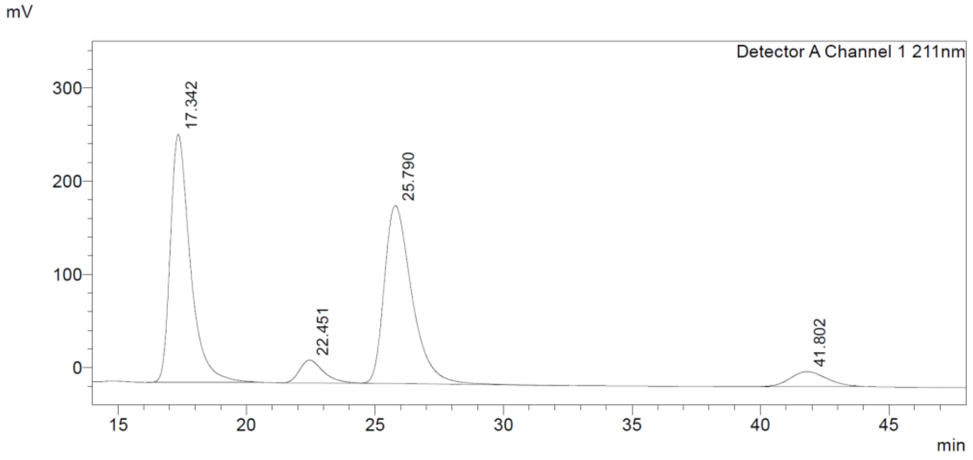

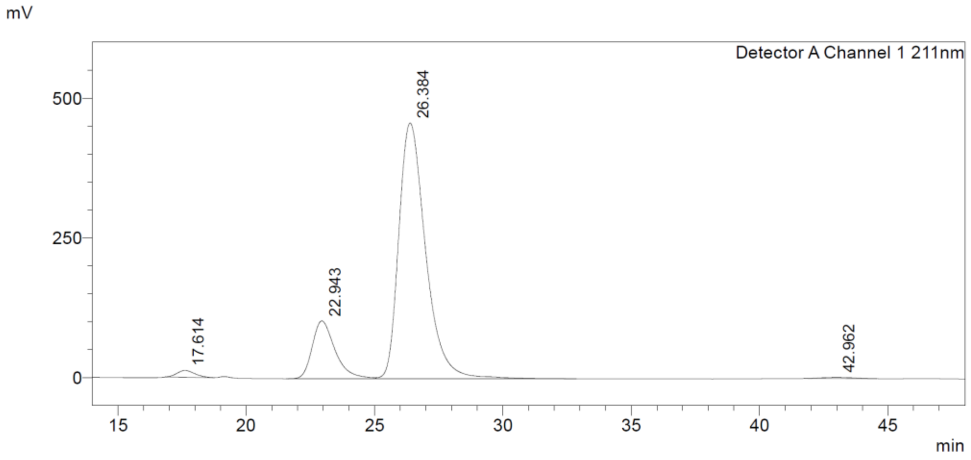

Racemic — Detector A Channel 1 211nm

Peak#	Ret. Time	Area%
1	17.342	45.242
2	22.451	4.959
3	25.790	44.878
4	41.802	4.921
Total		100.000

Enantioenriched — Detector A Channel 1 211nm

Peak#	Ret. Time	Area%
1	17.614	1.422
2	22.943	15.888
3	26.384	82.197
4	42.962	0.493
Total		100.000

Chiral HPLC analysis, Chiralpak IA (85:15 hexane:^{i}PrOH, flow rate 1 ml·min^{-1}, 211 nm, 30 °C) major diastereomer: tR (3*R*,3'*S*)-**11**: 32.0 min, tR (3*S*,3'*R*)-**11**: 41.8 min, 98:2 er.

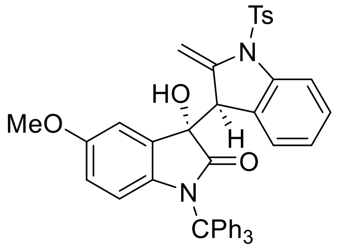

11

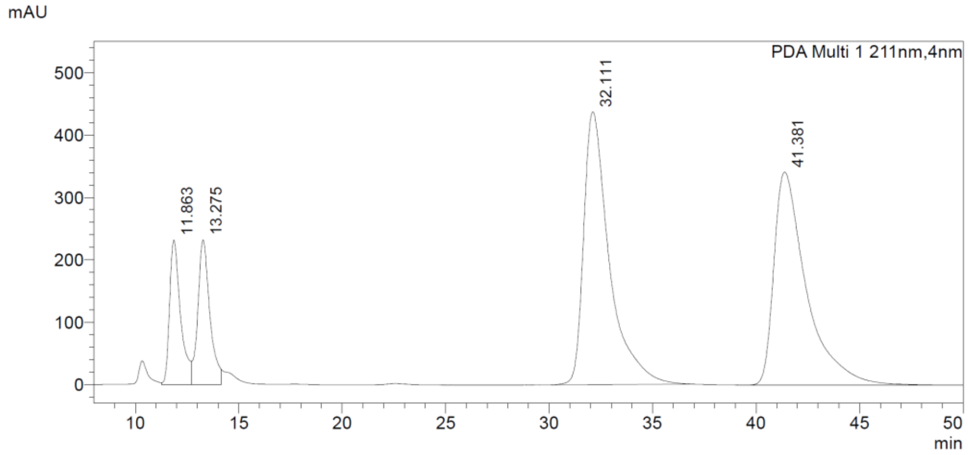

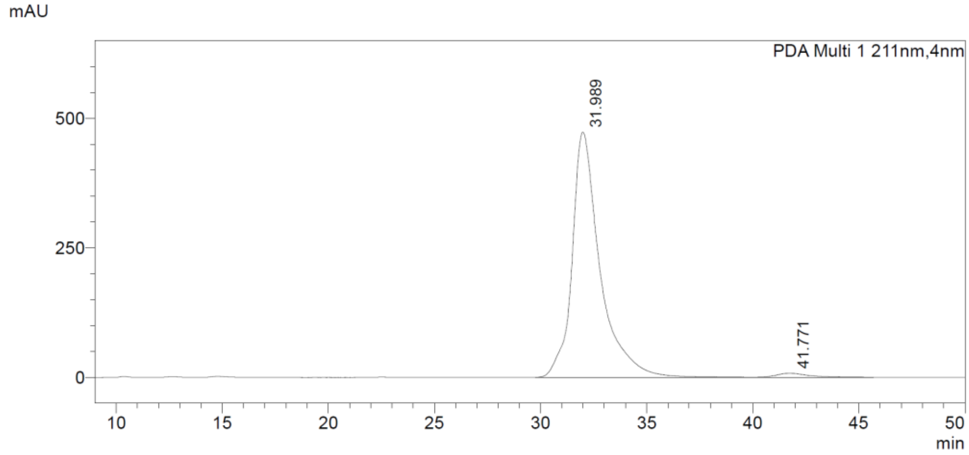

Racemic	Enantioenriched
PDA Ch1 211nm	PDA Ch1 211nm

Racemic:

Peak#	Ret. Time	Area%
1	11.863	9.236
2	13.275	9.993
3	32.111	40.676
4	41.381	40.095
Total		100.000

Enantioenriched:

Peak#	Ret. Time	Area%
1	31.989	98.250
2	41.771	1.750
Total		100.000

Chiral HPLC analysis, Chiralpak ID (90:10 hexane:^{i}PrOH, flow rate 1 ml·min^{-1}, 211 nm, 30 °C) major diastereomer: tR (3*S*,3'*R*)-**12**: 12.4 min, tR (3*R*,3'*S*)-**12**: 18.0 min, 4:96 er.

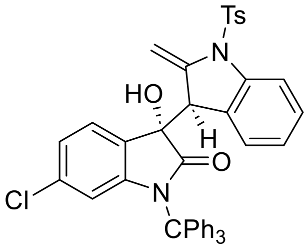

12

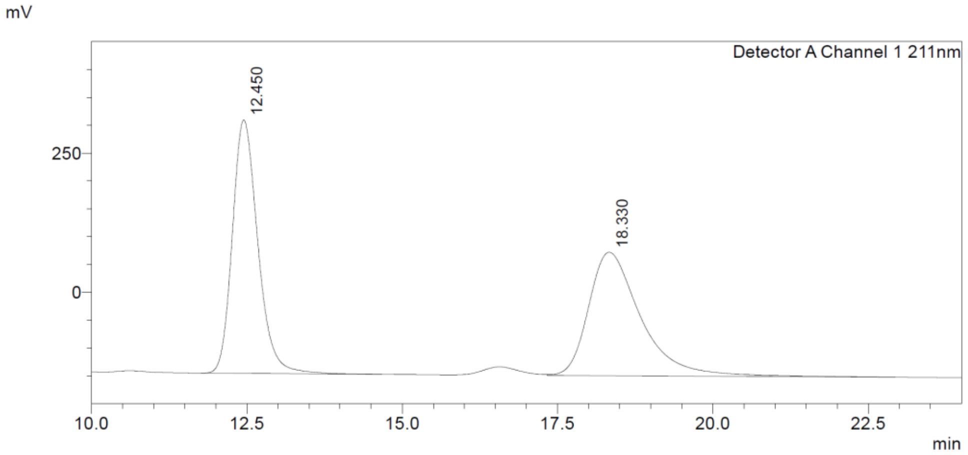

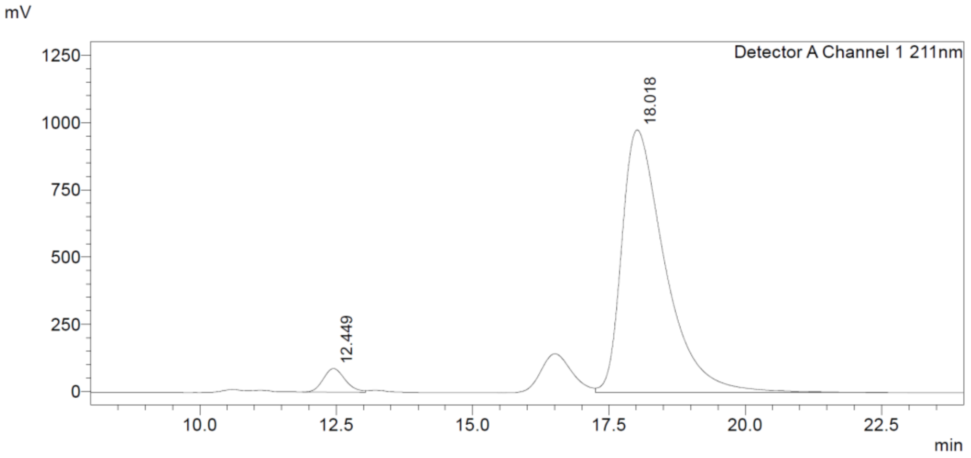

Racemic			Enantioenriched		
Detector A Channel 1 211nm			Detector A Channel 1 211nm		
Peak#	Ret. Time	Area%	Peak#	Ret. Time	Area%
1	12.450	50.075	1	12.449	4.337
2	18.330	49.925	2	18.018	95.663
Total		100.000	Total		100.000

Chiral HPLC analysis, Chiralpak IA (85:15 hexane:^{i}PrOH, flow rate 1 ml·min^{-1}, 211 nm, 30 °C) major diastereomer: tR (3*S*,3'*R*)-**13**: 19.6 min, tR (3*R*,3'*S*)-**13**: 25.9 min, 3:97 er.

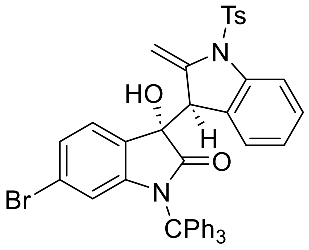

13

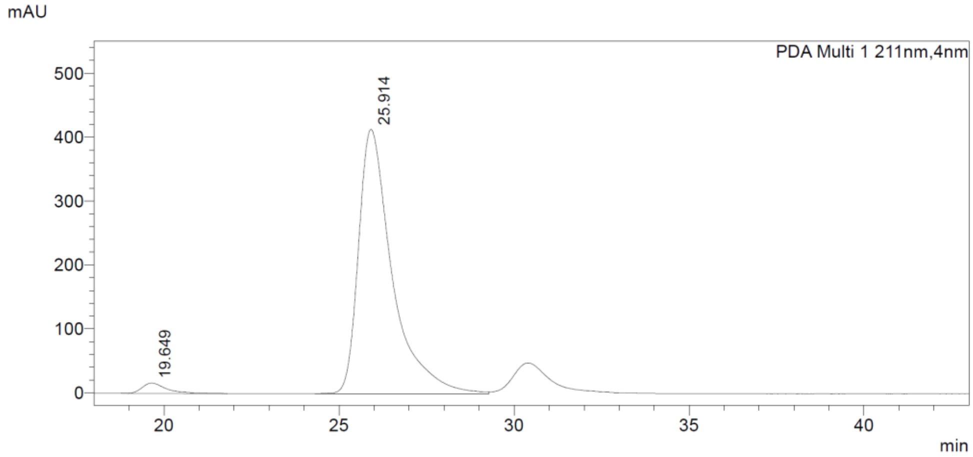

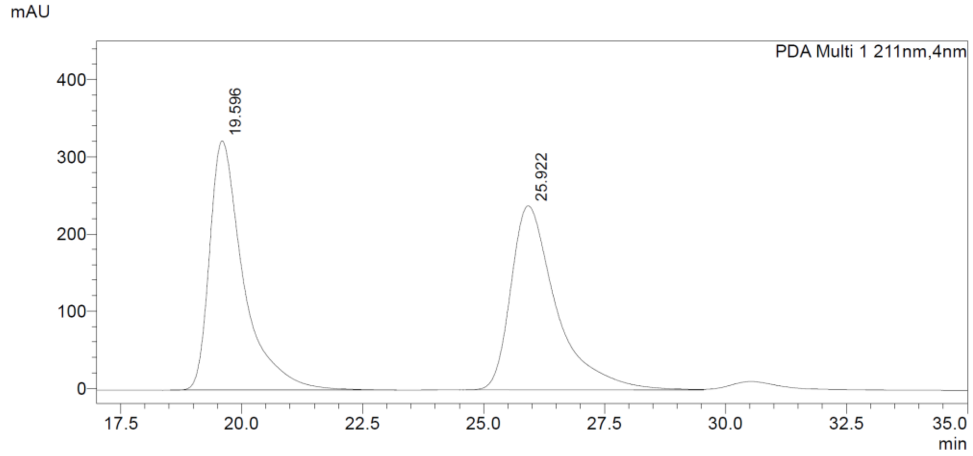

Racemic			Enantioenriched		
PDA Ch1 211nm			PDA Ch1 211nm		
Peak#	Ret. Time	Area%	Peak#	Ret. Time	Area%
1	19.596	50.245	1	19.649	2.812
2	25.922	49.755	2	25.914	97.188
Total		100.000	Total		100.000

Chiral HPLC analysis, Chiralpak ID (80:20 hexane:^{i}PrOH, flow rate 1 ml·min^{-1}, 211 nm, 30 °C) major diastereomer: tR (3*S*,3'*S*)-**14**: 15.5 min, tR (3*S*,3'*R*)-**14**: 19.7 min, tR (3*R*,3'*R*)-**14**: 27.1 min, tR (3*R*,3'*S*)-**14**: 35.9 min, 96:4 er.

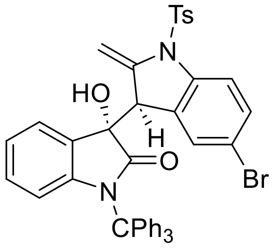

14

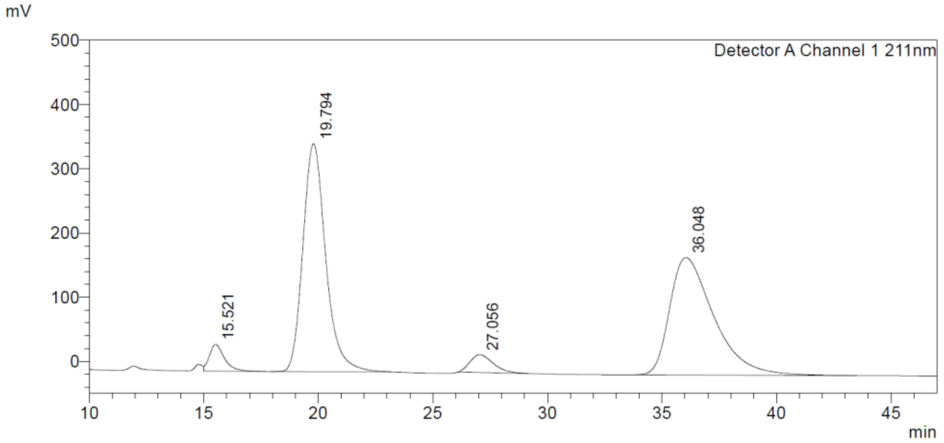

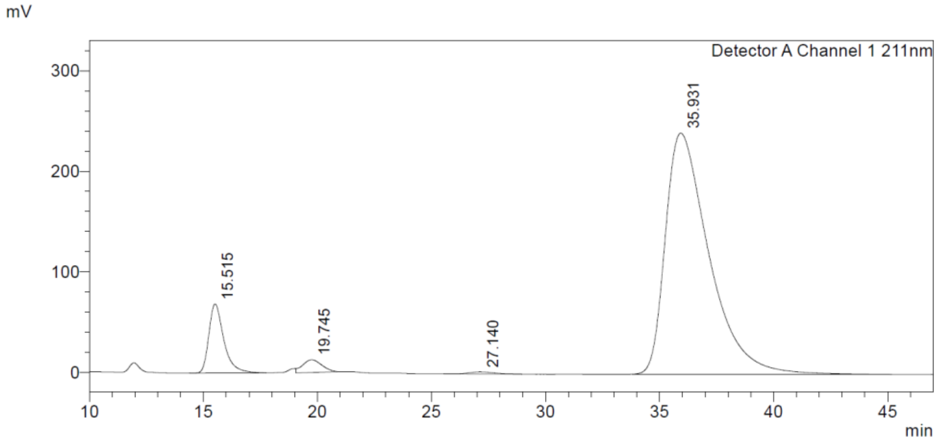

Racemic			Enantioenriched		
Detector A Channel 1 211nm			Detector A Channel 1 211nm		
Peak#	Ret. Time	Area%	Peak#	Ret. Time	Area%
1	15.521	3.616	1	15.515	8.739
2	19.794	46.408	2	19.745	2.076
3	27.056	3.680	3	27.140	0.423
4	36.048	46.296	4	35.931	88.762
Total		100.000	Total		100.000

Chiral HPLC analysis, Chiralpak ID (80:20 hexane:^{i}PrOH, flow rate 1 ml·min^{-1}, 211 nm, 30 °C) minor diastereomer: tR (3*S*,3'*S*)-**15**: 21.1 min, tR (3*S*,3'*R*)-**15**: 44.7 min, 97:3 er; major diastereomer tR (3*R*,3'*R*)-**15**: 25.0 min, tR (3*R*,3'*S*)-**15**: 66.0 min, 98:2 er.

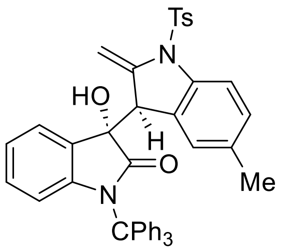

15

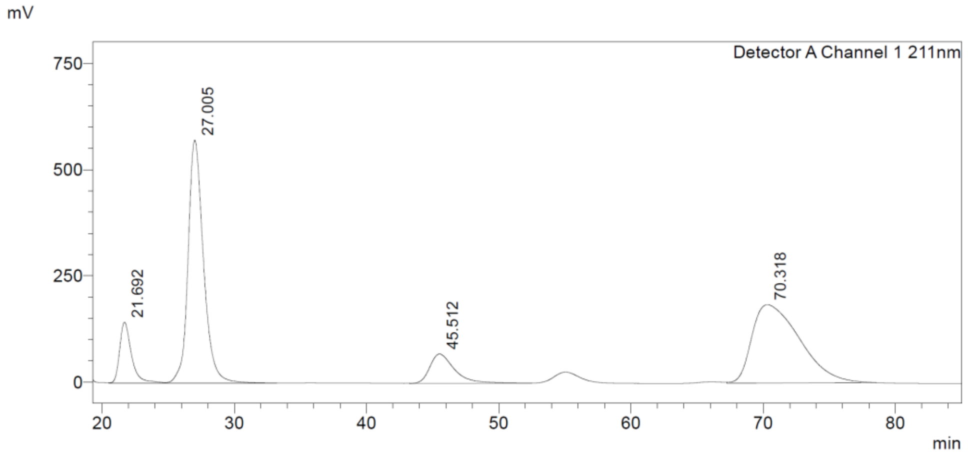

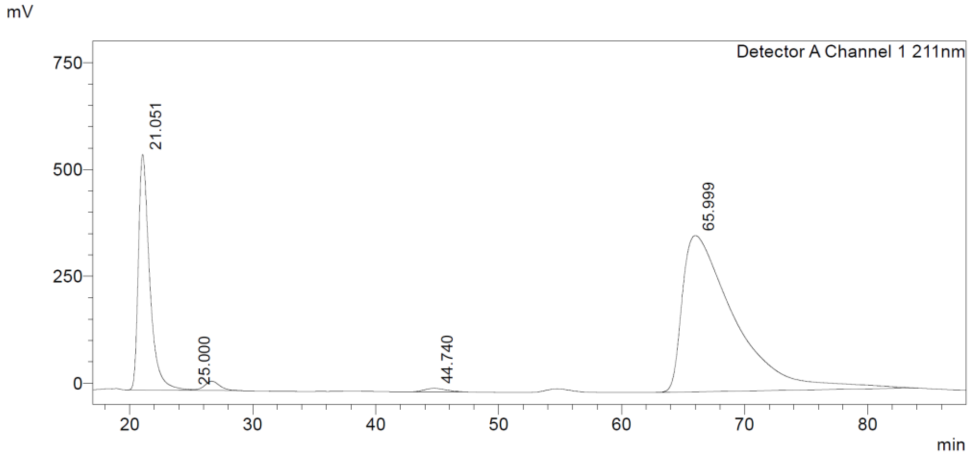

Racemic

Detector A Channel 1 211nm

Peak#	Ret. Time	Area%
1	21.692	8.031
2	27.005	41.847
3	45.512	8.221
4	70.318	41.901
Total		100.000

Enantioenriched

Detector A Channel 1 211nm

Peak#	Ret. Time	Area%
1	21.051	23.907
2	25.000	1.304
3	44.740	0.705
4	65.999	74.084
Total		100.000

Chiral HPLC analysis, Chiralpak IA (85:15 hexane:^{i}PrOH, flow rate 1 ml·min^{-1}, 211 nm, 30 °C) major diastereomer: tR (3*S*,3'*R*)-**16**: 16.9 min, tR (3*R*,3'*S*)-**16**: 28.6 min, 2:98 er; minor diastereomer tR (3*S*,3'*S*)-**16**: 34.3 min, tR (3*R*,3'*R*)-**16**: 61.1 min, 98:2 er.

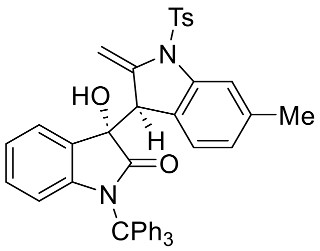

16

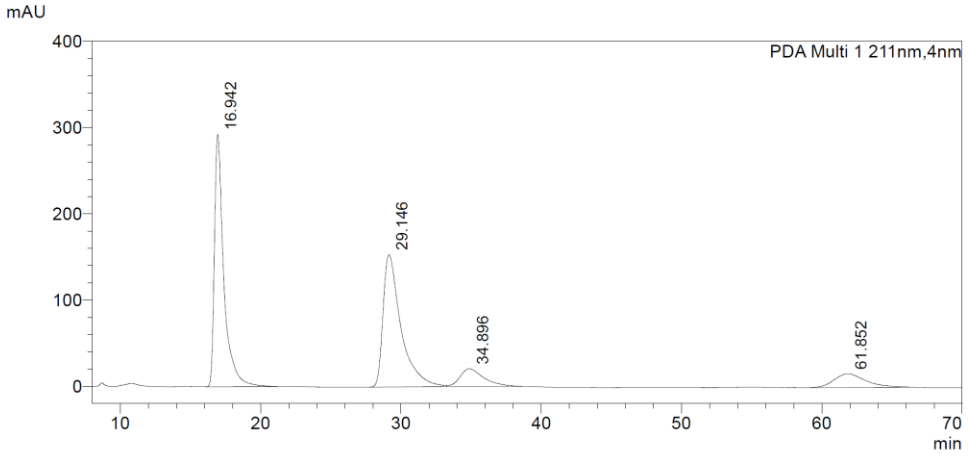

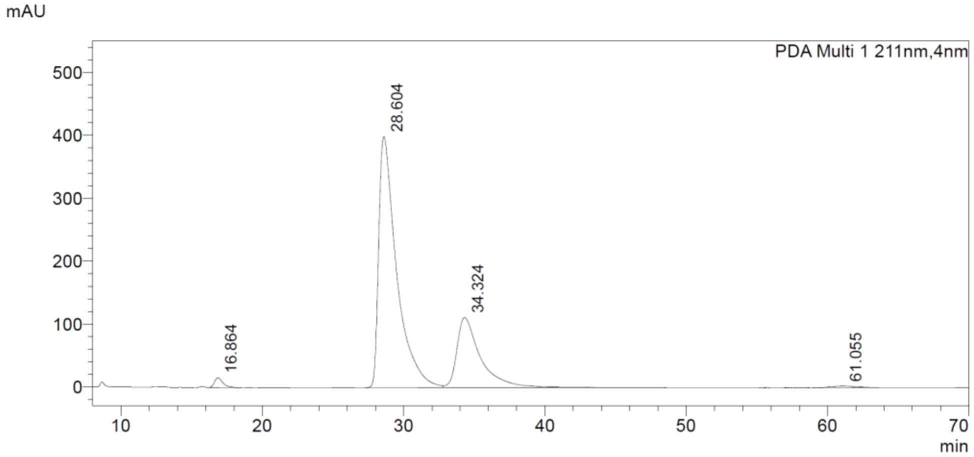

Racemic			Enantioenriched		
PDA Ch1 211nm			PDA Ch1 211nm		
Peak#	Ret. Time	Area%	Peak#	Ret. Time	Area%
1	16.942	42.458	1	16.864	1.342
2	29.146	42.359	2	28.604	71.814
3	34.896	7.625	3	34.324	26.240
4	61.852	7.559	4	61.055	0.604
Total		100.000	Total		100.000

Chiral HPLC analysis, Chiralpak ID (90:10 hexane:^{i}PrOH, flow rate 1 ml·min^{-1}, 211 nm, 30 °C) major diastereomer: tR (3*S*,3'*S*)-**17**: 8.3 min, tR (3*R*,3'*R*)-**17**: 9.0 min, 95:5 er; minor diastereomer tR (3*S*,3'*R*)-**17**: 9.7 min, tR (3*R*,3'*S*)-**17**: 11.6 min, 92:8 er.

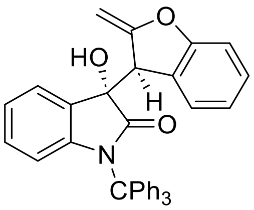

17

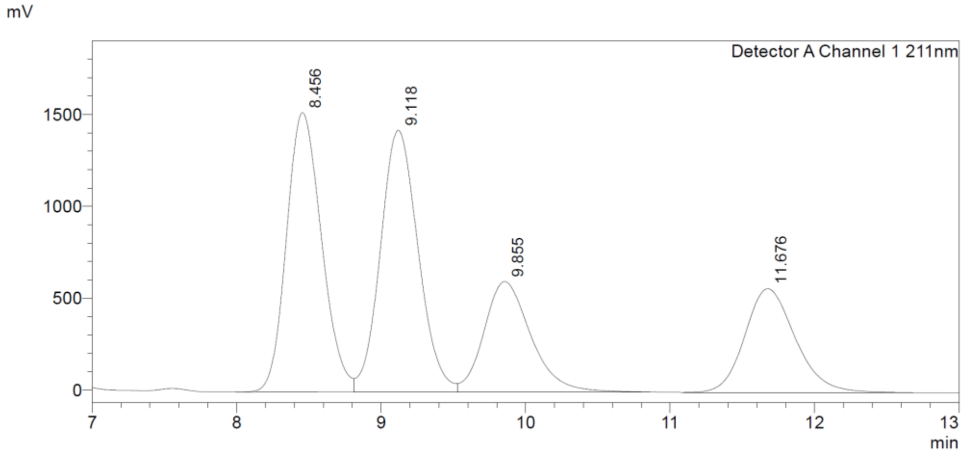

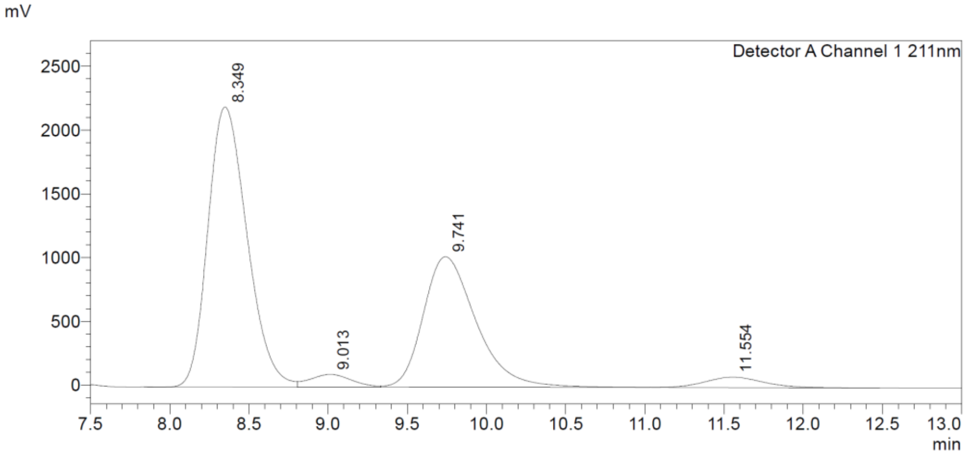

Racemic

Detector A Channel 1 211nm

Peak#	Ret. Time	Area%
1	8.456	32.585
2	9.118	32.803
3	9.855	17.202
4	11.676	17.409
Total		100.000

Enantioenriched

Detector A Channel 1 211nm

Peak#	Ret. Time	Area%
1	8.349	57.769
2	9.013	2.808
3	9.741	36.227
4	11.554	3.196
Total		100.000

Chiral HPLC analysis, Chiralpak AD-H (85:15 hexane:^{i}PrOH, flow rate 1 ml·min^{-1}, 211 nm, 30 °C) major diastereomer: tR (3*R*,3'*S*)-**18**: 43.3 min, tR (3*S*,3'*R*)-**18**: 50.8 min, 96:4 er.

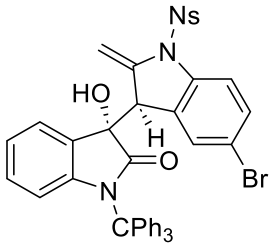

18

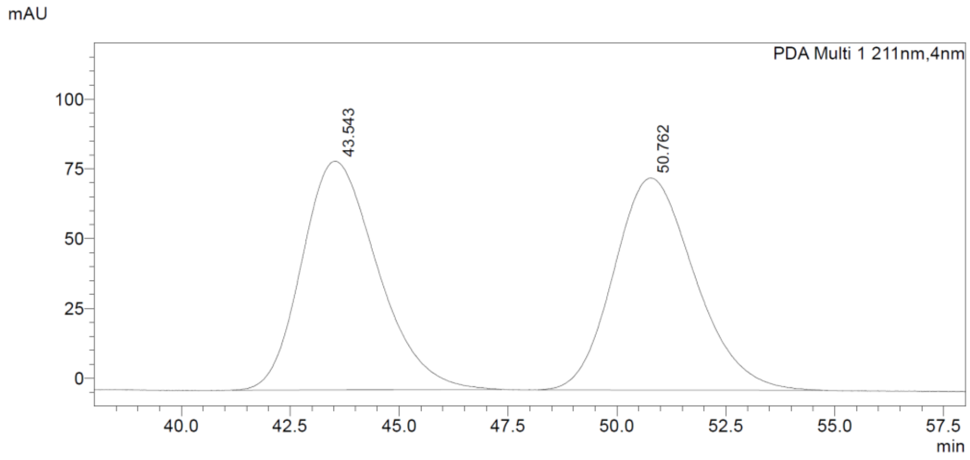

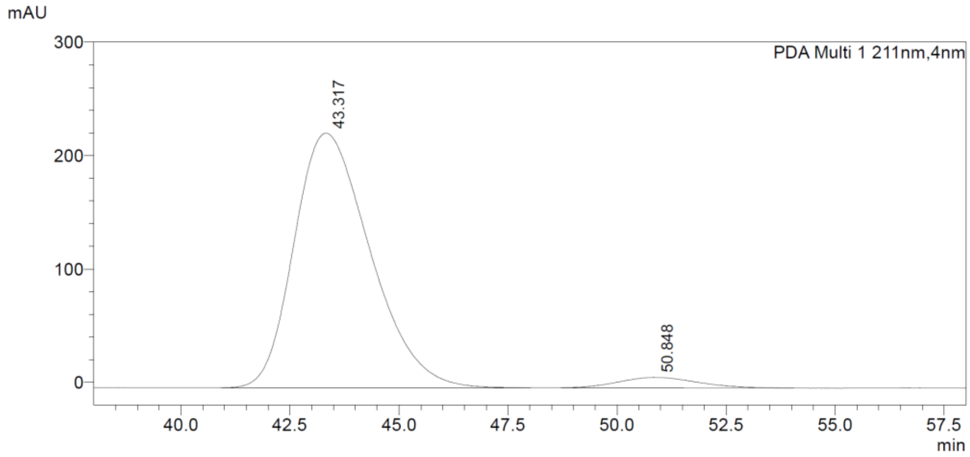

Racemic	Enantioenriched
PDA Ch1 211nm	PDA Ch1 211nm

Racemic:

Peak#	Ret. Time	Area%
1	43.543	50.135
2	50.762	49.865
Total		100.000

Enantioenriched:

Peak#	Ret. Time	Area%
1	43.317	95.941
2	50.848	4.059
Total		100.000

Chiral HPLC analysis, Chiralpak OD-H (90:10 hexane:^{i}PrOH, flow rate 1 ml·min^{-1}, 211 nm, 30 °C) tR (*R*)-**6**: 13.3 min, tR (*S*)-**6**: 16.6 min, 4:96 er.

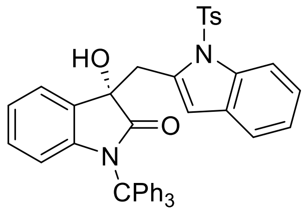

6

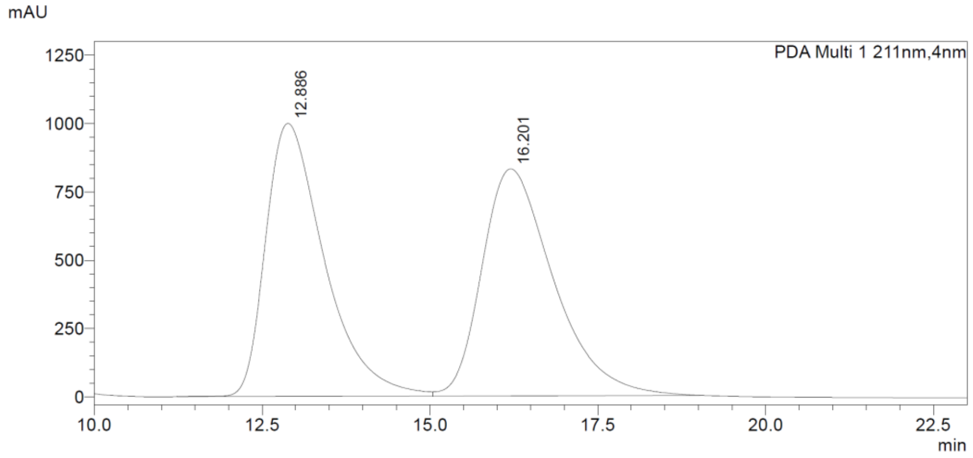

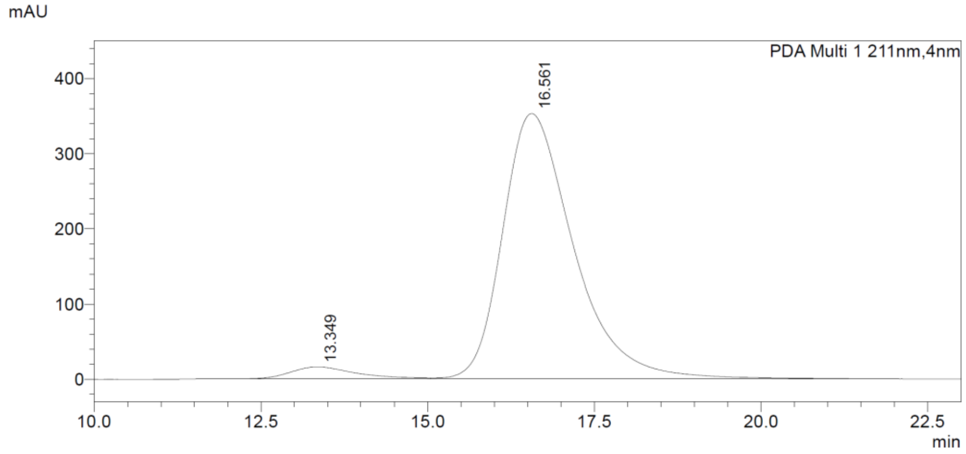

Racemic	Enantioenriched
PDA Ch1 211nm	PDA Ch1 211nm

Racemic:

Peak#	Ret. Time	Area%
1	13.349	3.842
2	16.561	96.158
Total		100.000

Enantioenriched:

Peak#	Ret. Time	Area%
1	12.886	49.612
2	16.201	50.388
Total		100.000

Chiral HPLC analysis, Chiralpak OD-H (95:5 hexane:^{i}PrOH, flow rate 1 ml·min^{-1}, 211 nm, 30 °C) tR (*R*)-**19**: 18.4 min, tR (*S*)-**19**: 24.0 min, 7:93 er.

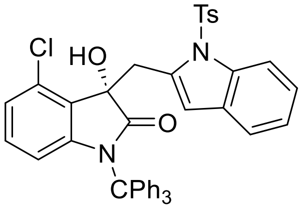

19

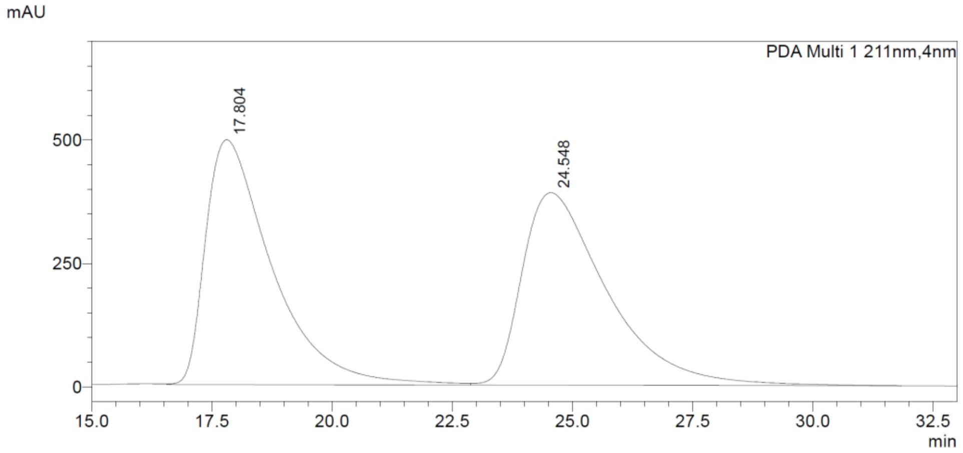

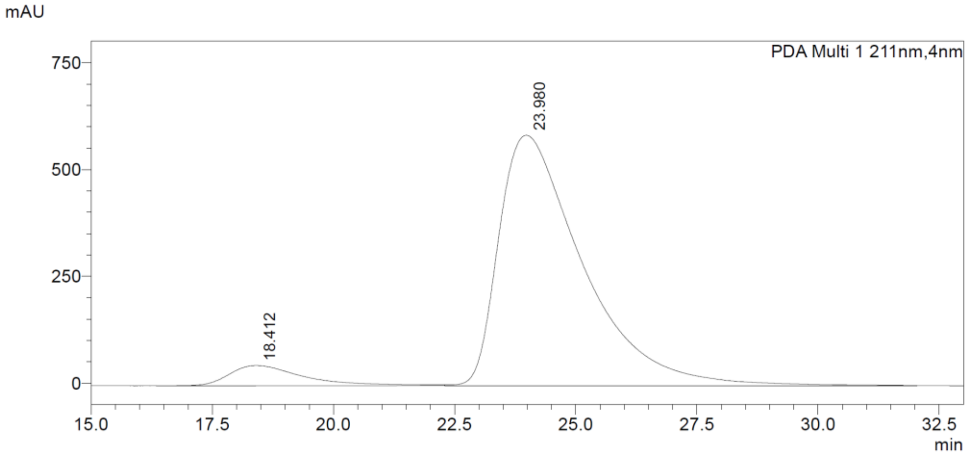

Racemic			Enantioenriched		
PDA Ch1 211nm			PDA Ch1 211nm		
Peak#	Ret. Time	Area%	Peak#	Ret. Time	Area%
1	17.804	49.938	1	18.412	6.620
2	24.548	50.062	2	23.980	93.380
Total		100.000	Total		100.000

Chiral HPLC analysis, Chiralpak IA (80:20 hexane:^{i}PrOH, flow rate 1 ml·min^{-1}, 211 nm, 30 °C) tR (*R*)-**20**: 23.9 min, tR (*S*)-**20**: 37.2 min, 5:95 er.

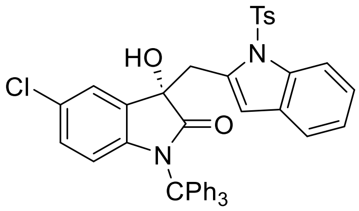

20

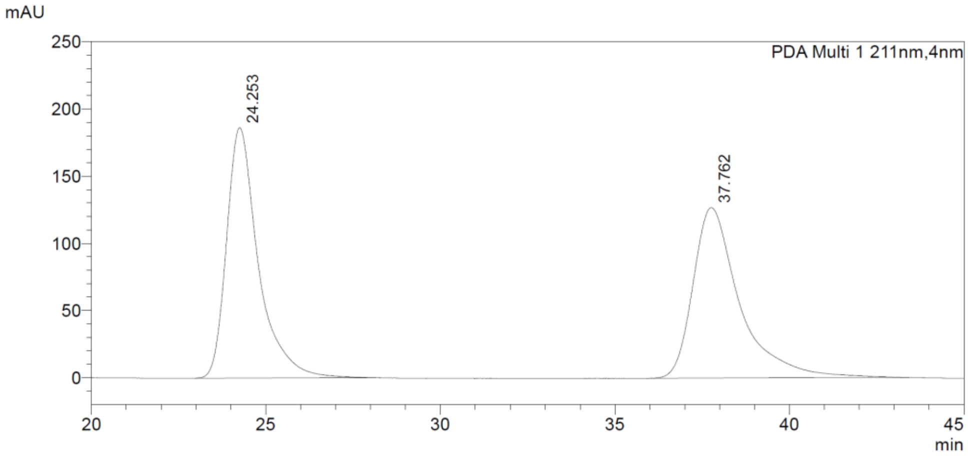

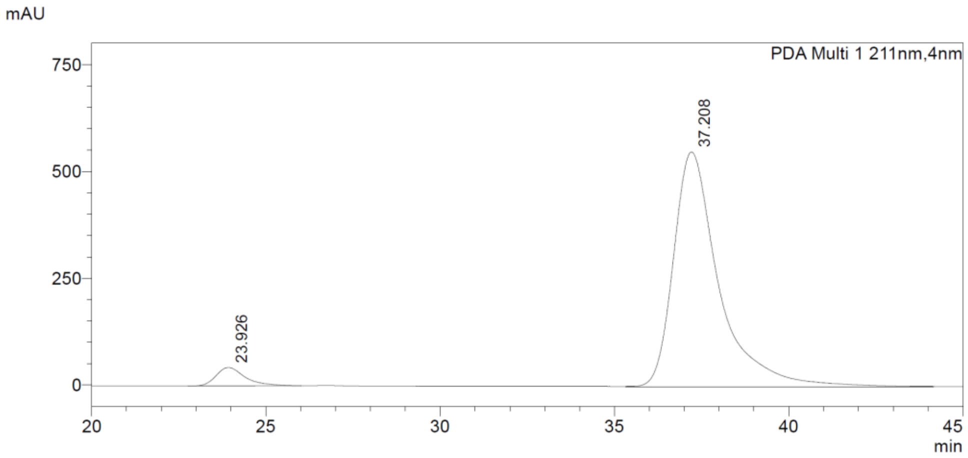

Racemic

PDA Ch1 211nm

Peak#	Ret. Time	Area%
1	24.253	49.902
2	37.762	50.098
Total		100.000

Enantioenriched

PDA Ch1 211nm

Peak#	Ret. Time	Area%
1	23.926	4.801
2	37.208	95.199
Total		100.000

Chiral HPLC analysis, Chiralpak OD-H (95:5 hexane:^{i}PrOH, flow rate 1 ml·min^{-1}, 211 nm, 30 °C) tR (*R*)-**21**: 44.7 min, tR (*S*)-**21**: 55.3 min, 3:97 er.

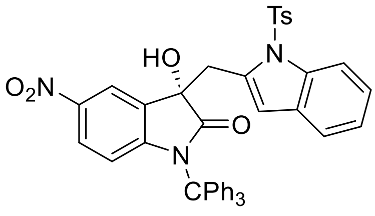

21

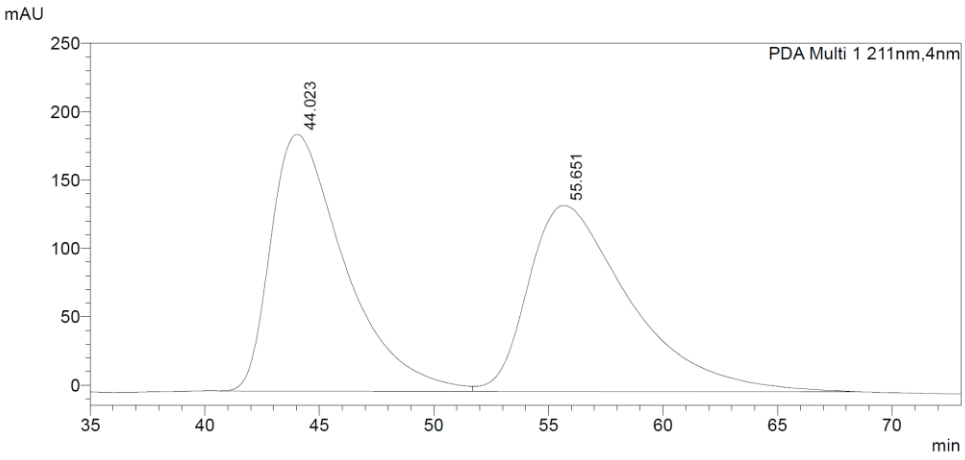

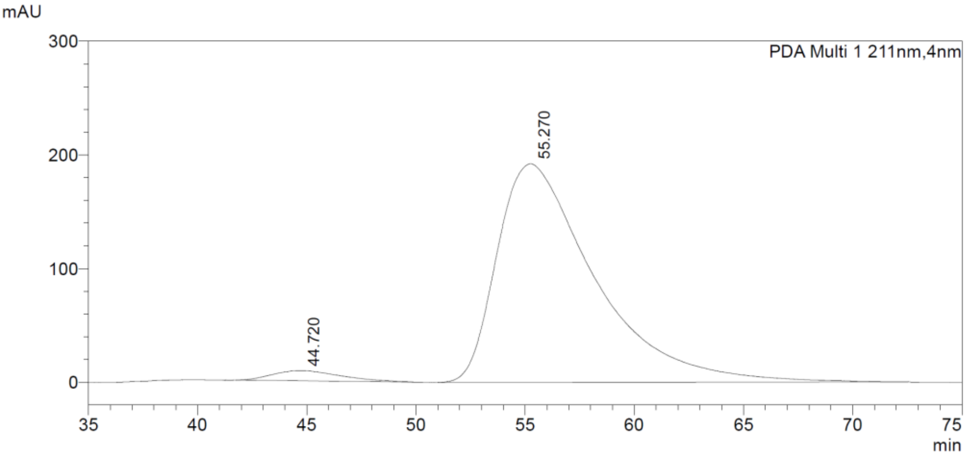

Racemic — PDA Ch1 211nm

Peak#	Ret. Time	Area%
1	44.023	50.092
2	55.651	49.908
Total		100.000

Enantioenriched — PDA Ch1 211nm

Peak#	Ret. Time	Area%
1	44.720	3.119
2	55.270	96.881
Total		100.000

Chiral HPLC analysis, Chiralpak IA (80:20 hexane:^{i}PrOH, flow rate 1 ml·min^{-1}, 211 nm, 30 °C) tR (*R*)-**22**: 25.7 min, tR (*S*)-**22**: 34.7 min, 5:95 er.

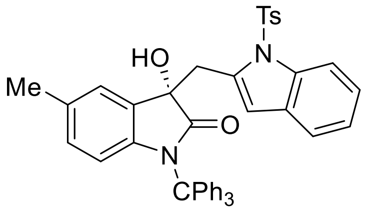

22

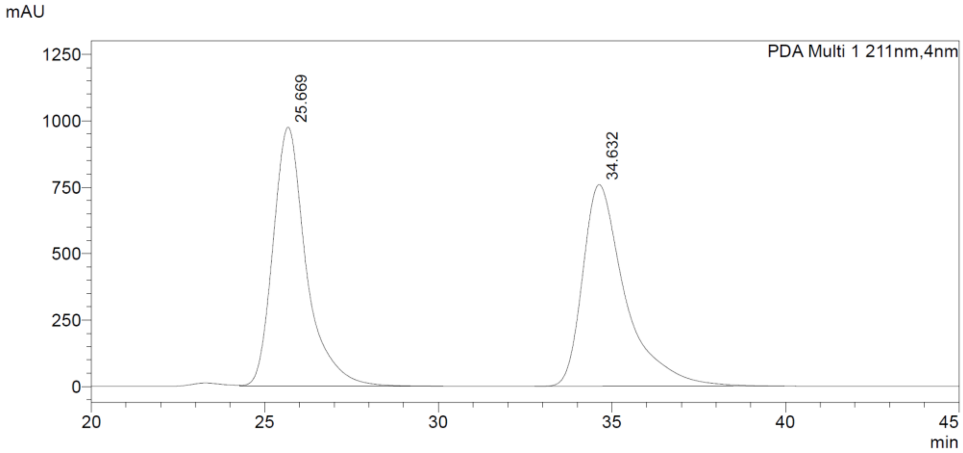

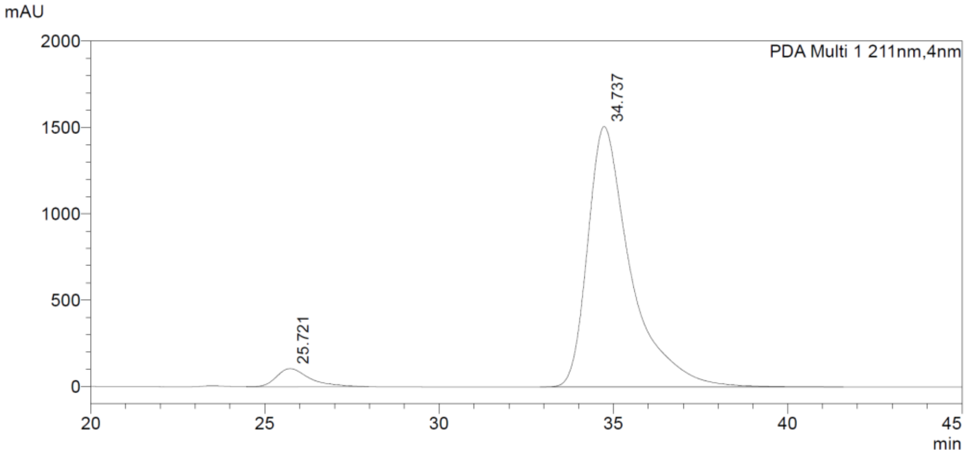

Racemic

PDA Ch1 211nm

Peak#	Ret. Time	Area%
1	25.669	50.049
2	34.632	49.951
Total		100.000

Enantioenriched

PDA Ch1 211nm

Peak#	Ret. Time	Area%
1	25.721	5.083
2	34.737	94.917
Total		100.000

Chiral HPLC analysis, Chiralpak IA (80:20 hexane:^{i}PrOH, flow rate 1 ml·min^{-1}, 211 nm, 30 °C) tR (*S*)-**23**: 19.6 min, tR (*R*)-**23**: 49.3 min, 95:5 er.

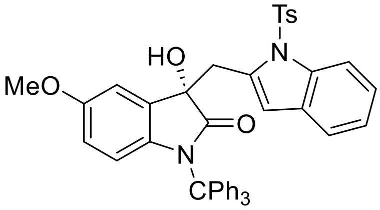

23

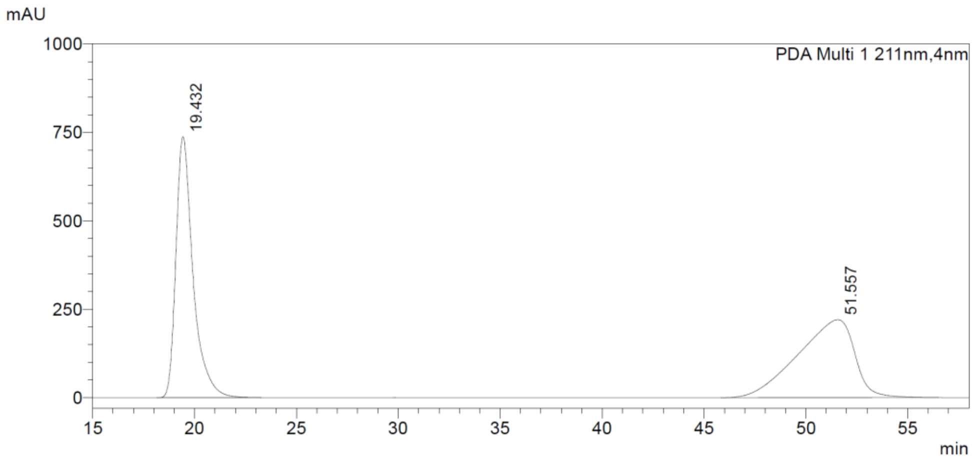

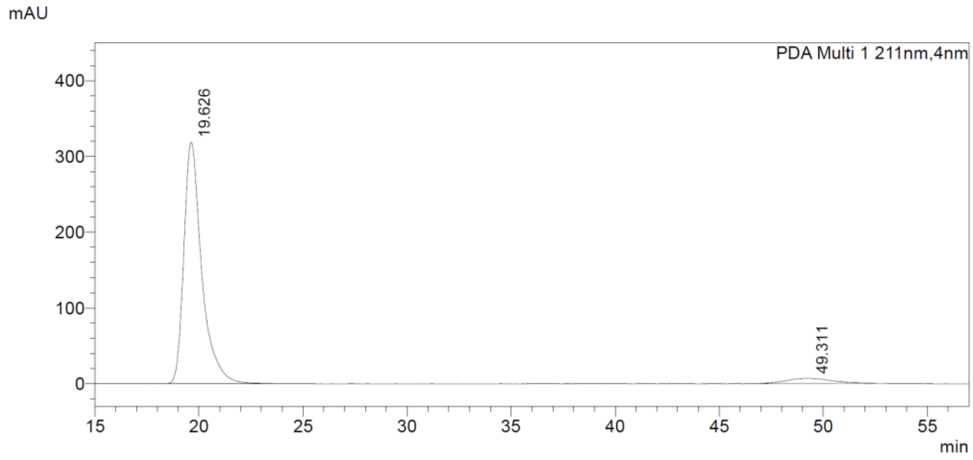

Racemic	Enantioenriched
PDA Ch1 211nm	PDA Ch1 211nm

Racemic — PDA Ch1 211nm

Peak#	Ret. Time	Area%
1	19.432	50.098
2	51.557	49.902
Total		100.000

Enantioenriched — PDA Ch1 211nm

Peak#	Ret. Time	Area%
1	19.626	94.765
2	49.311	5.235
Total		100.000

Chiral HPLC analysis, Chiralpak IB (95:5 hexane:PrOH, flow rate 1 ml·min^{-1}, 211 nm, 30 °C) tR (*R*)-**24**: 25.8 min, tR (*S*)-**24**: 31.9 min, 5:95 er.

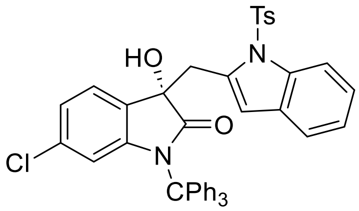

24

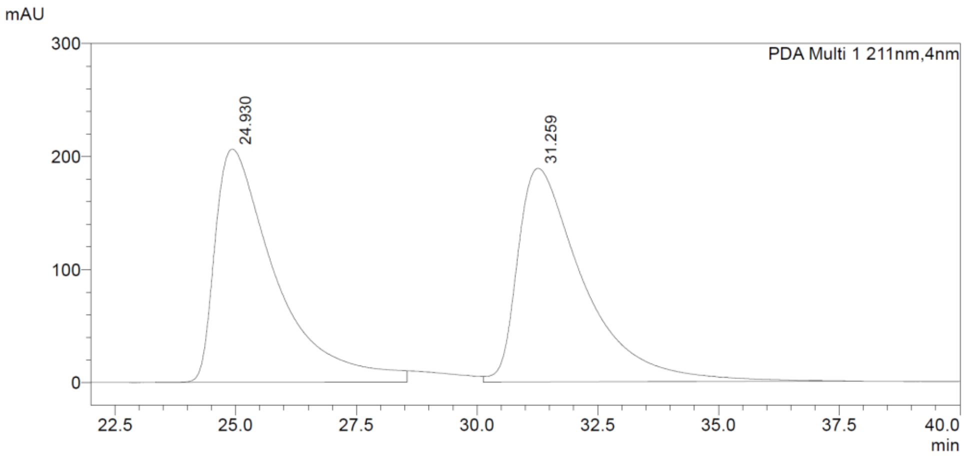

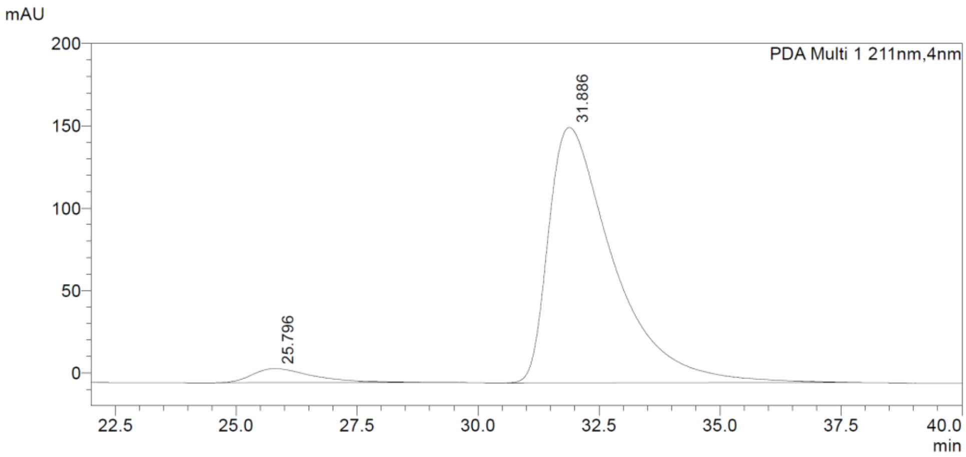

Racemic

PDA Ch1 211nm

Peak#	Ret. Time	Area%
1	24.930	50.054
2	31.259	49.946
Total		100.000

Enantioenriched

PDA Ch1 211nm

Peak#	Ret. Time	Area%
1	25.796	4.876
2	31.886	95.124
Total		100.000

Chiral HPLC analysis, Chiralpak IB (95:5 hexane:^{i}PrOH, flow rate 1 ml·min^{-1}, 211 nm, 30 °C) tR (*R*)-**25**: 17.2 min, tR (*S*)-**25**: 38.5 min, 6:94 er.

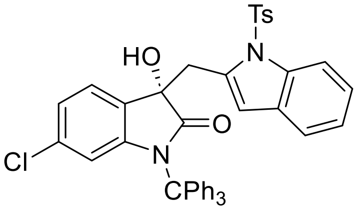

25

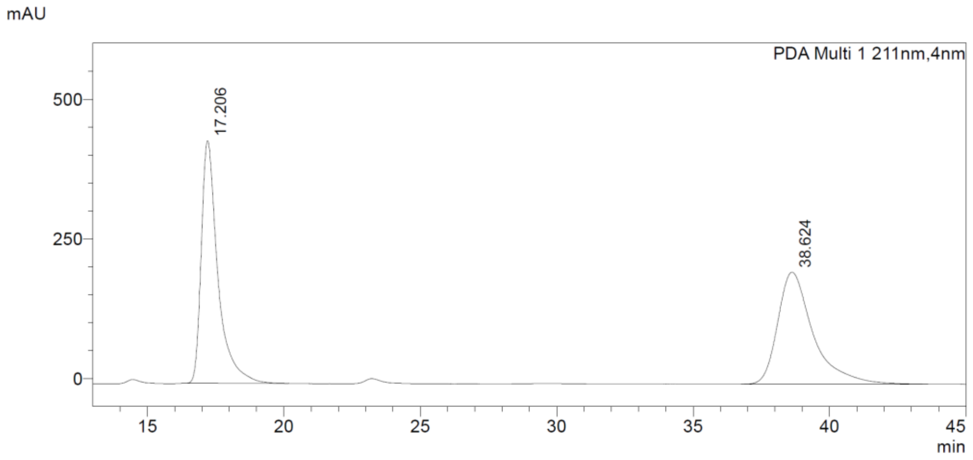

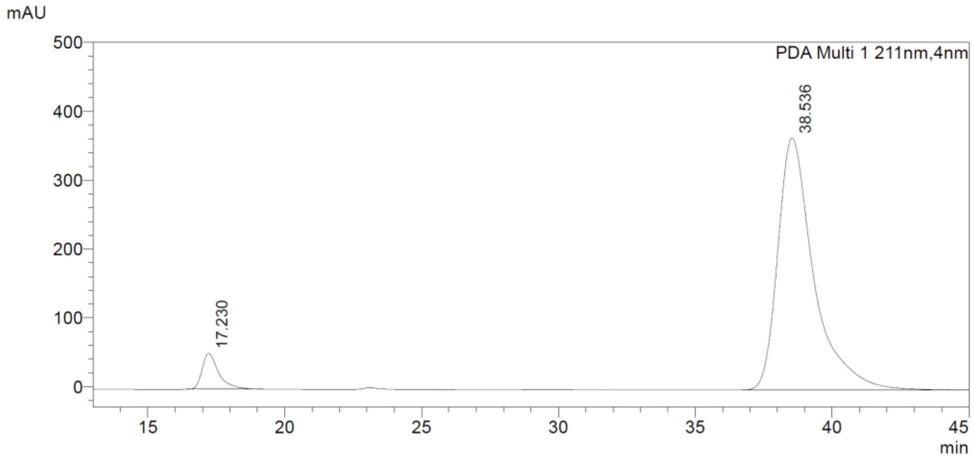

Racemic			Enantioenriched		
PDA Ch1 211nm			PDA Ch1 211nm		
Peak#	Ret. Time	Area%	Peak#	Ret. Time	Area%
1	17.206	50.333	1	17.230	6.175
2	38.624	49.667	2	38.536	93.825
Total		100.000	Total		100.000

Chiral HPLC analysis, Chiralpak OD-H (90:10 hexane:^{i}PrOH, flow rate 1 ml·min^{-1}, 211 nm, 30 °C) tR (*R*)-**26**: 12.0 min, tR (*S*)-**26**: 15.1 min, 5:95 er.

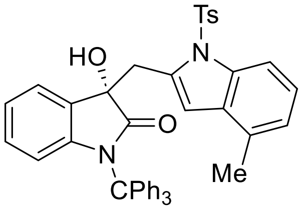

28

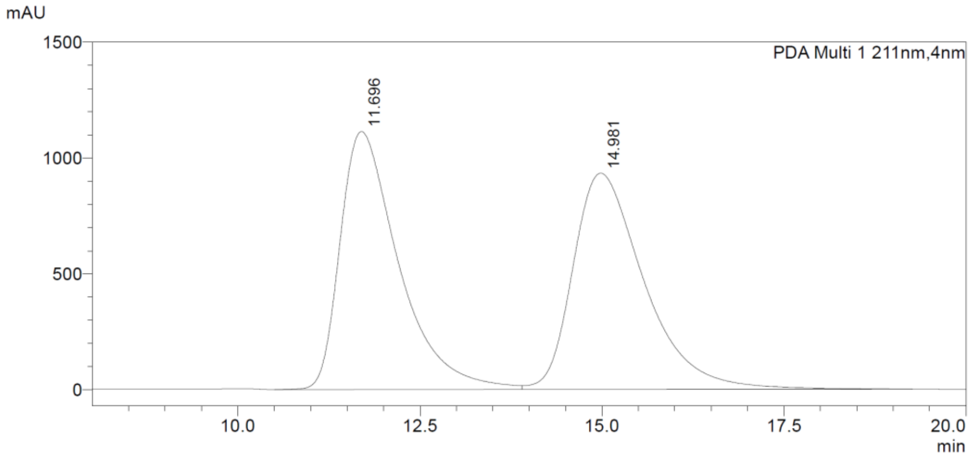

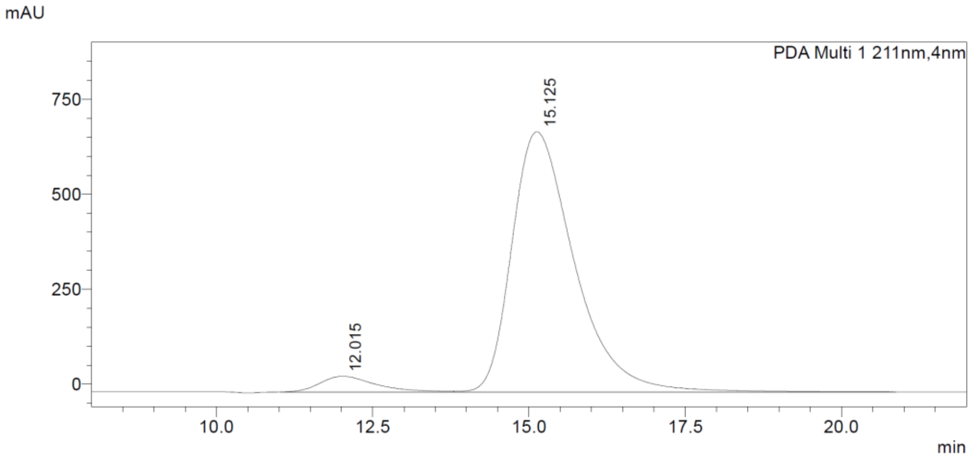

Racemic

PDA Ch1 211nm

Peak#	Ret. Time	Area%
1	11.696	49.515
2	14.981	50.485
Total		100.000

Enantioenriched

PDA Ch1 211nm

Peak#	Ret. Time	Area%
1	12.015	5.067
2	15.125	94.933
Total		100.000

Chiral HPLC analysis, Chiralpak OD-H (90:10 hexane:^{i}PrOH, flow rate 1 ml·min^{-1}, 211 nm, 30 °C) tR (*R*)-**27**: 13.0 min, tR (*S*)-**27**: 16.7 min, 5:95 er.

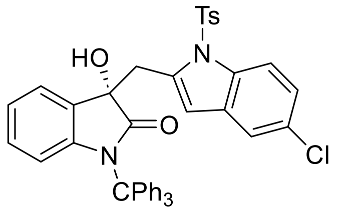

27

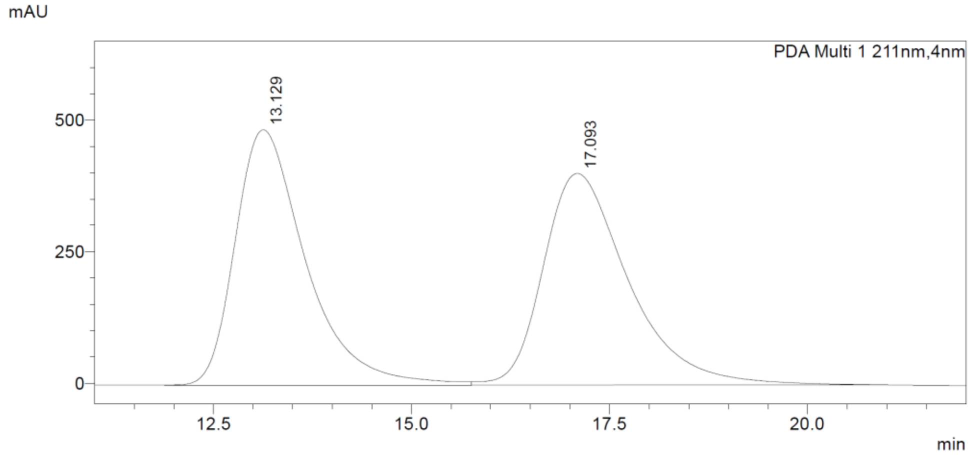

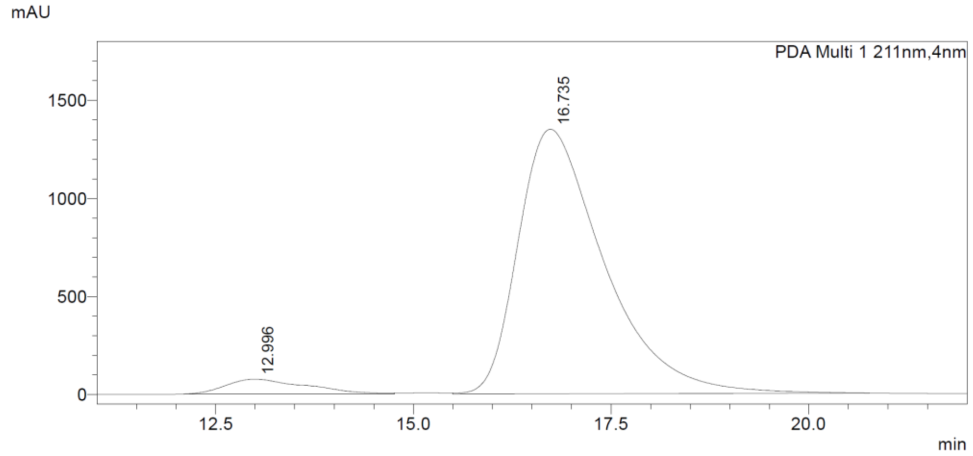

Racemic	Enantioenriched
PDA Ch1 211nm	PDA Ch1 211nm

Racemic — PDA Ch1 211nm

Peak#	Ret. Time	Area%
1	13.129	49.950
2	17.093	50.050
Total		100.000

Enantioenriched — PDA Ch1 211nm

Peak#	Ret. Time	Area%
1	12.996	5.060
2	16.735	94.940
Total		100.000

Chiral HPLC analysis, Chiralpak IA (80:20 hexane:^{i}PrOH, flow rate 1 ml·min^{-1}, 211 nm, 30 °C) tR (*S*)-**28**: 35.0 min, tR (*R*)-**28**: 44.2 min, 96:4 er.

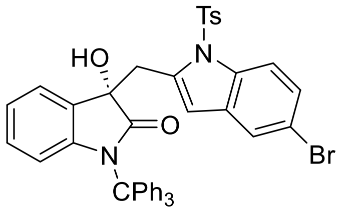

28

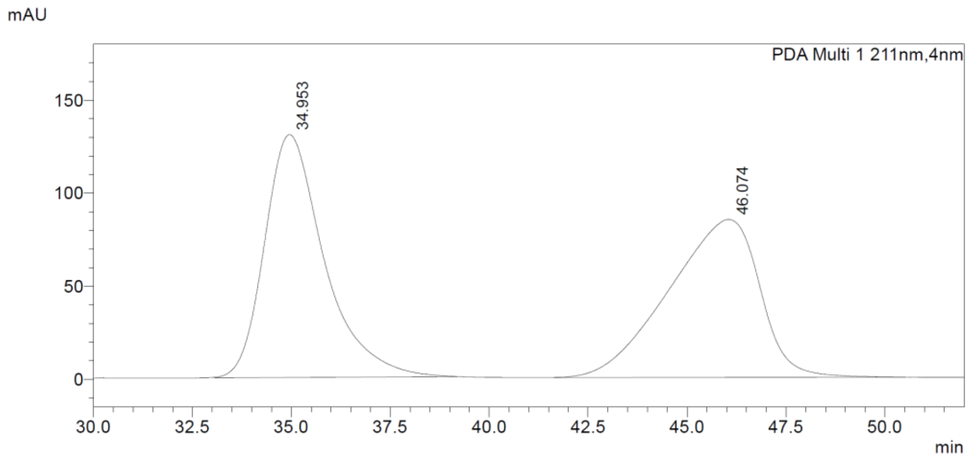

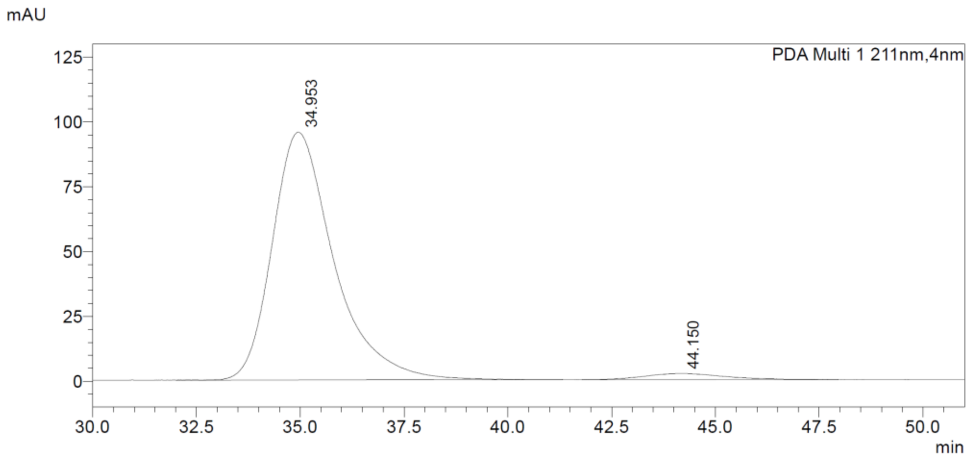

Racemic

PDA Ch1 211nm

Peak#	Ret. Time	Area%
1	34.953	50.173
2	46.074	49.827
Total		100.000

Enantioenriched

PDA Ch1 211nm

Peak#	Ret. Time	Area%
1	34.953	96.907
2	44.150	3.093
Total		100.000

Chiral HPLC analysis, Chiralpak OD-H (90:10 hexane:^{i}PrOH, flow rate 1 ml·min^{-1}, 211 nm, 30 °C) tR (*R*)-**29**: 12.0 min, tR (*S*)-**29**: 16.0 min, 6:94 er.

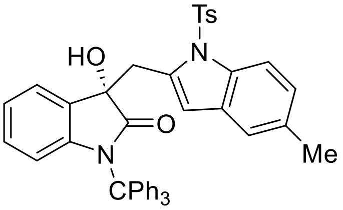

29

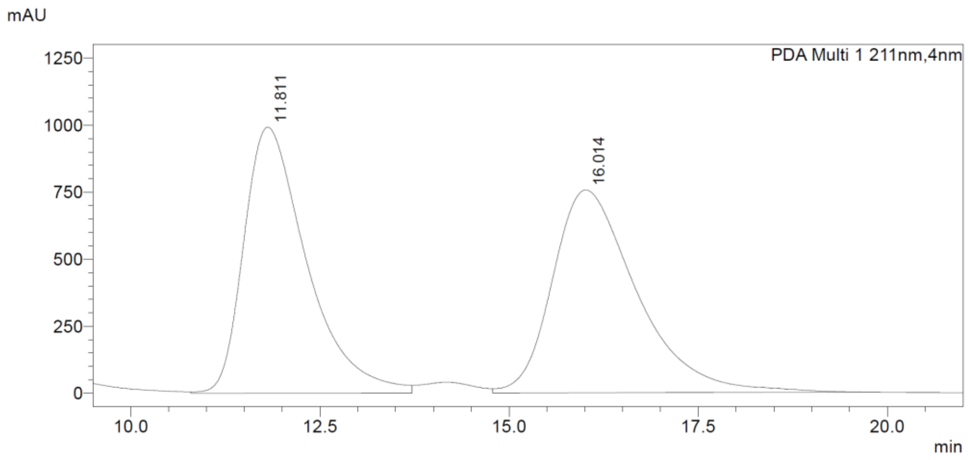

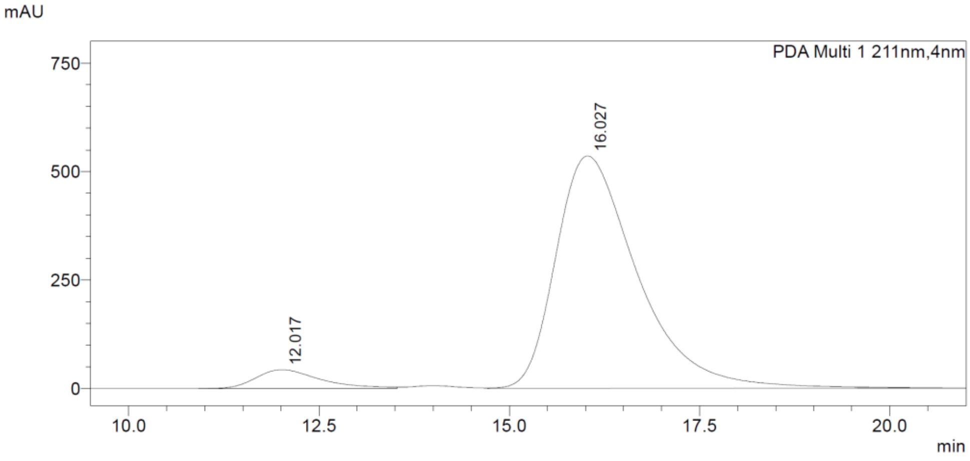

Racemic			Enantioenriched		
PDA Ch1 211nm			PDA Ch1 211nm		
Peak#	Ret. Time	Area%	Peak#	Ret. Time	Area%
1	11.811	49.477	1	12.017	6.030
2	16.014	50.523	2	16.027	93.970
Total		100.000	Total		100.000

Chiral HPLC analysis, Chiralpak OD-H (90:10 hexane:^{i}PrOH, flow rate 1 ml·min^{-1}, 211 nm, 30 °C) tR (*R*)-**30**: 12.0 min, tR (*S*)-**30**: 15.2 min, 7:93 er.

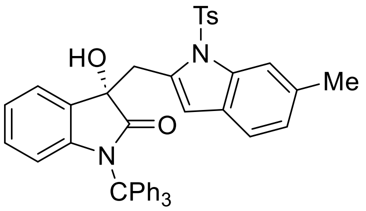

30

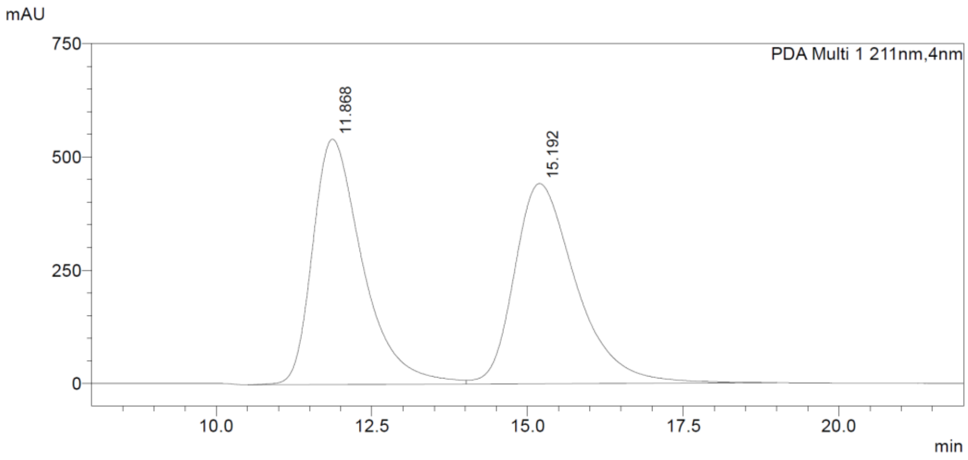

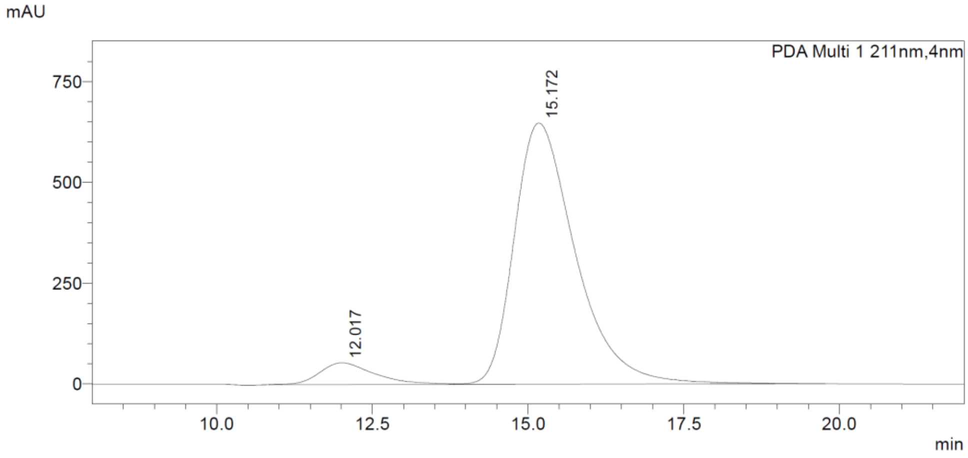

Racemic	Enantioenriched
PDA Ch1 211nm	PDA Ch1 211nm

Racemic:

Peak#	Ret. Time	Area%
1	11.868	49.816
2	15.192	50.184
Total		100.000

Enantioenriched:

Peak#	Ret. Time	Area%
1	12.017	6.934
2	15.172	93.066
Total		100.000

Chiral HPLC analysis, Chiralpak OD-H (90:10 hexane:^{i}PrOH, flow rate 1 ml·min^{-1}, 211 nm, 30 °C) tR (*R*)-**31**: 9.5 min, tR (*S*)-**31**: 15.8 min, 3.5:96.5 er.

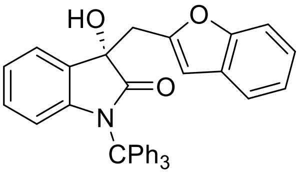

31

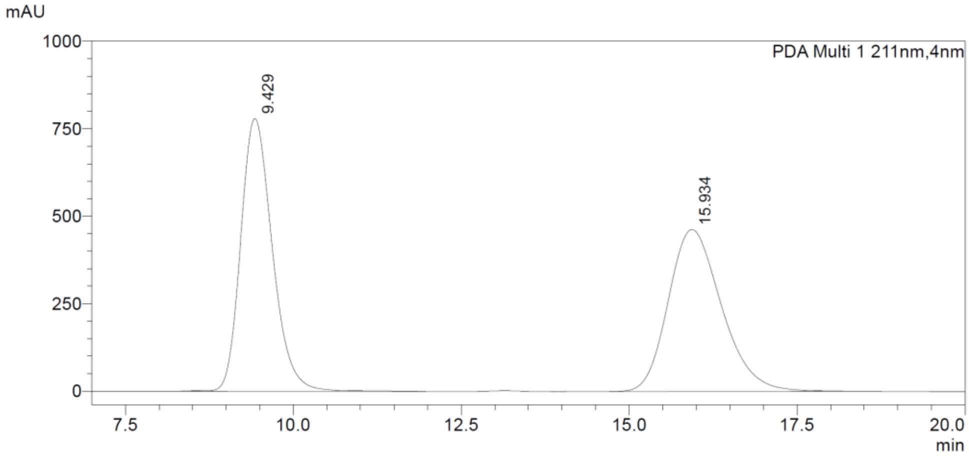

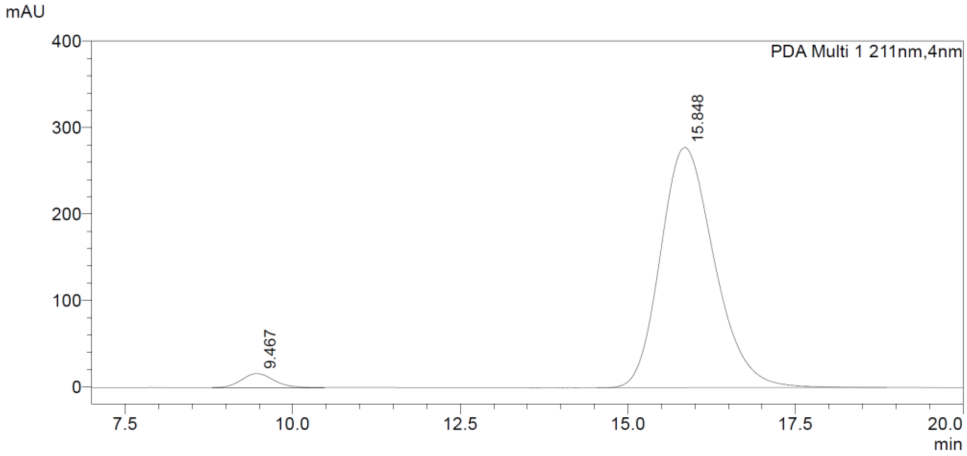

Racemic

PDA Ch1 211nm

Peak#	Ret. Time	Area%
1	9.429	50.064
2	15.934	49.936
Total		100.000

Enantioenriched

PDA Ch1 211nm

Peak#	Ret. Time	Area%
1	9.467	3.370
2	15.848	96.630
Total		100.000

Chiral HPLC analysis, Chiralpak OD-H (90:10 hexane:^{i}PrOH, flow rate 1 ml·min^{-1}, 211 nm, 30 °C) tR (*R*)-**32**: 10.7 min, tR (*S*)-**32**: 13.5 min, 9:91 er.

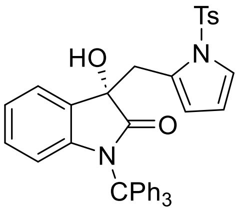

32

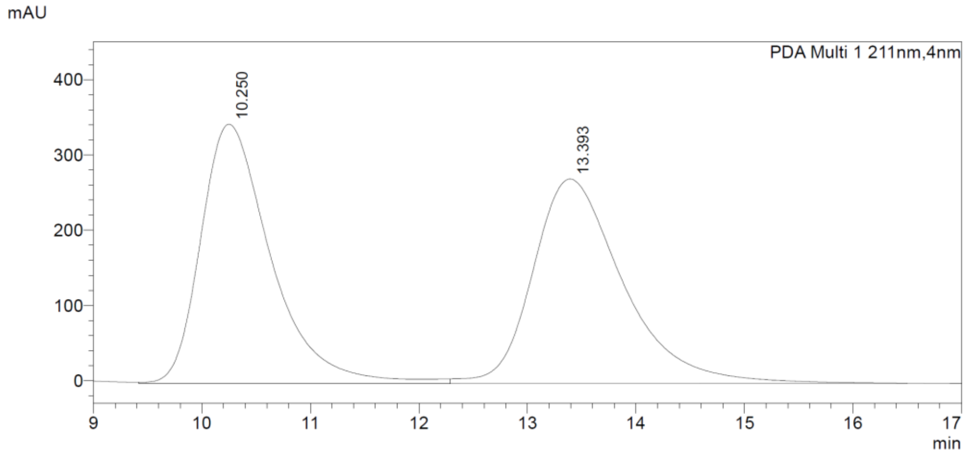

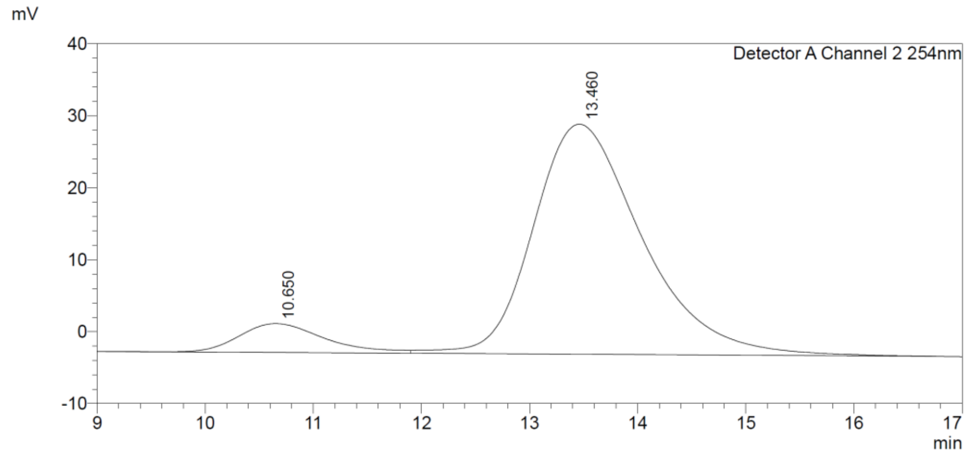

Racemic			Enantioenriched		
PDA Ch1 211nm			Detector A Channel 2 254nm		
Peak#	Ret. Time	Area%	Peak#	Ret. Time	Area%
1	10.250	49.681	1	10.650	8.993
2	13.393	50.319	2	13.460	91.007
Total		100.000	Total		100.000

Chiral HPLC analysis, Chiralpak IB (95:5 hexane: ^{i}PrOH, flow rate 1 ml·min^{-1}, 211 nm, 30 °C) tR (*S*)-**33**: 20.1 min, tR (*R*)-**33**: 32.3 min, 92:8 er.

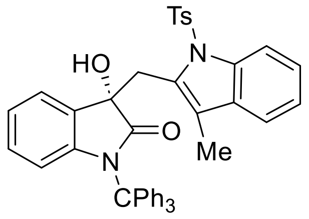

33

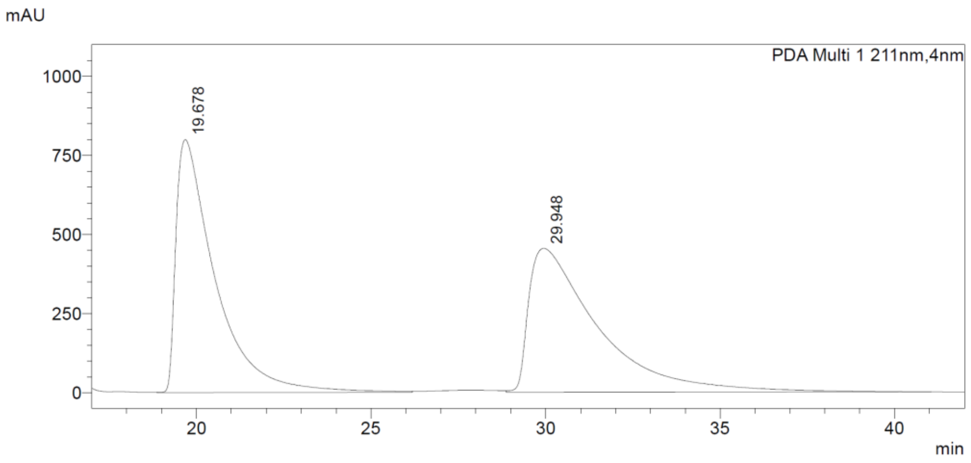

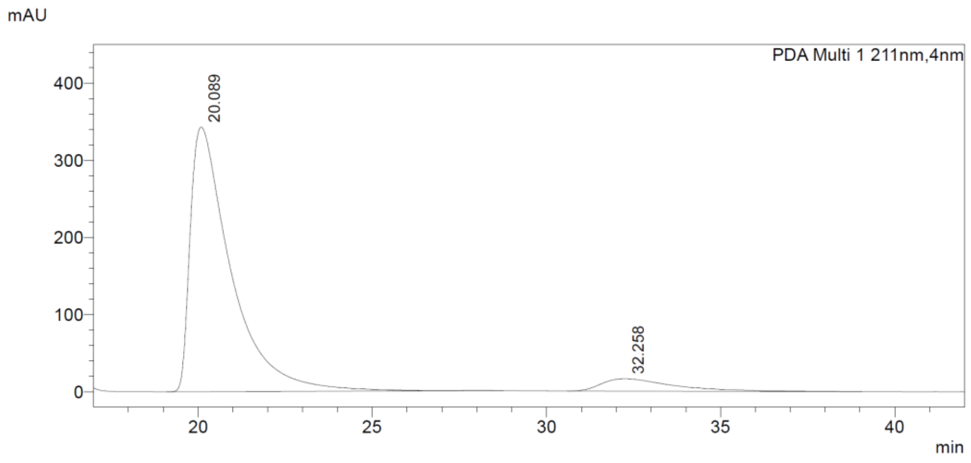

Racemic			Enantioenriched		
PDA Ch1 211nm			PDA Ch1 211nm		
Peak#	Ret. Time	Area%	Peak#	Ret. Time	Area%
1	19.678	49.776	1	20.089	92.273
2	29.948	50.224	2	32.258	7.727
Total		100.000	Total		100.000

Chiral HPLC analysis, Chiralpak IA (70:30 hexane:^{i}PrOH, flow rate 2 ml·min^{-1}, 211 nm, 30 °C) tR (*R*)-**34**: 14.7 min, tR (*S*)-**34**: 29.5 min, 4:96 er.

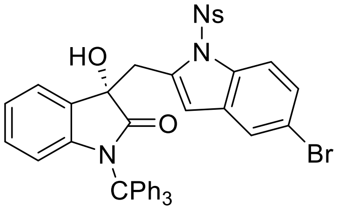

34

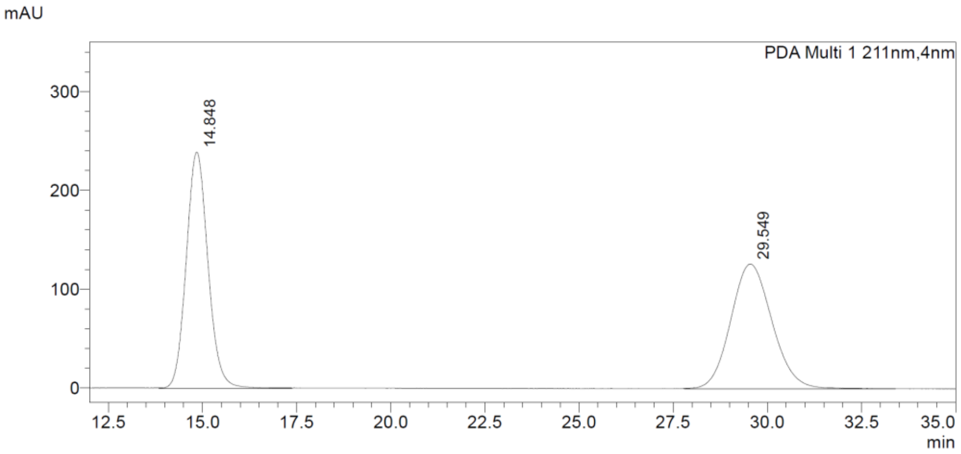

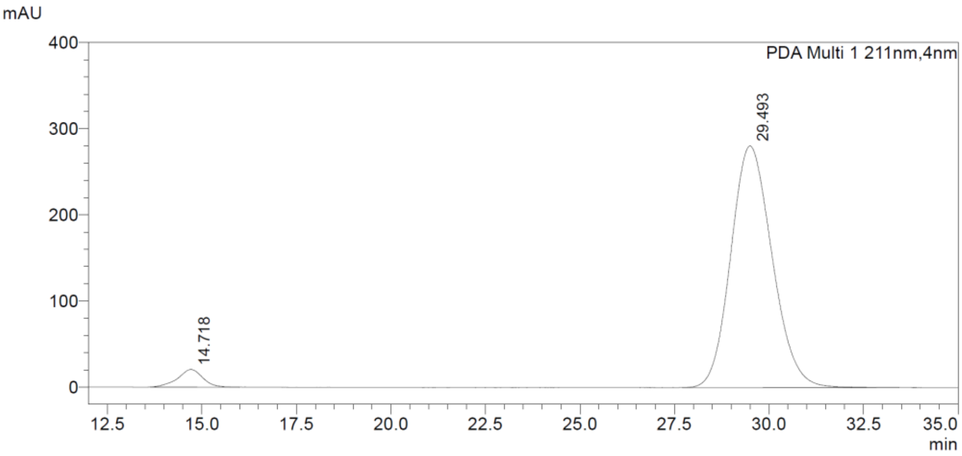

Racemic

PDA Ch1 211nm

Peak#	Ret. Time	Area%
1	14.848	49.902
2	29.549	50.098
Total		100.000

Enantioenriched

PDA Ch1 211nm

Peak#	Ret. Time	Area%
1	14.718	4.223
2	29.493	95.777
Total		100.000

Chiral HPLC analysis, Chiralpak IA (70:30 hexane:^{i}PrOH, flow rate 1 ml·min^{-1}, 211 nm, 30 °C) tR (*S*)-**35**: 11.3 min, tR (*R*)-**35**: 13.1 min, 92:8 er.

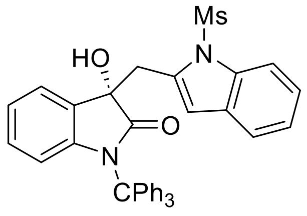

35

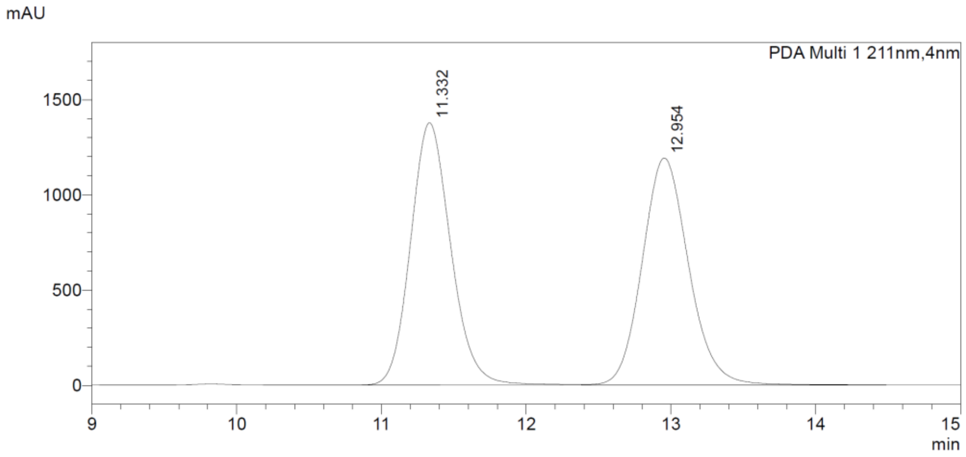

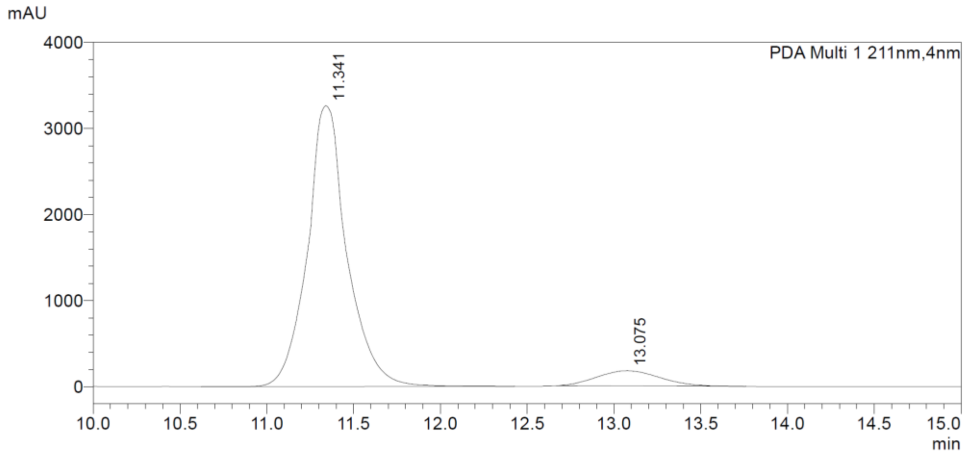

Racemic

PDA Ch1 211nm

Peak#	Ret. Time	Area%
1	11.332	50.076
2	12.954	49.924
Total		100.000

Enantioenriched

PDA Ch1 211nm

Peak#	Ret. Time	Area%
1	11.341	92.017
2	13.075	7.983
Total		100.000

Chiral HPLC analysis, Chiralpak OD-H (90:10 hexane:^{i}PrOH, flow rate 1 ml·min^{-1}, 211 nm, 30 °C) tR (*S*)-**4**: 7.4 min, tR (*R*)-**4**: 10.4 min, 3:97 er; **IR** ν_{max} (film) 1732 (C=O).

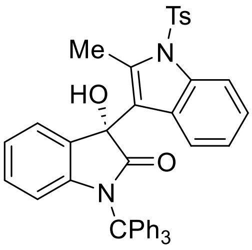

4

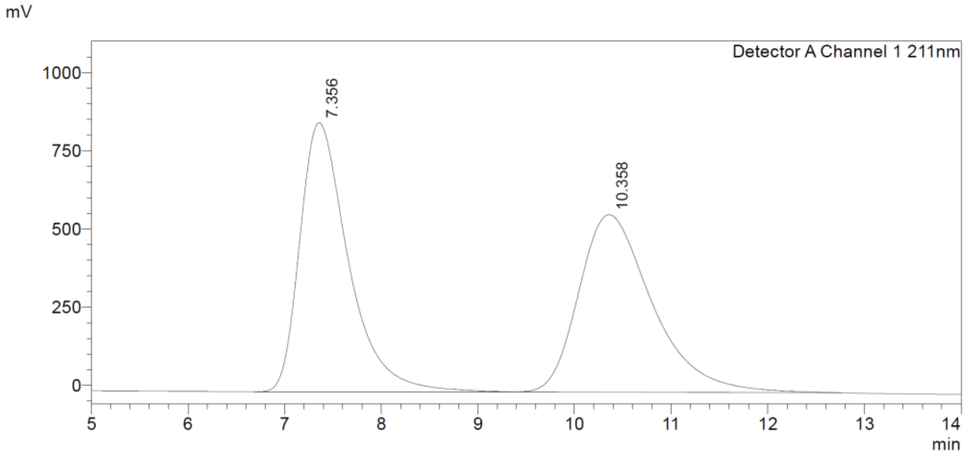

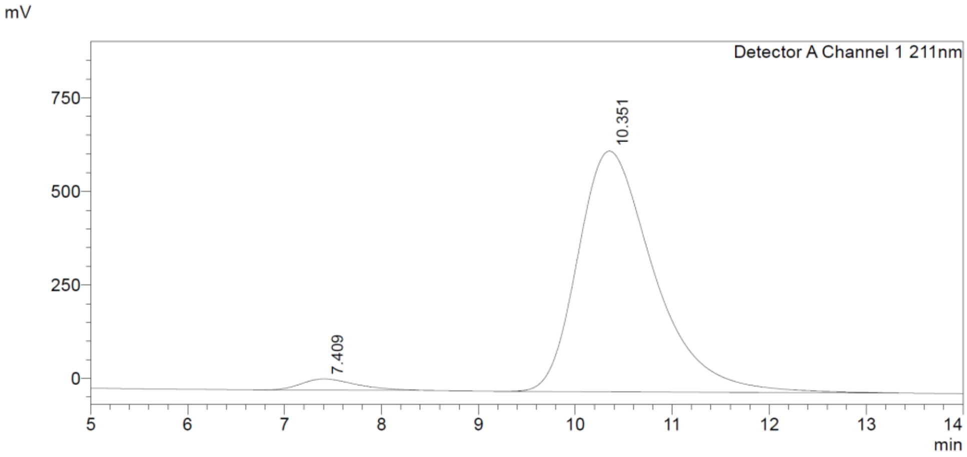

Racemic			Enantioenriched		
Detector A Channel 1 211nm			Detector A Channel 1 211nm		
Peak#	Ret. Time	Area%	Peak#	Ret. Time	Area%
1	7.356	50.023	1	7.409	3.129
2	10.358	49.977	2	10.351	96.871
Total		100.000	Total		100.000

Chiral HPLC analysis, Chiralpak OD-H (95:5 hexane:^{i}PrOH, flow rate 1 ml·min^{-1}, 211 nm, 30 °C) tR (*S*)-**36**: 10.1 min, tR (*R*)-**36**: 13.3 min, 2:98 er.

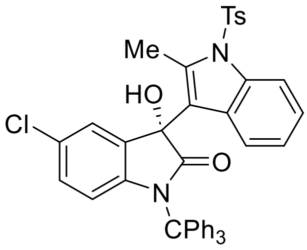

36

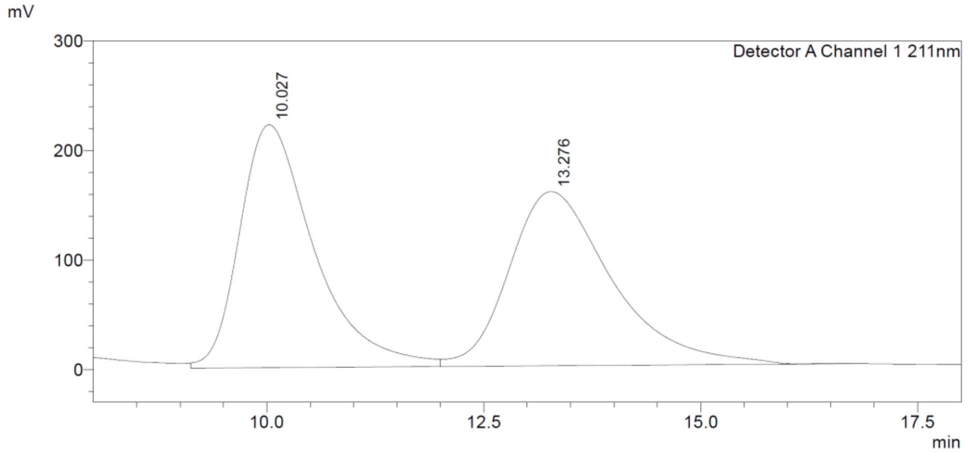

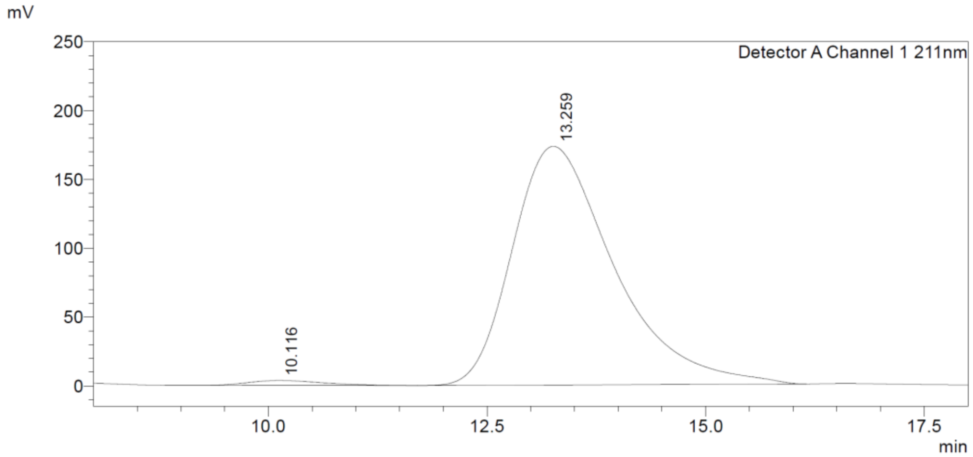

Racemic	Enantioenriched
Detector A Channel 1 211nm	Detector A Channel 1 211nm

Racemic — Detector A Channel 1 211nm

Peak#	Ret. Time	Area%
1	10.027	50.256
2	13.276	49.744
Total		100.000

Enantioenriched — Detector A Channel 1 211nm

Peak#	Ret. Time	Area%
1	10.116	1.509
2	13.259	98.491
Total		100.000

Chiral HPLC analysis, Chiralpak IA (85:15 hexane:^{i}PrOH, flow rate 1 ml·min^{-1}, 211 nm, 30 °C) tR (*S*)-**37**: 11.7 min, tR (*R*)-**37**: 15.6 min, 5:95 er.

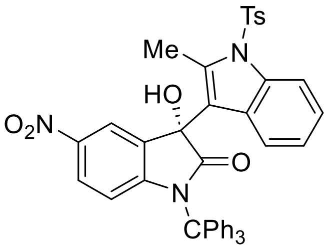

37

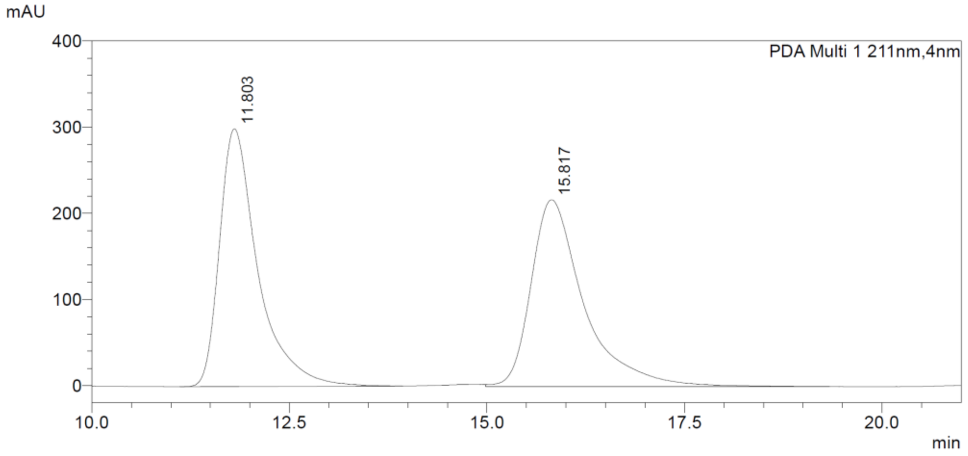

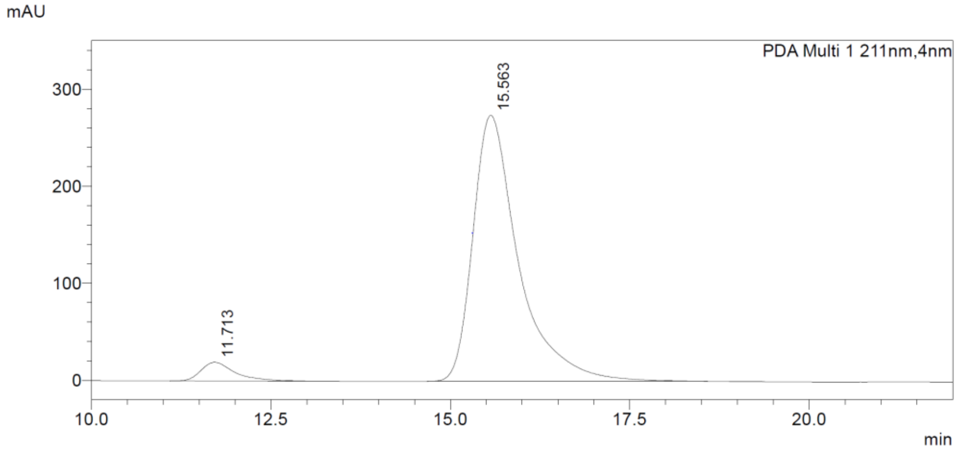

Racemic	Enantioenriched

Racemic — PDA Ch1 211nm

Peak#	Ret. Time	Area%
1	11.803	49.948
2	15.817	50.052
Total		100.000

Enantioenriched — PDA Ch1 211nm

Peak#	Ret. Time	Area%
1	11.713	4.994
2	15.563	95.006
Total		100.000

Chiral HPLC analysis, Chiralpak OD-H (90:10 hexane:^{i}PrOH, flow rate 1 ml·min^{-1}, 211 nm, 30 °C) tR (*S*)-**38**: 7.0 min, tR (*R*)-**38**: 8.6 min, 4:96 er.

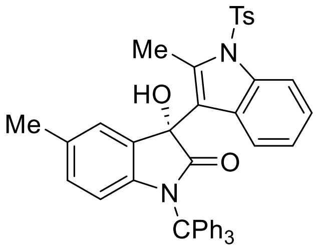

38

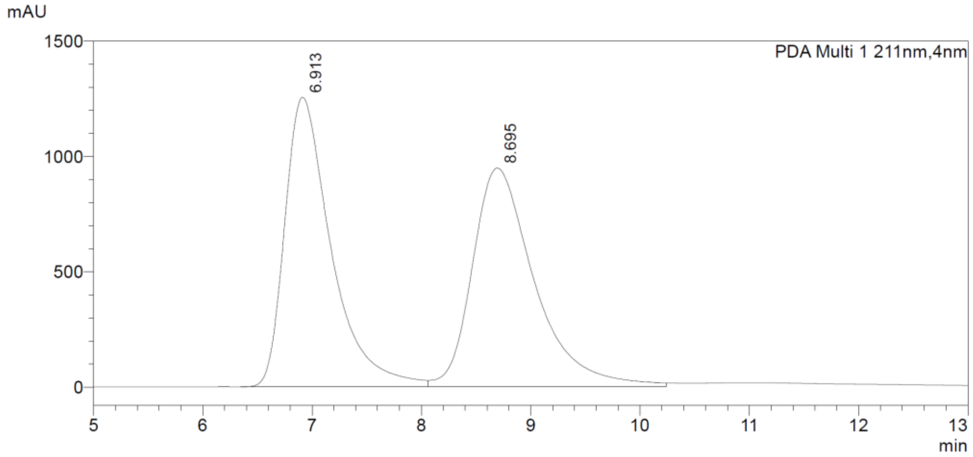

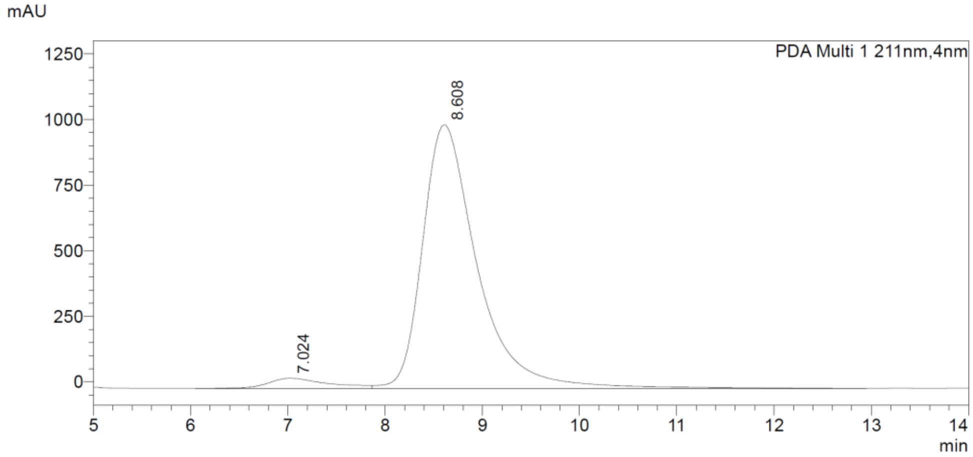

Racemic

PDA Ch1 211nm

Peak#	Ret. Time	Area%
1	6.913	49.682
2	8.695	50.318
Total		100.000

Enantioenriched

PDA Ch1 211nm

Peak#	Ret. Time	Area%
1	7.024	4.105
2	8.608	95.895
Total		100.000

Chiral HPLC analysis, Chiralpak AD-H (80:20 hexane:^{i}PrOH, flow rate 1 ml·min^{-1}, 211 nm, 30 °C) tR (*R*)-**39**: 8.7 min, tR (*S*)-**39**: 10.5 min, 98:2 er.

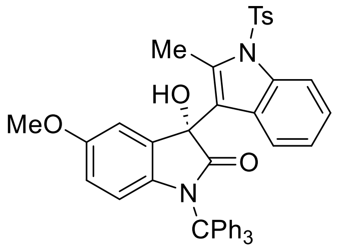

39

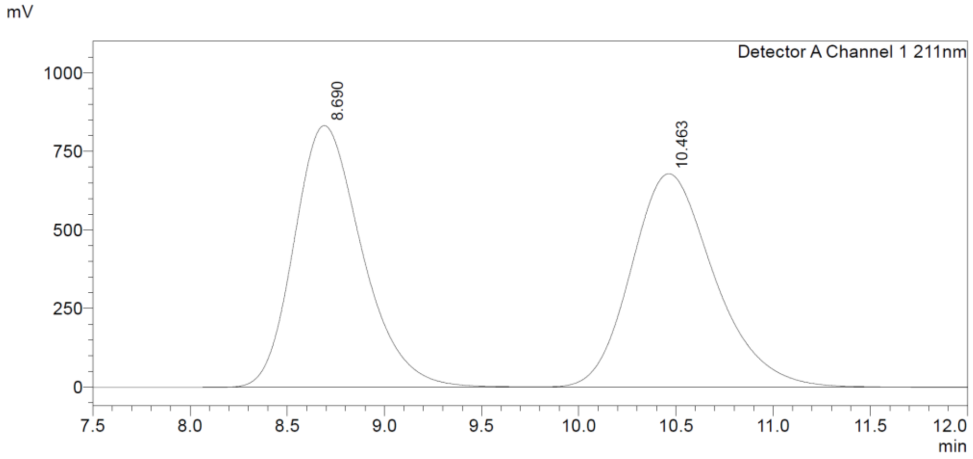

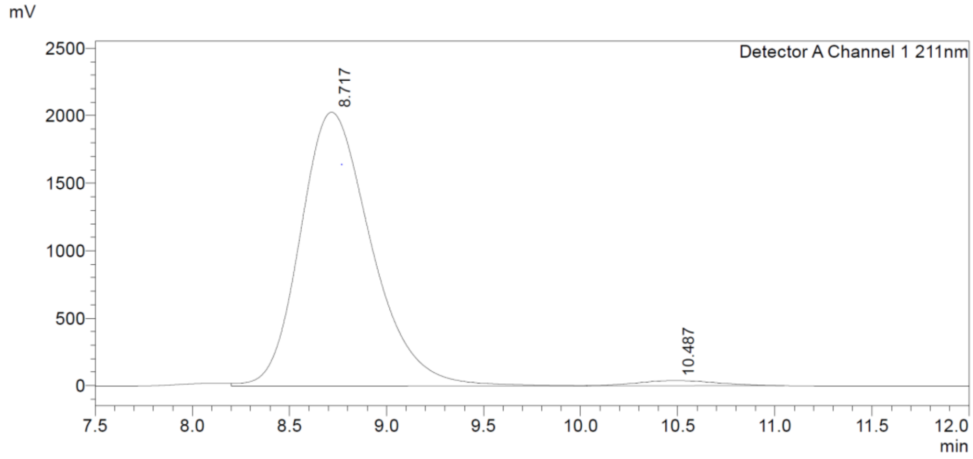

Racemic

Detector A Channel 1 211nm

Peak#	Ret. Time	Area%
1	8.690	49.973
2	10.463	50.027
Total		100.000

Enantioenriched

Detector A Channel 1 211nm

Peak#	Ret. Time	Area%
1	8.717	97.927
2	10.487	2.073
Total		100.000

Chiral HPLC analysis, Chiralpak OD-H (90:10 hexane:^{i}PrOH, flow rate 1 ml·min^{-1}, 211 nm, 30 °C) tR (*S*)-**40**: 7.3 min, tR (*R*)-**40**: 11.1 min, 3:97 er.

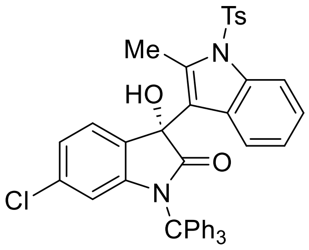

40

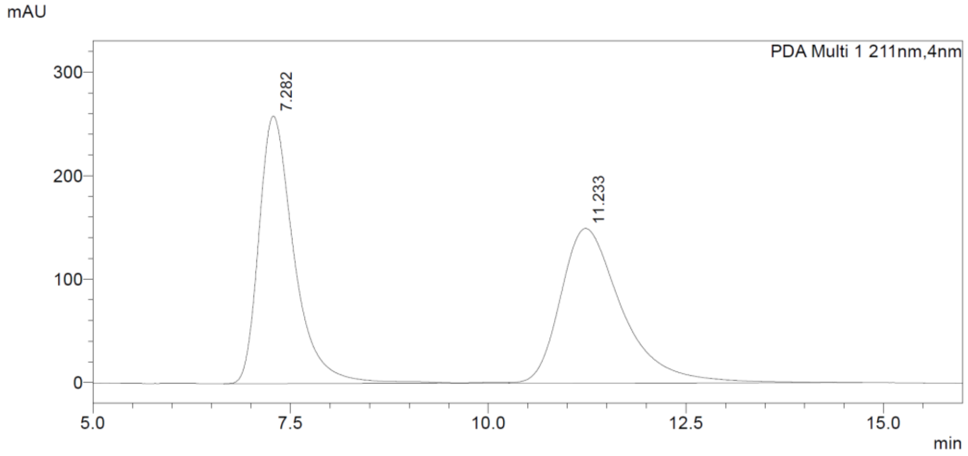

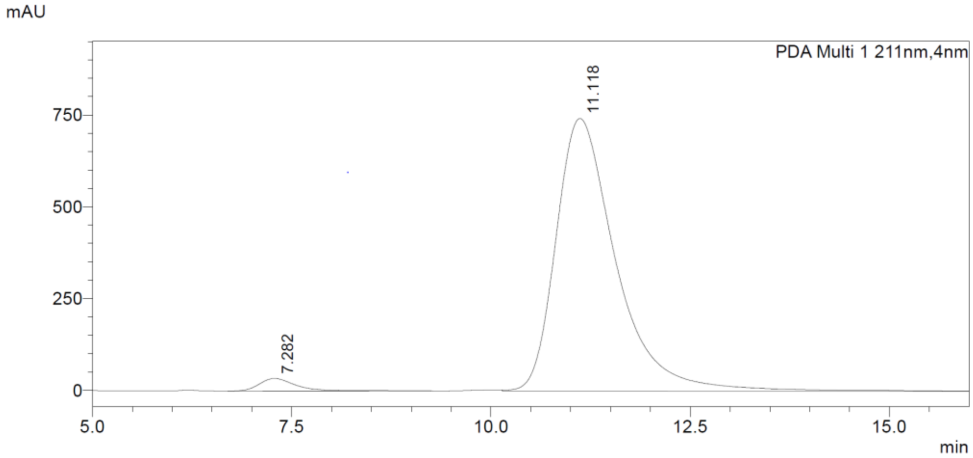

Racemic			Enantioenriched		
PDA Ch1 211nm			PDA Ch1 211nm		
Peak#	Ret. Time	Area%	Peak#	Ret. Time	Area%
1	7.282	49.933	1	7.282	2.722
2	11.233	50.067	2	11.118	97.278
Total		100.000	Total		100.000

Chiral HPLC analysis, Chiralpak IB (95:5 hexane:^{i}PrOH, flow rate 1 ml·min^{-1}, 211 nm, 30 °C) tR (*S*)-**41**: 13.5 min, tR (*R*)-**41**: 15.6 min, 3:97 er.

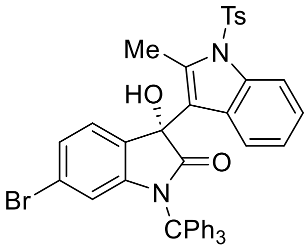

41

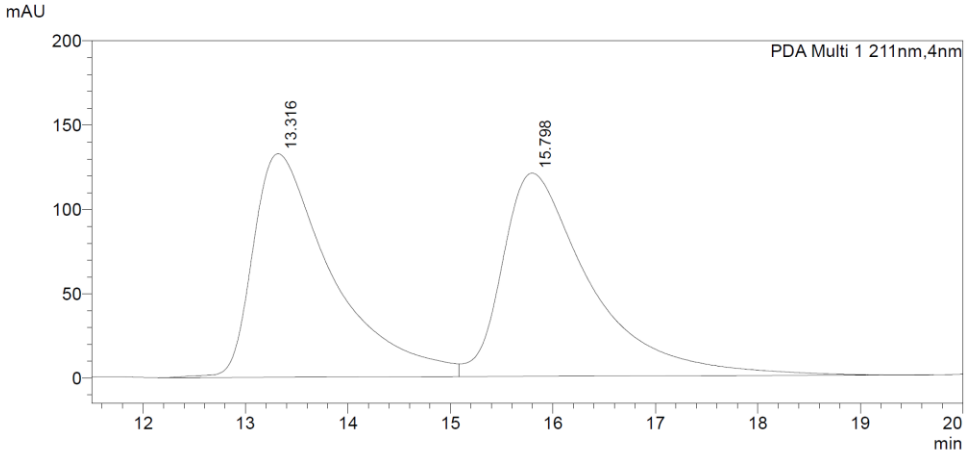

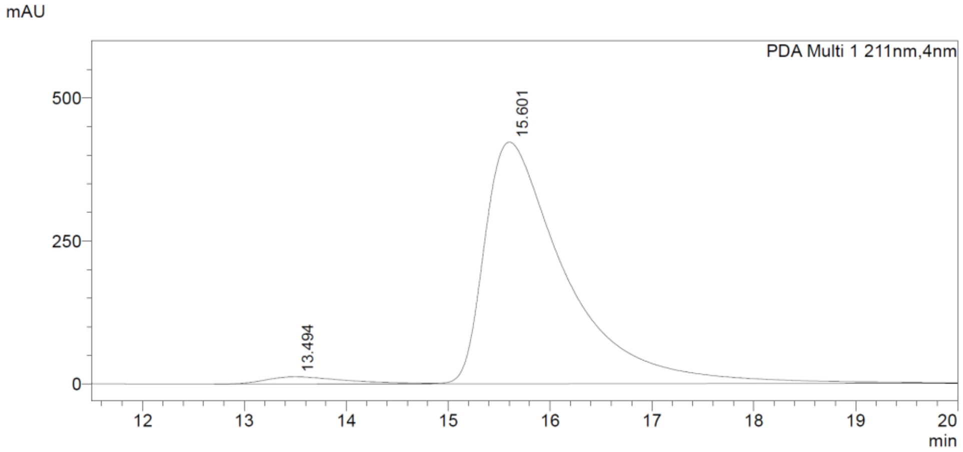

Racemic	Enantioenriched

Racemic — PDA Ch1 211nm

Peak#	Ret. Time	Area%
1	13.316	49.498
2	15.798	50.502
Total		100.000

Enantioenriched — PDA Ch1 211nm

Peak#	Ret. Time	Area%
1	13.494	2.835
2	15.601	97.165
Total		100.000

Chiral HPLC analysis, Chiralpak OD-H (90:10 hexane:^{i}PrOH, flow rate 1 ml·min^{-1}, 211 nm, 30 °C) tR (*S*)-**42**: 6.6 min, tR (*R*)-**42**: 10.8 min, 3:97 er.

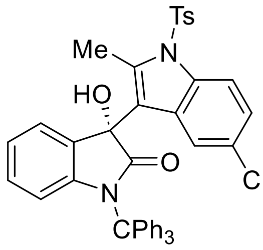

42

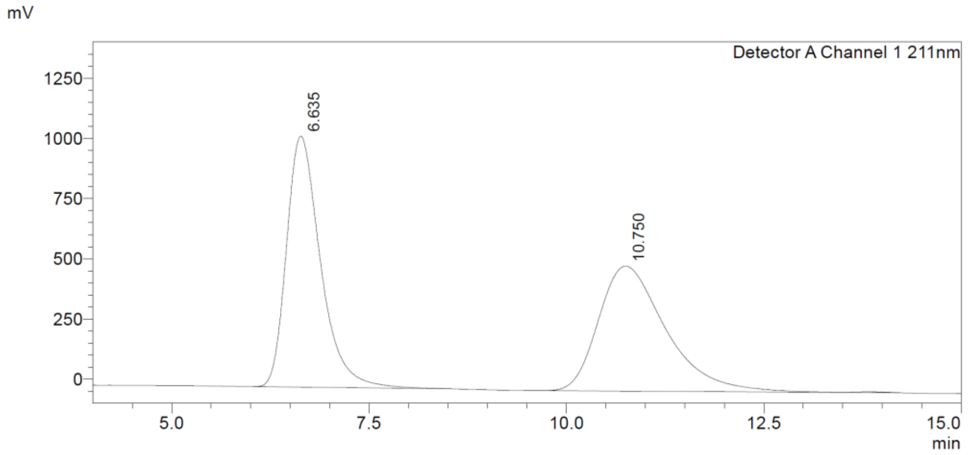

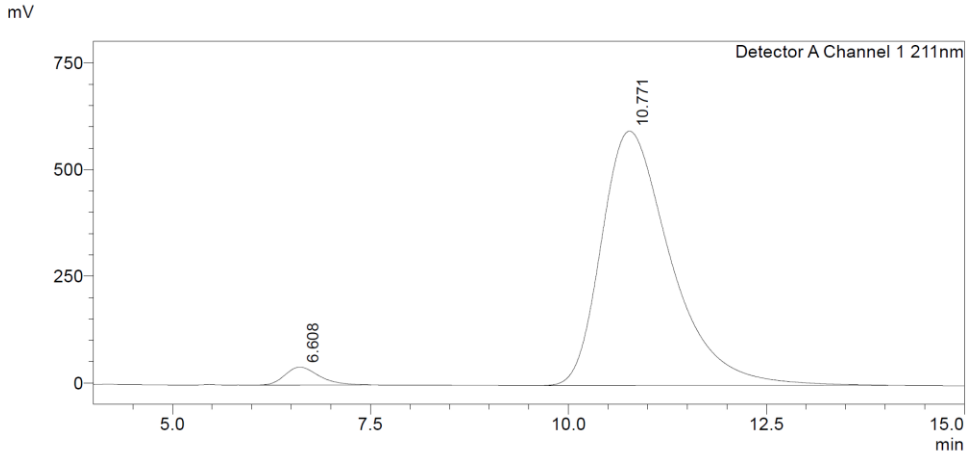

Racemic

Detector A Channel 1 211nm

Peak#	Ret. Time	Area%
1	6.635	50.293
2	10.750	49.707
Total		100.000

Enantioenriched

Detector A Channel 1 211nm

Peak#	Ret. Time	Area%
1	6.608	3.314
2	10.771	96.686
Total		100.000

Chiral HPLC analysis, Chiralpak OD-H (90:10 hexane:^{i}PrOH, flow rate 1 ml·min^{-1}, 211 nm, 30 °C) tR (*S*)-**43**: 7.0 min, tR (*R*)-**43**: 12.4 min, 2:98 er.

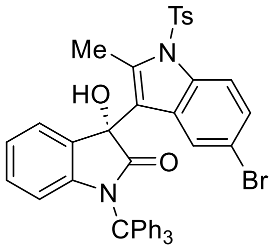

43

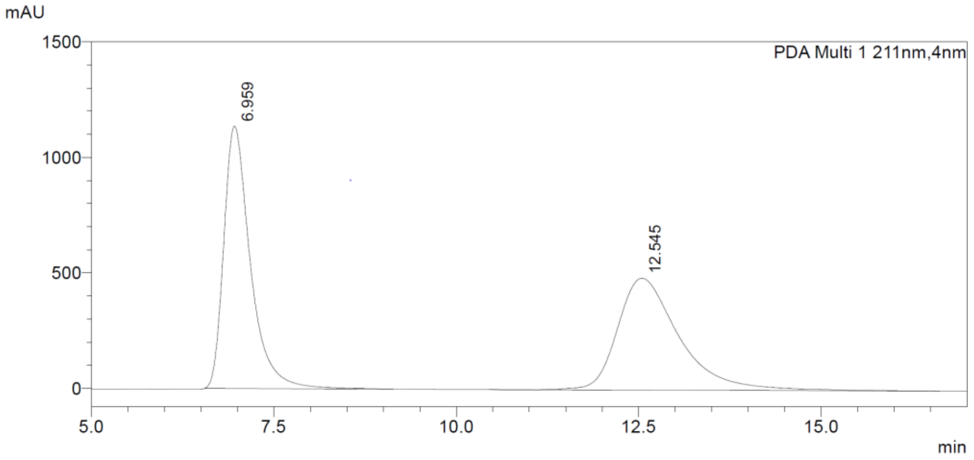

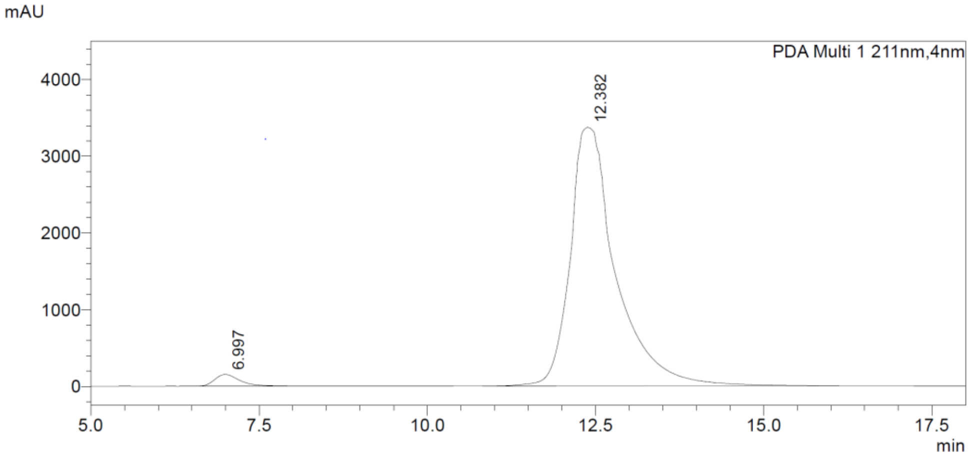

Racemic			Enantioenriched		
PDA Ch1 211nm			PDA Ch1 211nm		
Peak#	Ret. Time	Area%	Peak#	Ret. Time	Area%
1	6.959	50.255	1	6.997	2.389
2	12.545	49.745	2	12.382	97.611
Total		100.000	Total		100.000

Chiral HPLC analysis, Chiralpak OD-H (90:10 hexane:^{i}PrOH, flow rate 1 ml·min^{-1}, 211 nm, 30 °C) tR (*S*)-**44**: 7.4 min, tR (*R*)-**44**: 11.6 min, 2:98 er.

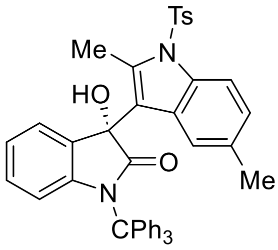

44

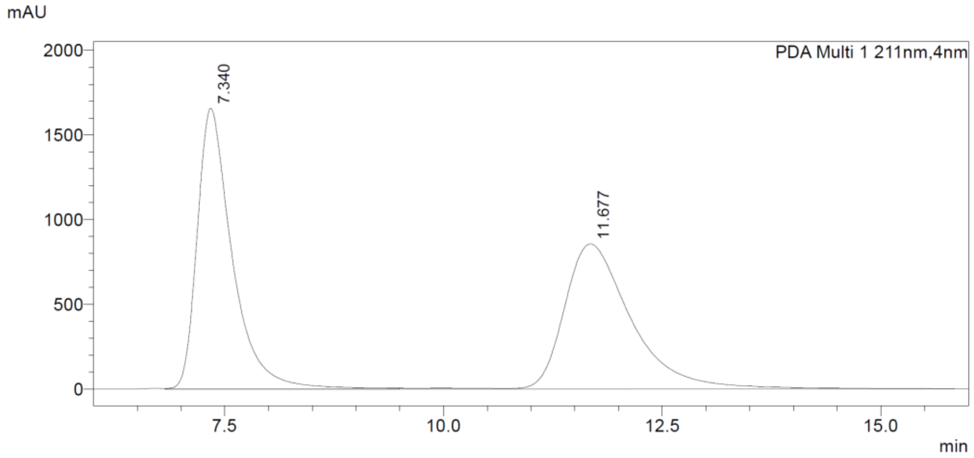

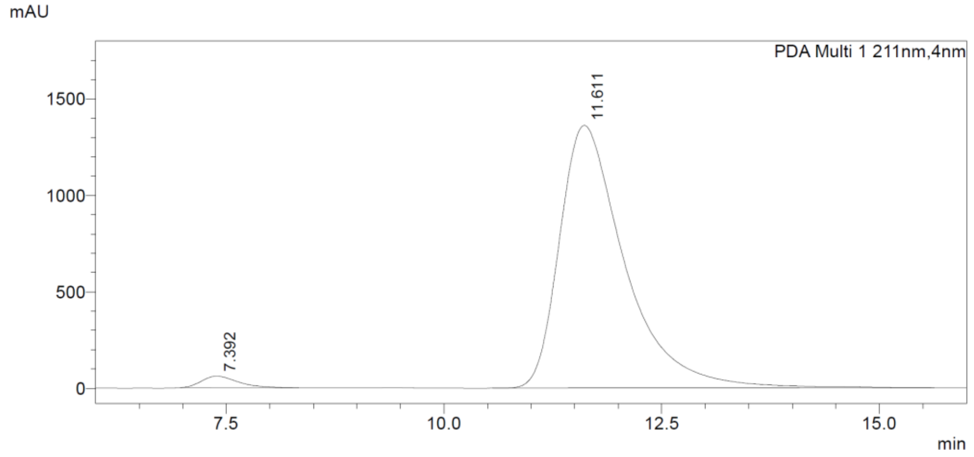

Racemic

PDA Ch1 211nm

Peak#	Ret. Time	Area%
1	7.340	50.368
2	11.677	49.632
Total		100.000

Enantioenriched

PDA Ch1 211nm

Peak#	Ret. Time	Area%
1	7.392	2.375
2	11.611	97.625
Total		100.000

Chiral HPLC analysis, Chiralpak IA (95:5 hexane:^{i}PrOH, flow rate 1 ml·min^{-1}, 211 nm, 30 °C) tR (*R*)-**45**: 18.1 min, tR (*S*)-**45**: 24.5 min, 98:2 er.

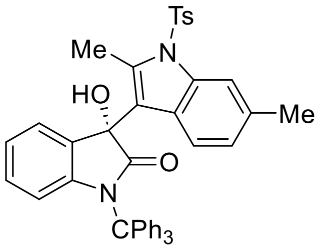

45

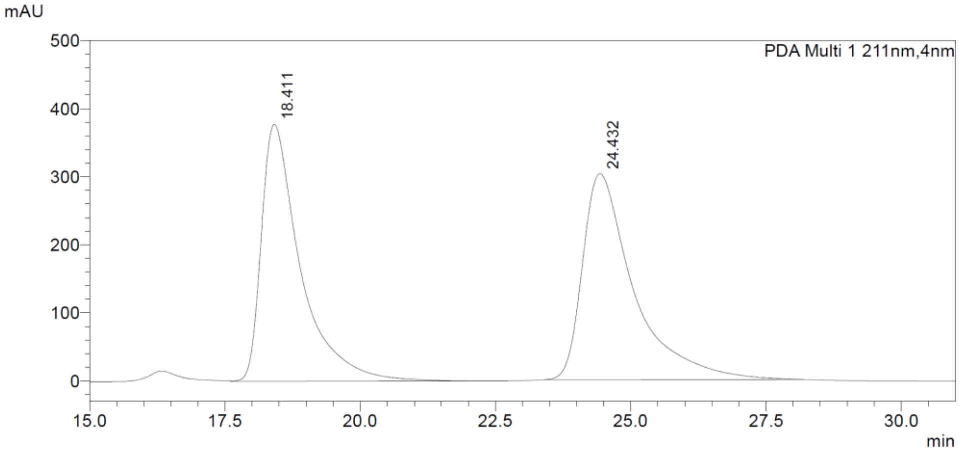

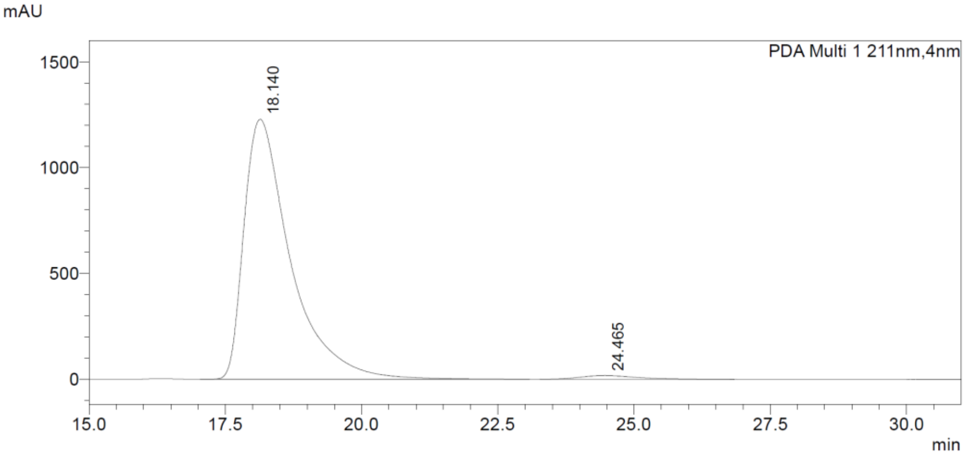

Racemic

PDA Ch1 211nm

Peak#	Ret. Time	Area%
1	18.411	48.696
2	24.432	51.304
Total		100.000

Enantioenriched

PDA Ch1 211nm

Peak#	Ret. Time	Area%
1	18.140	98.286
2	24.465	1.714
Total		100.000

Chiral HPLC analysis, Chiralpak OD-H (95:5 hexane:^{i}PrOH, flow rate 1 ml·min^{-1}, 211 nm, 30 °C) tR (*R*)-**46**: 7.3 min, tR (*S*)-**46**: 9.1 min, 94:6 er.

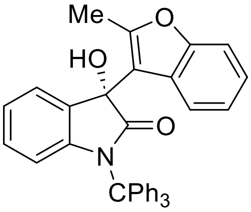

46

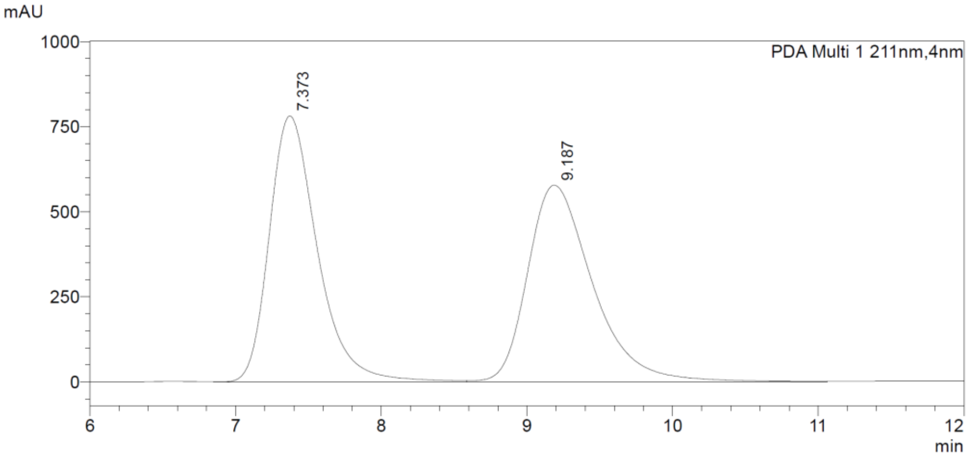

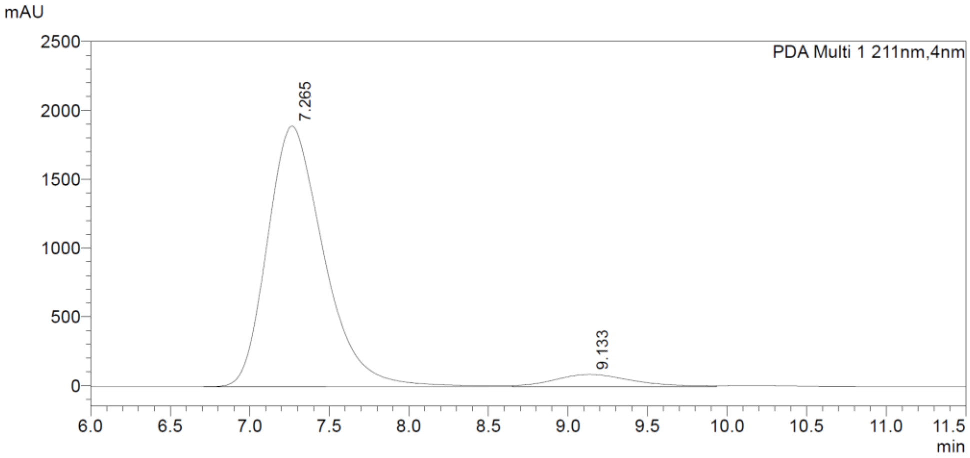

Racemic

PDA Ch1 211nm

Peak#	Ret. Time	Area%
1	7.373	49.927
2	9.187	50.073
Total		100.000

Enantioenriched

PDA Ch1 211nm

Peak#	Ret. Time	Area%
1	7.265	93.851
2	9.133	6.149
Total		100.000

Chiral HPLC analysis, **Chiral HPLC analysis**, Chiralpak AD-H (80:20 hexane:^{i}PrOH, flow rate 1 ml·min^{-1}, 211 nm, 30 °C) tR (*R*)-**47**: 7.4 min, tR (*S*)-**47**: 14.0 min, 96:4 er.

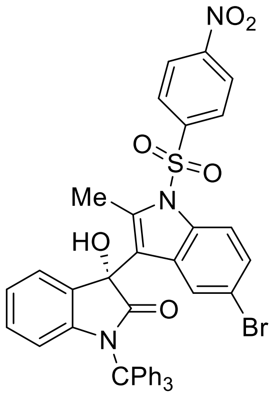

47

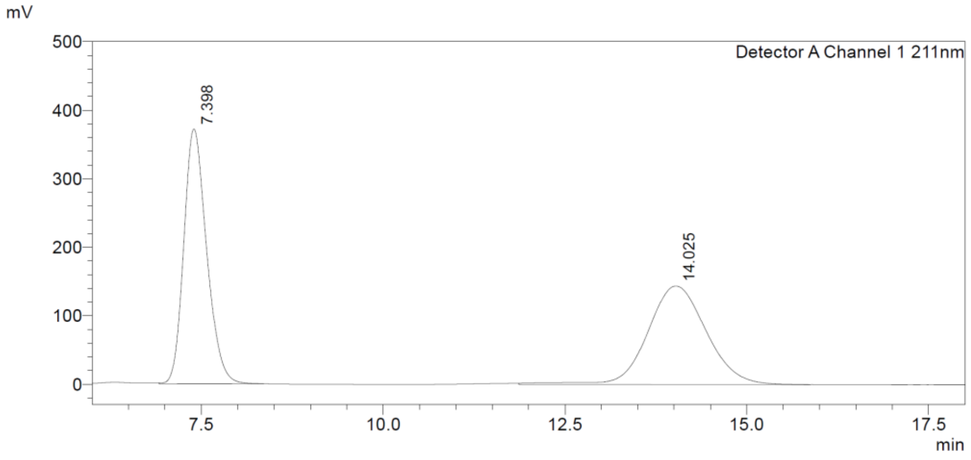

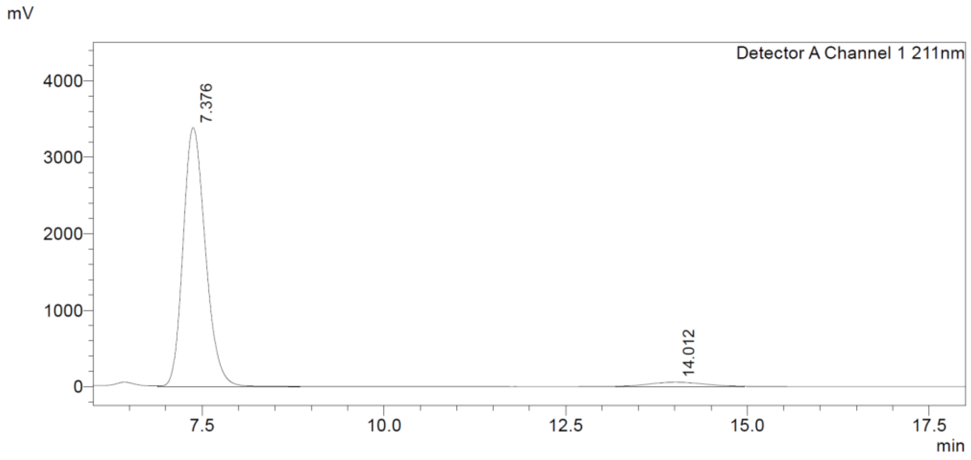

Racemic	Enantioenriched

Racemic — Detector A Channel 1 211nm

Peak#	Ret. Time	Area%
1	7.398	50.123
2	14.025	49.877
Total		100.000

Enantioenriched — Detector A Channel 1 211nm

Peak#	Ret. Time	Area%
1	7.376	95.958
2	14.012	4.042
Total		100.000

Chiral HPLC analysis, Chiralpak AD-H (80:20 hexane:^{i}PrOH, flow rate 1 ml·min^{-1}, 211 nm, 30 °C) tR (*R*)-**48**: 45.1 min, tR (*S*)-**48**: 50.6 min, 90:10 er.

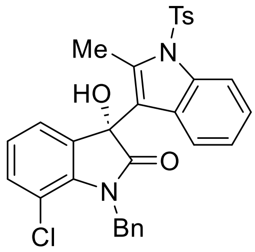

48

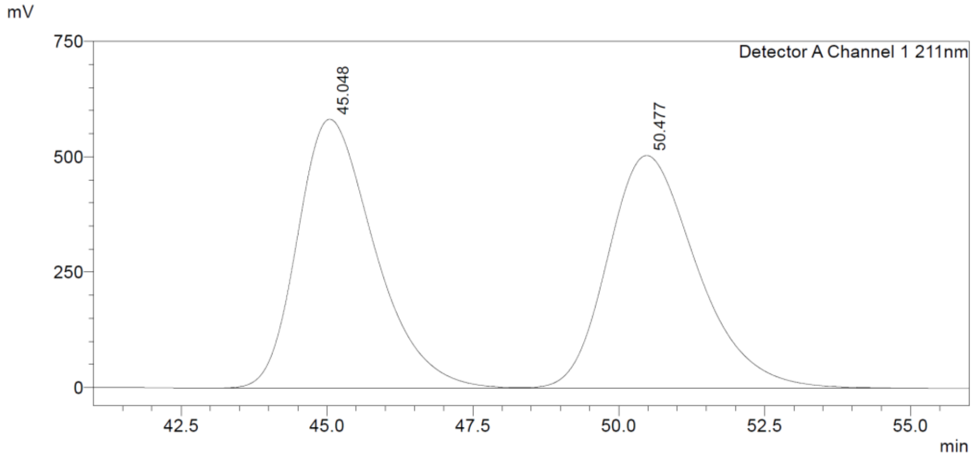

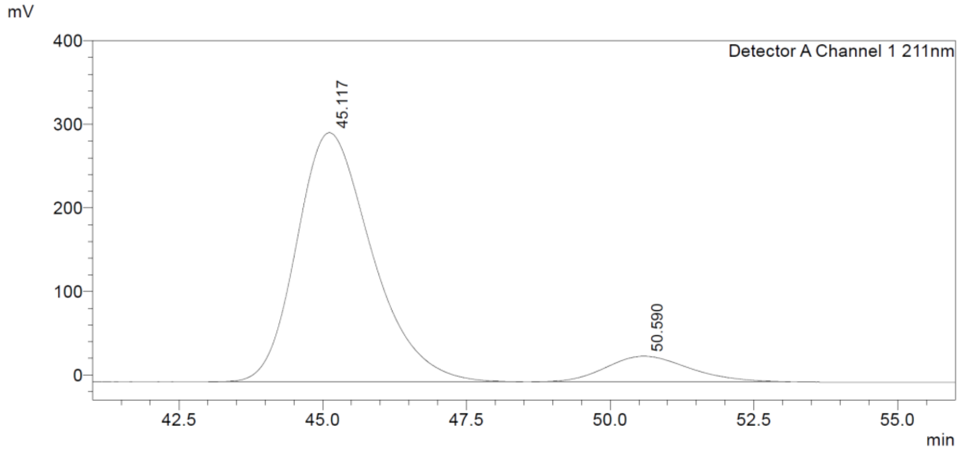

Racemic

Detector A Channel 1 211nm

Peak#	Ret. Time	Area%
1	45.048	49.940
2	50.477	50.060
Total		100.000

Enantioenriched

Detector A Channel 1 211nm

Peak#	Ret. Time	Area%
1	45.117	89.775
2	50.590	10.225
Total		100.000

Chiral HPLC analysis, Chiralpak IA (90:10 hexane:^{i}PrOH, flow rate 1 ml·min^{-1}, 211 nm, 30 °C) tR (*R*)-**49**: 16.0 min, tR (*S*)-**49**: 20.8 min, 96:4 er.

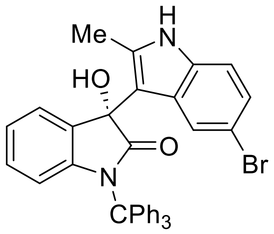

49

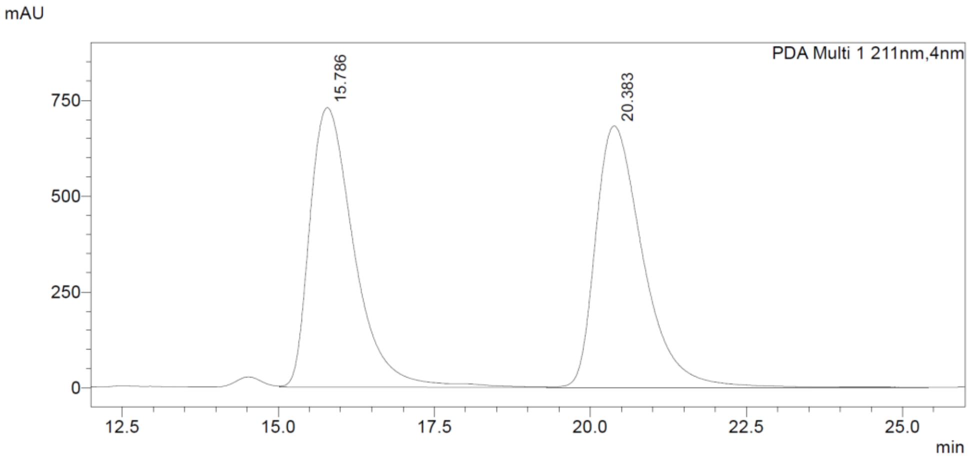

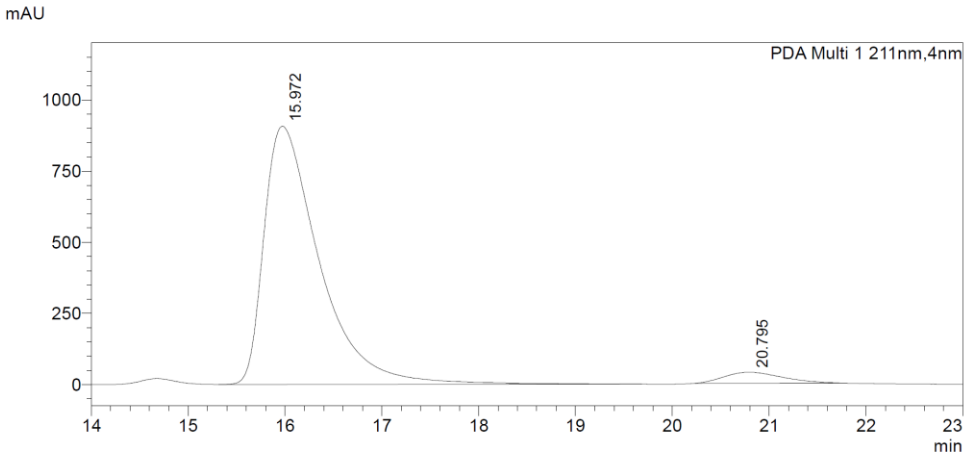

Racemic			Enantioenriched		
PDA Ch1 211nm			PDA Ch1 211nm		
Peak#	Ret. Time	Area%	Peak#	Ret. Time	Area%
1	15.786	50.048	1	15.972	95.739
2	20.383	49.952	2	20.795	4.261
Total		100.000	Total		100.000

9. NMR spectral data for compounds

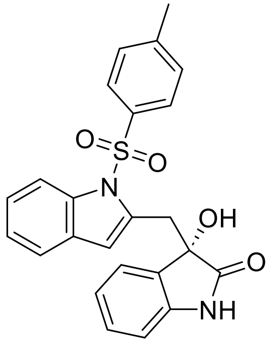

S1

^1H, $CDCl_3$, 500 MHz

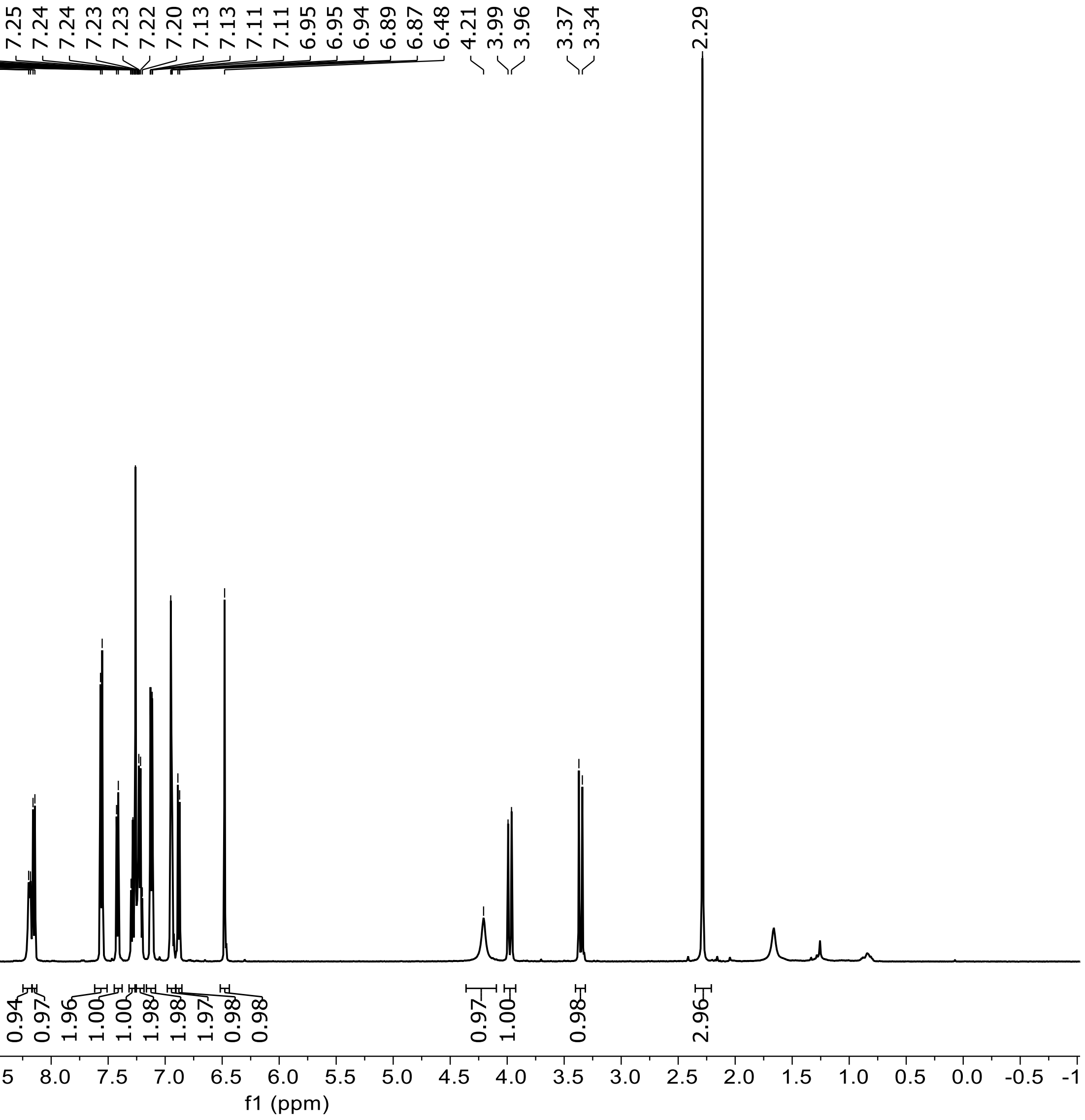

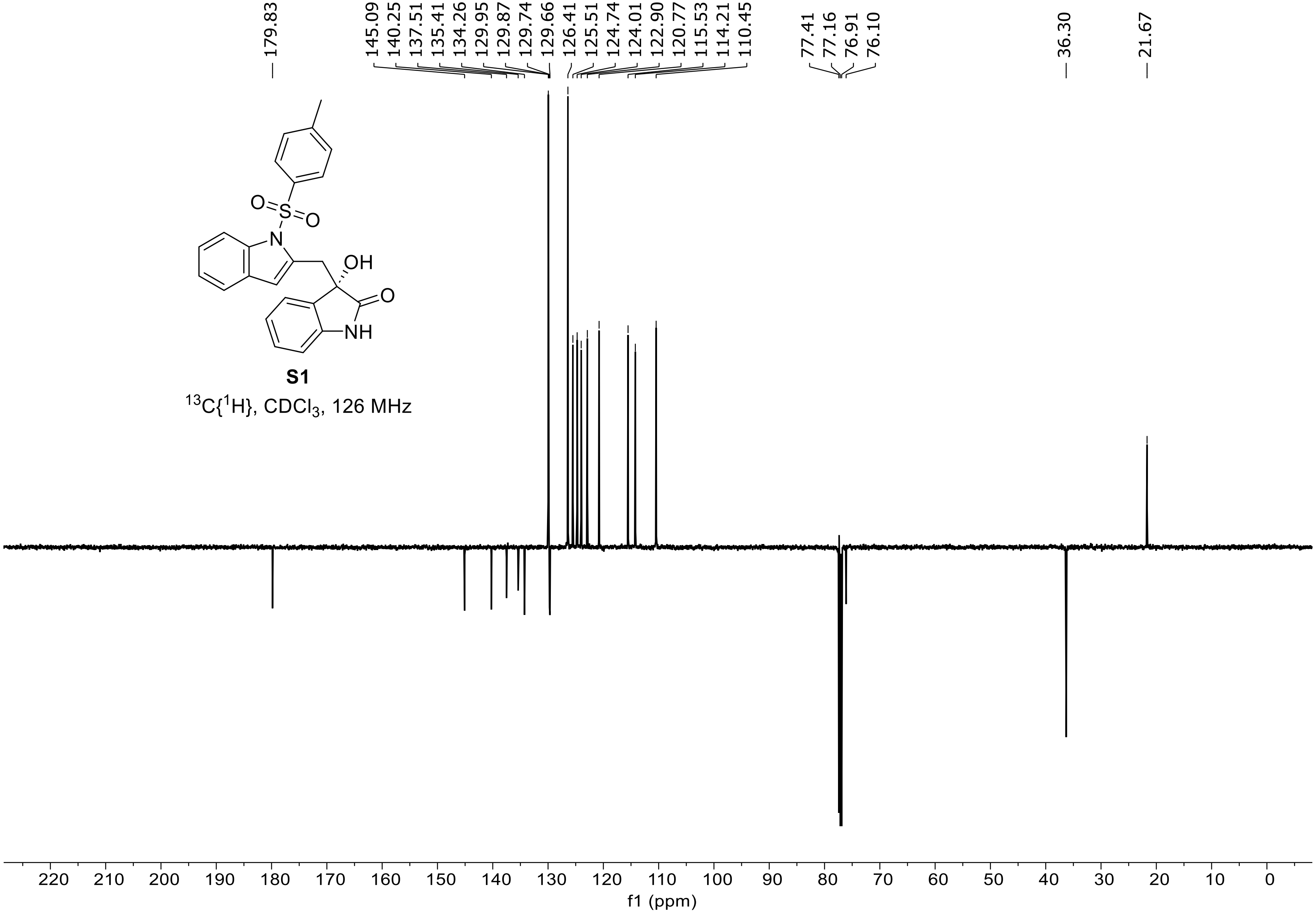

S1

$^{13}C\{^1H\}$, $CDCl_3$, 126 MHz

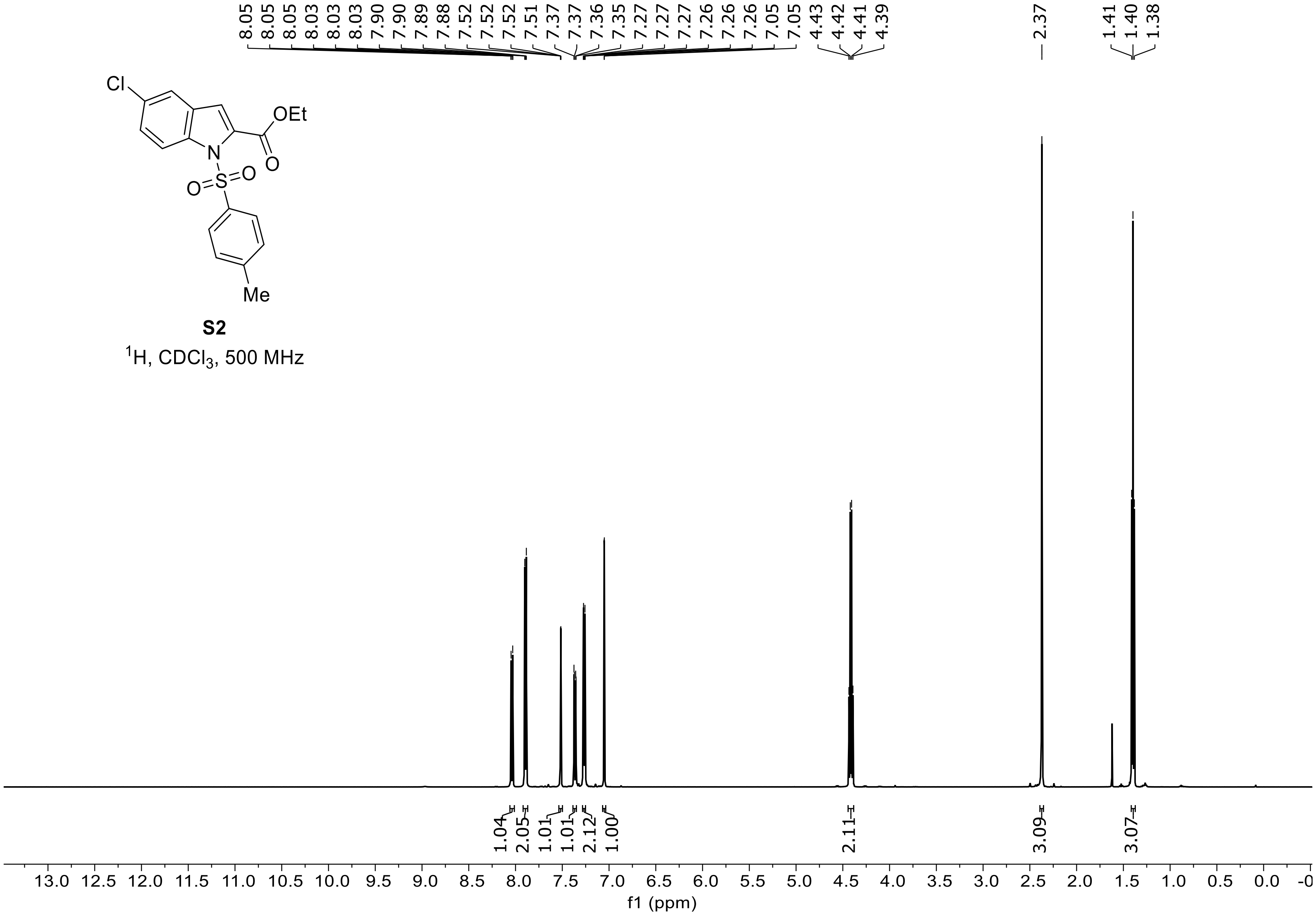

S2

^{1}H, $CDCl_3$, 500 MHz

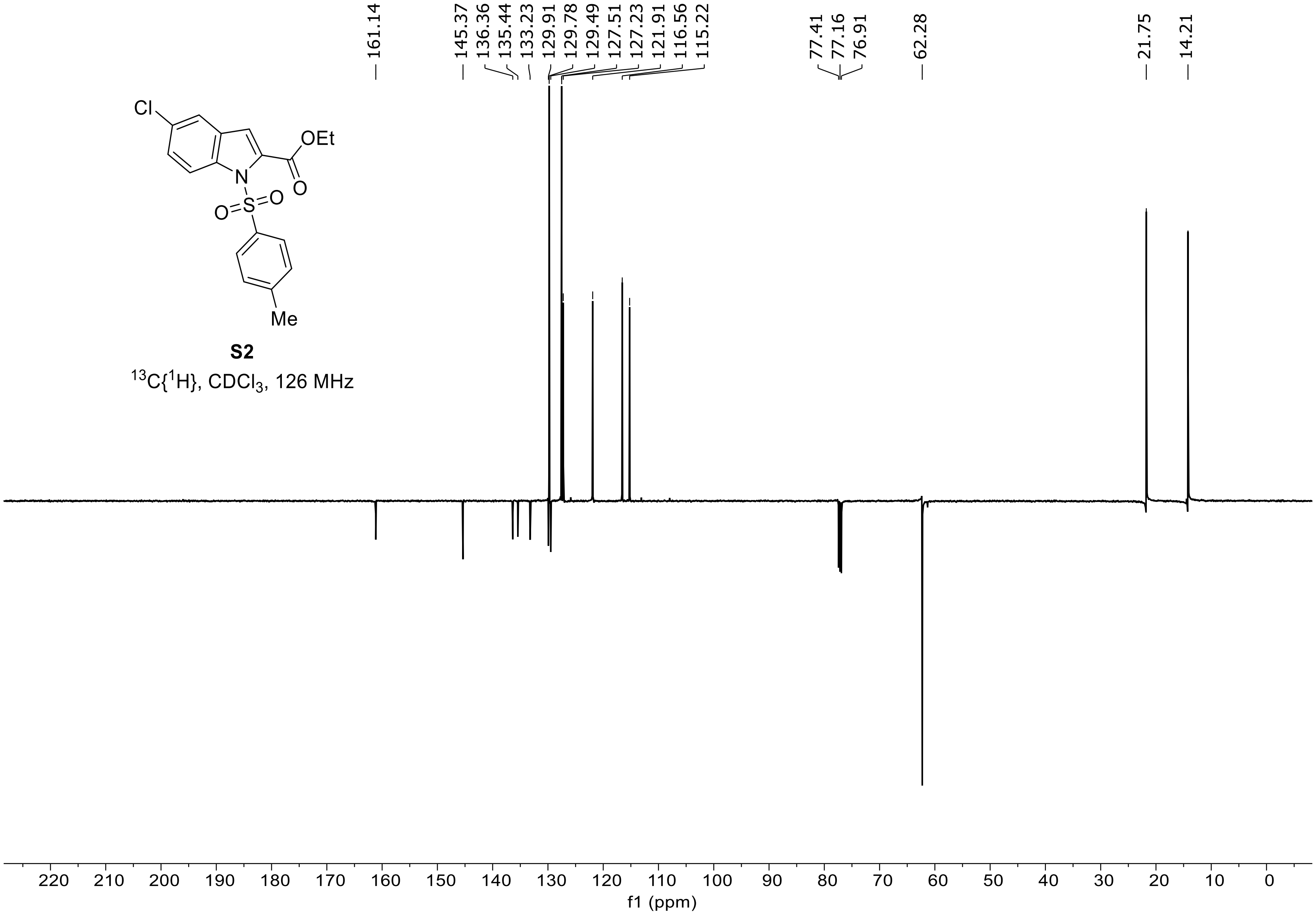

S2

$^{13}C\{^1H\}$, $CDCl_3$, 126 MHz

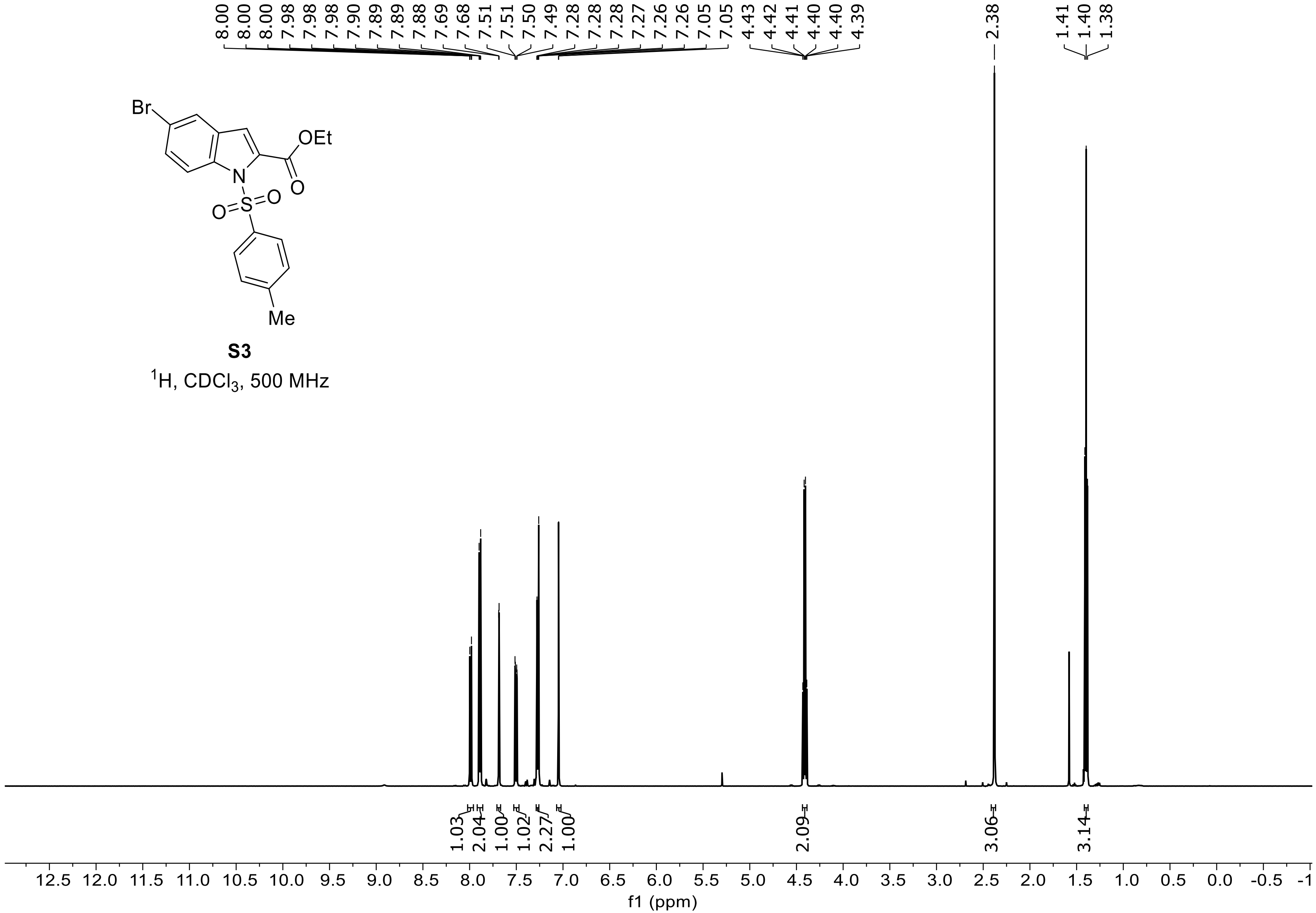
8.00 8.00 8.00 7.98 7.98 7.98 7.90 7.89 7.89 7.88 7.69 7.68 7.51 7.51 7.50 7.49 7.28 7.28 7.28 7.27 7.26 7.26 7.05 7.05 4.43 4.42 4.41 4.40 4.40 4.39 2.38 1.41 1.40 1.38
Br
OEt
N
O
S
O
O
Me
S3
1H, CDCl3, 500 MHz
1.03 2.04 1.00 1.02 2.27 1.00 2.09 3.06 3.14
12.5 12.0 11.5 11.0 10.5 10.0 9.5 9.0 8.5 8.0 7.5 7.0 6.5 6.0 5.5 5.0 4.5 4.0 3.5 3.0 2.5 2.0 1.5 1.0 0.5 0.0 -0.5 -1
f1 (ppm)

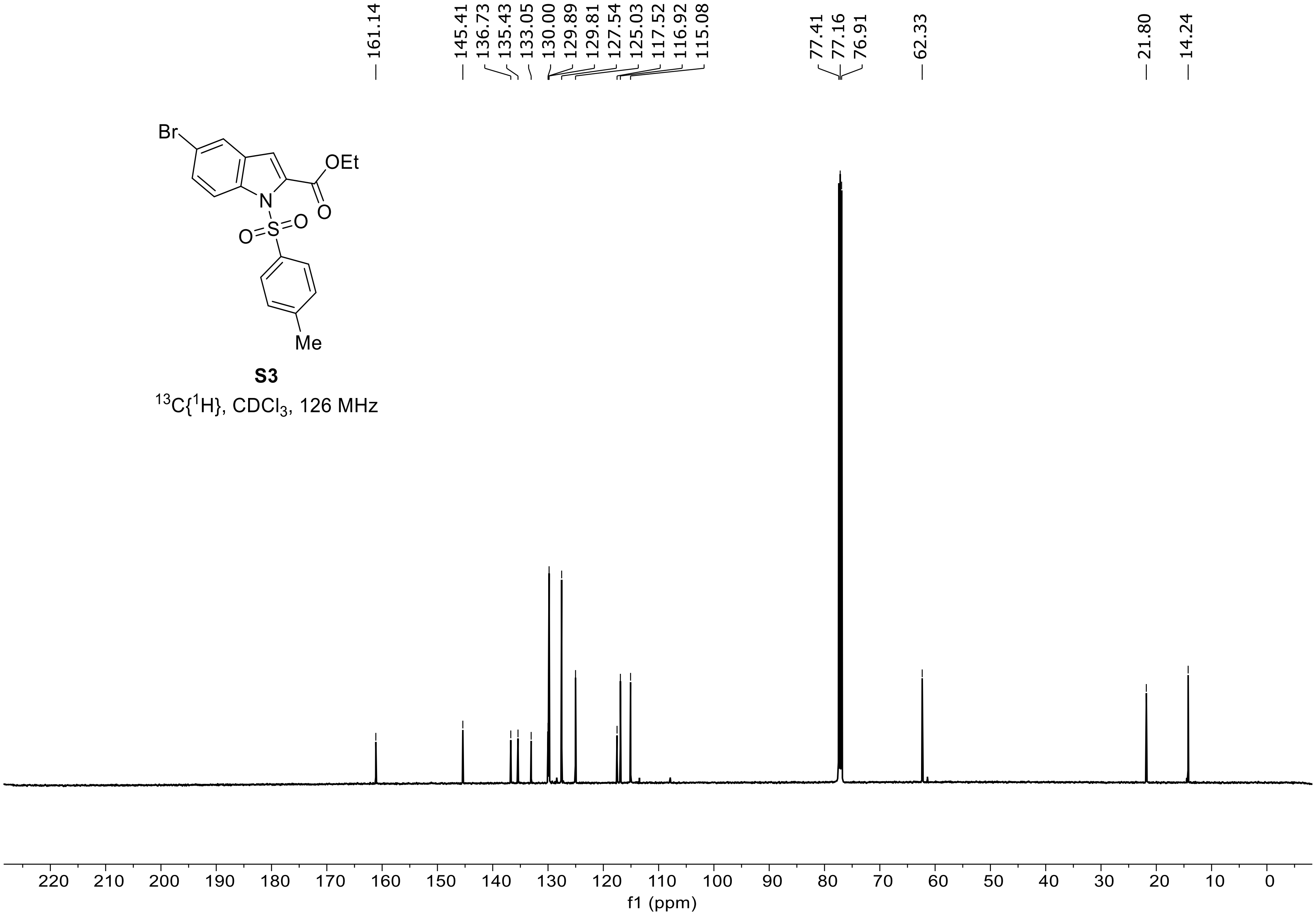

S3

$^{13}C\{^1H\}$, $CDCl_3$, 126 MHz

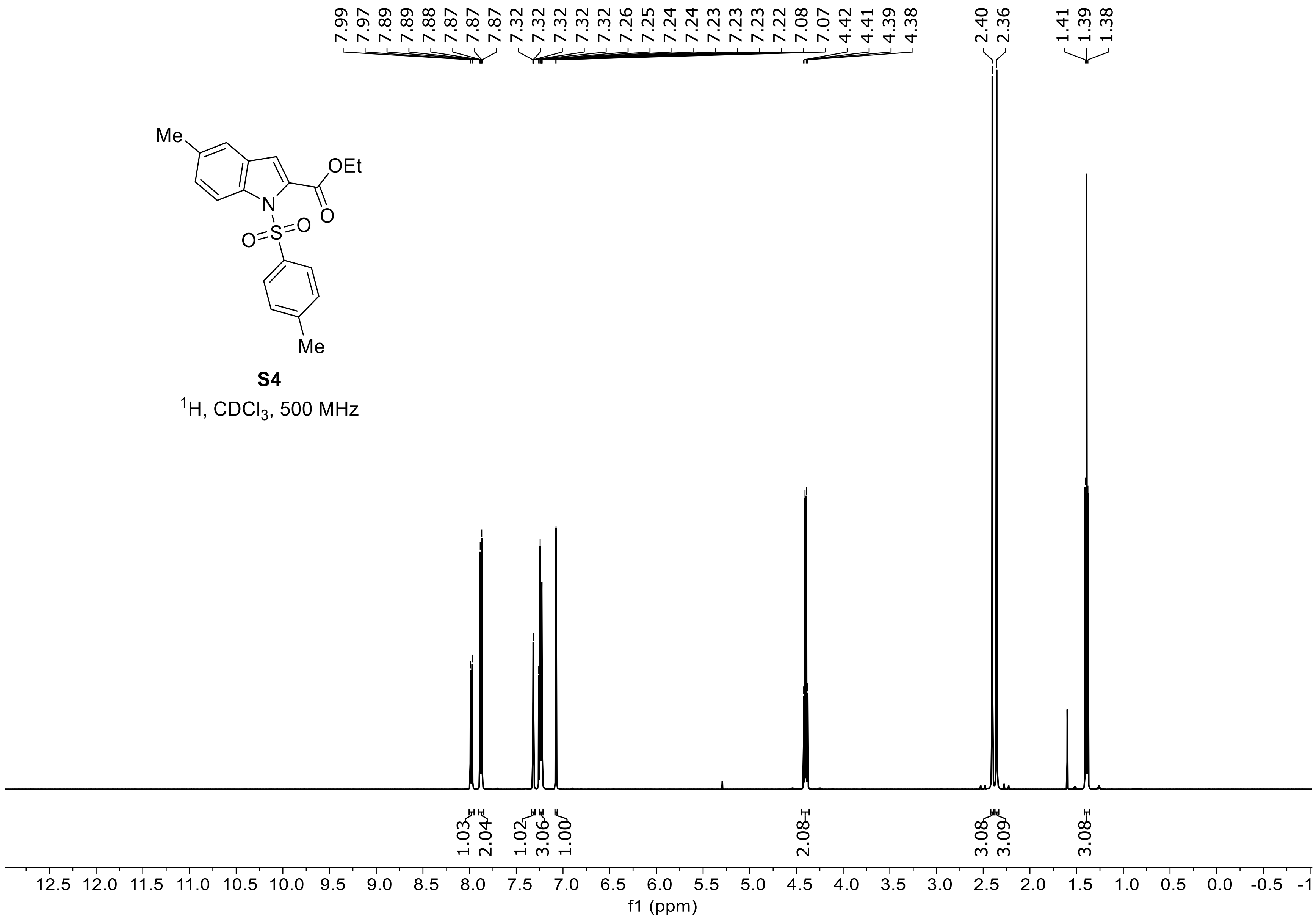
Me
OEt
N
O
S
O
O
Me
S4
¹H, CDCl3, 500 MHz
7.99 7.97 7.89 7.89 7.88 7.87 7.87 7.87 7.32 7.32 7.32 7.32 7.32 7.26 7.25 7.24 7.24 7.23 7.23 7.23 7.22 7.08 7.07 4.42 4.41 4.39 4.38
2.40 2.36
1.41 1.39 1.38
1.03 2.04 1.02 3.06 1.00 2.08 3.08 3.09 3.08
12.5 12.0 11.5 11.0 10.5 10.0 9.5 9.0 8.5 8.0 7.5 7.0 6.5 6.0 5.5 5.0 4.5 4.0 3.5 3.0 2.5 2.0 1.5 1.0 0.5 0.0 -0.5 -1
f1 (ppm)

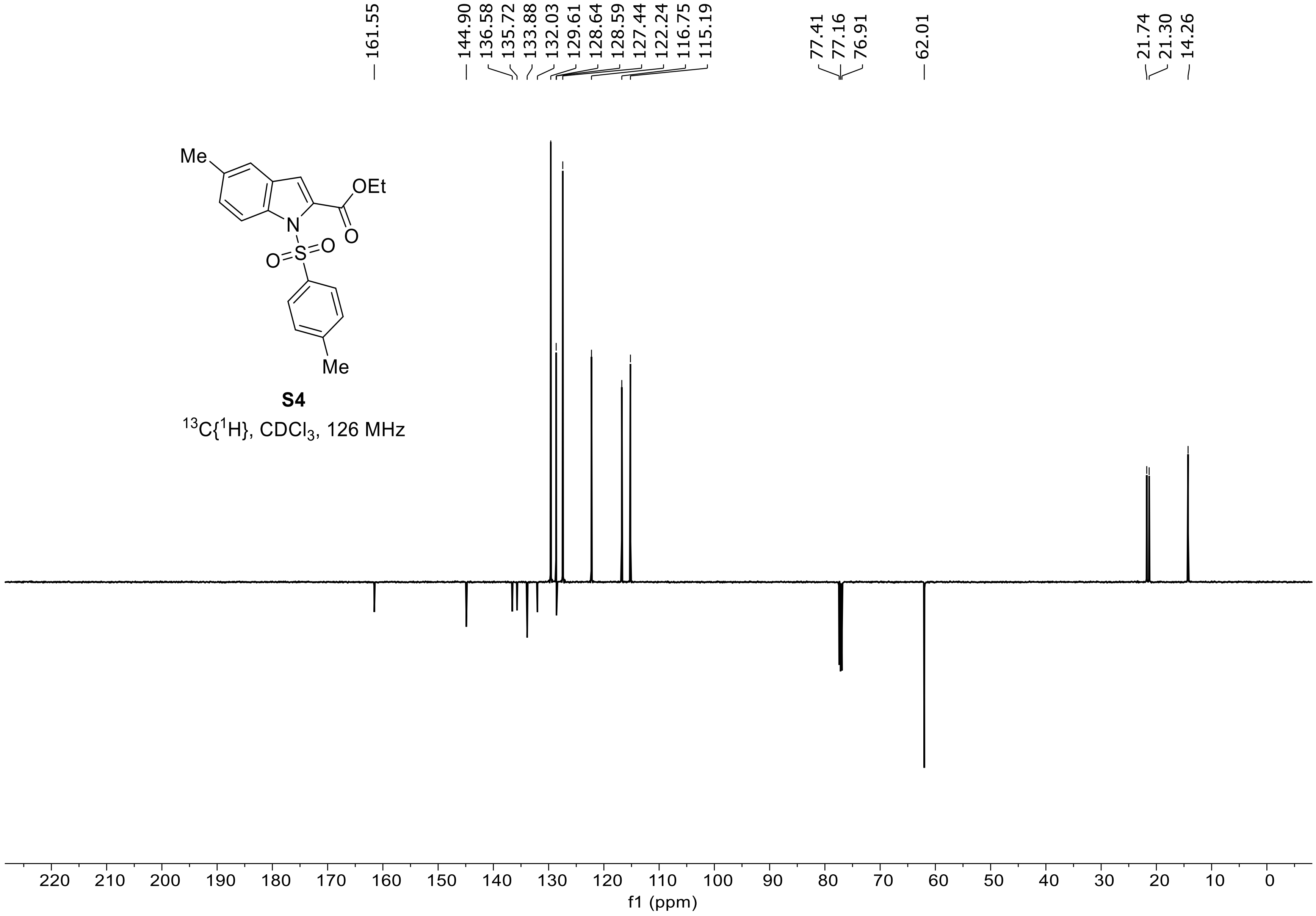
161.55
144.90
136.58
135.72
133.88
132.03
129.61
128.64
128.59
127.44
122.24
116.75
115.19
77.41
77.16
76.91
62.01
21.74
21.30
14.26
Me
OEt
N
O
S
O
O
Me
S4
$^{13}C\{^1H\}$, $CDCl_3$, 126 MHz
220 210 200 190 180 170 160 150 140 130 120 110 100 90 80 70 60 50 40 30 20 10 0
f1 (ppm)

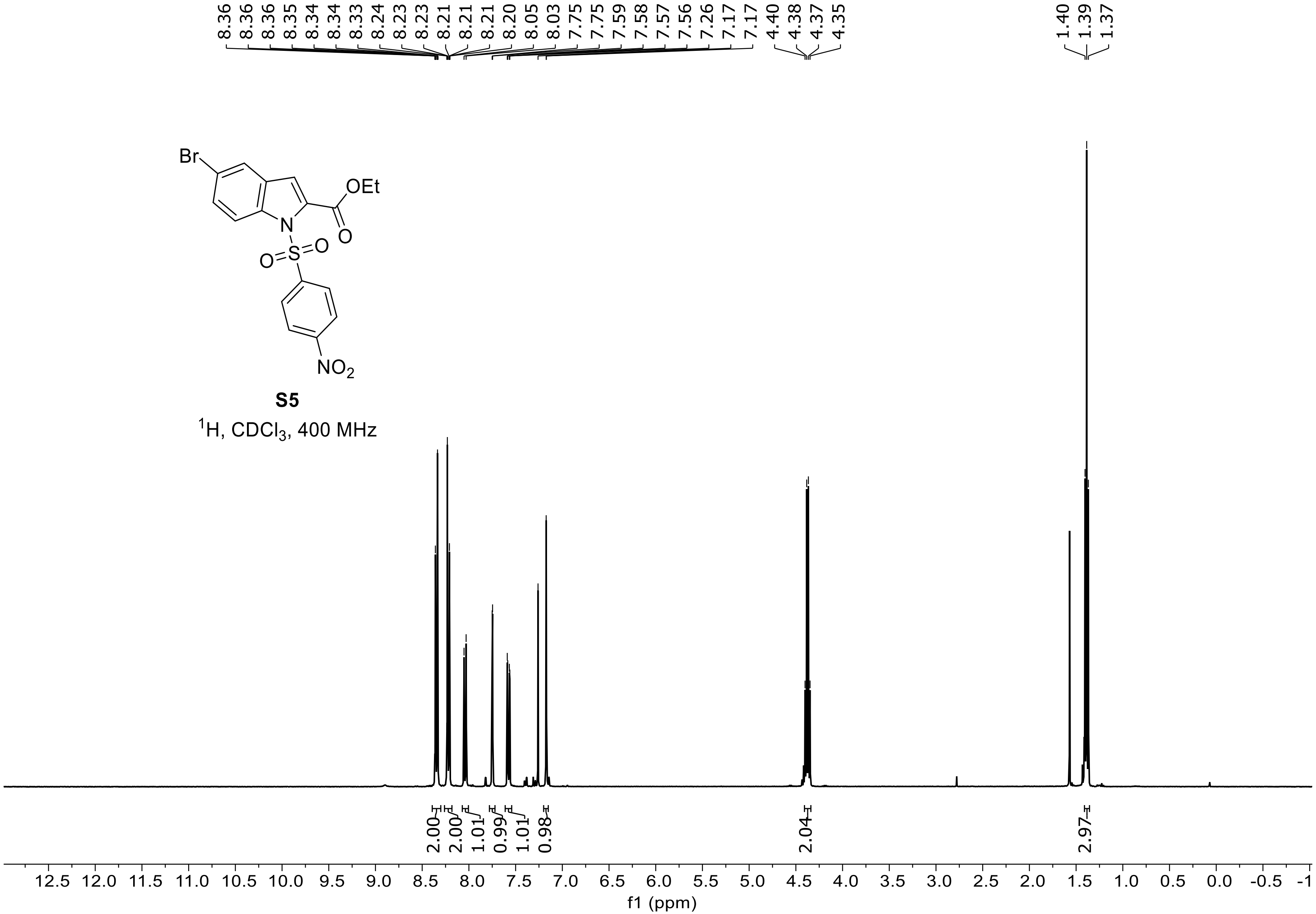

S5

^{1}H, $CDCl_3$, 400 MHz

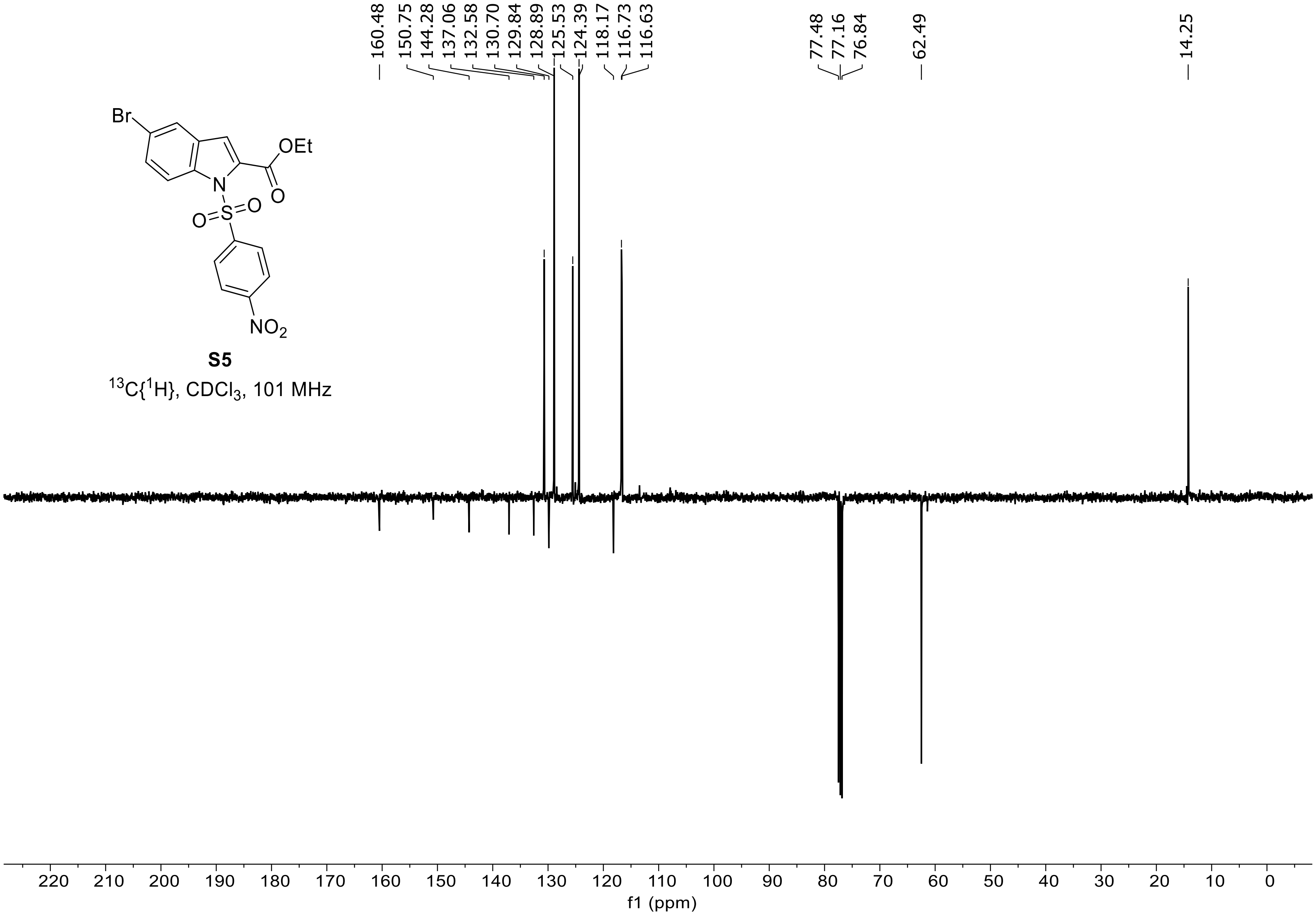

S5

$^{13}C\{^{1}H\}$, $CDCl_3$, 101 MHz

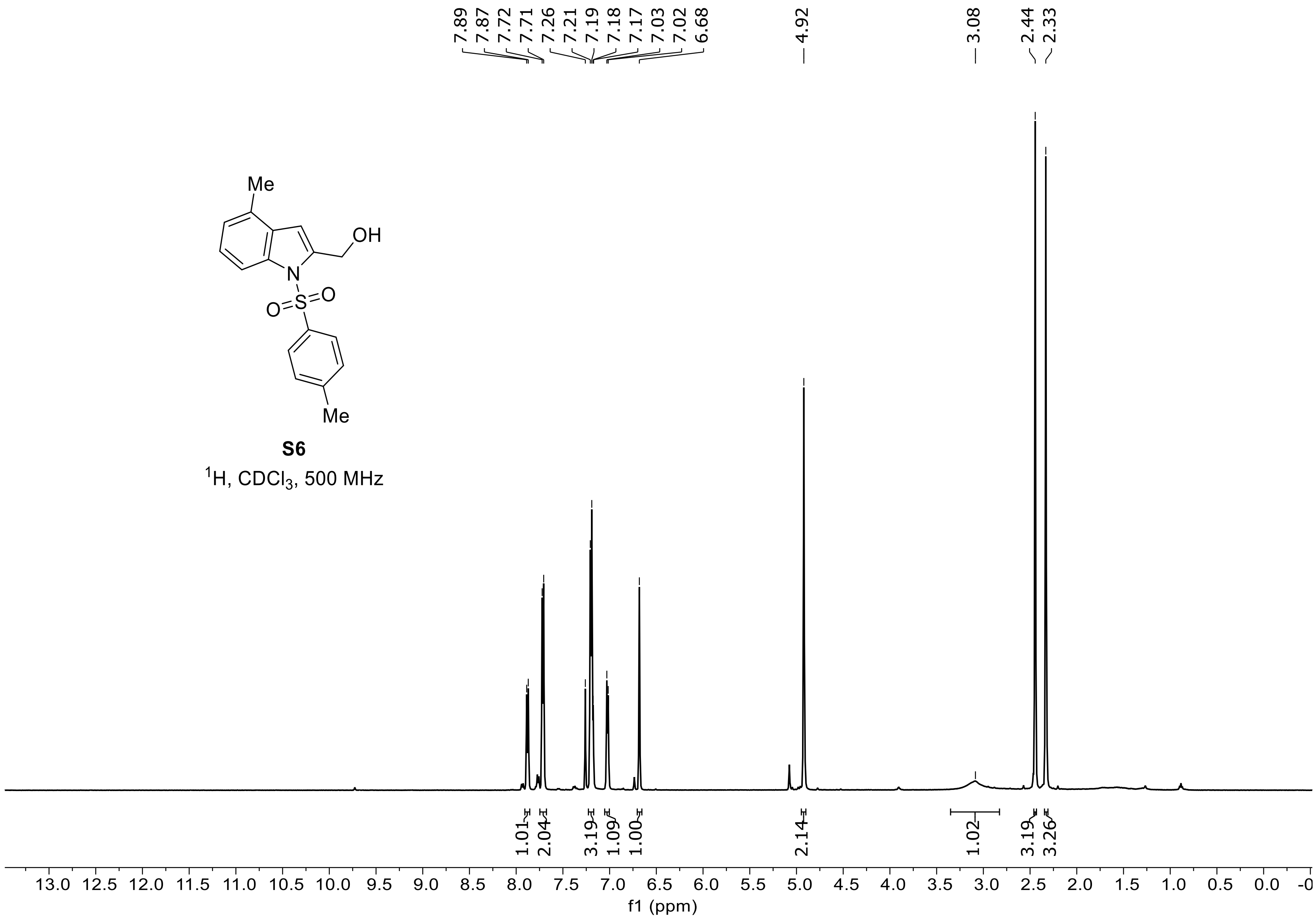

7.89
7.87
7.72
7.71
7.26
7.21
7.19
7.18
7.17
7.03
7.02
6.68
4.92
3.08
2.44
2.33
Me
OH
N
O=S=O
Me
S6
^{1}H, CDCl$_3$, 500 MHz
1.01
2.04
3.19
1.09
1.00
2.14
1.02
3.19
3.26
13.0 12.5 12.0 11.5 11.0 10.5 10.0 9.5 9.0 8.5 8.0 7.5 7.0 6.5 6.0 5.5 5.0 4.5 4.0 3.5 3.0 2.5 2.0 1.5 1.0 0.5 0.0
f1 (ppm)

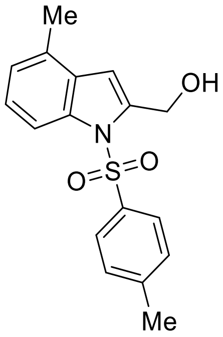

S6

$^{13}C\{^1H\}$, $CDCl_3$, 126 MHz

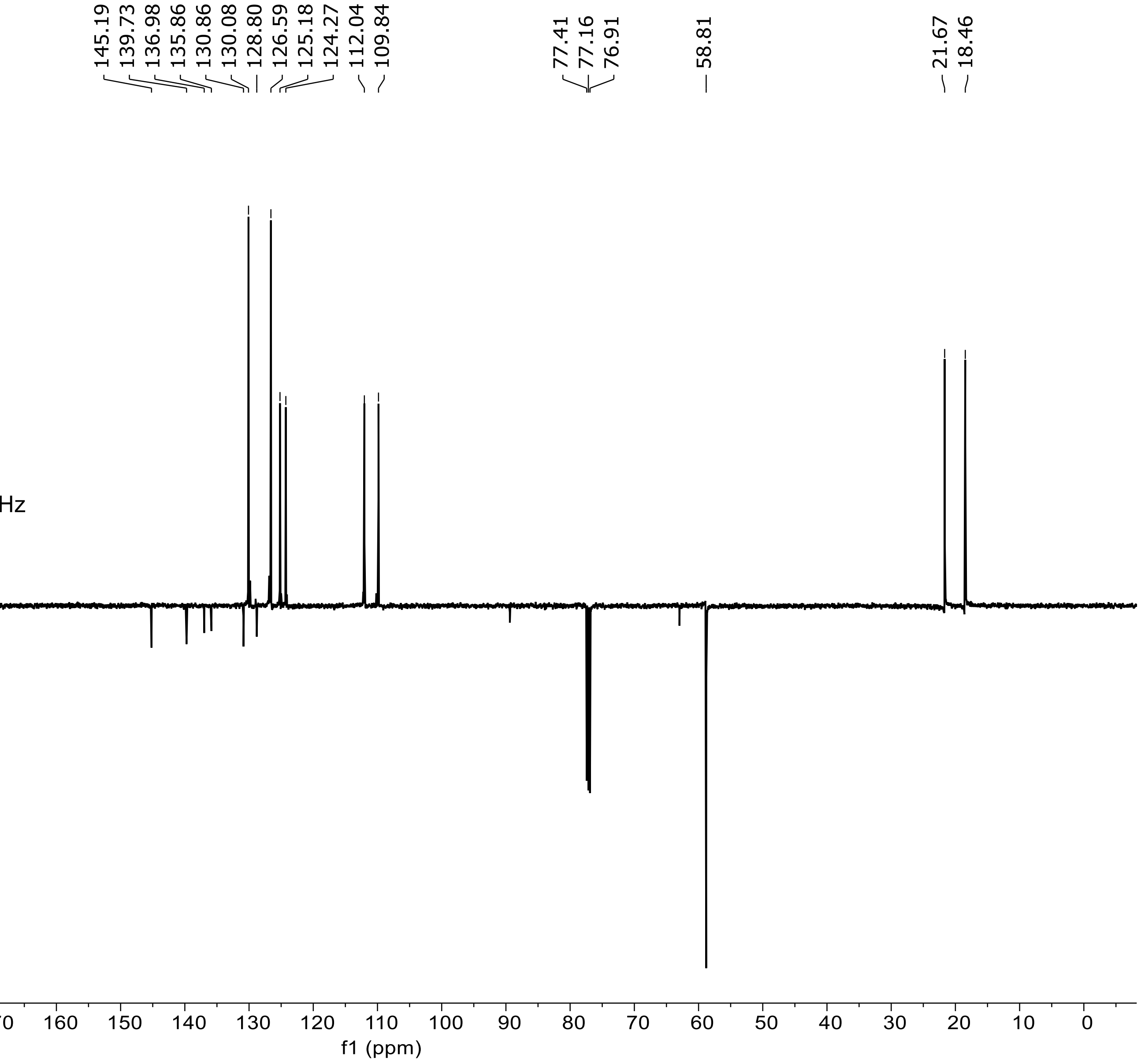

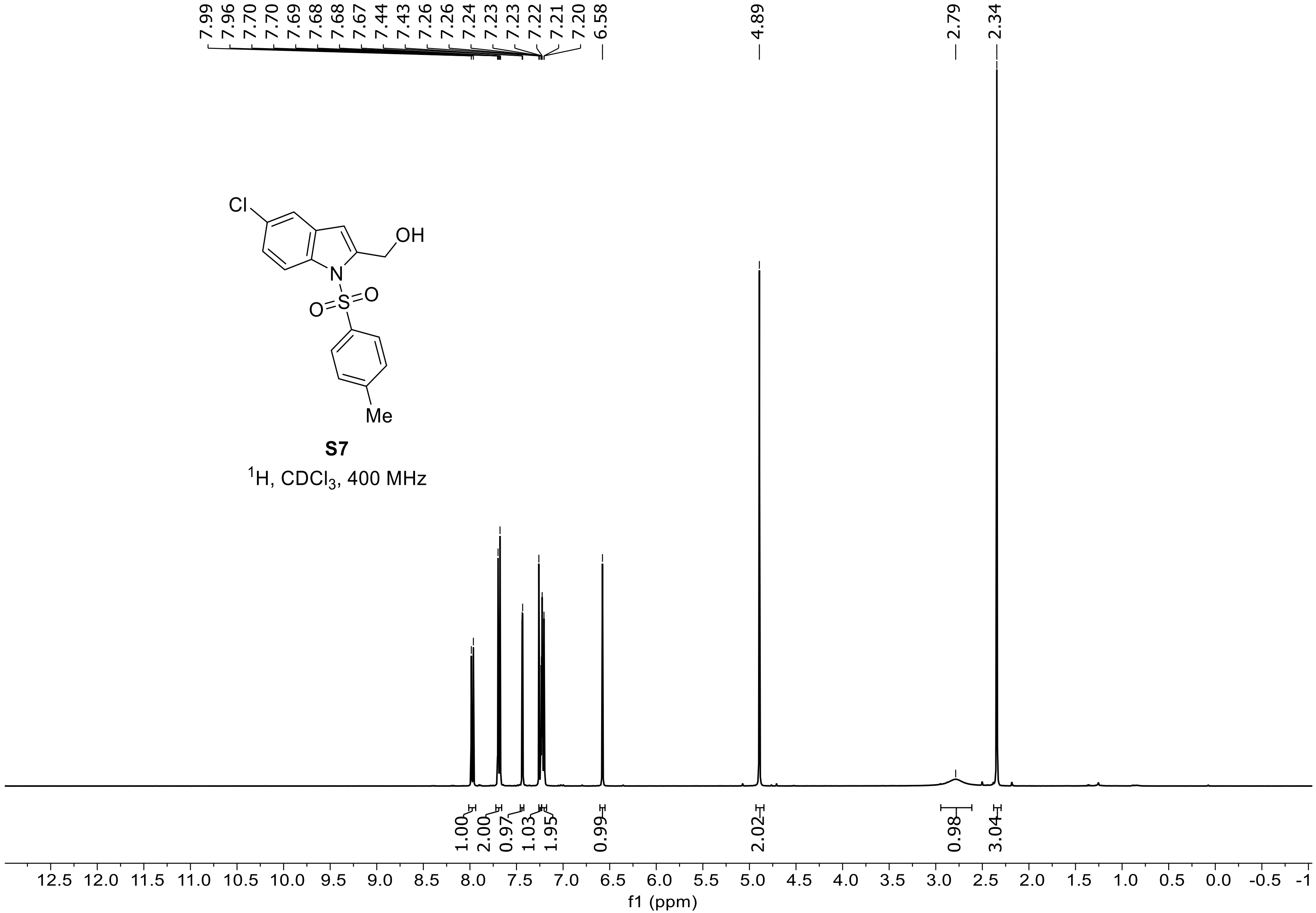
7.99
7.96
7.70
7.70
7.69
7.68
7.68
7.67
7.44
7.43
7.26
7.26
7.24
7.23
7.23
7.22
7.21
7.20
6.58
4.89
2.79
2.34
Cl
OH
N
O
S
O
Me
S7
^{1}H, $CDCl_3$, 400 MHz
1.00
2.00
0.97
1.03
1.95
0.99
2.02
0.98
3.04
12.5
12.0
11.5
11.0
10.5
10.0
9.5
9.0
8.5
8.0
7.5
7.0
6.5
6.0
5.5
5.0
4.5
4.0
3.5
3.0
2.5
2.0
1.5
1.0
0.5
0.0
-0.5
-1
f1 (ppm)

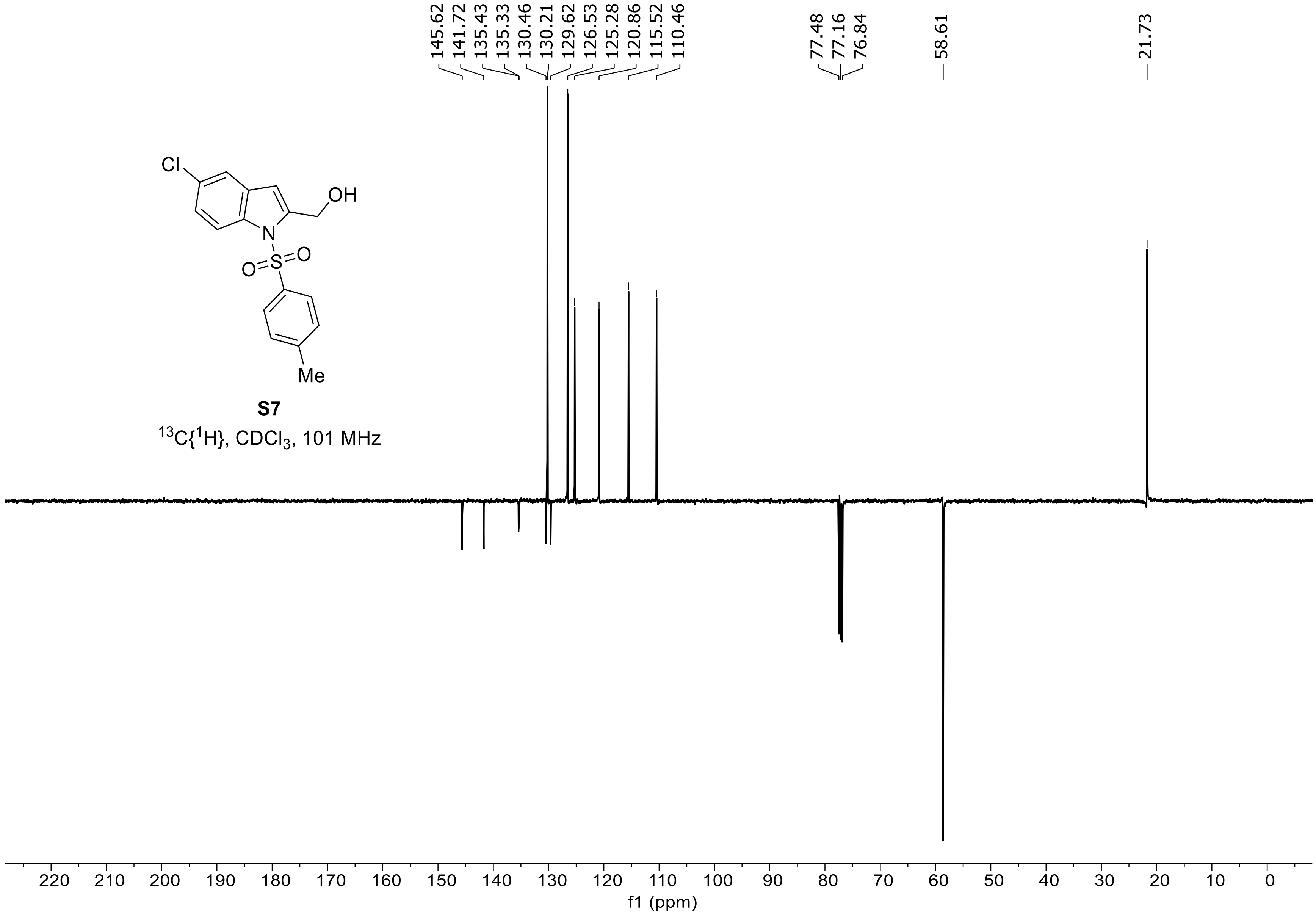
145.62
141.72
135.43
135.33
130.46
130.21
129.62
126.53
125.28
120.86
115.52
110.46
77.48
77.16
76.84
58.61
21.73
Cl
OH
N
O
S
O
Me
S7
$^{13}C\{^{1}H\}$, $CDCl_3$, 101 MHz
220 210 200 190 180 170 160 150 140 130 120 110 100 90 80 70 60 50 40 30 20 10 0
f1 (ppm)

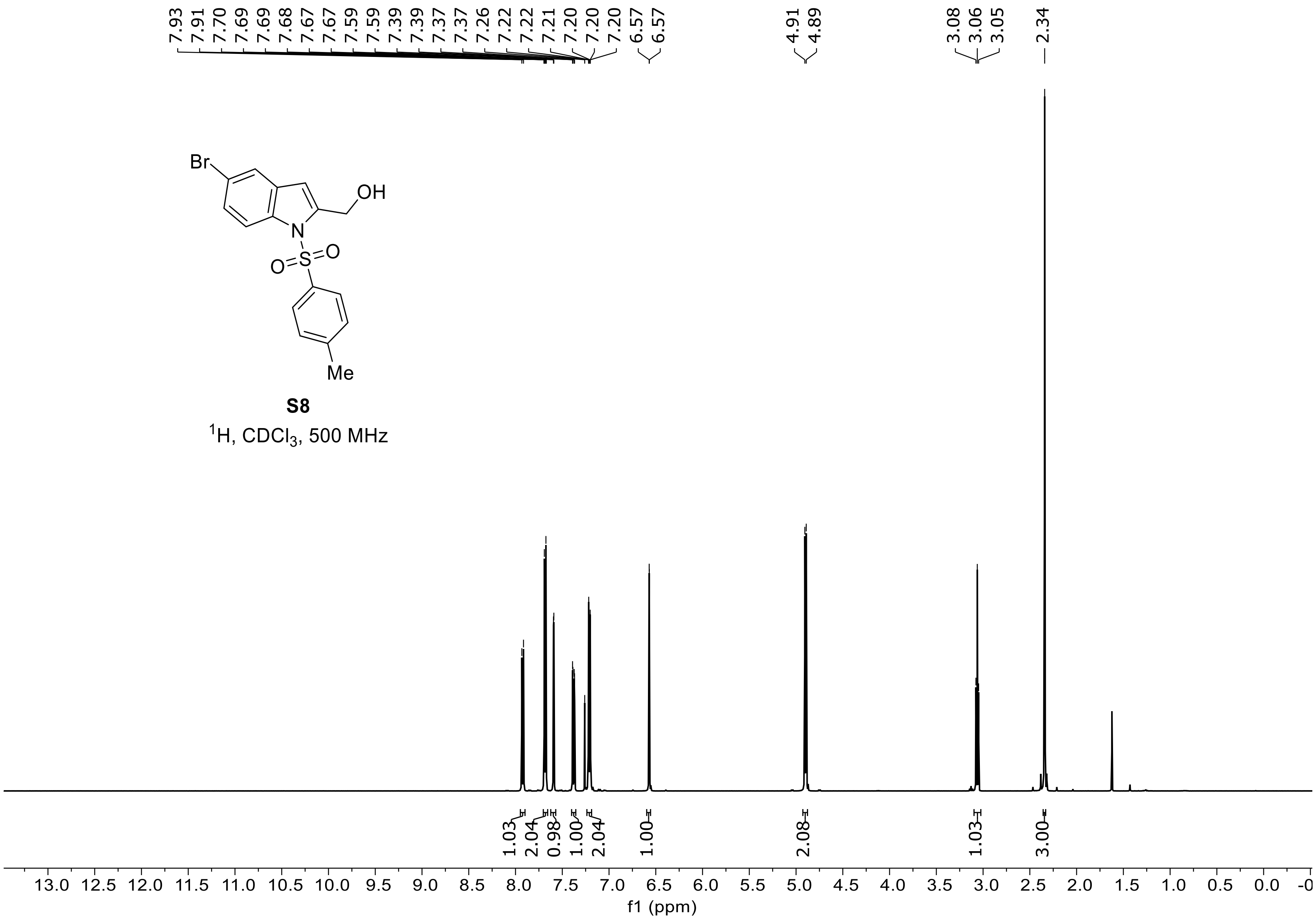
7.93
7.91
7.70
7.69
7.69
7.68
7.67
7.67
7.59
7.59
7.39
7.39
7.37
7.37
7.26
7.22
7.22
7.21
7.20
7.20
7.20
6.57
6.57
4.91
4.89
3.08
3.06
3.05
2.34
Br
OH
N
O=S=O
Me
S8
^{1}H, $CDCl_3$, 500 MHz
1.03
2.04
0.98
1.00
2.04
1.00
2.08
1.03
3.00
13.0 12.5 12.0 11.5 11.0 10.5 10.0 9.5 9.0 8.5 8.0 7.5 7.0 6.5 6.0 5.5 5.0 4.5 4.0 3.5 3.0 2.5 2.0 1.5 1.0 0.5 0.0 -0
f1 (ppm)

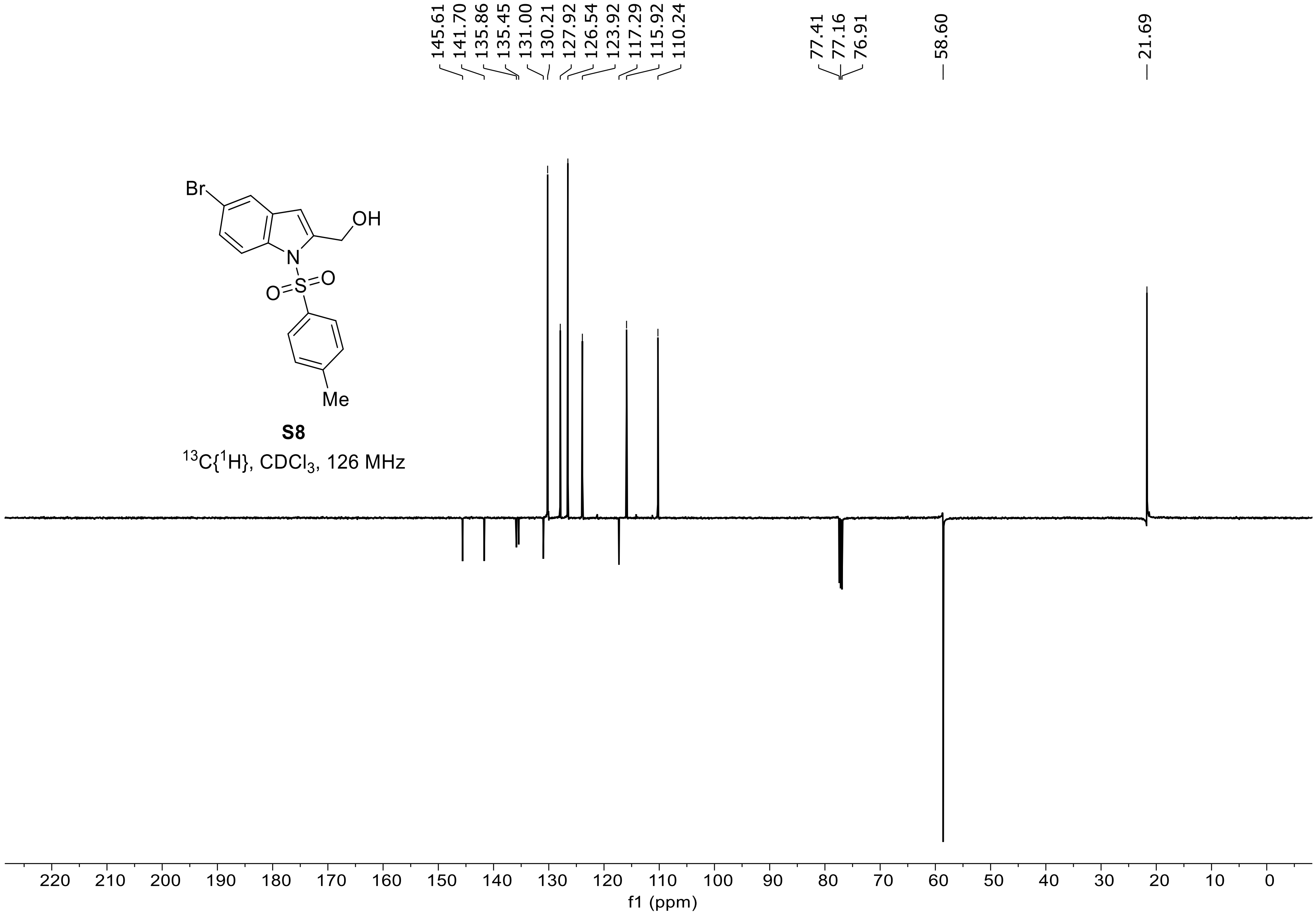
145.61
141.70
135.86
135.45
131.00
130.21
127.92
126.54
123.92
117.29
115.92
110.24
77.41
77.16
76.91
58.60
21.69
Br
OH
N
S
O
O
Me
S8
$^{13}C\{^1H\}$, $CDCl_3$, 126 MHz
220 210 200 190 180 170 160 150 140 130 120 110 100 90 80 70 60 50 40 30 20 10 0
f1 (ppm)

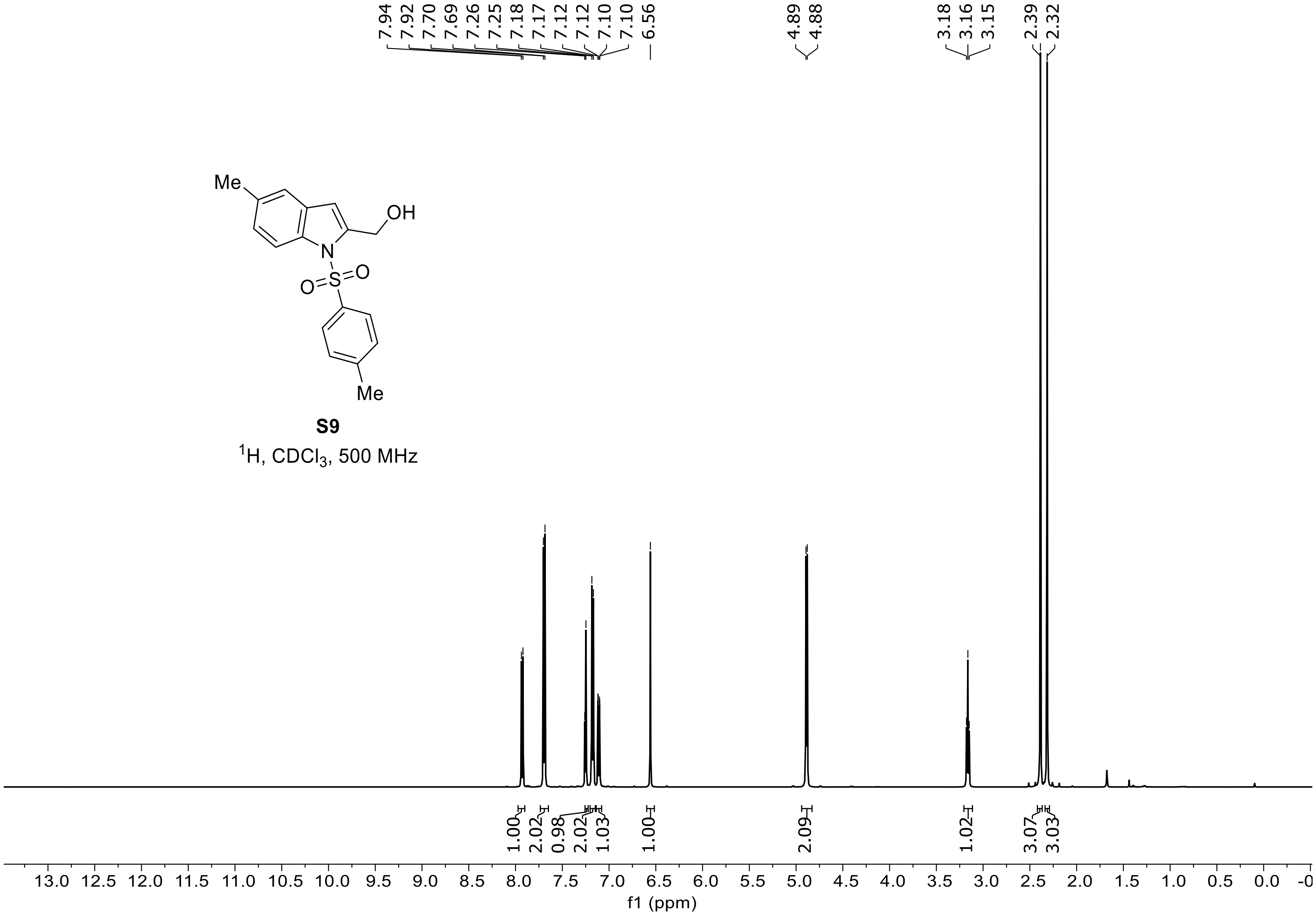

S9

^{1}H, $CDCl_3$, 500 MHz

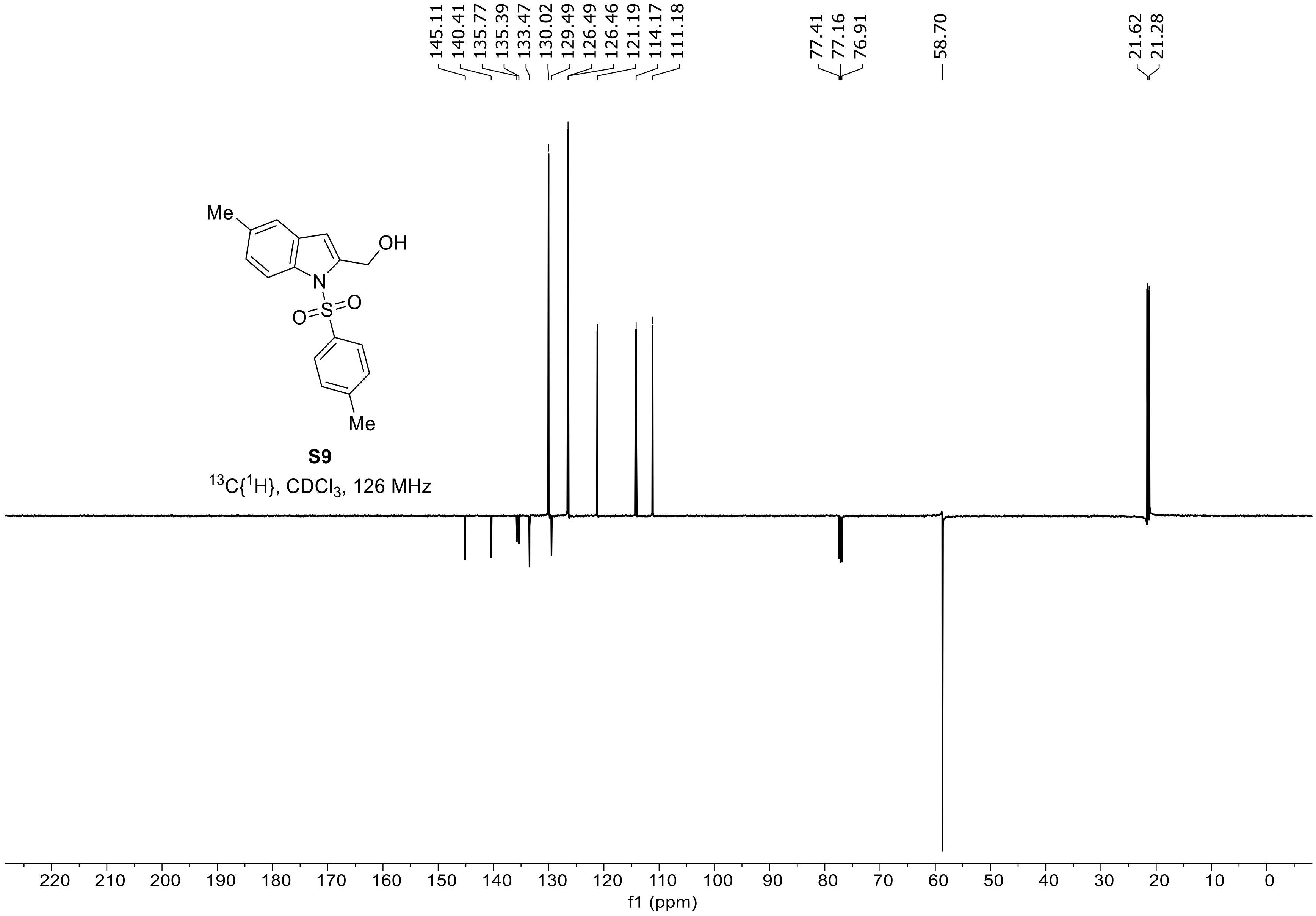
145.11
140.41
135.77
135.39
133.47
130.02
129.49
126.49
126.46
121.19
114.17
111.18
77.41
77.16
76.91
58.70
21.62
21.28
Me
OH
N
O
S
O
Me
S9
$^{13}C\{^1H\}$, $CDCl_3$, 126 MHz
220 210 200 190 180 170 160 150 140 130 120 110 100 90 80 70 60 50 40 30 20 10 0
f1 (ppm)

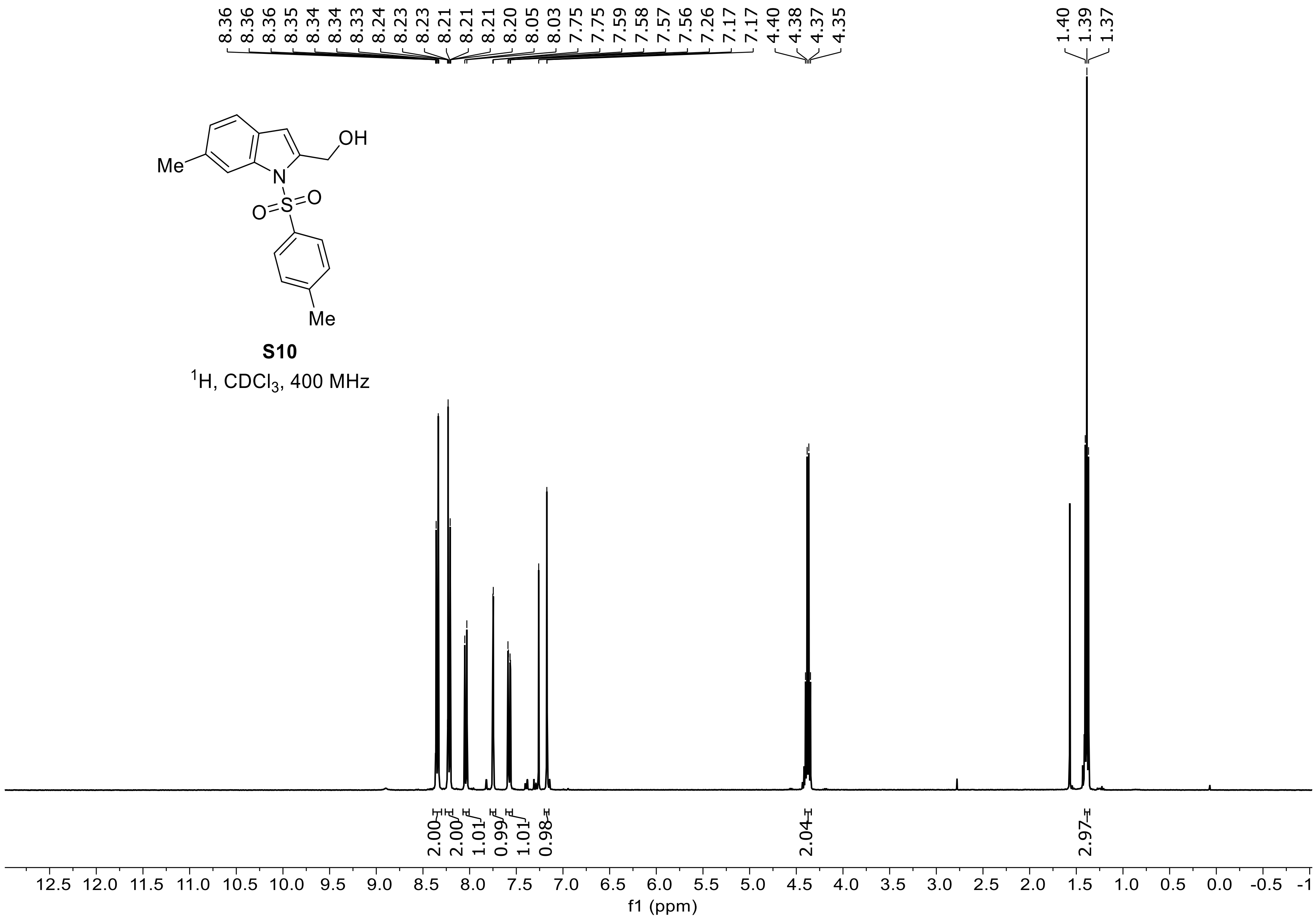

S10

^{1}H, $CDCl_3$, 400 MHz

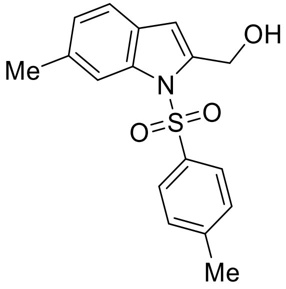

S10

$^{13}C\{^{1}H\}$, $CDCl_3$, 101 MHz

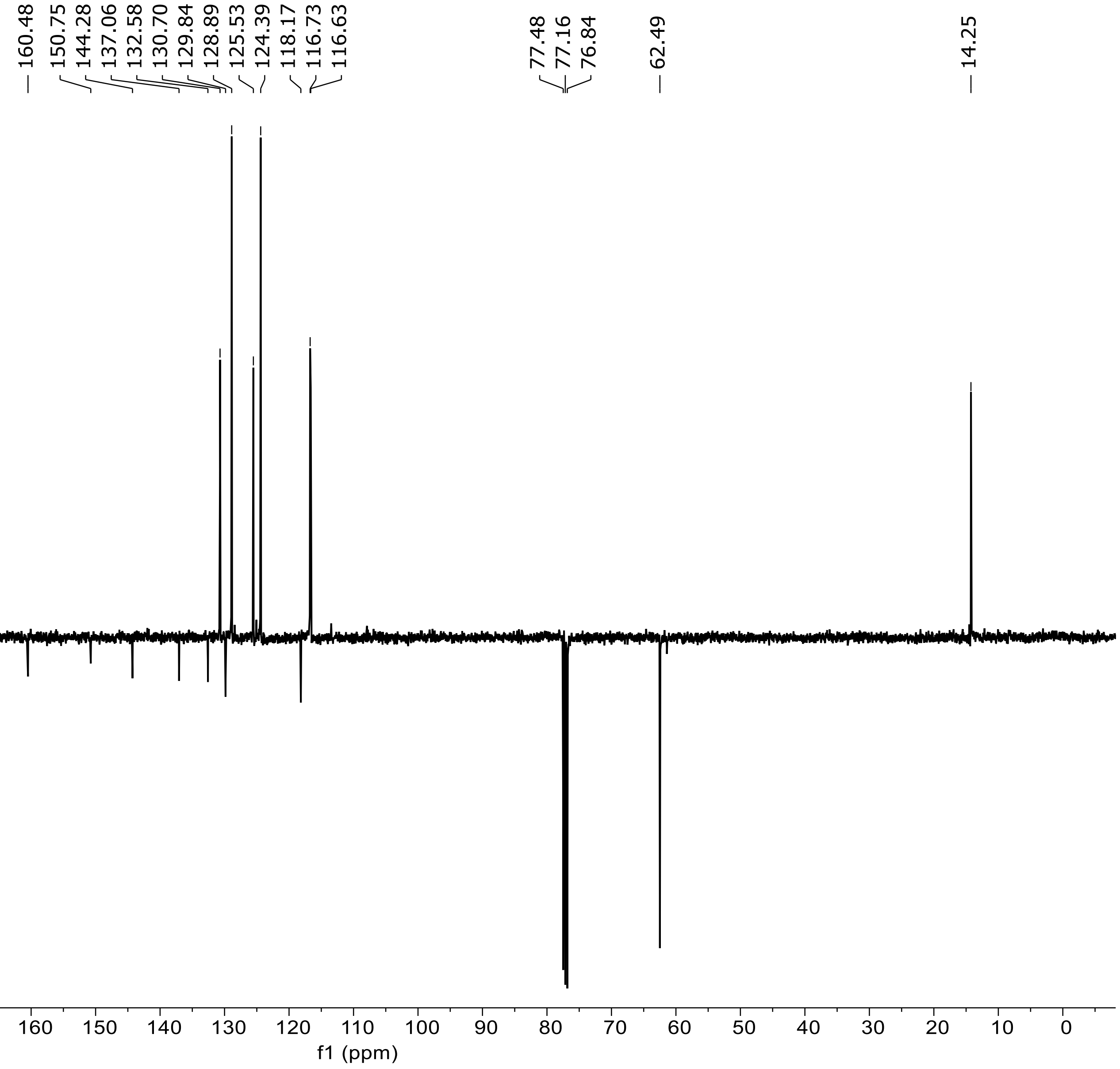

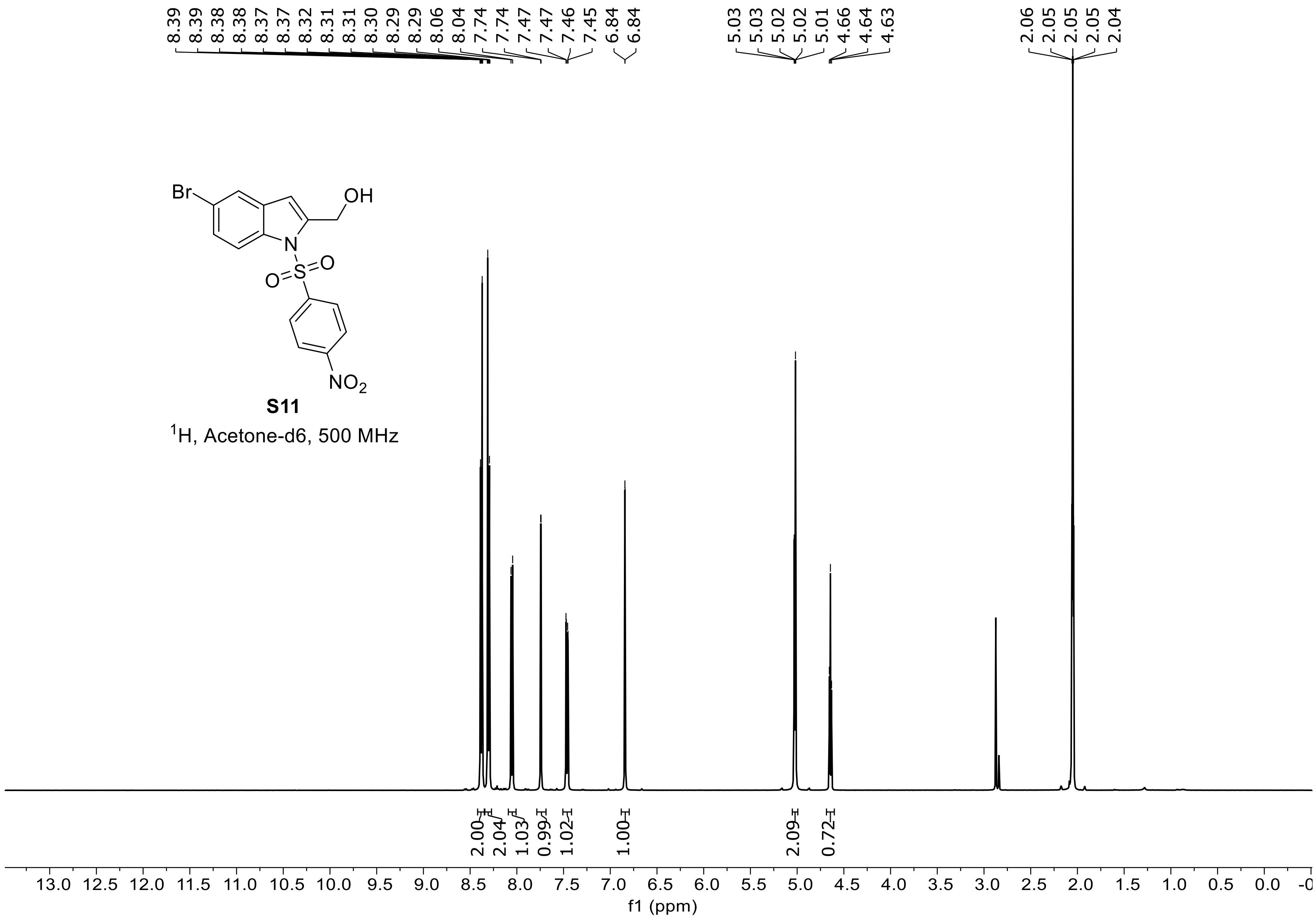

S11

^{1}H, Acetone-d6, 500 MHz

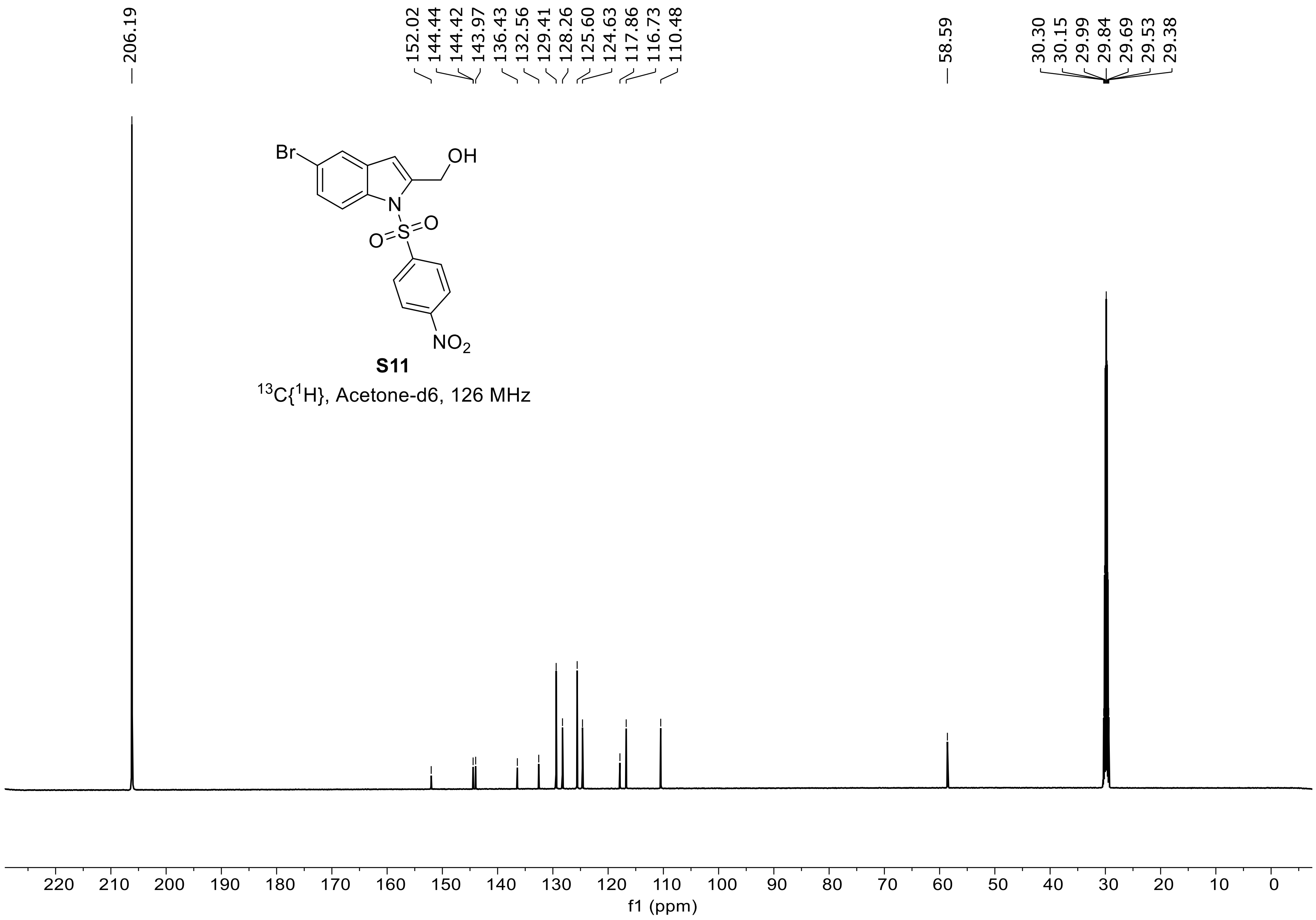
206.19
152.02
144.44
144.42
143.97
136.43
132.56
129.41
128.26
125.60
124.63
117.86
116.73
110.48
58.59
30.30
30.15
29.99
29.84
29.69
29.53
29.38
Br
OH
N
S
O
O
NO2
S11
13C{1H}, Acetone-d6, 126 MHz
220 210 200 190 180 170 160 150 140 130 120 110 100 90 80 70 60 50 40 30 20 10 0
f1 (ppm)

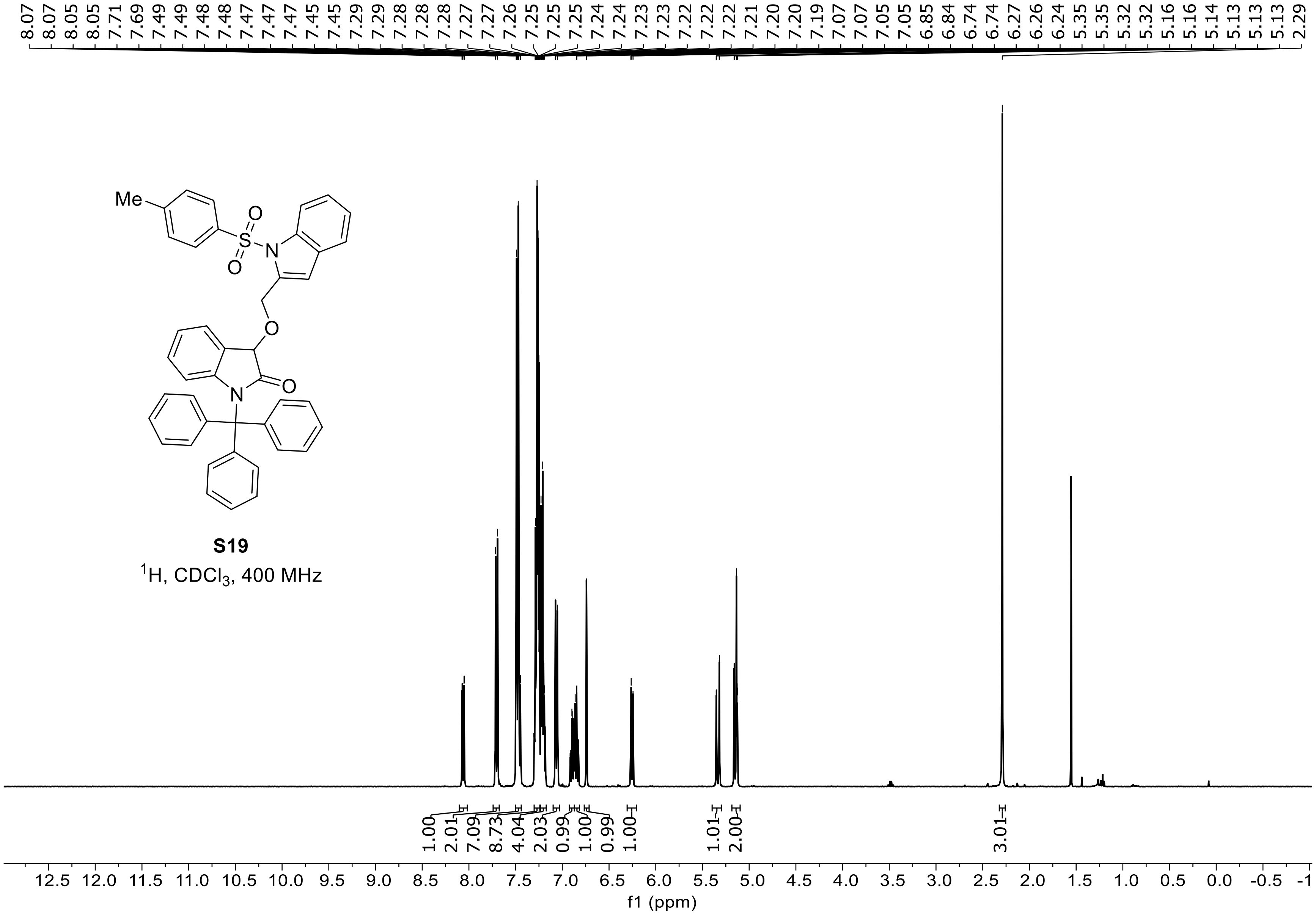

S19

^{1}H, $CDCl_3$, 400 MHz

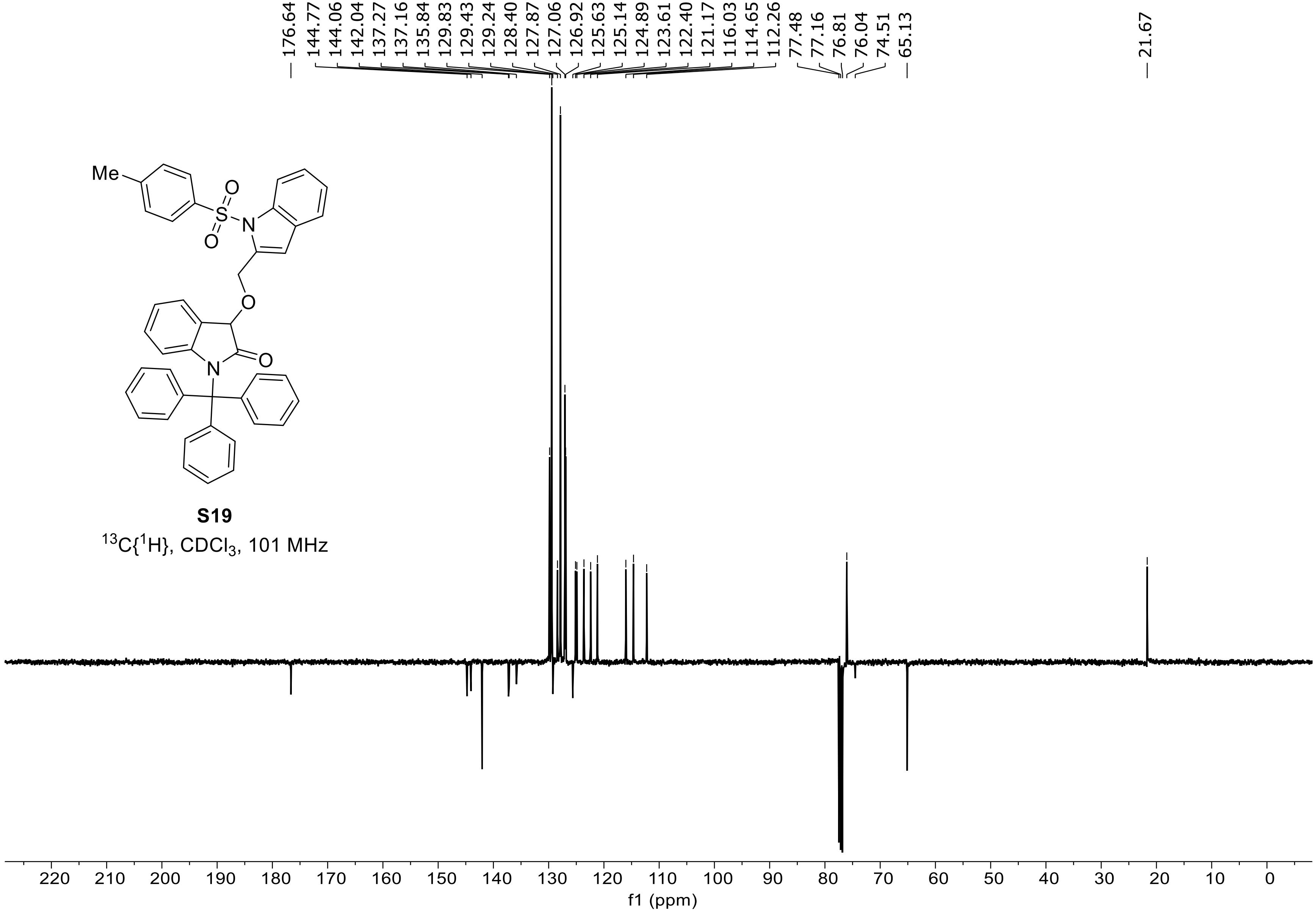

S19

$^{13}C\{^{1}H\}$, $CDCl_3$, 101 MHz

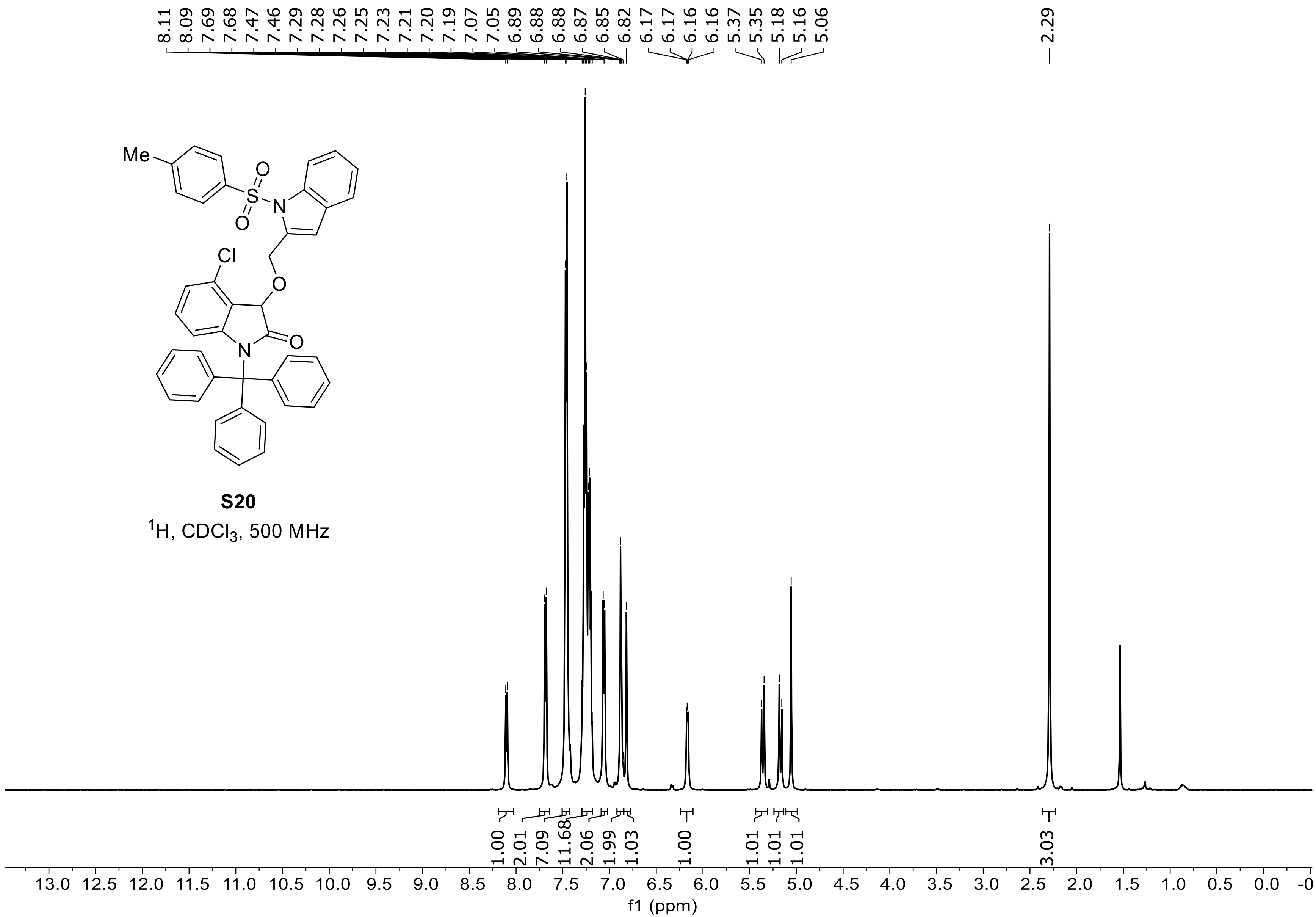

S20

^{1}H, $CDCl_3$, 500 MHz

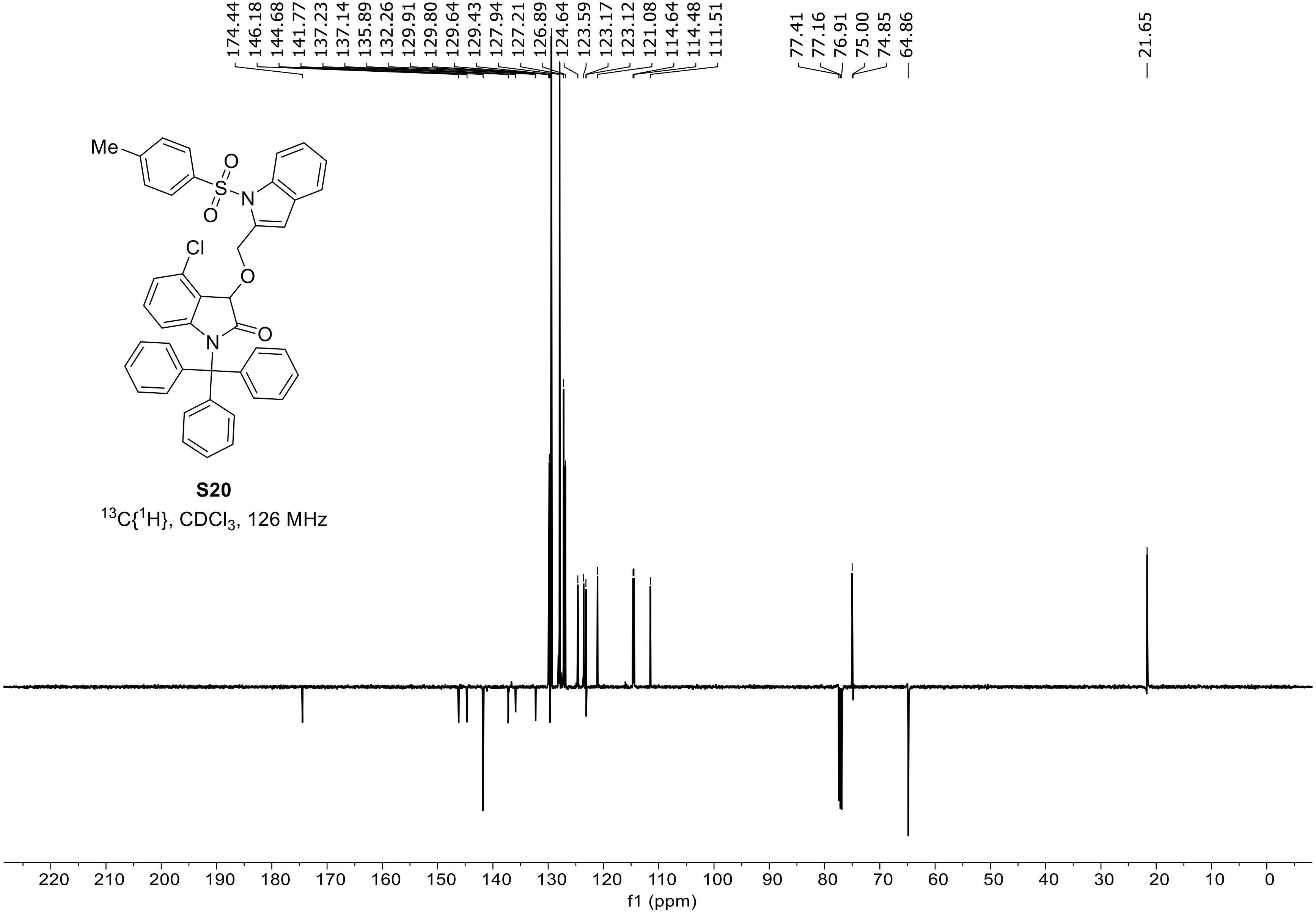
174.44
146.18
144.68
141.77
137.23
137.14
135.89
132.26
129.91
129.80
129.64
129.43
127.94
127.21
126.89
124.64
123.59
123.17
123.12
121.08
114.64
114.48
111.51
77.41
77.16
76.91
75.00
74.85
64.86
21.65
Me
O
S
N
O
Cl
O
O
N
S20
$^{13}C\{^{1}H\}$, $CDCl_3$, 126 MHz
220 210 200 190 180 170 160 150 140 130 120 110 100 90 80 70 60 50 40 30 20 10 0
f1 (ppm)

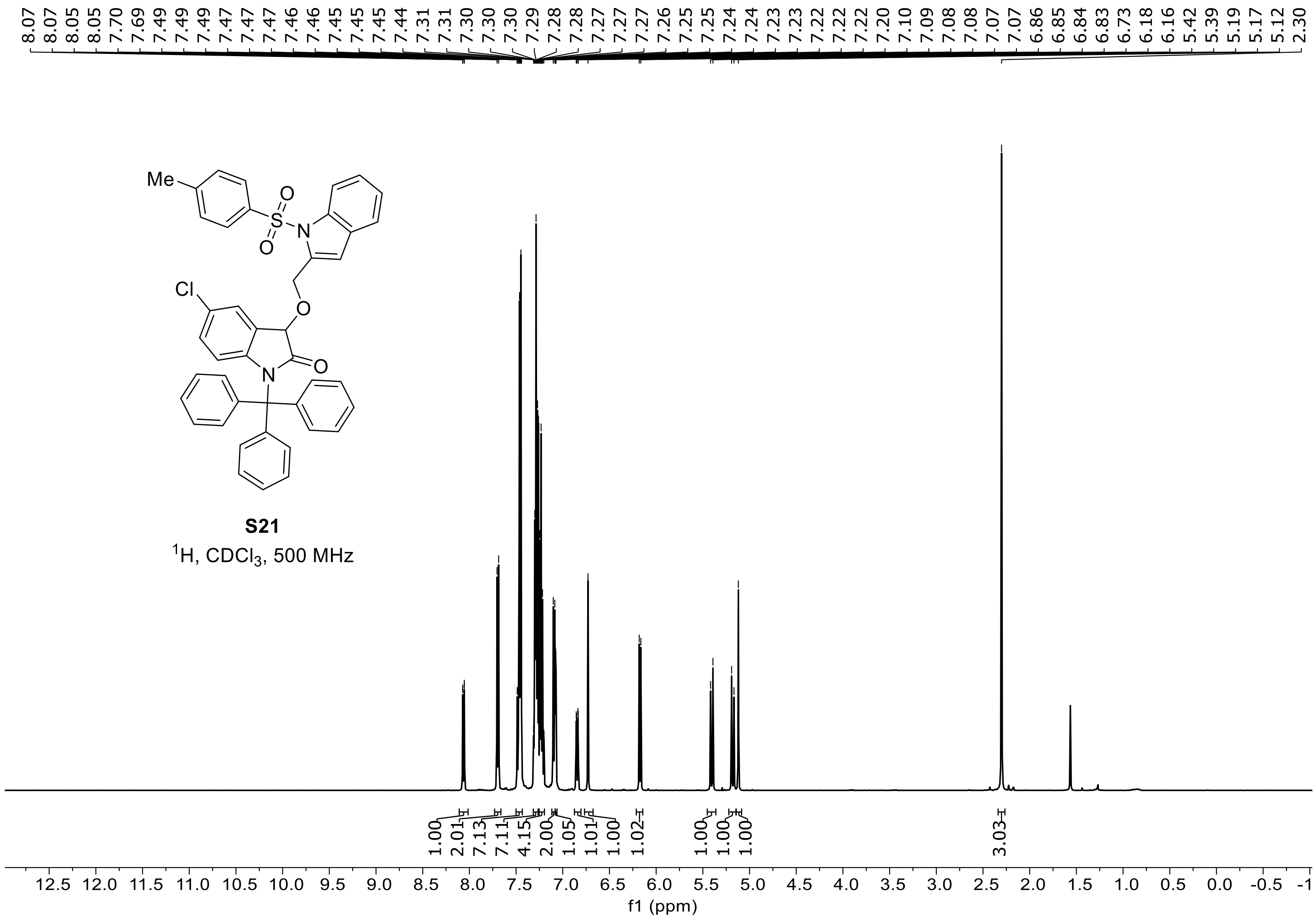
8.07 8.07 8.05 8.05 7.70 7.69 7.49 7.49 7.49 7.47 7.47 7.47 7.46 7.46 7.45 7.45 7.45 7.44 7.31 7.31 7.30 7.30 7.30 7.29 7.28 7.28 7.27 7.27 7.27 7.26 7.25 7.25 7.24 7.24 7.23 7.23 7.22 7.22 7.22 7.20 7.10 7.09 7.08 7.08 7.07 7.07 6.86 6.85 6.84 6.83 6.73 6.18 6.16 5.42 5.39 5.19 5.17 5.12 2.30
Me
O
S
O
N
Cl
O
N
O
S21
^{1}H, $CDCl_3$, 500 MHz
1.00 2.01 7.13 7.11 4.15 2.00 1.05 1.01 1.00 1.02 1.00 1.00 1.00 3.03
12.5 12.0 11.5 11.0 10.5 10.0 9.5 9.0 8.5 8.0 7.5 7.0 6.5 6.0 5.5 5.0 4.5 4.0 3.5 3.0 2.5 2.0 1.5 1.0 0.5 0.0 -0.5 -1
f1 (ppm)

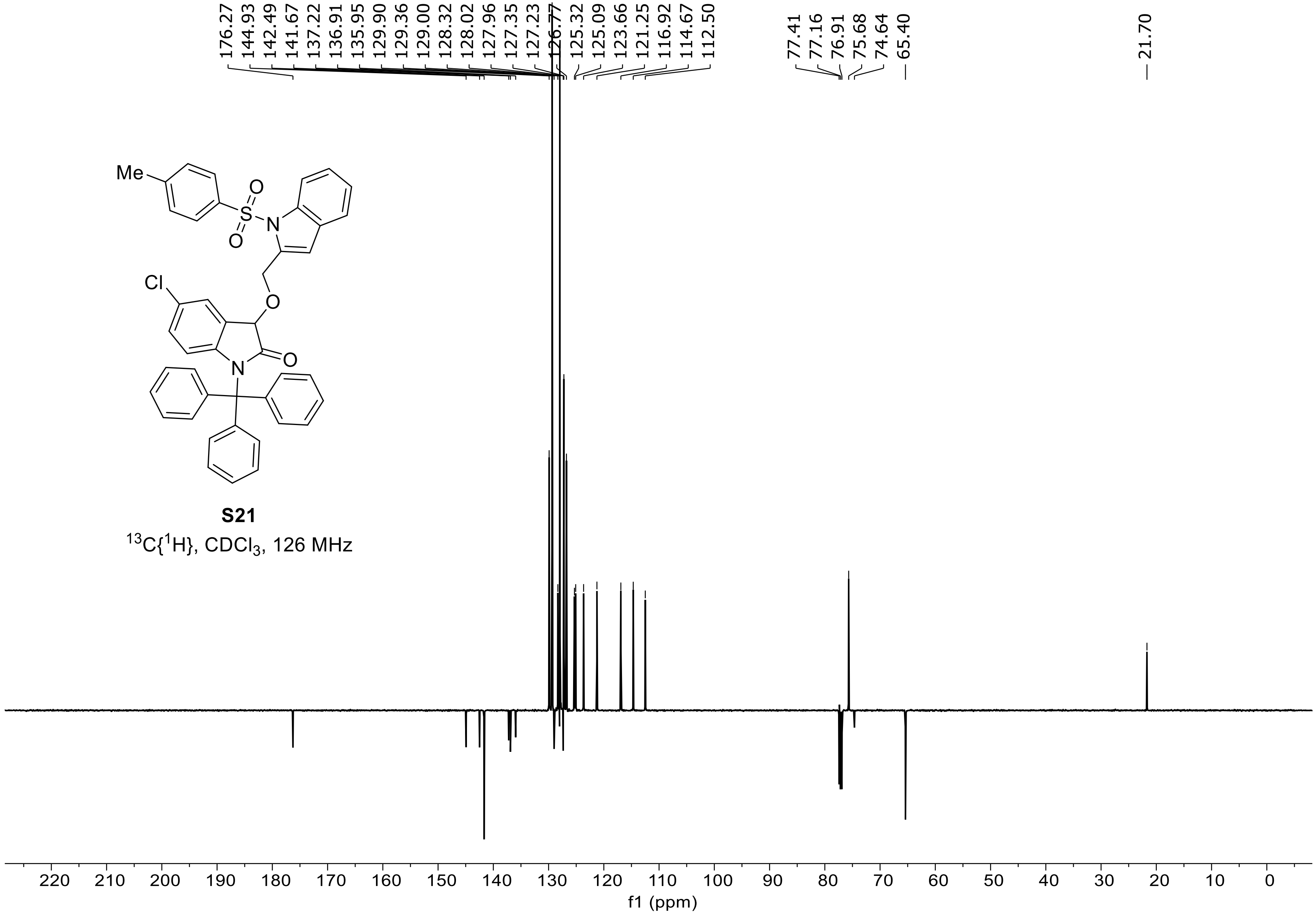
176.27
144.93
142.49
141.67
137.22
136.91
135.95
129.90
129.36
129.00
128.32
128.02
127.96
127.35
127.23
126.77
125.32
125.09
123.66
121.25
116.92
114.67
112.50
77.41
77.16
76.91
75.68
74.64
65.40
21.70
Me
O
S
N
O
Cl
O
N
O
S21
$^{13}C\{^1H\}$, $CDCl_3$, 126 MHz
220 210 200 190 180 170 160 150 140 130 120 110 100 90 80 70 60 50 40 30 20 10 0
f1 (ppm)

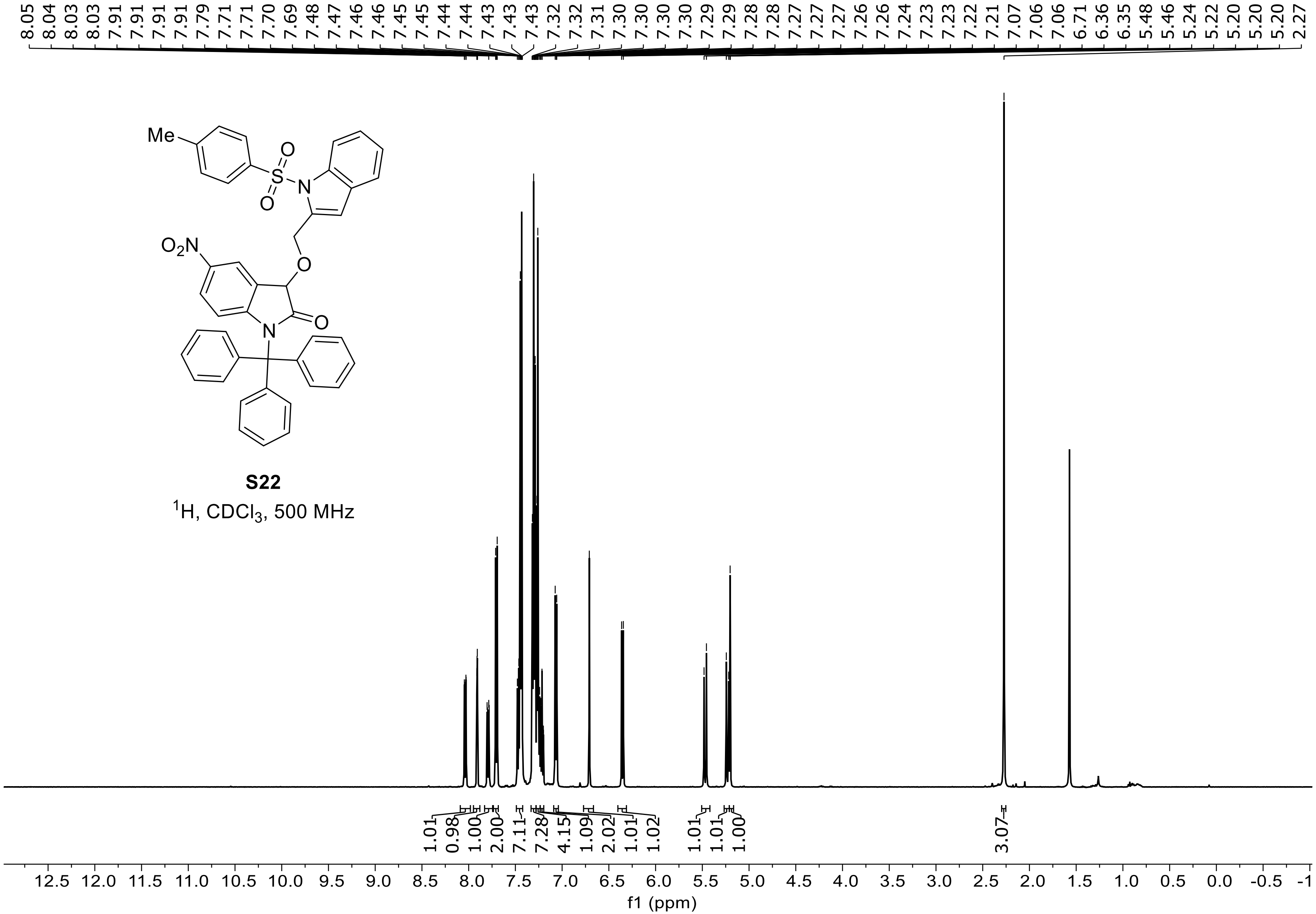
8.05 8.04 8.03 8.03 7.91 7.91 7.91 7.91 7.79 7.71 7.71 7.70 7.69 7.48 7.47 7.46 7.46 7.45 7.45 7.44 7.44 7.43 7.43 7.43 7.32 7.32 7.31 7.30 7.30 7.30 7.30 7.29 7.29 7.28 7.28 7.27 7.27 7.27 7.26 7.26 7.24 7.23 7.23 7.22 7.21 7.07 7.06 7.06 6.71 6.36 6.35 5.48 5.46 5.24 5.22 5.20 5.20 5.20 2.27
Me
O
S
O
N
O
O_2N
O
N
S22
^{1}H, $CDCl_3$, 500 MHz
1.01 0.98 1.00 2.00 7.11 7.28 4.15 1.09 2.02 1.01 1.02 1.01 1.01 1.00 3.07
12.5 12.0 11.5 11.0 10.5 10.0 9.5 9.0 8.5 8.0 7.5 7.0 6.5 6.0 5.5 5.0 4.5 4.0 3.5 3.0 2.5 2.0 1.5 1.0 0.5 0.0 -0.5 -1
f1 (ppm)

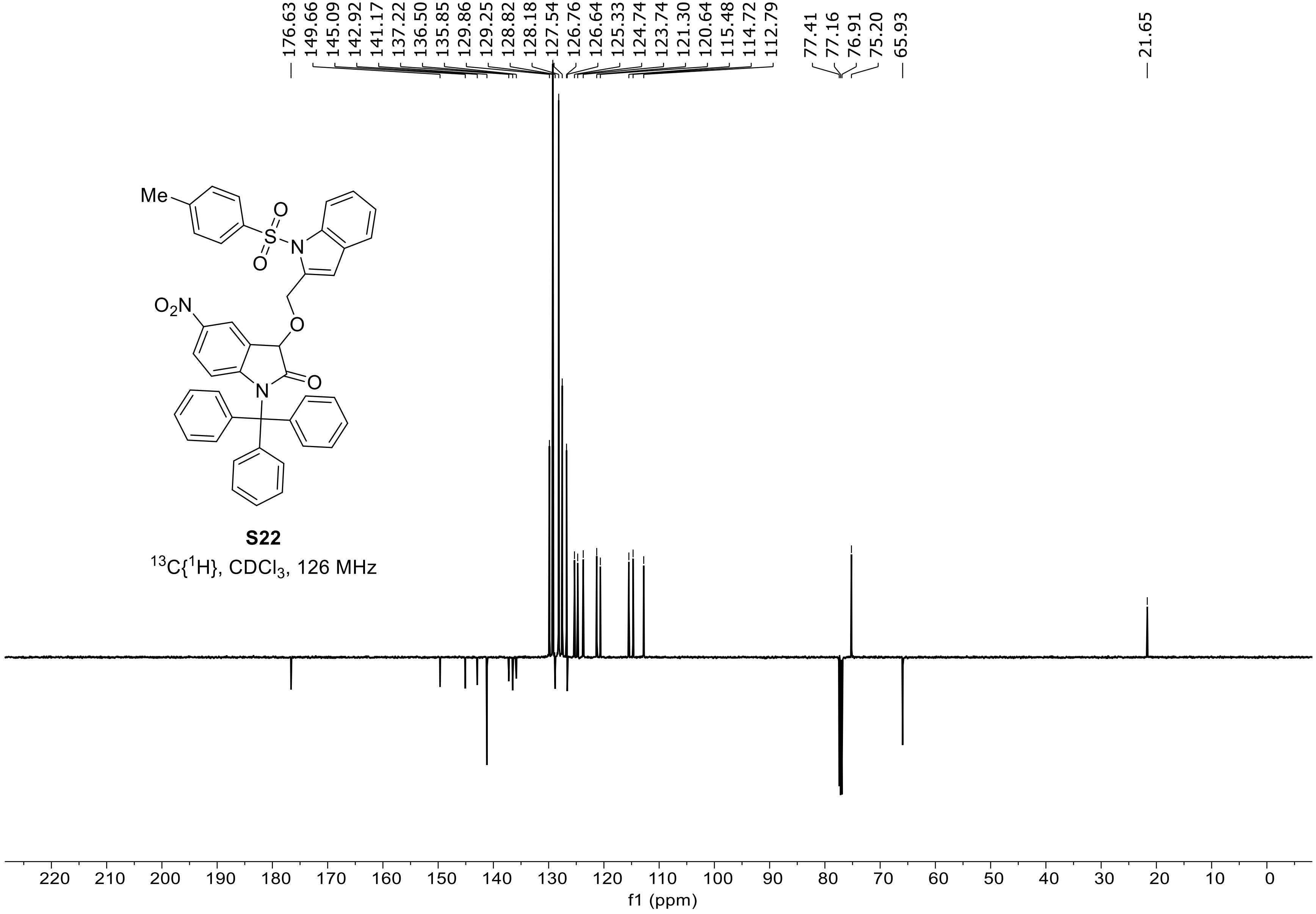
176.63
149.66
145.09
142.92
141.17
137.22
136.50
135.85
129.86
129.25
128.82
128.18
127.54
126.76
126.64
125.33
124.74
123.74
121.30
120.64
115.48
114.72
112.79
77.41
77.16
76.91
75.20
65.93
21.65
Me
O
S
O
N
O_2N
O
N
O
S22
$^{13}C\{^1H\}$, $CDCl_3$, 126 MHz
220 210 200 190 180 170 160 150 140 130 120 110 100 90 80 70 60 50 40 30 20 10 0
f1 (ppm)

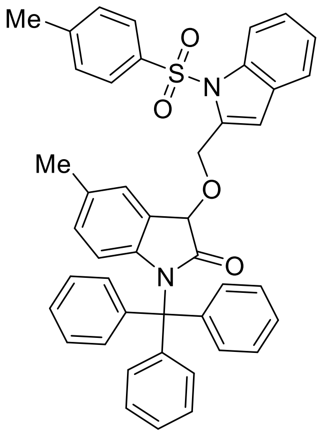

S23

^{1}H, $CDCl_3$, 500 MHz

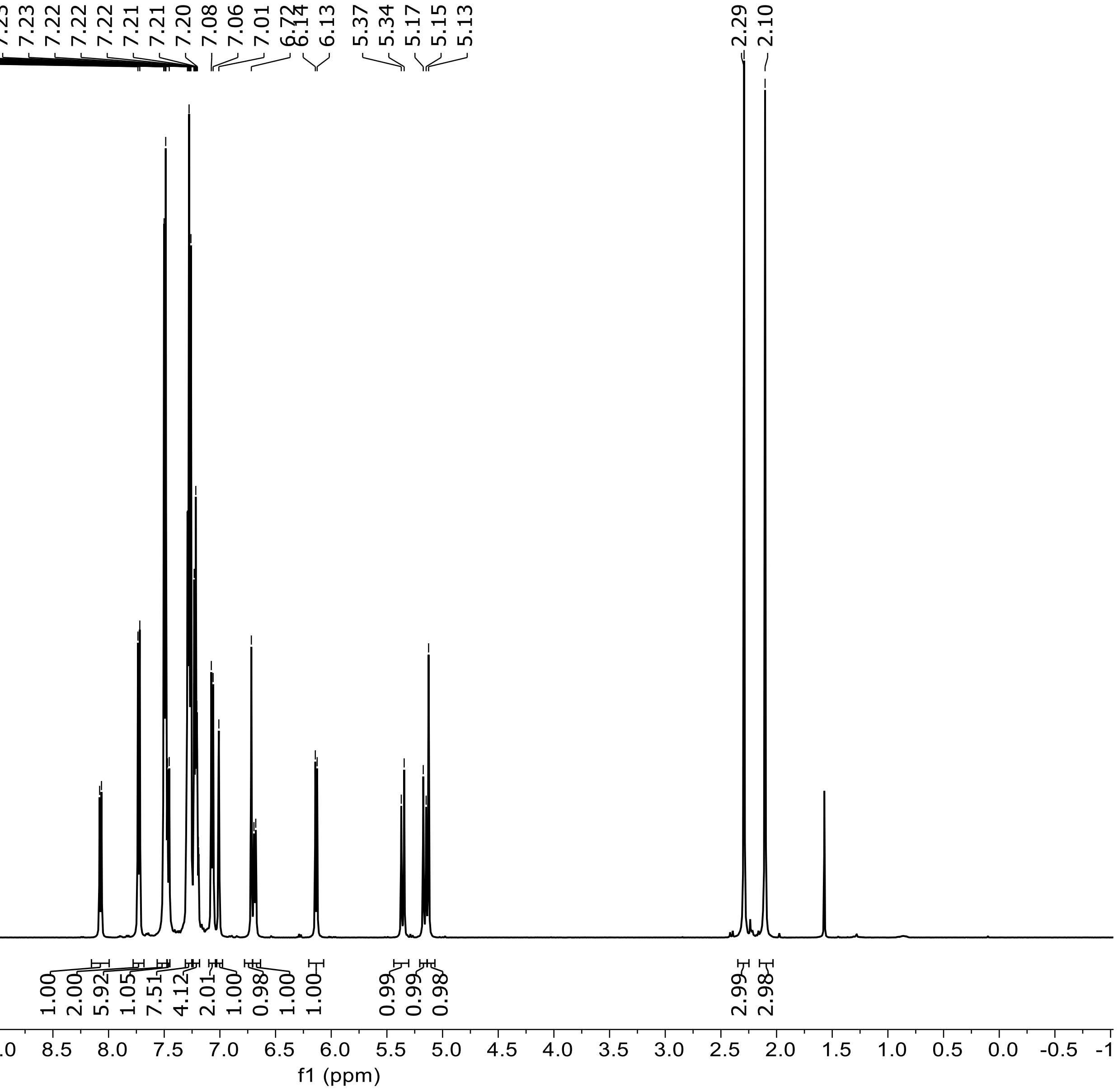

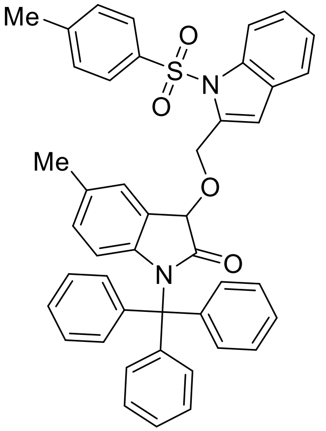

S23

$^{13}C\{^{1}H\}$, $CDCl_3$, 126 MHz

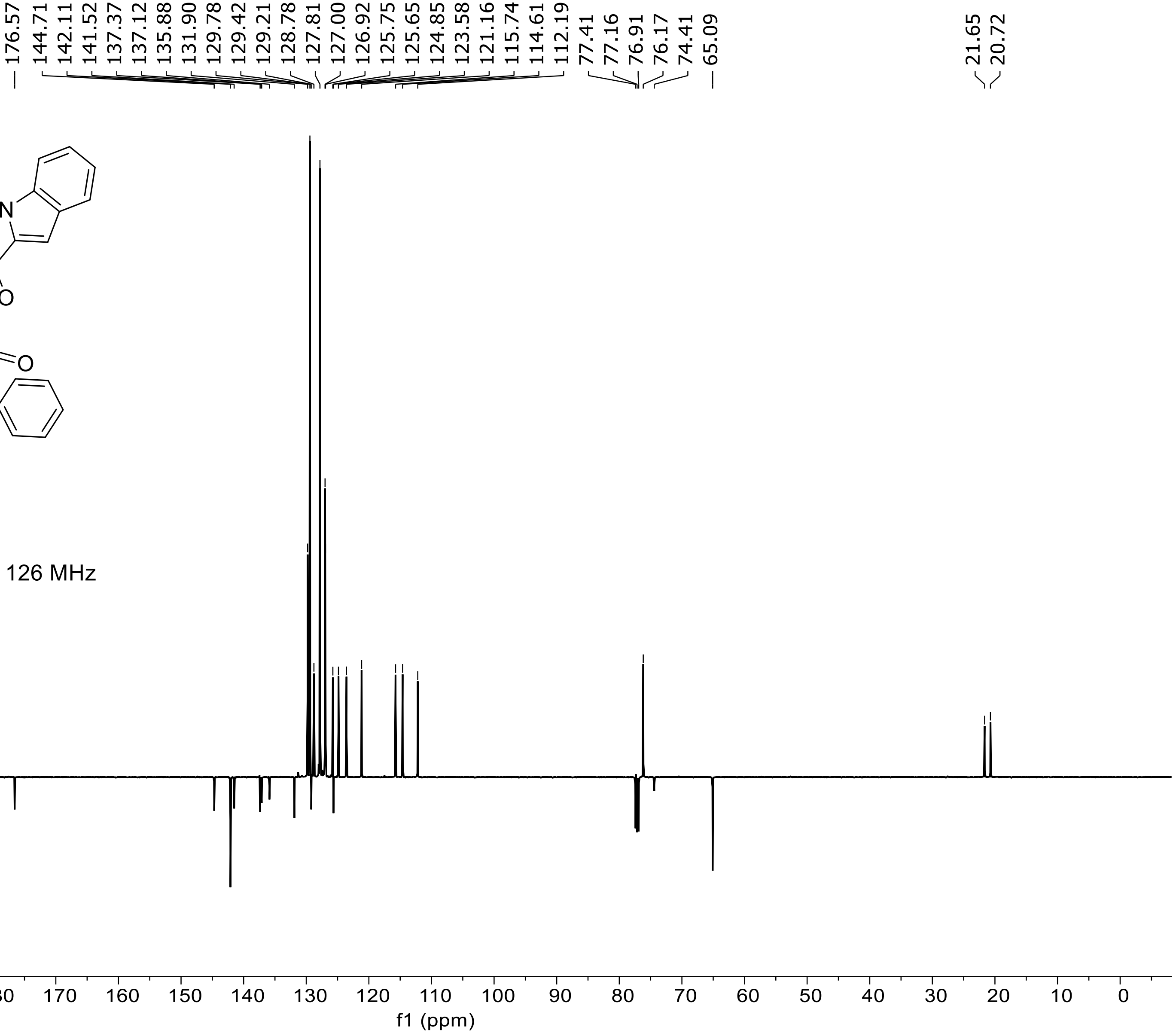

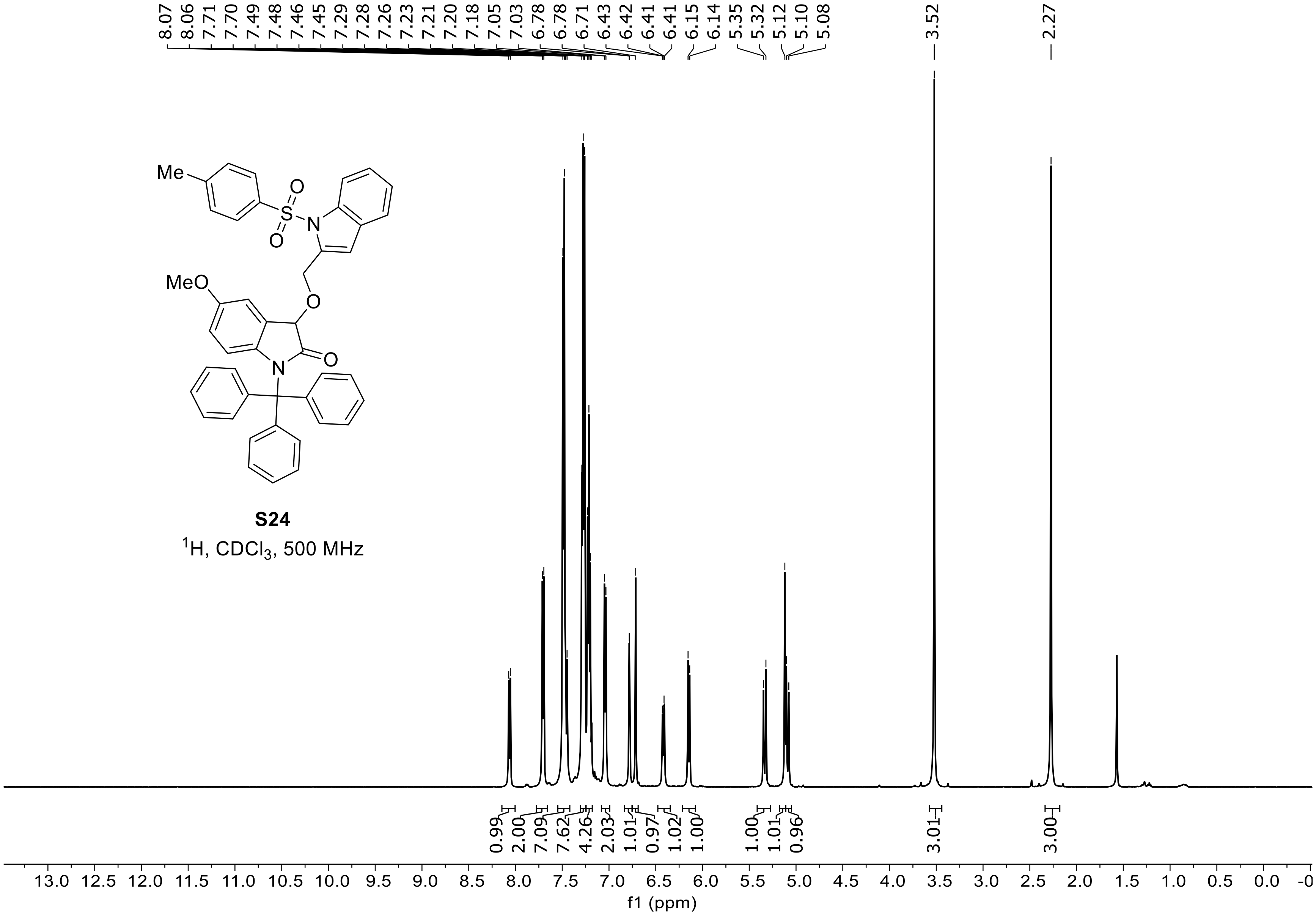
8.07
8.06
7.71
7.70
7.49
7.48
7.46
7.45
7.29
7.28
7.26
7.23
7.21
7.20
7.18
7.05
7.03
6.78
6.78
6.71
6.43
6.42
6.41
6.41
6.15
6.14
5.35
5.32
5.12
5.10
5.08
3.52
2.27
Me
O
O
S
N
MeO
O
N
O
S24
1H, CDCl3, 500 MHz
0.99
2.00
7.09
7.62
4.26
2.03
1.01
0.97
1.02
1.00
1.00
1.01
0.96
3.01
3.00
13.0
12.5
12.0
11.5
11.0
10.5
10.0
9.5
9.0
8.5
8.0
7.5
7.0
6.5
6.0
5.5
5.0
4.5
4.0
3.5
3.0
2.5
2.0
1.5
1.0
0.5
0.0
-0
f1 (ppm)

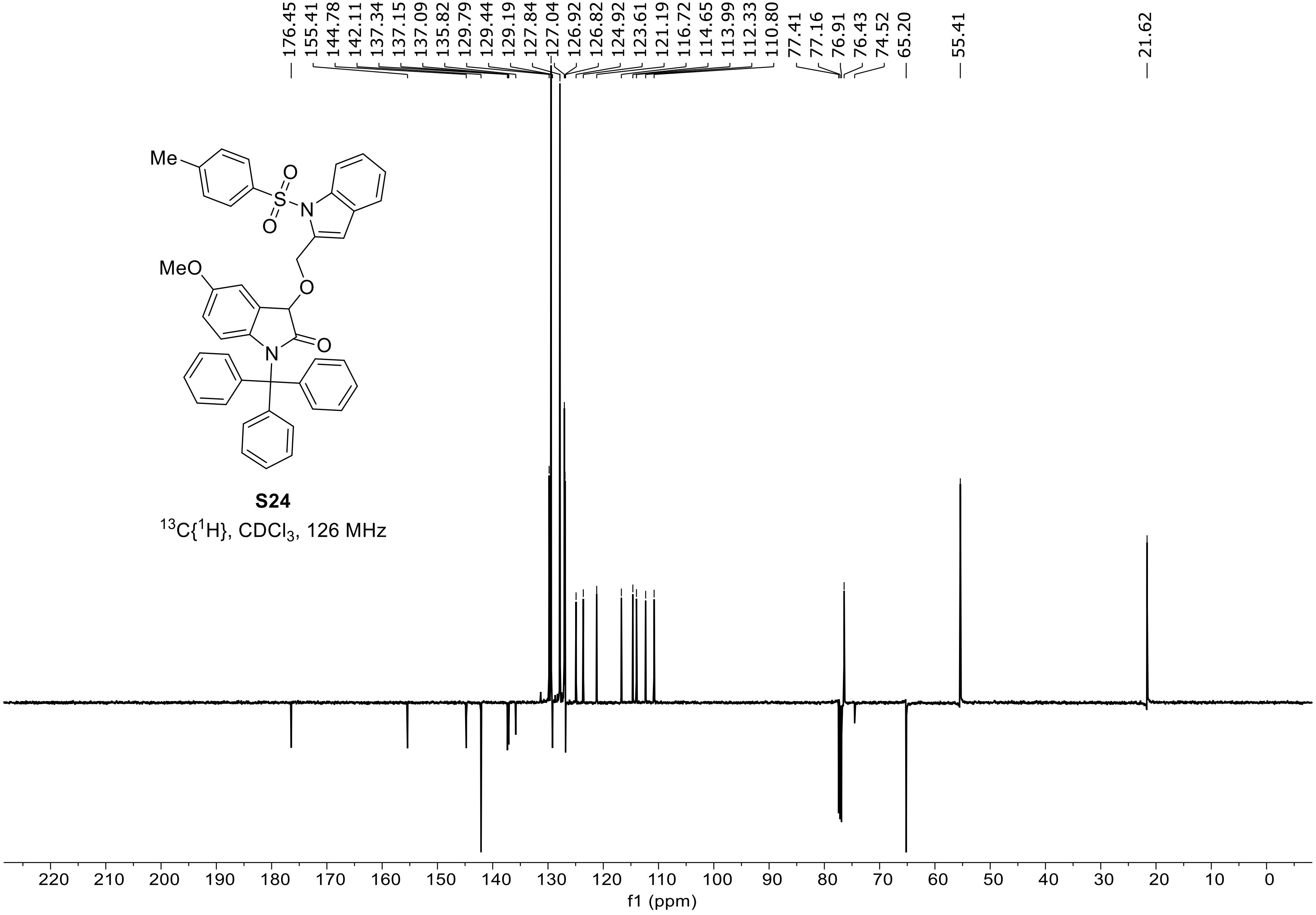

S24

$^{13}C\{^1H\}$, $CDCl_3$, 126 MHz

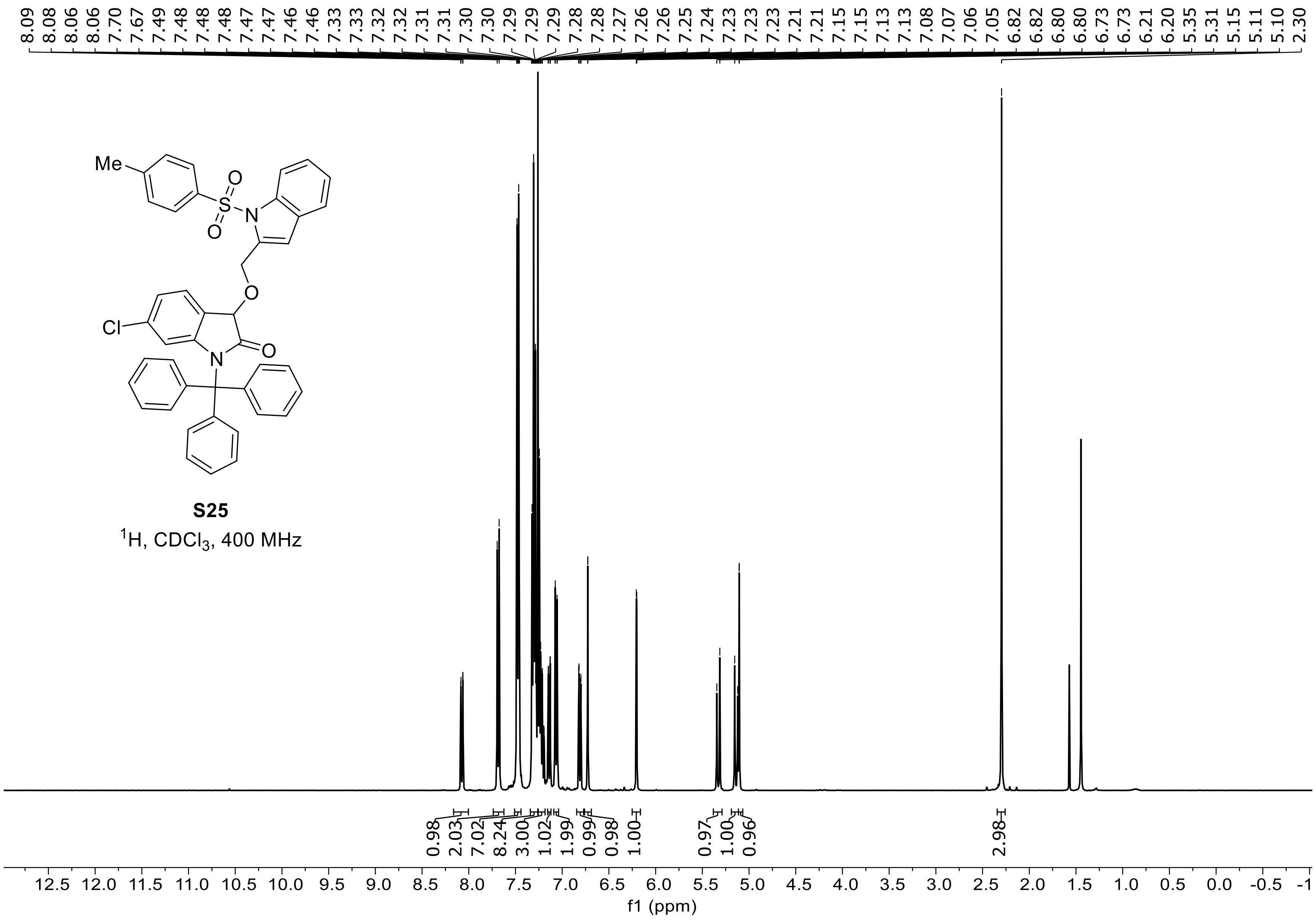
8.09 8.08 8.06 8.06 7.70 7.67 7.49 7.48 7.48 7.48 7.47 7.47 7.46 7.46 7.33 7.33 7.32 7.32 7.31 7.31 7.30 7.30 7.29 7.29 7.29 7.28 7.28 7.27 7.26 7.26 7.25 7.24 7.23 7.23 7.23 7.21 7.21 7.15 7.15 7.13 7.13 7.08 7.07 7.06 7.05 6.82 6.82 6.80 6.80 6.73 6.73 6.21 6.20 5.35 5.31 5.15 5.11 5.10 2.30
Me
O
S
O
N
O
Cl
O
N
S25
1H, CDCl3, 400 MHz
0.98 2.03 7.02 8.24 3.00 1.02 1.99 0.99 0.98 1.00 0.97 1.00 0.96 2.98
12.5 12.0 11.5 11.0 10.5 10.0 9.5 9.0 8.5 8.0 7.5 7.0 6.5 6.0 5.5 5.0 4.5 4.0 3.5 3.0 2.5 2.0 1.5 1.0 0.5 0.0 -0.5 -1
f1 (ppm)

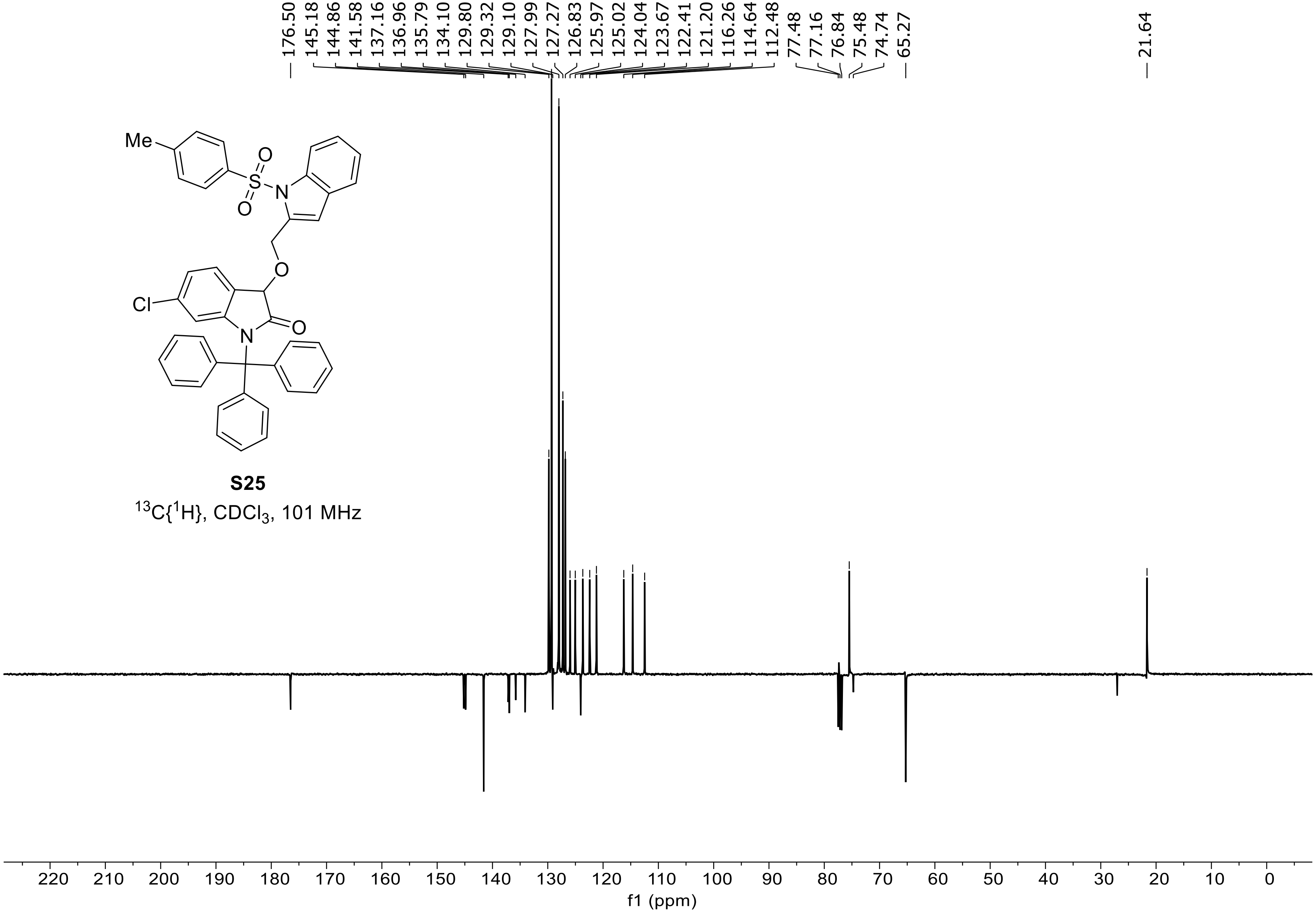
Me
O
S
O
N
O
Cl
N
O
S25
13C{1H}, CDCl3, 101 MHz
176.50
145.18
144.86
141.58
137.16
136.96
135.79
134.10
129.80
129.32
129.10
127.99
127.27
126.83
125.97
125.02
124.04
123.67
122.41
121.20
116.26
114.64
112.48
77.48
77.16
76.84
75.48
74.74
65.27
21.64
220 210 200 190 180 170 160 150 140 130 120 110 100 90 80 70 60 50 40 30 20 10 0
f1 (ppm)

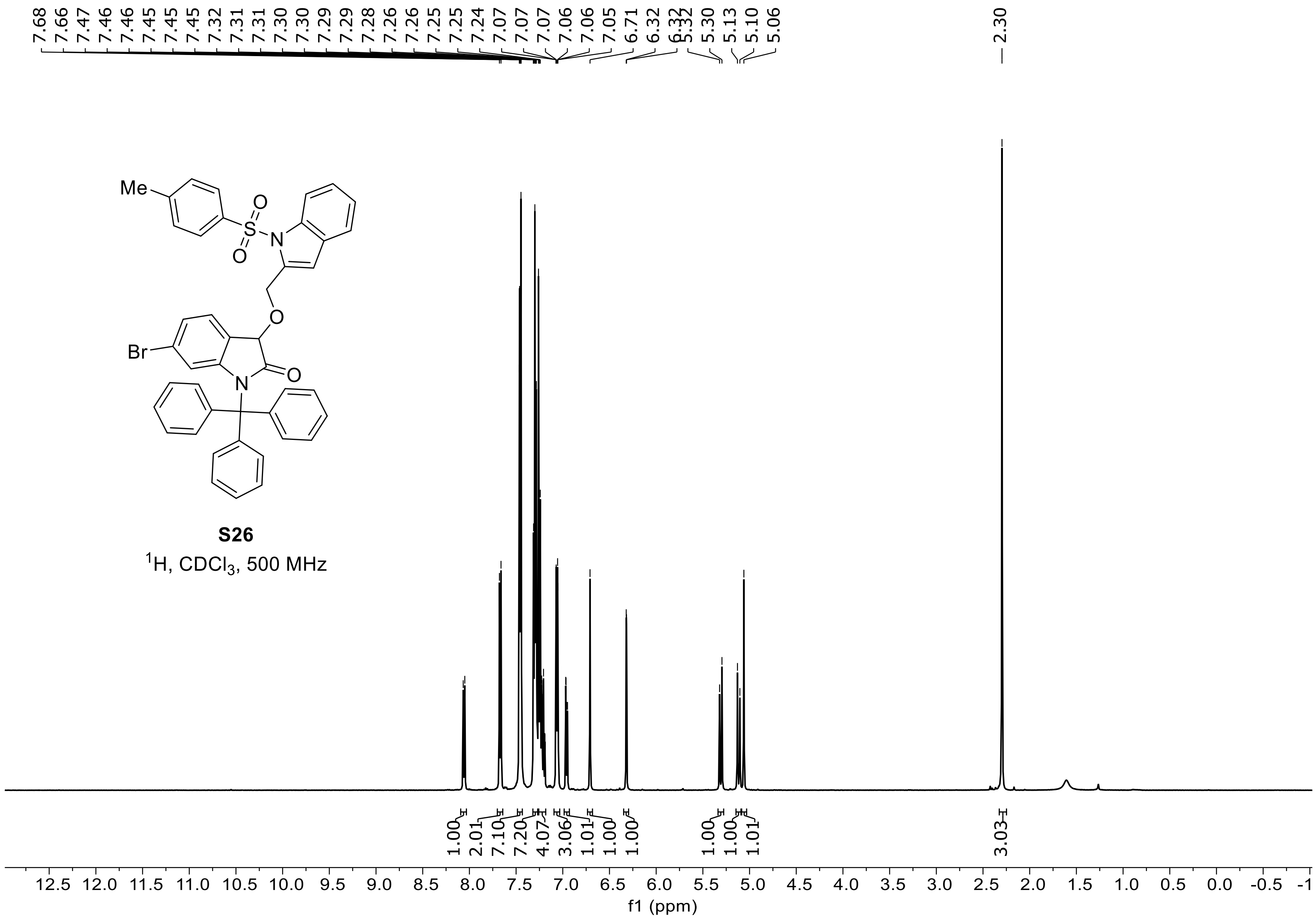
7.68 7.66 7.47 7.46 7.46 7.45 7.45 7.45 7.32 7.31 7.31 7.30 7.30 7.29 7.29 7.28 7.26 7.26 7.25 7.25 7.24 7.07 7.07 7.07 7.06 7.06 7.05 6.71 6.32 6.32 5.32 5.30 5.13 5.10 5.06
2.30
Me
O
S
O
N
O
Br
O
N
S26
^{1}H, CDCl$_3$, 500 MHz
1.00 2.01 7.10 7.20 4.07 3.06 1.01 1.00 1.00
1.00 1.00 1.01
3.03
12.5 12.0 11.5 11.0 10.5 10.0 9.5 9.0 8.5 8.0 7.5 7.0 6.5 6.0 5.5 5.0 4.5 4.0 3.5 3.0 2.5 2.0 1.5 1.0 0.5 0.0 -0.5 -1
f1 (ppm)

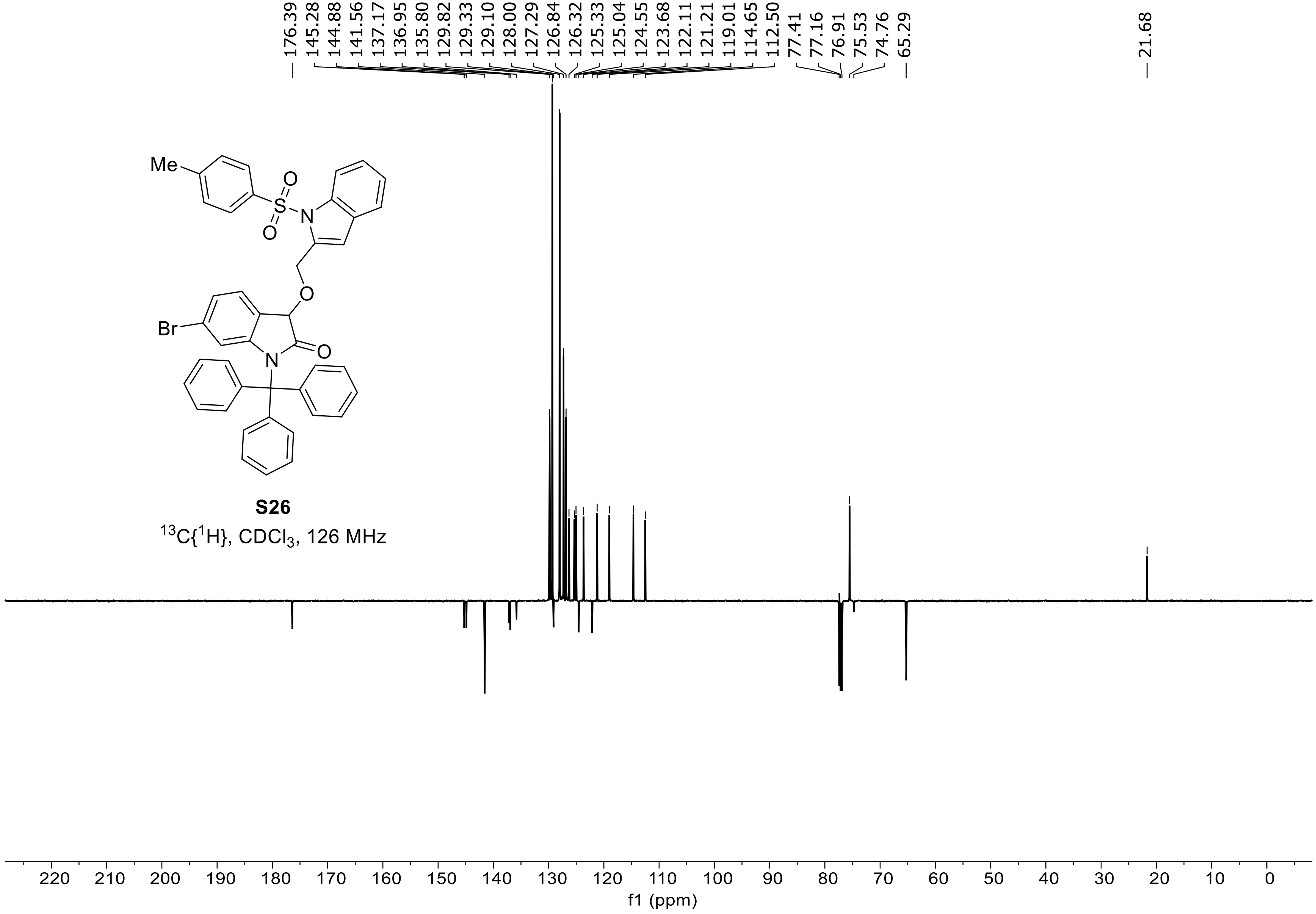
176.39
145.28
144.88
141.56
137.17
136.95
135.80
129.82
129.33
129.10
128.00
127.29
126.84
126.32
125.33
125.04
124.55
123.68
122.11
121.21
119.01
114.65
112.50
77.41
77.16
76.91
75.53
74.76
65.29
21.68
Me
O
S
N
O
O
Br
O
N
S26
$^{13}C\{^1H\}$, $CDCl_3$, 126 MHz
220 210 200 190 180 170 160 150 140 130 120 110 100 90 80 70 60 50 40 30 20 10 0
f1 (ppm)

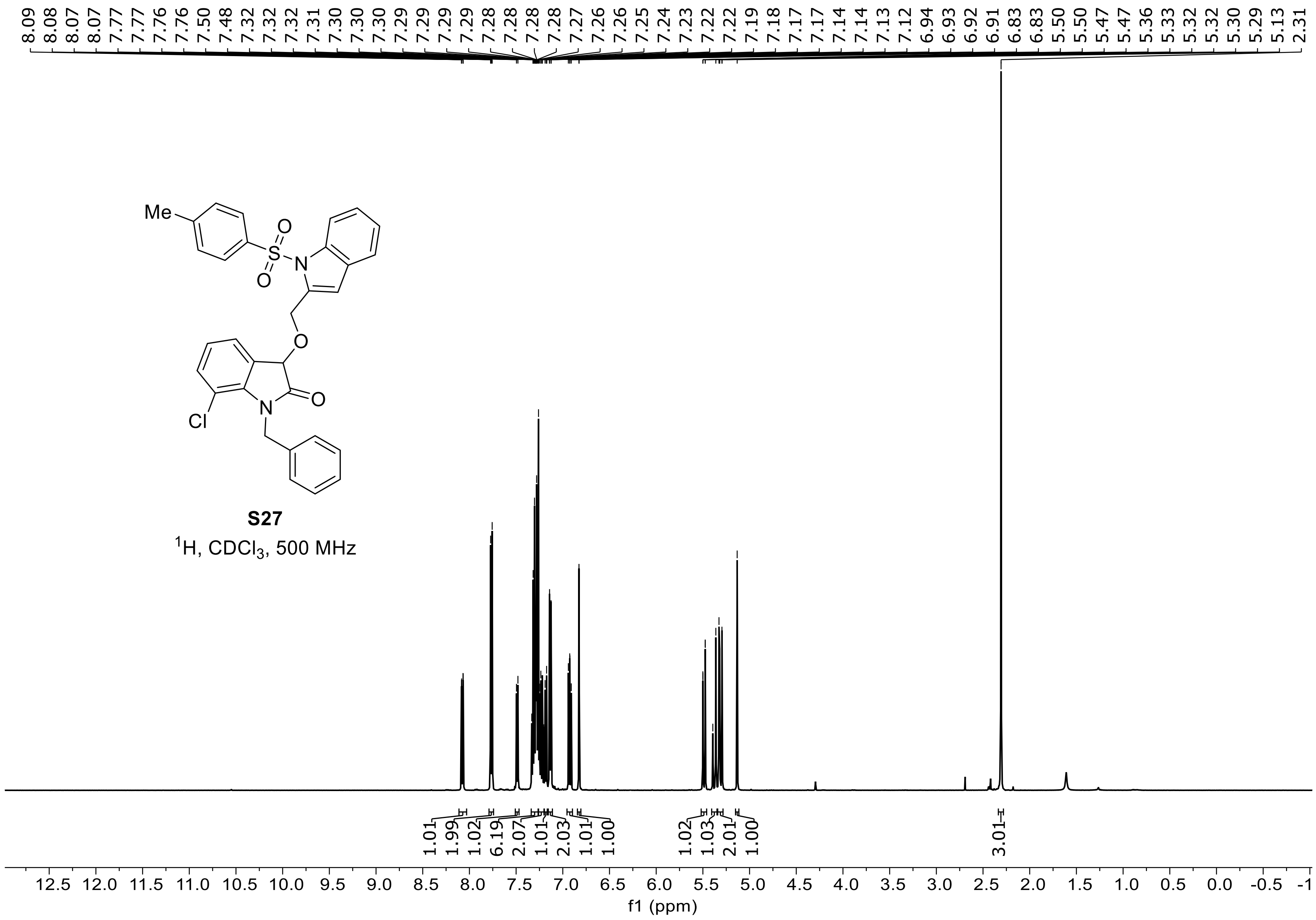

S27

^{1}H, $CDCl_3$, 500 MHz

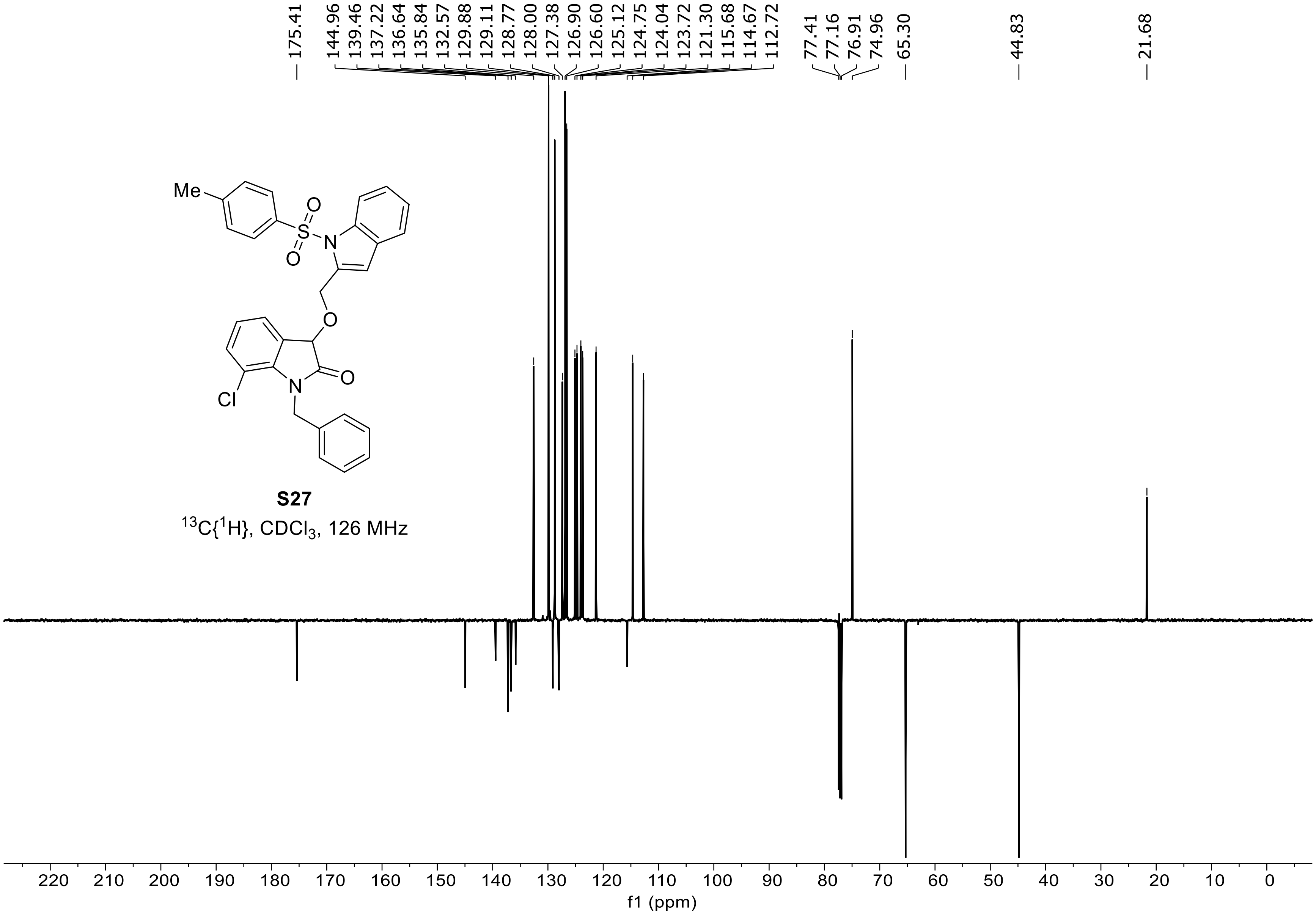
175.41
144.96
139.46
137.22
136.64
135.84
132.57
129.88
129.11
128.77
128.00
127.38
126.90
126.60
125.12
124.75
124.04
123.72
121.30
115.68
114.67
112.72
77.41
77.16
76.91
74.96
65.30
44.83
21.68
Me
O
S
O
N
O
O
N
Cl
S27
$^{13}C\{^1H\}$, $CDCl_3$, 126 MHz
220 210 200 190 180 170 160 150 140 130 120 110 100 90 80 70 60 50 40 30 20 10 0
f1 (ppm)

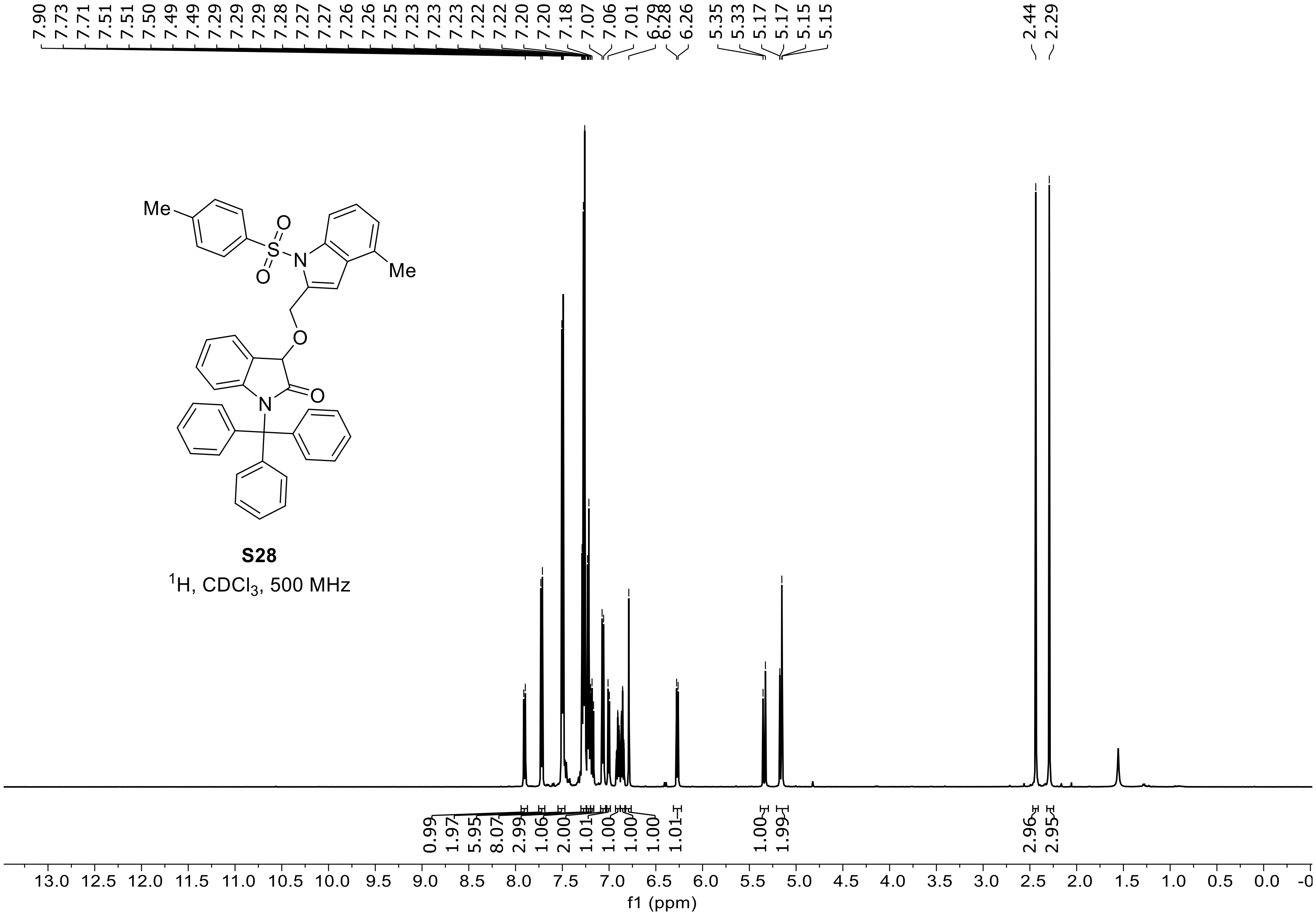

S28

^{1}H, $CDCl_3$, 500 MHz

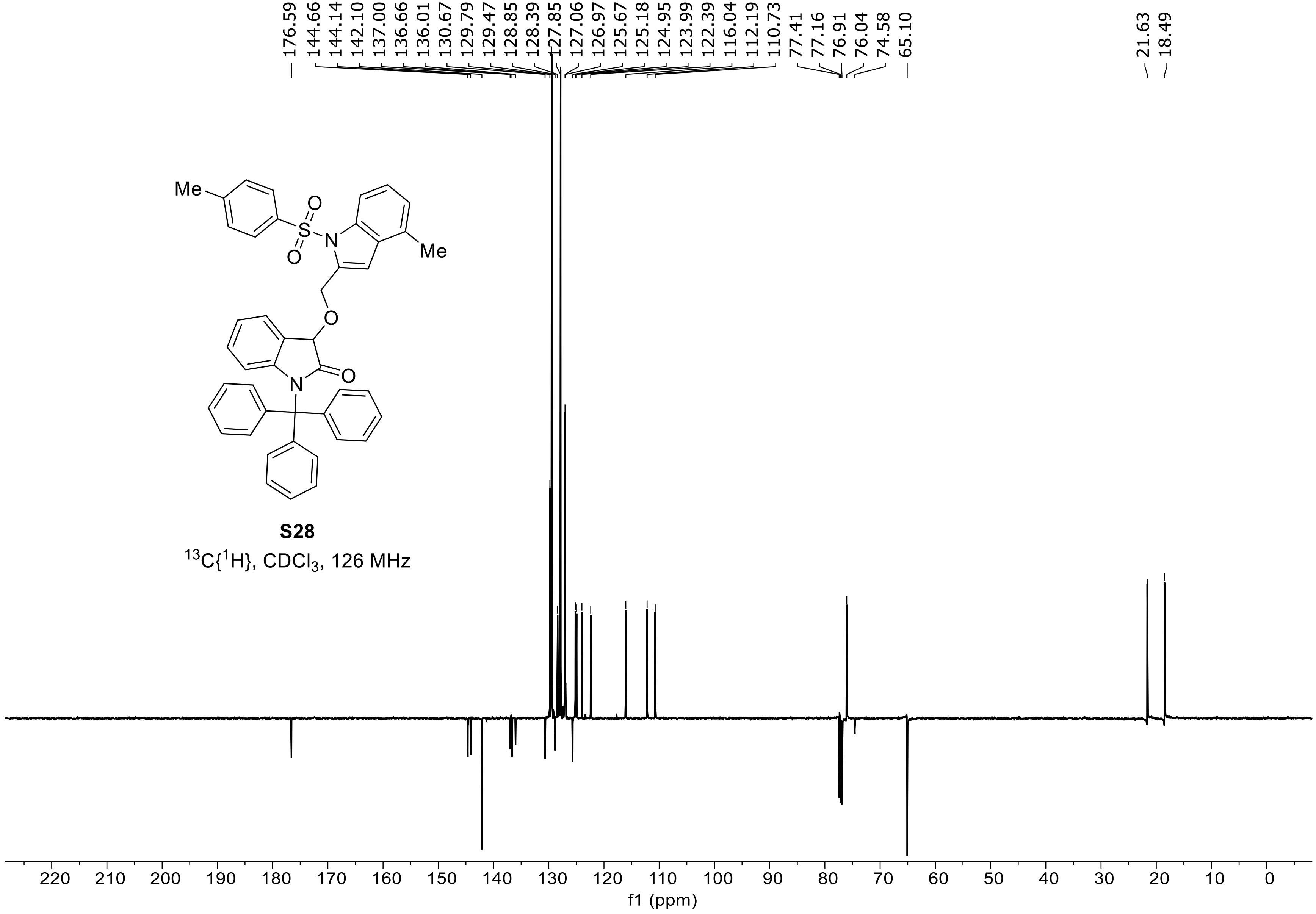

176.59
144.66
144.14
142.10
137.00
136.66
136.01
130.67
129.79
129.47
128.85
128.39
127.85
127.06
126.97
125.67
125.18
124.95
123.99
122.39
116.04
112.19
110.73
77.41
77.16
76.91
76.04
74.58
65.10
21.63
18.49
Me
O
S
O
N
Me
O
O
N
S28
$^{13}C\{^{1}H\}$, $CDCl_3$, 126 MHz
220
210
200
190
180
170
160
150
140
130
120
110
100
90
80
70
60
50
40
30
20
10
0
f1 (ppm)

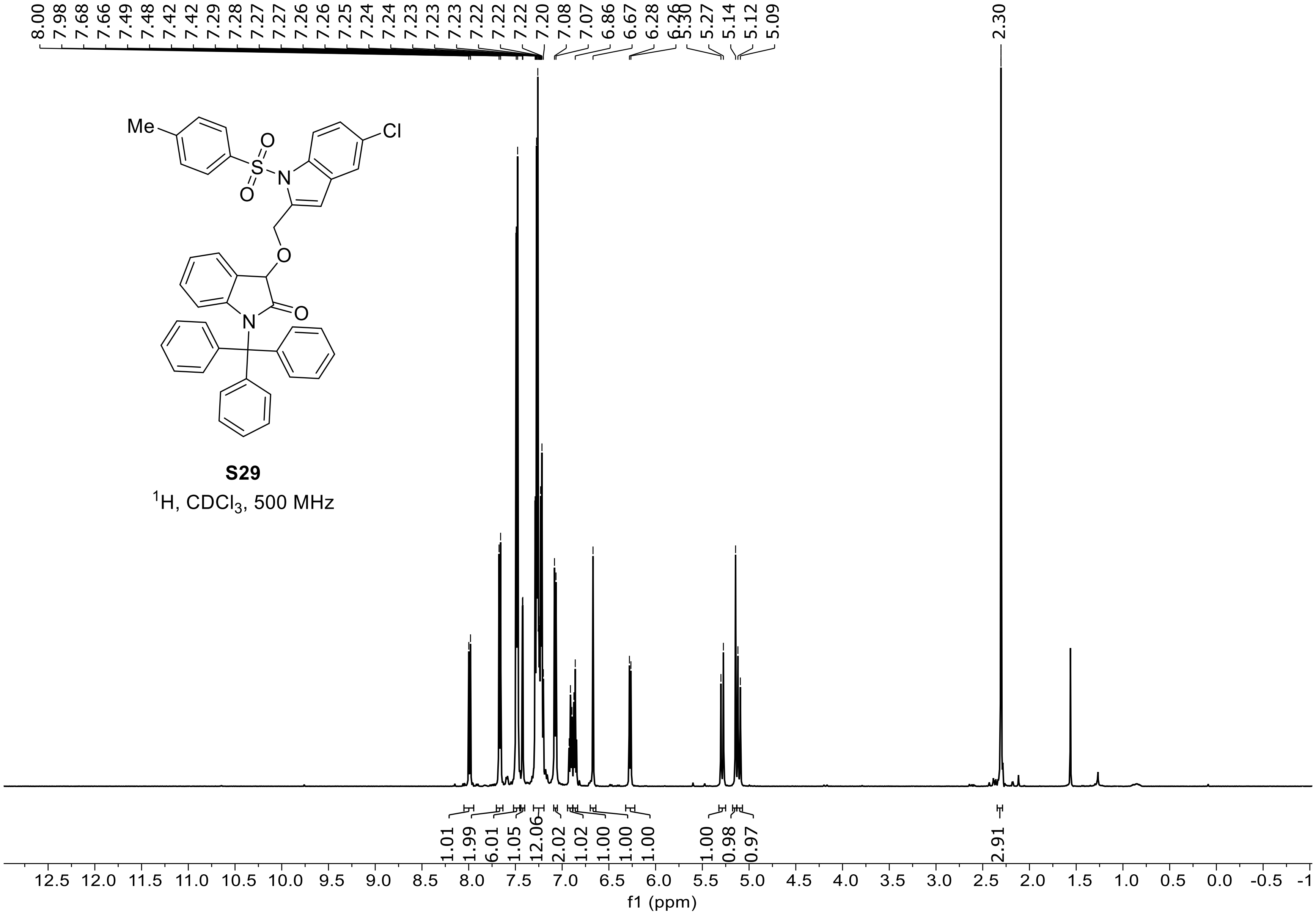

S29

^{1}H, $CDCl_3$, 500 MHz

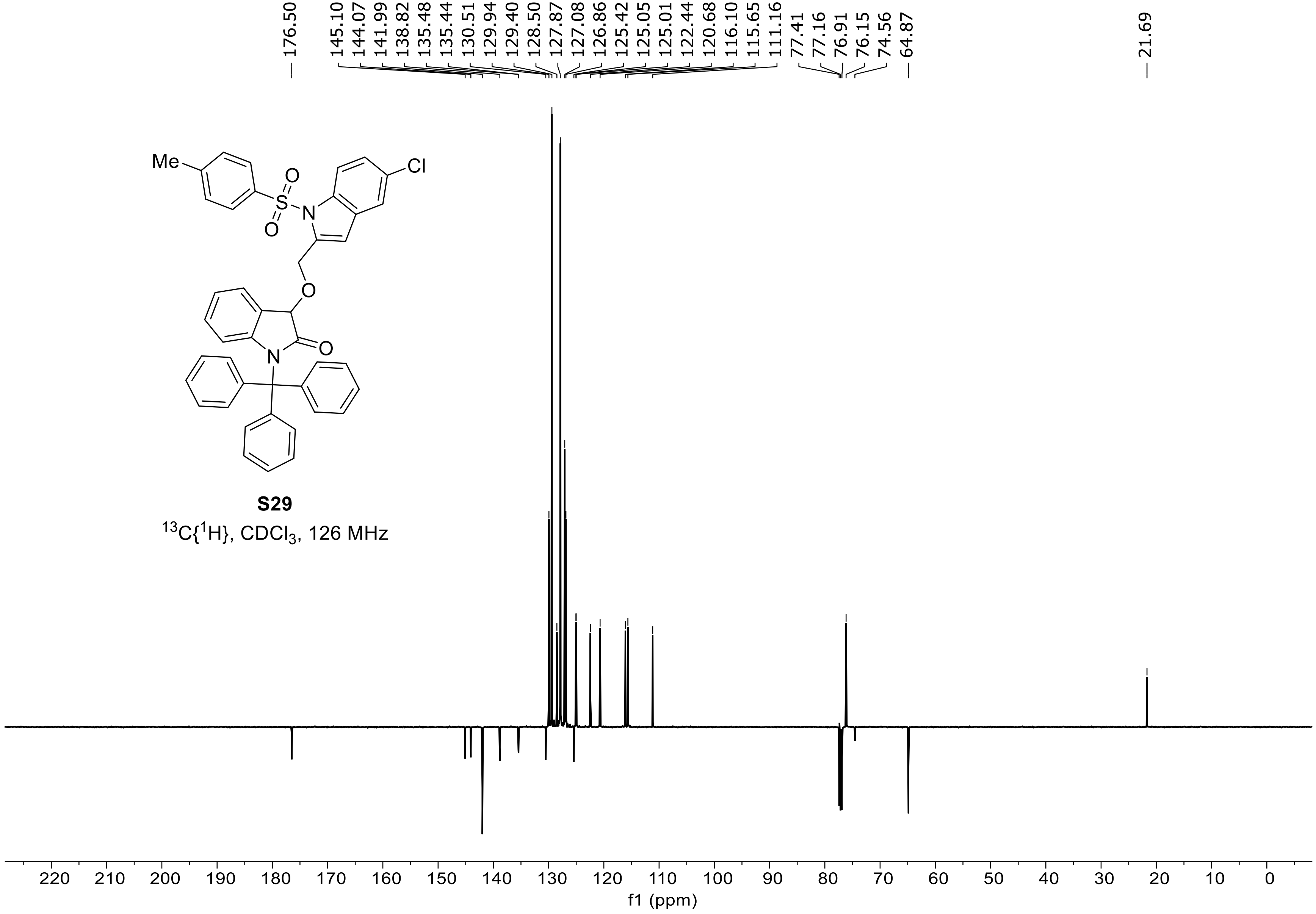
176.50
145.10
144.07
141.99
138.82
135.48
135.44
130.51
129.94
129.40
128.50
127.87
127.08
126.86
125.42
125.05
125.01
122.44
120.68
116.10
115.65
111.16
77.41
77.16
76.91
76.15
74.56
64.87
21.69
Me
Cl
S
O
N
S29
$^{13}C\{^1H\}$, $CDCl_3$, 126 MHz
220 210 200 190 180 170 160 150 140 130 120 110 100 90 80 70 60 50 40 30 20 10 0
f1 (ppm)

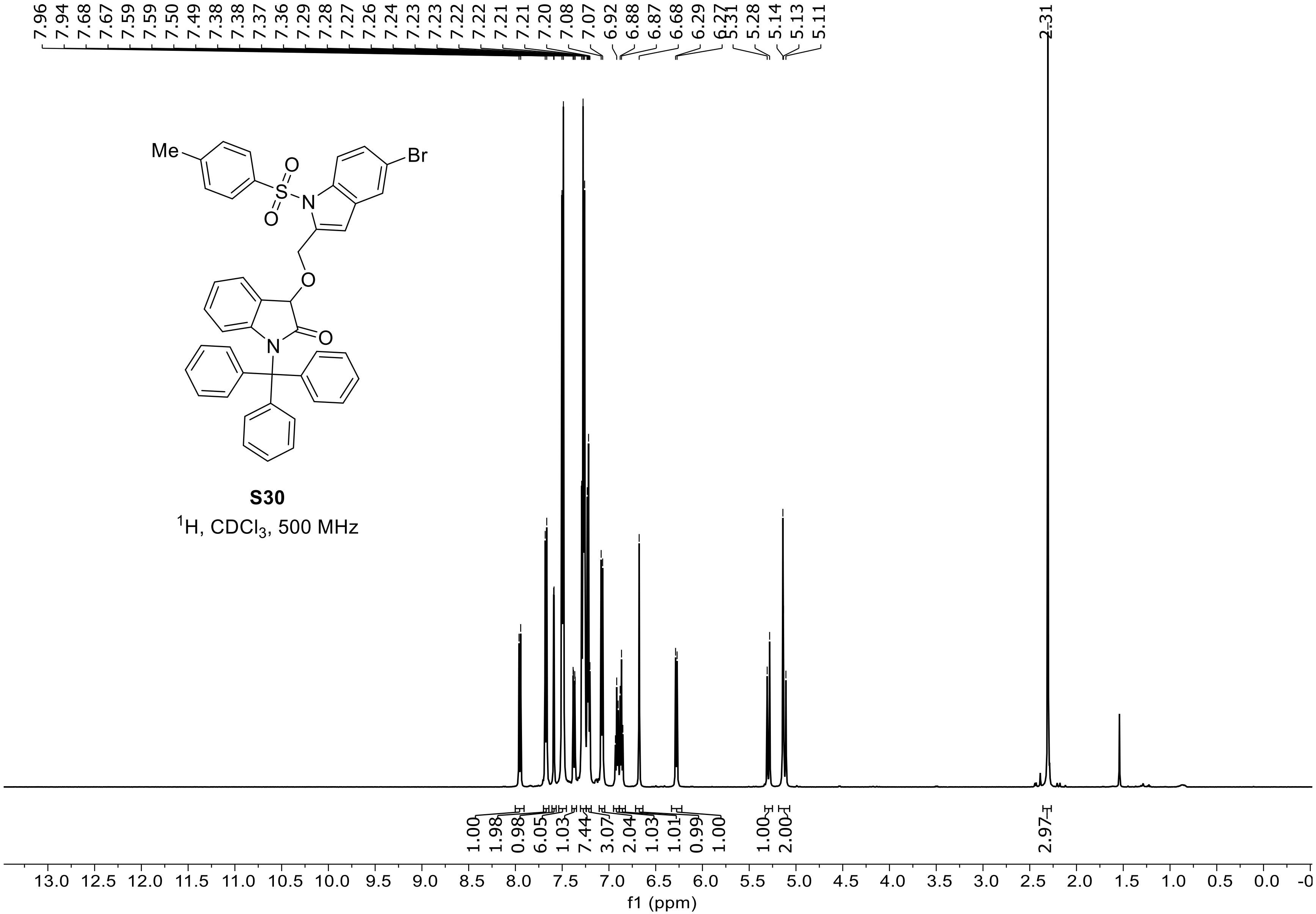
7.96 7.94 7.68 7.67 7.59 7.59 7.50 7.49 7.38 7.38 7.37 7.36 7.29 7.28 7.27 7.26 7.24 7.23 7.23 7.22 7.22 7.21 7.21 7.20 7.08 7.07 6.92 6.88 6.87 6.68 6.29 6.27 5.31 5.28 5.14 5.13 5.11
2.31
Me
Br
O
S
N
O
N
O
O
N
O
S30
^{1}H, CDCl$_3$, 500 MHz
1.00 1.98 0.98 6.05 1.03 7.44 3.07 2.04 1.03 1.01 0.99 1.00
1.00 2.00
2.97
13.0 12.5 12.0 11.5 11.0 10.5 10.0 9.5 9.0 8.5 8.0 7.5 7.0 6.5 6.0 5.5 5.0 4.5 4.0 3.5 3.0 2.5 2.0 1.5 1.0 0.5 0.0 -0
f1 (ppm)

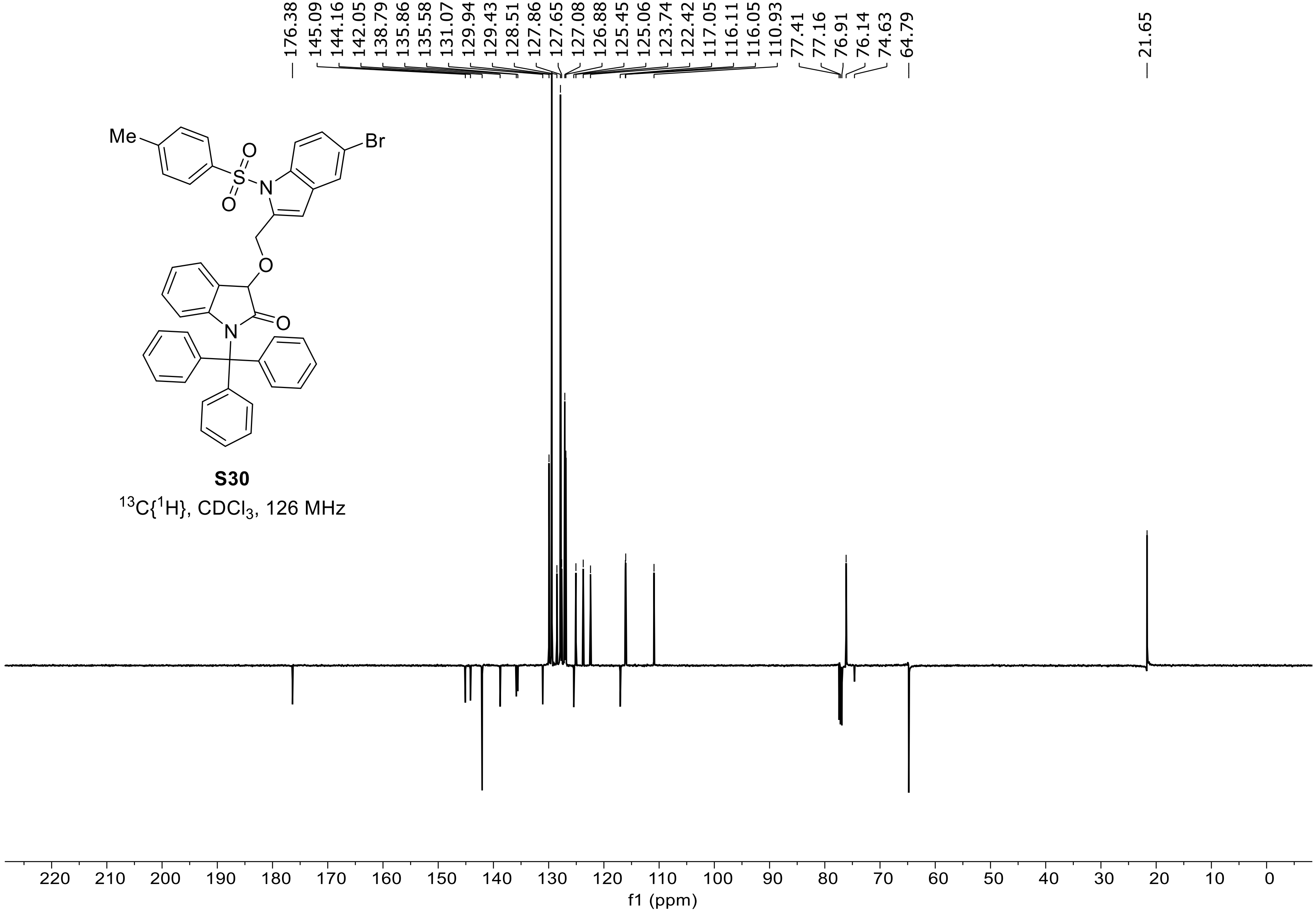

S30

$^{13}C\{^{1}H\}$, $CDCl_3$, 126 MHz

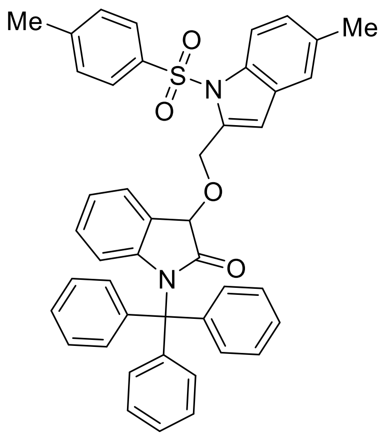

S31

^1H, $CDCl_3$, 500 MHz

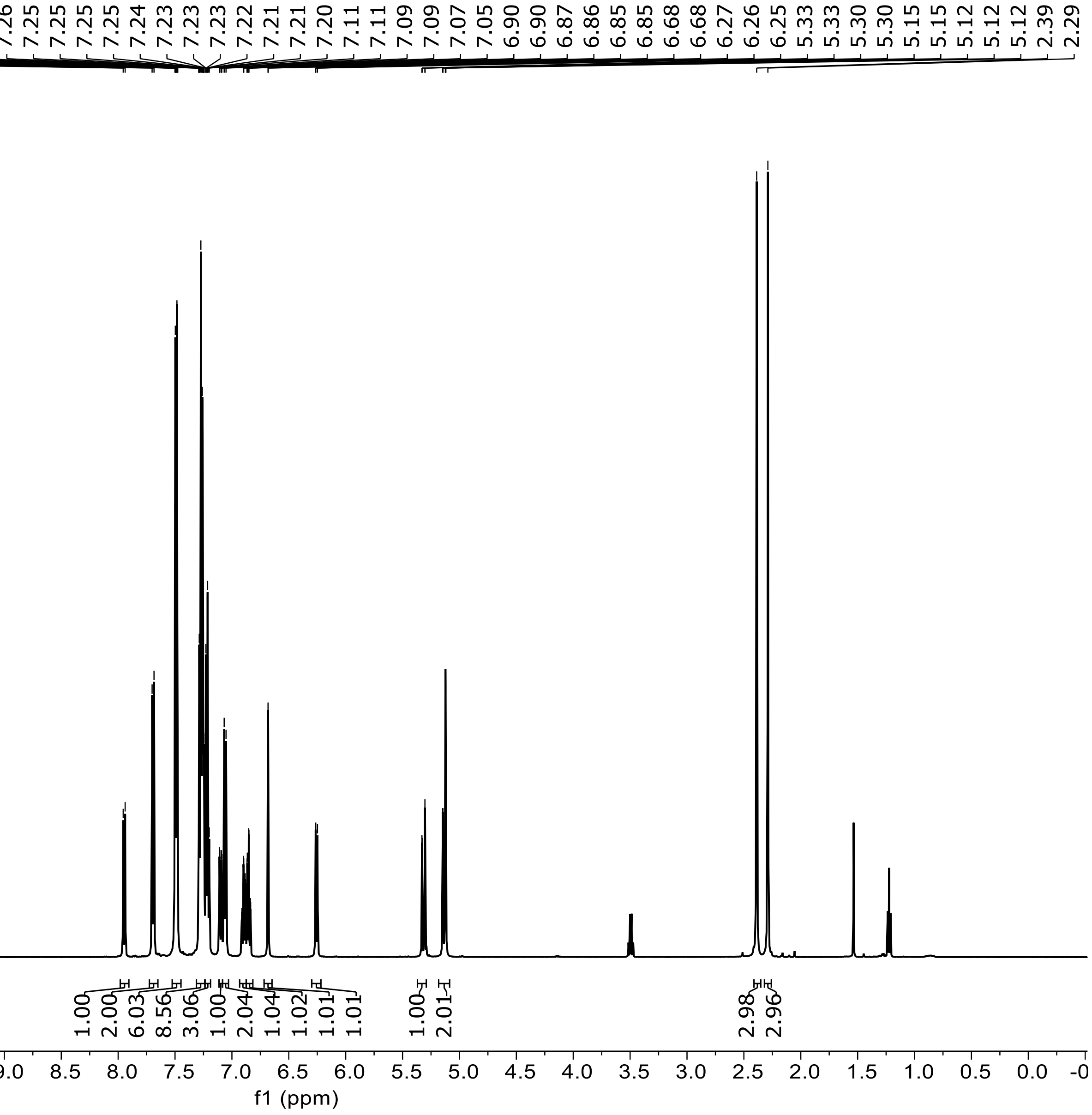

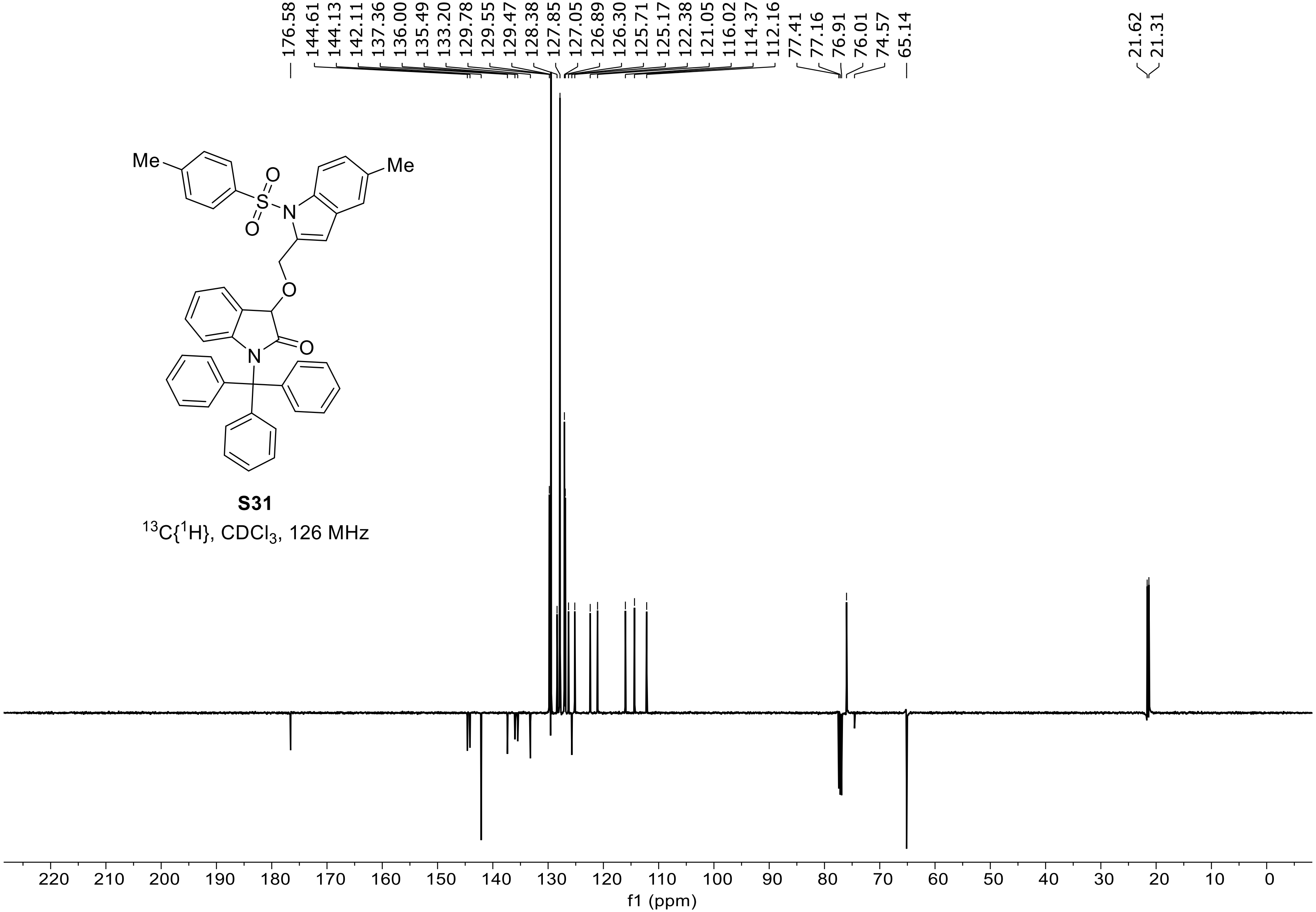

S31

$^{13}C\{^{1}H\}$, $CDCl_3$, 126 MHz

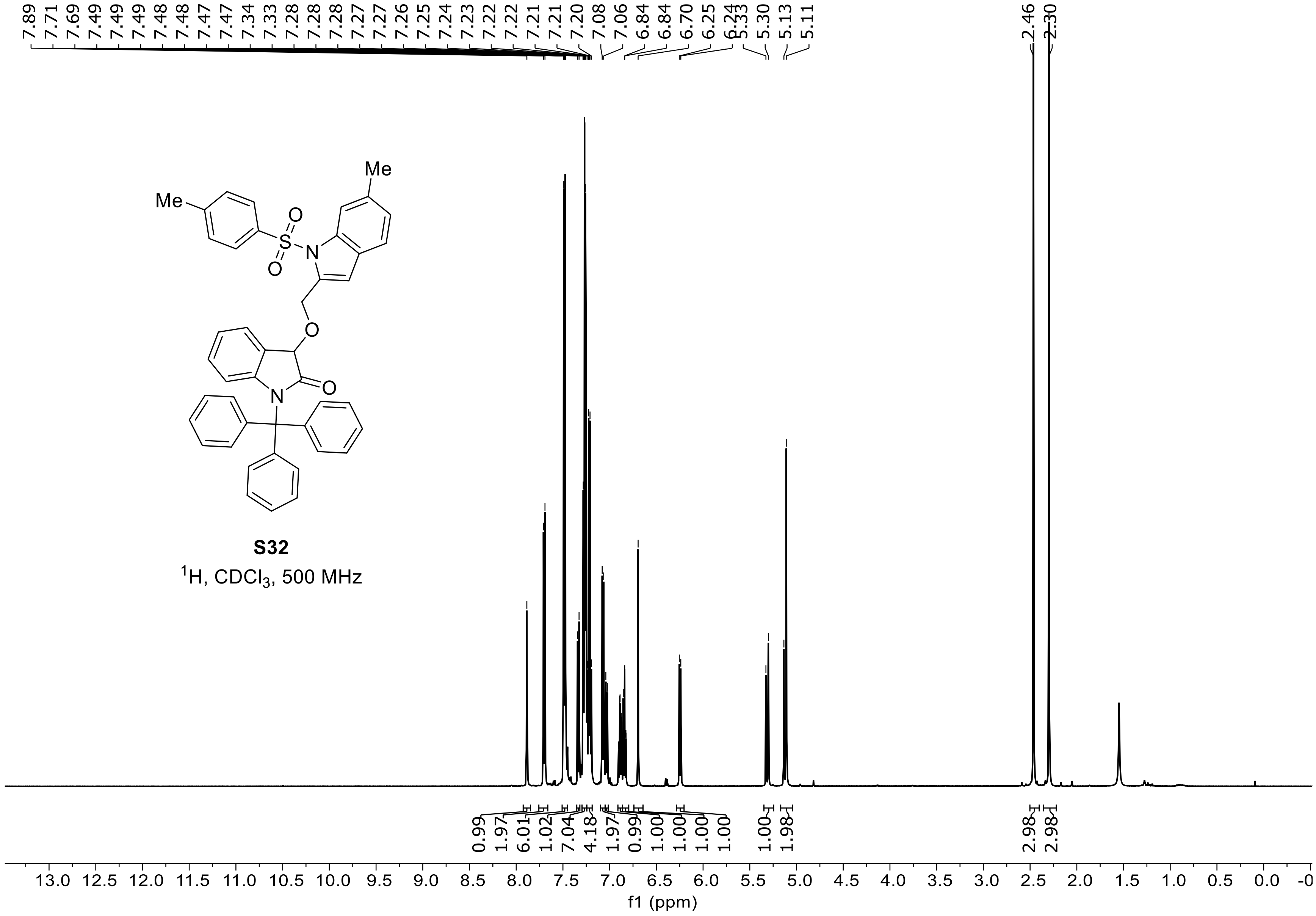

7.89 7.71 7.69 7.49 7.49 7.49 7.48 7.48 7.47 7.47 7.34 7.33 7.28 7.28 7.28 7.27 7.27 7.26 7.25 7.24 7.23 7.22 7.22 7.21 7.21 7.20 7.08 7.06 6.84 6.84 6.70 6.25 6.24 5.33 5.30 5.13 5.11
2.46 2.30
Me
Me
O
S
O
N
O
O
N
S32
^{1}H, $CDCl_3$, 500 MHz
0.99 1.97 6.01 1.02 7.04 4.18 1.97 0.99 1.00 1.00 1.00 1.00 1.00 1.98 2.98 2.98
13.0 12.5 12.0 11.5 11.0 10.5 10.0 9.5 9.0 8.5 8.0 7.5 7.0 6.5 6.0 5.5 5.0 4.5 4.0 3.5 3.0 2.5 2.0 1.5 1.0 0.5 0.0 -0
f1 (ppm)

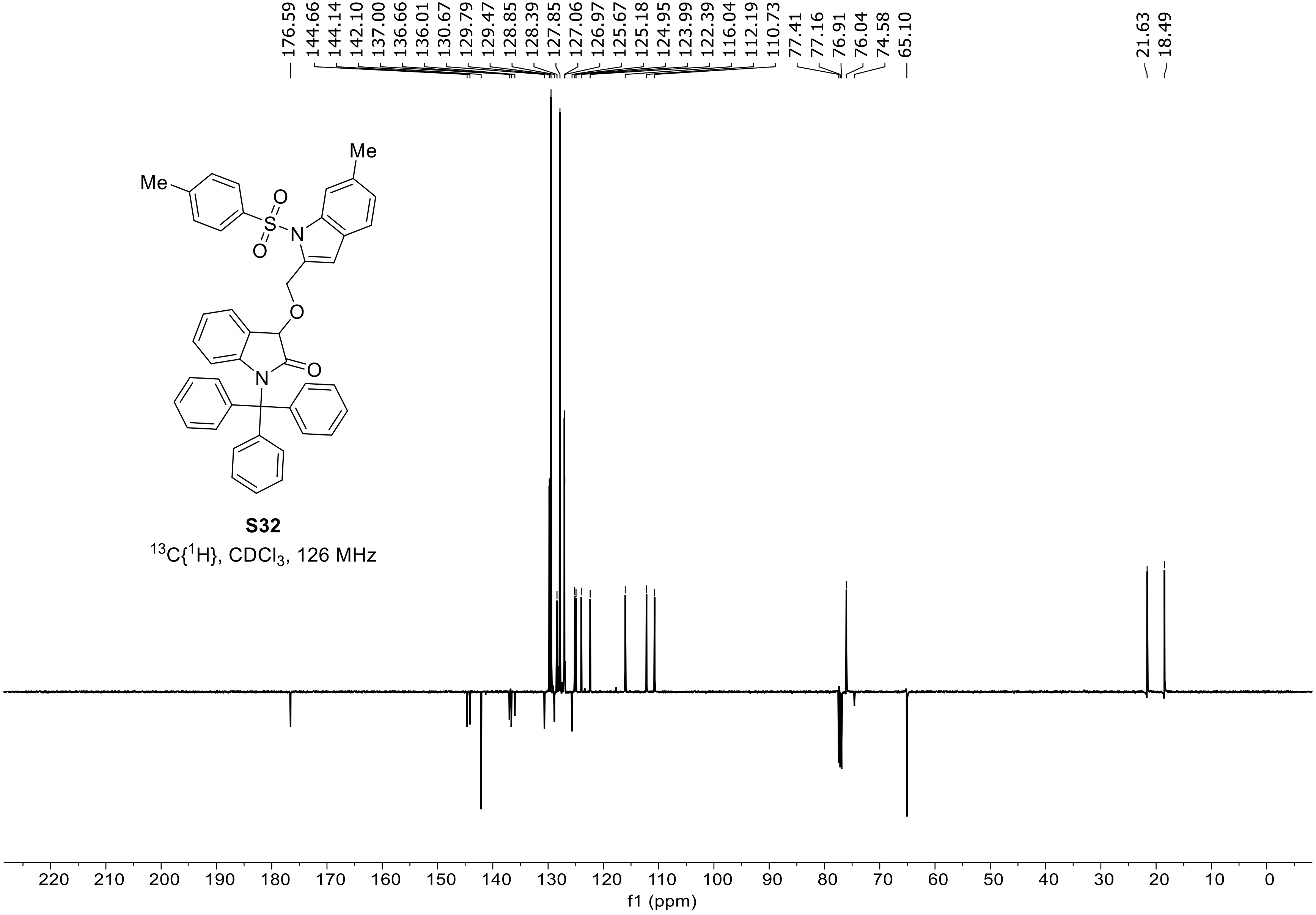
176.59
144.66
144.14
142.10
137.00
136.66
136.01
130.67
129.79
129.47
128.85
128.39
127.85
127.06
126.97
125.67
125.18
124.95
123.99
122.39
116.04
112.19
110.73
77.41
77.16
76.91
76.04
74.58
65.10
21.63
18.49
Me
Me
O
S
N
O
O
O
N
S32
$^{13}C\{^{1}H\}$, $CDCl_3$, 126 MHz
220 210 200 190 180 170 160 150 140 130 120 110 100 90 80 70 60 50 40 30 20 10 0
f1 (ppm)

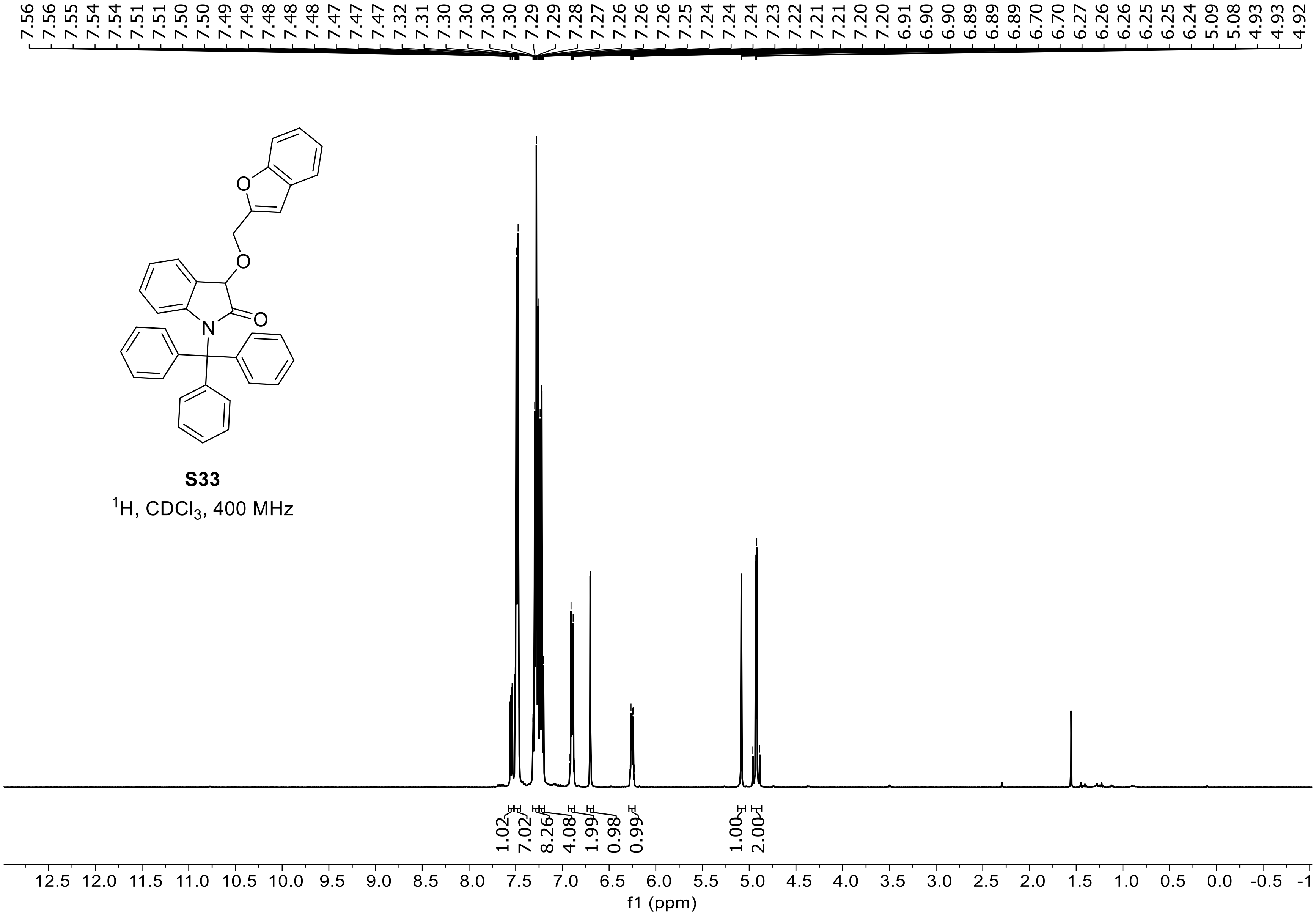
7.56 7.56 7.55 7.54 7.54 7.51 7.51 7.50 7.50 7.49 7.49 7.48 7.48 7.48 7.47 7.47 7.47 7.32 7.31 7.30 7.30 7.30 7.29 7.29 7.28 7.27 7.26 7.26 7.25 7.24 7.24 7.24 7.23 7.22 7.21 7.21 7.20 7.20 6.91 6.90 6.90 6.89 6.89 6.89 6.70 6.70 6.27 6.26 6.26 6.25 6.25 6.24 5.09 5.08 4.93 4.93 4.92
S33
^{1}H, CDCl$_3$, 400 MHz
1.02 7.02 8.26 4.08 1.99 0.98 0.99 1.00 2.00
12.5 12.0 11.5 11.0 10.5 10.0 9.5 9.0 8.5 8.0 7.5 7.0 6.5 6.0 5.5 5.0 4.5 4.0 3.5 3.0 2.5 2.0 1.5 1.0 0.5 0.0 -0.5 -1
f1 (ppm)

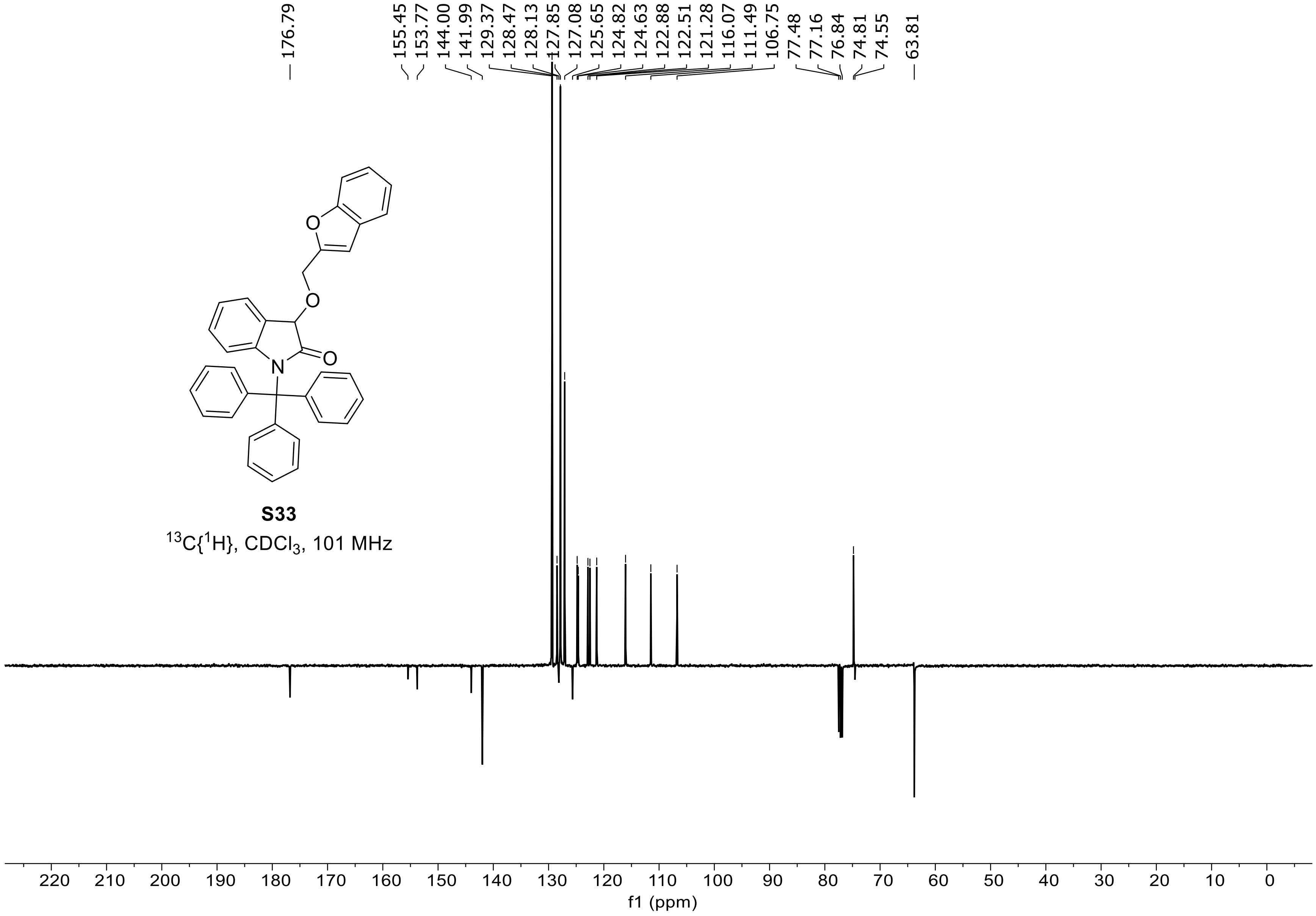
176.79
155.45
153.77
144.00
141.99
129.37
128.47
128.13
127.85
127.08
125.65
124.82
124.63
122.88
122.51
121.28
116.07
111.49
106.75
77.48
77.16
76.84
74.81
74.55
63.81
O
O
N
O
S33
$^{13}C\{^{1}H\}$, $CDCl_3$, 101 MHz
220 210 200 190 180 170 160 150 140 130 120 110 100 90 80 70 60 50 40 30 20 10 0
f1 (ppm)

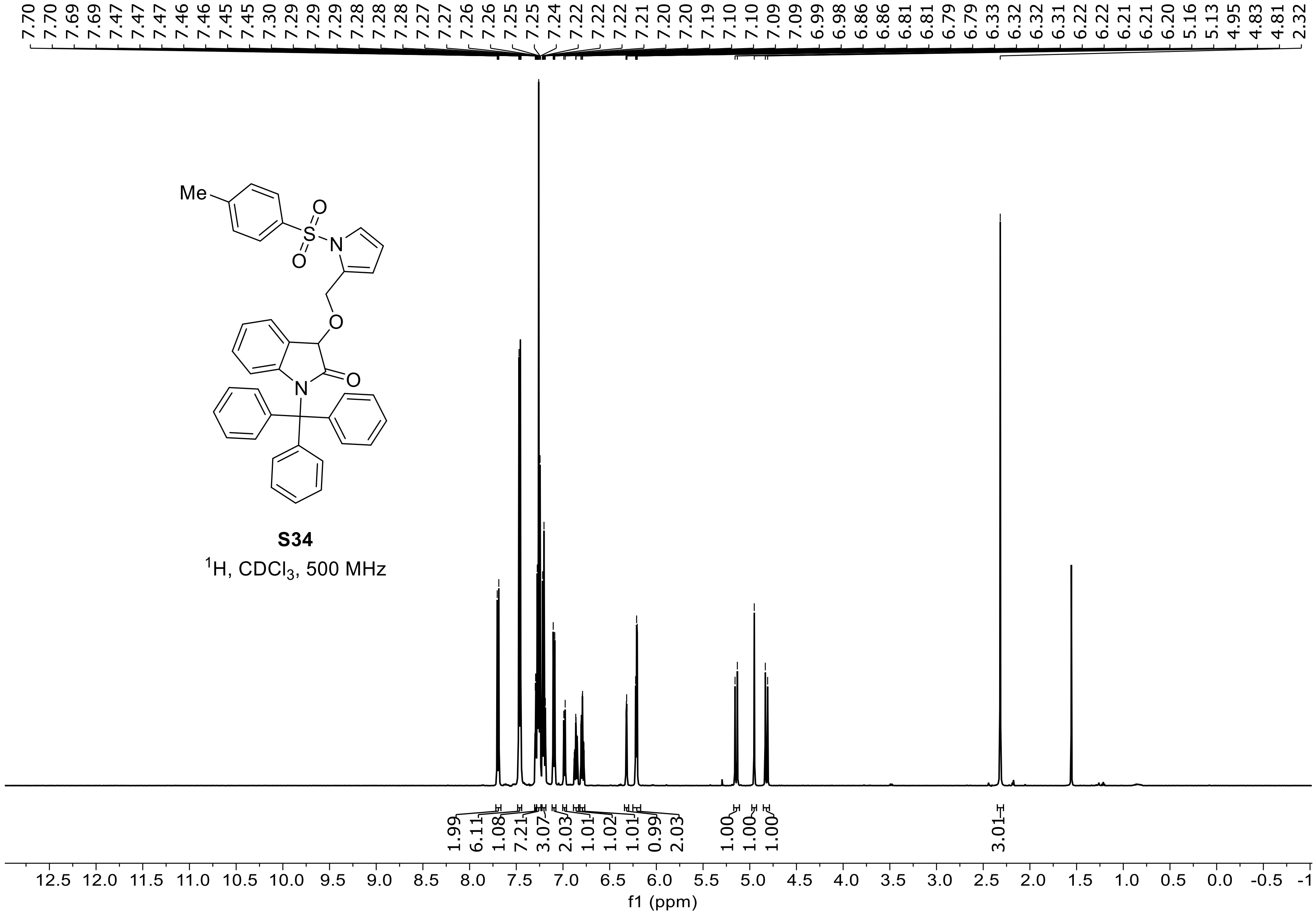
7.70 7.70 7.69 7.69 7.47 7.47 7.47 7.46 7.46 7.45 7.45 7.30 7.29 7.29 7.29 7.28 7.28 7.28 7.27 7.27 7.26 7.26 7.25 7.25 7.24 7.22 7.22 7.22 7.21 7.20 7.20 7.19 7.10 7.10 7.09 7.09 6.99 6.98 6.86 6.86 6.81 6.81 6.79 6.79 6.33 6.32 6.32 6.31 6.22 6.22 6.21 6.21 6.20 5.16 5.13 4.95 4.83 4.81 2.32
Me
O
S
N
O
O
N
O
S34
^1H, $CDCl_3$, 500 MHz
1.99 6.11 1.08 7.21 3.07 2.03 1.01 1.02 1.01 0.99 2.03 1.00 1.00 1.00 3.01
12.5 12.0 11.5 11.0 10.5 10.0 9.5 9.0 8.5 8.0 7.5 7.0 6.5 6.0 5.5 5.0 4.5 4.0 3.5 3.0 2.5 2.0 1.5 1.0 0.5 0.0 -0.5 -1
f1 (ppm)

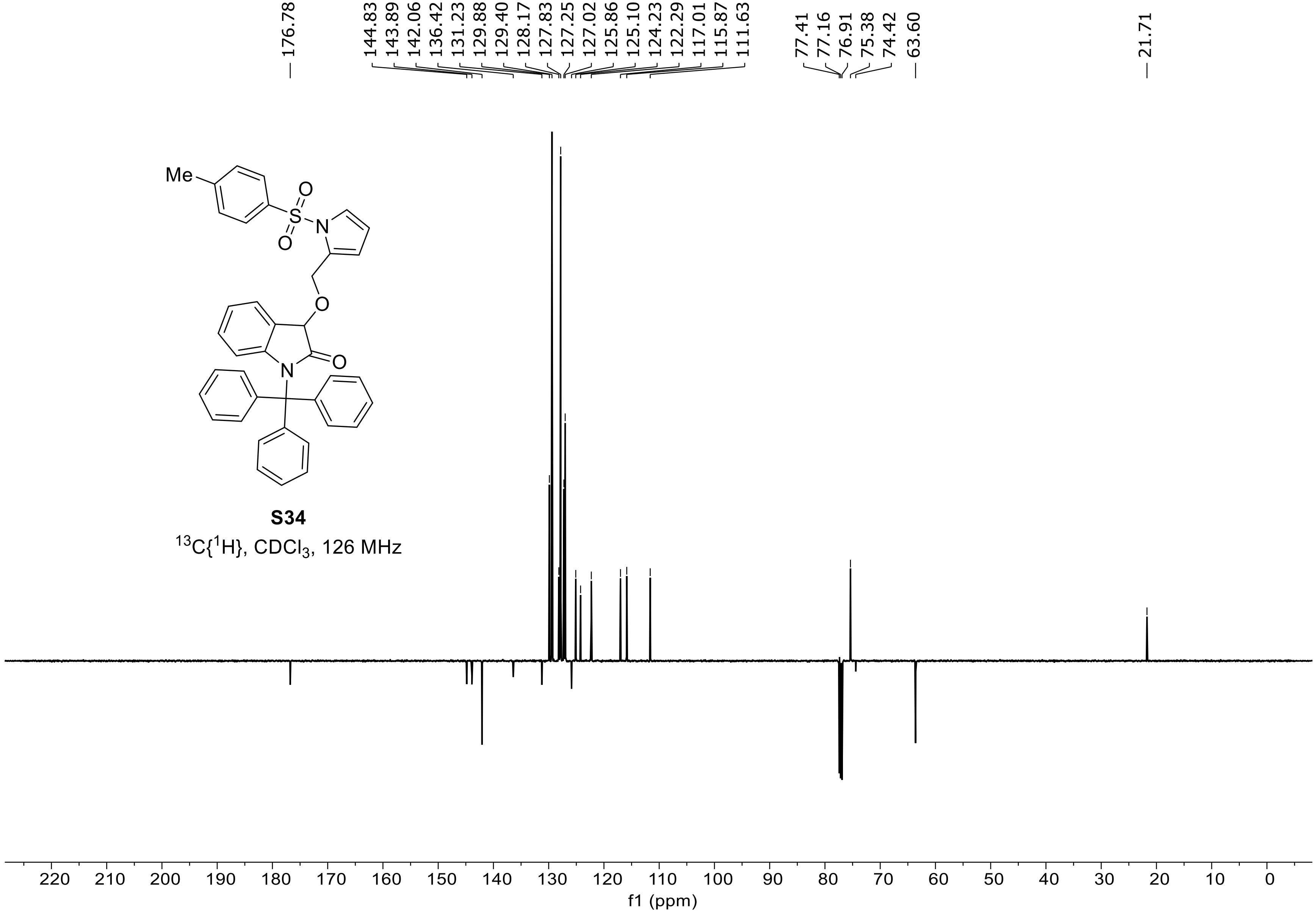
176.78
144.83
143.89
142.06
136.42
131.23
129.88
129.40
128.17
127.83
127.25
127.02
125.86
125.10
124.23
122.29
117.01
115.87
111.63
77.41
77.16
76.91
75.38
74.42
63.60
21.71
Me
S
O
O
N
O
N
O
S34
$^{13}C\{^{1}H\}$, $CDCl_3$, 126 MHz
220 210 200 190 180 170 160 150 140 130 120 110 100 90 80 70 60 50 40 30 20 10 0
f1 (ppm)

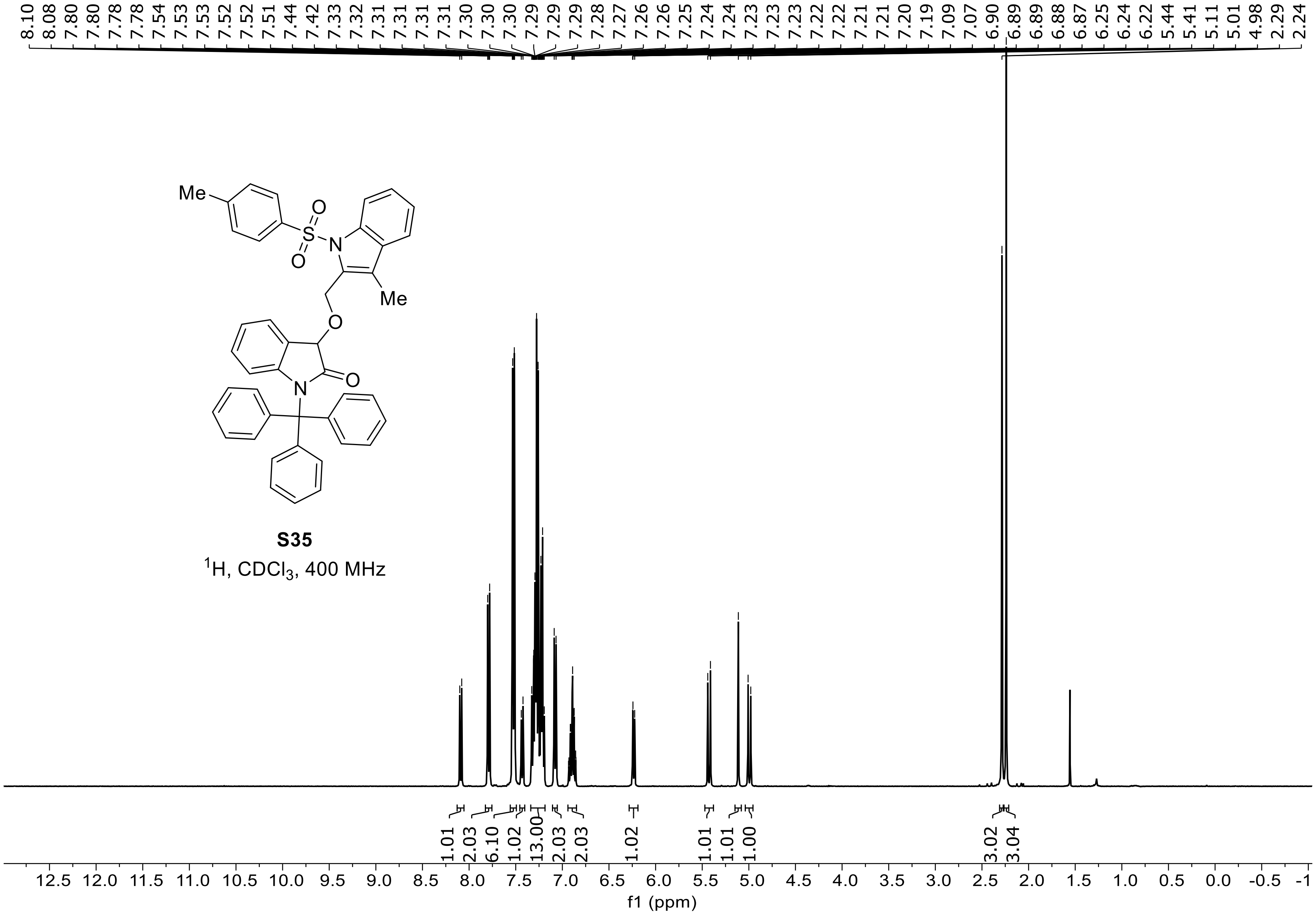
8.10 8.08 7.80 7.80 7.78 7.78 7.54 7.53 7.53 7.52 7.52 7.51 7.44 7.42 7.33 7.32 7.31 7.31 7.31 7.31 7.30 7.30 7.30 7.29 7.29 7.29 7.28 7.27 7.26 7.26 7.25 7.24 7.24 7.23 7.23 7.23 7.22 7.22 7.21 7.21 7.20 7.19 7.09 7.07 6.90 6.89 6.89 6.88 6.87 6.25 6.24 6.22 5.44 5.41 5.11 5.01 4.98 2.29 2.24
Me
O
S
O
N
Me
O
O
N
O
S35
^{1}H, $CDCl_3$, 400 MHz
1.01 2.03 6.10 1.02 13.00 2.03 2.03 1.02 1.01 1.01 1.00 3.02 3.04
12.5 12.0 11.5 11.0 10.5 10.0 9.5 9.0 8.5 8.0 7.5 7.0 6.5 6.0 5.5 5.0 4.5 4.0 3.5 3.0 2.5 2.0 1.5 1.0 0.5 0.0 -0.5 -1
f1 (ppm)

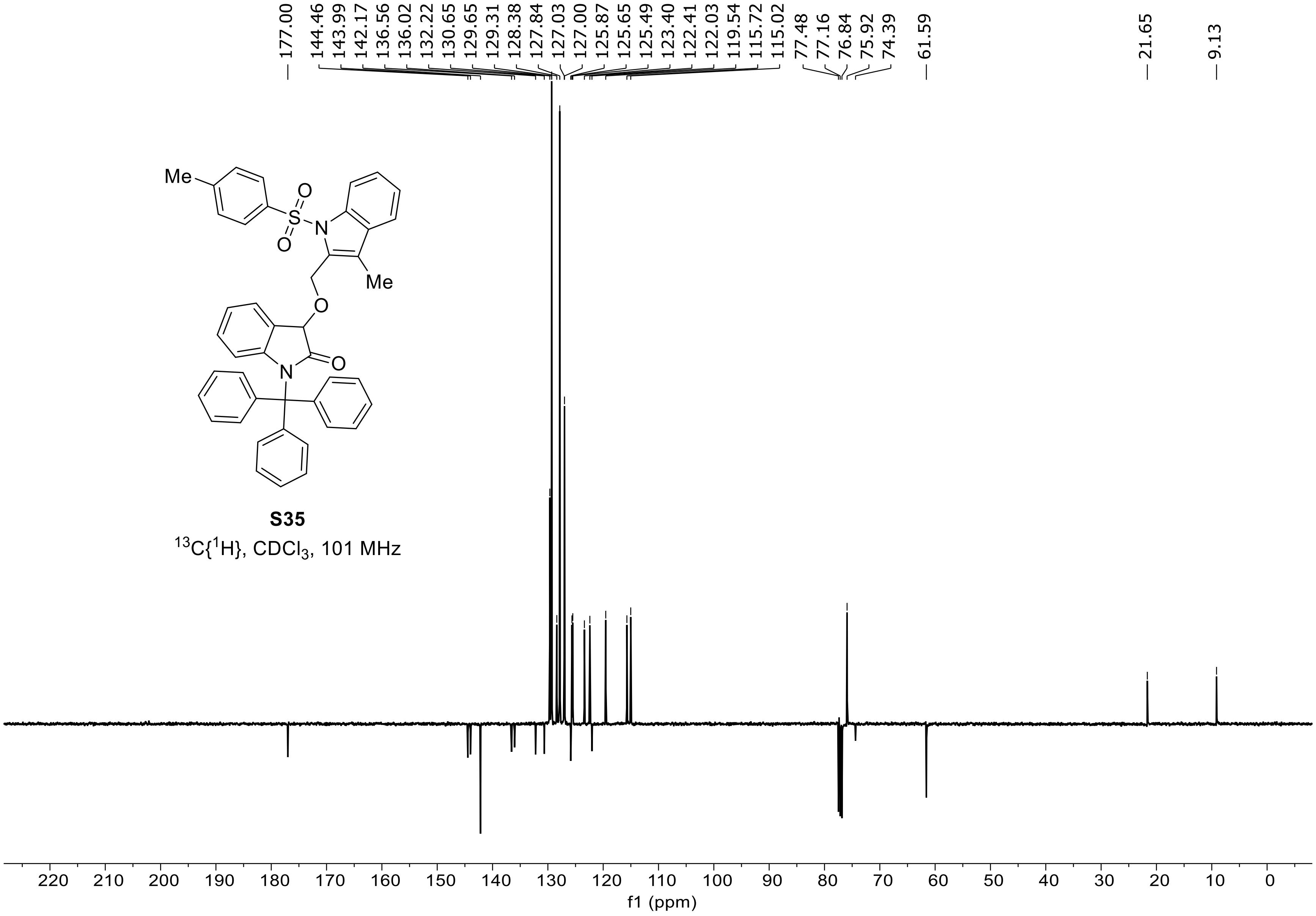
177.00
144.46
143.99
142.17
136.56
136.02
132.22
130.65
129.65
129.31
128.38
127.84
127.03
127.00
125.87
125.65
125.49
123.40
122.41
122.03
119.54
115.72
115.02
77.48
77.16
76.84
75.92
74.39
61.59
21.65
9.13
Me
O
S
O
N
Me
O
O
N
S35
$^{13}C\{^{1}H\}$, $CDCl_3$, 101 MHz
220 210 200 190 180 170 160 150 140 130 120 110 100 90 80 70 60 50 40 30 20 10 0
f1 (ppm)

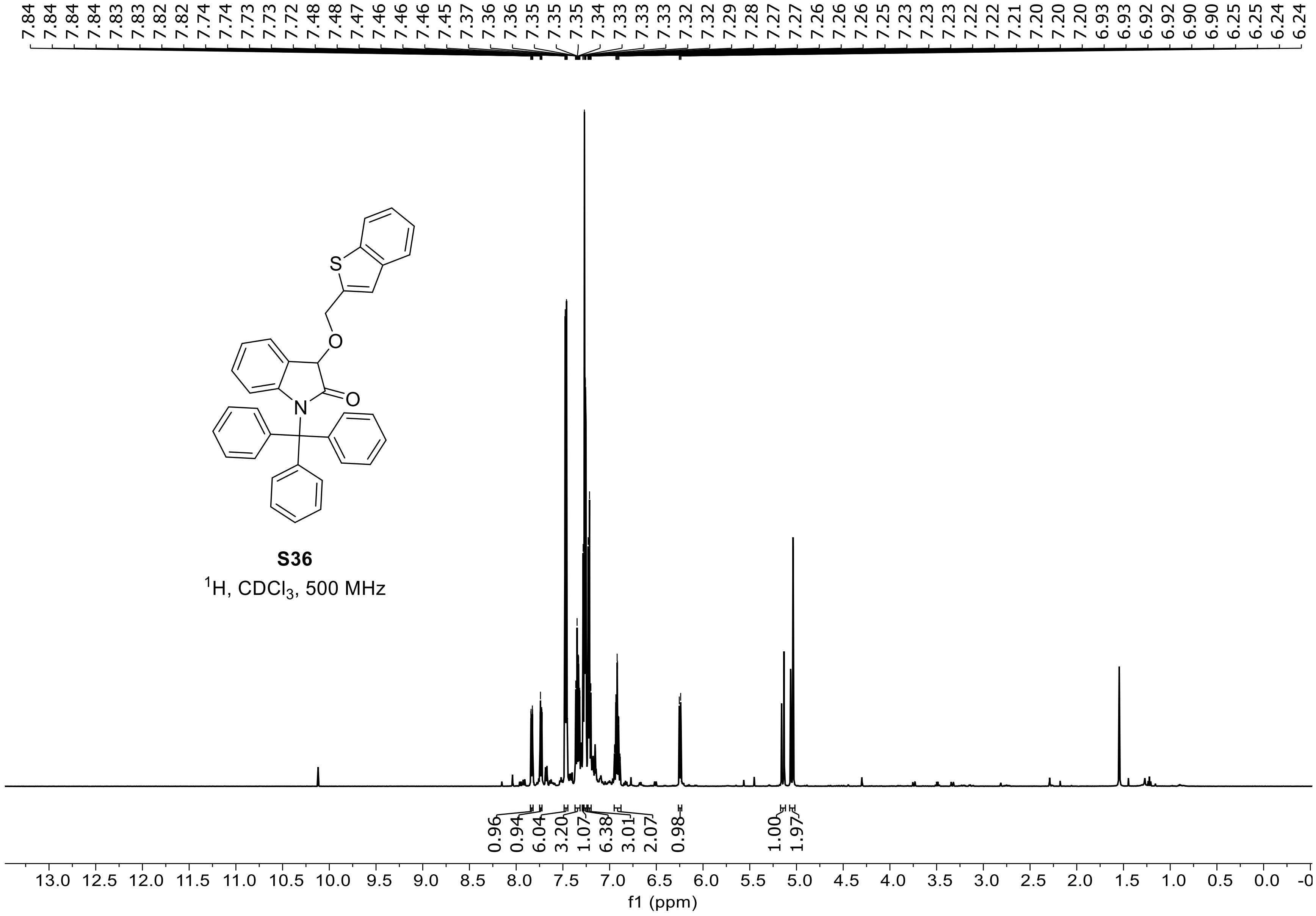
7.84
7.84
7.84
7.84
7.83
7.83
7.82
7.82
7.74
7.74
7.73
7.73
7.72
7.48
7.48
7.47
7.46
7.46
7.46
7.45
7.37
7.36
7.36
7.35
7.35
7.35
7.34
7.33
7.33
7.33
7.32
7.32
7.29
7.28
7.27
7.27
7.26
7.26
7.26
7.25
7.23
7.23
7.23
7.22
7.22
7.21
7.20
7.20
7.20
6.93
6.93
6.92
6.92
6.90
6.90
6.25
6.25
6.24
6.24
S
O
O
N
S36
^{1}H, CDCl$_3$, 500 MHz
0.96
0.94
6.04
3.20
1.07
6.38
3.01
2.07
0.98
1.00
1.97
13.0 12.5 12.0 11.5 11.0 10.5 10.0 9.5 9.0 8.5 8.0 7.5 7.0 6.5 6.0 5.5 5.0 4.5 4.0 3.5 3.0 2.5 2.0 1.5 1.0 0.5 0.0 -0
f1 (ppm)

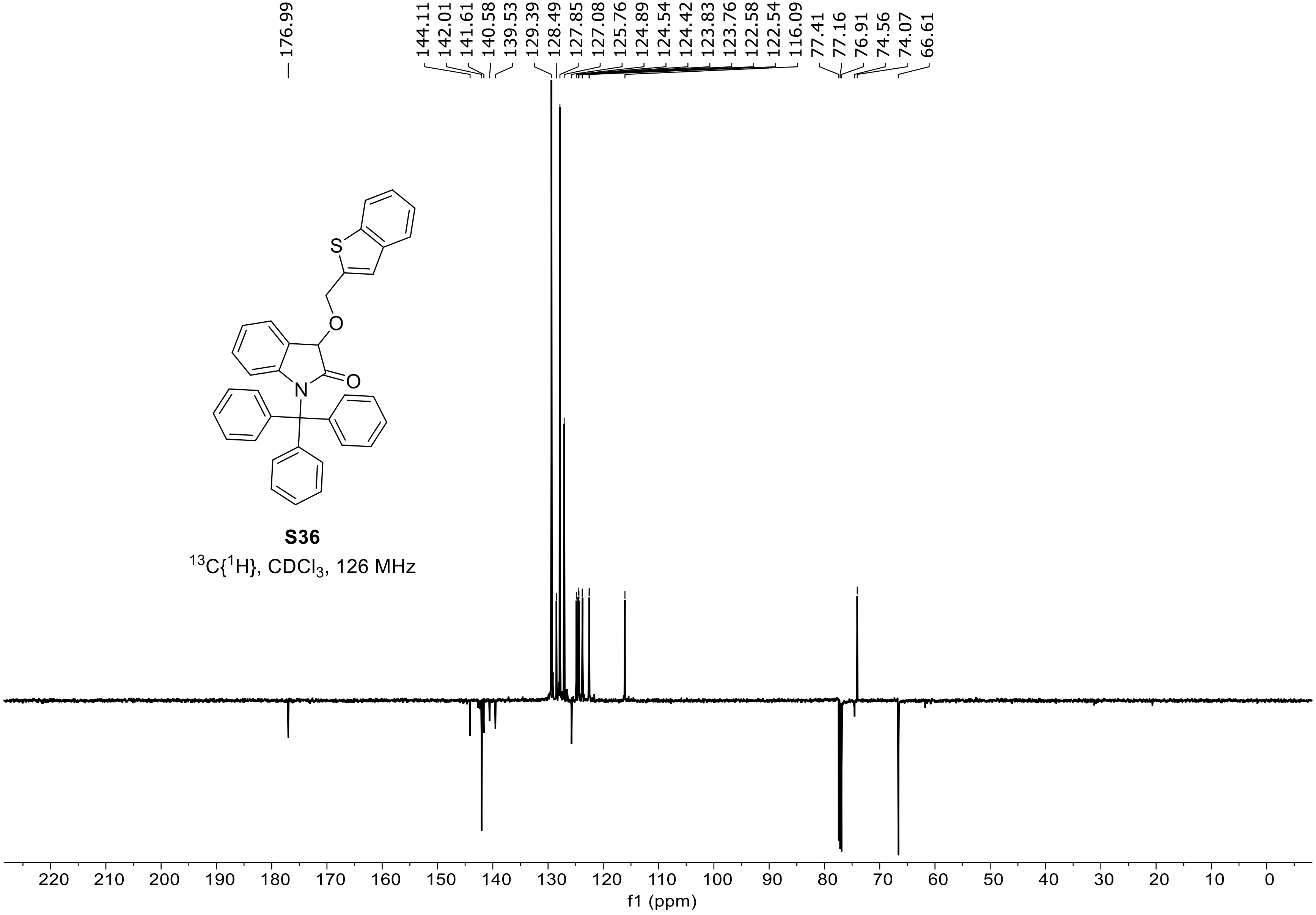

S36

$^{13}C\{^{1}H\}$, $CDCl_3$, 126 MHz

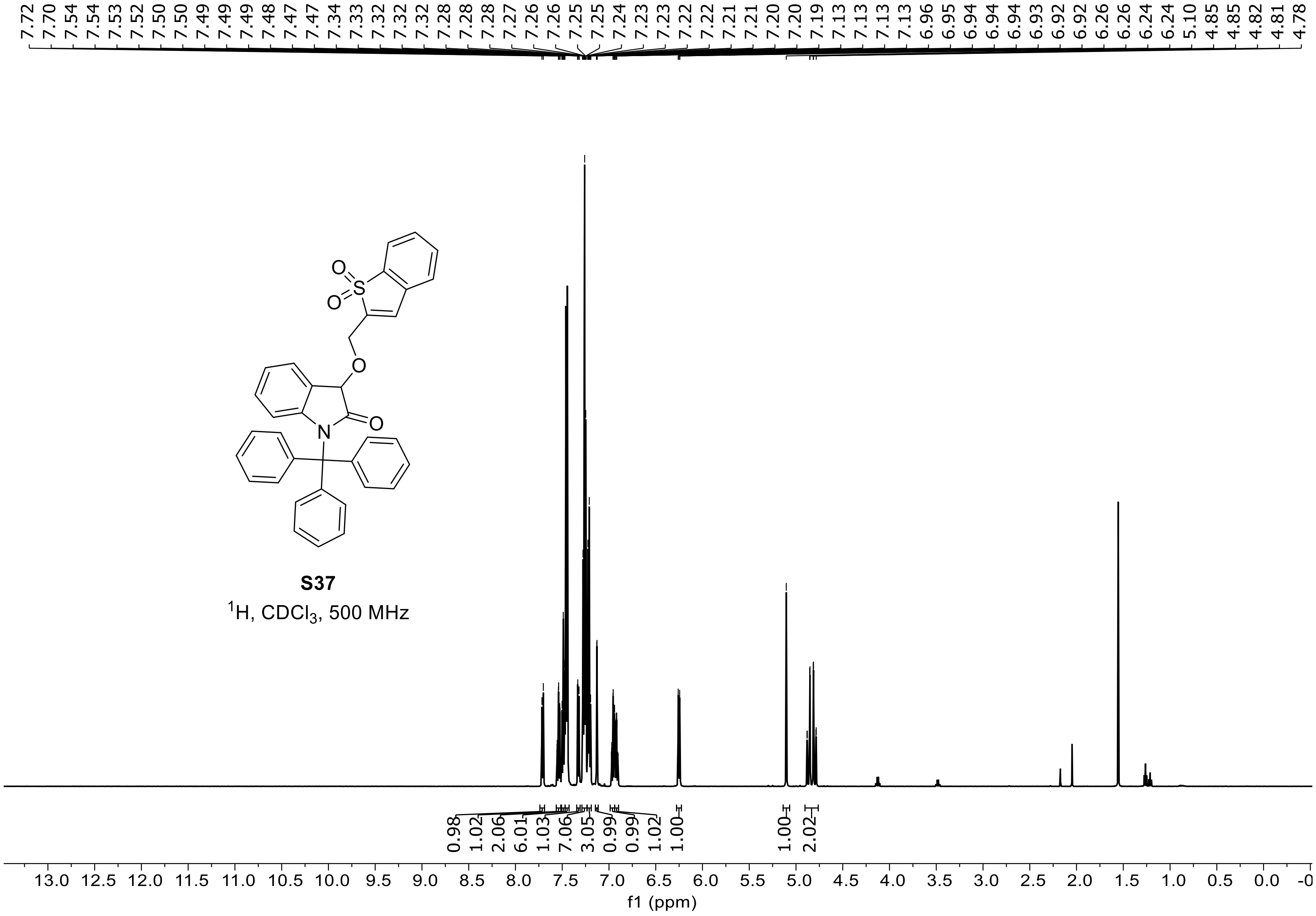
7.72 7.70 7.54 7.54 7.53 7.52 7.50 7.50 7.49 7.49 7.49 7.48 7.47 7.47 7.34 7.33 7.32 7.32 7.32 7.28 7.28 7.28 7.27 7.26 7.26 7.25 7.25 7.24 7.23 7.23 7.22 7.22 7.21 7.21 7.20 7.20 7.19 7.13 7.13 7.13 7.13 6.96 6.95 6.94 6.94 6.94 6.93 6.92 6.92 6.26 6.26 6.24 6.24 5.10 4.85 4.85 4.82 4.81 4.78
S37
^{1}H, CDCl$_3$, 500 MHz
0.98 1.02 2.06 6.01 1.03 7.06 3.05 0.99 0.99 1.02 1.00 1.00 2.02
13.0 12.5 12.0 11.5 11.0 10.5 10.0 9.5 9.0 8.5 8.0 7.5 7.0 6.5 6.0 5.5 5.0 4.5 4.0 3.5 3.0 2.5 2.0 1.5 1.0 0.5 0.0 -0
f1 (ppm)

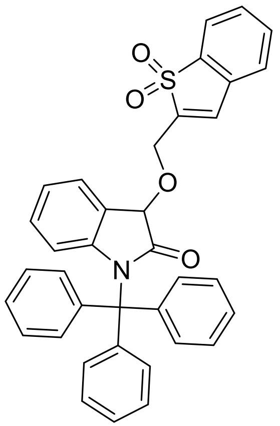

S37

$^{13}C\{^{1}H\}$, $CDCl_3$, 126 MHz

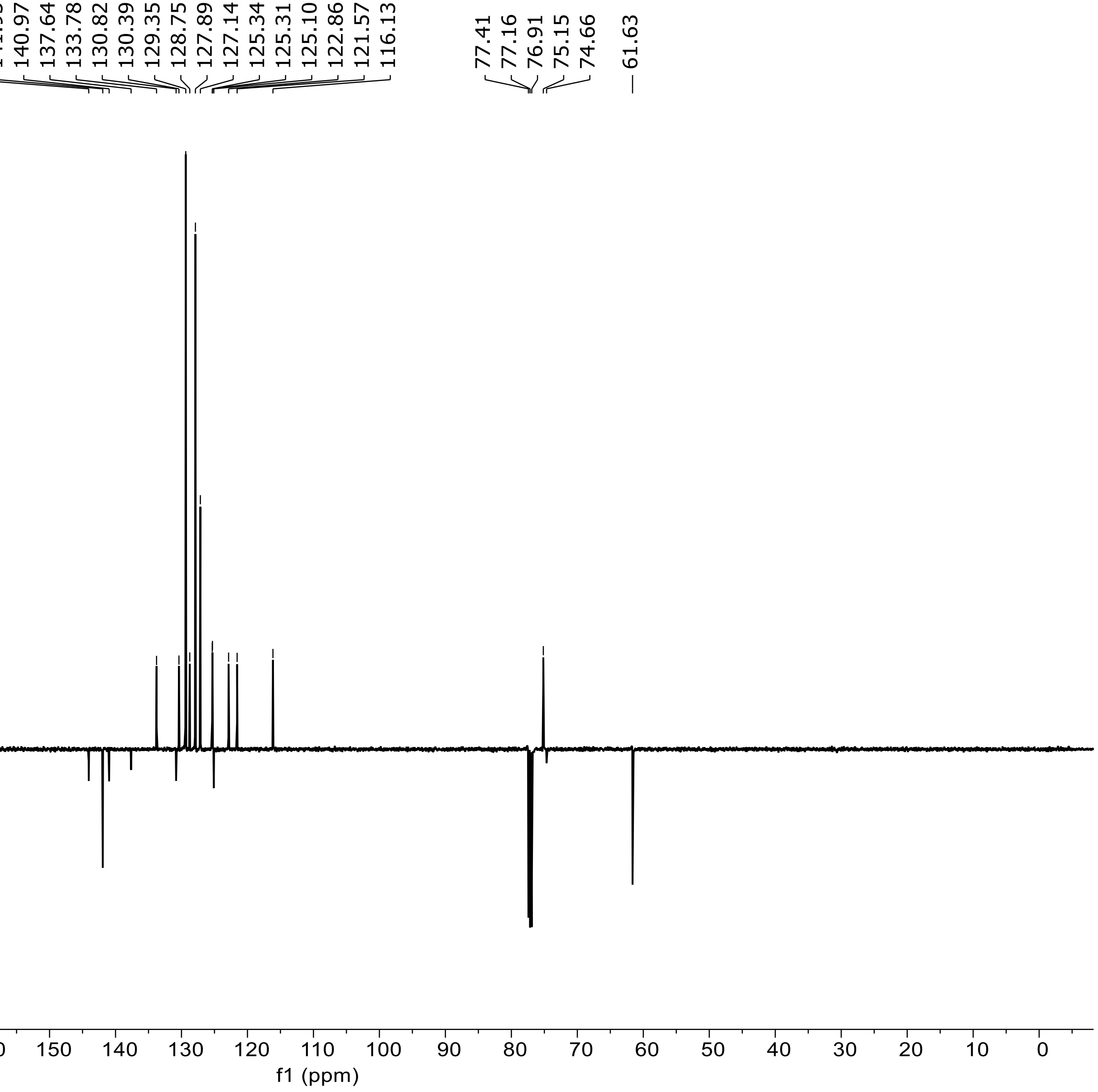

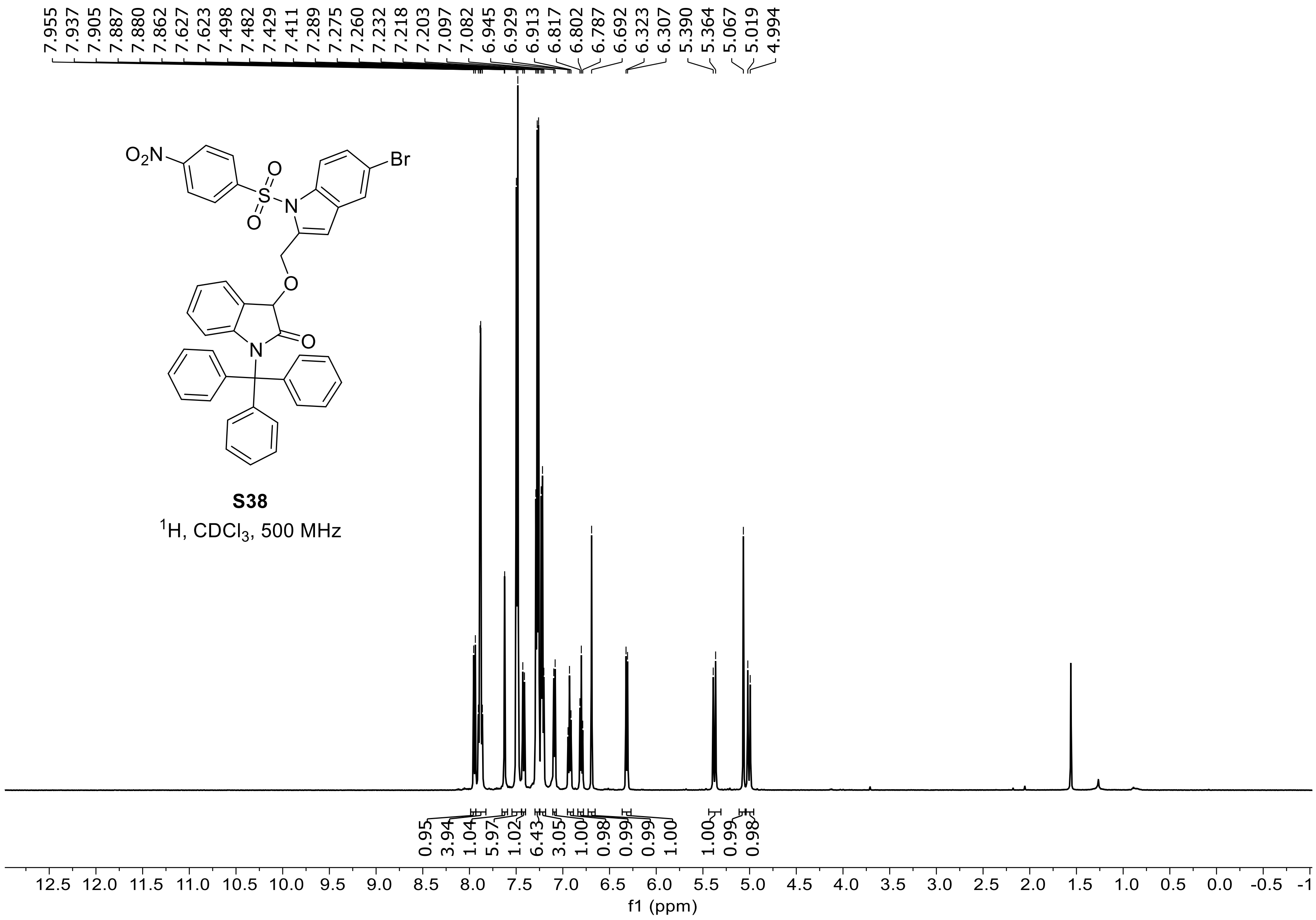

S38
^{1}H, CDCl$_3$, 500 MHz
7.955 7.937 7.905 7.887 7.880 7.862 7.627 7.623 7.498 7.482 7.429 7.411 7.289 7.275 7.260 7.232 7.218 7.203 7.097 7.082 6.945 6.929 6.913 6.817 6.802 6.787 6.692 6.323 6.307 5.390 5.364 5.067 5.019 4.994
0.95 3.94 1.04 5.97 1.02 6.43 3.05 1.00 0.98 0.99 0.99 1.00 1.00 0.99 0.98
12.5 12.0 11.5 11.0 10.5 10.0 9.5 9.0 8.5 8.0 7.5 7.0 6.5 6.0 5.5 5.0 4.5 4.0 3.5 3.0 2.5 2.0 1.5 1.0 0.5 0.0 -0.5 -1
f1 (ppm)

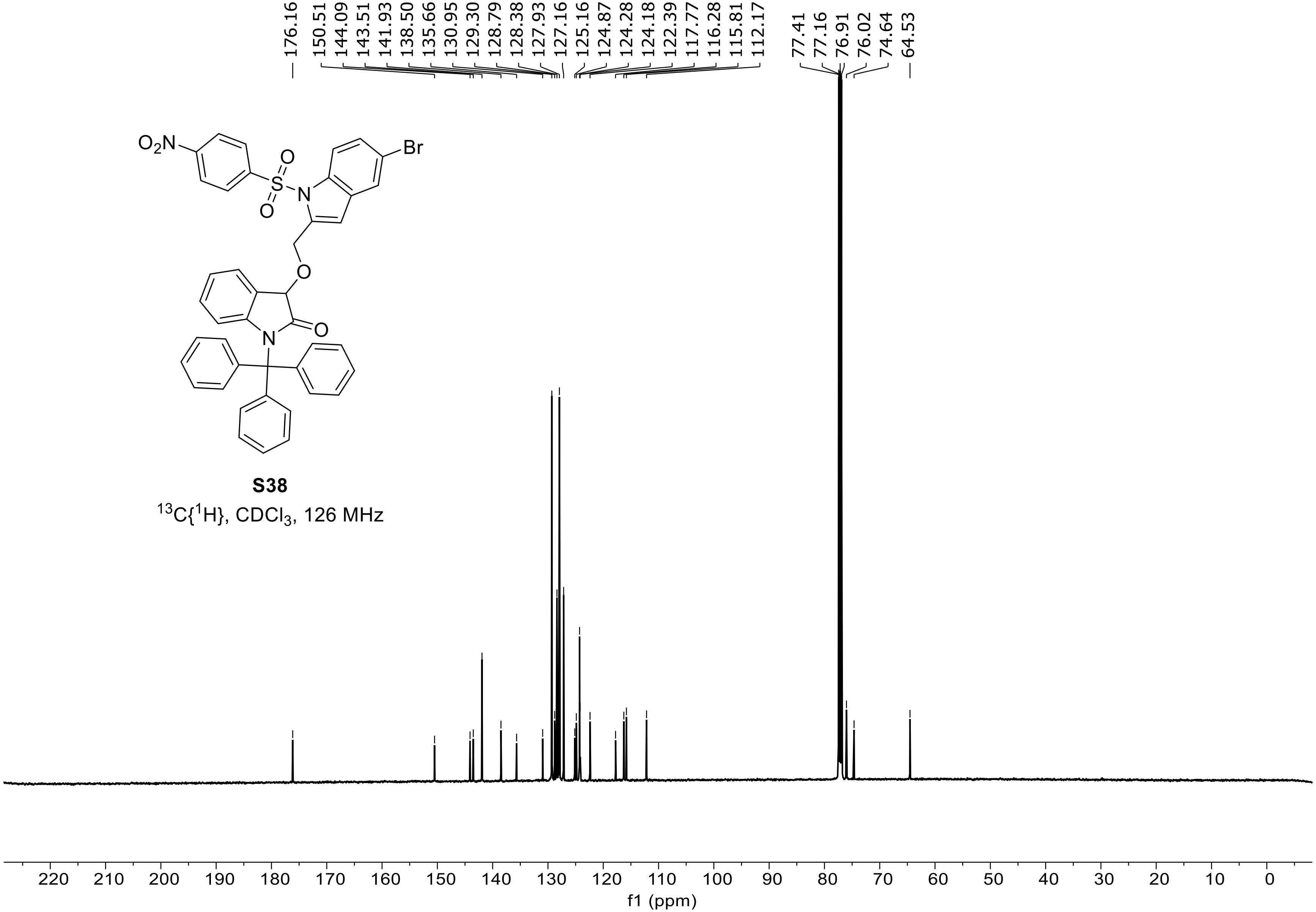

S38

$^{13}C\{^{1}H\}$, $CDCl_3$, 126 MHz

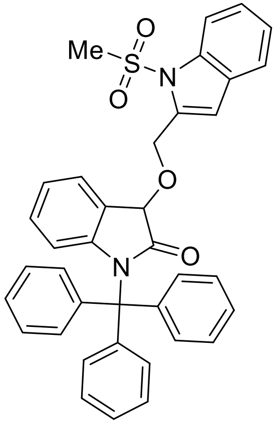

S39

^{1}H, $CDCl_3$, 500 MHz

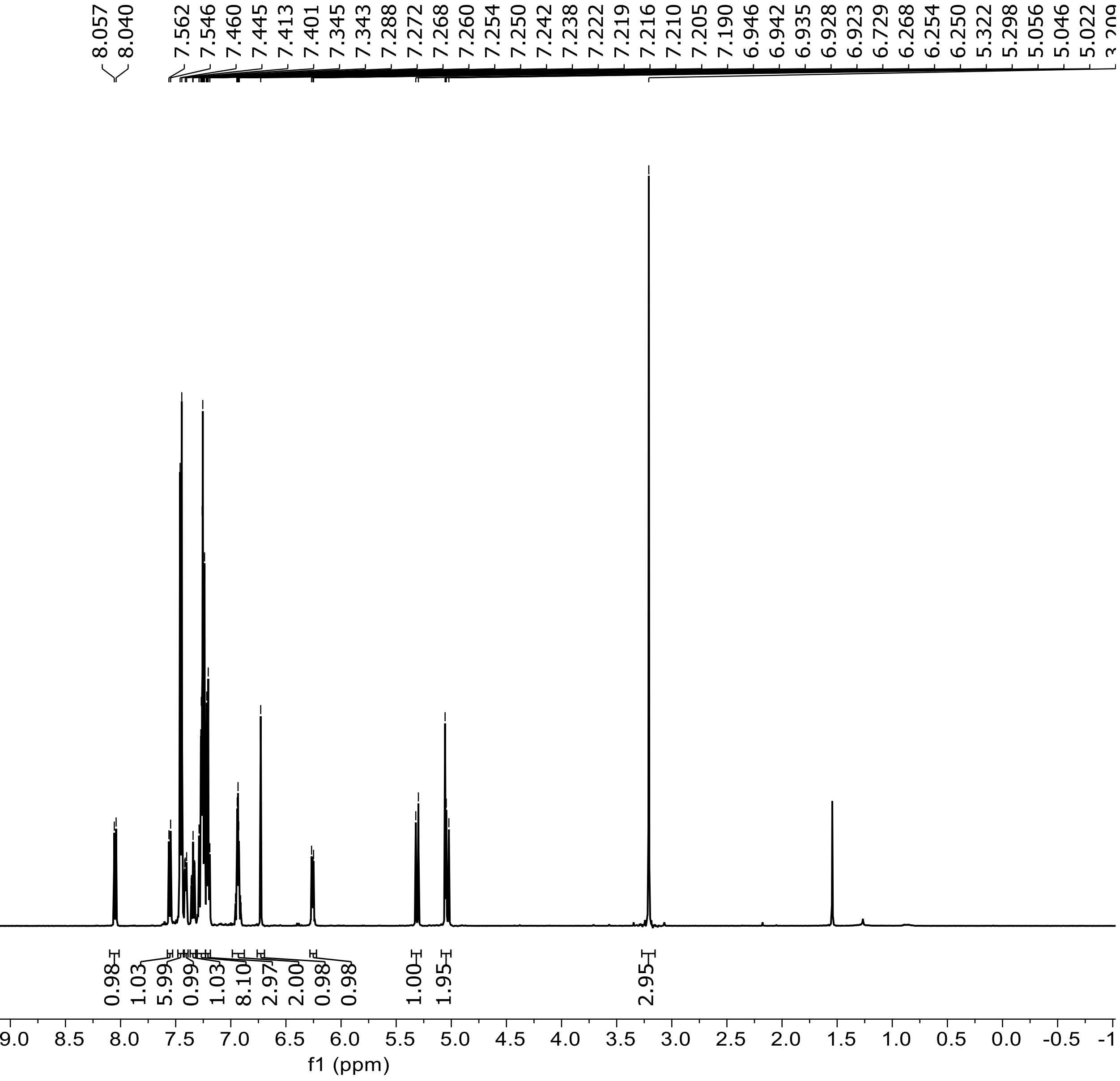

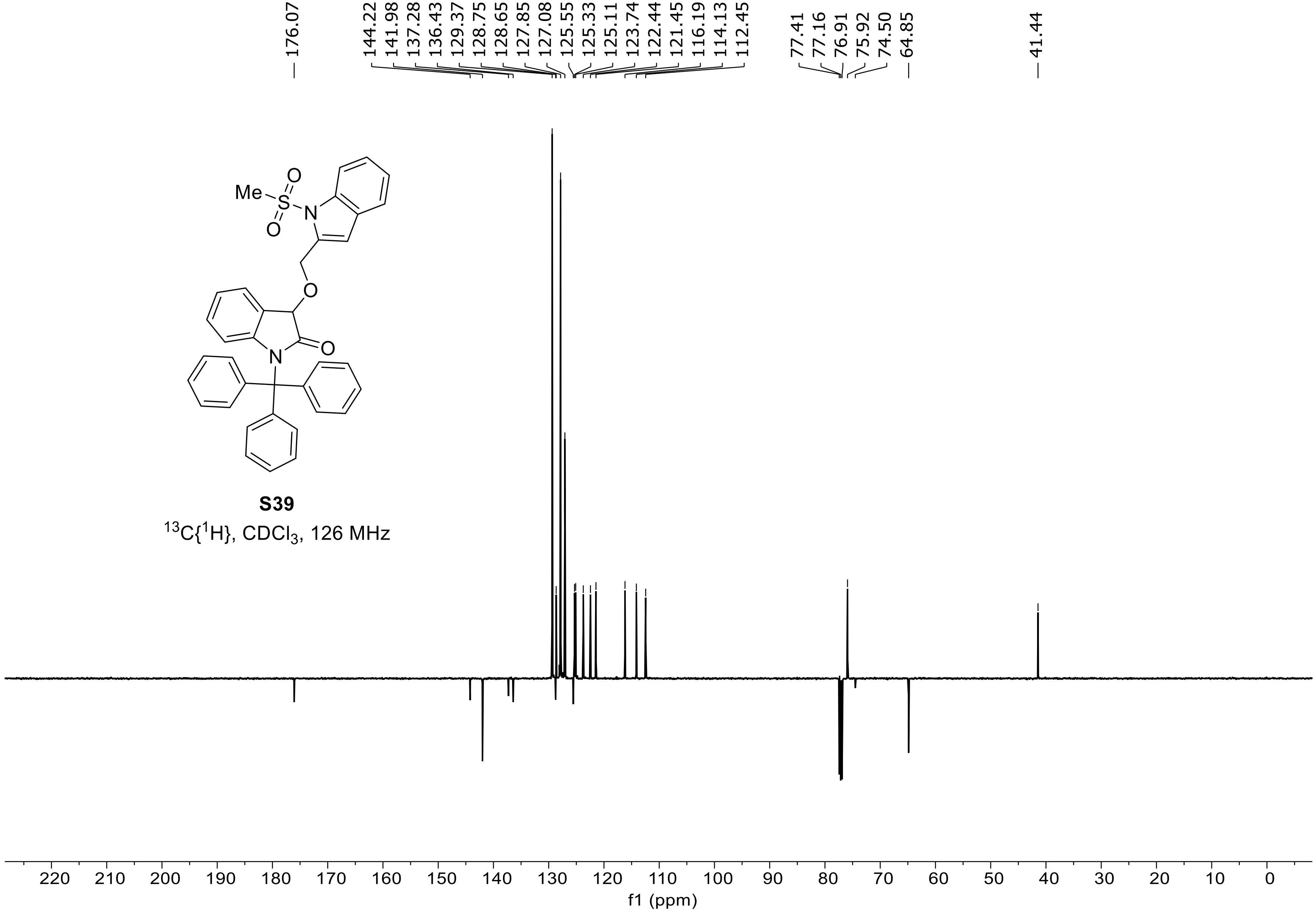

S39

$^{13}C\{^{1}H\}$, $CDCl_3$, 126 MHz

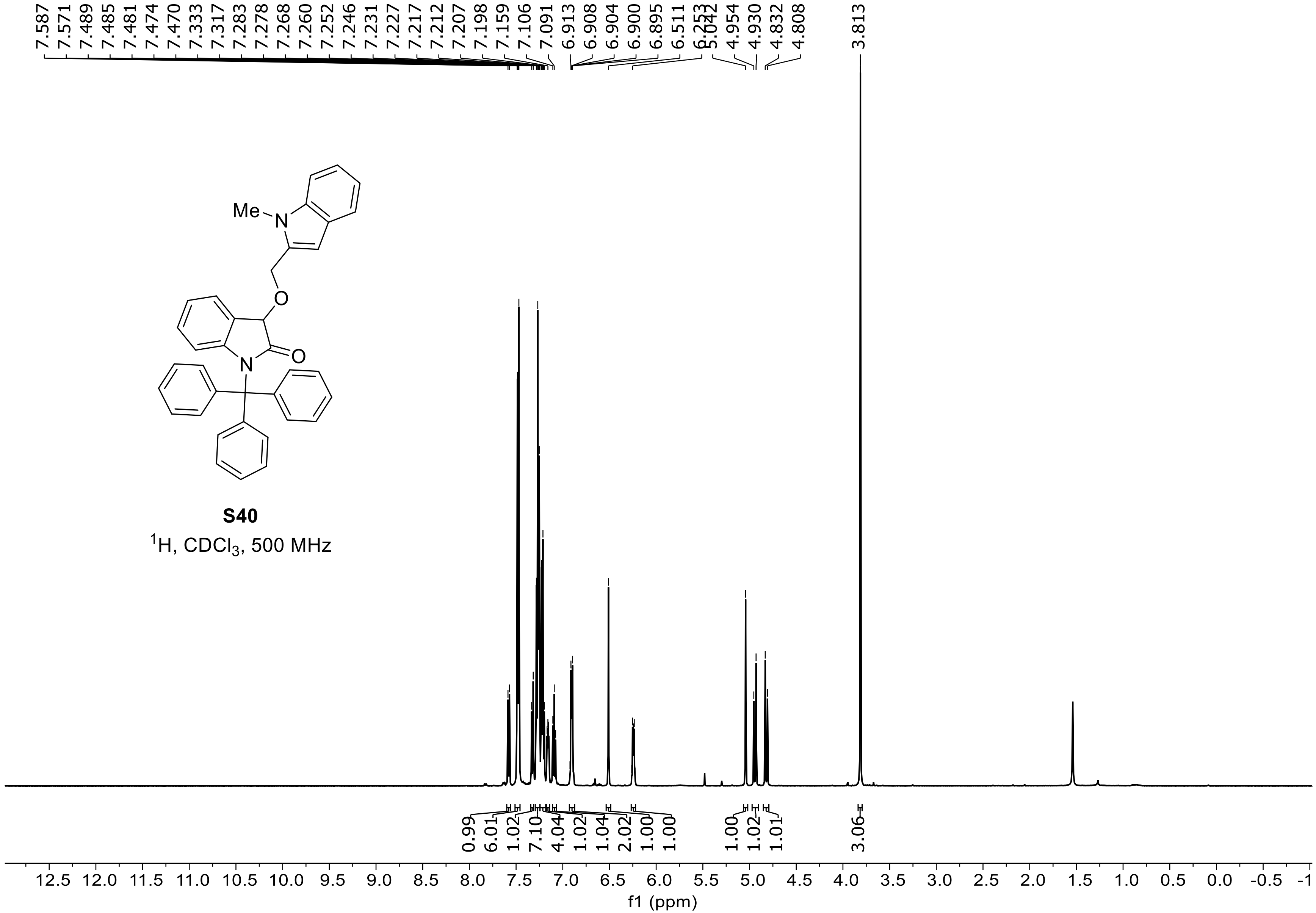
Me
N
O
O
N
S40
1H, CDCl3, 500 MHz
f1 (ppm)

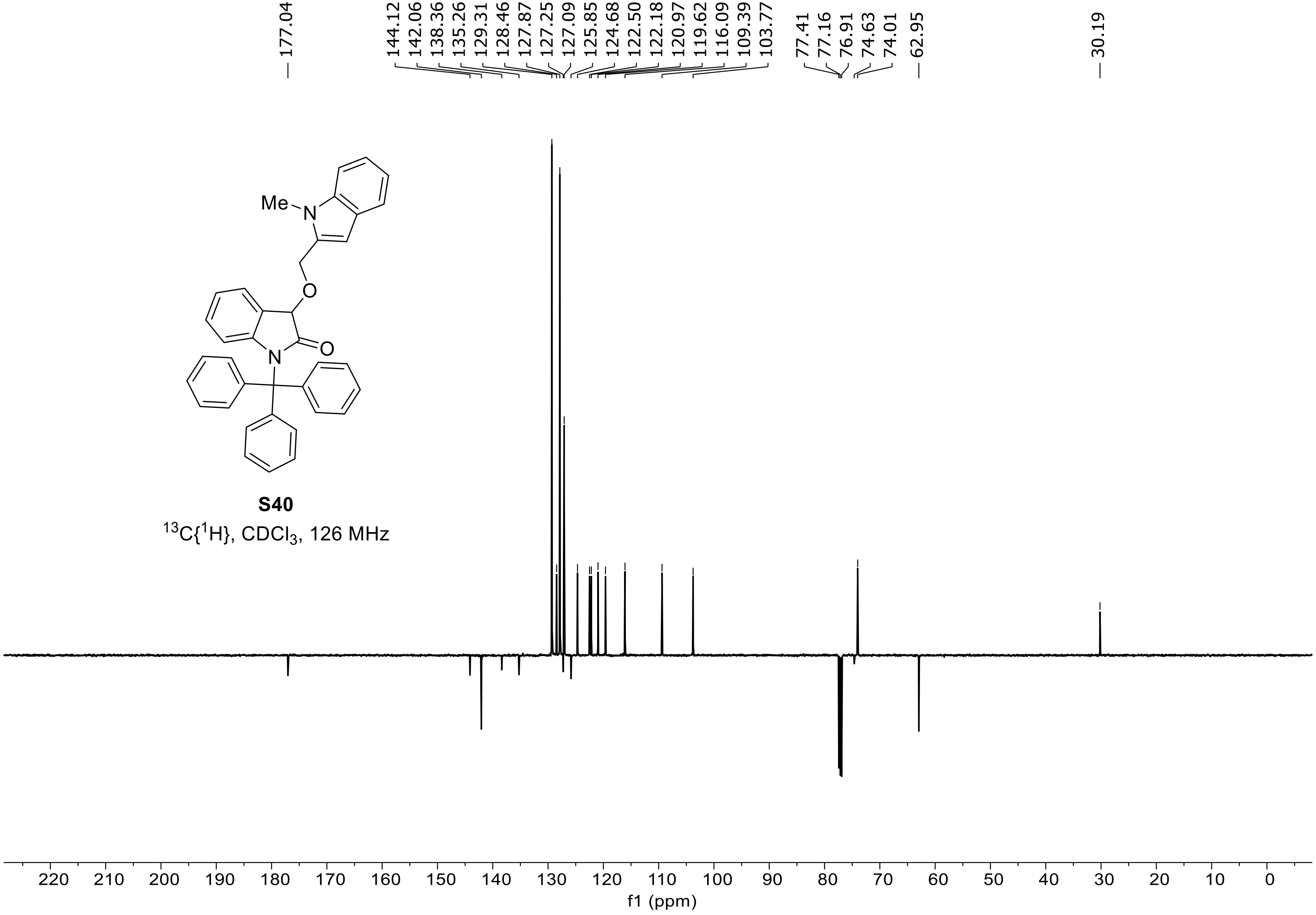

S40

$^{13}C\{^1H\}$, $CDCl_3$, 126 MHz

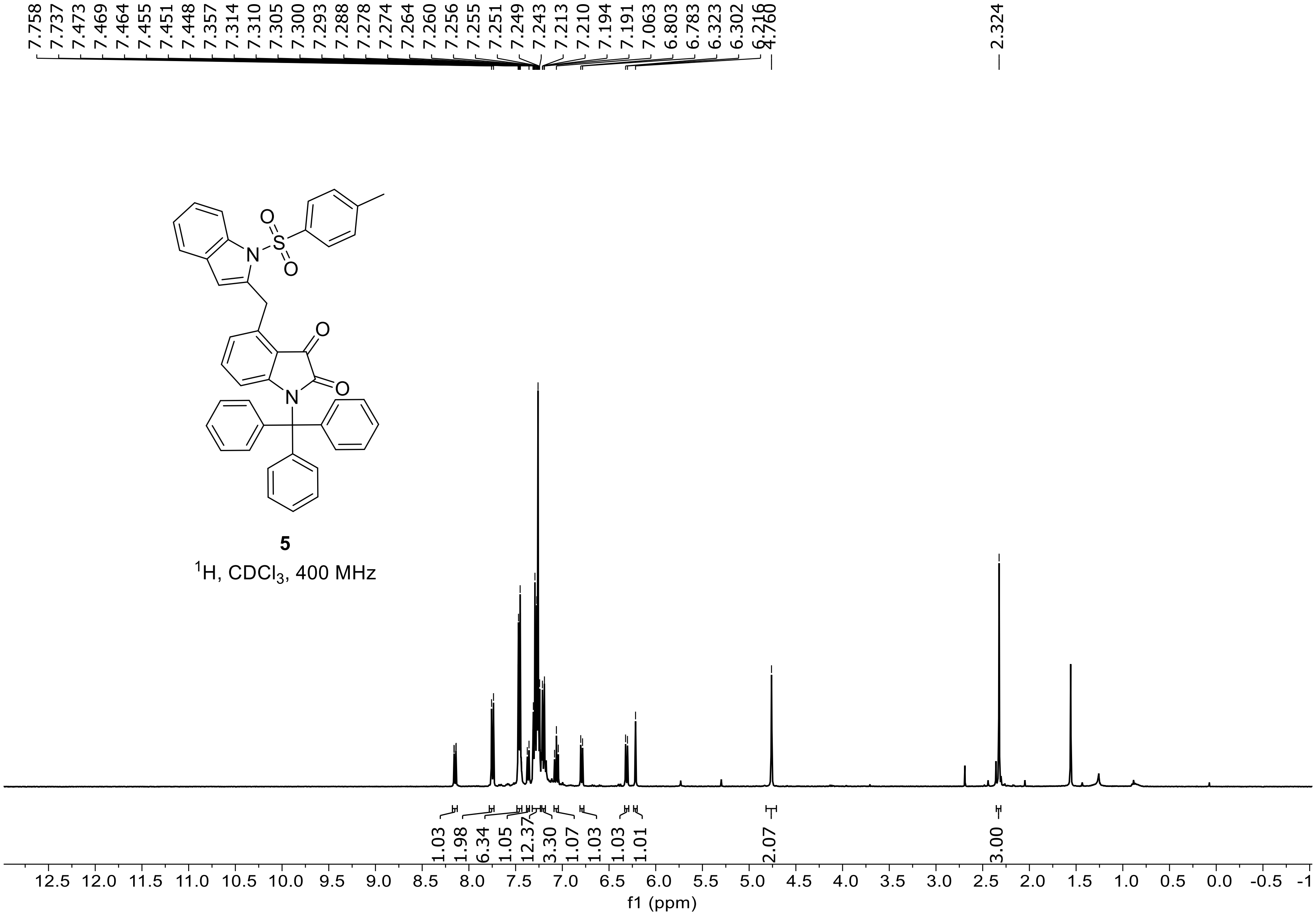

7.758
7.737
7.473
7.469
7.464
7.455
7.451
7.448
7.357
7.314
7.310
7.305
7.300
7.293
7.288
7.278
7.274
7.264
7.260
7.256
7.255
7.251
7.249
7.243
7.213
7.210
7.194
7.191
7.063
6.803
6.783
6.323
6.302
6.216
4.760
2.324
5
^{1}H, CDCl$_3$, 400 MHz
1.03
1.98
6.34
1.05
12.37
3.30
1.07
1.03
1.03
1.01
2.07
3.00
12.5 12.0 11.5 11.0 10.5 10.0 9.5 9.0 8.5 8.0 7.5 7.0 6.5 6.0 5.5 5.0 4.5 4.0 3.5 3.0 2.5 2.0 1.5 1.0 0.5 0.0 -0.5 -1
f1 (ppm)

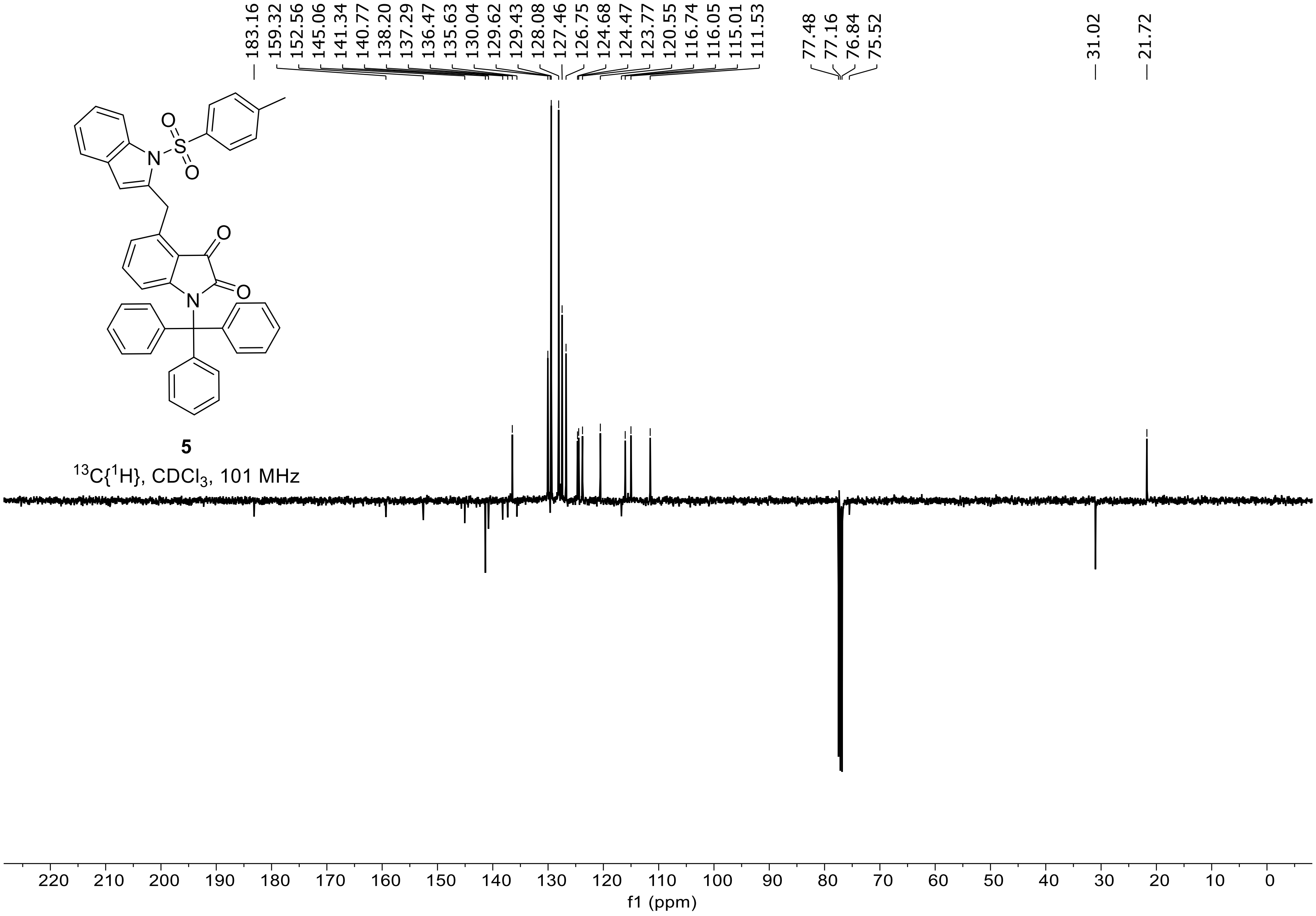
183.16
159.32
152.56
145.06
141.34
140.77
138.20
137.29
136.47
135.63
130.04
129.62
129.43
128.08
127.46
126.75
124.68
124.47
123.77
120.55
116.74
116.05
115.01
111.53
77.48
77.16
76.84
75.52
31.02
21.72
N
S
O
O
O
O
N
5
$^{13}C\{^{1}H\}$, $CDCl_3$, 101 MHz
220 210 200 190 180 170 160 150 140 130 120 110 100 90 80 70 60 50 40 30 20 10 0
f1 (ppm)

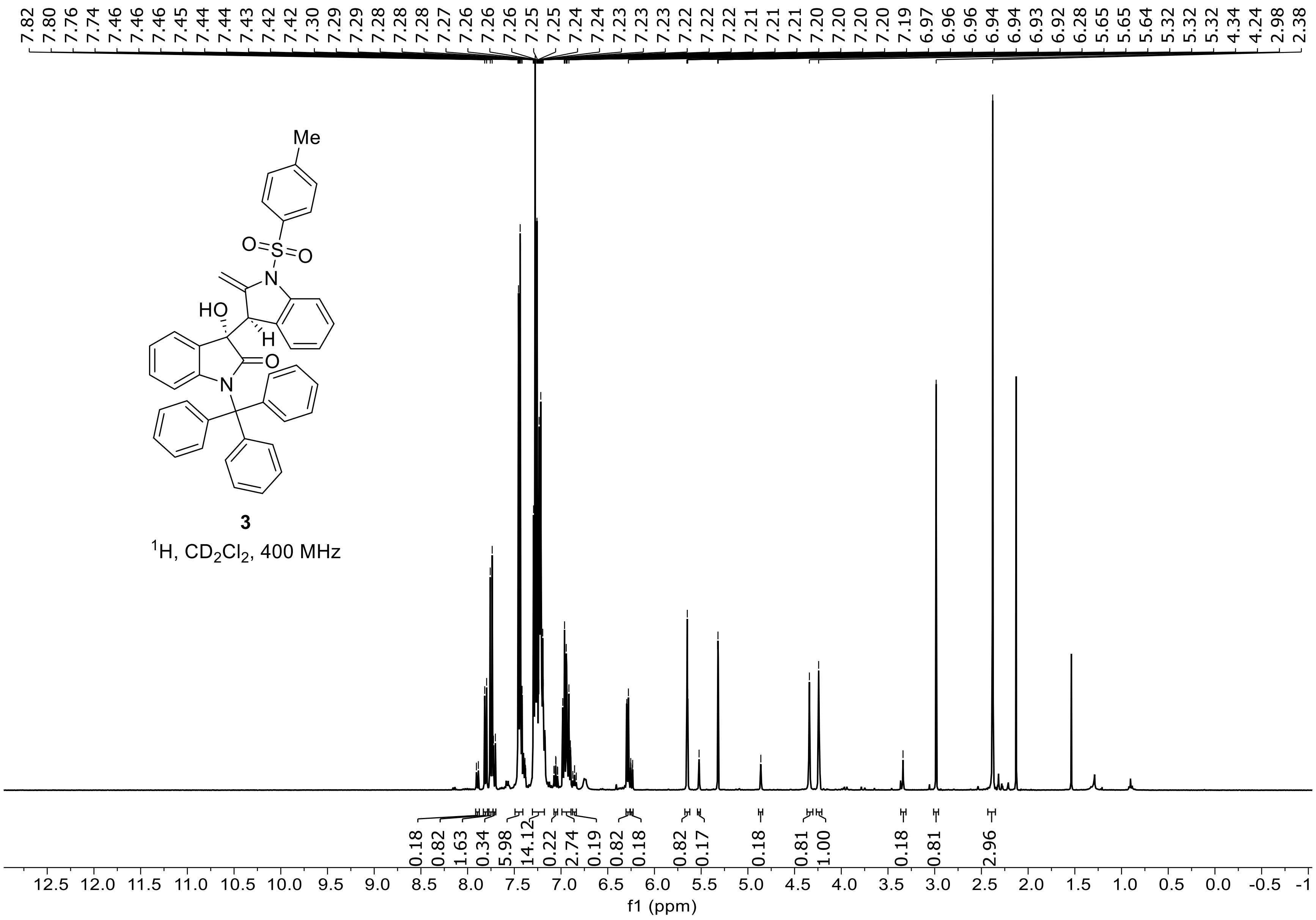

3

^1H, CD_2Cl_2, 400 MHz

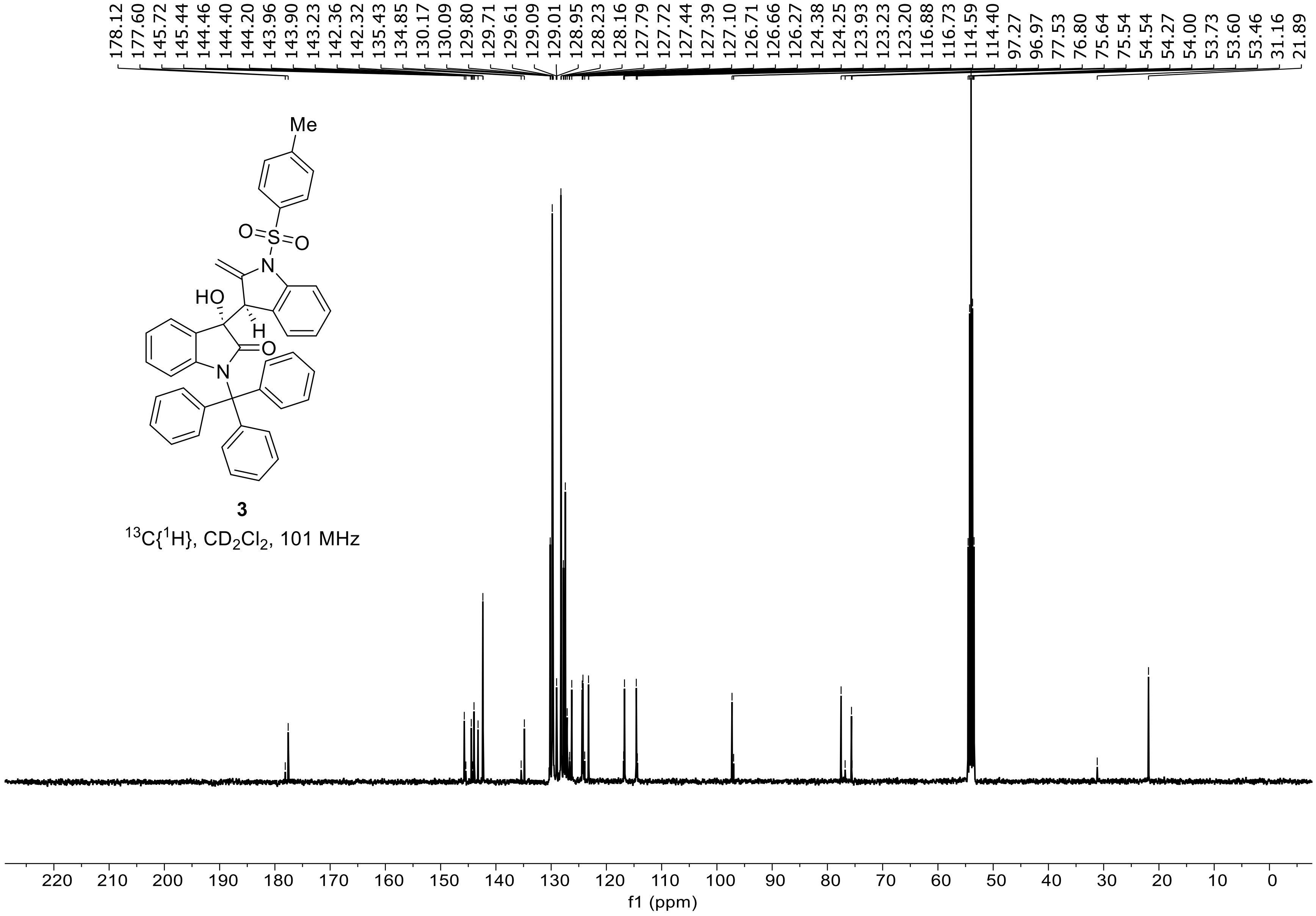
178.12 177.60 145.72 145.44 144.46 144.40 144.20 143.96 143.90 143.23 142.36 142.32 135.43 134.85 130.17 130.09 129.80 129.71 129.61 129.09 129.01 128.95 128.23 128.16 127.79 127.72 127.44 127.39 127.10 126.71 126.66 126.27 124.38 124.25 123.93 123.23 123.20 116.88 116.73 114.59 114.40 97.27 96.97 77.53 76.80 75.64 75.54 54.54 54.27 54.00 53.73 53.60 53.46 31.16 21.89
Me
O=S=O
N
HO
H
O
N
3
13C{1H}, CD2Cl2, 101 MHz
220 210 200 190 180 170 160 150 140 130 120 110 100 90 80 70 60 50 40 30 20 10 0
f1 (ppm)

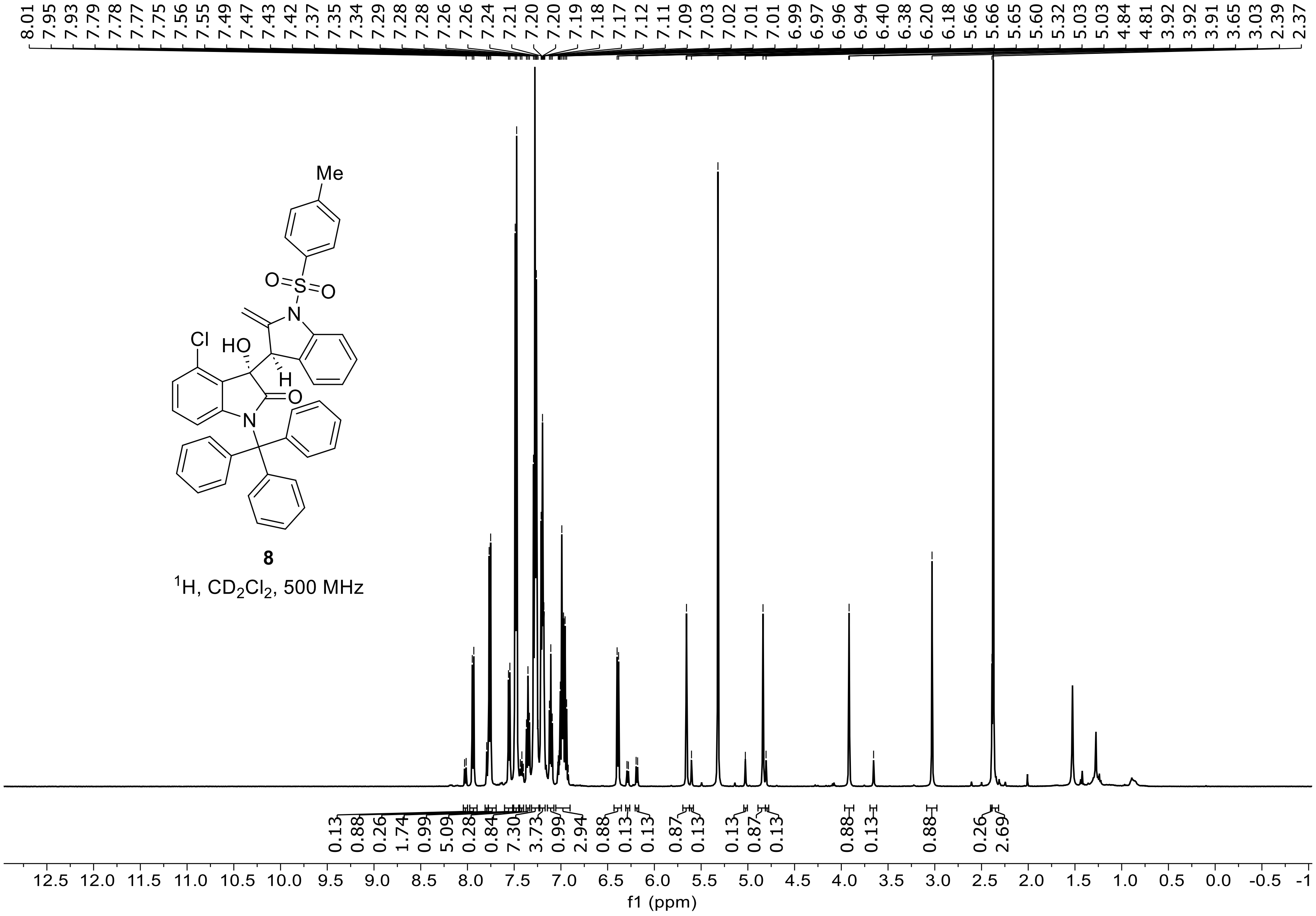

Me
O=S=O
N
Cl
HO
H
O
N
8
^{1}H, CD_2Cl_2, 500 MHz
f1 (ppm)

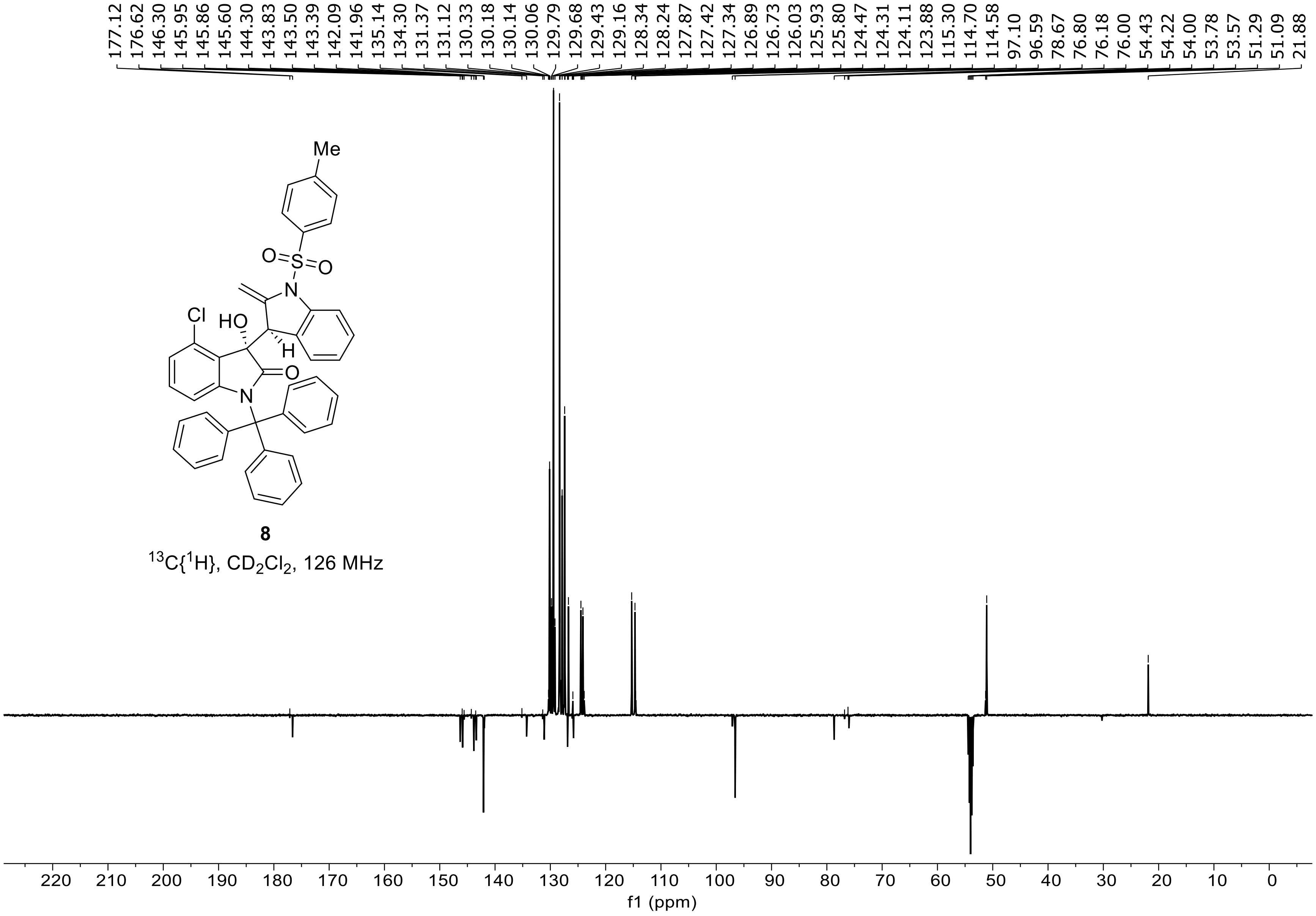
177.12
176.62
146.30
145.95
145.86
145.60
144.30
143.83
143.50
143.39
142.09
141.96
135.14
134.30
131.37
131.12
130.33
130.18
130.14
130.06
129.79
129.68
129.43
129.16
128.34
128.24
127.87
127.42
127.34
126.89
126.73
126.03
125.93
125.80
124.47
124.31
124.11
123.88
115.30
114.70
114.58
97.10
96.59
78.67
76.80
76.18
76.00
54.43
54.22
54.00
53.78
53.57
51.29
51.09
21.88
Me
O=S=O
N
Cl
HO
H
O
N
8
13C{1H}, CD2Cl2, 126 MHz
220 210 200 190 180 170 160 150 140 130 120 110 100 90 80 70 60 50 40 30 20 10 0
f1 (ppm)

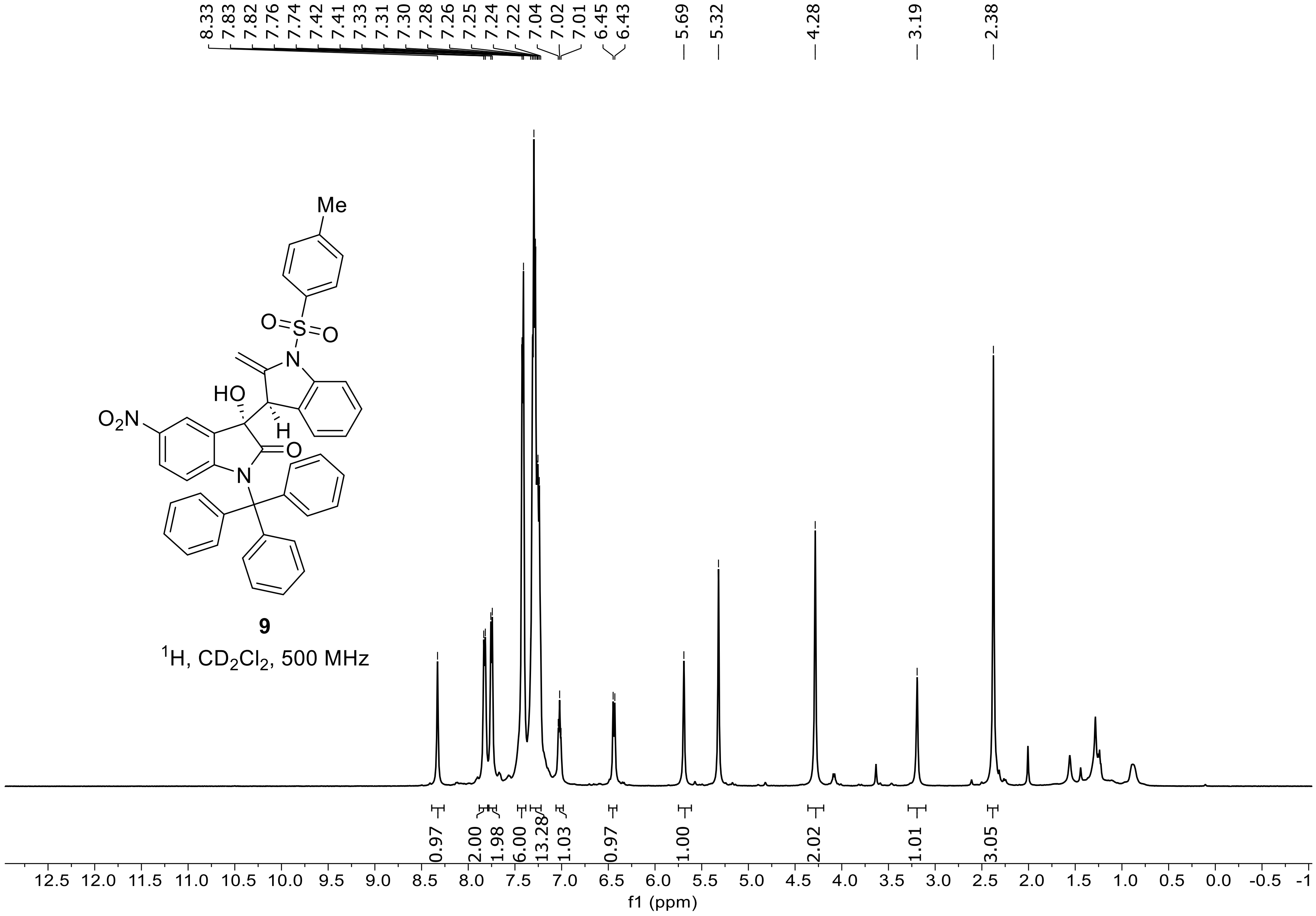

9

^{1}H, CD_2Cl_2, 500 MHz

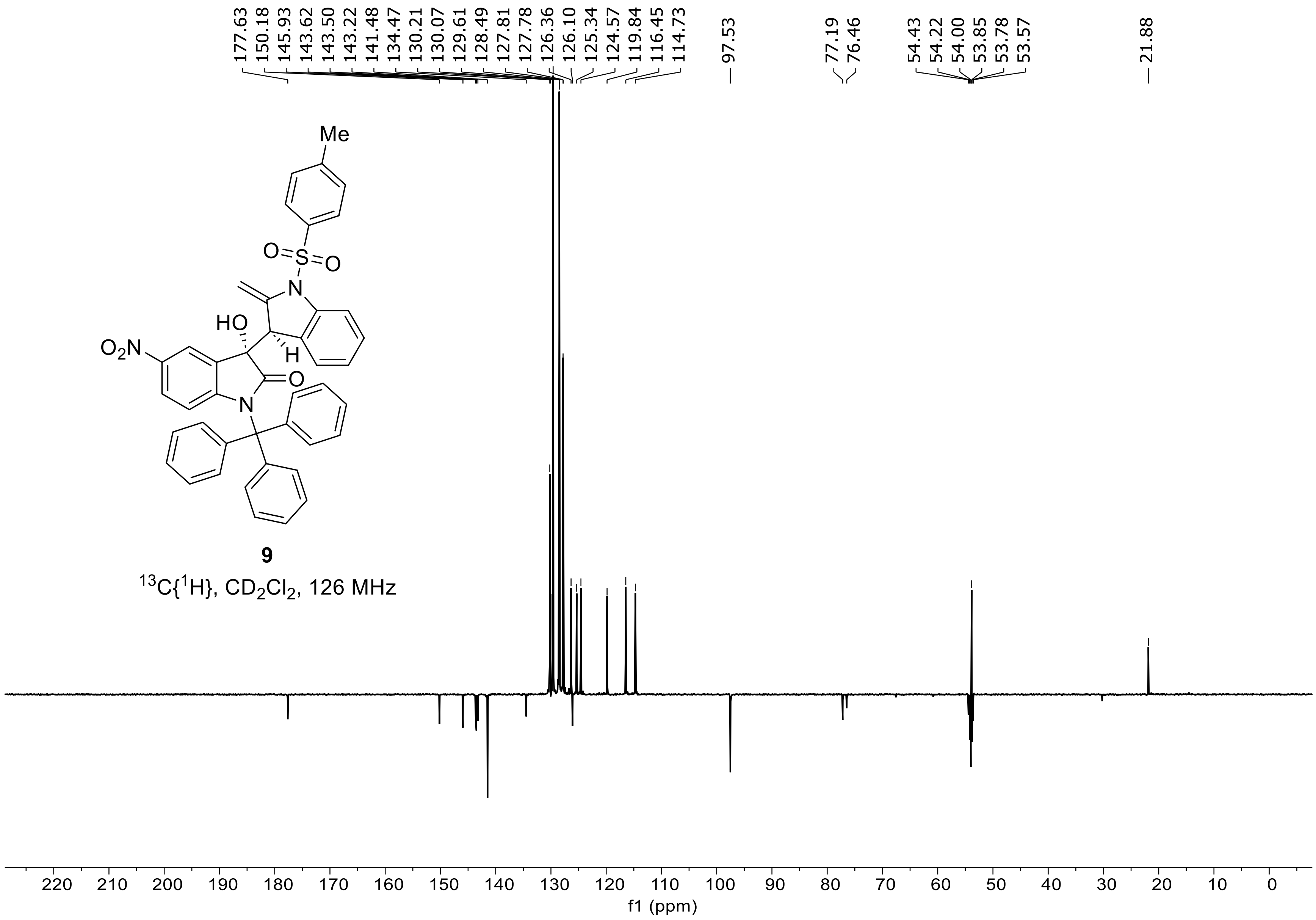
177.63
150.18
145.93
143.62
143.50
143.22
141.48
134.47
130.21
130.07
129.61
128.49
127.81
127.78
126.36
126.10
125.34
124.57
119.84
116.45
114.73
97.53
77.19
76.46
54.43
54.22
54.00
53.85
53.78
53.57
21.88
Me
O=S=O
N
HO
H
O_2N
O
N
9
$^{13}C\{^1H\}$, CD_2Cl_2, 126 MHz
220 210 200 190 180 170 160 150 140 130 120 110 100 90 80 70 60 50 40 30 20 10 0
f1 (ppm)

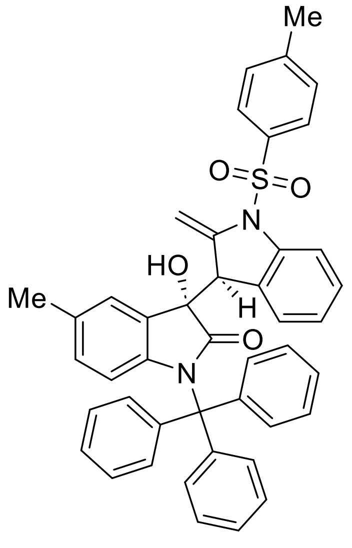

10

^{1}H, CD_2Cl_2, 500 MHz

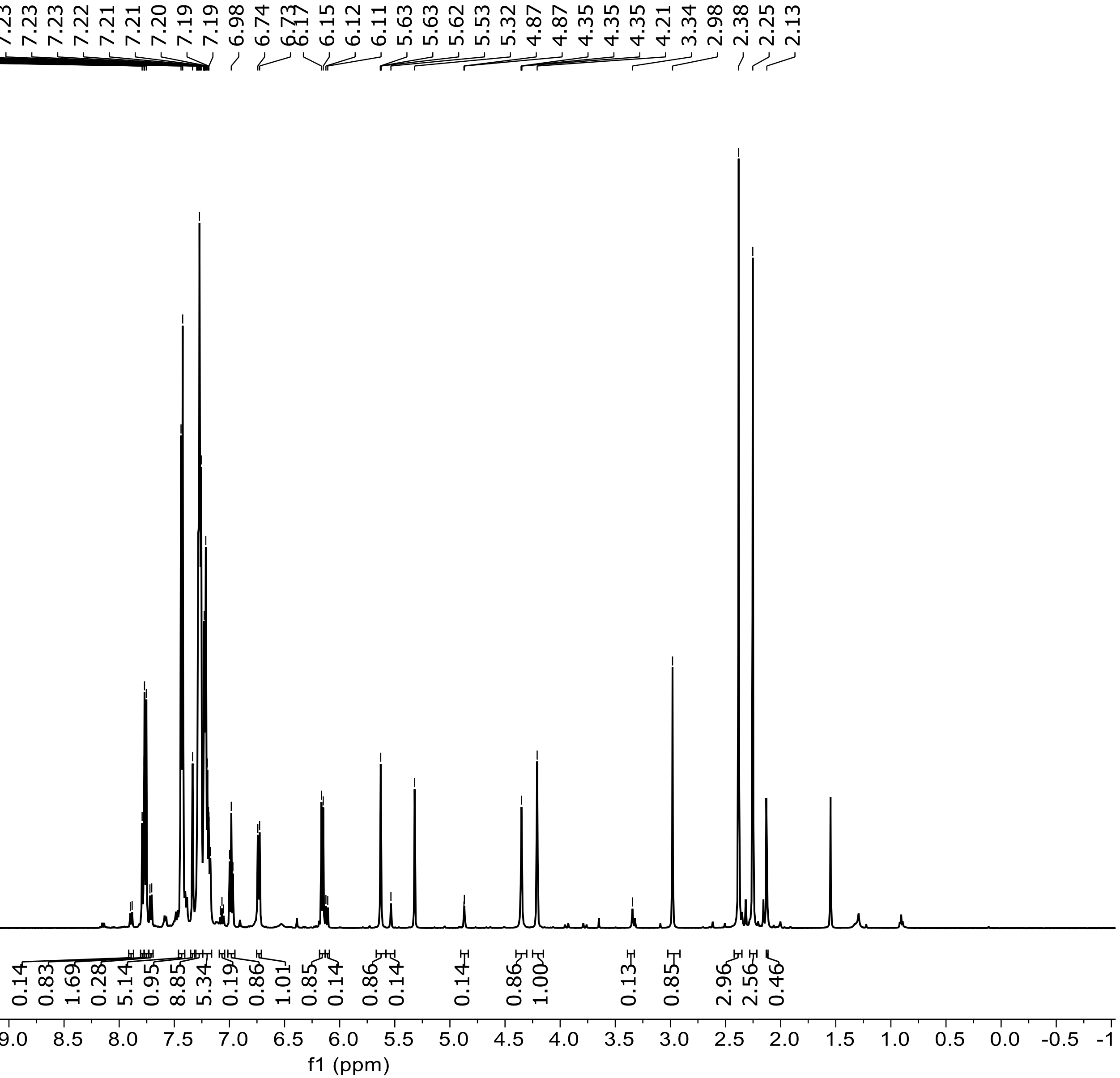

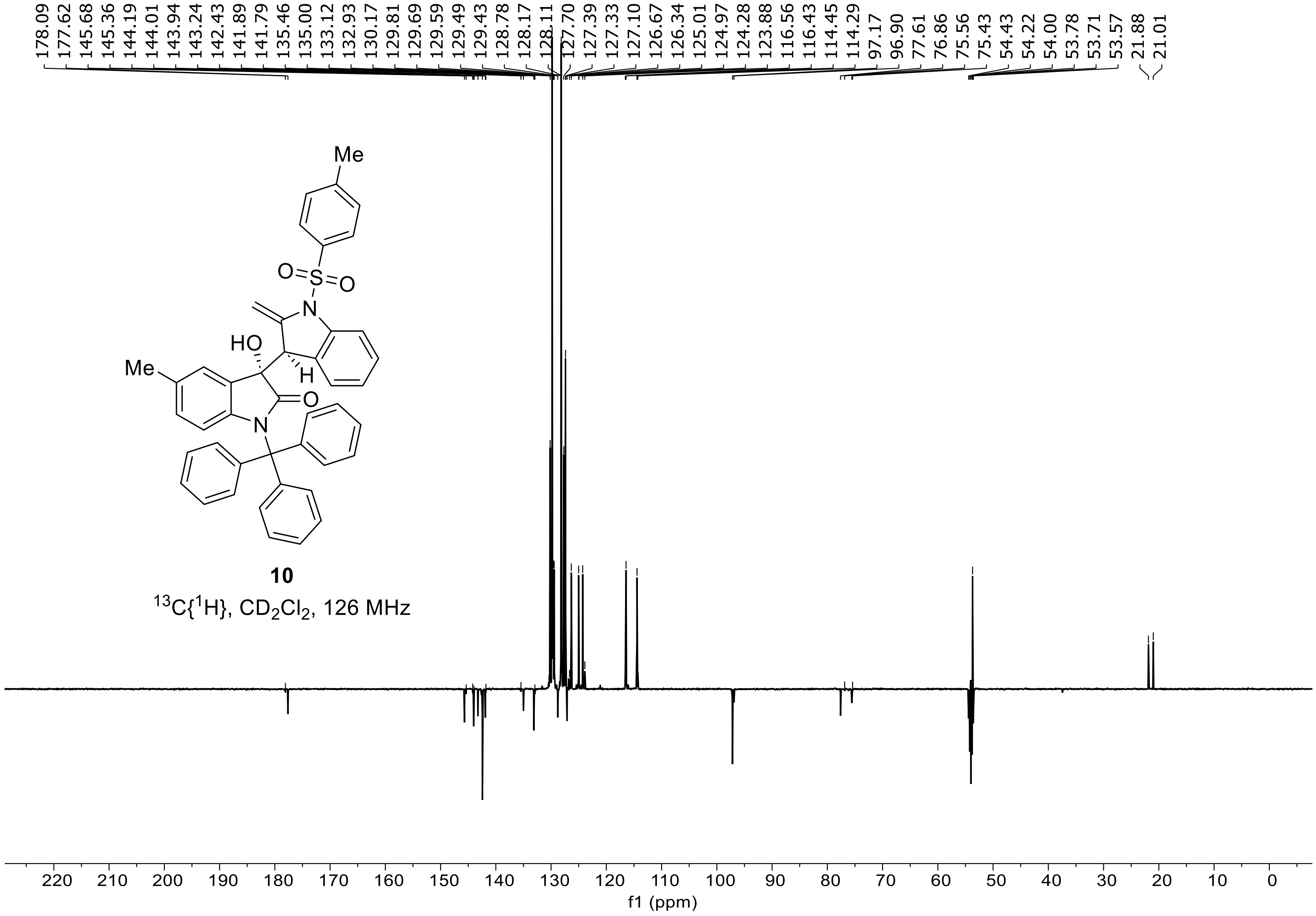
178.09
177.62
145.68
145.36
144.19
144.01
143.94
143.24
142.43
141.89
141.79
135.46
135.00
133.12
132.93
130.17
129.81
129.69
129.59
129.49
129.43
128.78
128.17
128.11
127.70
127.39
127.33
127.10
126.67
126.34
125.01
124.97
124.28
123.88
116.56
116.43
114.45
114.29
97.17
96.90
77.61
76.86
75.56
75.43
54.43
54.22
54.00
53.78
53.71
53.57
21.88
21.01
Me
O=S=O
N
HO
H
Me
O
N
10
$^{13}C\{^1H\}$, CD_2Cl_2, 126 MHz
220 210 200 190 180 170 160 150 140 130 120 110 100 90 80 70 60 50 40 30 20 10 0
f1 (ppm)

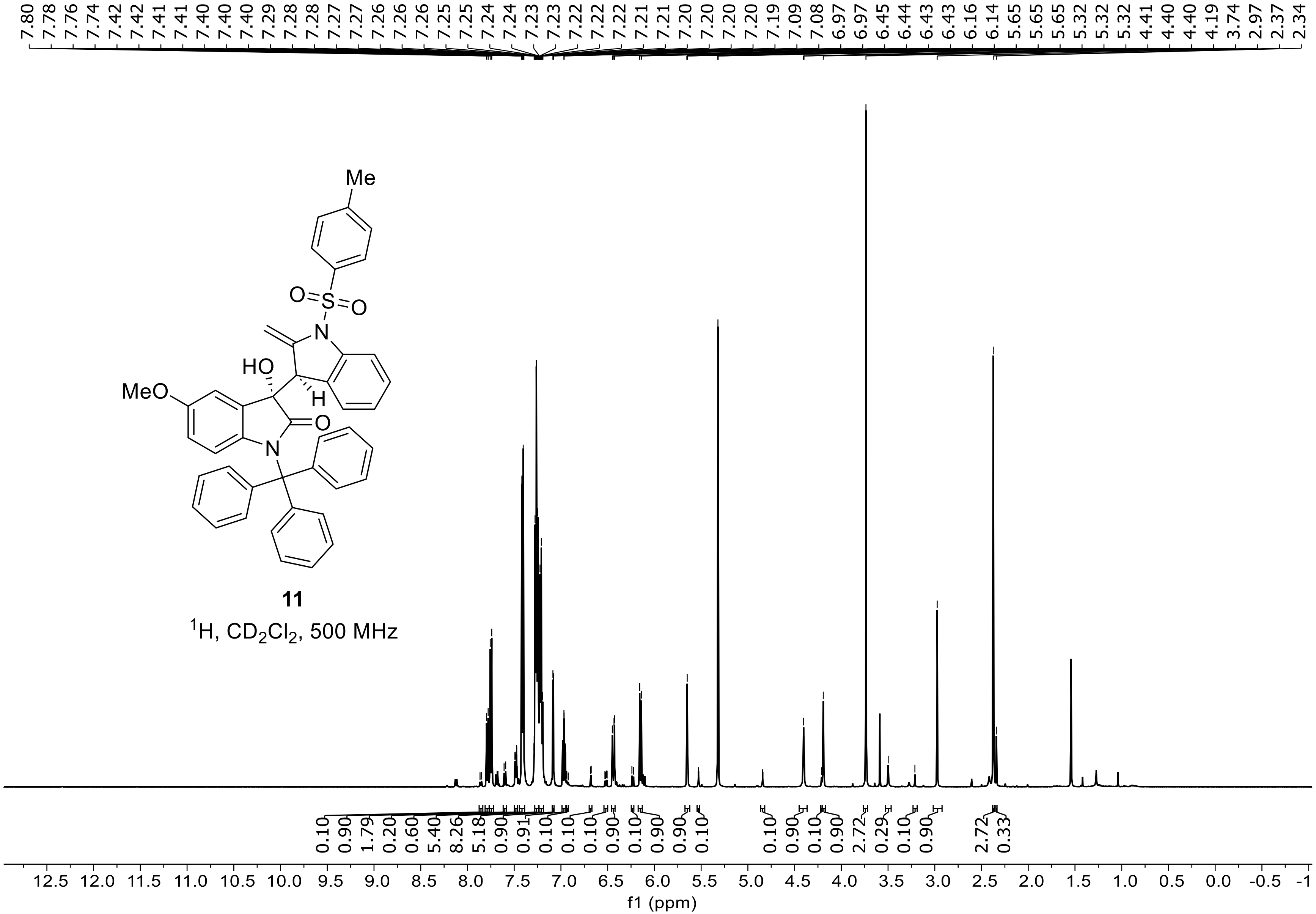

11

^{1}H, CD_2Cl_2, 500 MHz

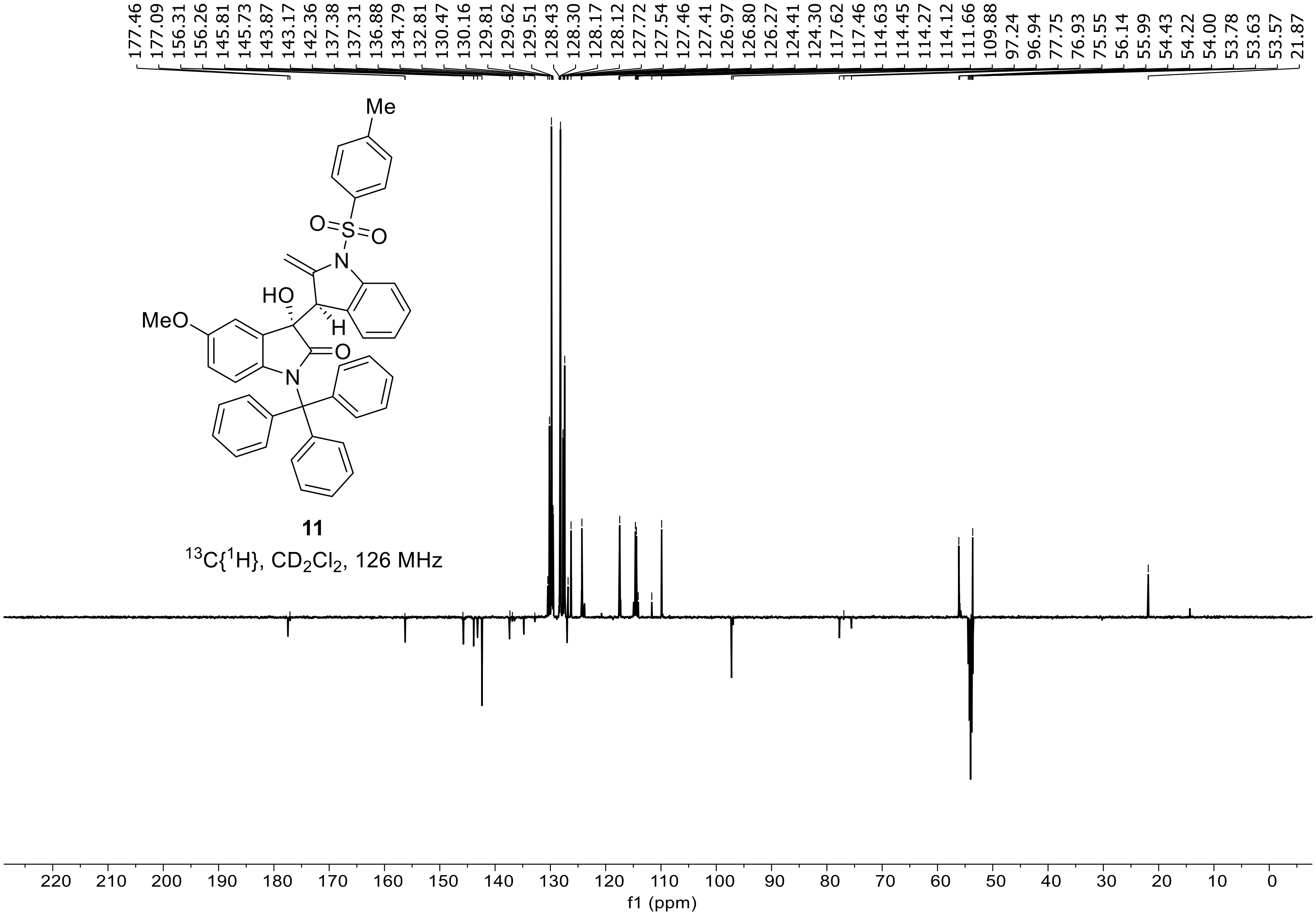

11

$^{13}C\{^{1}H\}$, CD_2Cl_2, 126 MHz

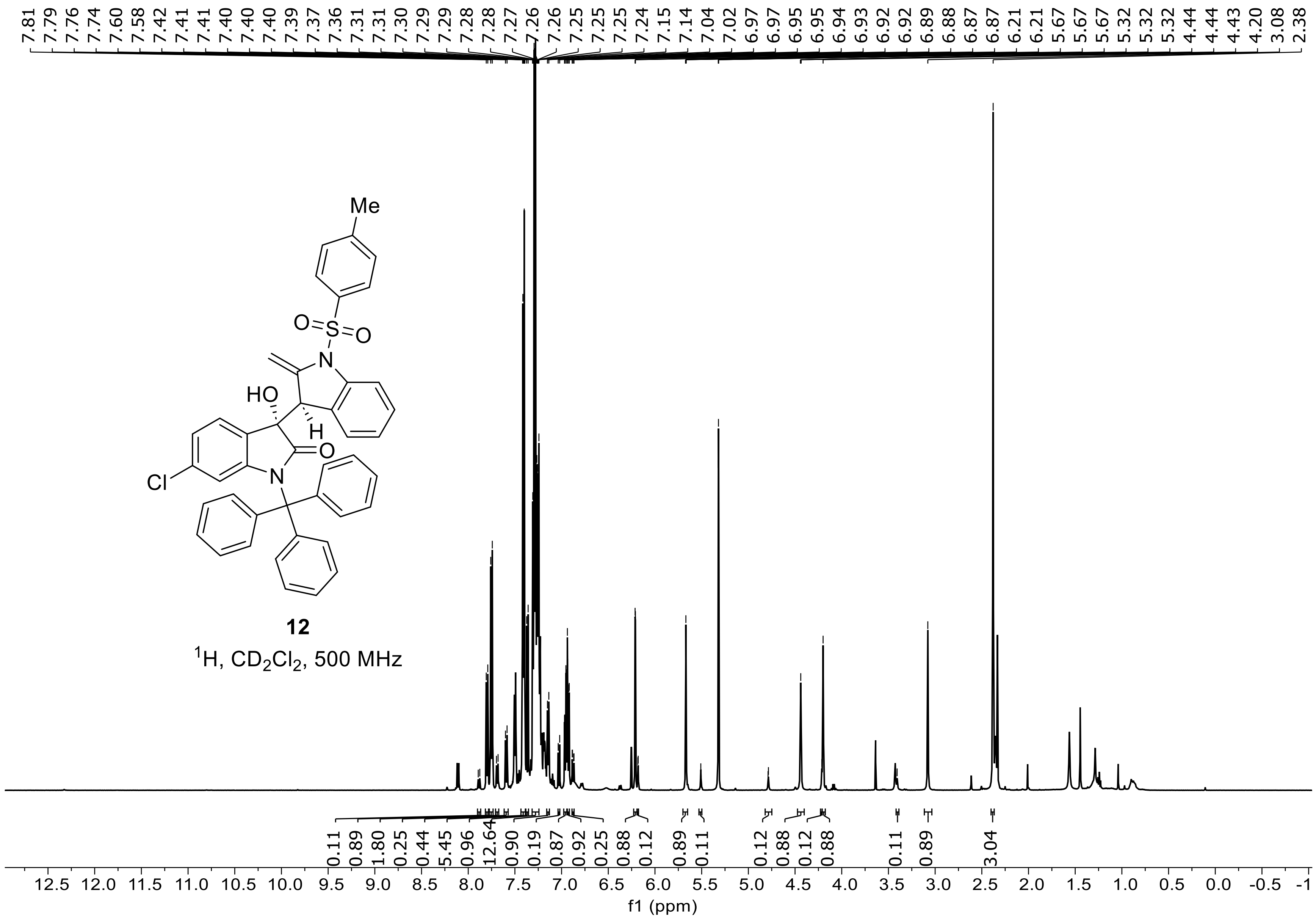

12

^1H, CD_2Cl_2, 500 MHz

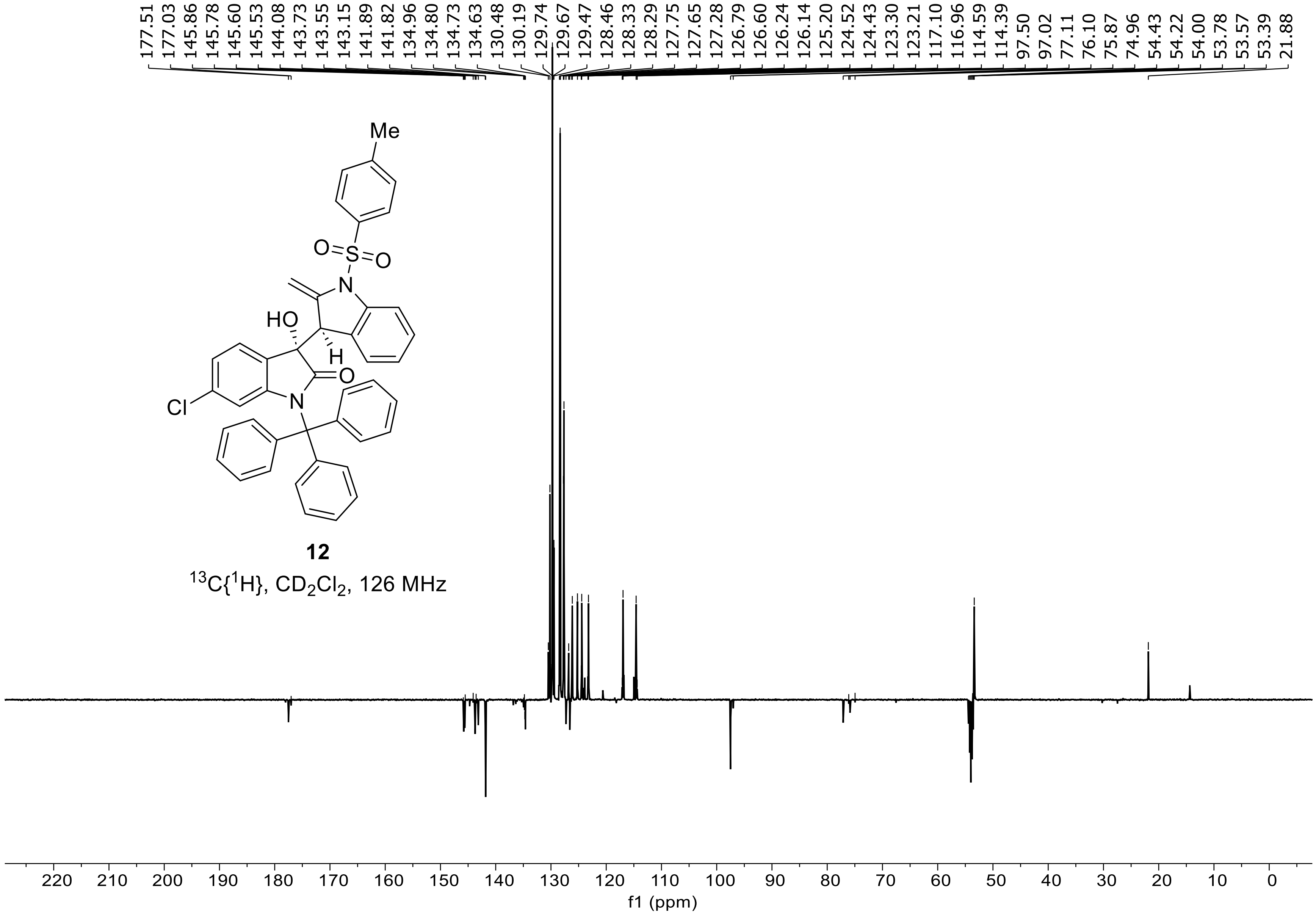

12

$^{13}C\{^{1}H\}$, CD_2Cl_2, 126 MHz

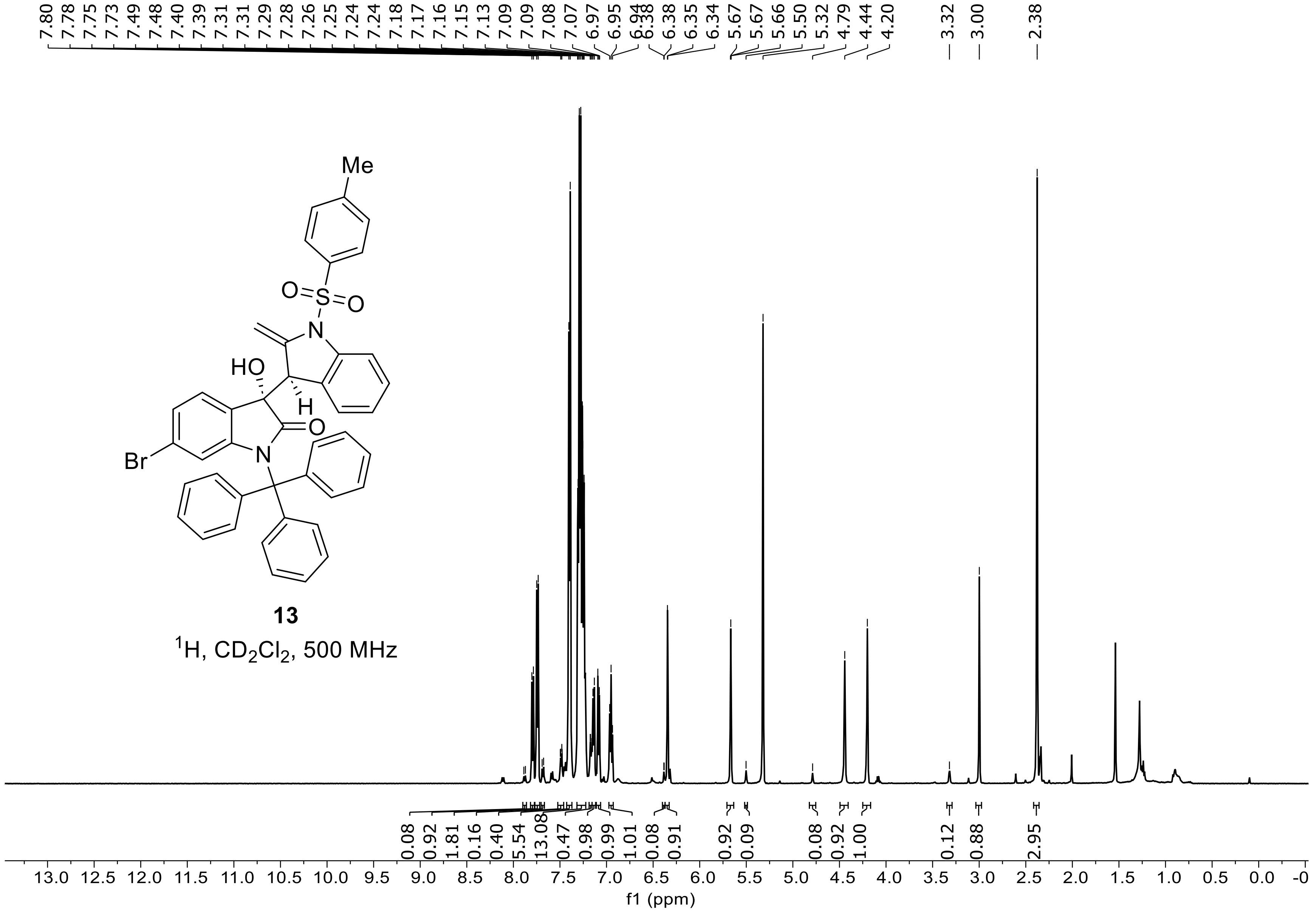

13
^1H, CD_2Cl_2, 500 MHz

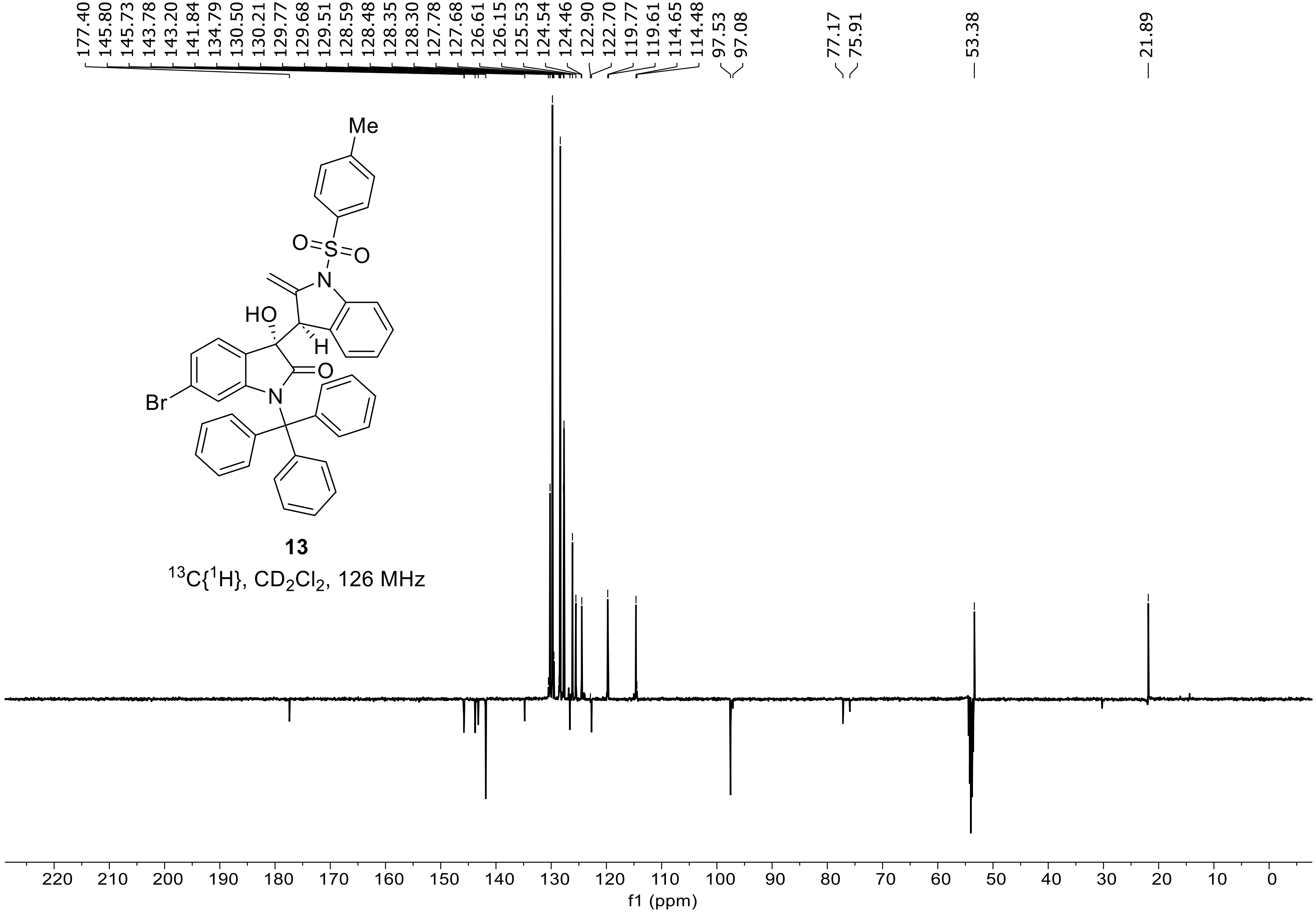

13

$^{13}C\{^{1}H\}$, CD_2Cl_2, 126 MHz

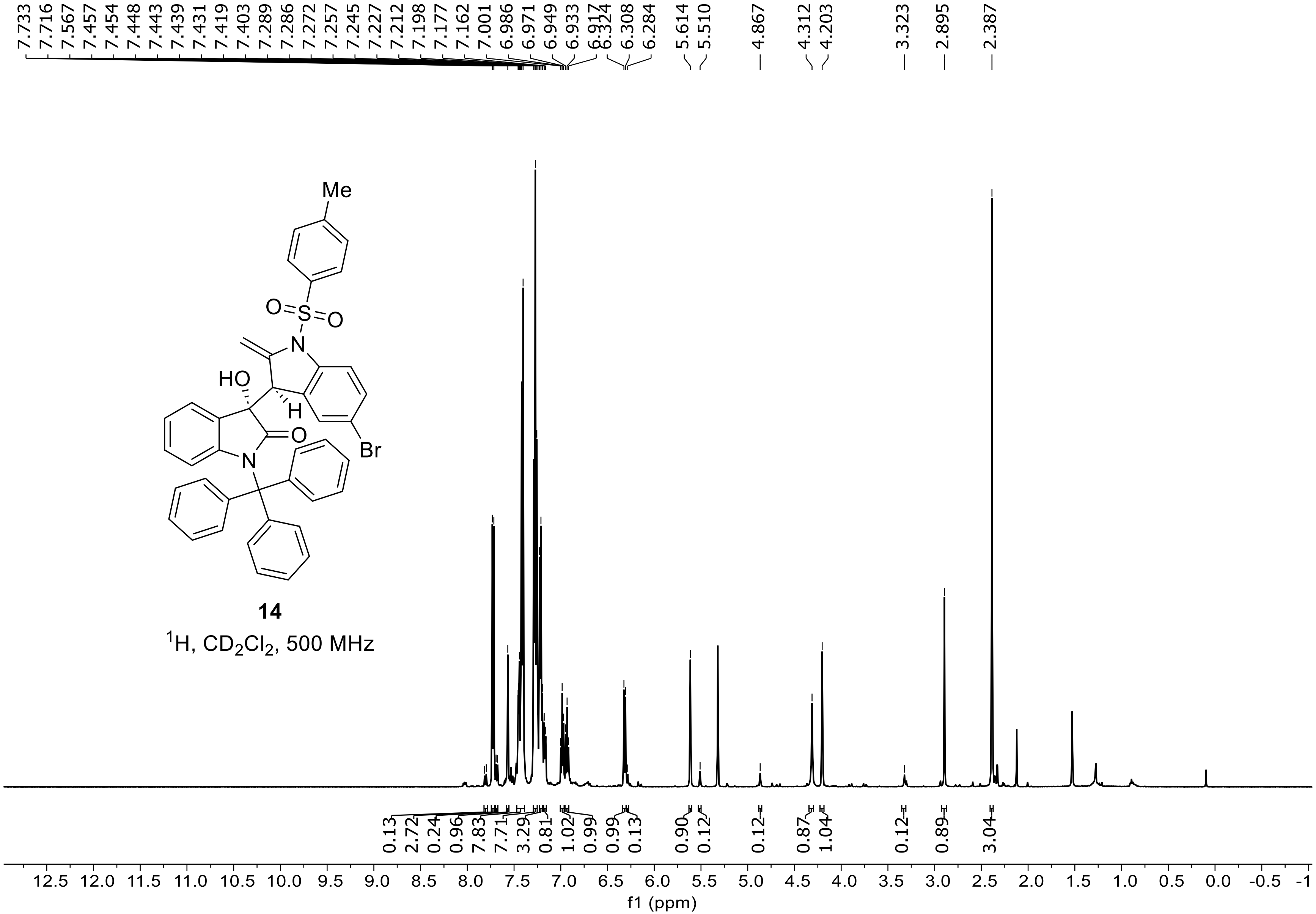
Me
O=S=O
N
HO
H
O
N
Br
14
^{1}H, CD_2Cl_2, 500 MHz
7.733
7.716
7.567
7.457
7.454
7.448
7.443
7.439
7.431
7.419
7.403
7.289
7.286
7.272
7.257
7.245
7.227
7.212
7.198
7.177
7.162
7.001
6.986
6.971
6.949
6.933
6.917
6.324
6.308
6.284
5.614
5.510
4.867
4.312
4.203
3.323
2.895
2.387
0.13
2.72
0.24
0.96
7.83
7.71
3.29
0.81
1.02
0.99
0.99
0.13
0.90
0.12
0.12
0.87
1.04
0.12
0.89
3.04
f1 (ppm)

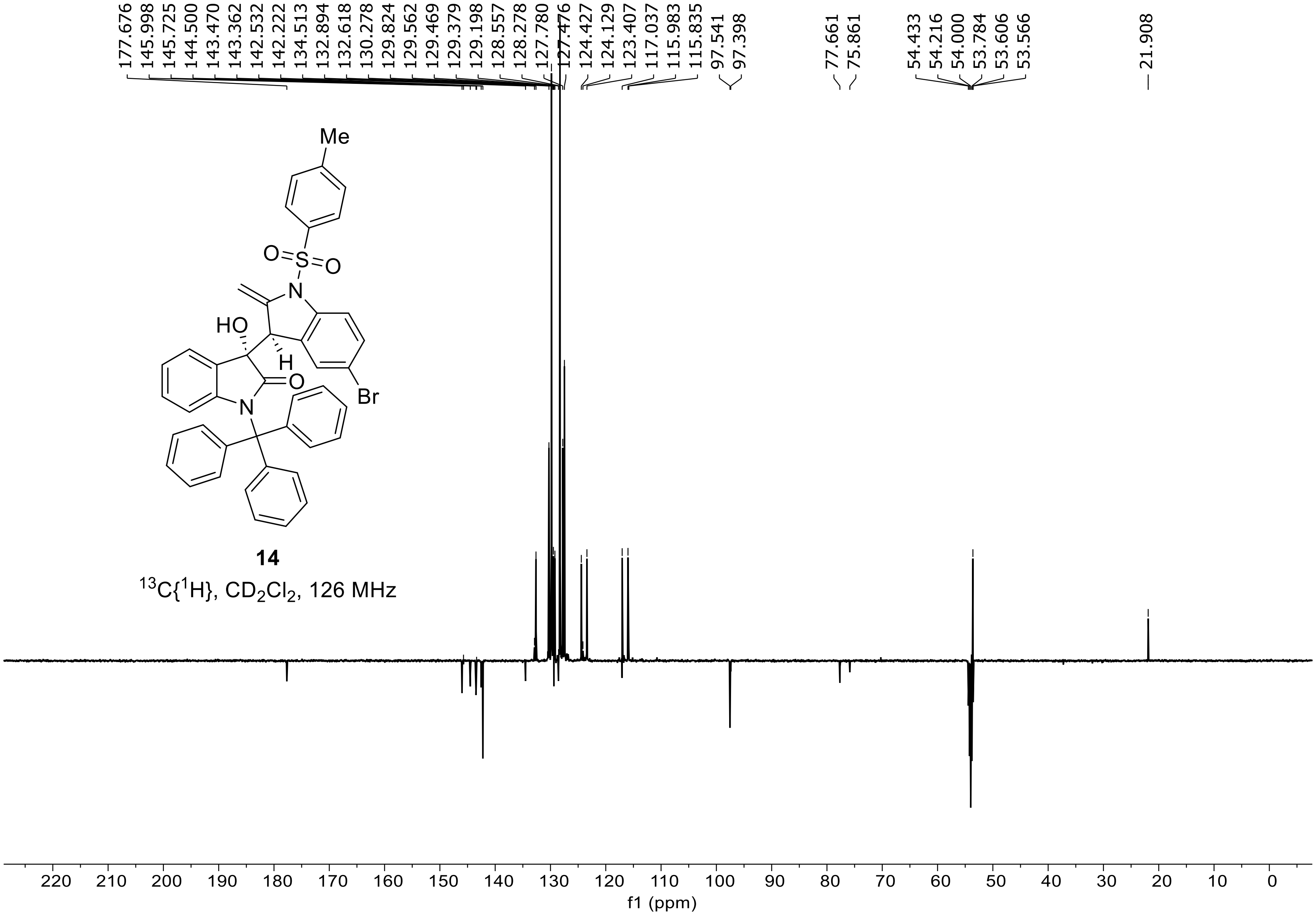
177.676
145.998
145.725
144.500
143.470
143.362
142.532
142.222
134.513
132.894
132.618
130.278
129.824
129.562
129.469
129.379
129.198
128.557
128.278
127.780
127.476
124.427
124.129
123.407
117.037
115.983
115.835
97.541
97.398
77.661
75.861
54.433
54.216
54.000
53.784
53.606
53.566
21.908
Me
O=S=O
N
HO
H
O
N
Br
14
$^{13}C\{^1H\}$, CD_2Cl_2, 126 MHz
220 210 200 190 180 170 160 150 140 130 120 110 100 90 80 70 60 50 40 30 20 10 0
f1 (ppm)

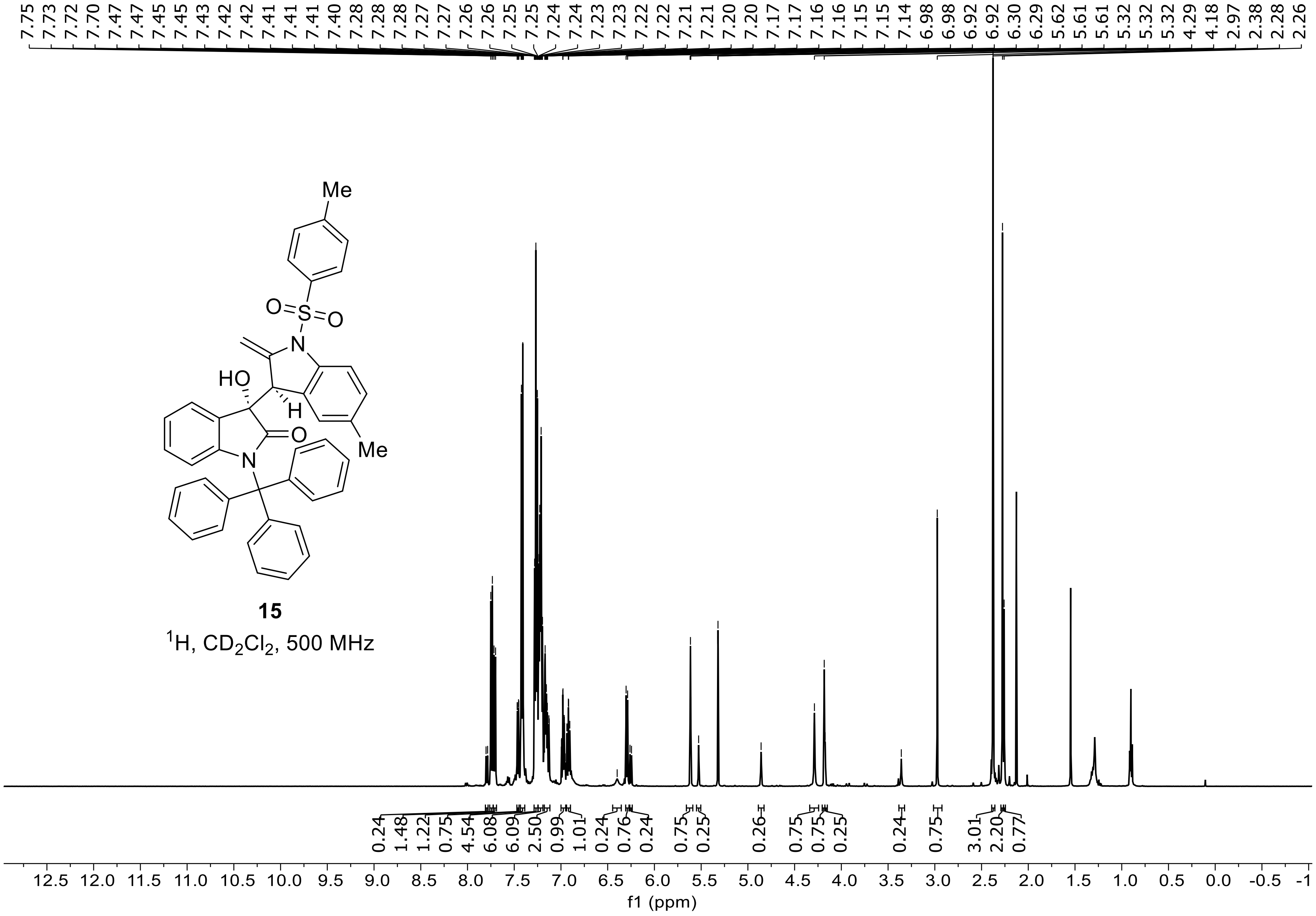

15

^{1}H, CD_2Cl_2, 500 MHz

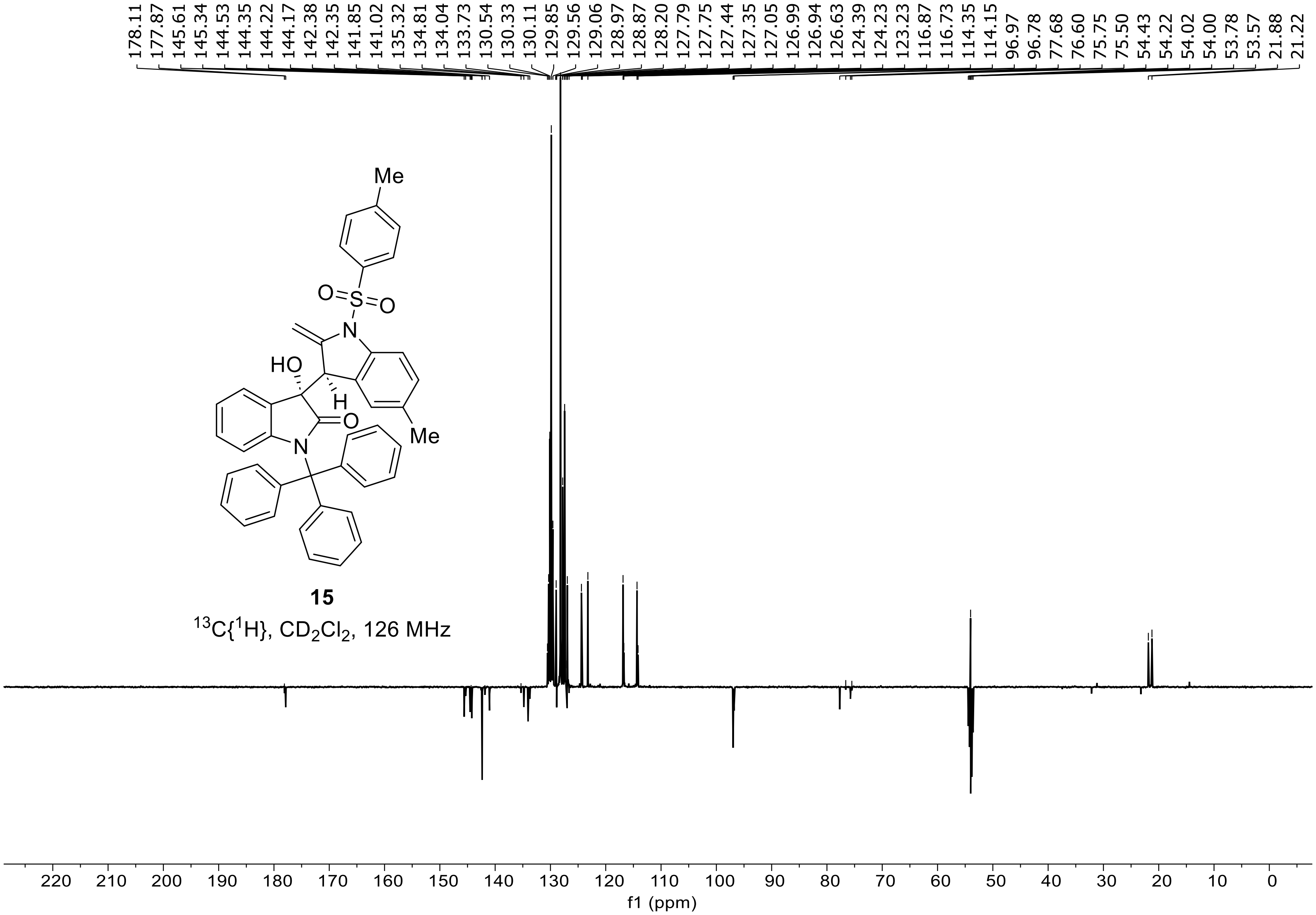
178.11
177.87
145.61
145.34
144.53
144.35
144.22
144.17
142.38
142.35
141.85
141.02
135.32
134.81
134.04
133.73
130.54
130.33
130.11
129.85
129.56
129.06
128.97
128.87
128.20
127.79
127.75
127.44
127.35
127.05
126.99
126.94
126.63
124.39
124.23
123.23
116.87
116.73
114.35
114.15
96.97
96.78
77.68
76.60
75.75
75.50
54.43
54.22
54.02
54.00
53.78
53.57
21.88
21.22
Me
O=S=O
N
HO
H
O
N
Me
15
$^{13}C\{^1H\}$, CD_2Cl_2, 126 MHz
220 210 200 190 180 170 160 150 140 130 120 110 100 90 80 70 60 50 40 30 20 10 0
f1 (ppm)

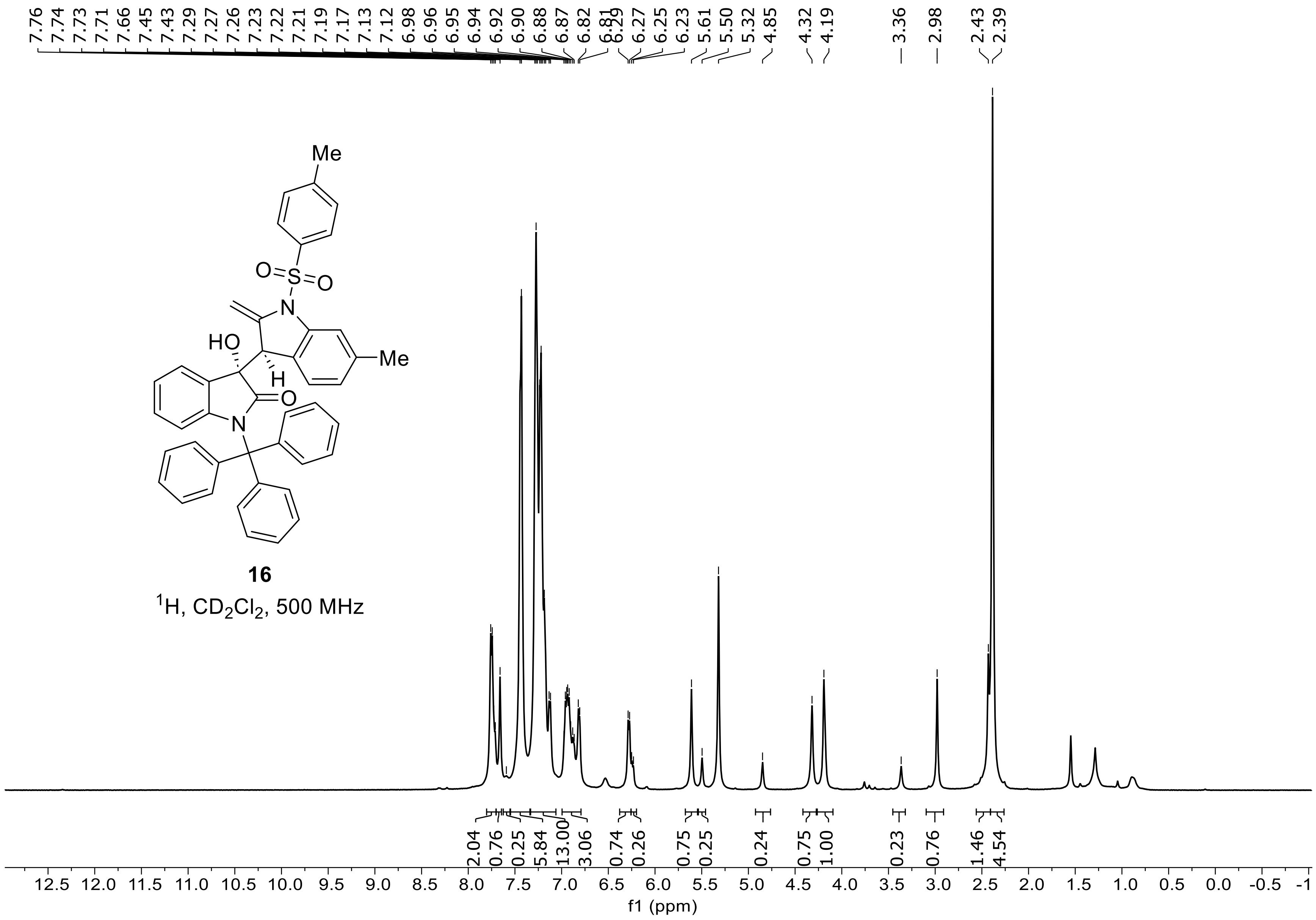
7.76 7.74 7.73 7.71 7.66 7.45 7.43 7.29 7.27 7.26 7.23 7.22 7.21 7.19 7.17 7.13 7.12 6.98 6.96 6.95 6.94 6.92 6.90 6.88 6.87 6.82 6.81 6.29 6.27 6.25 6.23 5.61 5.50 5.32 4.85 4.32 4.19 3.36 2.98 2.43 2.39
Me
O=S=O
N
HO
H
Me
O
N
16
^{1}H, CD$_2$Cl$_2$, 500 MHz
2.04 0.76 0.25 5.84 13.00 3.06 0.74 0.26 0.75 0.25 0.24 0.75 1.00 0.23 0.76 1.46 4.54
12.5 12.0 11.5 11.0 10.5 10.0 9.5 9.0 8.5 8.0 7.5 7.0 6.5 6.0 5.5 5.0 4.5 4.0 3.5 3.0 2.5 2.0 1.5 1.0 0.5 0.0 -0.5 -1
f1 (ppm)

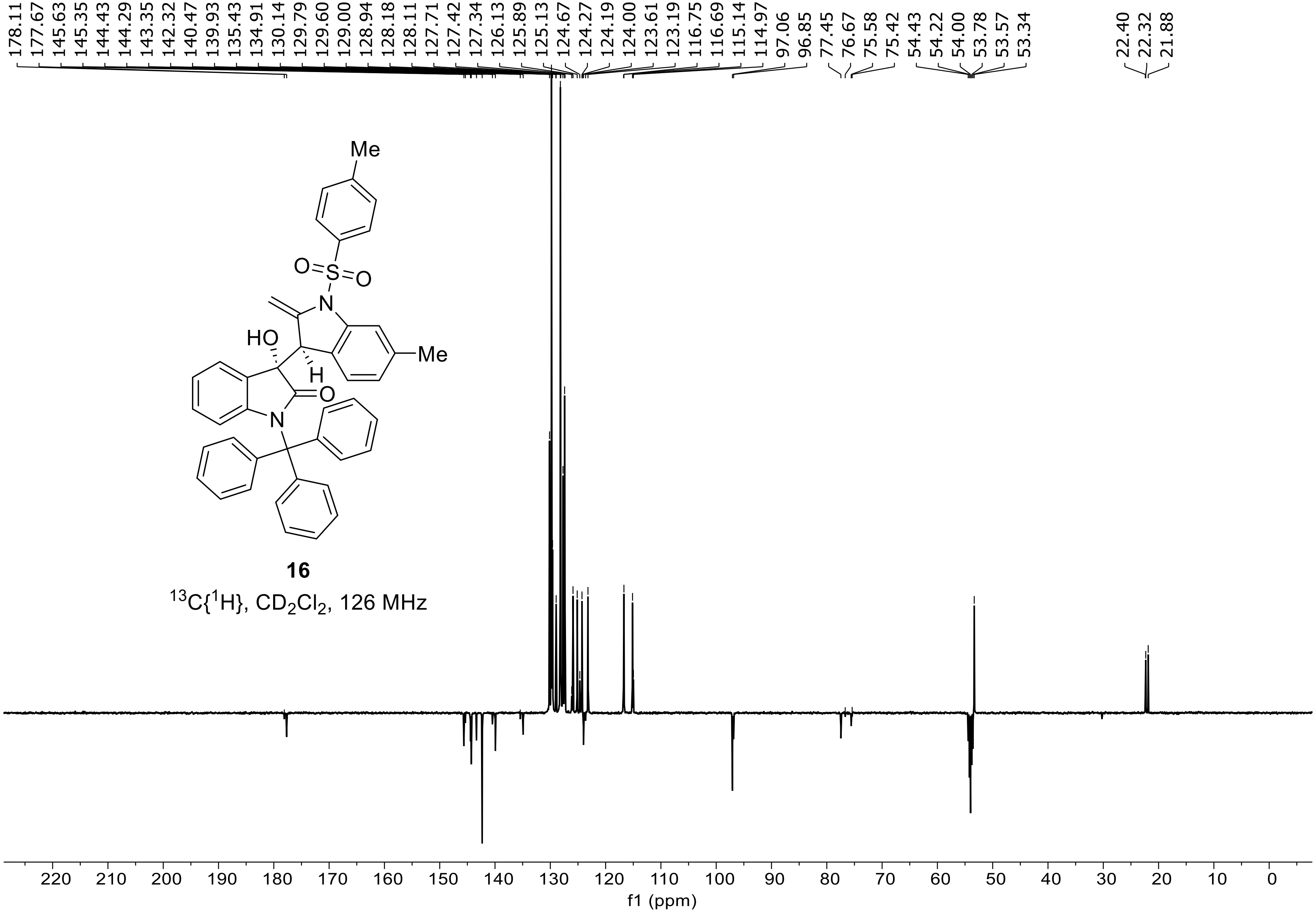
178.11
177.67
145.63
145.35
144.43
144.29
143.35
142.32
140.47
139.93
135.43
134.91
130.14
129.79
129.60
129.00
128.94
128.18
128.11
127.71
127.42
127.34
126.13
125.89
125.13
124.67
124.27
124.19
124.00
123.61
123.19
116.75
116.69
115.14
114.97
97.06
96.85
77.45
76.67
75.58
75.42
54.43
54.22
54.00
53.78
53.57
53.34
22.40
22.32
21.88
Me
O=S=O
N
HO
Me
H
O
N
16
$^{13}C\{^1H\}$, CD_2Cl_2, 126 MHz
220 210 200 190 180 170 160 150 140 130 120 110 100 90 80 70 60 50 40 30 20 10 0
f1 (ppm)

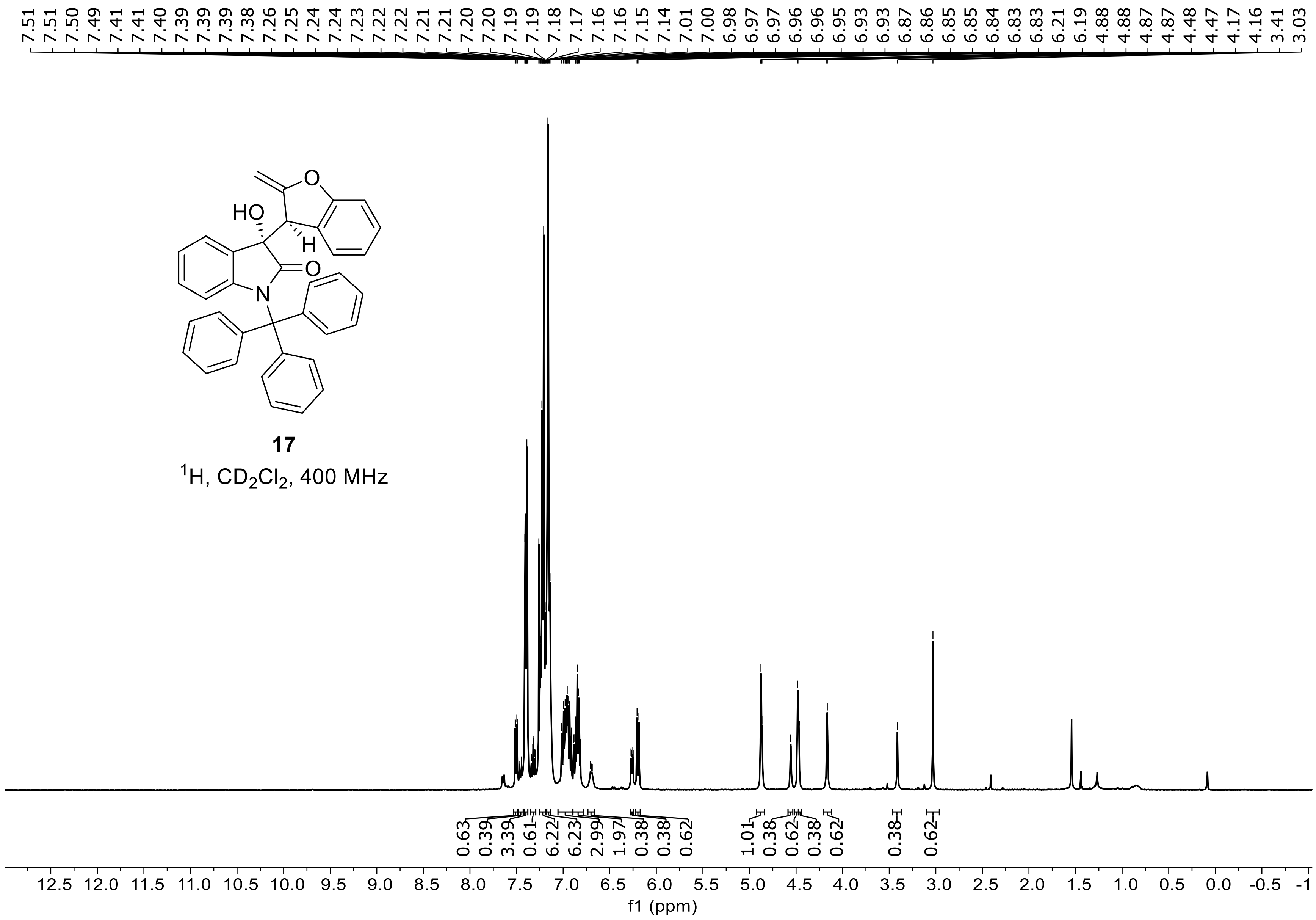
7.51 7.51 7.50 7.49 7.41 7.41 7.40 7.39 7.39 7.39 7.38 7.26 7.25 7.24 7.24 7.23 7.22 7.22 7.21 7.21 7.20 7.20 7.19 7.19 7.18 7.17 7.16 7.16 7.15 7.14 7.01 7.00 6.98 6.97 6.97 6.96 6.96 6.95 6.93 6.93 6.87 6.86 6.85 6.85 6.84 6.83 6.83 6.21 6.19 4.88 4.88 4.87 4.87 4.48 4.47 4.17 4.16 3.41 3.03
HO
O
H
N
17
^{1}H, CD_2Cl_2, 400 MHz
0.63 0.39 3.39 0.61 6.22 6.23 2.99 1.97 0.38 0.38 0.62 1.01 0.38 0.62 0.38 0.62 0.38 0.62
12.5 12.0 11.5 11.0 10.5 10.0 9.5 9.0 8.5 8.0 7.5 7.0 6.5 6.0 5.5 5.0 4.5 4.0 3.5 3.0 2.5 2.0 1.5 1.0 0.5 0.0 -0.5 -1
f1 (ppm)

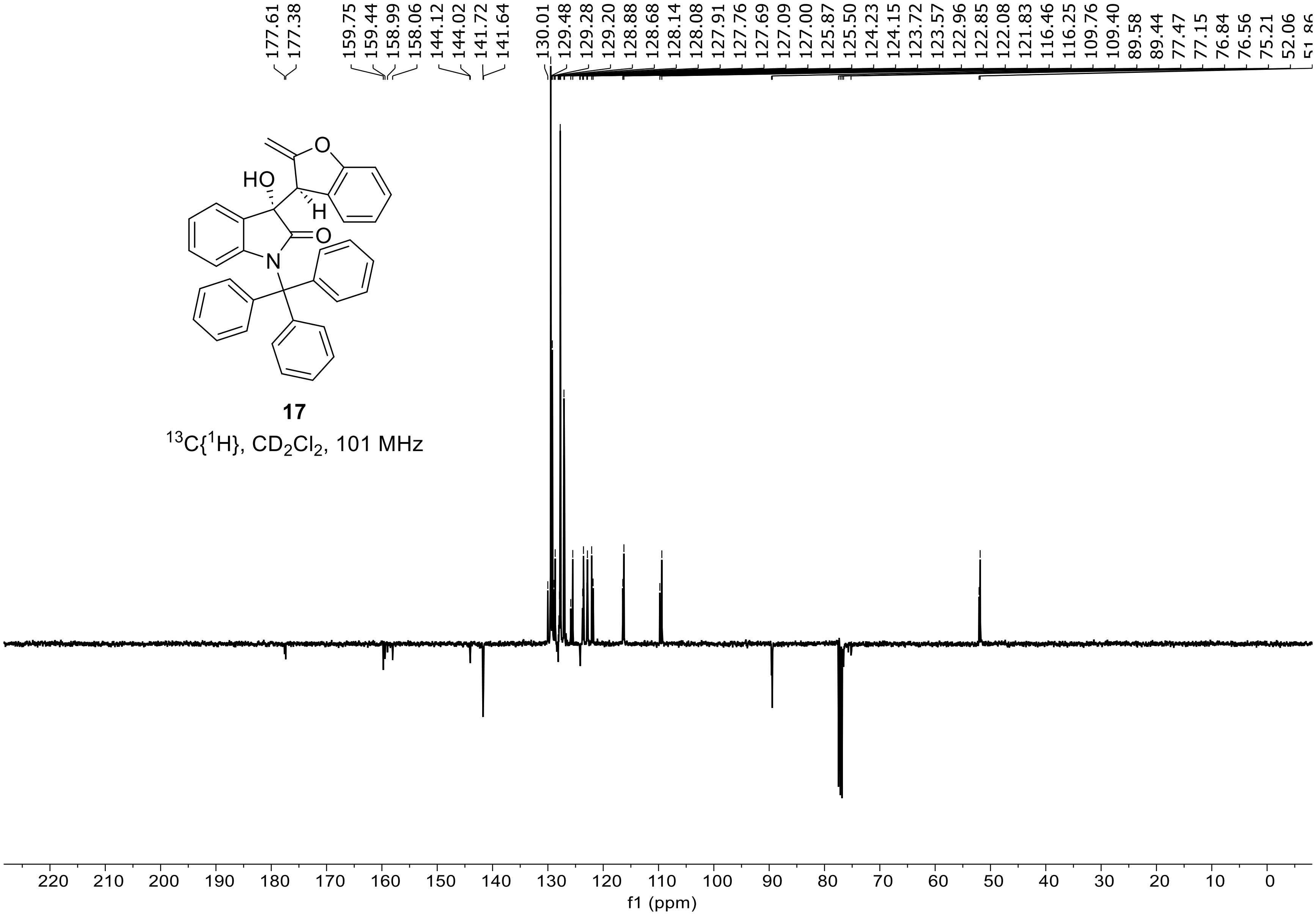

17

$^{13}C\{^1H\}$, CD_2Cl_2, 101 MHz

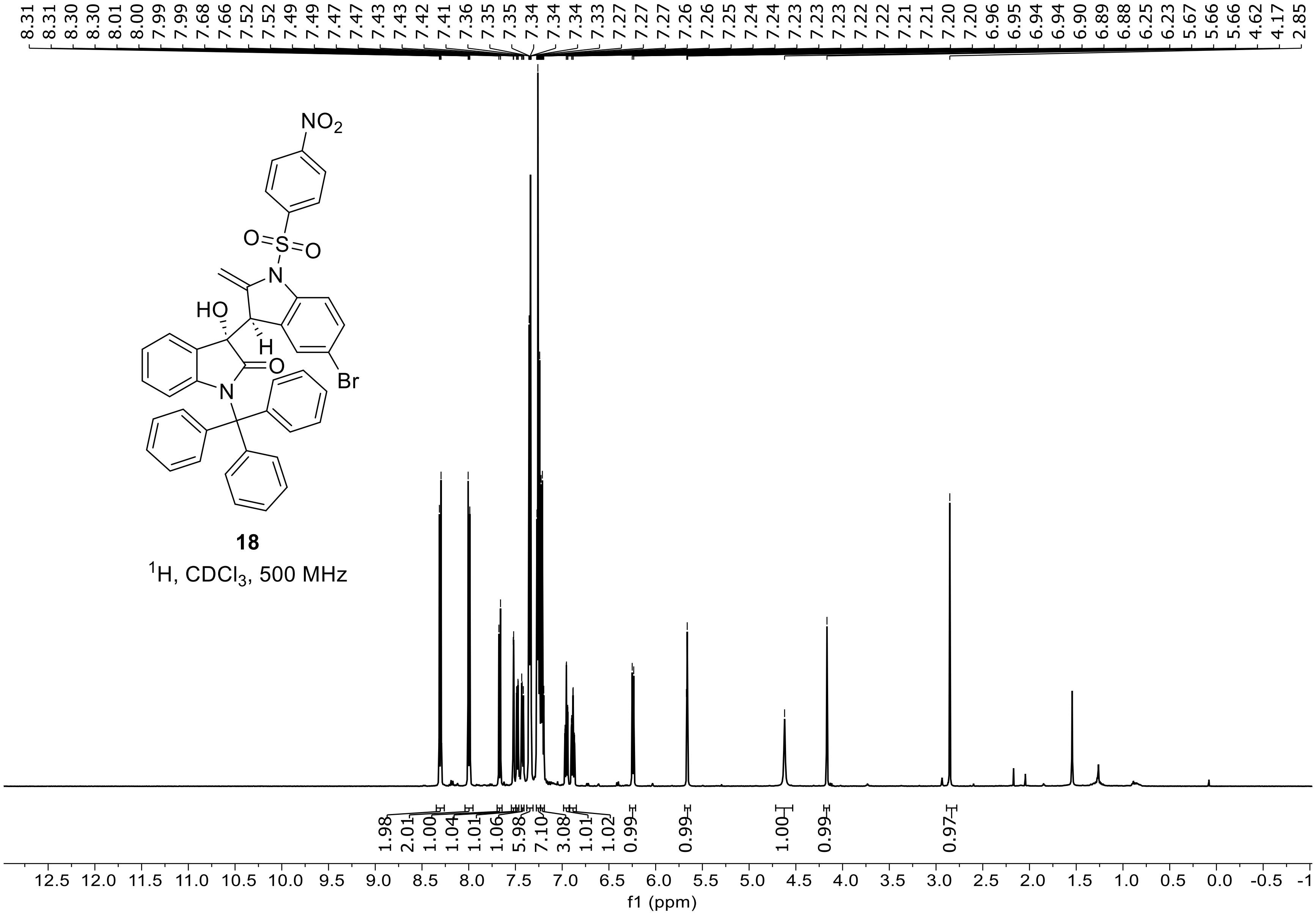

18

^{1}H, $CDCl_3$, 500 MHz

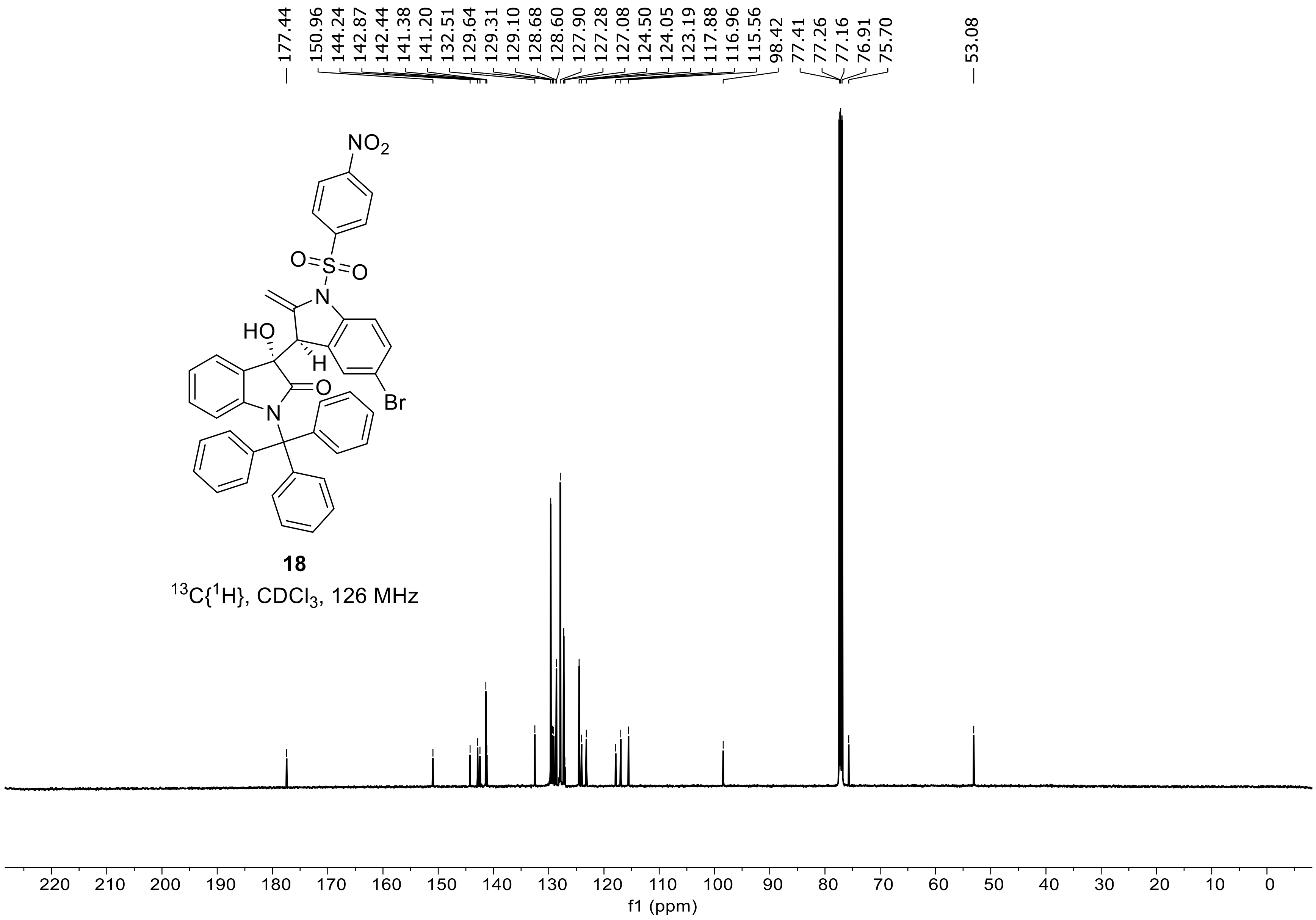
177.44
150.96
144.24
142.87
142.44
141.38
141.20
132.51
129.64
129.31
129.10
128.68
128.60
127.90
127.28
127.08
124.50
124.05
123.19
117.88
116.96
115.56
98.42
77.41
77.26
77.16
76.91
75.70
53.08
NO2
O=S=O
N
HO
H
O
N
Br
18
13C{1H}, CDCl3, 126 MHz
220 210 200 190 180 170 160 150 140 130 120 110 100 90 80 70 60 50 40 30 20 10 0
f1 (ppm)

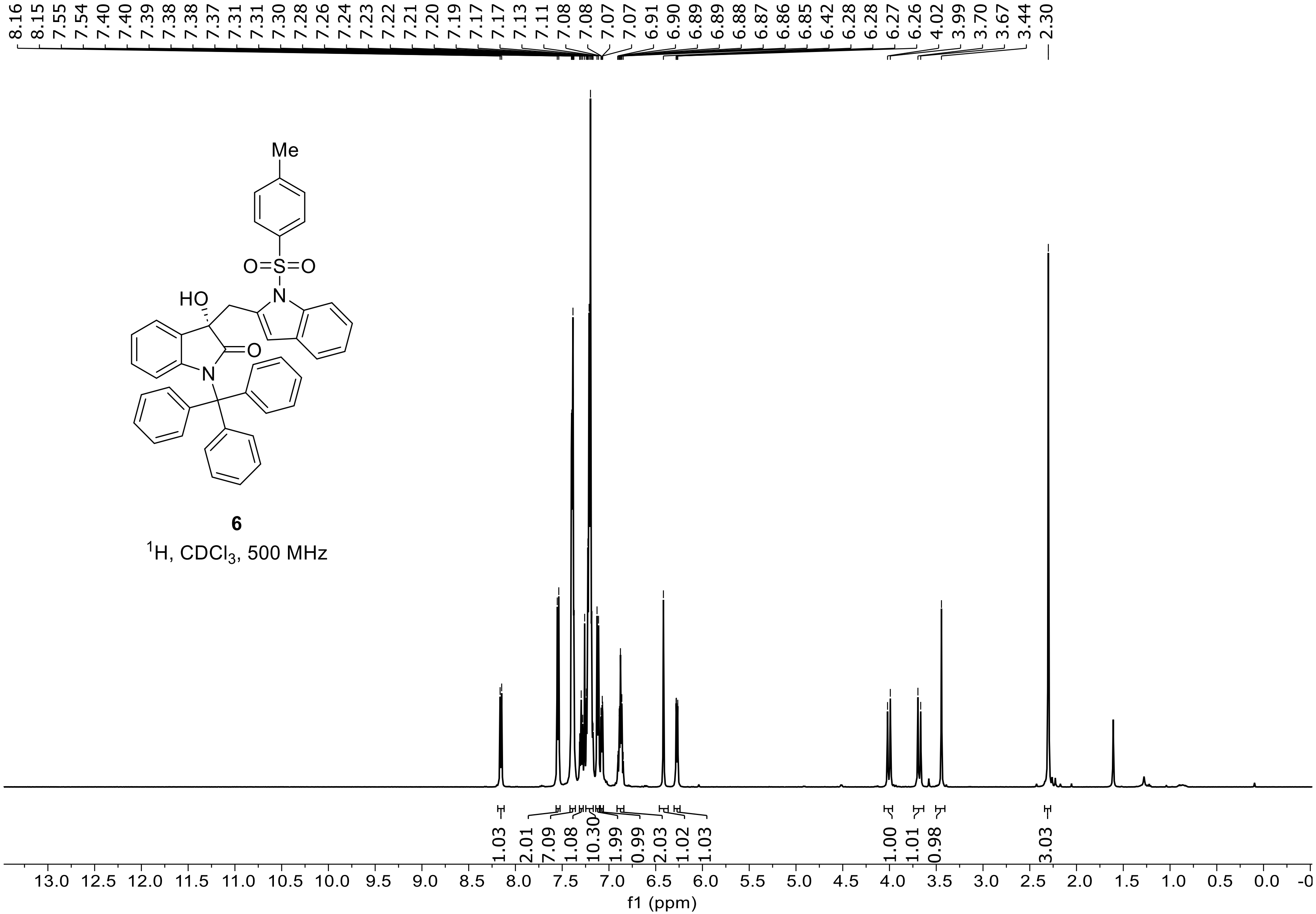
8.16 8.15 7.55 7.54 7.40 7.40 7.39 7.38 7.38 7.37 7.31 7.31 7.30 7.28 7.26 7.24 7.23 7.22 7.21 7.20 7.19 7.17 7.17 7.13 7.11 7.08 7.08 7.07 7.07 6.91 6.90 6.89 6.89 6.88 6.87 6.86 6.85 6.42 6.28 6.28 6.27 6.26 4.02 3.99 3.70 3.67 3.44 2.30
Me
O=S=O
N
HO
O
N
6
^{1}H, CDCl$_3$, 500 MHz
1.03 2.01 7.09 1.08 10.30 1.99 0.99 2.03 1.02 1.03 1.00 1.01 0.98 3.03
13.0 12.5 12.0 11.5 11.0 10.5 10.0 9.5 9.0 8.5 8.0 7.5 7.0 6.5 6.0 5.5 5.0 4.5 4.0 3.5 3.0 2.5 2.0 1.5 1.0 0.5 0.0 -0
f1 (ppm)

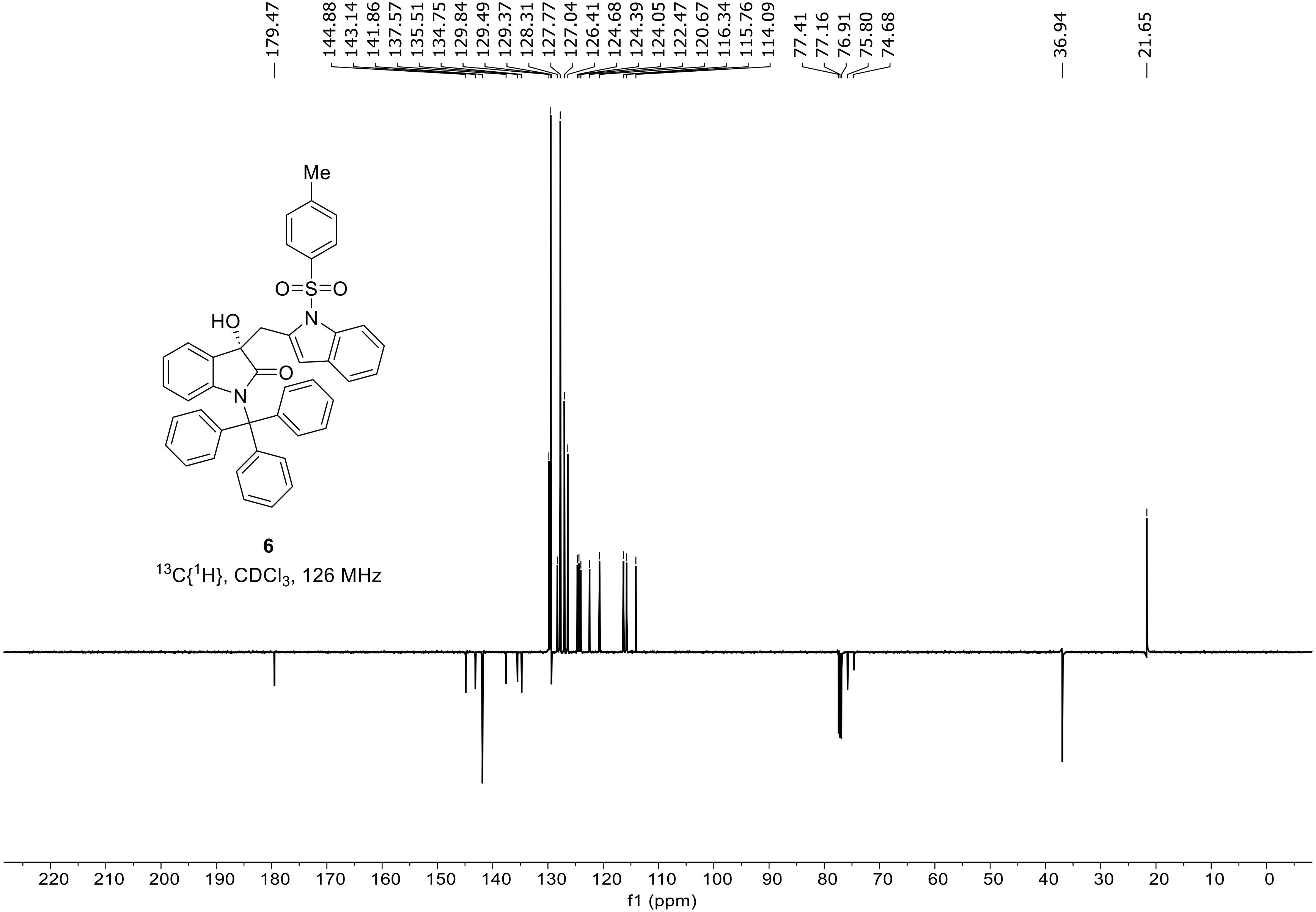
179.47
144.88
143.14
141.86
137.57
135.51
134.75
129.84
129.49
129.37
128.31
127.77
127.04
126.41
124.68
124.39
124.05
122.47
120.67
116.34
115.76
114.09
77.41
77.16
76.91
75.80
74.68
36.94
21.65
Me
O=S=O
HO
N
O
N
6
13C{1H}, CDCl3, 126 MHz
220 210 200 190 180 170 160 150 140 130 120 110 100 90 80 70 60 50 40 30 20 10 0
f1 (ppm)

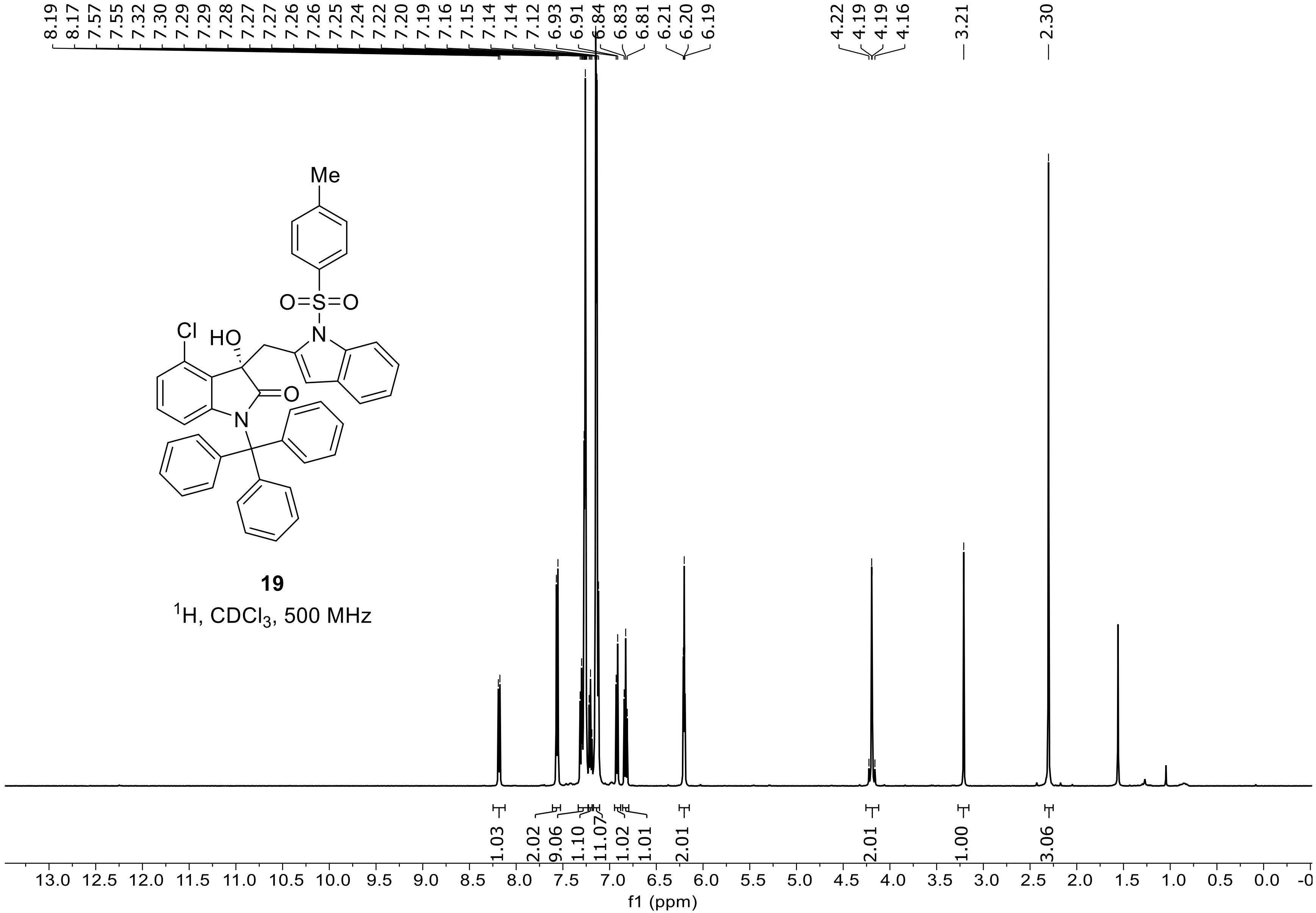
Me
O=S=O
N
Cl
HO
O
N
19
1H, CDCl3, 500 MHz
8.19
8.17
7.57
7.55
7.32
7.30
7.29
7.29
7.28
7.27
7.27
7.26
7.26
7.25
7.24
7.22
7.20
7.19
7.16
7.15
7.14
7.14
7.12
6.93
6.91
6.84
6.83
6.81
6.21
6.20
6.19
4.22
4.19
4.19
4.16
3.21
2.30
1.03
2.02
9.06
1.10
11.07
1.02
1.01
2.01
2.01
1.00
3.06
f1 (ppm)

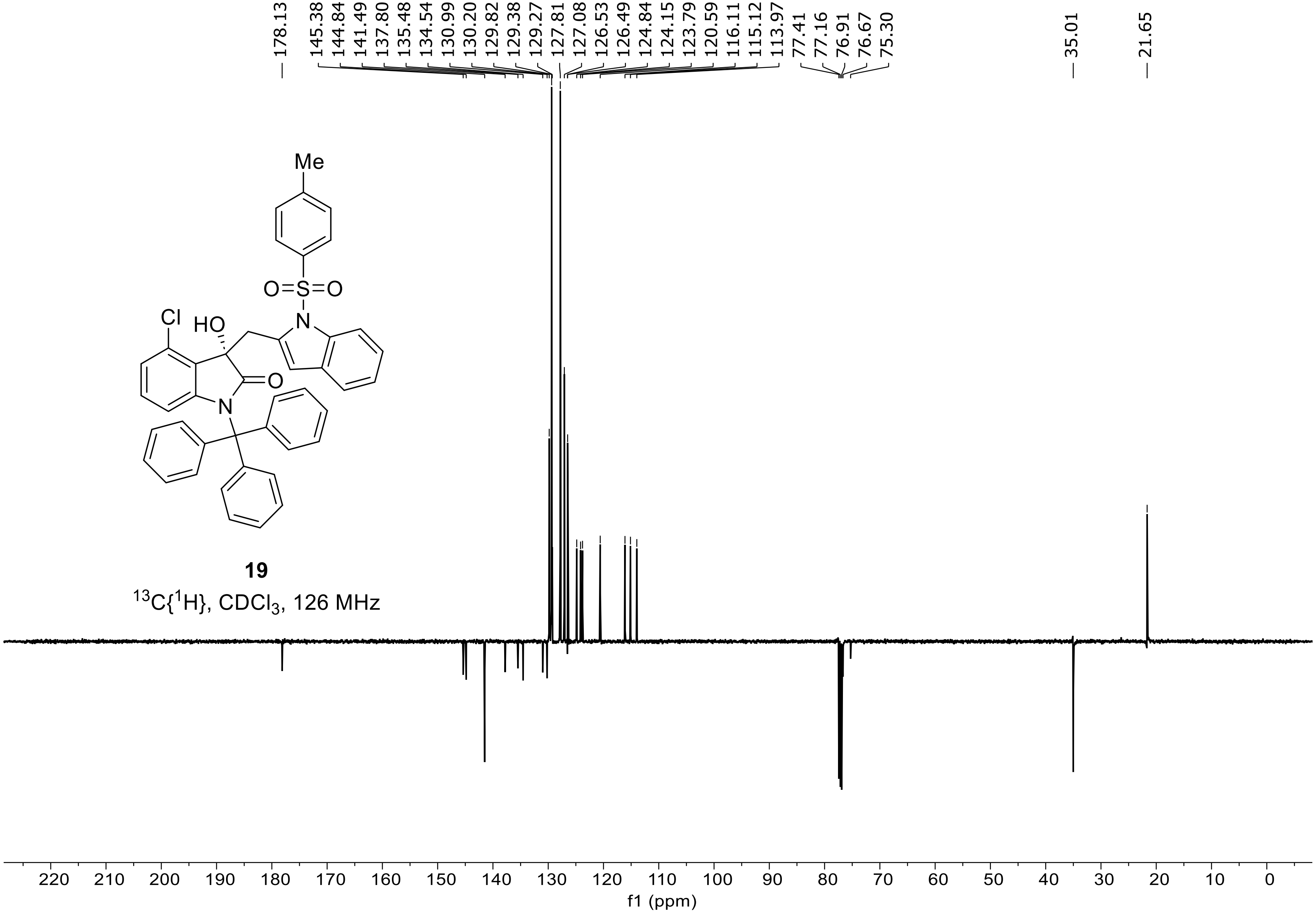
178.13
145.38
144.84
141.49
137.80
135.48
134.54
130.99
130.20
129.82
129.38
129.27
127.81
127.08
126.53
126.49
124.84
124.15
123.79
120.59
116.11
115.12
113.97
77.41
77.16
76.91
76.67
75.30
35.01
21.65
Me
O=S=O
N
Cl
HO
O
N
19
$^{13}C\{^1H\}$, CDCl$_3$, 126 MHz
220 210 200 190 180 170 160 150 140 130 120 110 100 90 80 70 60 50 40 30 20 10 0
f1 (ppm)

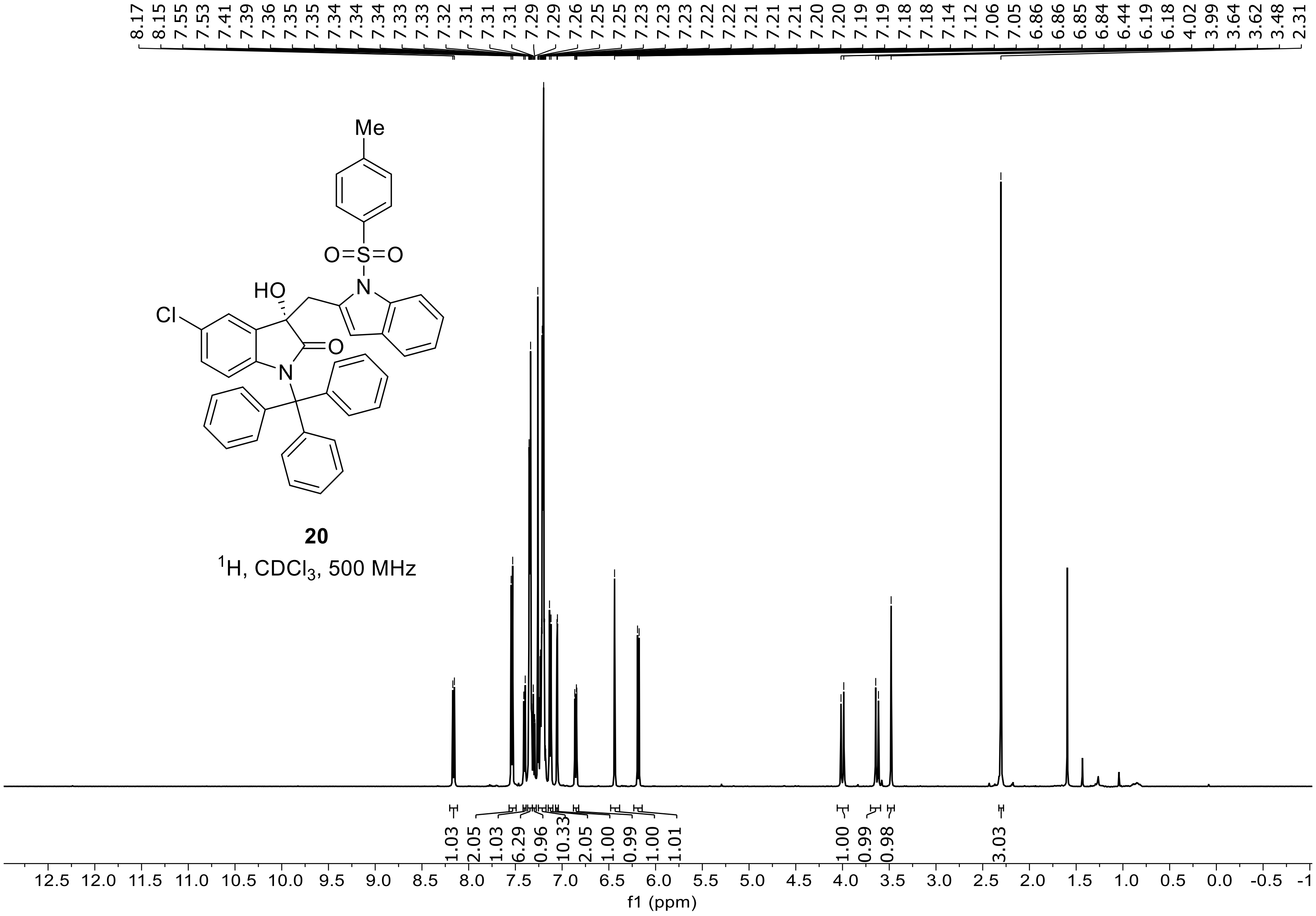
8.17 8.15 7.55 7.53 7.41 7.39 7.36 7.35 7.35 7.34 7.34 7.34 7.33 7.33 7.32 7.31 7.31 7.31 7.29 7.29 7.26 7.25 7.25 7.23 7.23 7.23 7.22 7.22 7.21 7.21 7.21 7.20 7.20 7.19 7.19 7.18 7.18 7.14 7.12 7.06 7.05 6.86 6.86 6.85 6.84 6.44 6.19 6.18 4.02 3.99 3.64 3.62 3.48 2.31
Me
O=S=O
N
HO
Cl
O
N
20
^{1}H, CDCl$_3$, 500 MHz
1.03 2.05 1.03 6.29 0.96 10.33 2.05 1.00 0.99 1.00 1.01 1.00 0.99 0.98 3.03
12.5 12.0 11.5 11.0 10.5 10.0 9.5 9.0 8.5 8.0 7.5 7.0 6.5 6.0 5.5 5.0 4.5 4.0 3.5 3.0 2.5 2.0 1.5 1.0 0.5 0.0 -0.5 -1
f1 (ppm)

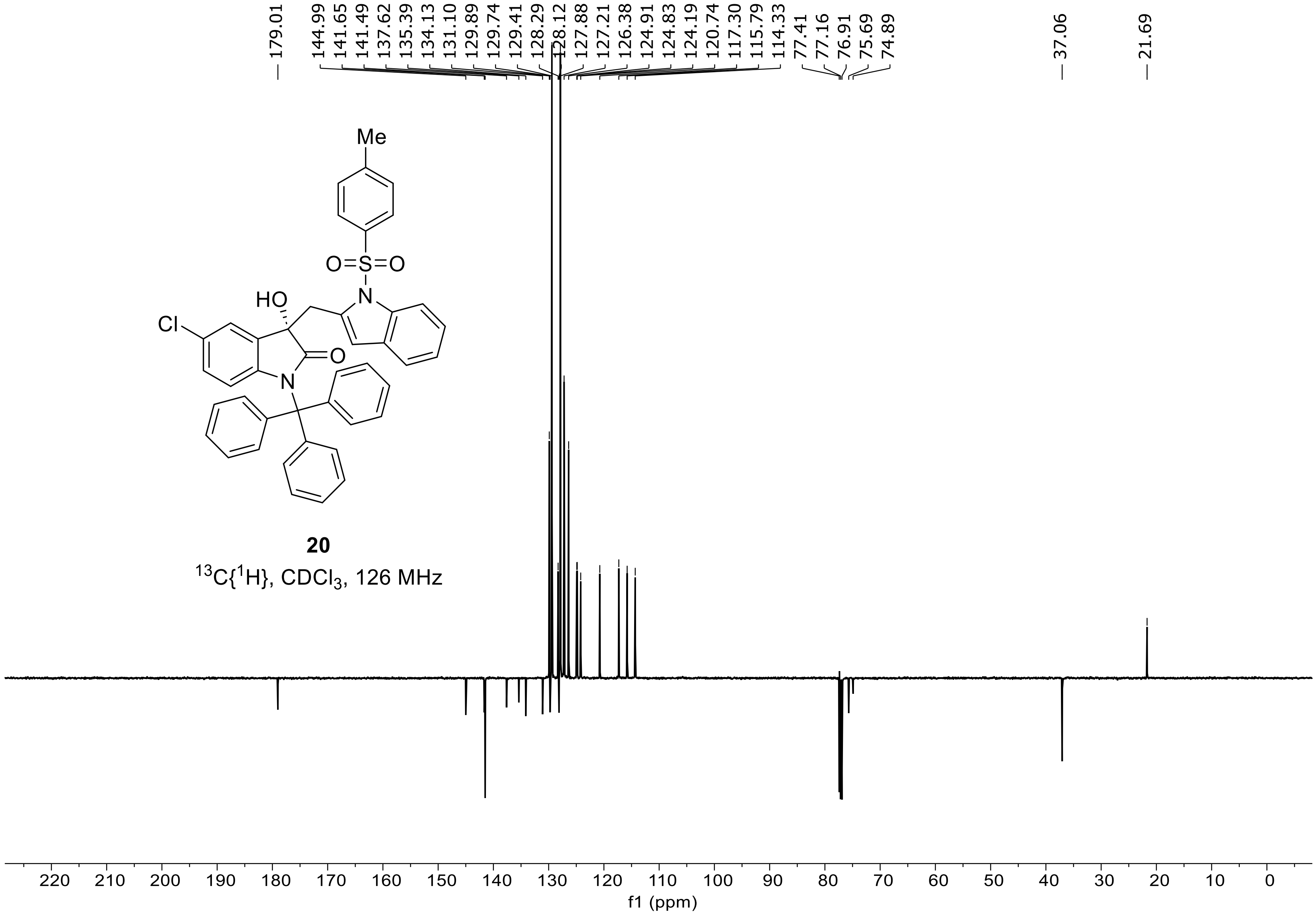
179.01
144.99
141.65
141.49
137.62
135.39
134.13
131.10
129.89
129.74
129.41
128.29
128.12
127.88
127.21
126.38
124.91
124.83
124.19
120.74
117.30
115.79
114.33
77.41
77.16
76.91
75.69
74.89
37.06
21.69
Me
O=S=O
N
HO
Cl
O
N
20
13C{1H}, CDCl3, 126 MHz
220 210 200 190 180 170 160 150 140 130 120 110 100 90 80 70 60 50 40 30 20 10 0
f1 (ppm)

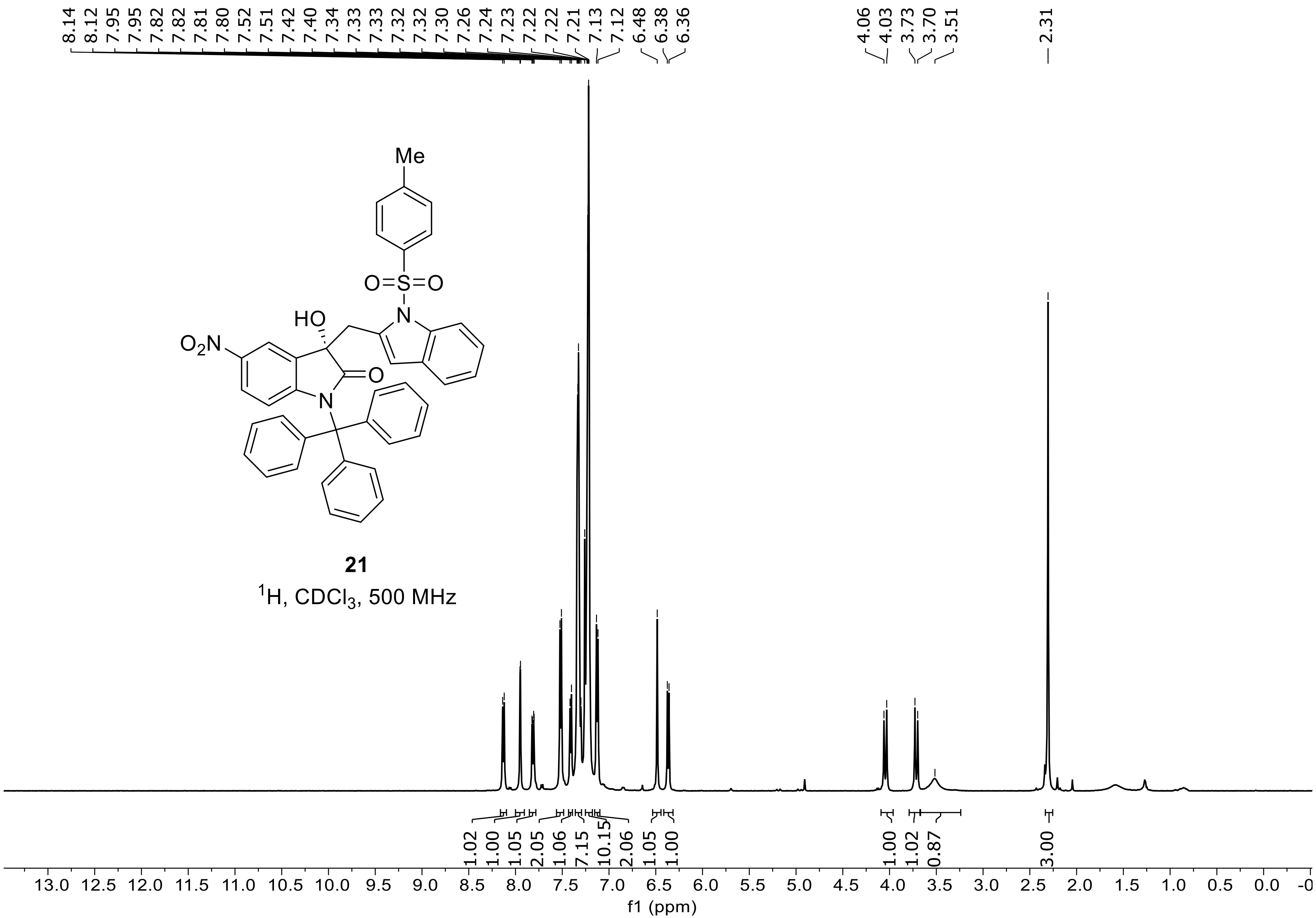

21

^1H, $CDCl_3$, 500 MHz

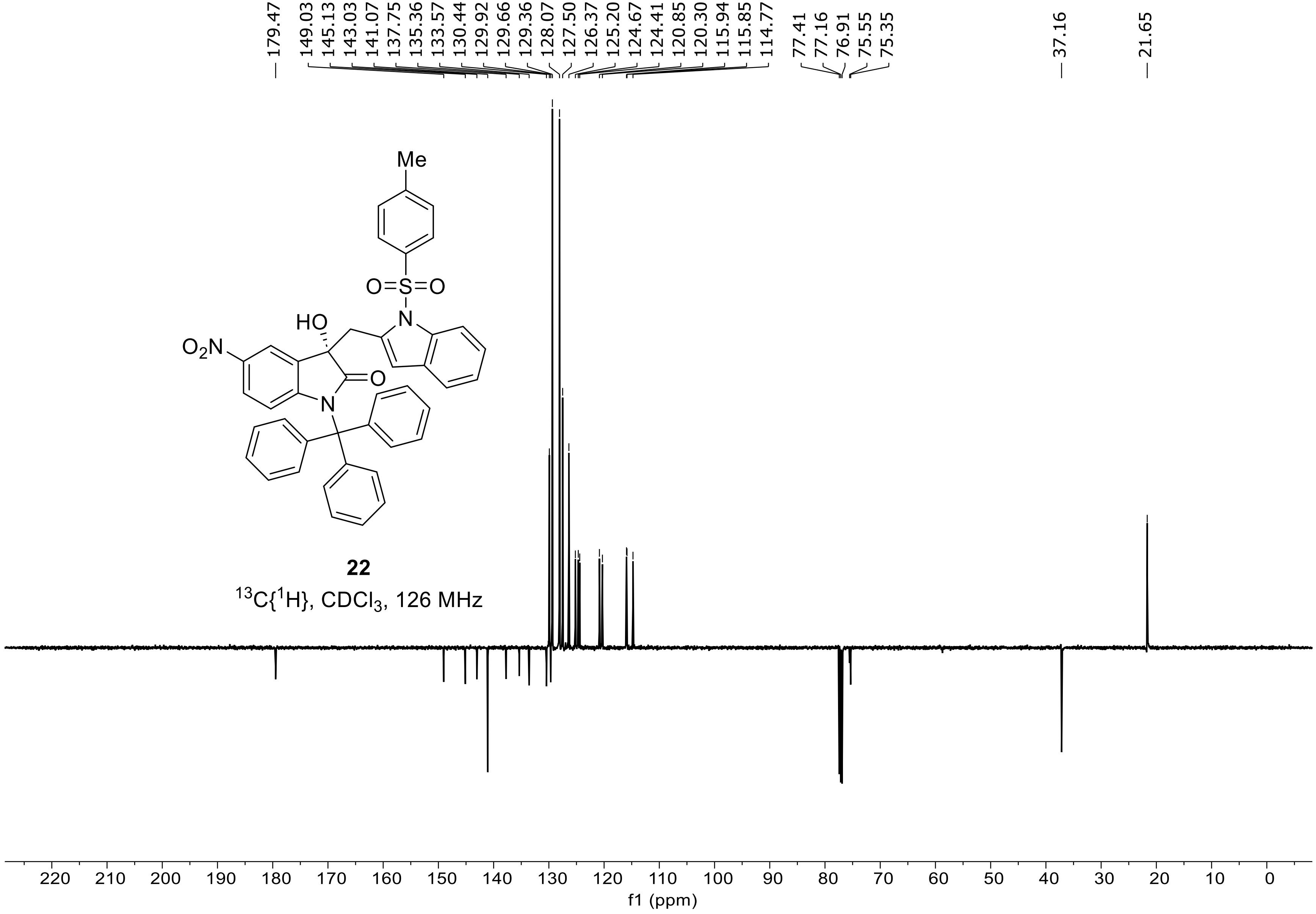
179.47
149.03
145.13
143.03
141.07
137.75
135.36
133.57
130.44
129.92
129.66
129.36
128.07
127.50
126.37
125.20
124.67
124.41
120.85
120.30
115.94
115.85
114.77
77.41
77.16
76.91
75.55
75.35
37.16
21.65
Me
O=S=O
N
HO
O_2N
O
N
22
$^{13}C\{^1H\}$, $CDCl_3$, 126 MHz
220 210 200 190 180 170 160 150 140 130 120 110 100 90 80 70 60 50 40 30 20 10 0
f1 (ppm)

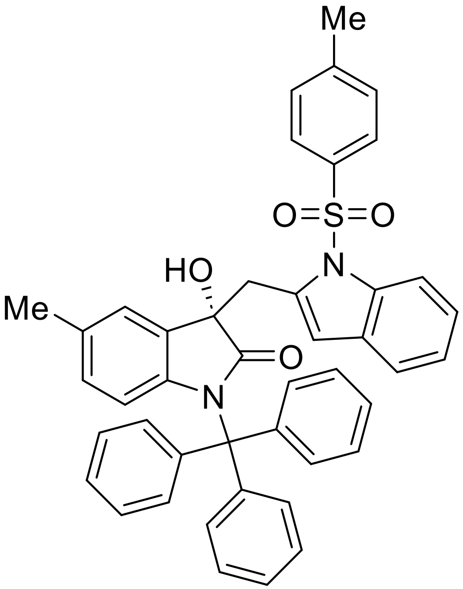

22

^{1}H, $CDCl_3$, 500 MHz

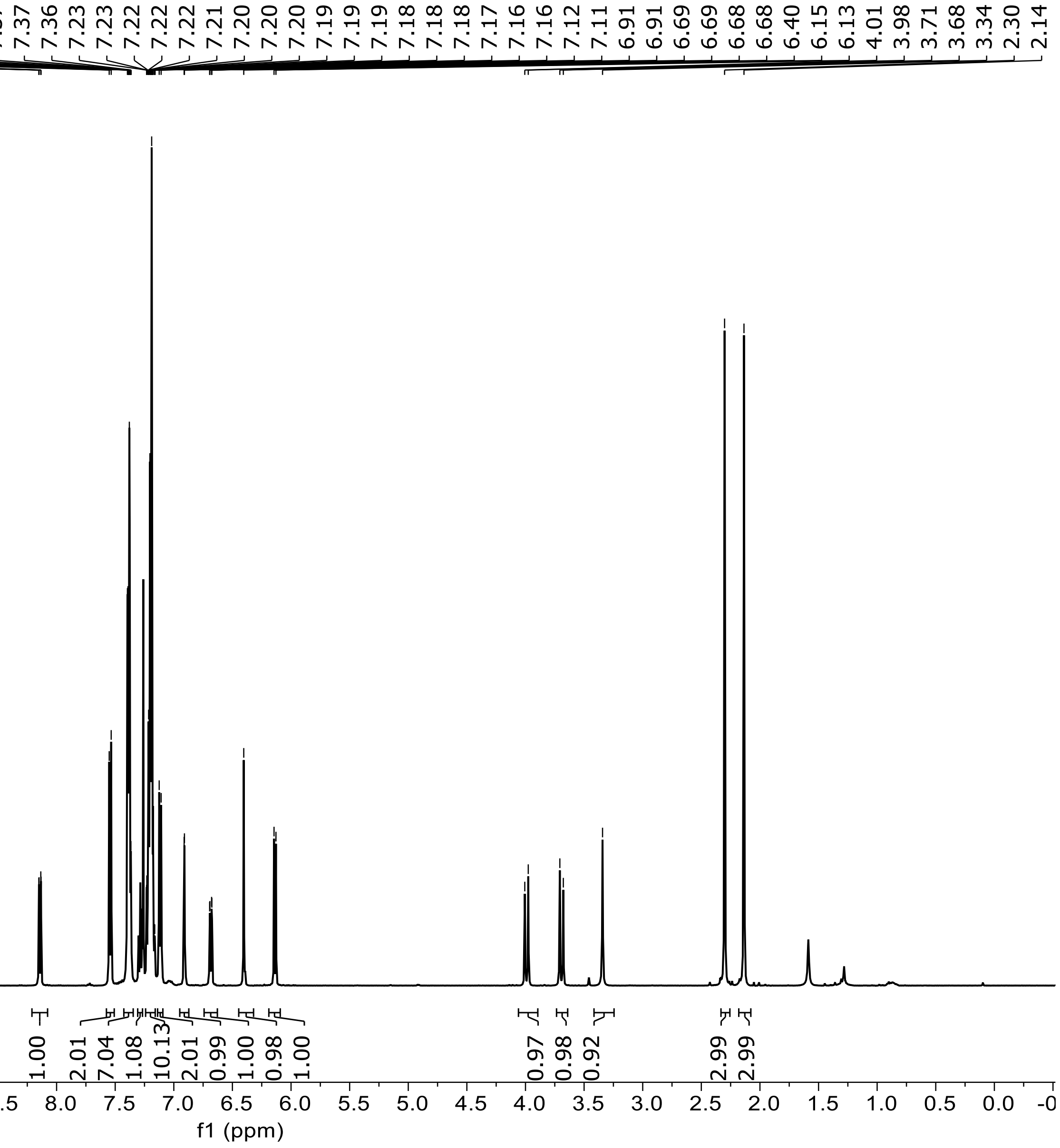

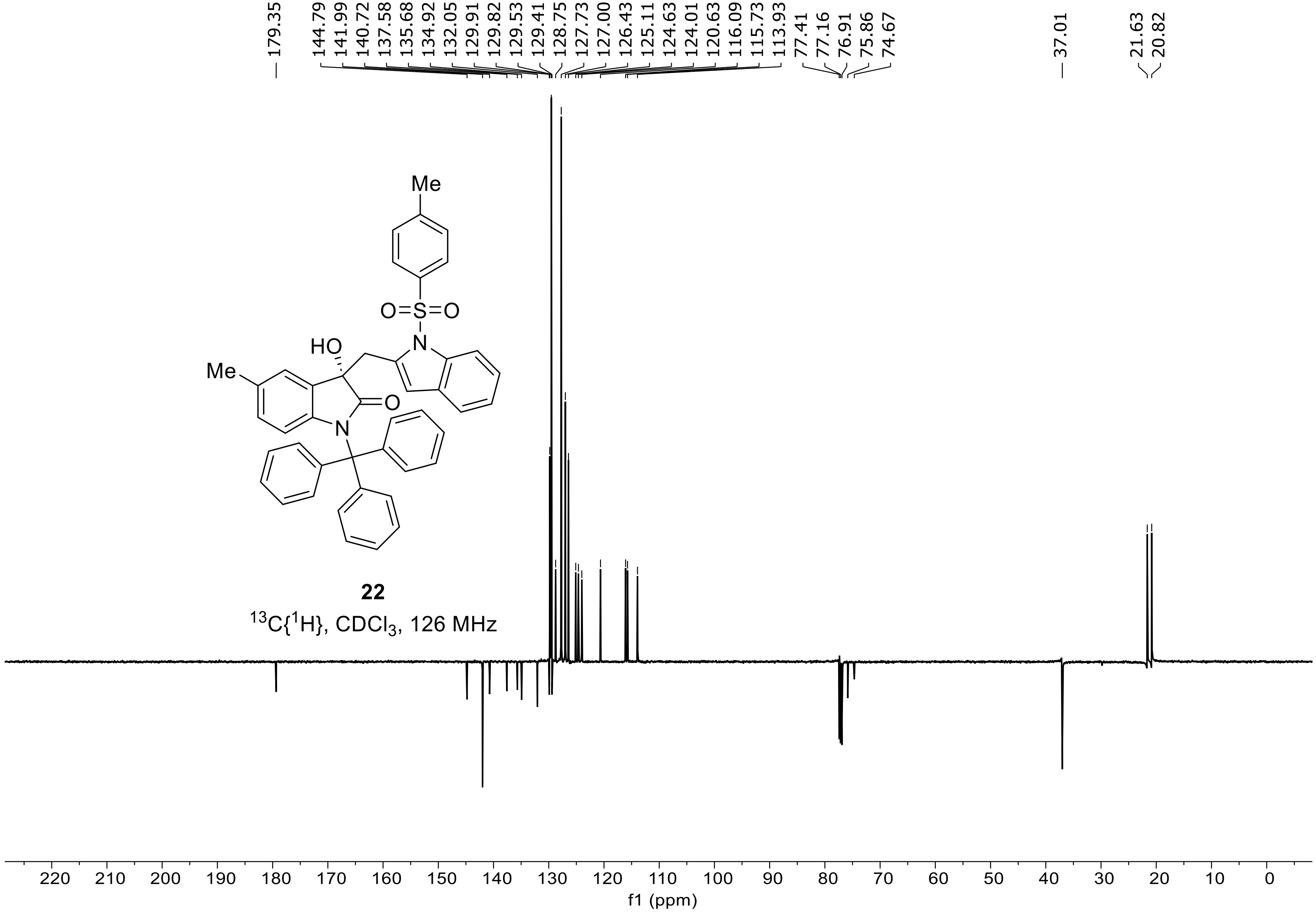

22

$^{13}C\{^{1}H\}$, $CDCl_3$, 126 MHz

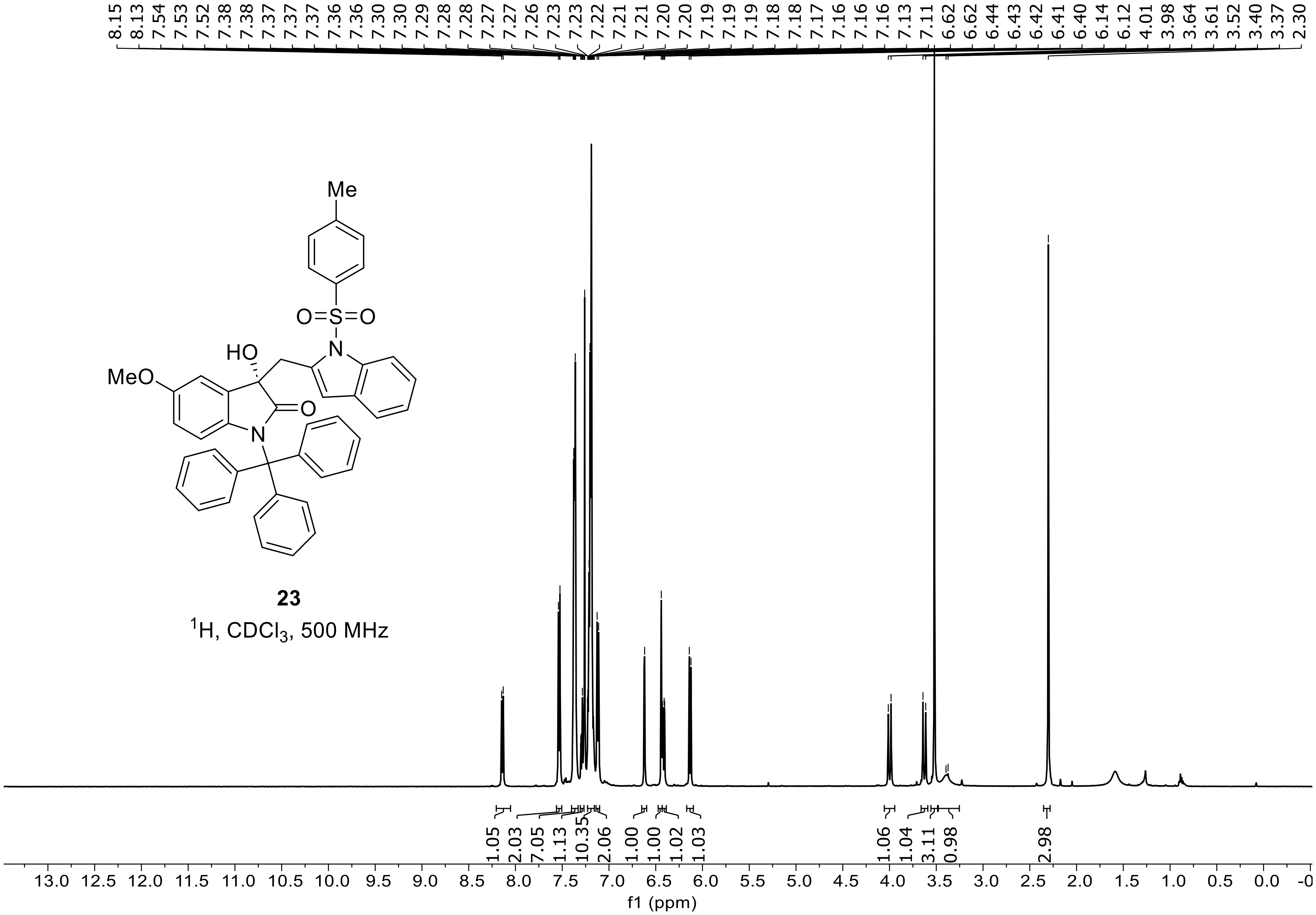
8.15 8.13 7.54 7.53 7.52 7.38 7.38 7.37 7.37 7.37 7.36 7.36 7.30 7.30 7.29 7.28 7.28 7.27 7.27 7.26 7.23 7.23 7.22 7.21 7.21 7.20 7.20 7.19 7.19 7.19 7.18 7.18 7.17 7.16 7.16 7.16 7.13 7.11 6.62 6.62 6.44 6.43 6.42 6.41 6.40 6.14 6.12 4.01 3.98 3.64 3.61 3.52 3.40 3.37 2.30
Me
O=S=O
N
HO
MeO
O
N
23
^{1}H, CDCl$_3$, 500 MHz
1.05 2.03 7.05 1.13 10.35 2.06 1.00 1.00 1.02 1.03 1.06 1.04 3.11 0.98 2.98
13.0 12.5 12.0 11.5 11.0 10.5 10.0 9.5 9.0 8.5 8.0 7.5 7.0 6.5 6.0 5.5 5.0 4.5 4.0 3.5 3.0 2.5 2.0 1.5 1.0 0.5 0.0 -0
f1 (ppm)

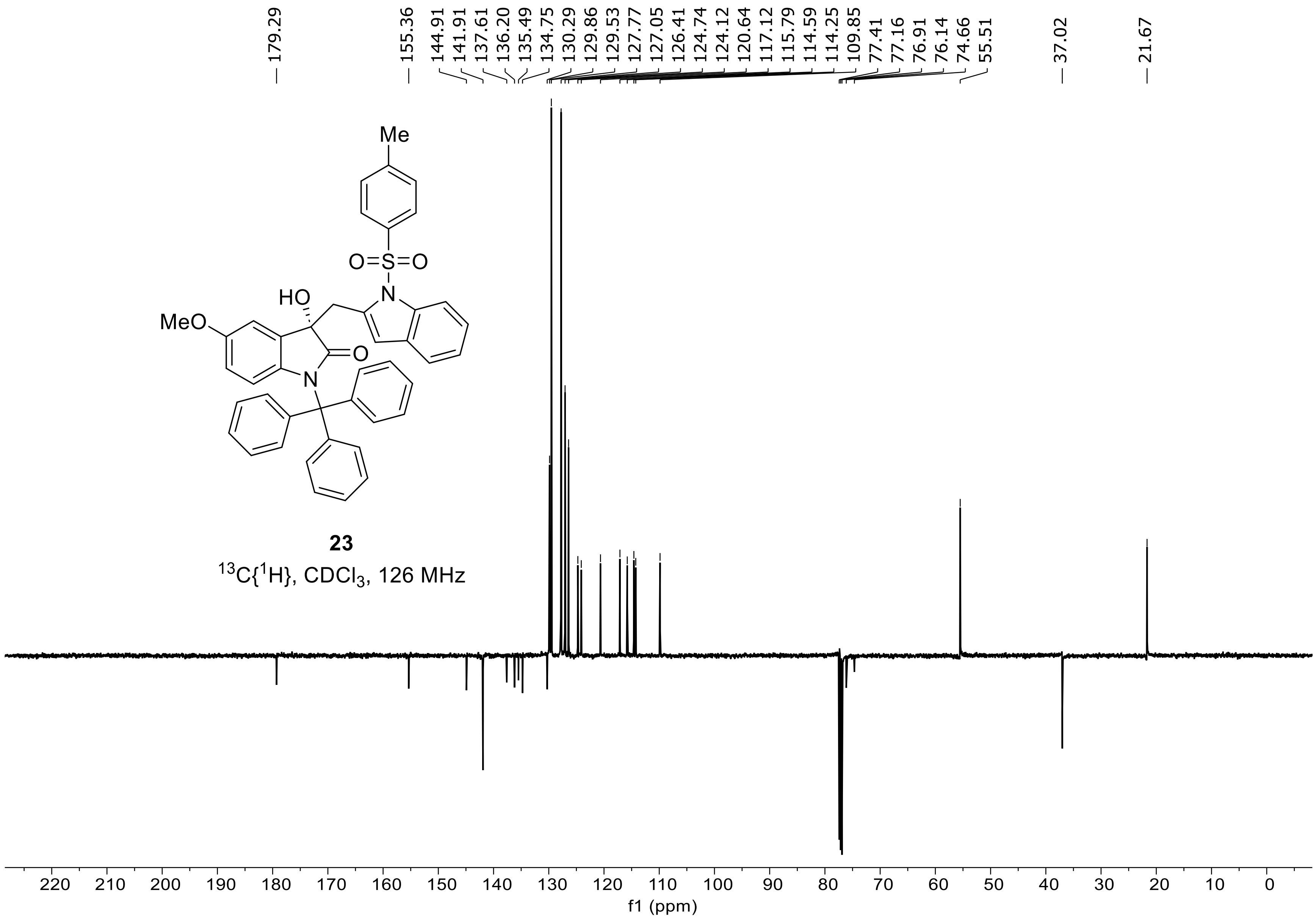
179.29
155.36
144.91
141.91
137.61
136.20
135.49
134.75
130.29
129.86
129.53
127.77
127.05
126.41
124.74
124.12
120.64
117.12
115.79
114.59
114.25
109.85
77.41
77.16
76.91
76.14
74.66
55.51
37.02
21.67
Me
O=S=O
N
HO
MeO
O
N
23
$^{13}C\{^1H\}$, $CDCl_3$, 126 MHz
220 210 200 190 180 170 160 150 140 130 120 110 100 90 80 70 60 50 40 30 20 10 0
f1 (ppm)

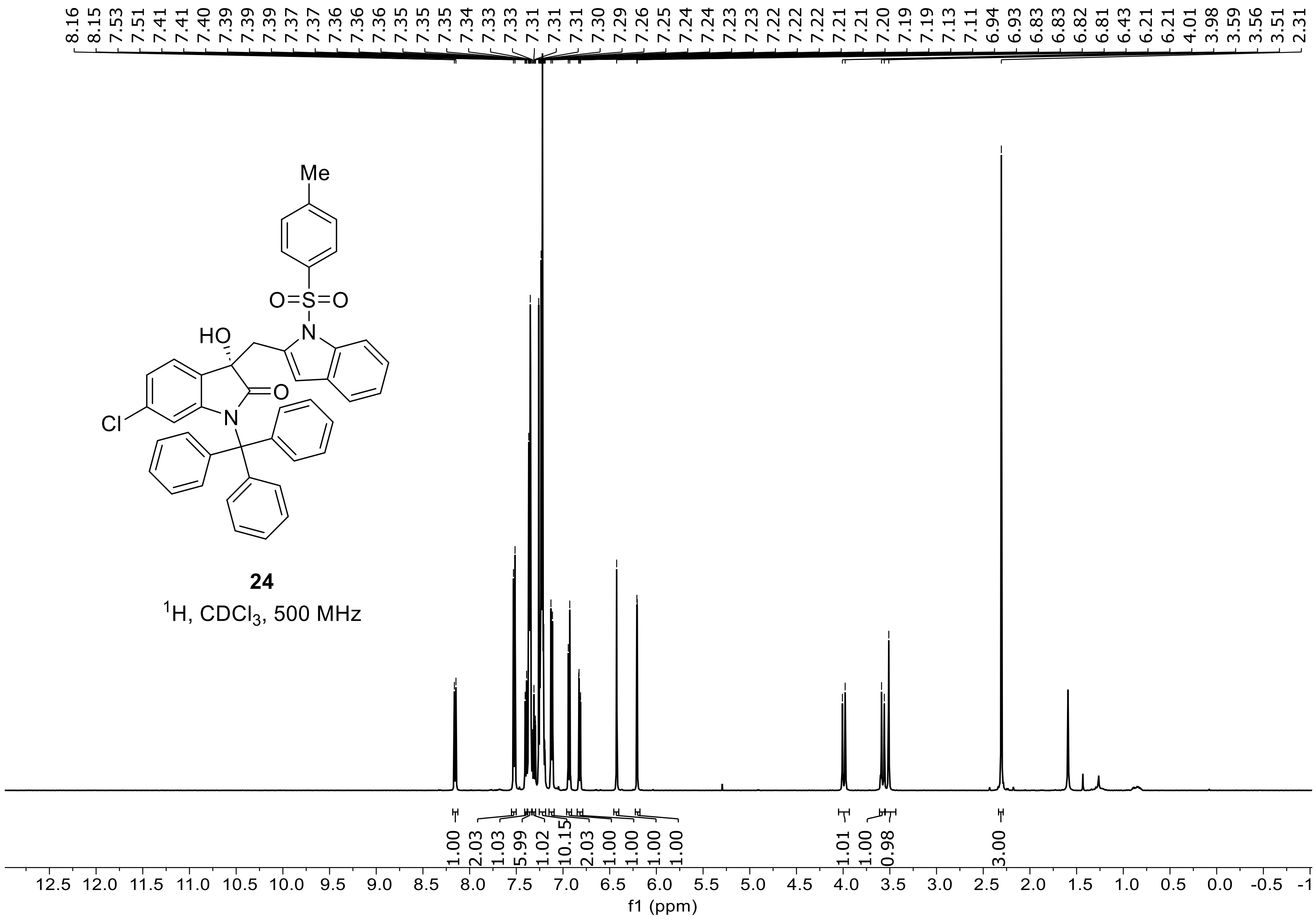
8.16 8.15 7.53 7.51 7.41 7.41 7.40 7.39 7.39 7.39 7.37 7.37 7.36 7.36 7.36 7.35 7.35 7.35 7.34 7.33 7.33 7.31 7.31 7.31 7.30 7.29 7.26 7.25 7.24 7.24 7.23 7.23 7.22 7.22 7.22 7.21 7.21 7.20 7.19 7.19 7.13 7.11 6.94 6.93 6.83 6.83 6.82 6.81 6.43 6.21 6.21 4.01 3.98 3.59 3.56 3.51 2.31
Me
O=S=O
N
HO
O
Cl
N
24
^{1}H, $CDCl_3$, 500 MHz
1.00 2.03 1.03 5.99 1.02 10.15 2.03 1.00 1.00 1.00 1.00
1.01 1.00 0.98
3.00
12.5 12.0 11.5 11.0 10.5 10.0 9.5 9.0 8.5 8.0 7.5 7.0 6.5 6.0 5.5 5.0 4.5 4.0 3.5 3.0 2.5 2.0 1.5 1.0 0.5 0.0 -0.5 -1
f1 (ppm)

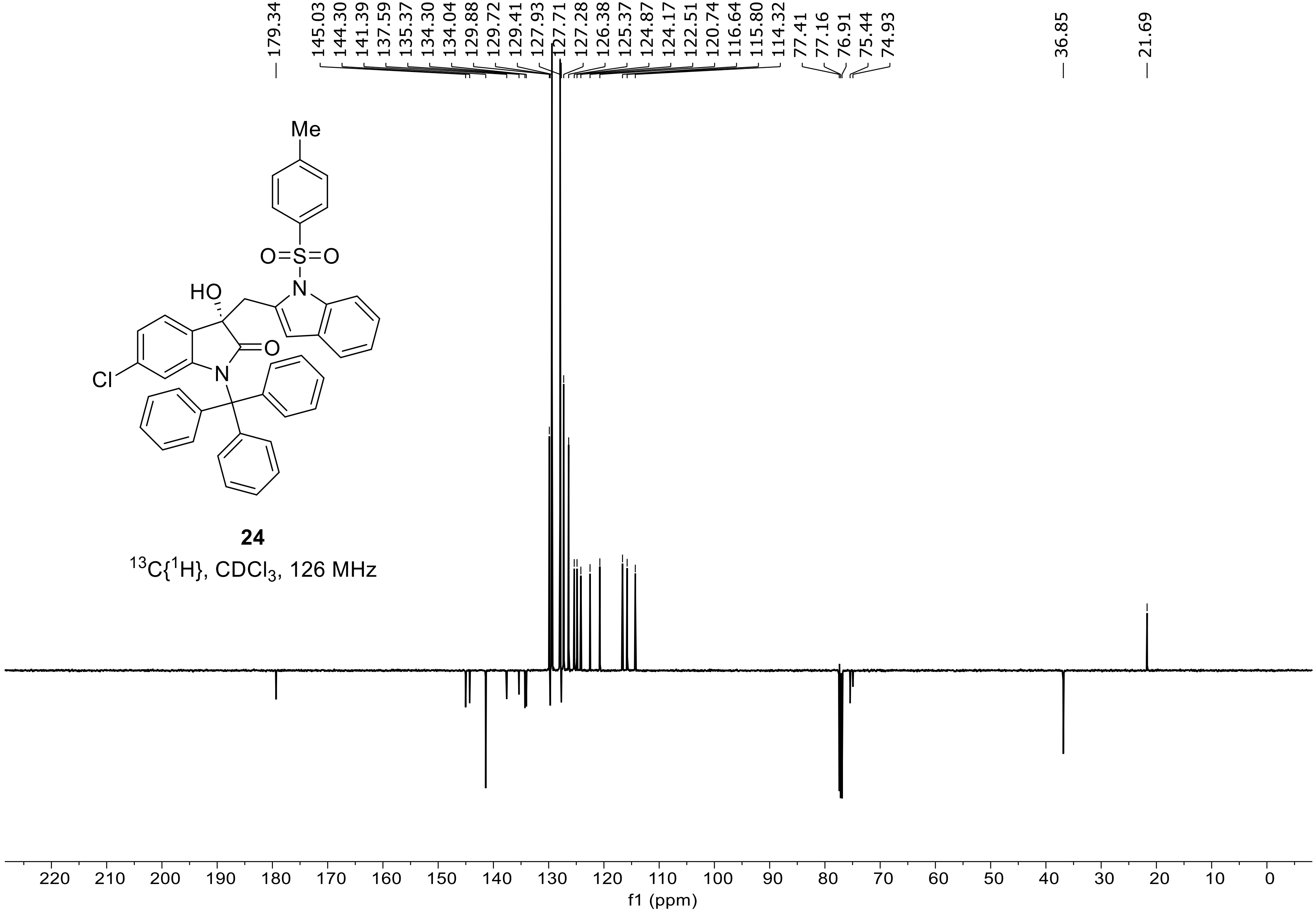
179.34
145.03
144.30
141.39
137.59
135.37
134.30
134.04
129.88
129.72
129.41
127.93
127.71
127.28
126.38
125.37
124.87
124.17
122.51
120.74
116.64
115.80
114.32
77.41
77.16
76.91
75.44
74.93
36.85
21.69
Me
O=S=O
N
HO
O
Cl
N
24
$^{13}C\{^{1}H\}$, $CDCl_3$, 126 MHz
220 210 200 190 180 170 160 150 140 130 120 110 100 90 80 70 60 50 40 30 20 10 0
f1 (ppm)

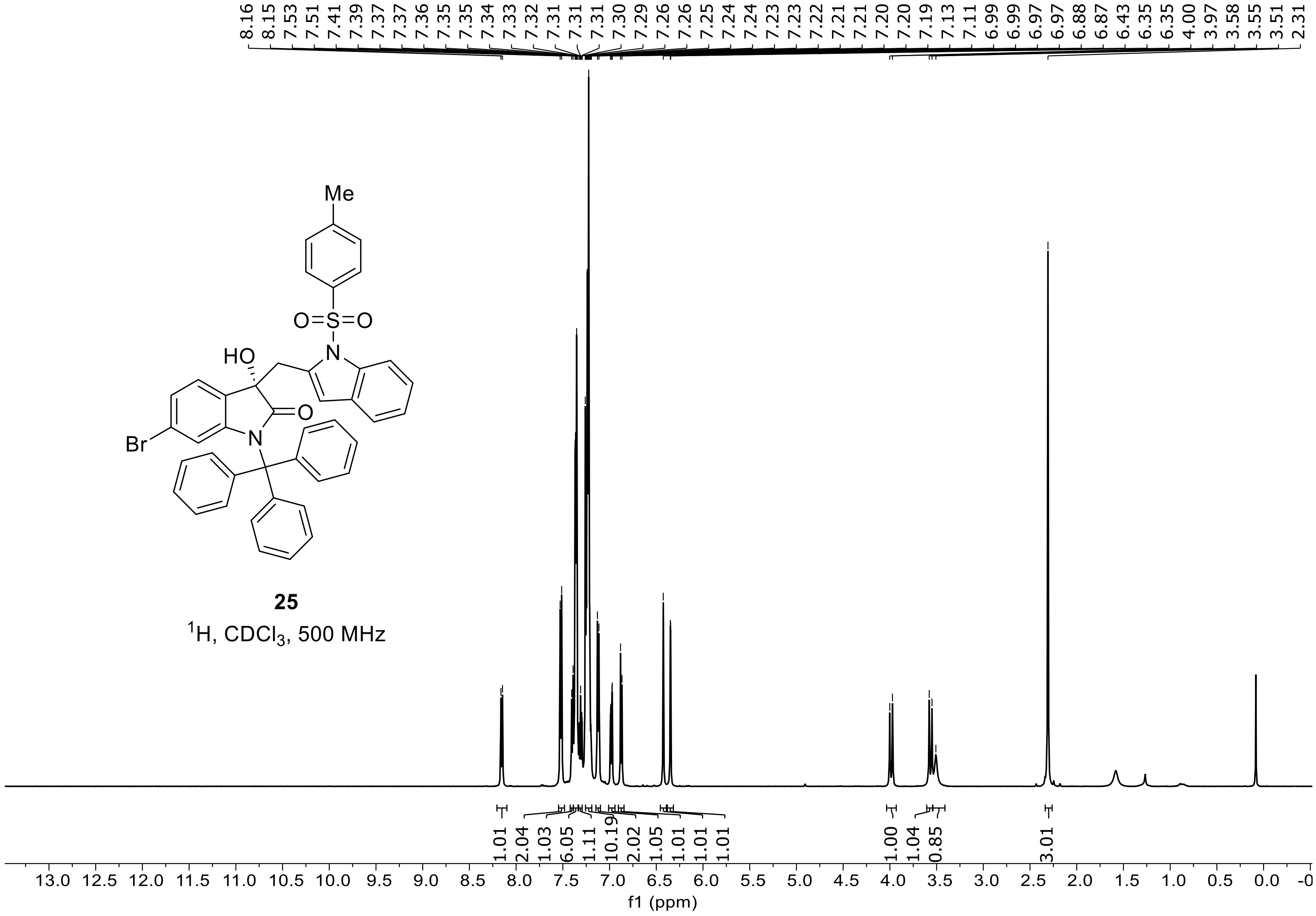

25

^{1}H, $CDCl_3$, 500 MHz

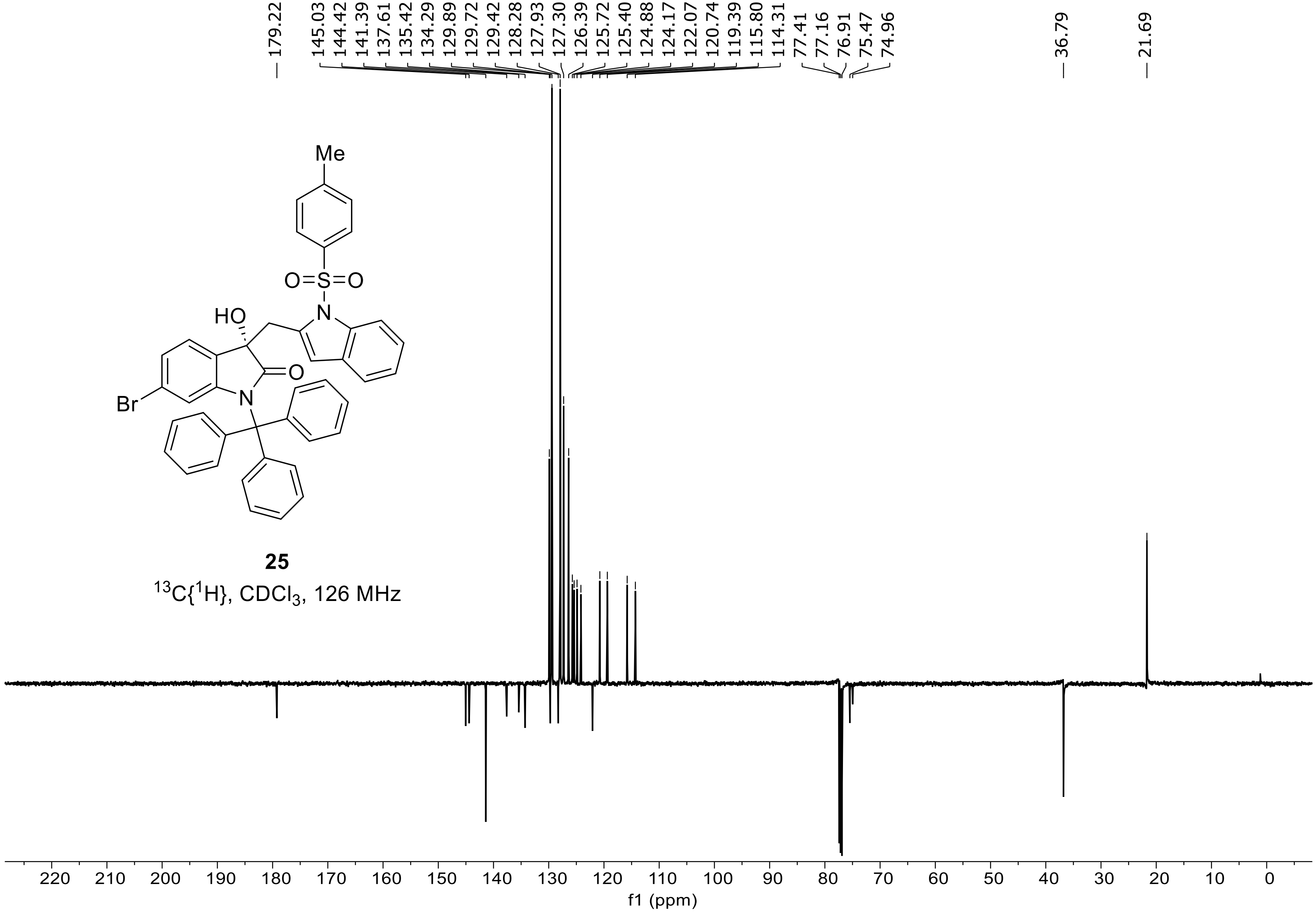
179.22
145.03
144.42
141.39
137.61
135.42
134.29
129.89
129.72
129.42
128.28
127.93
127.30
126.39
125.72
125.40
124.88
124.17
122.07
120.74
119.39
115.80
114.31
77.41
77.16
76.91
75.47
74.96
36.79
21.69
Me
O=S=O
N
HO
O
Br
N
25
$^{13}C\{^1H\}$, $CDCl_3$, 126 MHz
220 210 200 190 180 170 160 150 140 130 120 110 100 90 80 70 60 50 40 30 20 10 0
f1 (ppm)

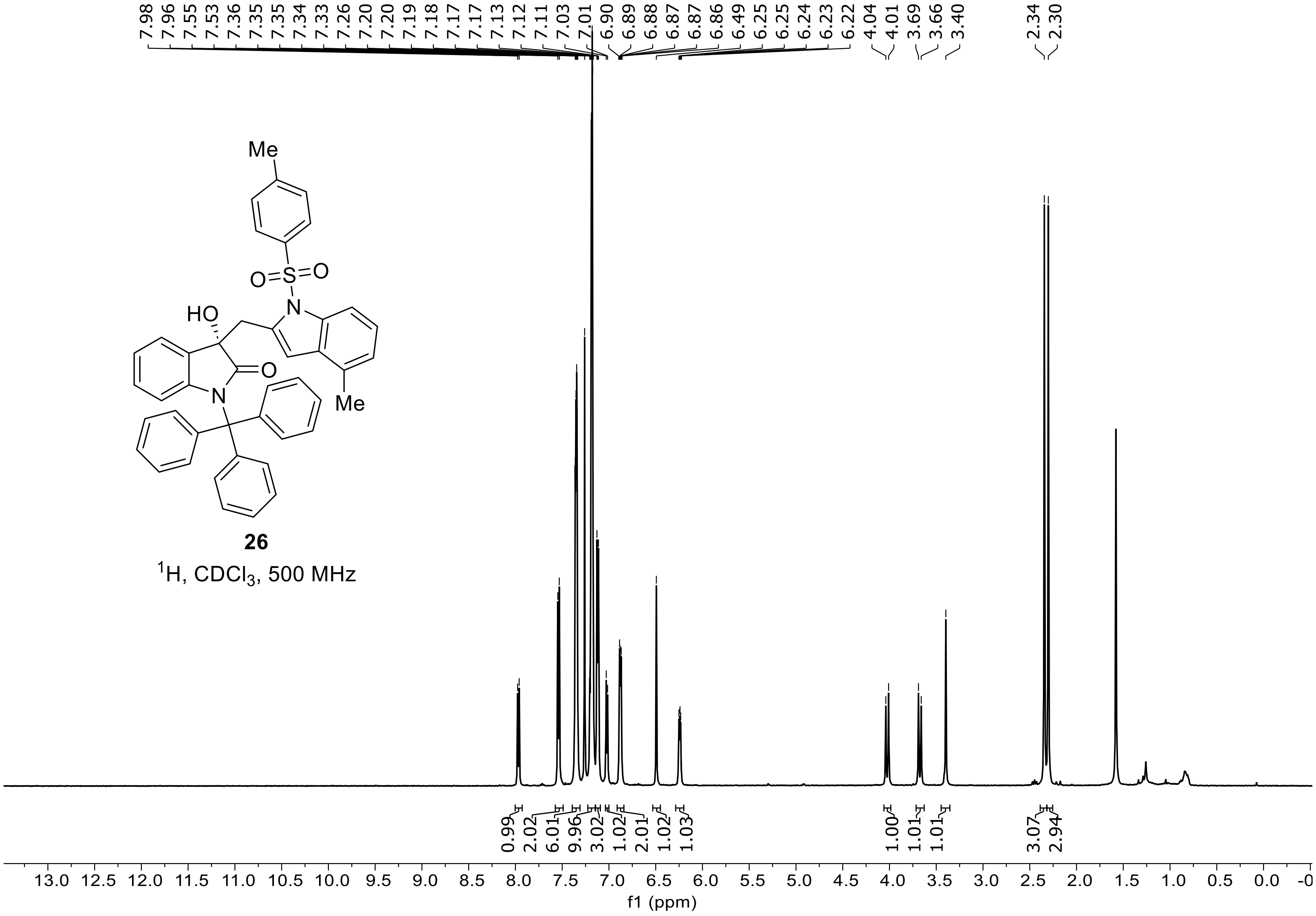
7.98 7.96 7.55 7.53 7.36 7.35 7.35 7.34 7.33 7.26 7.20 7.20 7.19 7.18 7.17 7.17 7.13 7.12 7.11 7.03 7.01 6.90 6.89 6.88 6.87 6.87 6.86 6.49 6.25 6.25 6.24 6.23 6.22 4.04 4.01 3.69 3.66 3.40 2.34 2.30
Me
HO
O=S=O
N
O
N
Me
26
^{1}H, CDCl$_3$, 500 MHz
0.99 2.02 6.01 9.96 3.02 1.02 2.01 1.02 1.03 1.00 1.01 1.01 3.07 2.94
13.0 12.5 12.0 11.5 11.0 10.5 10.0 9.5 9.0 8.5 8.0 7.5 7.0 6.5 6.0 5.5 5.0 4.5 4.0 3.5 3.0 2.5 2.0 1.5 1.0 0.5 0.0 -0
f1 (ppm)

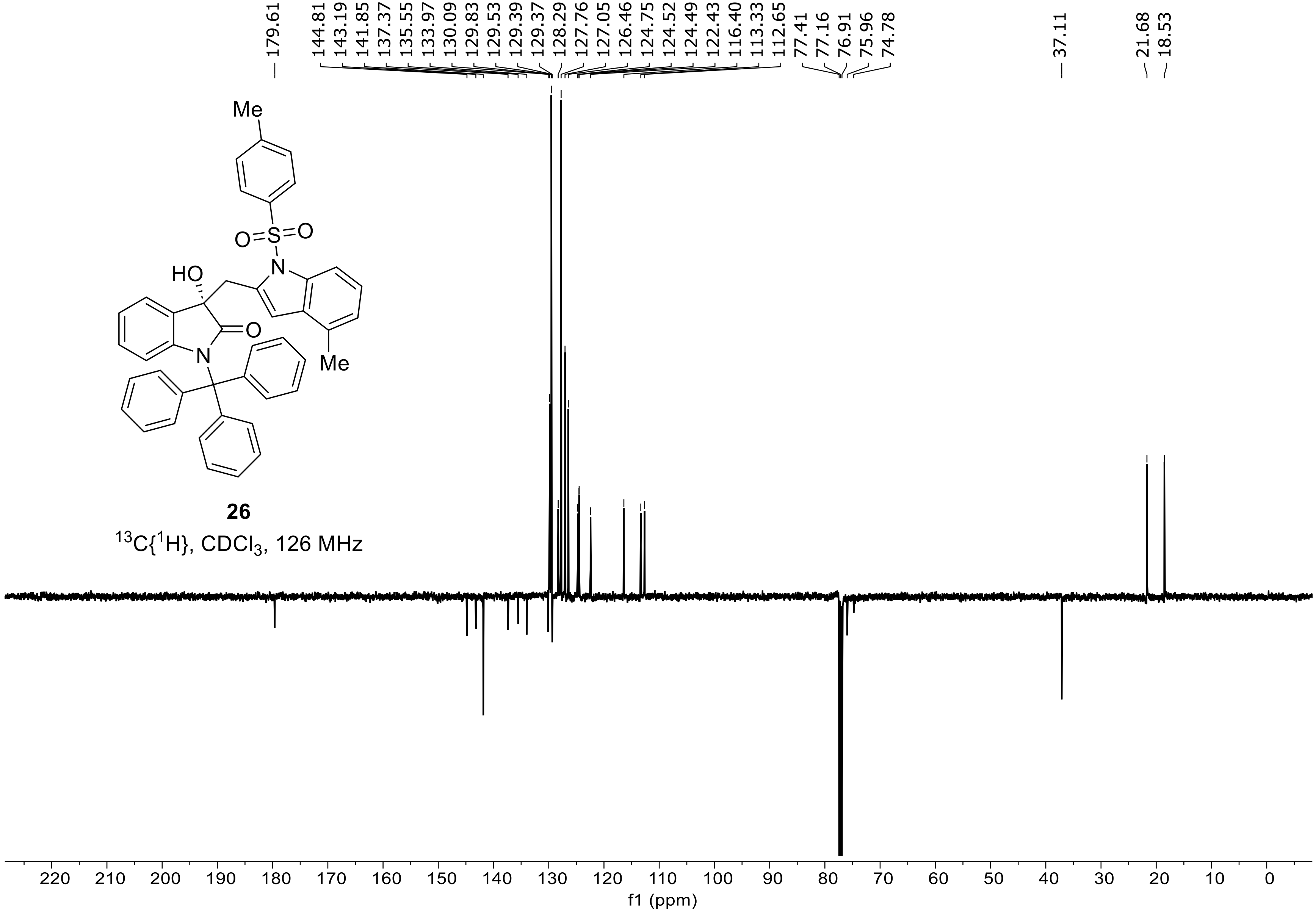
179.61
144.81
143.19
141.85
137.37
135.55
133.97
130.09
129.83
129.53
129.39
129.37
128.29
127.76
127.05
126.46
124.75
124.52
124.49
122.43
116.40
113.33
112.65
77.41
77.16
76.91
75.96
74.78
37.11
21.68
18.53
Me
O=S=O
N
HO
O
N
Me
26
$^{13}C\{^1H\}$, $CDCl_3$, 126 MHz
220 210 200 190 180 170 160 150 140 130 120 110 100 90 80 70 60 50 40 30 20 10 0
f1 (ppm)

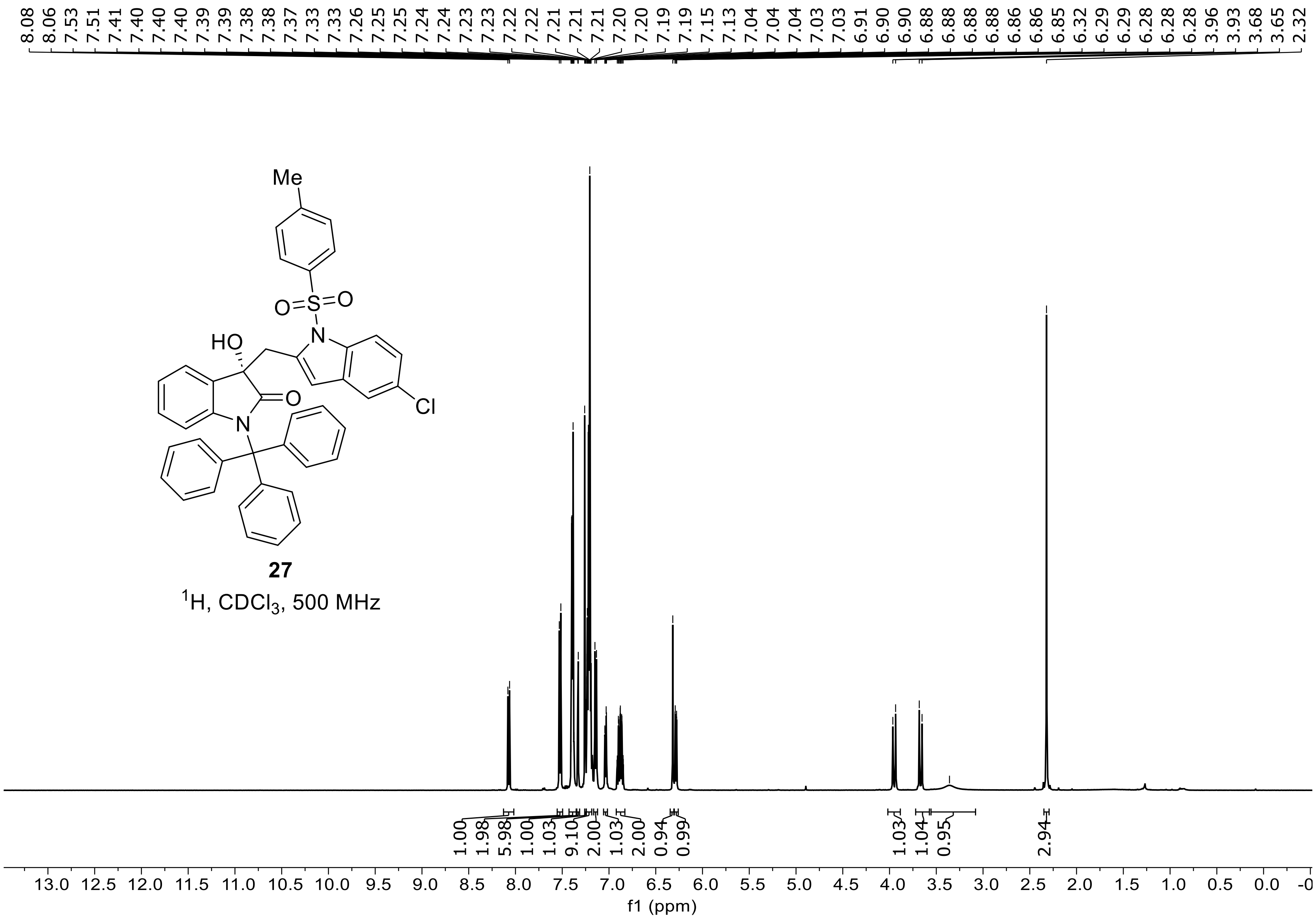
8.08 8.06 7.53 7.51 7.41 7.40 7.40 7.39 7.39 7.38 7.38 7.37 7.33 7.33 7.26 7.25 7.25 7.24 7.24 7.23 7.23 7.22 7.22 7.21 7.21 7.21 7.20 7.20 7.19 7.19 7.15 7.13 7.04 7.04 7.04 7.03 7.03 6.91 6.90 6.90 6.88 6.88 6.88 6.86 6.86 6.85 6.32 6.29 6.29 6.28 6.28 6.28 3.96 3.93 3.68 3.65 2.32
Me
O=S=O
N
HO
O
N
Cl
27
^{1}H, CDCl$_3$, 500 MHz
1.00 1.98 5.98 1.00 1.03 9.10 2.00 1.03 2.00 0.94 0.99 1.03 1.04 0.95 2.94
13.0 12.5 12.0 11.5 11.0 10.5 10.0 9.5 9.0 8.5 8.0 7.5 7.0 6.5 6.0 5.5 5.0 4.5 4.0 3.5 3.0 2.5 2.0 1.5 1.0 0.5 0.0 -0
f1 (ppm)

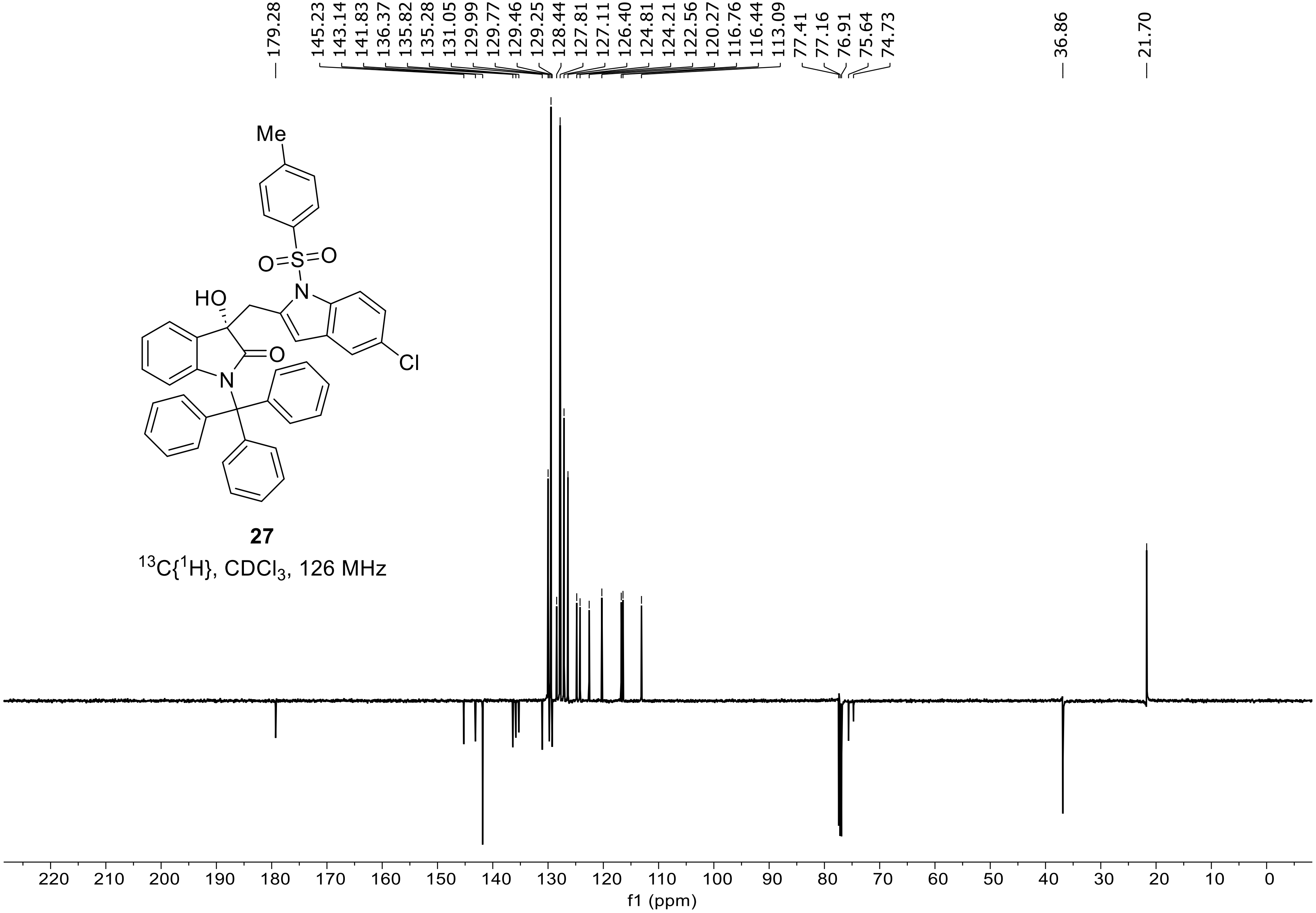
179.28
145.23
143.14
141.83
136.37
135.82
135.28
131.05
129.99
129.77
129.46
129.25
128.44
127.81
127.11
126.40
124.81
124.21
122.56
120.27
116.76
116.44
113.09
77.41
77.16
76.91
75.64
74.73
36.86
21.70
Me
O=S=O
N
HO
O
N
Cl
27
13C{1H}, CDCl3, 126 MHz
220 210 200 190 180 170 160 150 140 130 120 110 100 90 80 70 60 50 40 30 20 10 0
f1 (ppm)

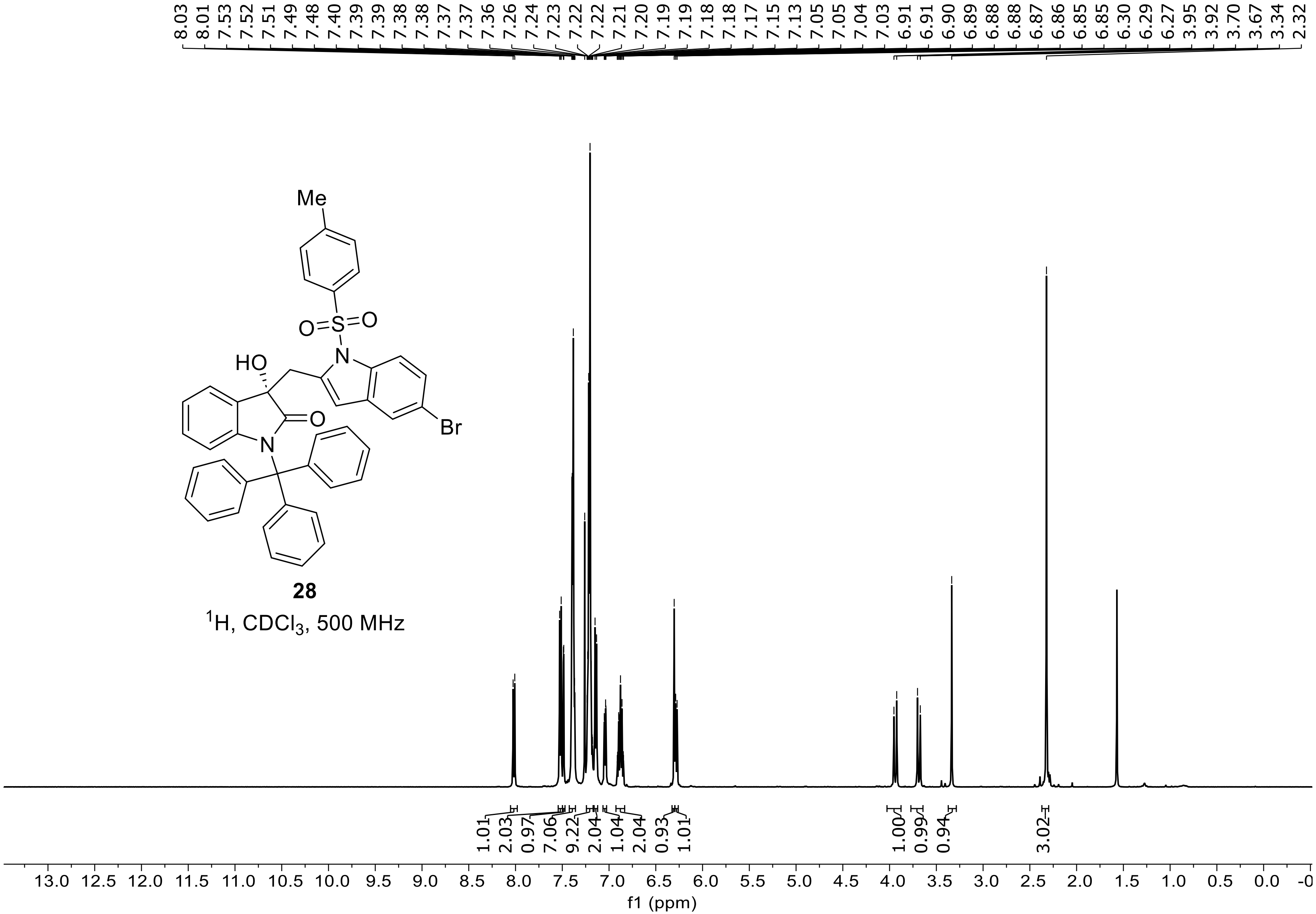
8.03 8.01 7.53 7.52 7.51 7.49 7.48 7.40 7.39 7.39 7.38 7.38 7.37 7.37 7.36 7.26 7.24 7.23 7.22 7.22 7.21 7.20 7.19 7.19 7.18 7.18 7.17 7.15 7.13 7.05 7.05 7.04 7.03 6.91 6.91 6.90 6.89 6.88 6.88 6.87 6.86 6.85 6.85 6.30 6.29 6.27 3.95 3.92 3.70 3.67 3.34 2.32
Me
O=S=O
N
HO
O
N
Br
28
1H, CDCl3, 500 MHz
1.01 2.03 0.97 7.06 9.22 2.04 1.04 2.04 0.93 1.01
1.00 0.99 0.94
3.02
13.0 12.5 12.0 11.5 11.0 10.5 10.0 9.5 9.0 8.5 8.0 7.5 7.0 6.5 6.0 5.5 5.0 4.5 4.0 3.5 3.0 2.5 2.0 1.5 1.0 0.5 0.0 -0
f1 (ppm)

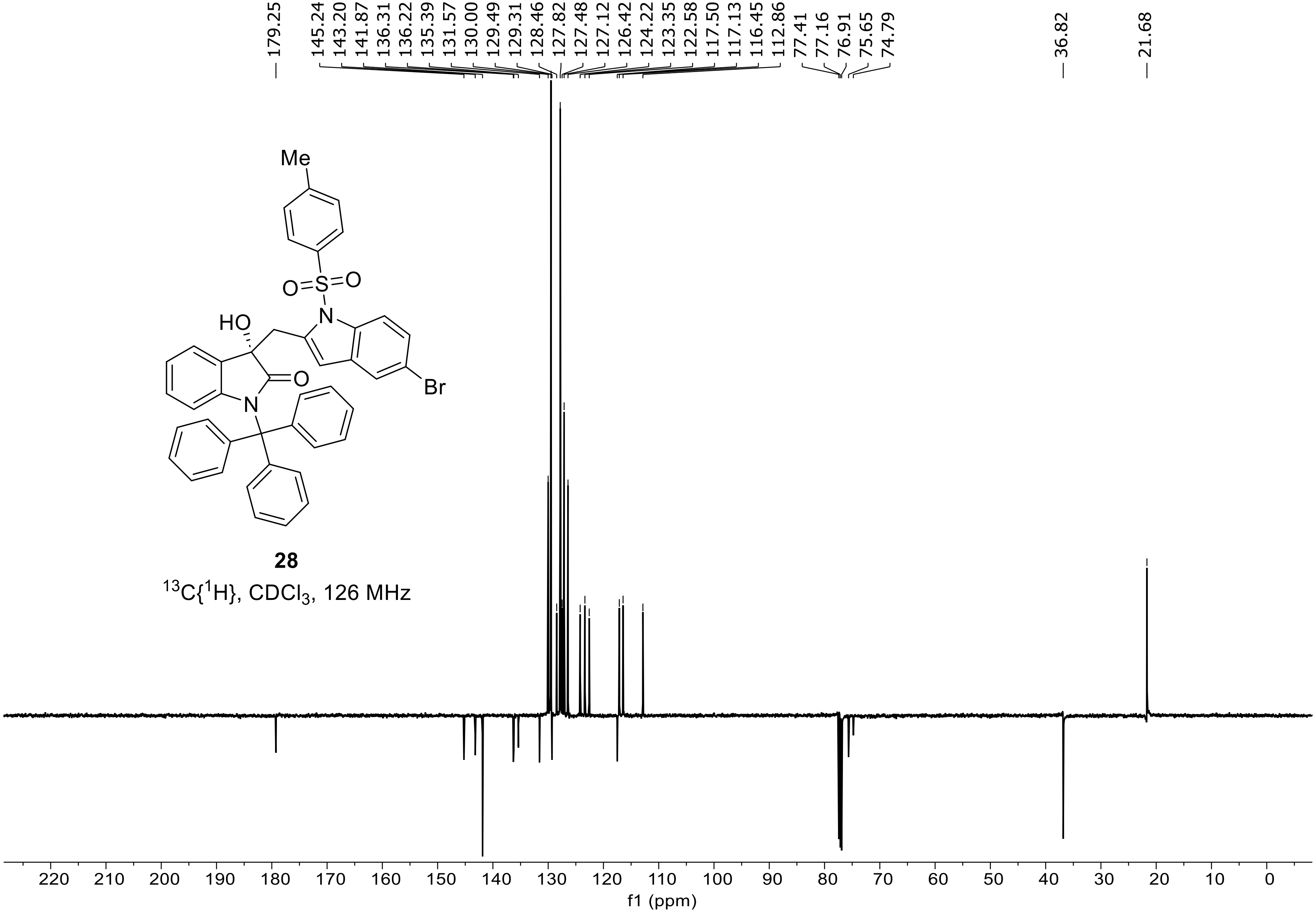

179.25
145.24
143.20
141.87
136.31
136.22
135.39
131.57
130.00
129.49
129.31
128.46
127.82
127.48
127.12
126.42
124.22
123.35
122.58
117.50
117.13
116.45
112.86
77.41
77.16
76.91
75.65
74.79
36.82
21.68
Me
O=S=O
N
HO
O
N
Br
28
$^{13}C\{^{1}H\}$, $CDCl_3$, 126 MHz
220 210 200 190 180 170 160 150 140 130 120 110 100 90 80 70 60 50 40 30 20 10 0
f1 (ppm)

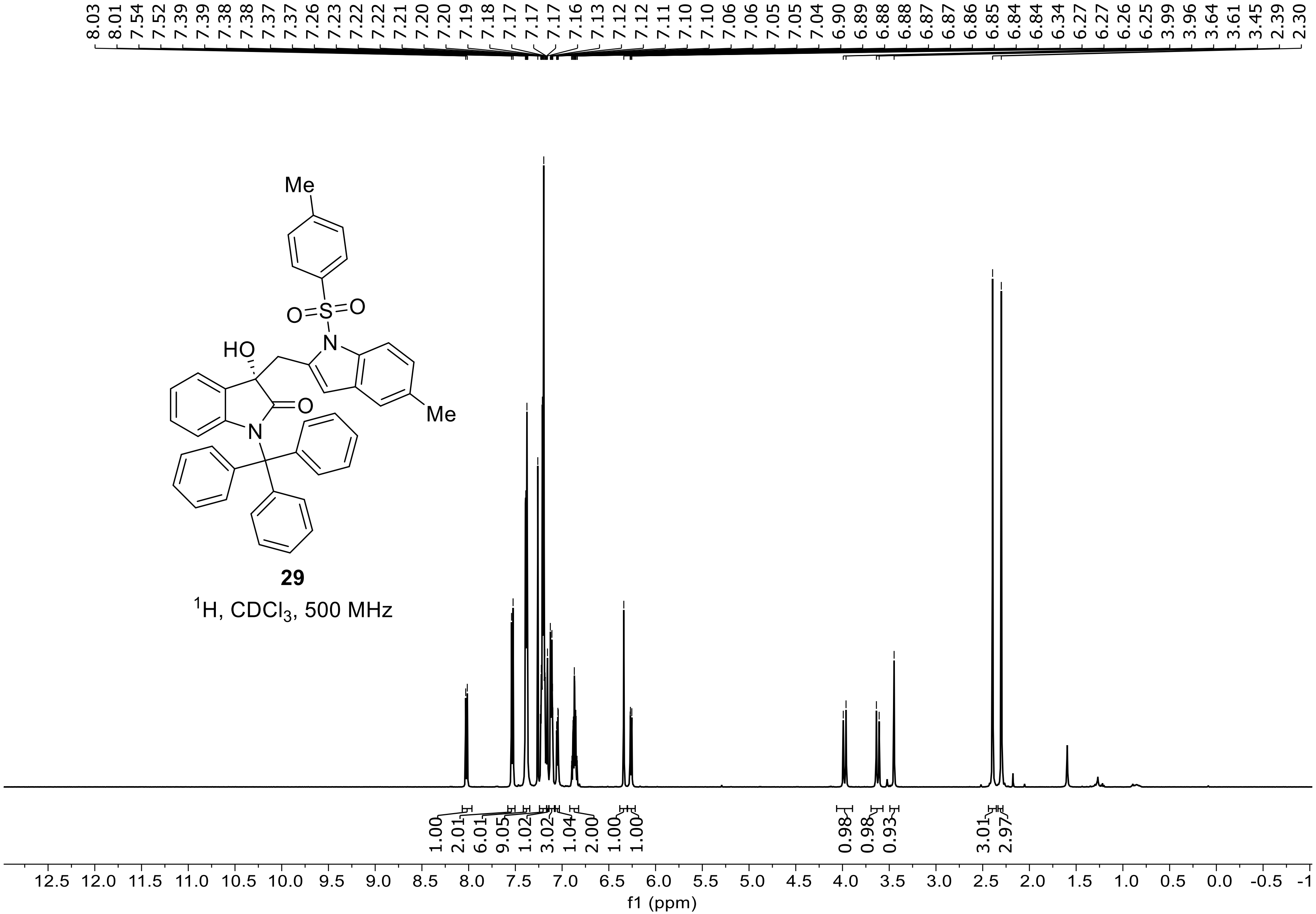
8.03 8.01 7.54 7.52 7.39 7.39 7.38 7.38 7.37 7.37 7.26 7.23 7.22 7.22 7.21 7.20 7.20 7.19 7.18 7.17 7.17 7.17 7.16 7.13 7.12 7.12 7.11 7.10 7.10 7.06 7.06 7.05 7.05 7.04 6.90 6.89 6.88 6.88 6.87 6.87 6.86 6.85 6.84 6.84 6.34 6.27 6.27 6.26 6.25 3.99 3.96 3.64 3.61 3.45 2.39 2.30
Me
O=S=O
N
HO
O
N
Me
29
1H, CDCl3, 500 MHz
1.00 2.01 6.01 9.05 1.02 3.02 1.04 2.00 1.00 1.00 0.98 0.98 0.93 3.01 2.97
12.5 12.0 11.5 11.0 10.5 10.0 9.5 9.0 8.5 8.0 7.5 7.0 6.5 6.0 5.5 5.0 4.5 4.0 3.5 3.0 2.5 2.0 1.5 1.0 0.5 0.0 -0.5 -1
f1 (ppm)

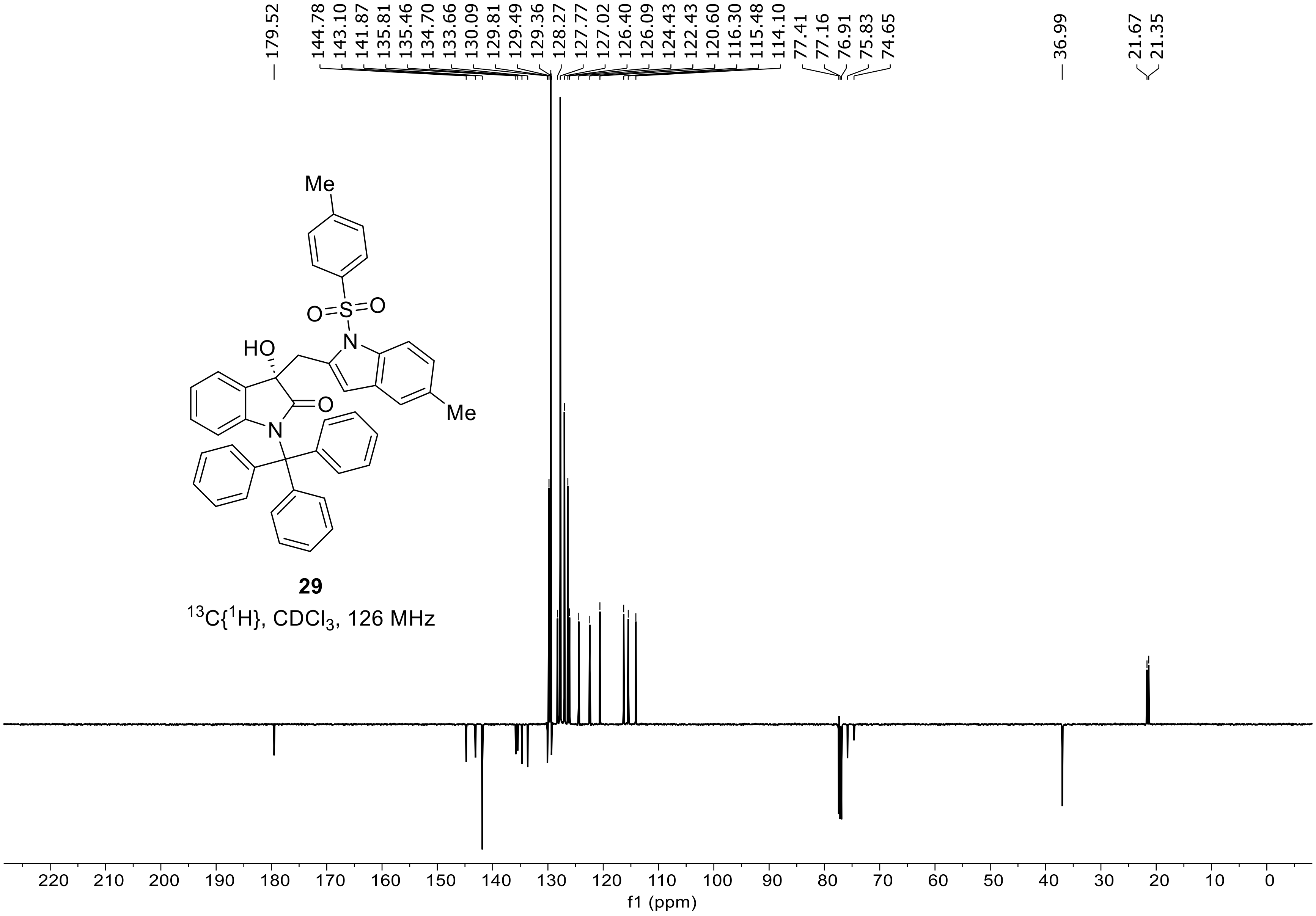

29

$^{13}C\{^{1}H\}$, $CDCl_3$, 126 MHz

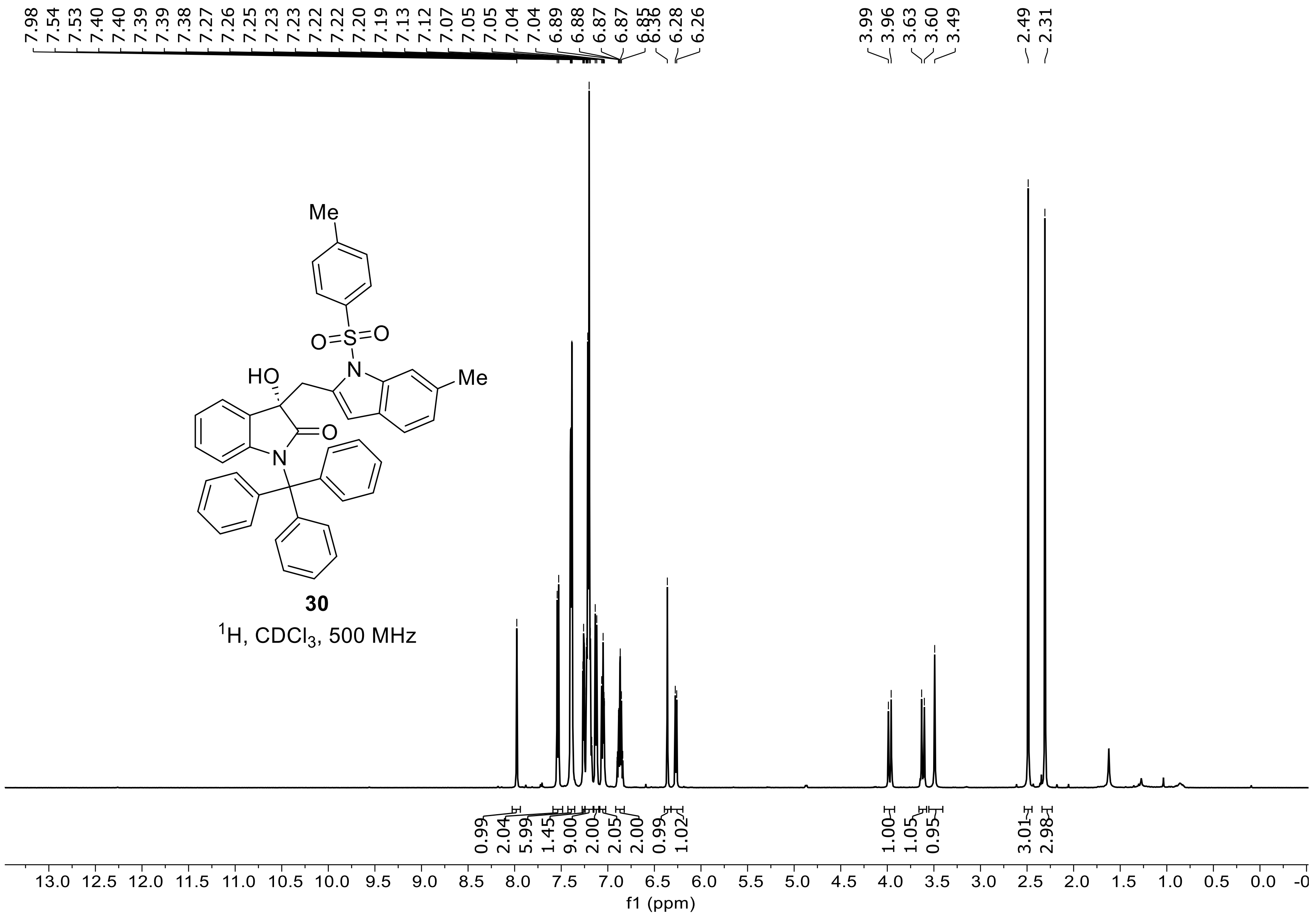
7.98
7.54
7.53
7.40
7.40
7.39
7.39
7.38
7.27
7.26
7.25
7.23
7.23
7.22
7.22
7.20
7.19
7.13
7.12
7.07
7.05
7.05
7.04
7.04
6.89
6.88
6.87
6.87
6.85
6.36
6.28
6.26
3.99
3.96
3.63
3.60
3.49
2.49
2.31
Me
O=S=O
N
Me
HO
O
N
30
^{1}H, $CDCl_3$, 500 MHz
0.99
2.04
5.99
1.45
9.00
2.00
2.05
2.00
0.99
1.02
1.00
1.05
0.95
3.01
2.98
13.0 12.5 12.0 11.5 11.0 10.5 10.0 9.5 9.0 8.5 8.0 7.5 7.0 6.5 6.0 5.5 5.0 4.5 4.0 3.5 3.0 2.5 2.0 1.5 1.0 0.5 0.0 -0
f1 (ppm)

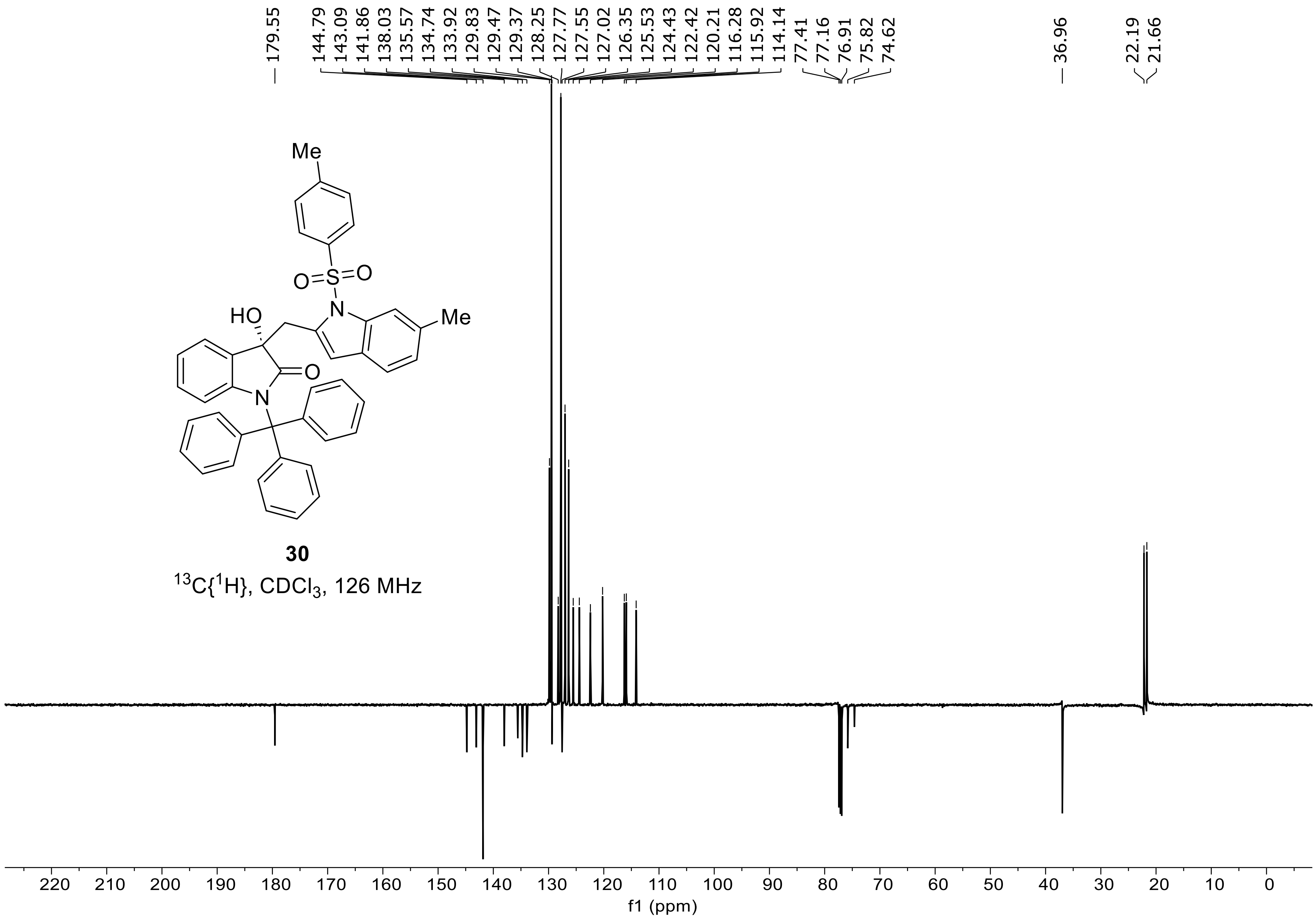
179.55
144.79
143.09
141.86
138.03
135.57
134.74
133.92
129.83
129.47
129.37
128.25
127.77
127.55
127.02
126.35
125.53
124.43
122.42
120.21
116.28
115.92
114.14
77.41
77.16
76.91
75.82
74.62
36.96
22.19
21.66
Me
O=S=O
N
HO
Me
O
N
30
$^{13}C\{^1H\}$, $CDCl_3$, 126 MHz
220 210 200 190 180 170 160 150 140 130 120 110 100 90 80 70 60 50 40 30 20 10 0
f1 (ppm)

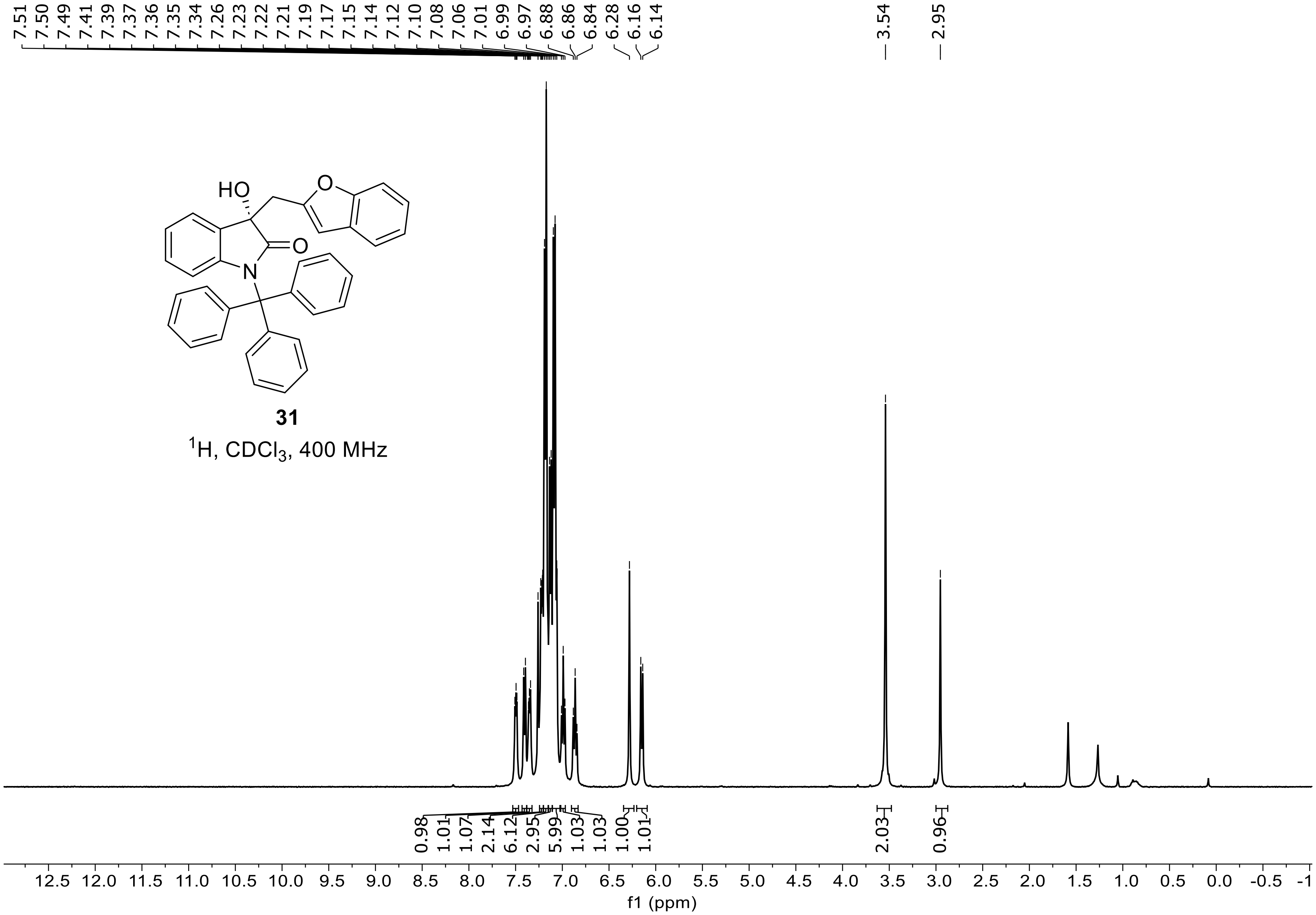
7.51 7.50 7.49 7.41 7.39 7.37 7.36 7.35 7.34 7.26 7.23 7.22 7.21 7.19 7.17 7.15 7.14 7.12 7.10 7.08 7.06 7.01 6.99 6.97 6.88 6.86 6.84 6.28 6.16 6.14
3.54
2.95
HO
O
N
31
^{1}H, CDCl$_3$, 400 MHz
0.98 1.01 1.07 2.14 6.12 2.95 5.99 1.03 1.03 1.00 1.01
2.03
0.96
12.5 12.0 11.5 11.0 10.5 10.0 9.5 9.0 8.5 8.0 7.5 7.0 6.5 6.0 5.5 5.0 4.5 4.0 3.5 3.0 2.5 2.0 1.5 1.0 0.5 0.0 -0.5 -1
f1 (ppm)

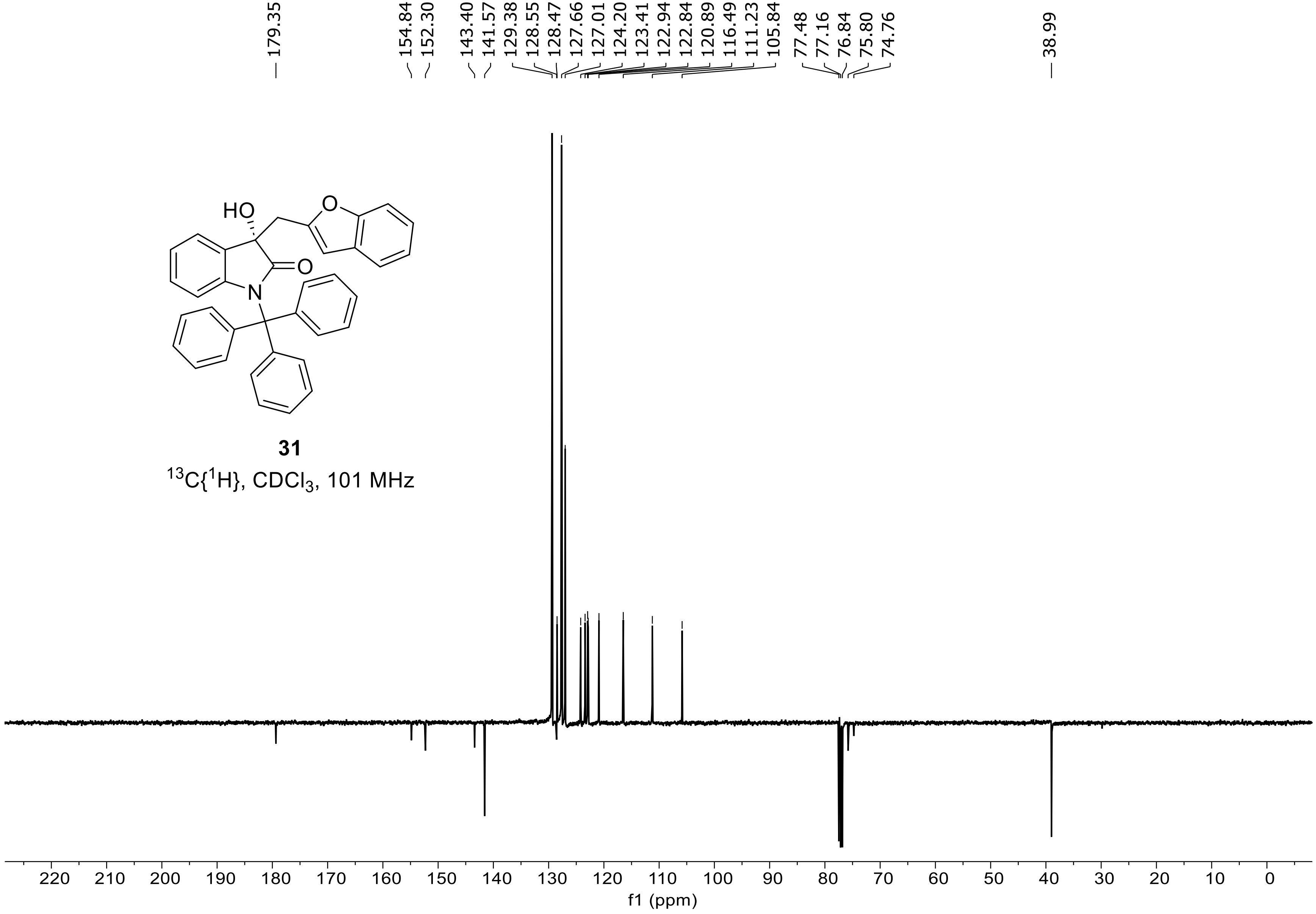

179.35
154.84
152.30
143.40
141.57
129.38
128.55
128.47
127.66
127.01
124.20
123.41
122.94
122.84
120.89
116.49
111.23
105.84
77.48
77.16
76.84
75.80
74.76
38.99
HO
O
O
N
31
$^{13}C\{^1H\}$, $CDCl_3$, 101 MHz
220 210 200 190 180 170 160 150 140 130 120 110 100 90 80 70 60 50 40 30 20 10 0
f1 (ppm)

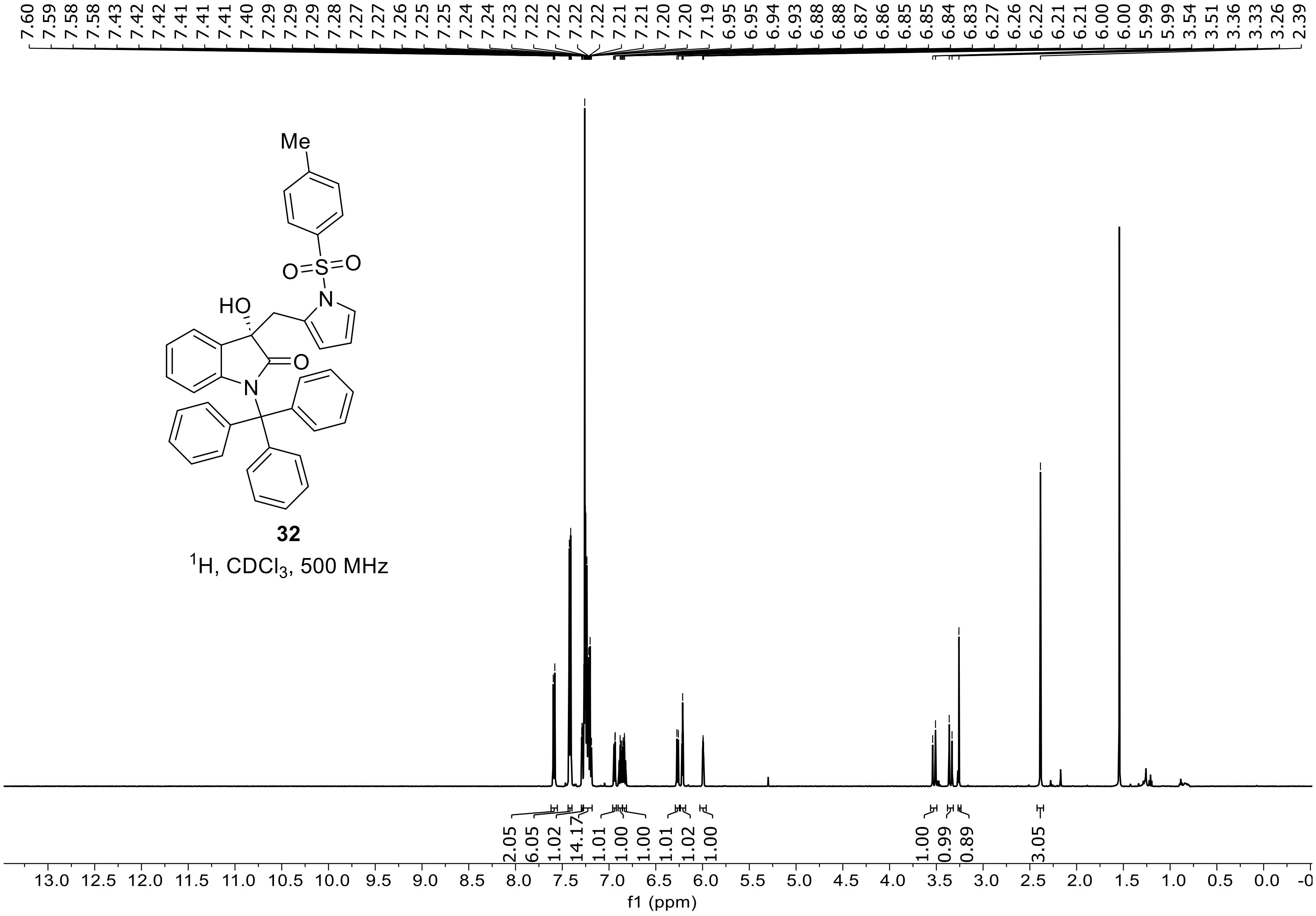
7.60 7.59 7.58 7.58 7.43 7.42 7.42 7.41 7.41 7.41 7.40 7.29 7.29 7.29 7.28 7.27 7.27 7.26 7.25 7.25 7.24 7.24 7.23 7.22 7.22 7.22 7.22 7.21 7.21 7.20 7.20 7.19 6.95 6.95 6.94 6.93 6.88 6.88 6.87 6.86 6.85 6.85 6.84 6.83 6.27 6.26 6.22 6.21 6.21 6.00 6.00 5.99 5.99 3.54 3.51 3.36 3.33 3.26 2.39
Me
O=S=O
N
HO
O
N
32
^{1}H, CDCl$_3$, 500 MHz
2.05 6.05 1.02 14.17 1.01 1.00 1.00 1.01 1.02 1.00 1.00 0.99 0.89 3.05
13.0 12.5 12.0 11.5 11.0 10.5 10.0 9.5 9.0 8.5 8.0 7.5 7.0 6.5 6.0 5.5 5.0 4.5 4.0 3.5 3.0 2.5 2.0 1.5 1.0 0.5 0.0 -0
f1 (ppm)

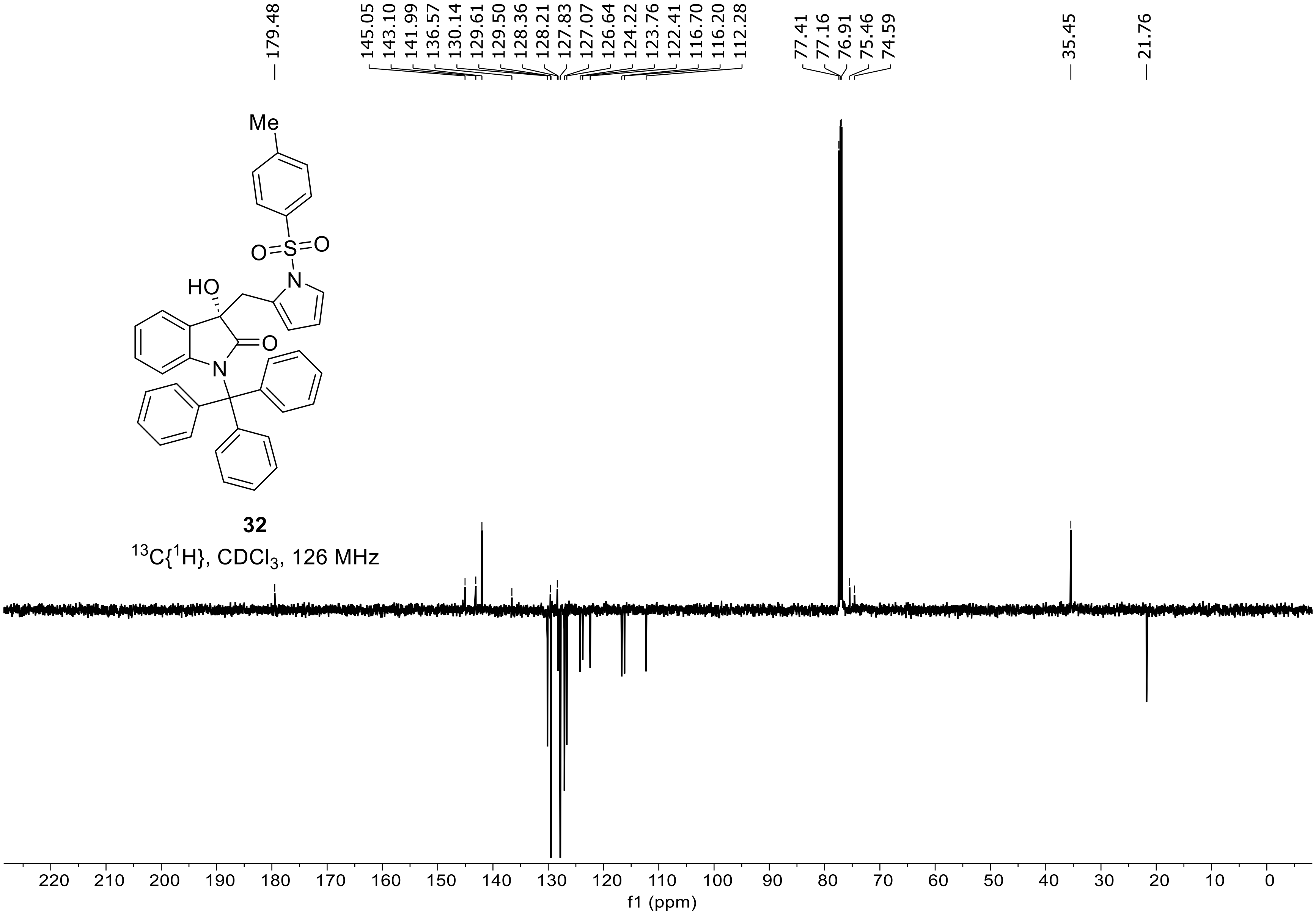

32

$^{13}C\{^{1}H\}$, $CDCl_3$, 126 MHz

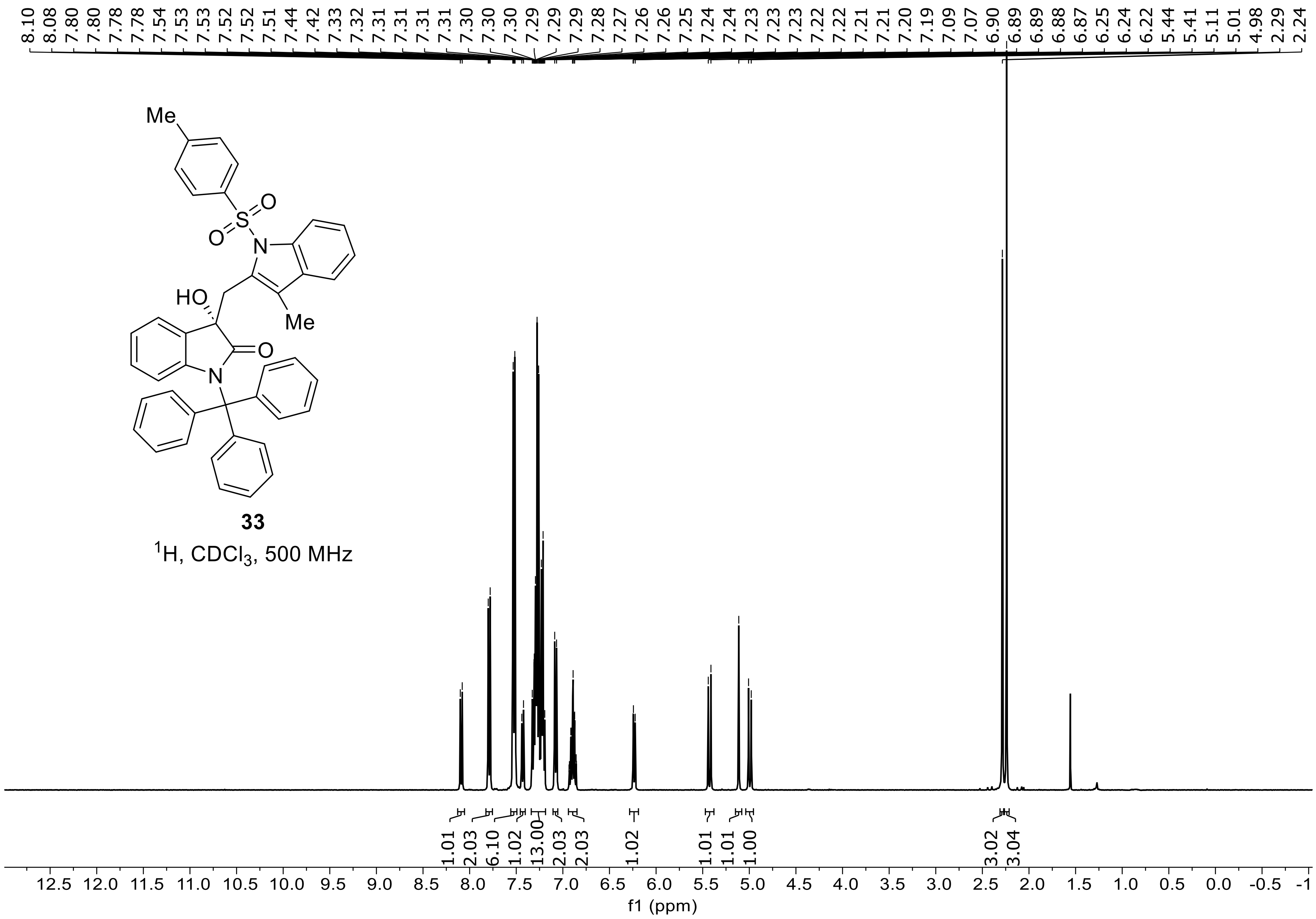

33

^{1}H, $CDCl_3$, 500 MHz

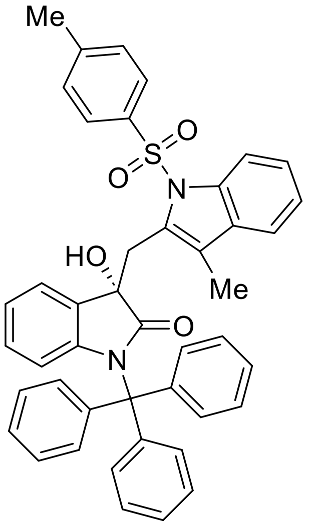

33

$^{13}C\{^{1}H\}$, $CDCl_3$, 126 MHz

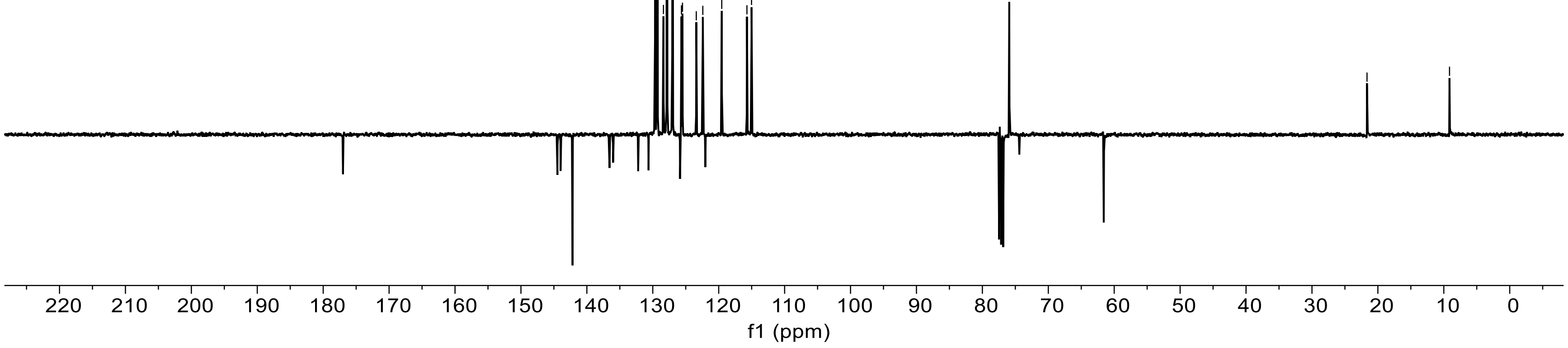

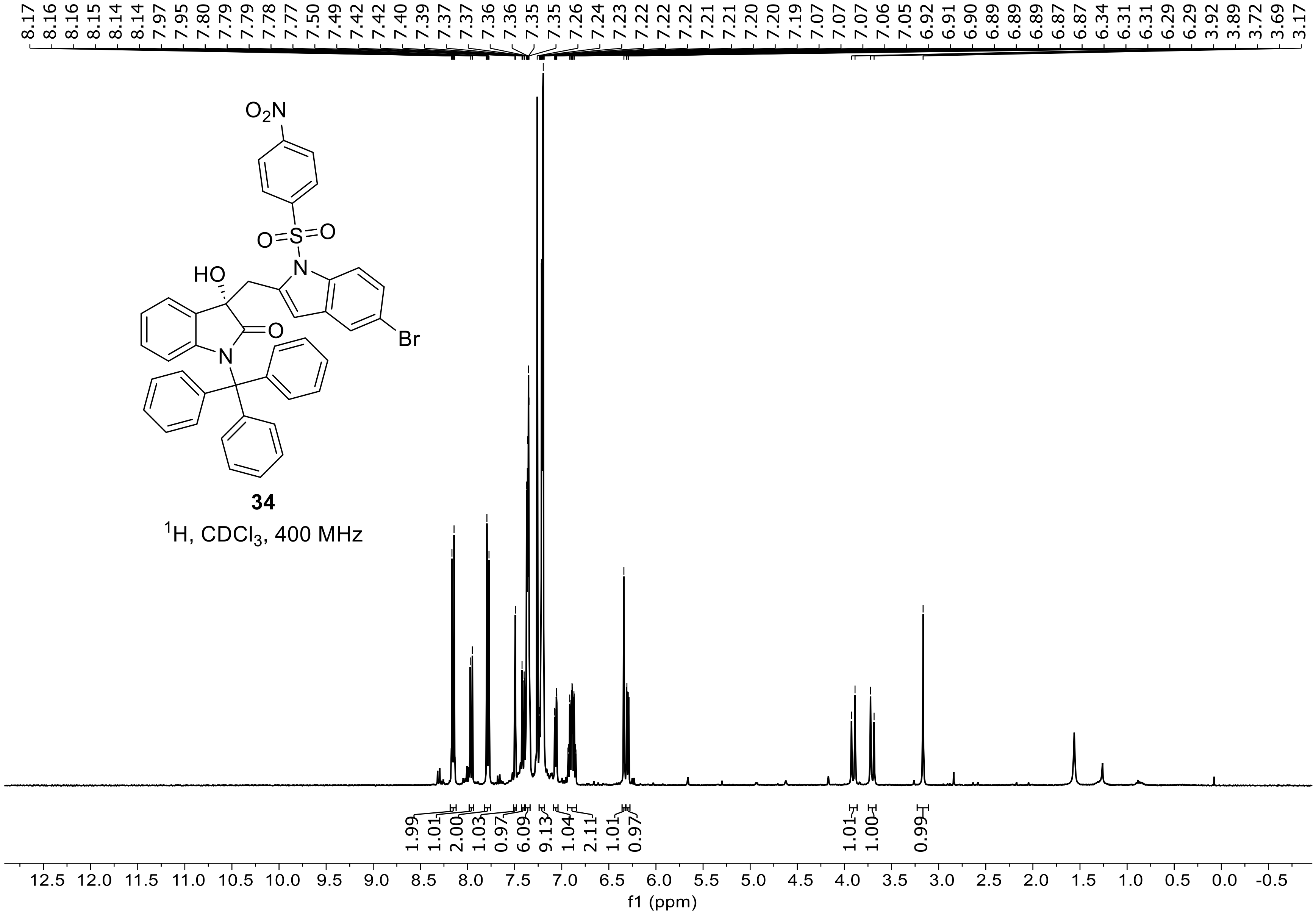

34

^{1}H, $CDCl_3$, 400 MHz

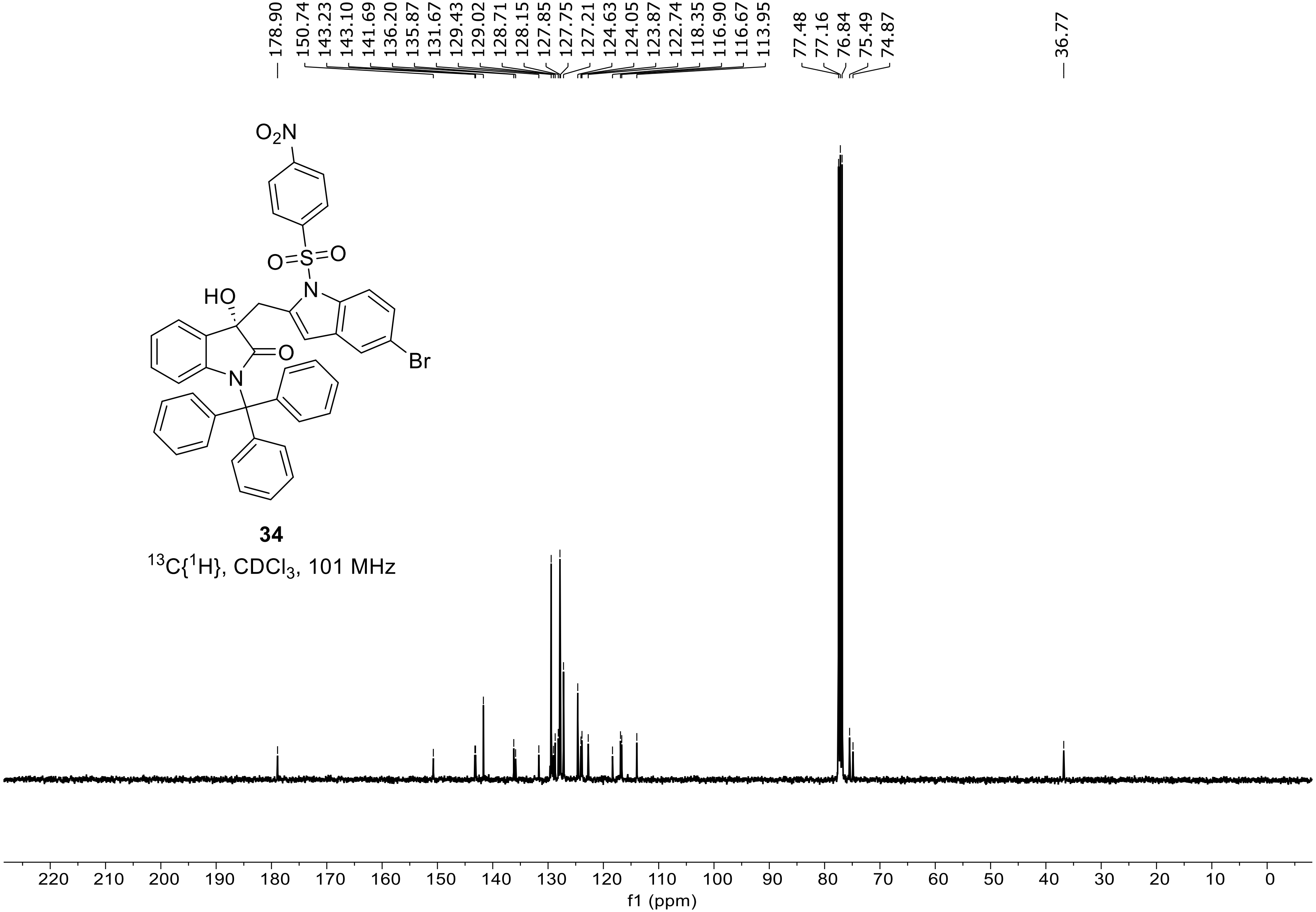

34

$^{13}C\{^{1}H\}$, $CDCl_3$, 101 MHz

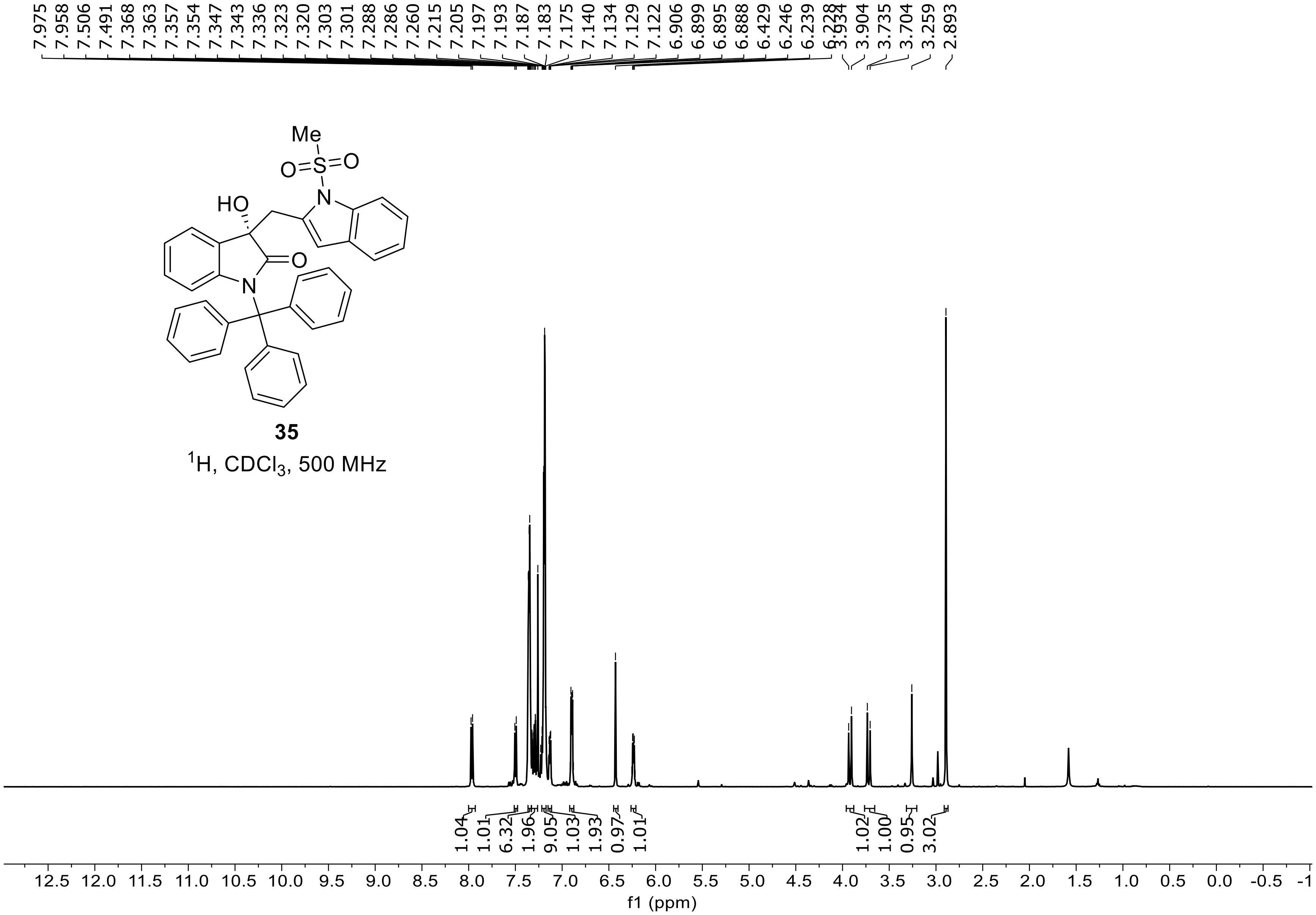
7.975
7.958
7.506
7.491
7.368
7.363
7.357
7.354
7.347
7.343
7.336
7.323
7.320
7.303
7.301
7.288
7.286
7.260
7.215
7.205
7.197
7.193
7.187
7.183
7.175
7.140
7.134
7.129
7.122
6.906
6.899
6.895
6.888
6.429
6.246
6.239
6.228
3.934
3.904
3.735
3.704
3.259
2.893
Me
O=S=O
N
HO
O
N
35
1H, CDCl3, 500 MHz
1.04
1.01
6.32
1.96
9.05
1.03
1.93
0.97
1.01
1.02
1.00
0.95
3.02
12.5 12.0 11.5 11.0 10.5 10.0 9.5 9.0 8.5 8.0 7.5 7.0 6.5 6.0 5.5 5.0 4.5 4.0 3.5 3.0 2.5 2.0 1.5 1.0 0.5 0.0 -0.5 -1
f1 (ppm)

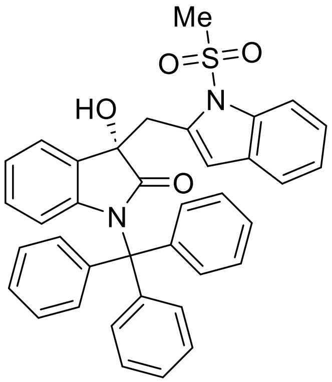

35

$^{13}C\{^1H\}$, $CDCl_3$, 126 MHz

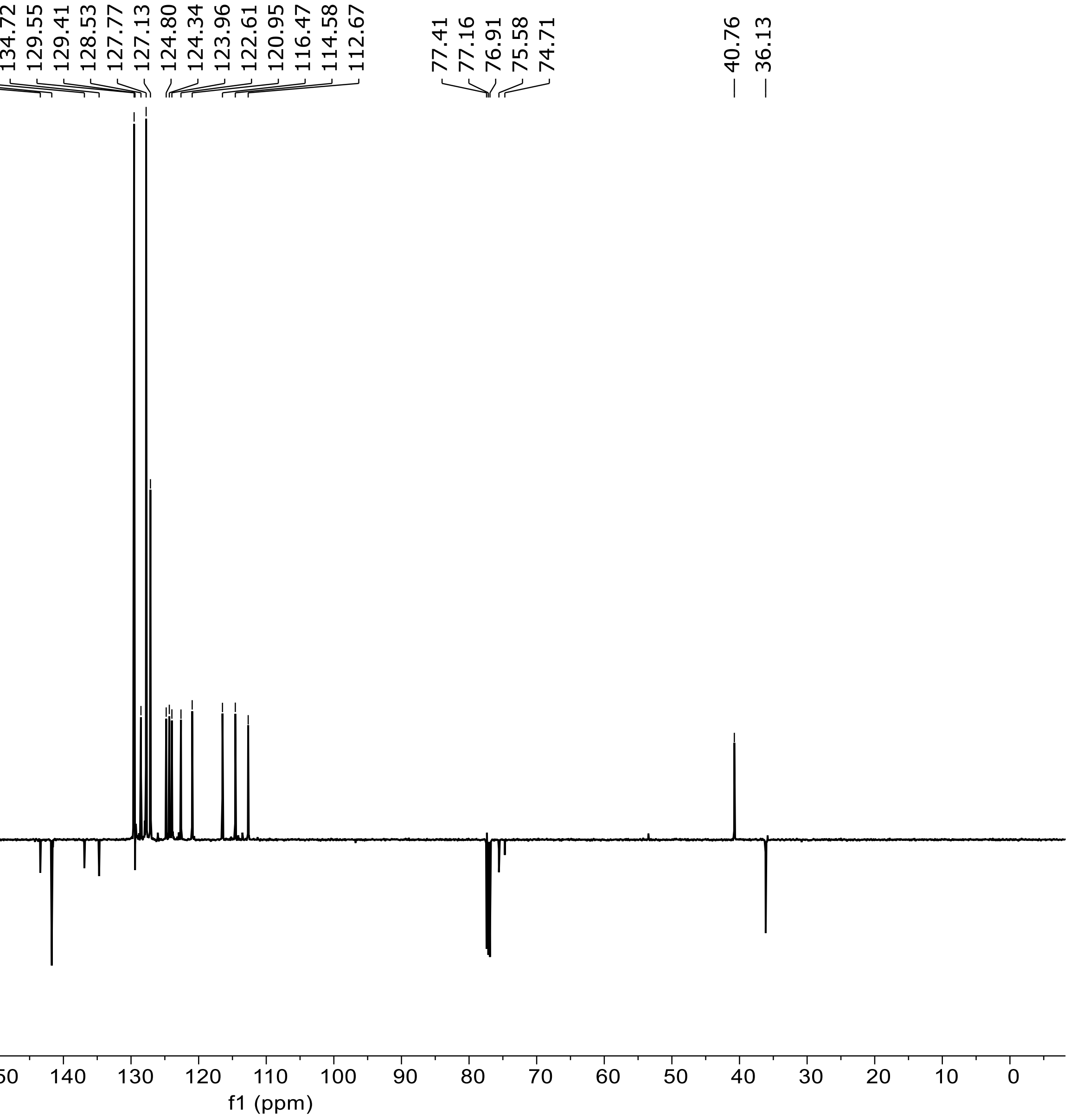

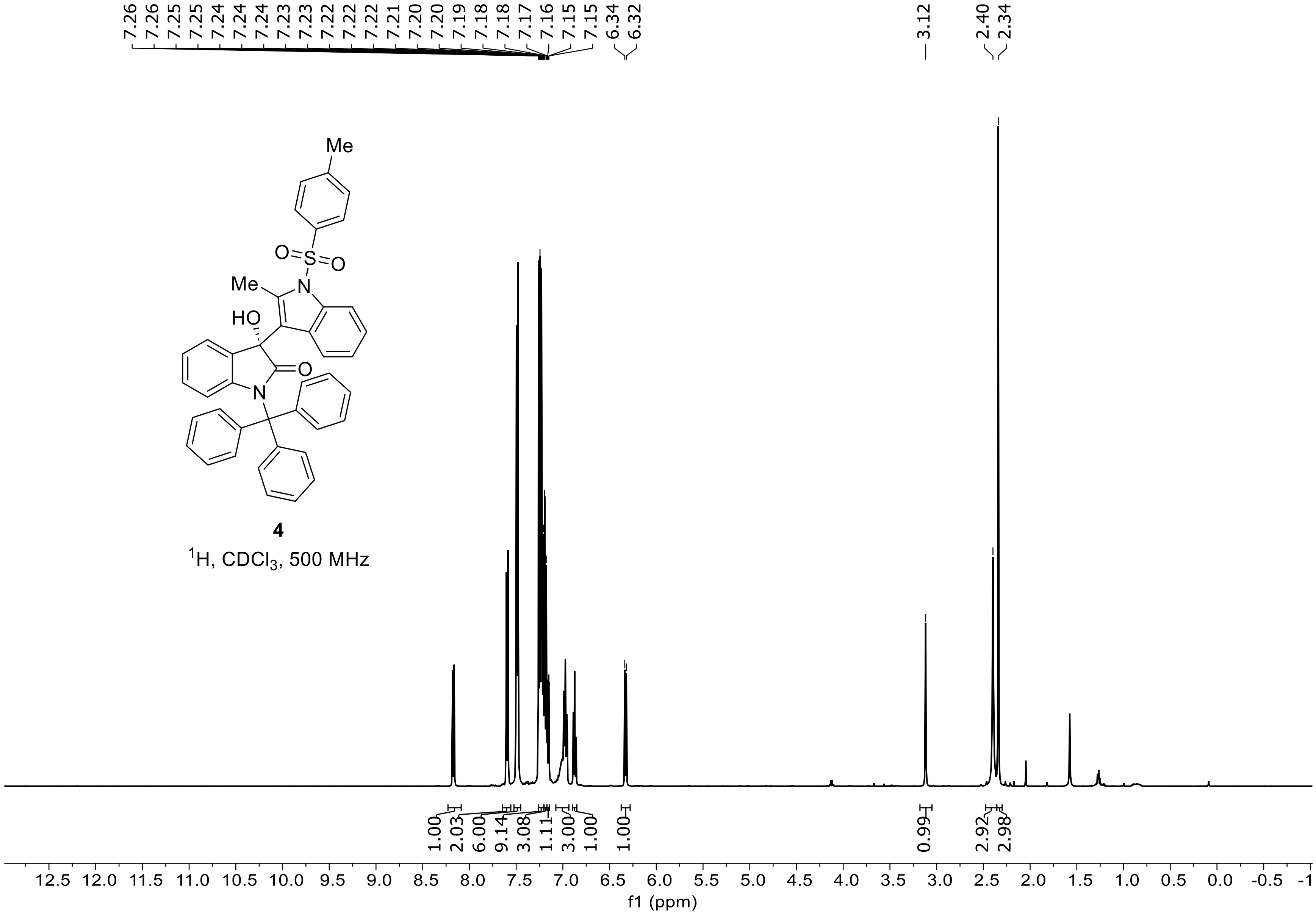
7.26 7.26 7.25 7.25 7.24 7.24 7.24 7.23 7.23 7.22 7.22 7.22 7.21 7.20 7.20 7.19 7.18 7.18 7.17 7.16 7.15 7.15 6.34 6.32
3.12
2.40 2.34
Me
O=S=O
Me
N
HO
O
N
4
^{1}H, CDCl$_3$, 500 MHz
1.00 2.03 6.00 9.14 3.08 1.11 3.00 1.00 1.00
0.99
2.92 2.98
12.5 12.0 11.5 11.0 10.5 10.0 9.5 9.0 8.5 8.0 7.5 7.0 6.5 6.0 5.5 5.0 4.5 4.0 3.5 3.0 2.5 2.0 1.5 1.0 0.5 0.0 -0.5 -1
f1 (ppm)

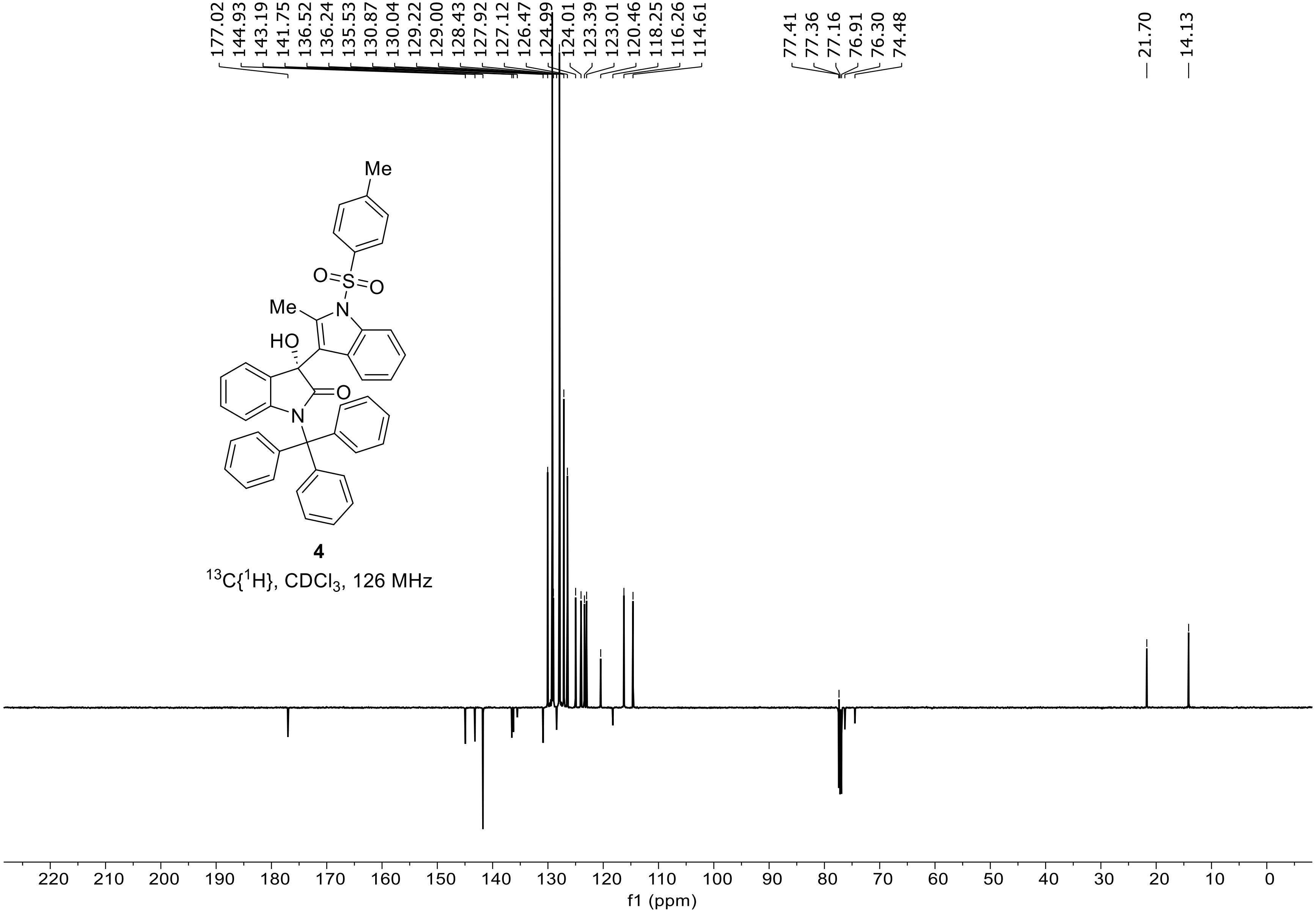
177.02
144.93
143.19
141.75
136.52
136.24
135.53
130.87
130.04
129.22
129.00
128.43
127.92
127.12
126.47
124.99
124.01
123.39
123.01
120.46
118.25
116.26
114.61
77.41
77.36
77.16
76.91
76.30
74.48
21.70
14.13
Me
O=S=O
N
Me
HO
O
N
4
$^{13}C\{^1H\}$, $CDCl_3$, 126 MHz
220 210 200 190 180 170 160 150 140 130 120 110 100 90 80 70 60 50 40 30 20 10 0
f1 (ppm)

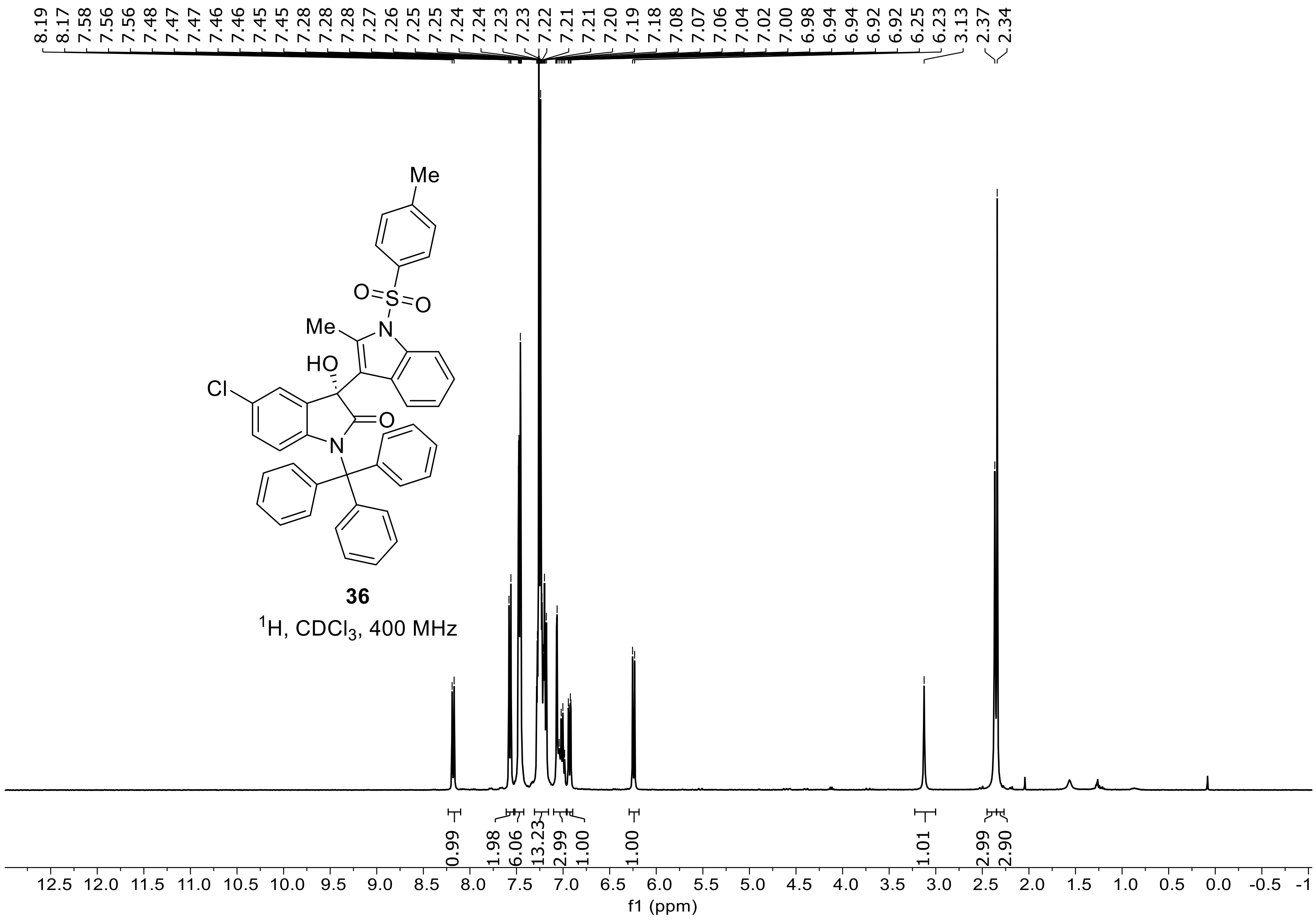
8.19 8.17 7.58 7.56 7.56 7.48 7.47 7.47 7.46 7.46 7.45 7.45 7.28 7.28 7.28 7.27 7.26 7.25 7.25 7.24 7.24 7.23 7.23 7.22 7.21 7.21 7.20 7.19 7.18 7.08 7.07 7.06 7.04 7.02 7.00 6.98 6.94 6.94 6.92 6.92 6.25 6.23 3.13 2.37 2.34
Me
O=S=O
Me
N
HO
Cl
O
N
36
^{1}H, $CDCl_3$, 400 MHz
0.99 1.98 6.06 13.23 2.99 1.00 1.00 1.01 2.99 2.90
12.5 12.0 11.5 11.0 10.5 10.0 9.5 9.0 8.5 8.0 7.5 7.0 6.5 6.0 5.5 5.0 4.5 4.0 3.5 3.0 2.5 2.0 1.5 1.0 0.5 0.0 -0.5 -1
f1 (ppm)

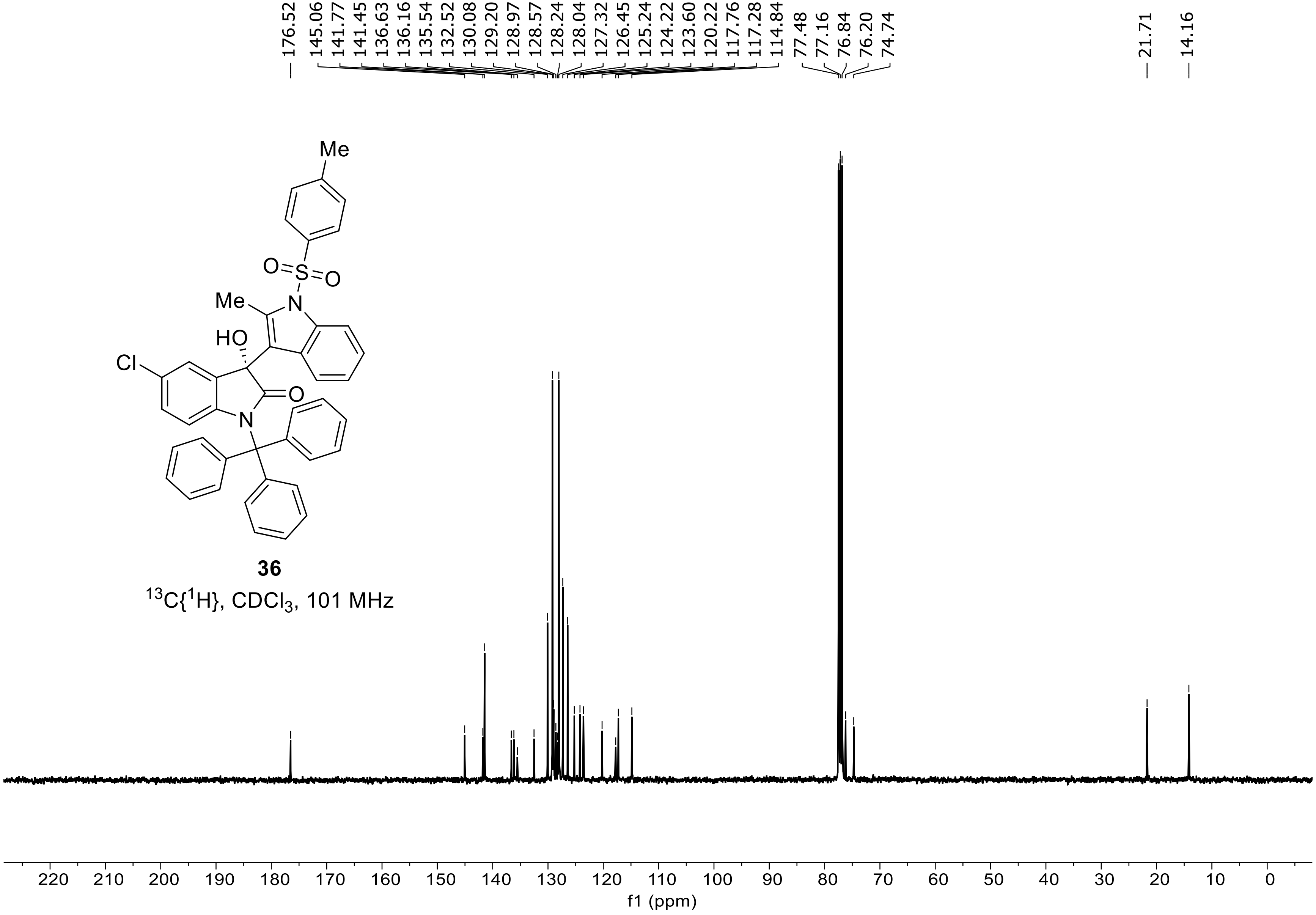
176.52
145.06
141.77
141.45
136.63
136.16
135.54
132.52
130.08
129.20
128.97
128.57
128.24
128.04
127.32
126.45
125.24
124.22
123.60
120.22
117.76
117.28
114.84
77.48
77.16
76.84
76.20
74.74
21.71
14.16
Me
O=S=O
N
HO
Cl
O
36
13C{1H}, CDCl3, 101 MHz
220 210 200 190 180 170 160 150 140 130 120 110 100 90 80 70 60 50 40 30 20 10 0
f1 (ppm)

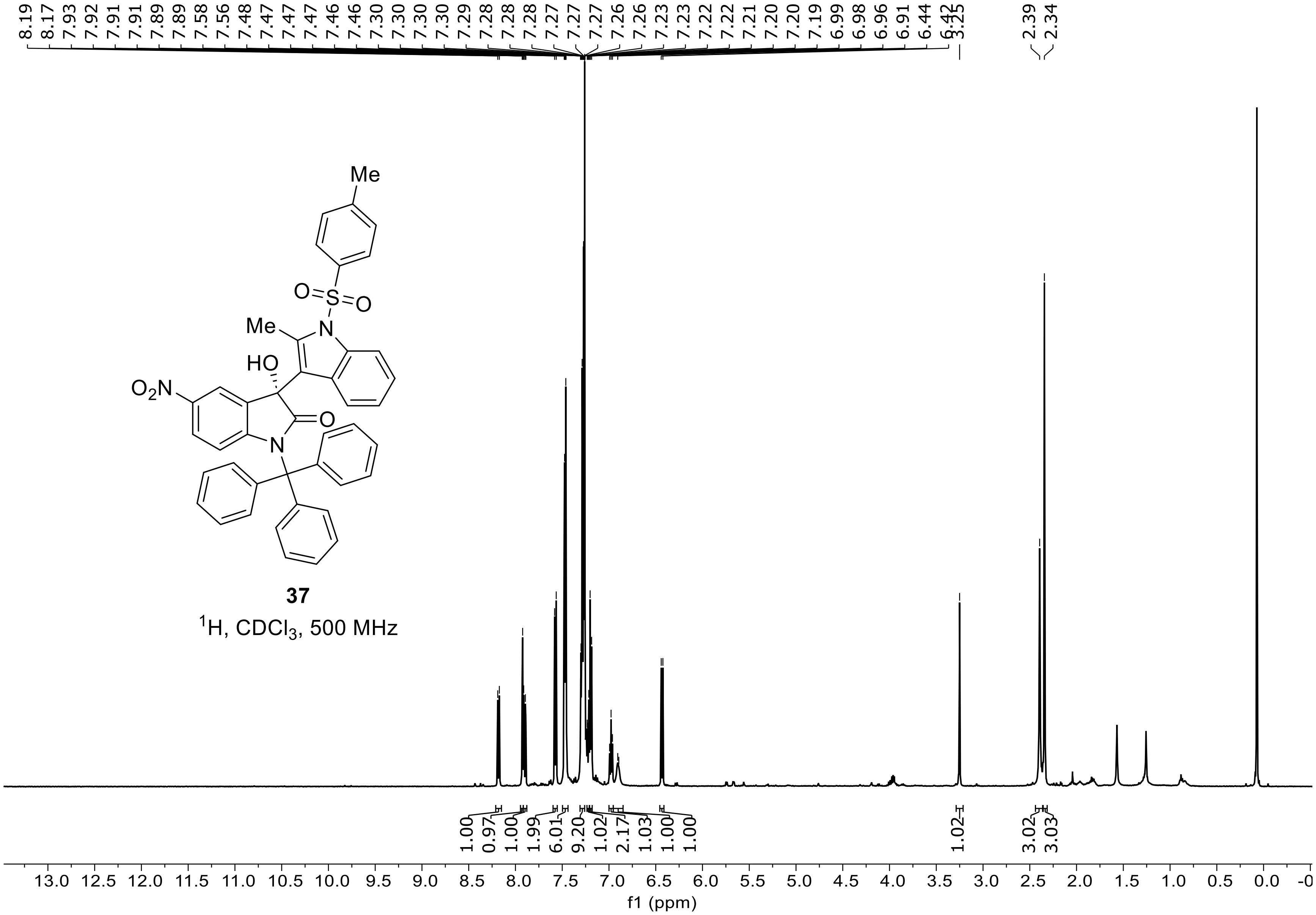
8.19 8.17 7.93 7.92 7.91 7.91 7.89 7.89 7.58 7.56 7.48 7.47 7.47 7.47 7.46 7.46 7.30 7.30 7.30 7.30 7.29 7.28 7.28 7.28 7.27 7.27 7.27 7.26 7.26 7.23 7.23 7.22 7.22 7.21 7.20 7.20 7.19 6.99 6.98 6.96 6.91 6.44 6.42 3.25
2.39 2.34
Me
O=S=O
Me
N
HO
O2N
O
N
37
1H, CDCl3, 500 MHz
1.00 0.97 1.00 1.99 6.01 9.20 1.02 2.17 1.03 1.00 1.00
1.02
3.02 3.03
13.0 12.5 12.0 11.5 11.0 10.5 10.0 9.5 9.0 8.5 8.0 7.5 7.0 6.5 6.0 5.5 5.0 4.5 4.0 3.5 3.0 2.5 2.0 1.5 1.0 0.5 0.0 -0
f1 (ppm)

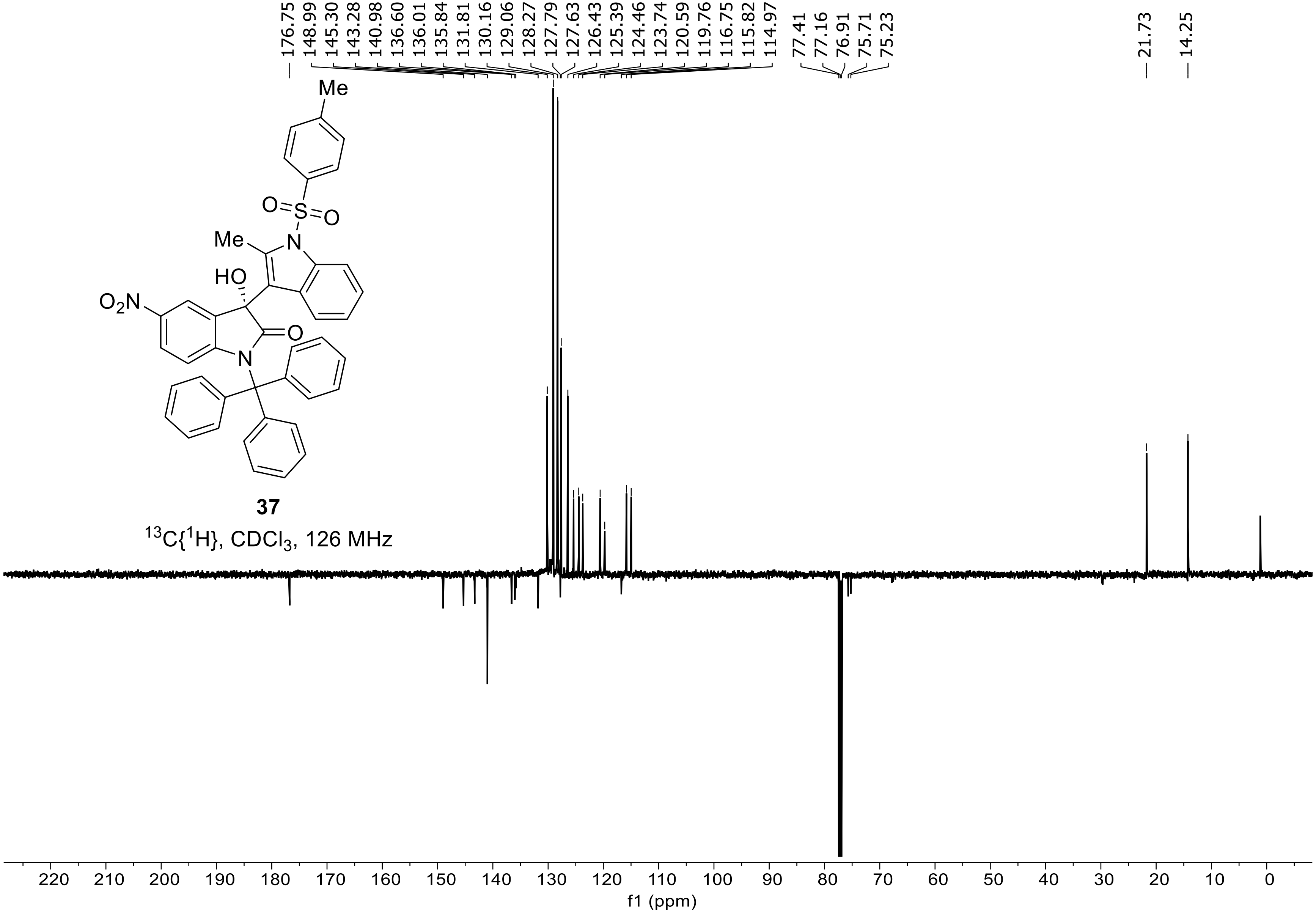
176.75
148.99
145.30
143.28
140.98
136.60
136.01
135.84
131.81
130.16
129.06
128.27
127.79
127.63
126.43
125.39
124.46
123.74
120.59
119.76
116.75
115.82
114.97
77.41
77.16
76.91
75.71
75.23
21.73
14.25
Me
O=S=O
N
Me
HO
O₂N
O
N
37
13C{1H}, CDCl3, 126 MHz
220 210 200 190 180 170 160 150 140 130 120 110 100 90 80 70 60 50 40 30 20 10 0
f1 (ppm)

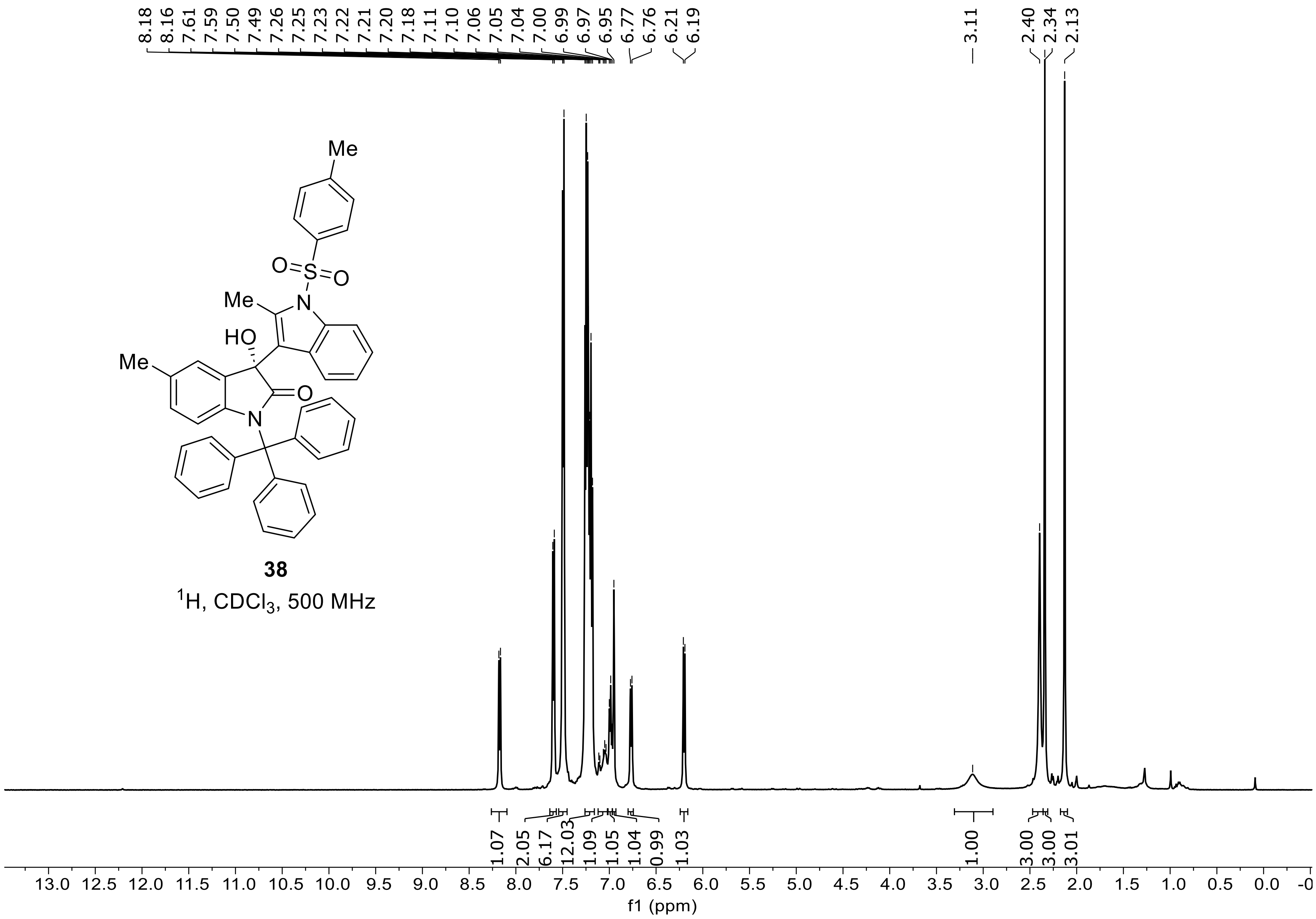
8.18 8.16 7.61 7.59 7.50 7.49 7.26 7.25 7.23 7.22 7.21 7.20 7.18 7.11 7.10 7.06 7.05 7.04 7.00 6.99 6.97 6.95 6.77 6.76 6.21 6.19
3.11
2.40 2.34 2.13
Me
O=S=O
Me
N
HO
Me
O
N
38
^{1}H, CDCl$_3$, 500 MHz
1.07 2.05 6.17 12.03 1.09 1.05 1.04 0.99 1.03
1.00
3.00 3.00 3.01
13.0 12.5 12.0 11.5 11.0 10.5 10.0 9.5 9.0 8.5 8.0 7.5 7.0 6.5 6.0 5.5 5.0 4.5 4.0 3.5 3.0 2.5 2.0 1.5 1.0 0.5 0.0 -0
f1 (ppm)

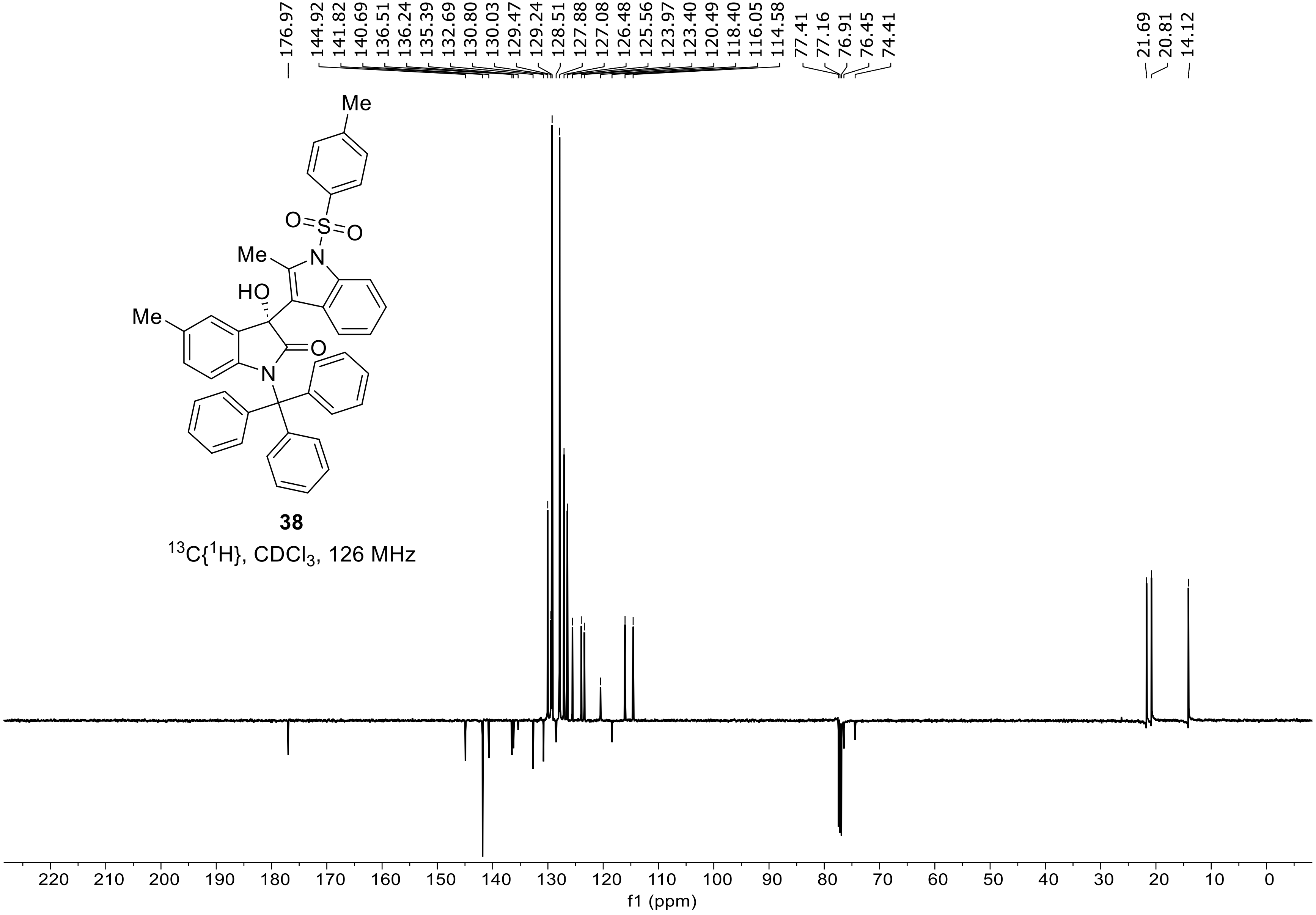

38

$^{13}C\{^{1}H\}$, $CDCl_3$, 126 MHz

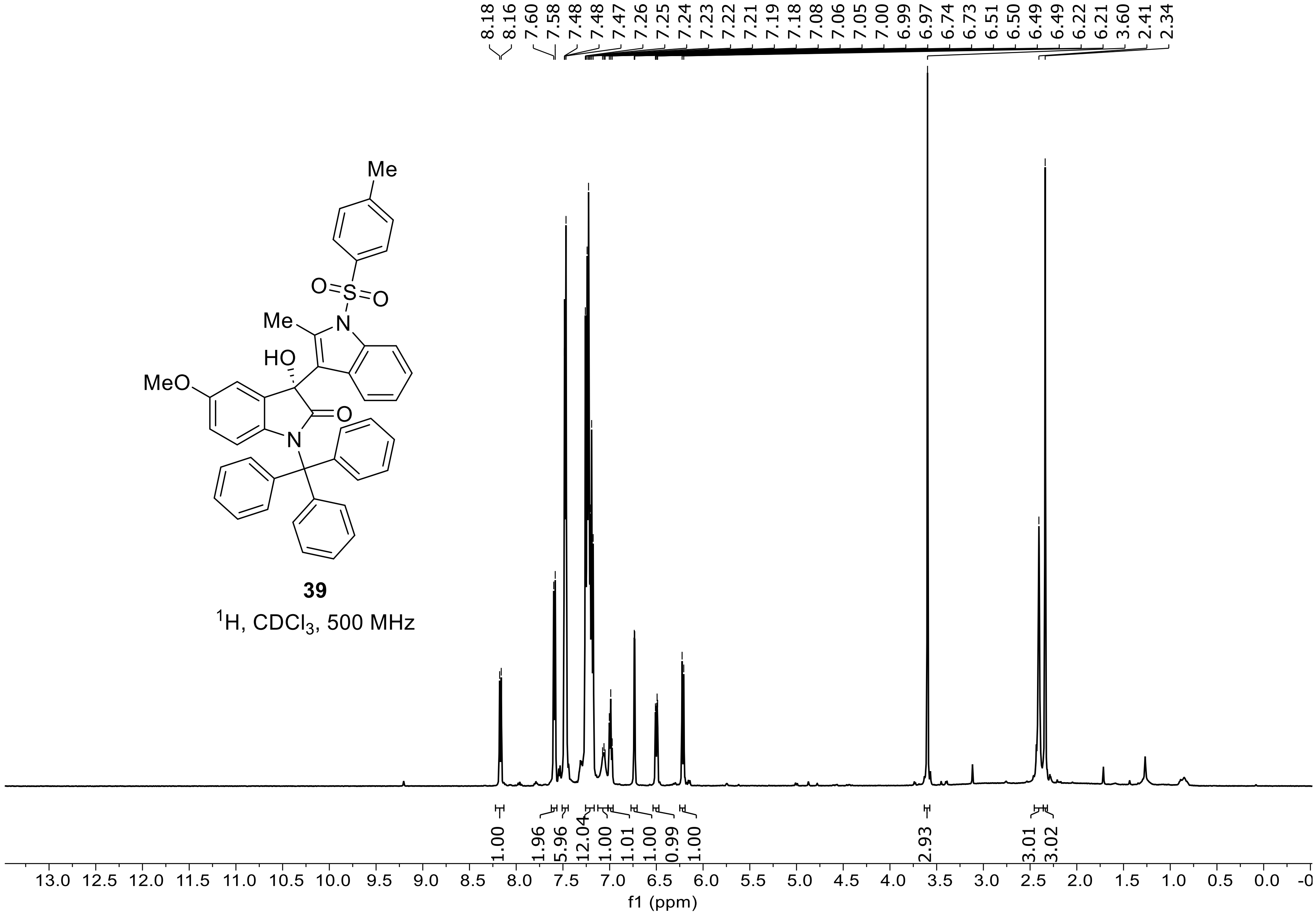

39

^{1}H, $CDCl_3$, 500 MHz

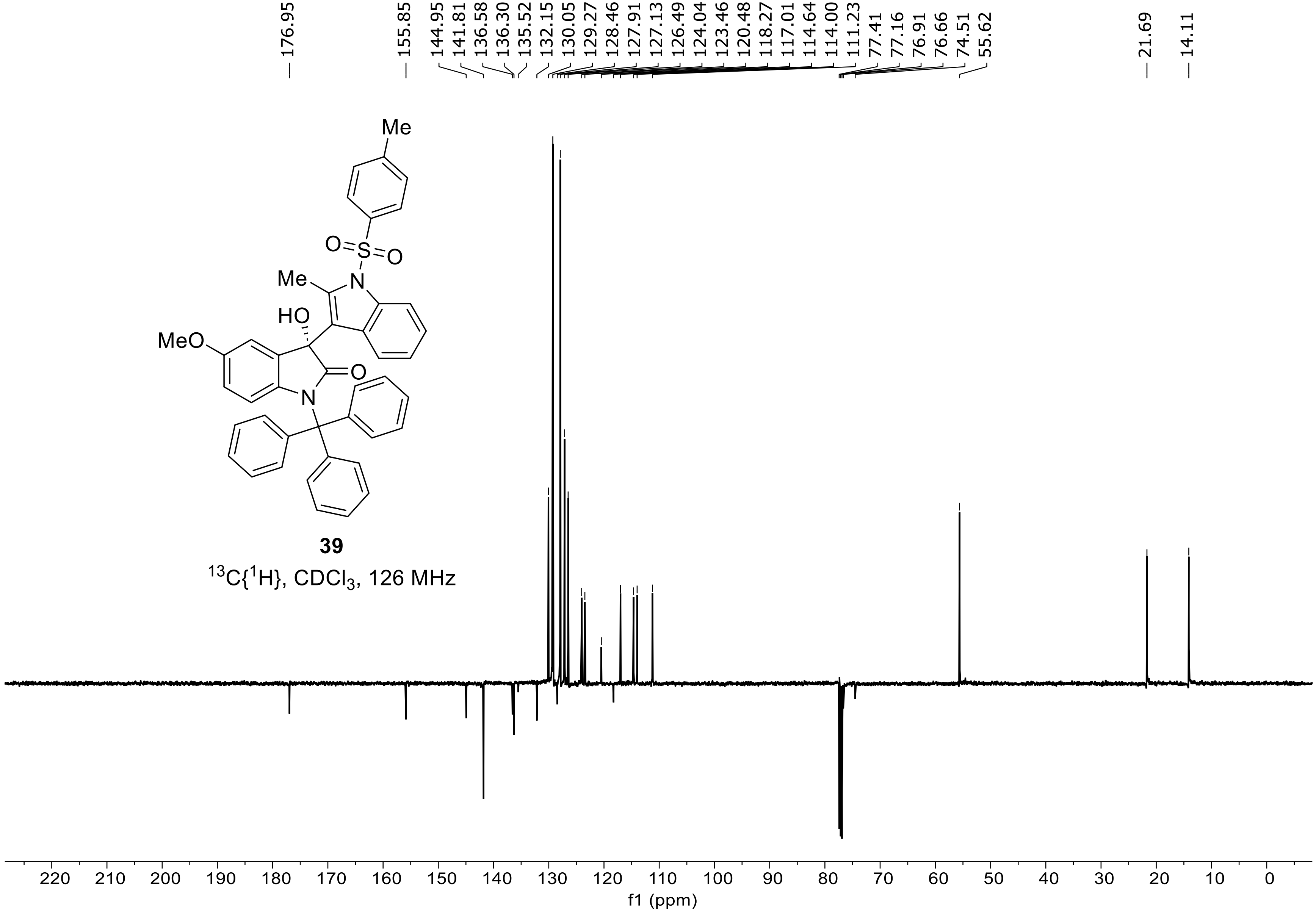
176.95
155.85
144.95
141.81
136.58
136.30
135.52
132.15
130.05
129.27
128.46
127.91
127.13
126.49
124.04
123.46
120.48
118.27
117.01
114.64
114.00
111.23
77.41
77.16
76.91
76.66
74.51
55.62
21.69
14.11
Me
O=S=O
N
Me
HO
MeO
O
N
39
13C{1H}, CDCl3, 126 MHz
220 210 200 190 180 170 160 150 140 130 120 110 100 90 80 70 60 50 40 30 20 10 0
f1 (ppm)

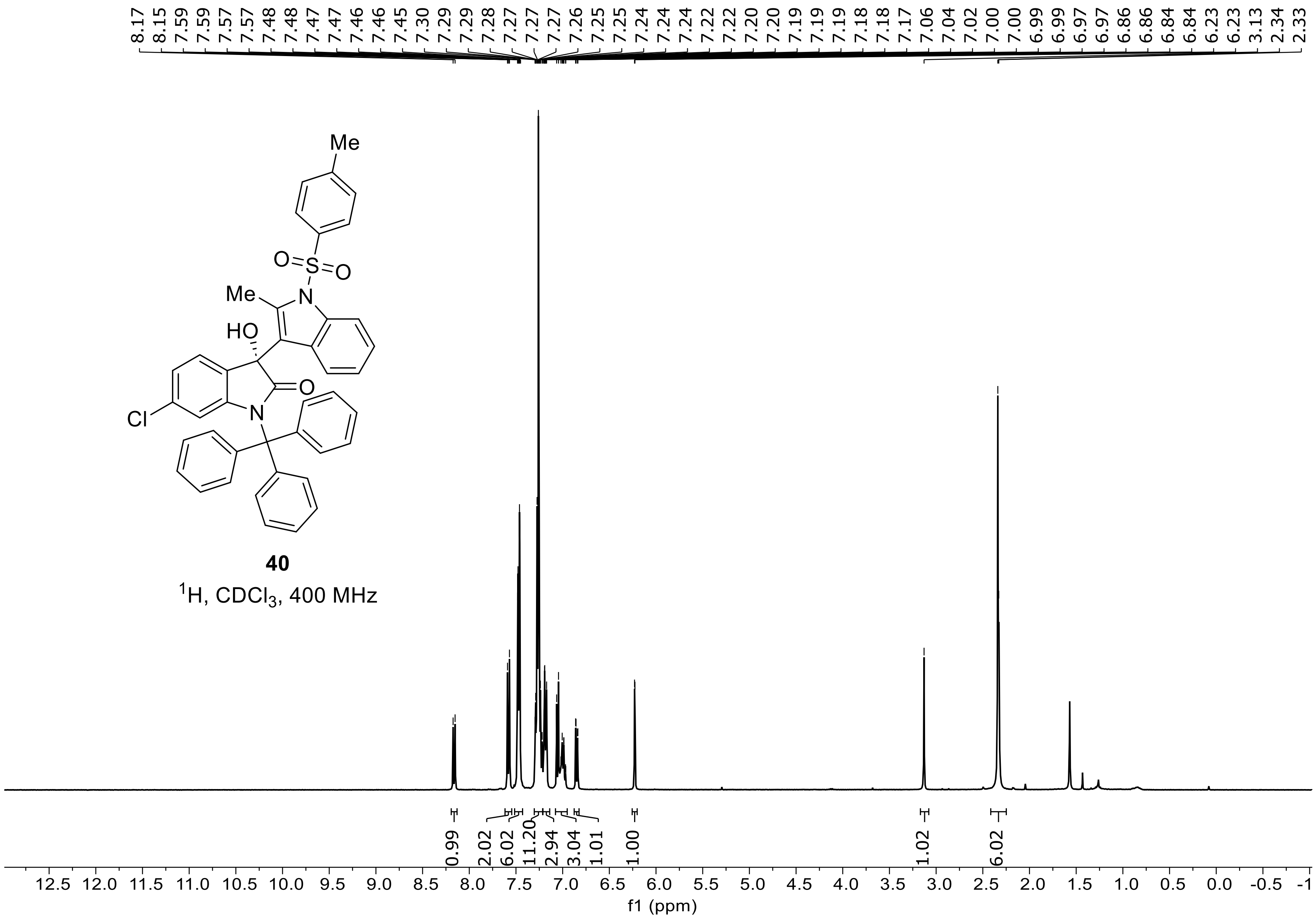
Me
O=S=O
Me
N
HO
O
N
Cl
40
1H, CDCl3, 400 MHz
8.17 8.15 7.59 7.59 7.57 7.57 7.48 7.48 7.47 7.47 7.46 7.46 7.45 7.30 7.29 7.29 7.28 7.27 7.27 7.27 7.26 7.25 7.25 7.24 7.24 7.24 7.22 7.22 7.20 7.20 7.19 7.19 7.19 7.18 7.18 7.17 7.06 7.04 7.02 7.00 7.00 6.99 6.99 6.97 6.97 6.86 6.86 6.84 6.84 6.23 6.23 3.13 2.34 2.33
0.99 2.02 6.02 11.20 2.94 3.04 1.01 1.00 1.02 6.02
12.5 12.0 11.5 11.0 10.5 10.0 9.5 9.0 8.5 8.0 7.5 7.0 6.5 6.0 5.5 5.0 4.5 4.0 3.5 3.0 2.5 2.0 1.5 1.0 0.5 0.0 -0.5 -1
f1 (ppm)

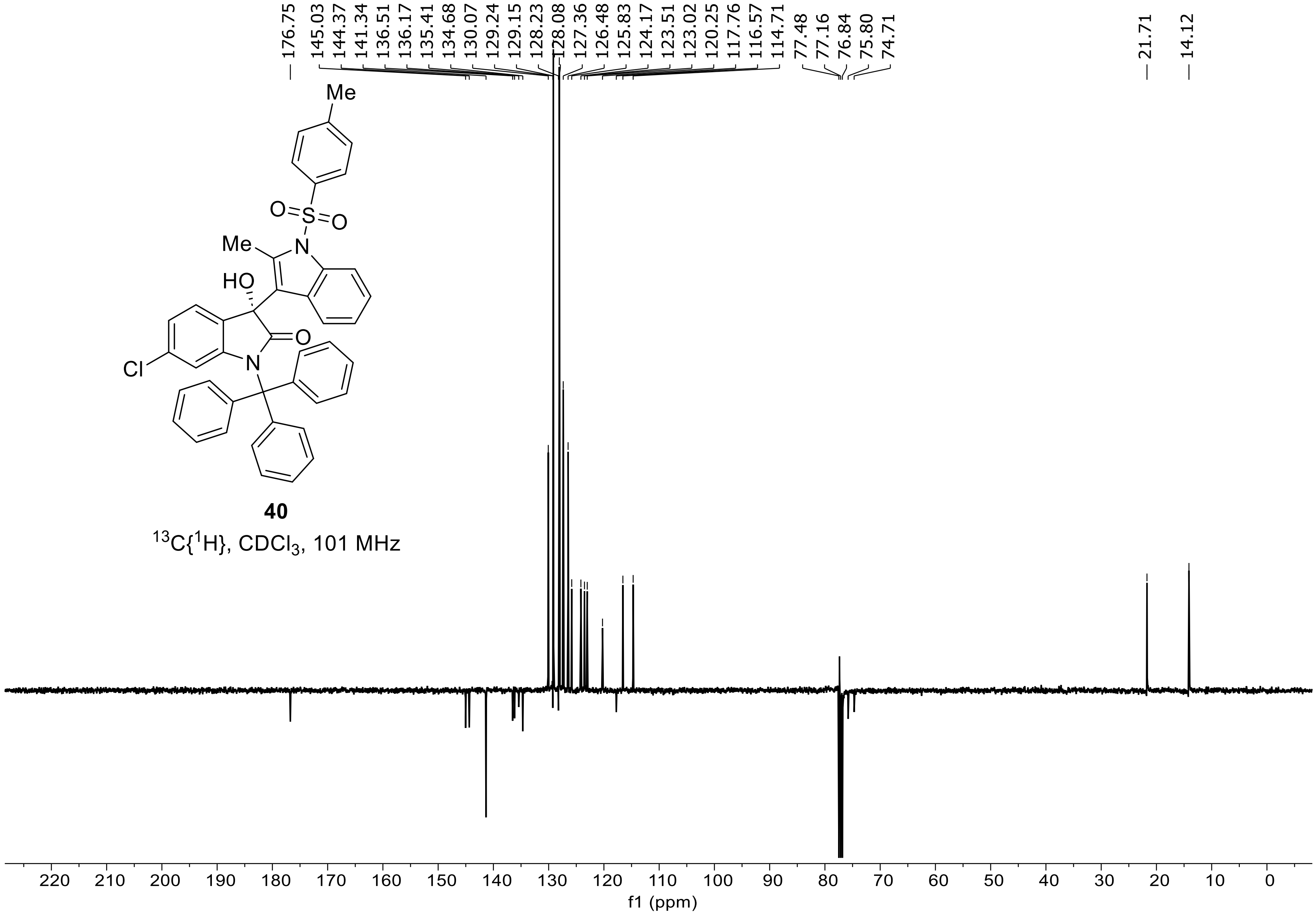
176.75
145.03
144.37
141.34
136.51
136.17
135.41
134.68
130.07
129.24
129.15
128.23
128.08
127.36
126.48
125.83
124.17
123.51
123.02
120.25
117.76
116.57
114.71
77.48
77.16
76.84
75.80
74.71
21.71
14.12
Me
O=S=O
Me
N
HO
O
N
Cl
40
13C{1H}, CDCl3, 101 MHz
220 210 200 190 180 170 160 150 140 130 120 110 100 90 80 70 60 50 40 30 20 10 0
f1 (ppm)

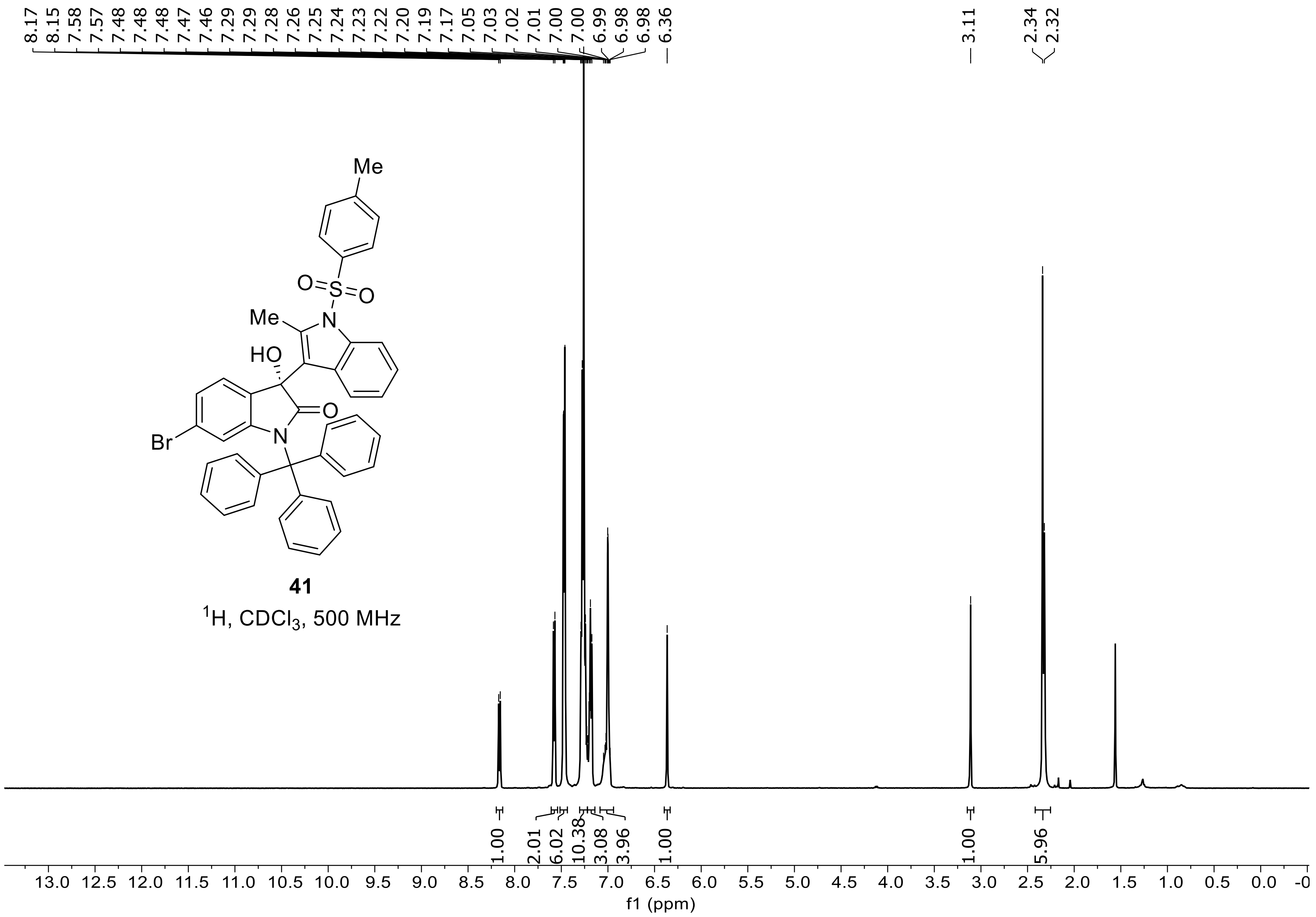
8.17
8.15
7.58
7.57
7.48
7.48
7.48
7.47
7.46
7.29
7.29
7.28
7.26
7.25
7.24
7.23
7.22
7.20
7.19
7.17
7.05
7.03
7.02
7.01
7.00
7.00
6.99
6.98
6.98
6.36
3.11
2.34
2.32
Me
O=S=O
Me
N
HO
O
Br
N
41
^{1}H, CDCl$_3$, 500 MHz
1.00
2.01
6.02
10.38
3.08
3.96
1.00
1.00
5.96
13.0 12.5 12.0 11.5 11.0 10.5 10.0 9.5 9.0 8.5 8.0 7.5 7.0 6.5 6.0 5.5 5.0 4.5 4.0 3.5 3.0 2.5 2.0 1.5 1.0 0.5 0.0 -0
f1 (ppm)

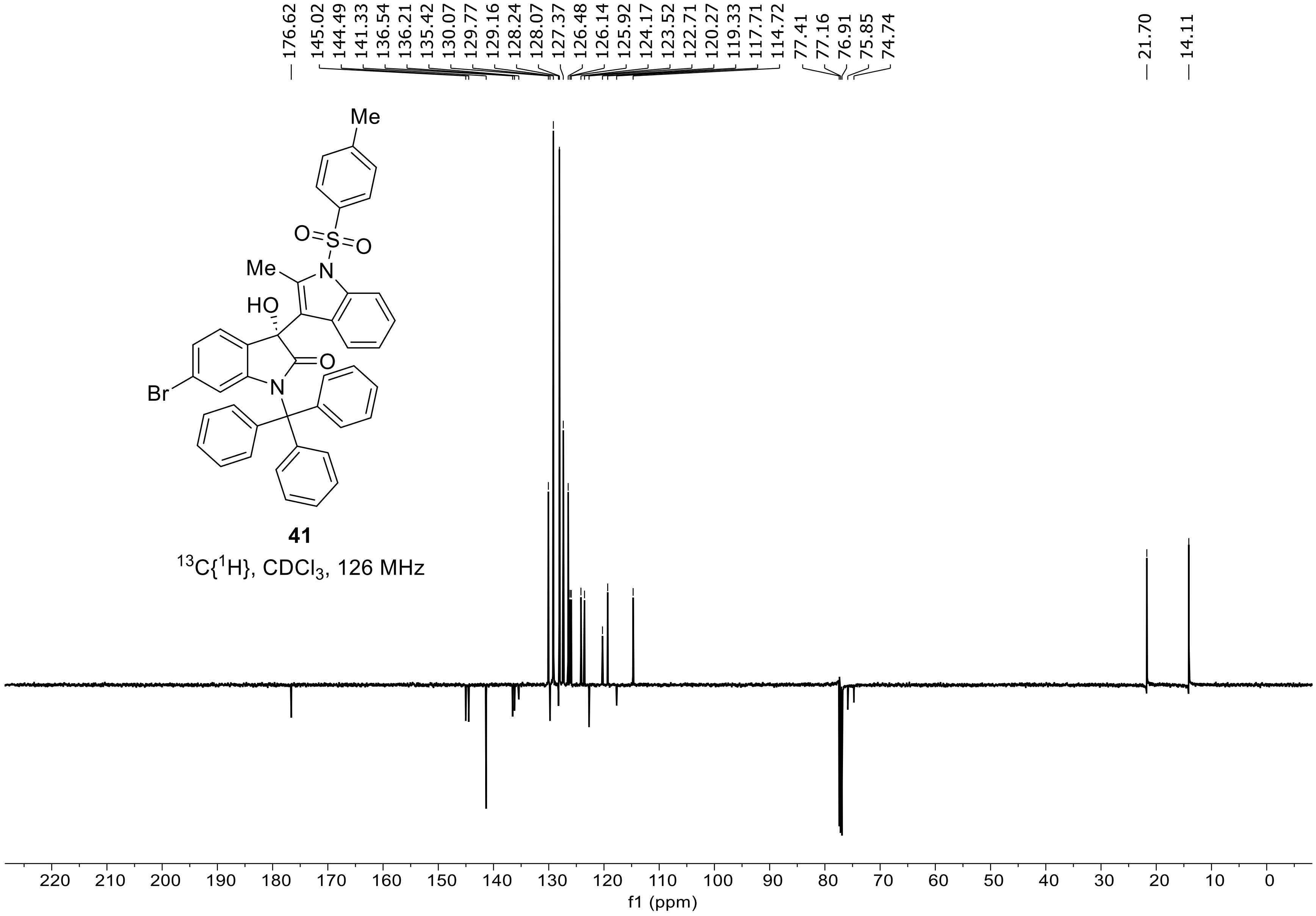
176.62
145.02
144.49
141.33
136.54
136.21
135.42
130.07
129.77
129.16
128.24
128.07
127.37
126.48
126.14
125.92
124.17
123.52
122.71
120.27
119.33
117.71
114.72
77.41
77.16
76.91
75.85
74.74
21.70
14.11
Me
O
S
O
Me
N
HO
O
Br
N
41
$^{13}C\{^{1}H\}$, $CDCl_3$, 126 MHz
220 210 200 190 180 170 160 150 140 130 120 110 100 90 80 70 60 50 40 30 20 10 0
f1 (ppm)

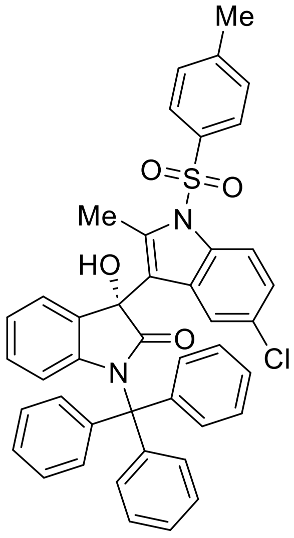

42

^{1}H, $CDCl_3$, 500 MHz

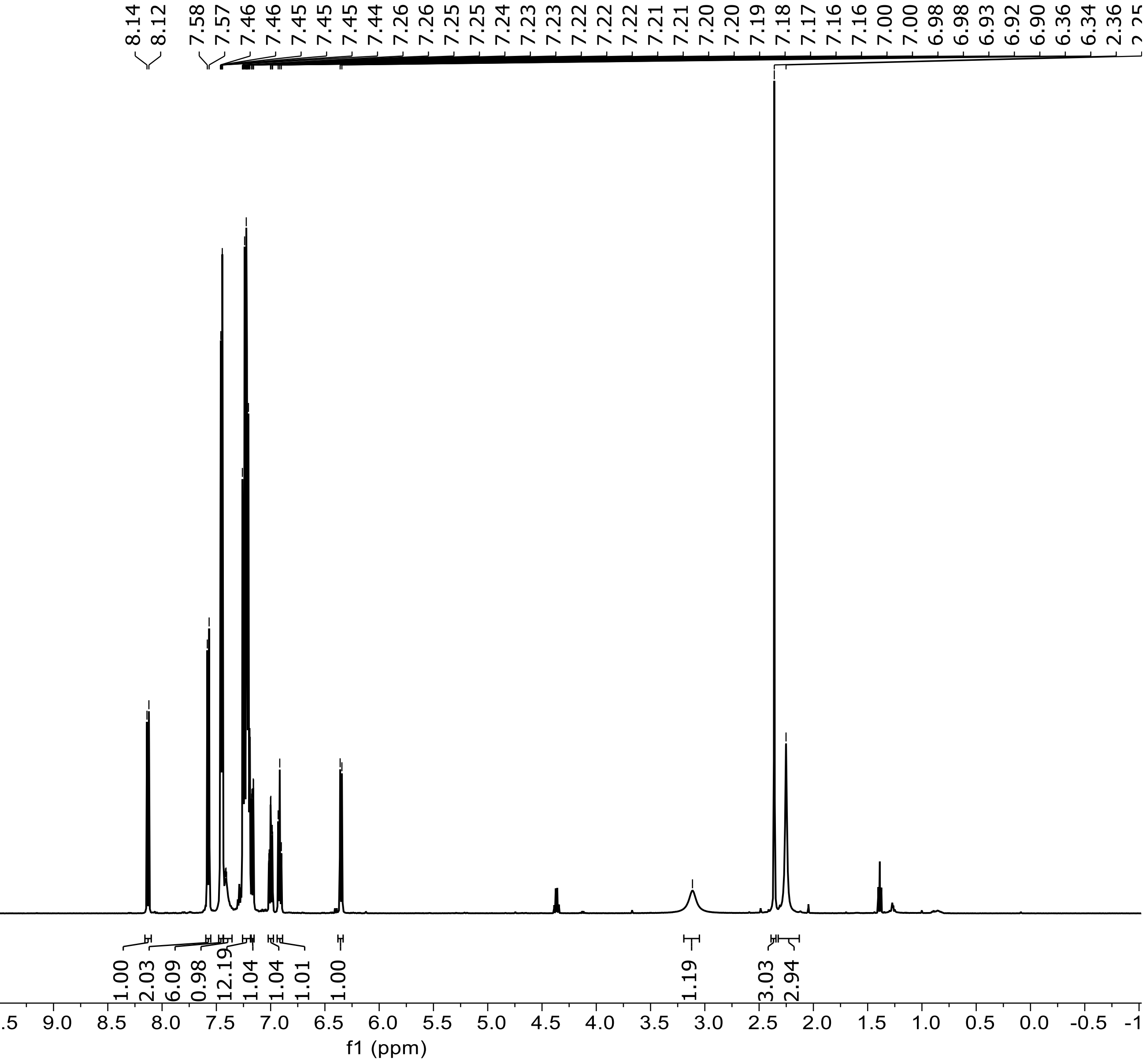

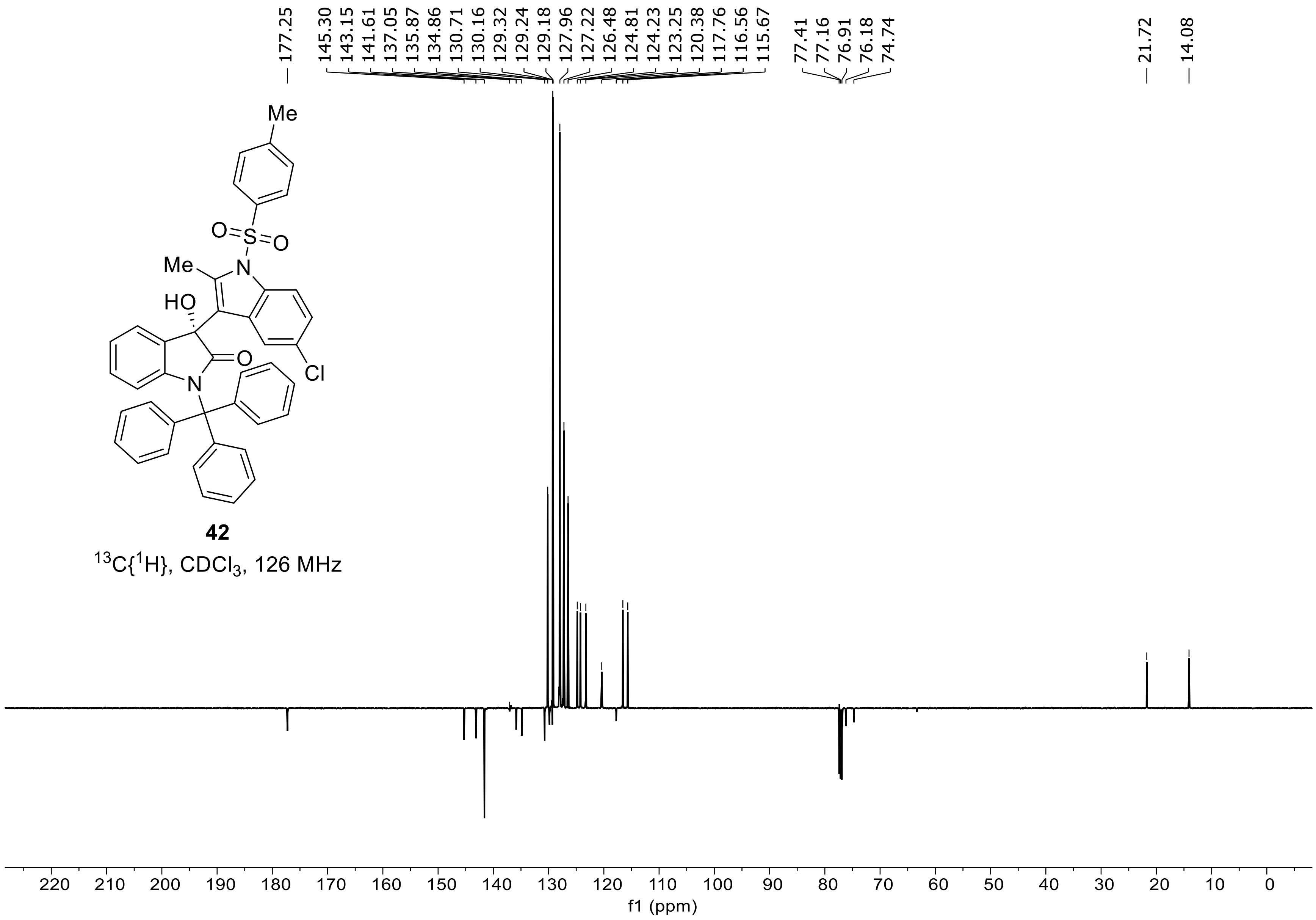
177.25
145.30
143.15
141.61
137.05
135.87
134.86
130.71
130.16
129.32
129.24
129.18
127.96
127.22
126.48
124.81
124.23
123.25
120.38
117.76
116.56
115.67
77.41
77.16
76.91
76.18
74.74
21.72
14.08
Me
O=S=O
Me
N
HO
O
Cl
N
42
13C{1H}, CDCl3, 126 MHz
220 210 200 190 180 170 160 150 140 130 120 110 100 90 80 70 60 50 40 30 20 10 0
f1 (ppm)

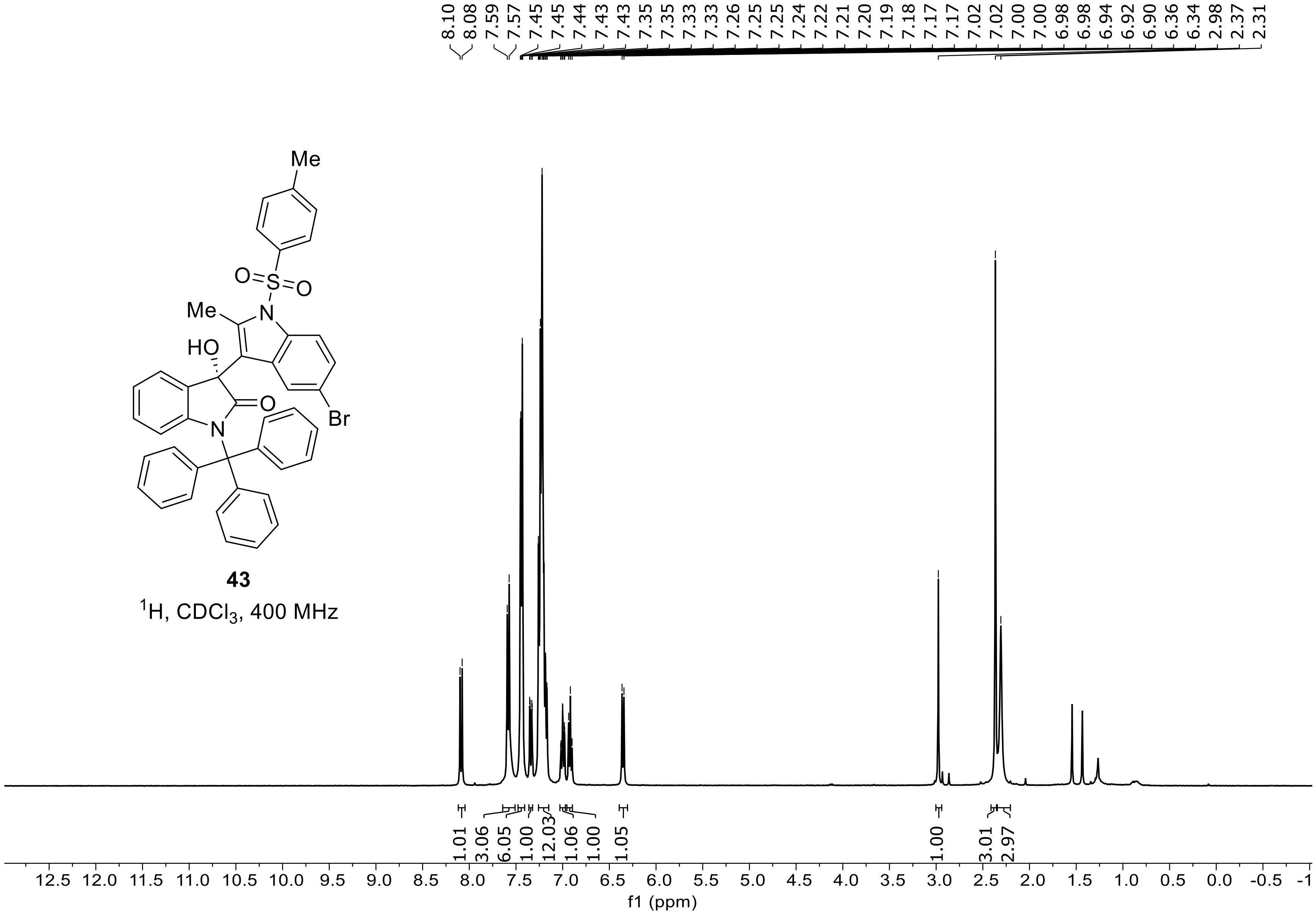

43

^{1}H, $CDCl_3$, 400 MHz

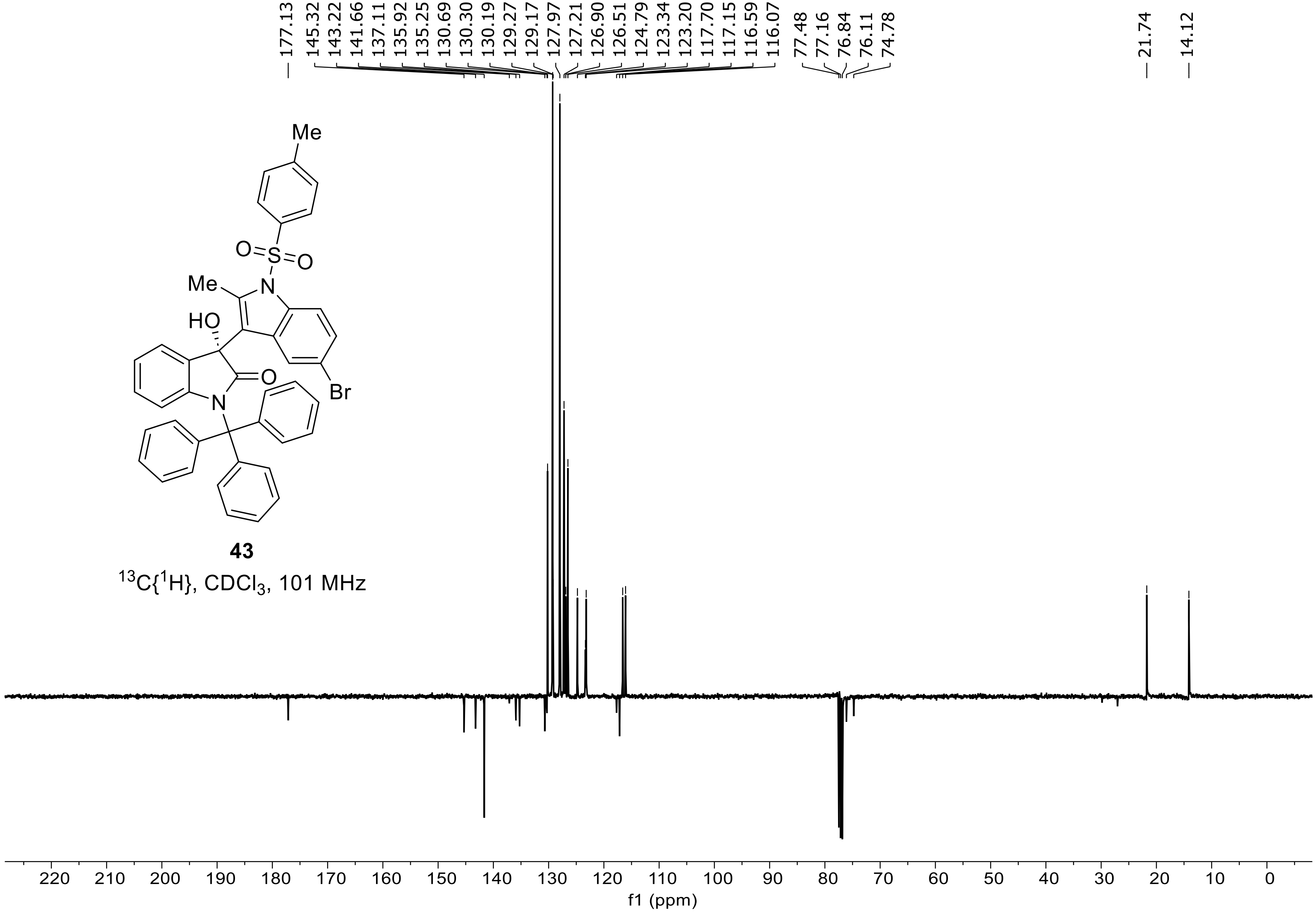
Me
O=S=O
Me
N
HO
O
Br
N
43
$^{13}C\{^1H\}$, $CDCl_3$, 101 MHz
177.13
145.32
143.22
141.66
137.11
135.92
135.25
130.69
130.30
130.19
129.27
129.17
127.97
127.21
126.90
126.51
124.79
123.34
123.20
117.70
117.15
116.59
116.07
77.48
77.16
76.84
76.11
74.78
21.74
14.12
220 210 200 190 180 170 160 150 140 130 120 110 100 90 80 70 60 50 40 30 20 10 0
f1 (ppm)

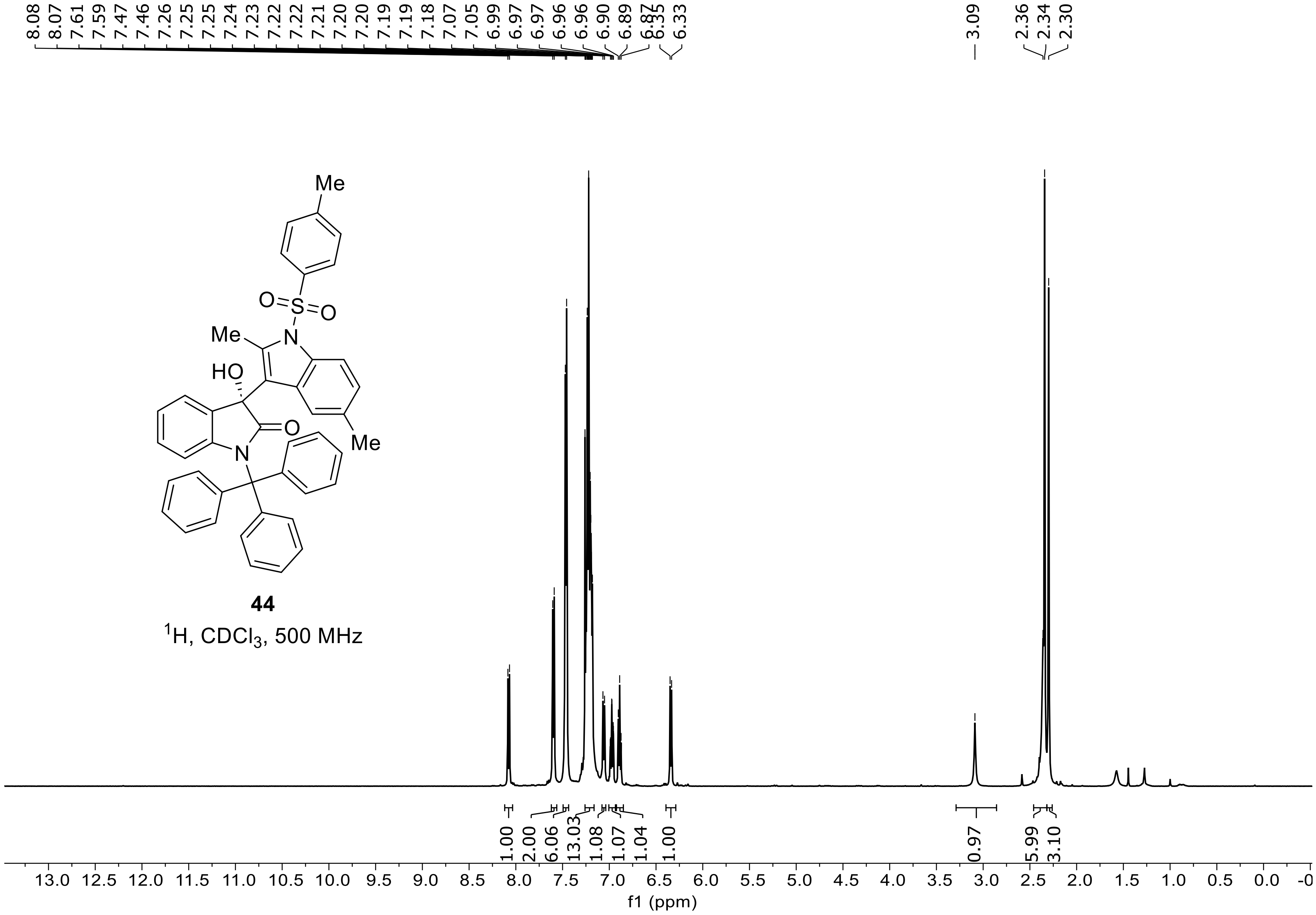

44

^{1}H, $CDCl_3$, 500 MHz

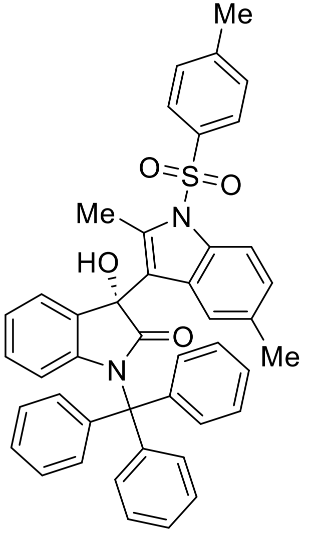

44

$^{13}C\{^{1}H\}$, $CDCl_3$, 126 MHz

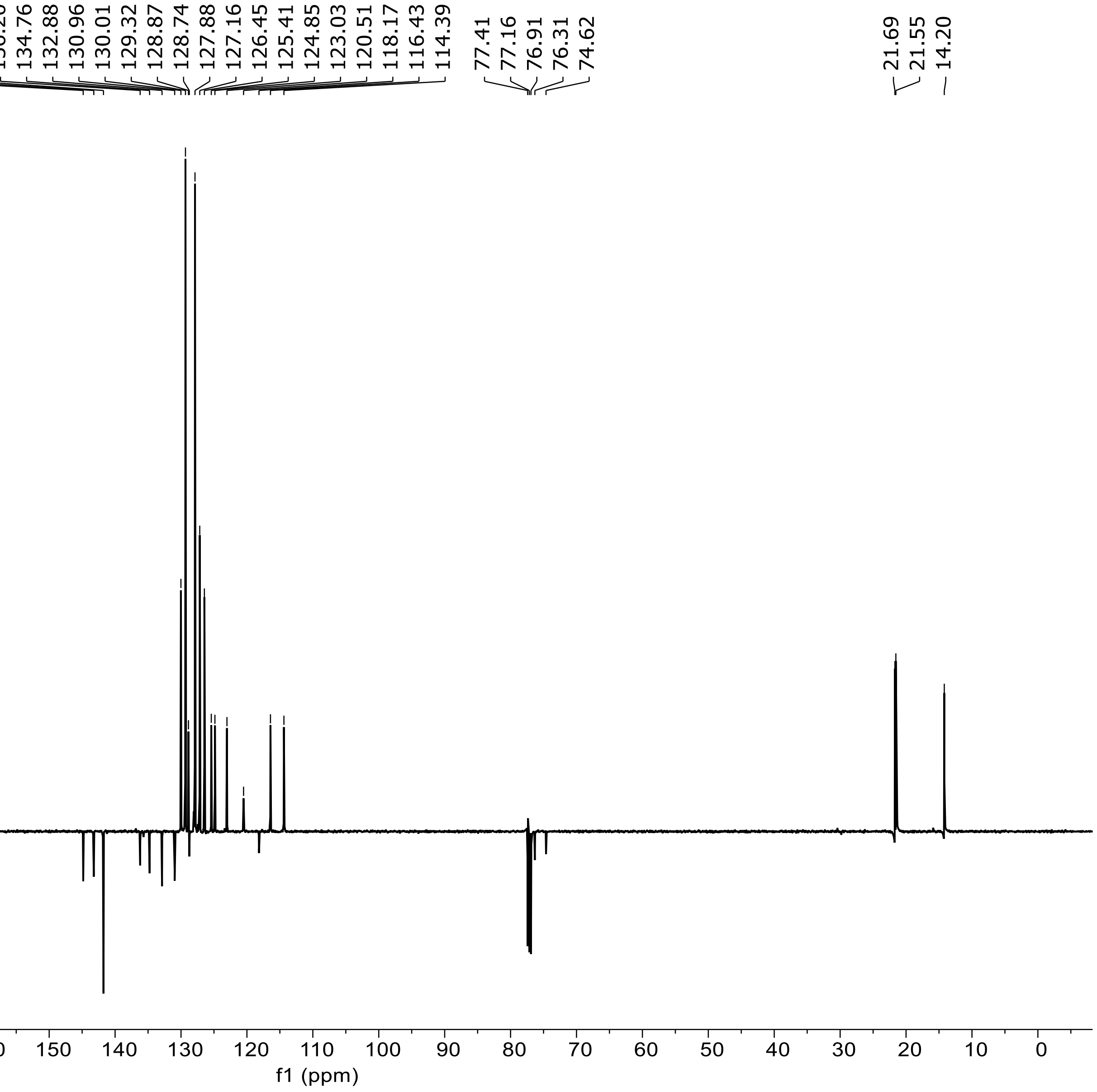

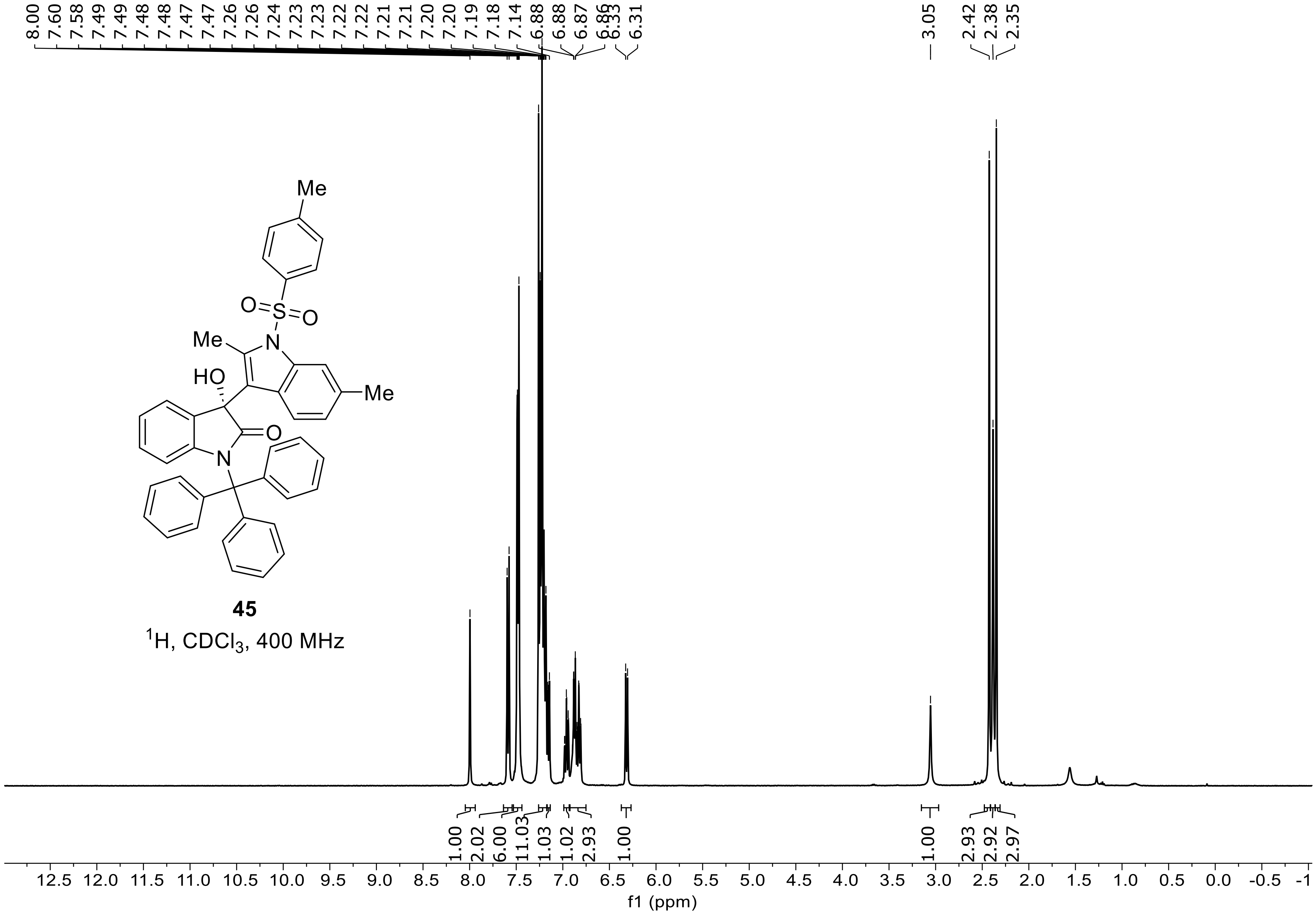

45

^1H, $CDCl_3$, 400 MHz

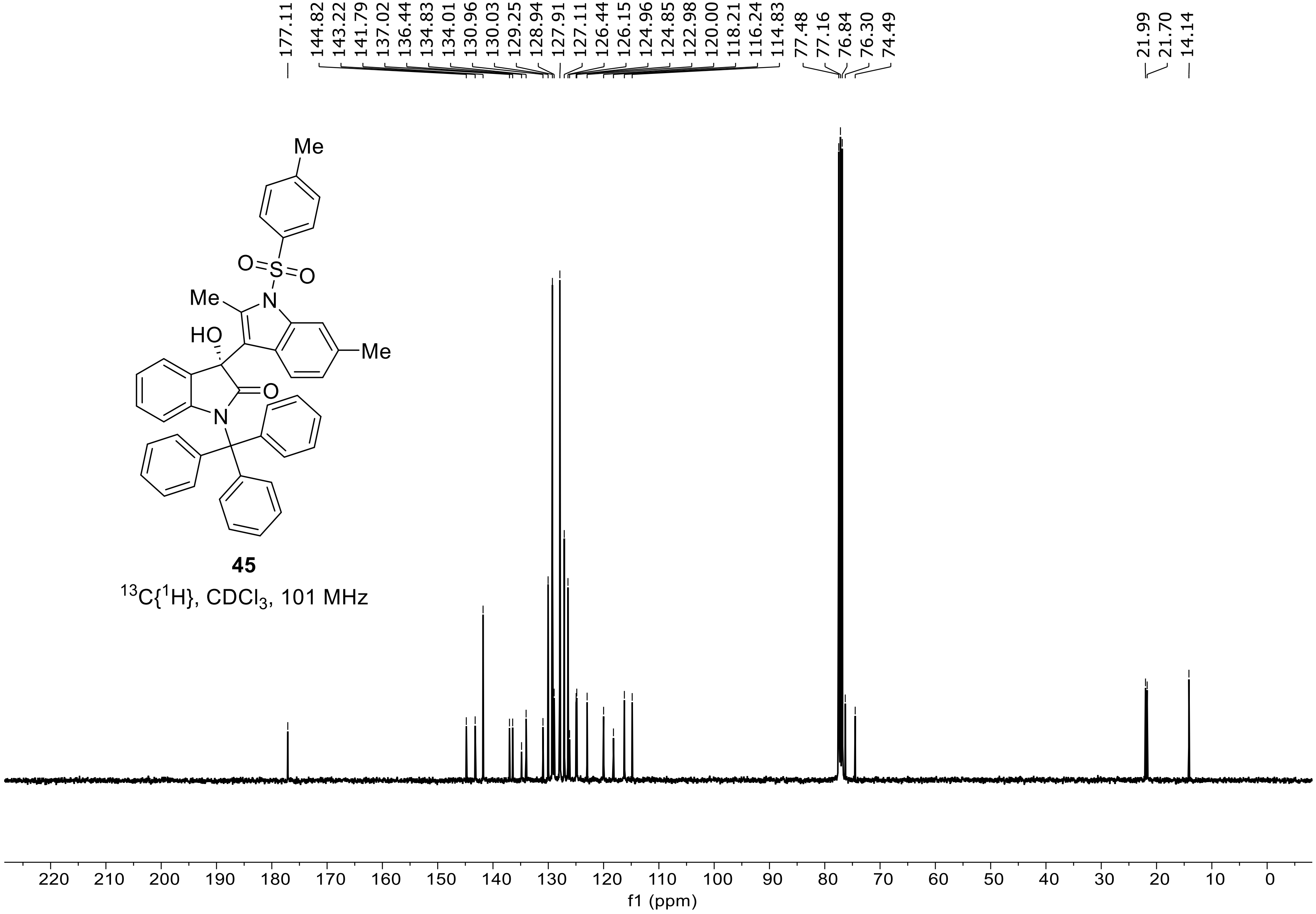

45

$^{13}C\{^{1}H\}$, $CDCl_3$, 101 MHz

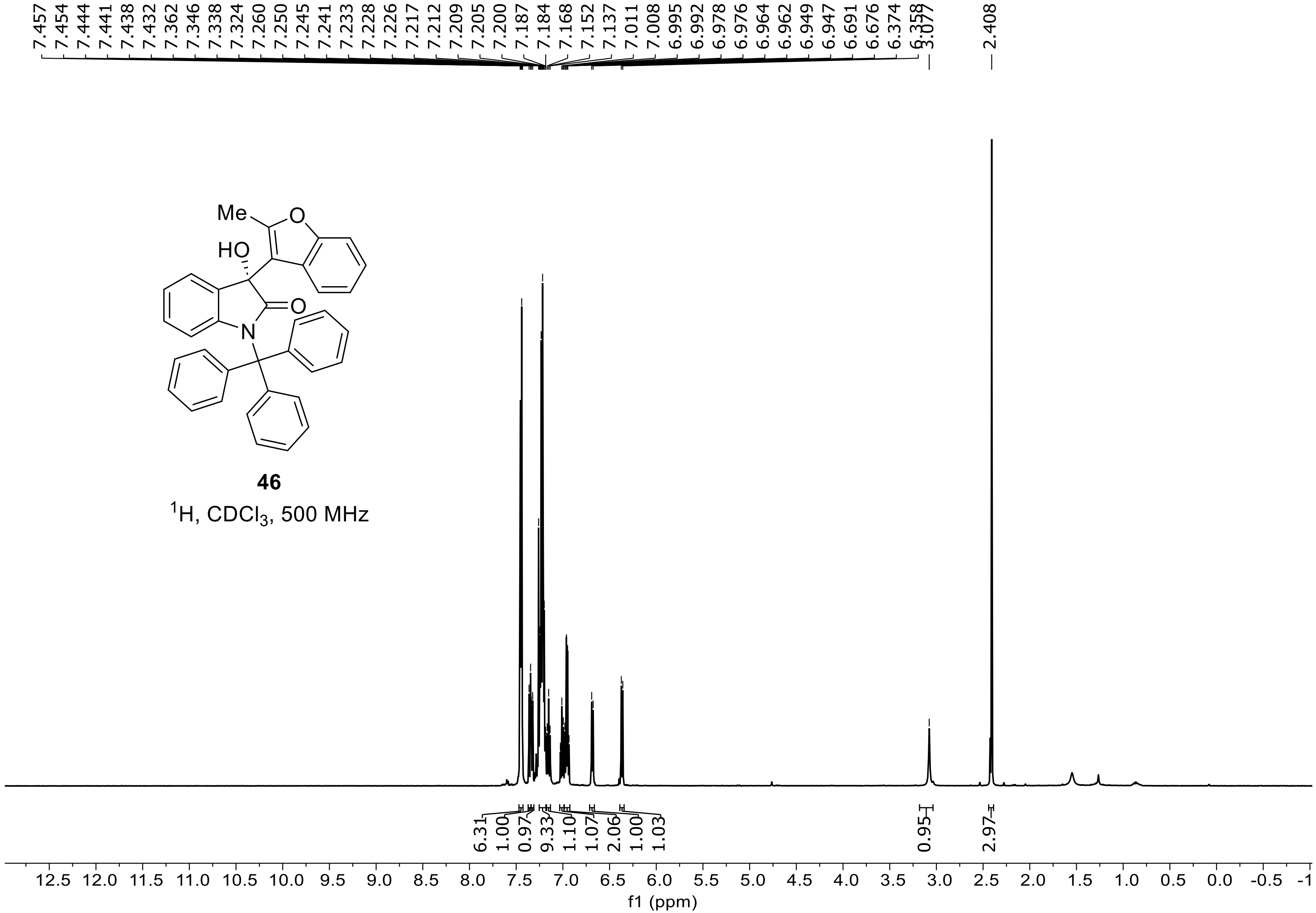
7.457
7.454
7.444
7.441
7.438
7.432
7.362
7.346
7.338
7.324
7.260
7.250
7.245
7.241
7.233
7.228
7.226
7.217
7.212
7.209
7.205
7.200
7.187
7.184
7.168
7.152
7.137
7.011
7.008
6.995
6.992
6.978
6.976
6.964
6.962
6.949
6.947
6.691
6.676
6.374
6.358
3.077
2.408
Me
O
HO
O
N
46
1H, CDCl3, 500 MHz
6.31
1.00
0.97
9.33
1.10
1.07
2.06
1.00
1.03
0.95
2.97
12.5 12.0 11.5 11.0 10.5 10.0 9.5 9.0 8.5 8.0 7.5 7.0 6.5 6.0 5.5 5.0 4.5 4.0 3.5 3.0 2.5 2.0 1.5 1.0 0.5 0.0 -0.5 -1
f1 (ppm)

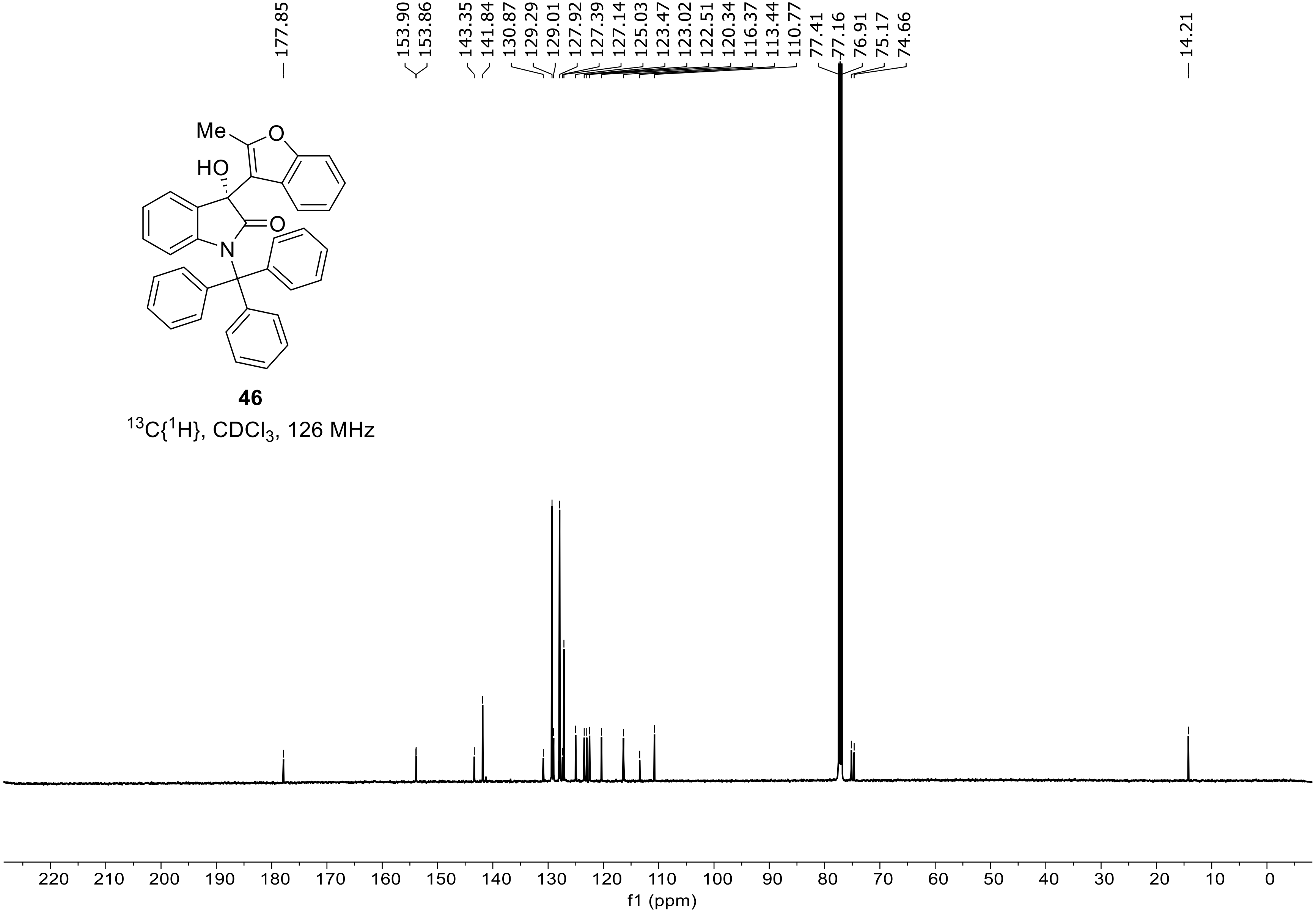
177.85
153.90
153.86
143.35
141.84
130.87
129.29
129.01
127.92
127.39
127.14
125.03
123.47
123.02
122.51
120.34
116.37
113.44
110.77
77.41
77.16
76.91
75.17
74.66
14.21
Me
O
HO
O
N
46
$^{13}C\{^1H\}$, $CDCl_3$, 126 MHz
220 210 200 190 180 170 160 150 140 130 120 110 100 90 80 70 60 50 40 30 20 10 0
f1 (ppm)

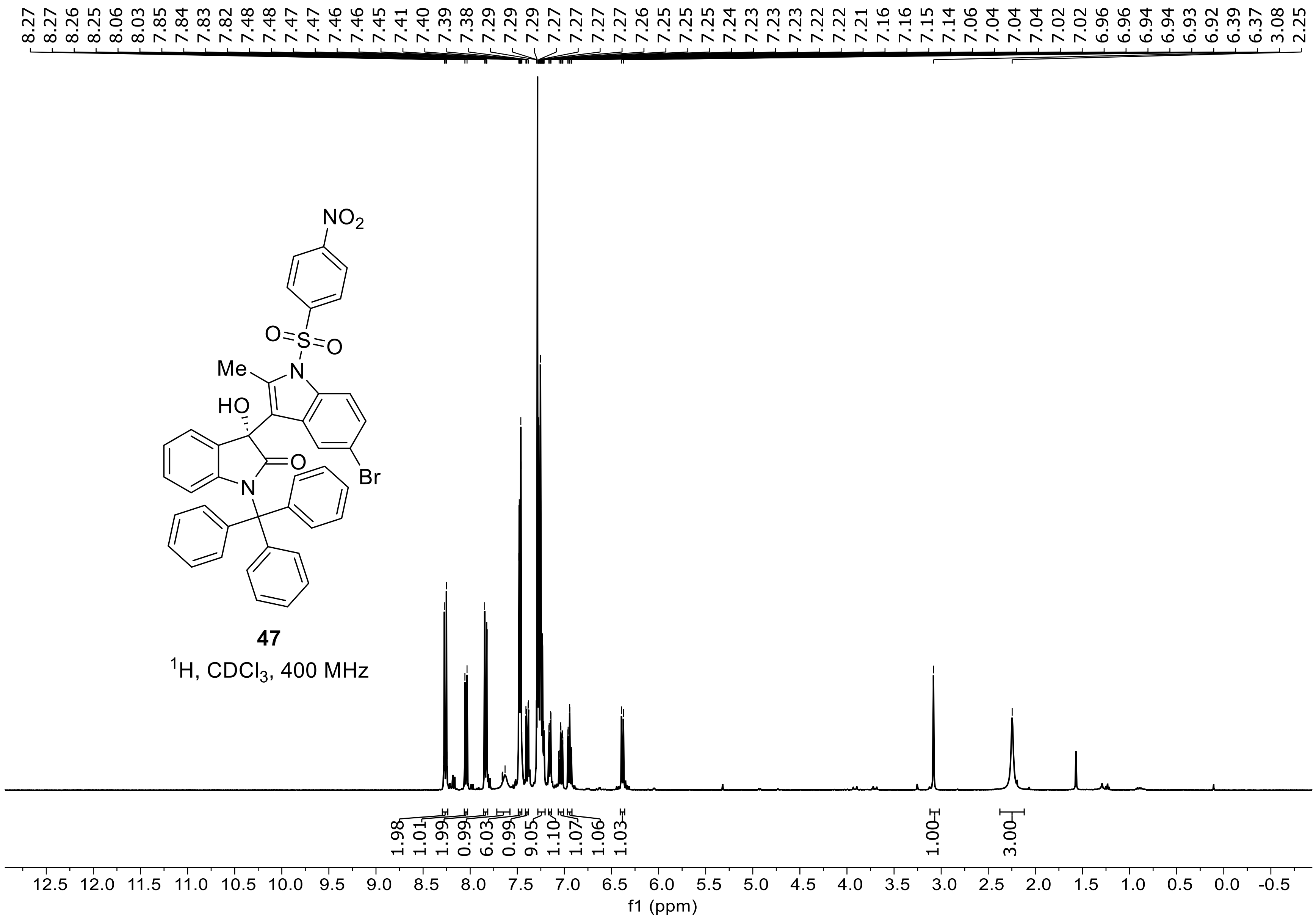

8.27 8.27 8.26 8.25 8.06 8.03 7.85 7.84 7.83 7.82 7.48 7.48 7.47 7.47 7.46 7.46 7.45 7.41 7.40 7.39 7.38 7.29 7.29 7.29 7.27 7.27 7.27 7.27 7.26 7.25 7.25 7.25 7.24 7.23 7.23 7.23 7.22 7.22 7.21 7.16 7.16 7.15 7.14 7.06 7.04 7.04 7.04 7.02 7.02 6.96 6.96 6.94 6.94 6.93 6.92 6.39 6.37 3.08 2.25
NO2
O=S=O
Me
N
HO
O
N
Br
47
1H, CDCl3, 400 MHz
1.98 1.01 1.99 0.99 6.03 0.99 9.05 1.10 1.07 1.06 1.03
1.00
3.00
12.5 12.0 11.5 11.0 10.5 10.0 9.5 9.0 8.5 8.0 7.5 7.0 6.5 6.0 5.5 5.0 4.5 4.0 3.5 3.0 2.5 2.0 1.5 1.0 0.5 0.0 -0.5
f1 (ppm)

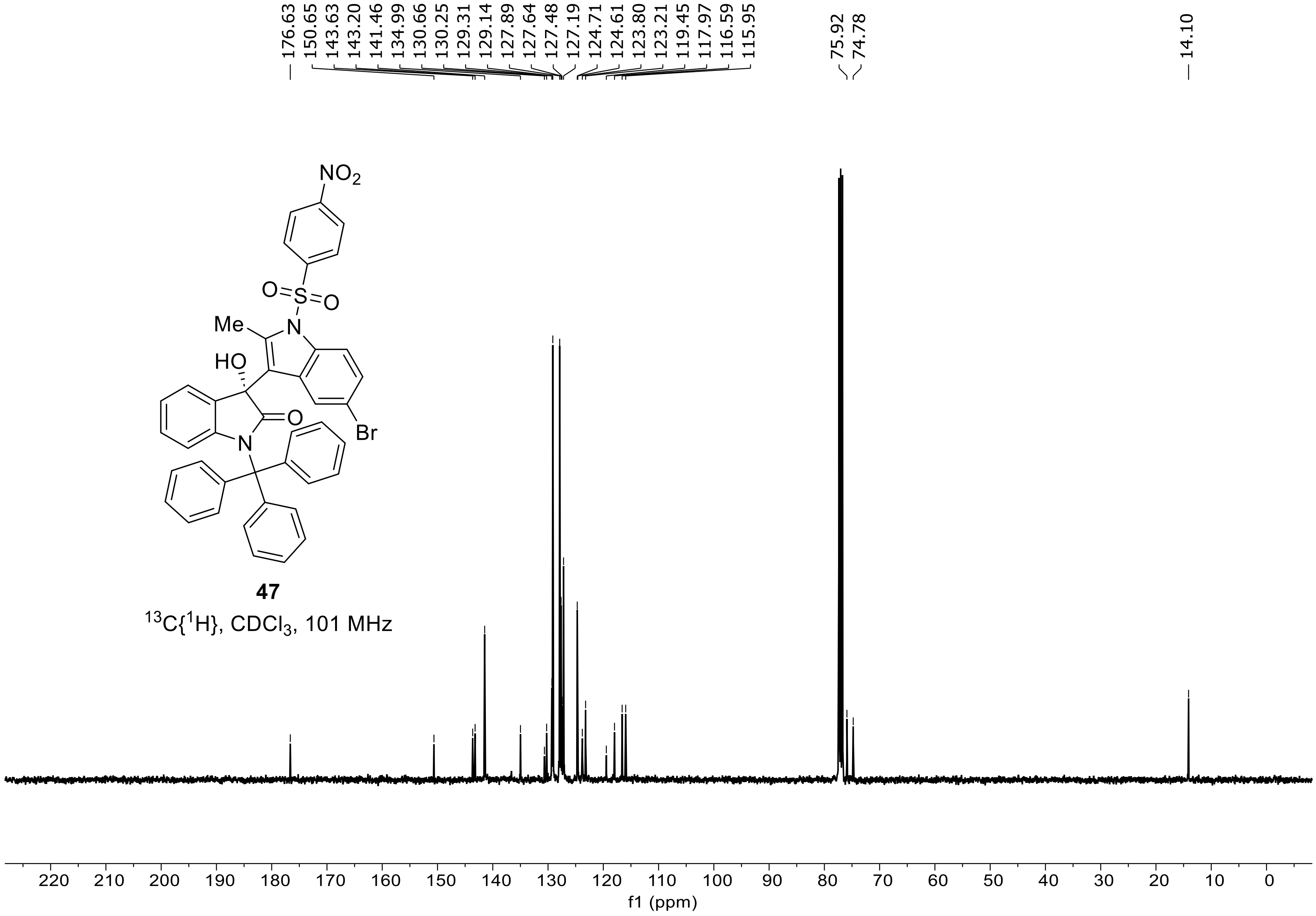
176.63
150.65
143.63
143.20
141.46
134.99
130.66
130.25
129.31
129.14
127.89
127.64
127.48
127.19
124.71
124.61
123.80
123.21
119.45
117.97
116.59
115.95
75.92
74.78
14.10
NO2
O=S=O
Me
N
HO
O
N
Br
47
13C{1H}, CDCl3, 101 MHz
220 210 200 190 180 170 160 150 140 130 120 110 100 90 80 70 60 50 40 30 20 10 0
f1 (ppm)

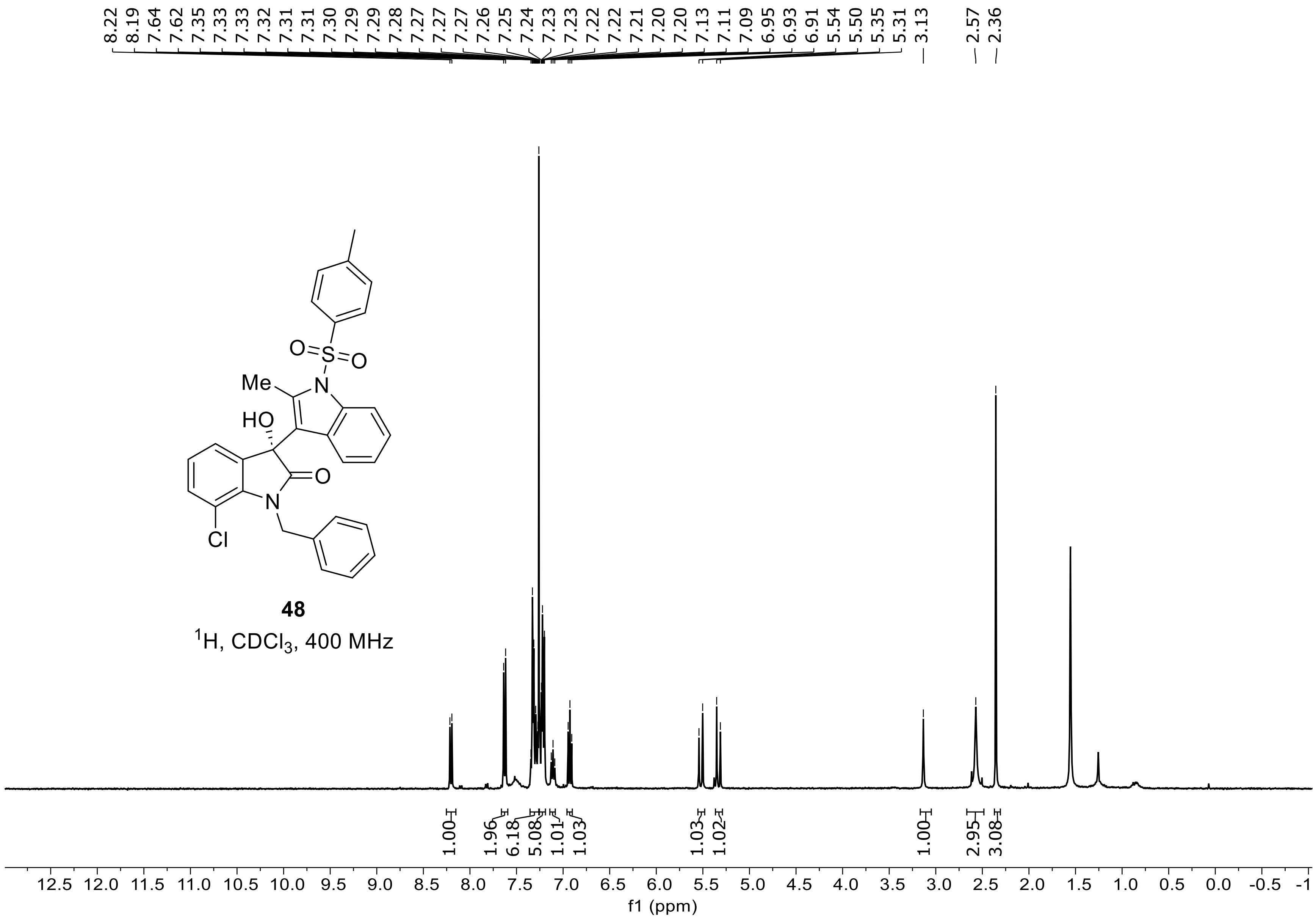
8.22 8.19 7.64 7.62 7.35 7.33 7.33 7.32 7.31 7.31 7.30 7.29 7.29 7.28 7.27 7.27 7.27 7.26 7.25 7.24 7.23 7.23 7.22 7.22 7.21 7.20 7.20 7.13 7.11 7.09 6.95 6.93 6.91 5.54 5.50 5.35 5.31 3.13 2.57 2.36
O=S=O
Me
N
HO
O
N
Cl
48
^{1}H, CDCl$_3$, 400 MHz
1.00 1.96 6.18 5.08 1.01 1.03 1.03 1.02 1.00 2.95 3.08
12.5 12.0 11.5 11.0 10.5 10.0 9.5 9.0 8.5 8.0 7.5 7.0 6.5 6.0 5.5 5.0 4.5 4.0 3.5 3.0 2.5 2.0 1.5 1.0 0.5 0.0 -0.5 -1
f1 (ppm)

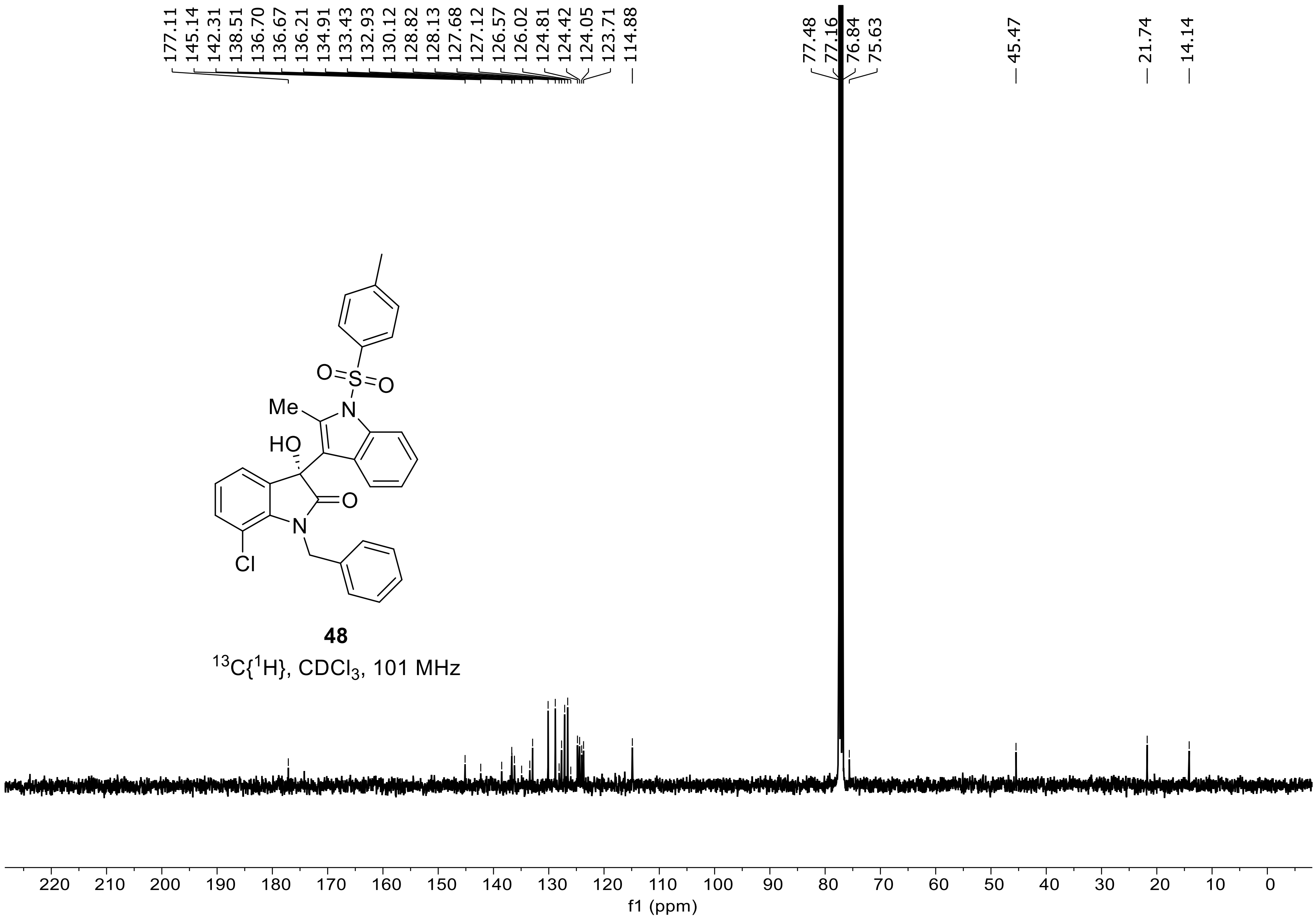

48

$^{13}C\{^{1}H\}$, $CDCl_3$, 101 MHz

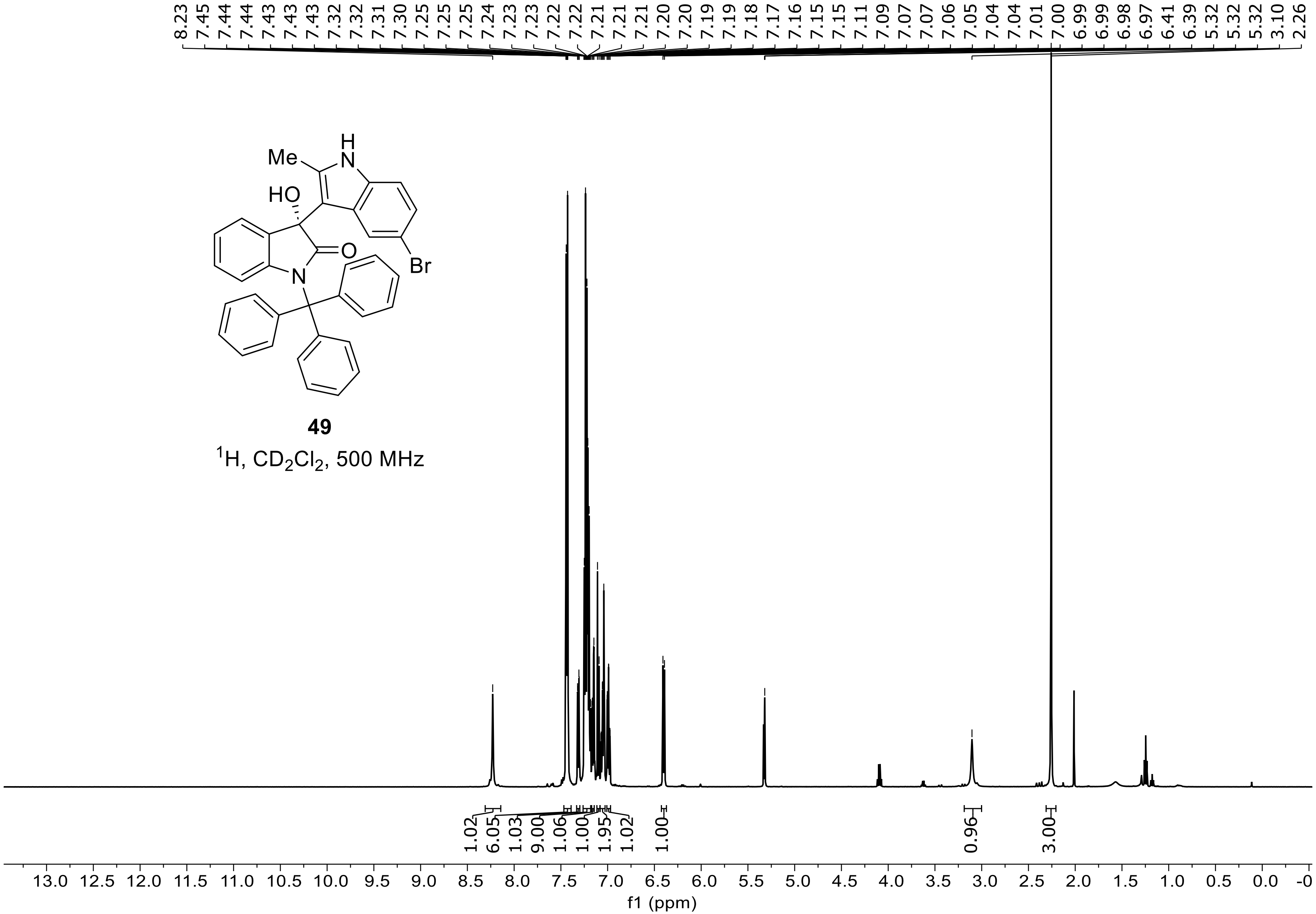

49

^1H, CD_2Cl_2, 500 MHz

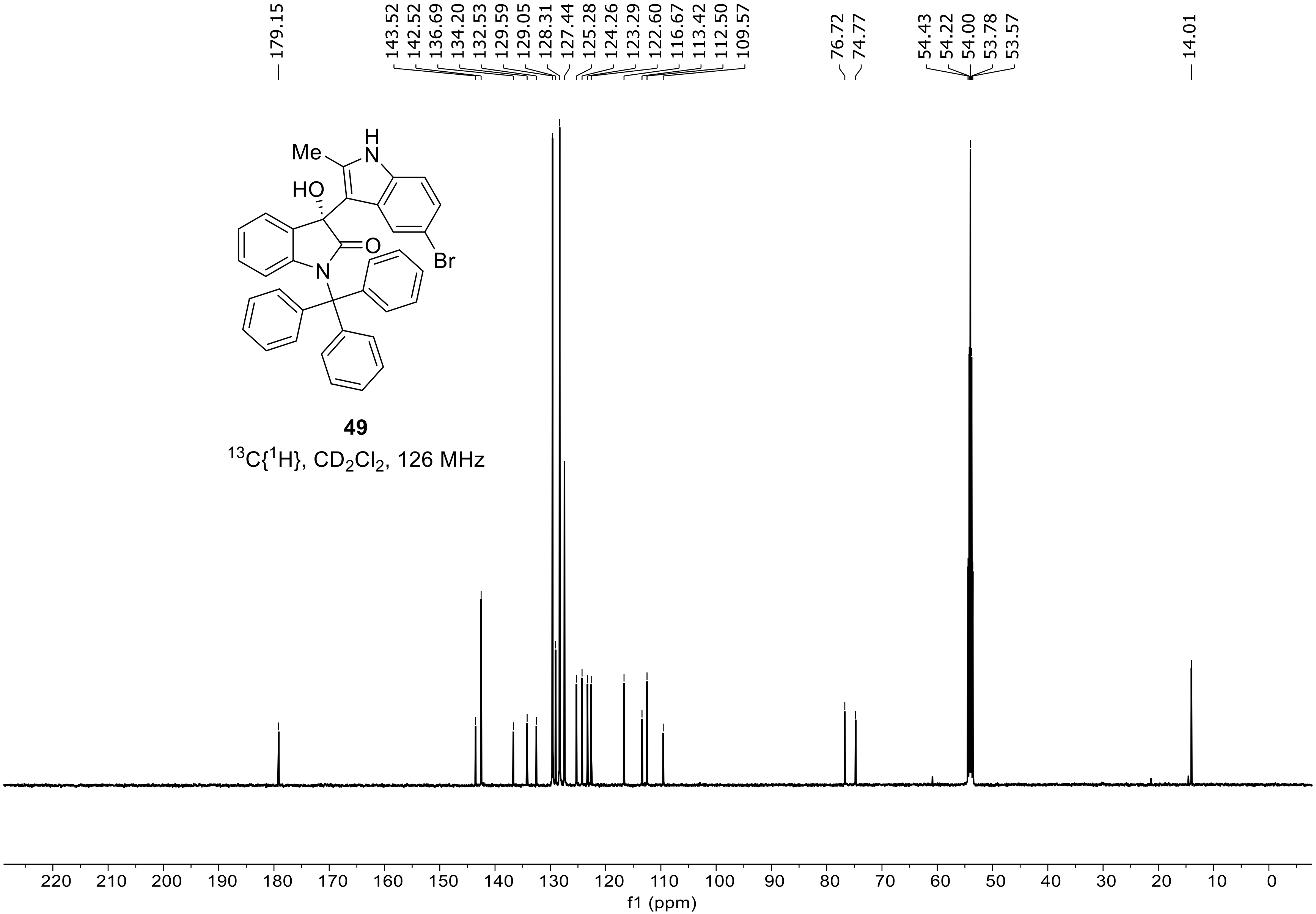
179.15
143.52
142.52
136.69
134.20
132.53
129.59
129.05
128.31
127.44
125.28
124.26
123.29
122.60
116.67
113.42
112.50
109.57
76.72
74.77
54.43
54.22
54.00
53.78
53.57
14.01
Me
H
N
HO
O
N
Br
49
$^{13}C\{^1H\}$, CD_2Cl_2, 126 MHz
220 210 200 190 180 170 160 150 140 130 120 110 100 90 80 70 60 50 40 30 20 10 0
f1 (ppm)

10. Computational raw data

Table S2 Relative energies and distances of the conformers computed with DFT (also available as .csv). RMSD and distances in Å, with relative energies in kcal/mol, relative to SR_0070, the lowest energy conformer.

filename	RMSD	RMSD_TS	xtbCO	xtbCC	dftCO	dftCC	rel_xTB	rel_DFT_xTB	rel_DFT	rel_DFT_DFT	rel_G	rel_sub	rel_BIMP
RR_0002	0.31	0.15	2.25	2.24	2.06	2.80	-0.70	5.79	2.04	4.05	3.44	8.85	-5.11
RR_0010	0.35	0.11	2.18	2.19	2.07	2.66	0.86	0.21	-1.75	0.74	1.67	5.21	-5.42
RR_0019	1.95	0.44	2.10	2.16	2.04	2.55	1.62	3.71	2.88	5.41	5.28	4.22	-4.14
RR_0070	0.33	0.15	2.10	2.15	2.04	2.54	1.68	3.15	3.11	5.50	4.48	4.27	-4.46
RR_0081	0.45	0.23	2.18	2.17	2.06	2.67	2.50	6.01	3.54	4.75	5.20	3.64	-1.14
RS_0015	0.30	0.10	2.21	2.31	2.05	2.72	2.84	-0.19	0.27	2.04	1.82	4.49	-2.21
RS_0043	0.24	0.13	2.20	2.29	2.07	2.71	2.19	-0.69	1.48	2.73	2.55	4.77	-2.94
RS_0103	0.17	0.15	2.21	2.29	2.08	2.72	1.46	-1.48	0.60	3.43	3.37	6.40	-3.52
RS_0111	0.26	0.14	2.22	2.27	2.07	2.71	2.24	0.33	1.58	2.77	2.93	4.76	-3.33
RS_0238	0.30	0.19	2.30	2.22	2.07	2.72	4.67	0.62	1.33	2.56	5.16	-2.32	2.72
SR_0053	0.25	0.14	2.23	2.20	2.08	2.61	-1.04	-2.96	0.66	1.42	1.39	0.55	-2.11
SR_0070	0.37	0.21	2.25	2.23	2.06	2.66	0.00	0.00	0.00	0.00	0.00	0.00	0.00
SR_0101	0.25	0.12	2.28	2.22	2.08	2.68	-0.44	-3.31	-0.24	1.14	1.70	-0.07	-0.79
SR_0121	0.24	0.13	2.28	2.22	2.08	2.68	-0.44	-3.28	-0.23	1.18	1.62	0.00	-0.91
SR_0180	3.63	0.98	2.54	2.02	2.00	2.70	-0.05	-2.80	4.55	4.71	4.00	0.34	0.62
SS_0000	0.34	0.22	2.26	2.19	2.07	2.73	-3.11	0.08	-0.56	3.92	3.36	10.79	-6.27
SS_0001	2.64	0.82	2.26	2.19	2.05	2.73	-3.06	0.20	-0.72	3.26	2.53	9.45	-6.63
SS_0016	0.53	0.16	2.24	2.24	2.07	2.72	-0.96	1.22	-1.38	2.55	3.73	10.84	-1.92
SS_0210	0.69	0.16	2.33	2.25	2.12	2.97	0.76	2.22	1.01	5.22	4.91	1.80	-3.48
SS_0388	0.29	0.17	2.18	2.21	2.01	2.67	1.57	-0.38	2.94	4.71	5.07	8.62	-5.35

RR_0002_TS
Frequencies, energies and thermodynamic properties:
Lowest Vibrational Mode (1/cm) = -334.76
2nd Lowest Vibrational Mode (1/cm) = 8.32
3rd Lowest Vibrational Mode (1/cm) = 11.55
4th Lowest Vibrational Mode (1/cm) = 12.19
CREST Electronic Energy (a.u.) = -286.5632529
xTB Electronic Energy (a.u.) = -286.56413629
DFT//xTB Electronic Energy (a.u.) = -5585.41941118
DFT Electronic Energy (a.u.) = -5585.472772466288
DFT//DFT Electronic Energy (a.u.) = -5590.871129509165
Gibbs Free Energy (a.u.) = -5589.613241
Substrate Energy (a.u.) = -2466.955566751594
BIMP Energy (a.u.) = -3123.684509152896
DFT optimised cartesian coordinates:

O	1.84143218175451	0.22597069119384	-1.34184081541447
C	2.81141415977895	0.90660958677385	-0.98543477422560
N	4.17099111730801	0.66224540722492	-1.29382229880674
C	4.66845498746262	-0.30929047933978	-2.29247406840138
C	4.92342157725962	1.79924066160586	-0.92018142061601
C	6.26247251582704	2.10785542162005	-1.14731094662307
C	6.76474637827806	3.32047402994481	-0.65279849815355
C	5.94864380170040	4.21324132643418	0.04899106483460
C	4.59821610391929	3.92098879327294	0.25330049487123
C	4.08715355225216	2.71799799082681	-0.23871118055945
C	2.74913145464483	2.17815961622937	-0.27245209266717
O	1.65524652984896	2.72076532511781	0.09601488468164
C	1.13528649152124	2.27600241439736	2.03493716298465
C	1.81360550862038	1.06749429763648	2.18786195109253
C	3.19780898584253	0.92332237376659	2.18460856371772
C	3.51533217681196	-0.47454175718968	2.21148672717949
C	2.29936953731799	-1.20195849885331	2.13547194417434
C	2.28701856029007	-2.60014317474419	2.15051165339379
C	3.51390221916323	-3.26004990100044	2.24000567636788
C	4.72411557082976	-2.55652502091638	2.33217798215307
C	4.73102017806746	-1.16627382417874	2.32707666705735
H	6.91896644327639	1.43584777520606	-1.69650482121725
H	7.81341281839009	3.56625734695702	-0.82743262112230
H	6.36655250021266	5.14940324314903	0.42349827703891
H	3.93846488548263	4.62072897660654	0.77143320138364
H	1.36095190118620	-3.16534658706815	2.08880736697039
H	3.52221984962536	-4.35156213587119	2.24566202119092
H	5.66448297313909	-3.10451793622188	2.41218994281259
H	5.66588383366669	-0.61024306521838	2.41951258887340
H	1.64826446080071	3.17078879286871	2.38214974917318
H	0.05220507399595	2.32213757244275	1.96214134714583
H	3.89760842532625	1.74766505096544	2.29525441529345
N	1.26121831843982	-0.24976113851356	2.09085103284882
S	-0.37029861517348	-0.58045657170552	2.01936801841260
O	-0.47716289861738	-2.00177238834619	1.72259610196235
O	-0.96088173548605	0.37926725715637	1.08866633145382
C	3.69209362157996	-1.50080560418226	-2.42423894130907
C	3.37316350116124	-2.22573470550710	-1.26525268047649
C	3.23052824385926	-1.96661882448803	-3.65360912253296
C	2.58384817493254	-3.36509140656457	-1.33646507803951
H	3.74708733342806	-1.88492445592094	-0.29506916557782
C	2.42921262529887	-3.11488649939632	-3.72579645404013
H	3.48391990518676	-1.43874173148832	-4.57337761296042
C	2.09741954482582	-3.81487462397926	-2.57096945528941
H	2.35471416272498	-3.90794324158465	-0.41730123182432
H	2.07182304552388	-3.45786916912242	-4.69879423543227
H	1.47466804047353	-4.71045025299059	-2.62447149801803
C	5.96384520285949	-1.00359090121239	-1.82397033598795
C	6.43030460319450	-0.90789097379617	-0.51318622030768
C	6.61643397751026	-1.88435914101879	-2.70021123184278
C	7.53906124767235	-1.64576692934805	-0.09010075559787
H	5.91922881368191	-0.25591022889048	0.19127288269366
C	7.72731485756878	-2.61408454019526	-2.28601481183974
H	6.23824994206159	-2.01448784278708	-3.71671724819928
C	8.19756972073403	-2.49536772641069	-0.97522433496204
H	7.88163481086051	-1.55244405068987	0.94264726935405
H	8.22126971598762	-3.28861521194529	-2.98743818052415
H	9.06516869621129	-3.07037831745930	-0.64739322227670
C	4.82983032633701	0.47957483947483	-3.60845018329644
C	3.67861152857055	1.10398986510597	-4.11150532788902
C	6.04048296086202	0.69080322133710	-4.27162894242212
C	3.72588190799931	1.89034812705730	-5.25775150813937
H	2.73199549047456	0.95573537148275	-3.58997207968207
C	6.09344004454387	1.48564067839435	-5.42232623734506
H	6.96468091690006	0.25277221122228	-3.89528051389374
C	4.94016732087094	2.08185728887634	-5.92369220582625
H	2.81227015049257	2.35804481975193	-5.63010894460081
H	7.05168956259587	1.63996733265084	-5.92129035286048
H	4.98499243899762	2.70031062177126	-6.82166856604406
C	-1.04288636745768	-0.29007697375413	3.62173485599824
C	-0.94027038410094	-1.31388907594681	4.56420366714303
C	-1.71903868776624	0.89864167993187	3.89716008399432
C	-1.54035988605207	-1.14063910400048	5.80688951585125
H	-0.41609595263601	-2.23970015117054	4.31988335246151
C	-2.29550647761949	1.05671666116909	5.15408218439153
H	-1.81443382805965	1.67691352357647	3.13727518918903
C	-2.23356072349327	0.03858627991276	6.11523321333422
H	-1.47743613848117	-1.93858015406322	6.54930269240207
H	-2.81775491913259	1.98538548413925	5.38900230124535

C	-2.93916211961171	0.19469589318334	7.43275518628791
H	-2.48407987633306	-0.43443479862054	8.20837912951985
H	-3.99145285528662	-0.11289120452474	7.32313220198243
H	-2.93122078553357	1.24070878821602	7.76636625482901
H	0.05273611459042	2.47032623806897	-0.66301025648756
N	-0.96778319098200	2.63765976163757	-0.73910878733229
C	-1.69566205886765	1.81943697548774	-1.54076896347972
C	-1.40705742597141	3.56984844952666	0.20665889477761
N	-0.95278938019781	0.89674174712760	-2.17562413856189
S	-3.37085580139342	1.99154570764193	-1.77977551133146
C	-0.56238203029550	4.65408897091143	0.47132910383736
C	-2.57326497418183	3.41489403883359	0.97146202802719
C	-1.45500932215620	-0.15885999307463	-3.04309883047595
H	0.03843708063677	0.81840391813193	-1.90512463230351
C	-0.87116987191139	5.55488790562626	1.48815477924732
H	0.36348782955355	4.75363787731834	-0.09676388189864
C	-2.87299252599670	4.34471682420749	1.96124043225968
H	-3.22485106250661	2.56244625898318	0.79650345058079
C	-1.01548438148538	-1.49098512130270	-2.42676235047706
H	-2.55180923153502	-0.12217212624245	-2.98101265388300
C	-1.06661988271622	0.04548011411359	-4.52783611161490
C	0.11140689754108	6.65627734035046	1.77966357435046
C	-2.03358889086898	5.42503282952514	2.23890226134495
C	-4.09014641638433	4.15392812477401	2.82183589292254
N	-1.54810666679606	-1.59234410603292	-1.07392851320911
H	0.08547616649495	-1.57157956484255	-2.39707922496938
H	-1.39185992298516	-2.33294929137501	-3.02279573816792
C	-1.84325315641117	1.25141639268931	-5.06749440783870
C	0.43249039763427	0.31104918537450	-4.66750314296599
C	-1.45927259518299	-1.20138768348256	-5.32932378797893
F	0.42853777468678	7.34494450393940	0.68117722270216
F	-0.35645321185213	7.52471789765900	2.67567330458588
F	1.25880885065901	6.16461356347663	2.26627934992064
H	-2.27834393593446	6.14023870368918	3.02476148293047
F	-4.73124075277700	5.30043032026401	3.04442255147327
F	-4.96680315724371	3.29876645135839	2.28886808026445
F	-3.76030851567805	3.66197978080770	4.02965193988646
H	-1.25326290183924	-0.87023629048342	-0.41616548389021
P	-2.68359960174910	-2.66994140604280	-0.58047545595908
H	-1.64112833342231	2.15170872576927	-4.46801682807375
H	-2.92794037507327	1.06490261255246	-5.03921403382618
H	-1.54948663547452	1.45485078409681	-6.10865403942062
H	0.71436651559150	0.36961214298740	-5.73047141641612
H	1.03484655911301	-0.48328338009492	-4.19999270680316
H	0.69744001046248	1.26893032549955	-4.19451627645701
H	-1.38511419334436	-0.99119781248695	-6.40660246996778
H	-2.49825544296028	-1.50118770929168	-5.11069260222722
H	-0.79877358918618	-2.05391285800301	-5.11017087850909
C	-3.87103273939116	-2.84881790281803	-1.92851480330119
C	-1.97485832617815	-4.28455851505114	-0.23883853830929
C	-3.43211724470497	-2.00397928617305	0.91412785587289
C	-3.88056418268383	-3.97181944551665	-2.77332063433684
C	-4.70923181513086	-1.76687618989909	-2.22753511240317
C	-0.58013717944295	-4.39754070853273	-0.13423529529812
C	-2.77611162125130	-5.41441483900701	-0.03397065645786
C	-3.83236590380168	-0.66328573037837	0.96415381903377
C	-3.58558908376564	-2.80874097369782	2.05602786927490
C	-4.72545204513135	-4.01591432912817	-3.87140674363638
H	-3.21614875182795	-4.81603116610098	-2.58133121789201
C	-5.55747800784075	-1.79916422109950	-3.33020120874599
H	-4.69897671936450	-0.86495541178687	-1.61104936470147
C	-0.00134989680136	-5.62112124507590	0.16203472132726
H	0.04465116971237	-3.51310592539366	-0.27643907451639
C	-2.20344961034439	-6.64679499038203	0.26912605136391
H	-3.86476067693339	-5.34409800003933	-0.10746499732437
C	-4.41469494055344	-0.13700565429439	2.11561046777633
H	-3.67078571127085	0.00386358160129	0.11136079527198
C	-4.15173095627556	-2.28917789930734	3.20777436722820
H	-3.24739572644908	-3.84599777430028	2.05353082737605
C	-5.57327899551600	-2.93252261625394	-4.15883522280137
H	-4.74425434115259	-4.88149137595741	-4.53421043551521
H	-6.19442508082945	-0.94054197234909	-3.53622144079344
C	-0.80650648140062	-6.75495723638467	0.36682430822489
H	1.08088965126098	-5.73459894179750	0.24691849001968
H	-2.84649872970121	-7.51137135696085	0.42465639494342
C	-4.57850058861655	-0.95054666675533	3.24679120966698
H	-4.71667838502632	0.90969734828423	2.12823382944488
H	-4.27288634690696	-2.90115027638055	4.10229118086006
O	-6.35672705060261	-3.05886154677932	-5.23776507259643
O	-0.16348436912514	-7.89586801209580	0.64939993458539
O	-5.12665135893554	-0.53430116244901	4.39773957115080
C	-7.20756336528821	-1.98502412001931	-5.59073968416425
C	-0.92089110038896	-9.07031952576438	0.86657268058222
C	-5.67624821110346	0.76774506333636	4.45242161413988
H	-6.62587776220385	-1.07482143974944	-5.80255473845774
H	-7.93354240574807	-1.77669189025962	-4.79012411896643
H	-7.74040764903944	-2.29691978579385	-6.49461707311382
H	-1.59822586112169	-8.95007517806424	1.72588322663102
H	-0.20144495957339	-9.86787992557059	1.07773968487742
H	-1.50766256126460	-9.33267141376353	-0.02708422286216
H	-6.10715656726962	0.88069420942139	5.45285501112994
H	-4.90289007098292	1.53369044692858	4.29862465268660
H	-6.46479765881802	0.89224335346054	3.69369773724008

RR_0010_TS

Frequencies, energies and thermodynamic properties:
Lowest Vibrational Mode (1/cm) = -310.62
2nd Lowest Vibrational Mode (1/cm) = -31.42
3rd Lowest Vibrational Mode (1/cm) = 11.06
4th Lowest Vibrational Mode (1/cm) = 12.81
CREST Electronic Energy (a.u.) = -286.55968787
xTB Electronic Energy (a.u.) = -286.56165709
DFT//xTB Electronic Energy (a.u.) = -5585.42830663
DFT Electronic Energy (a.u.) = -5585.478802166858
DFT//DFT Electronic Energy (a.u.) = -5590.876396261475
Gibbs Free Energy (a.u.) = -5589.616066
Substrate Energy (a.u.) = -2466.961368809897
BIMP Energy (a.u.) = -3123.685000862278
DFT optimised cartesian coordinates:

O	1.88658457225262	0.56446876524863	-1.04842046999783
C	2.38078974167803	1.19059683434049	-0.10413690015229
N	3.51094258081334	2.02716387907577	-0.19210054707747
C	3.86318394014785	2.66717172468131	-1.48901627740245
C	3.65411811243354	2.69832303273760	1.05695056708469
C	4.55068255006230	3.68064384285987	1.48264351904018
C	4.45809497617026	4.15782115804839	2.79827108122135
C	3.49284926312353	3.67909689995663	3.68691004086900
C	2.57824028203720	2.71426935704297	3.26387814555091
C	2.66173391491356	2.23398032560614	1.95719268994181
C	1.82744100817614	1.29575206822394	1.23965925652363
O	0.70672183496030	0.79171370159595	1.57337171140253
C	0.92772781730903	-1.04774788966653	2.50449398557357
C	2.08199351495756	-1.44751473003634	1.84359043262291
C	3.31062081520301	-0.79414797534938	1.95439769898526
C	4.20970696803809	-1.34802307807520	0.98505241008611
C	3.49196362025788	-2.30236150939027	0.21976712086167
C	4.10447922618601	-3.01230744488343	-0.81472240364140
C	5.45888947481992	-2.76739256002950	-1.05598348978570
C	6.18434590602036	-1.83154432538597	-0.30410218285164
C	5.56613057254181	-1.11407143461487	0.71568968001019
H	5.31029980229703	4.10107018363201	0.82964465341235
H	5.16115162307986	4.92634331101012	3.12367155529024
H	3.44634562079397	4.07167624367484	4.70418569465923
H	1.79464820934351	2.34297510936539	3.92834827499838
H	3.55060424369345	-3.73386038439728	-1.41056782929890
H	5.95789650788467	-3.31790199732203	-1.85542826599571
H	7.23981767777365	-1.66361310184005	-0.52473284406605
H	6.12191581317614	-0.38705995718088	1.31133967566143
H	1.06507882529254	-0.54771818961948	3.46154456463469
H	-0.04405240880313	-1.48899657149501	2.29896117648859
H	3.57272872180058	-0.11190575455785	2.75861942375221
N	2.16991629995381	-2.33447882059534	0.71469619618518
S	1.10456859893088	-3.62060010440416	0.54567492425829
O	1.51167711620405	-4.37282126149265	-0.63113063616935
O	-0.23087471804199	-3.04466088256481	0.60761906500899
C	3.97961410550993	1.66685202999194	-2.65951444549731
C	4.33530058445500	0.32892090601991	-2.46756817015637
C	3.93035934986092	2.16454673291483	-3.96768512707261
C	4.62065258743055	-0.49014571685545	-3.56051825062765
H	4.38484390504554	-0.08313733715364	-1.45902503391881
C	4.21896620606545	1.34696518275736	-5.06028361138073
H	3.67902034325578	3.21269794526631	-4.14107421144242
C	4.56764986795983	0.01225570384527	-4.86134407169360
H	4.89039222615284	-1.53281664857002	-3.38568446302280
H	4.17296042743915	1.76107639168387	-6.06915535238537
H	4.79518025695356	-0.63290438853474	-5.71182041688817
C	5.30540132782154	3.20634124121998	-1.43194881327809
C	6.28891378483780	2.43520337823056	-0.79726197425622
C	5.69685073970709	4.35925009399158	-2.11672753793987
C	7.62210063212549	2.83382981066740	-0.80378104467131
H	5.99590903180186	1.51788482298710	-0.28278332019062
C	7.03579605730076	4.76069161668534	-2.12463763109391
H	4.95951489840010	4.95558783120957	-2.65522011310242
C	8.00184414037225	4.00699827038939	-1.46174034298339
H	8.36968792347861	2.22474277437942	-0.29250055891694
H	7.31961076684475	5.66995044066236	-2.65732387399833
H	9.04609161451695	4.32366095961568	-1.46530885207012
C	2.76971285305840	3.72846165513519	-1.72648672324626
C	1.58694141088554	3.38309145215528	-2.40048503235903
C	2.85068269050022	5.00261898326864	-1.14920086915997
C	0.53509655176498	4.29071573667982	-2.51959267543210
H	1.48383501419076	2.38626975659899	-2.82670013005292
C	1.80044446252131	5.91461675451715	-1.27264448458174
H	3.74233359200347	5.29688986416419	-0.59534723953897
C	0.63993005054555	5.56539020911534	-1.96093461436948
H	-0.37857489074133	3.98784256420307	-3.03636652475054
H	1.88866854290893	6.89889217244096	-0.81077768249806
H	-0.18418698291586	6.27629306595990	-2.04570546150843
C	1.35434810907696	-4.63881006602753	1.96834543540344
C	0.58115024617434	-4.44167474614211	3.11209635378863
C	2.34160519761000	-5.62334197978910	1.91228107705840
C	0.79957766598132	-5.26159989313748	4.21496574001983
H	-0.19218848198179	-3.67379063798393	3.12863506030801
C	2.54279488984517	-6.43262824336775	3.02636606859833
H	2.93435283723997	-5.75893816346507	1.00579403696756
C	1.77359759716288	-6.26993311368295	4.18751881215728
H	0.19714753340605	-5.12064517383716	5.11483571123047
H	3.30971424710199	-7.20917057024243	2.99358075131284
C	1.96866136041799	-7.17864910200087	5.36993363028653
H	2.99773871571443	-7.55814535493470	5.41345018518220

H 1.29512855115775 -8.04667837840055 5.29455614492796
H 1.73777376873833 -6.66082103741207 6.31002938400392
H -0.09031896430685 1.34898139266173 -0.23849415962383
N -0.96217306803922 1.71206164808333 -0.63256814044426
C -1.50107367890147 0.96956314133586 -1.63718581701624
C -1.45416219482512 2.82515081840183 0.05910222443334
N -0.74633374328335 -0.09464567269599 -1.95211854958818
S -2.94590228481071 1.37119756428680 -2.43524318155010
C -0.50815654590407 3.73734508786590 0.55094665869701
C -2.80822491321407 3.00959420286258 0.36862961557086
C -0.99944714791958 -1.04963341003886 -3.01746966935167
H 0.16459739375789 -0.16033363938308 -1.47958487757956
C -0.91831227310651 4.81508810588564 1.32900921703423
H 0.55124491099236 3.59992181623624 0.31959357439056
C -3.19091985926505 4.09064455293041 1.15875760856479
H -3.54516609419341 2.28693979835574 0.02513889496800
C -1.62474136835662 -2.31897184241008 -2.43542465151330
H -1.72585213325965 -0.57925688406976 -3.69807057410026
C 0.28273849217970 -1.33436041901837 -3.84221648756640
C 0.08521561900993 5.81859301952327 1.82842957418155
C -2.26164303493103 5.00904214925109 1.64193078887991
C -4.63355768219219 4.27218481908521 1.54482830045555
N -3.02558885263296 -2.09272369688749 -2.06879227359072
H -1.05121207323170 -2.64172305105104 -1.55399251079086
H -1.57344897598249 -3.12521724323162 -3.18202814886965
C 0.95371405153541 -0.00200320284914 -4.19686745903772
C 1.26430601989672 -2.20778856082825 -3.05261827280119
C -0.10869954078528 -2.04128362806184 -5.14680281904091
F -0.15765151411356 6.15841739316638 3.09930265642305
F 1.33537928416353 5.36788073824345 1.76725225663932
F 0.03464057973475 6.95332875154310 1.11725746778163
H -2.57453502351804 5.84337526521800 2.27246739020195
F -5.36254883724547 3.18015431426832 1.31170463286669
F -4.75666995342014 4.56888983784148 2.84195846866047
F -5.20620299558104 5.27622971462983 0.86984554076960
H -3.49537363496656 -1.38266556556735 -2.63335151224915
P -3.81513789476241 -2.46160589103271 -0.66485764211414
H 1.46276678793387 0.42444463030352 -3.32122736767300
H 0.21655615351542 0.72474407975665 -4.57742060223140
H 1.71508489937477 -0.16284557042782 -4.97407298458137
H 0.86879862266941 -3.21815965806575 -2.87350354177901
H 1.51315422294793 -1.75967458598904 -2.07511604661316
H 2.20795573786394 -2.30615359036625 -3.61222003474047
H 0.79399083229218 -2.22472974180586 -5.74826711562590
H -0.79108879553484 -1.41373886939798 -5.74078864107148
H -0.59339152247938 -3.01284705213649 -4.97469614348506
C -3.72706490529720 -1.18341961016901 0.59594721323567
C -5.54359190508609 -2.68054739916014 -1.09968563895018
C -3.07563395748957 -3.95002668192740 -0.00623349400937
C -4.87421618966835 -0.64655801053579 1.20345647123123
C -2.46218397416880 -0.72749435938017 0.99158457863190
C -6.20035776596590 -3.89674285025121 -0.86021609198062
C -6.24170448331305 -1.62442510974838 -1.69852483604415
C -2.46660698905297 -4.87506612932730 -0.85956199442205
C -3.01914576175251 -4.14756274472024 1.38384500373062
C -4.75575727384802 0.35003287923334 2.16027505125249
H -5.86753523430366 -1.00397700597774 0.92716596746857
C -2.33434422758098 0.26593867018910 1.95610766555406
H -1.55385139057412 -1.14222863990908 0.54346082685399
C -7.53098888208445 -4.05022776215228 -1.21780031128635
H -5.66893092466065 -4.72801484341831 -0.39166637841960
C -7.57635325038440 -1.77186634112786 -2.06408567058438
H -5.74862712423600 -0.66415687720777 -1.87961538341173
C -1.72828444369369 -5.93502788248512 -0.34282928114860
H -2.53884066388197 -4.76138129932499 -1.94233216102324
C -2.32544592211673 -5.22462719838189 1.90430887244384
H -3.50129063646175 -3.43950461762852 2.06162812657461
C -3.48702822229670 0.82884237459946 2.52374711007269
H -5.63560820183310 0.79463639080489 2.62512961733221
H -1.33569621426065 0.62298889379211 2.20798073180354
C -8.22912823943786 -2.99184385200379 -1.82346290771294
H -8.05973822886793 -4.98704915938100 -1.03963050945057
H -8.09744875619180 -0.93654138182306 -2.52883840448570
C -1.62570204560607 -6.09299980683085 1.04775539790837
H -1.22924370546860 -6.62060954703083 -1.02547102974951
H -2.26096572798617 -5.39119037001302 2.97991167208471
O -3.45816539994062 1.84540503331853 3.39951744552853
O -9.51051018056979 -3.22735230294547 -2.13750435172717
O -0.88447625179682 -7.04120745968959 1.63633136328205
C -2.19941005991024 2.31556484889967 3.84410113644793
C -10.26192127322227 -2.19747873934925 -2.75026570678703
C -0.05651284249994 -7.84842399664803 0.82124460358436
H -2.40162695287692 3.16928096583238 4.49962985315169
H -1.57201546374813 2.64308638600556 3.00035725771644
H -1.66548902552943 1.53105982303929 4.40354191528848
H -10.32752154914590 -1.31410444503585 -2.09681535876892
H -11.26476596623463 -2.60337119427712 -2.91707184078751
H -9.81914138475647 -1.90569202959953 -3.71492369760776
H 0.61733287872681 -7.22098853209202 0.21624211817208
H 0.53553602873204 -8.47127616034326 1.50004801313870
H -0.65701183194765 -8.49088228165765 0.15879213513388

RR_0019_TS
Frequencies, energies and thermodynamic properties:
Lowest Vibrational Mode (1/cm) = -320.15

2nd Lowest Vibrational Mode (1/cm) = -52.36
3rd Lowest Vibrational Mode (1/cm) = 8.03
4th Lowest Vibrational Mode (1/cm) = 12.31
CREST Electronic Energy (a.u.) = -286.5589663
xTB Electronic Energy (a.u.) = -286.56043782
DFT//xTB Electronic Energy (a.u.) = -5585.42273191
DFT Electronic Energy (a.u.) = -5585.471423635403
DFT//DFT Electronic Energy (a.u.) = -5590.86896562025
Gibbs Free Energy (a.u.) = -5589.610303
Substrate Energy (a.u.) = -2466.962960818341
BIMP Energy (a.u.) = -3123.682972280698
DFT optimised cartesian coordinates:

O	1.65953622535956	0.85636880907205	-0.53379262905025
C	2.30876373094919	1.02596598927406	0.49901604558748
N	3.45996445396059	1.83288432916308	0.61226189114357
C	3.62472317026041	3.00755241974607	-0.28897766414325
C	3.82962329945206	1.87181836215890	1.98793350154959
C	4.84282199401711	2.56419733290462	2.65427987814459
C	4.97413912905363	2.40436730092159	4.04186683283382
C	4.11539769357590	1.57879138326720	4.76960191597260
C	3.08863600776917	0.89648808391112	4.11556533347064
C	2.95177659532238	1.04648841277008	2.73607360795361
C	1.97240871030519	0.50822380648618	1.81673480496230
O	0.84388463857153	-0.03962880594247	2.07753208078740
C	0.96123577607382	-2.07286179409981	2.08160959051069
C	2.00766641038599	-2.19383861049892	1.17969043196559
C	3.27250299711025	-1.63820767229877	1.39144875464840
C	3.99880139217210	-1.67750344874154	0.15166303527070
C	3.12616929344457	-2.17558294200727	-0.84379634124802
C	3.54248430152942	-2.33331235178549	-2.16538603401121
C	4.86479854337183	-1.99970096427044	-2.47325549656687
C	5.74170846541563	-1.50484860040749	-1.49775804163897
C	5.31530098940746	-1.33182186585266	-0.18299430617963
H	5.52535106828858	3.23574116687962	2.14111451523140
H	5.76700098440569	2.95033766459694	4.55577075697260
H	4.23762559712963	1.48147426406119	5.84975291040656
H	2.38290695341202	0.26939340259038	4.66464106154425
H	2.86396849949518	-2.70682157018922	-2.92997795415317
H	5.21445546170125	-2.12209440780379	-3.49970202804270
H	6.76560964266751	-1.24784884985114	-1.77454665289465
H	5.99136304895750	-0.94890059691986	0.58408896517770
H	1.22044360101326	-2.05798654678162	3.13869289651579
H	-0.05664024081168	-2.34984767187337	1.81748602858723
H	3.69238804654767	-1.41273723218601	2.36810955579804
N	1.87488274155953	-2.47111532245727	-0.23435665730020
S	1.05767577036156	-3.87192775663638	-0.69499144521876
O	1.03106477354470	-3.88626995148691	-2.14838790671139
O	-0.20408131074268	-3.87222818887043	0.03923756443057
C	3.48059060026382	2.64848684731924	-1.78396310479999
C	3.79547195267573	1.37784196943276	-2.27513593119596
C	3.20459919864007	3.66967196925478	-2.70068488866905
C	3.80418614113822	1.13130102757276	-3.64740803365046
H	4.01502463420438	0.56706736722432	-1.57985949669541
C	3.20982228855417	3.42345802452878	-4.07443047719526
H	2.98672547428549	4.67768361156969	-2.34298250625299
C	3.50619346023546	2.14937555369988	-4.55526343466508
H	4.04018833749688	0.12810768351101	-4.00633863064265
H	2.98359533426197	4.23596785133056	-4.76723446573020
H	3.50987299675824	1.95027034582400	-5.62839807729407
C	5.08253532181465	3.50055795701103	-0.24590262987559
C	6.11547919576694	2.55365516351690	-0.20704631230167
C	5.41462793001555	4.84954287594550	-0.39142448224191
C	7.44822152042830	2.95044121746587	-0.25967247527070
H	5.86344311353028	1.49479231972898	-0.11870974133825
C	6.75305742888234	5.24940552106201	-0.44487636336553
H	4.62942121726512	5.60272915494497	-0.46654457758892
C	7.77450219945026	4.30505317256646	-0.36959048375813
H	8.23750194589283	2.19796235038947	-0.21396885729098
H	6.99231325989850	6.30921407423794	-0.54802665351375
H	8.81892025215248	4.61906786430219	-0.40638206125167
C	2.56682483944679	4.02949133079367	0.17910191746846
C	1.27370350821383	4.00255022314937	-0.36616321087398
C	2.80773244437527	4.89718701046194	1.25391280866195
C	0.26974038579326	4.84058561898246	0.11679391330914
H	1.04566828783854	3.31103725995662	-1.17639852055250
C	1.80095823342871	5.73390474852486	1.74183656860684
H	3.79245514086118	4.92944883750041	1.72089693676012
C	0.52915748111299	5.71637121488137	1.17115130389550
H	-0.72939347049077	4.78178364991382	-0.32190878682898
H	2.01721498161814	6.39746333686827	2.58093623706816
H	-0.26132833168459	6.36416746634686	1.55698957607074
C	2.04916700463791	-5.21044903131935	-0.11408849576707
C	1.87500983280656	-5.66978226098922	1.19400490980465
C	3.02330157270433	-5.74470900600759	-0.95663792493037
C	2.70501194995446	-6.68349699720281	1.65866139688482
H	1.09640834807202	-5.24313506988483	1.82778272249213
C	3.84328737227371	-6.76004965764900	-0.46847864683866
H	3.12844417421214	-5.37993338305010	-1.97942832498261
C	3.70098014610289	-7.23938780513557	0.83997604638797
H	2.57694368236486	-7.05630007699057	2.67704054782970
H	4.60578231340835	-7.19182495788062	-1.11946347619649
C	4.59791178156380	-8.32338982470625	1.37149595928747
H	5.20664010138807	-8.76470264865409	0.57268029958546
H	4.00836000756857	-9.11876146622904	1.84865336364273
H	5.27634467588948	-7.91583292913638	2.13615923919042

H -0.31917115383586 1.10745550776421 1.25490018750368
N -1.03311030545763 1.80100200195765 0.98226878041289
C -1.63503436710408 1.54800999241734 -0.21717088063055
C -1.23382988668613 2.77989275203246 1.95590572315932
N -1.06741205224453 0.51299240971942 -0.86154939508664
S -2.95263330084750 2.41717430171218 -0.84461941644843
C -0.26360749582324 2.85826100119141 2.96739371776650
C -2.33333323032425 3.65231304944376 2.02202858813067
C -1.55154581219171 -0.08911622981582 -2.08580263153298
H -0.11734159312517 0.25661533907729 -0.55879282286015
C -0.37266155241791 3.79904492256497 3.98633139859714
H 0.58445408679841 2.17218025875381 2.95653289767555
C -2.41805185734440 4.57820913709220 3.05422129993901
H -3.11826613762102 3.59316975770333 1.27519730750821
C -1.66179267192272 -1.60700168042315 -1.89465065183921
H -2.55294321742732 0.32971681389887 -2.24835936656781
C -0.70412673399065 0.25796880246868 -3.35071122447936
C 0.71791076271157 3.83815809535099 5.02289275210896
C -1.44527430216831 4.68123695415024 4.04806738503649
C -3.58760528559907 5.52034414990948 3.10892632200139
N -2.37758833377429 -2.04535707044459 -0.70228569737855
H -0.65674328437680 -2.03342848099187 -1.79974936122056
H -2.11070980974828 -2.04998694007411 -2.79746732428396
C -1.60112495074910 0.08040238814088 -4.58307475847257
C -0.23639549649673 1.71512204563536 -3.27593219491629
C 0.53913160491549 -0.63531908306731 -3.47063149964607
F 0.48926548881749 4.76292774888473 5.95806140984905
F 0.83951639190133 2.66283097680771 5.65029201689487
F 1.90886146148227 4.10949831799701 4.48198298386363
H -1.52672193015544 5.41710694931633 4.84857682503033
F -3.20472432908612 6.78778013975158 2.90684278571745
F -4.51740201371264 5.24179935428403 2.19356312436052
F -4.18886044771321 5.49238709055201 4.30501562877784
H -1.80761232546167 -2.55868960120610 -0.02945183435383
P -3.99130544535940 -1.91530603907436 -0.40958118621443
H -2.43950608568625 0.79475848580288 -4.55632934126746
H -2.01977073986246 -0.93609426859876 -4.64808116120315
H -1.02351415397379 0.26475372028874 -5.50153931244073
H -1.07408097027925 2.39485137443295 -3.05974012310123
H 0.22279129276006 2.00702943473189 -4.23333366913367
H 0.52134933332863 1.82403995251916 -2.48568622359630
H 0.28881697954828 -1.68199796286636 -3.69916550502714
H 1.13171411334238 -0.61257911315745 -2.53999817922832
H 1.18236127595054 -0.25703415540739 -4.28030160701874
C -4.38903833366576 -0.57400845862266 0.71789056260807
C -4.82395591758632 -1.66391766739738 -1.97843037487632
C -4.48945292822347 -3.47062004496517 0.34288779308250
C -3.44018270437064 -0.22888875800903 1.69586047067918
C -5.61934403106484 0.08948389323369 0.69087035291660
C -5.04075283629473 -0.37687796736176 -2.48511066723004
C -5.16667637992135 -2.78192634914016 -2.76040883000590
C -3.71600339772318 -4.62239442823444 0.16694610376286
C -5.68443514007778 -3.54438960258042 1.08065147518539
C -3.71387441309059 0.77364204632382 2.60914537064786
H -2.46981619236406 -0.73158998812222 1.72530328088826
C -5.89551376368564 1.11302810348363 1.59526398139407
H -6.37788287878031 -0.17503175852874 -0.04933654022985
C -5.59229447821240 -0.19561792564071 -3.75089613500326
H -4.76497015221132 0.50416030862483 -1.89847833146482
C -5.71445921988382 -2.60942376778928 -4.01923483680790
H -5.00505019184383 -3.79329985426354 -2.37999121125038
C -4.11834452688885 -5.83837111995218 0.71742624487986
H -2.78000297107011 -4.57915618003366 -0.39413413352188
C -6.09399693443836 -4.74897587849847 1.62284111879382
H -6.29523627090442 -2.65221063135929 1.23779748429758
C -4.93463201306609 1.47005545654203 2.55263021970621
H -2.98751875935235 1.05814594254285 3.37140674256032
H -6.85343563868654 1.62743629960877 1.54185032814223
C -5.93085223874626 -1.31439544753811 -4.52693926909751
H -5.74885737199548 0.81618918343705 -4.12129334959834
H -5.98908621381877 -3.46336675823316 -4.63900121030552
C -5.31378103700689 -5.90761304008272 1.44671481132530
H -3.49431719176306 -6.71897695054885 0.57389707660832
H -7.01610648112448 -4.82620226156508 2.19962342756195
O -5.09733637263159 2.45994519134578 3.44304687837548
O -6.46354088815706 -1.24022115101326 -5.75214618365188
O -5.78300871624183 -7.02752398192968 2.01190450129035
C -6.33341350084306 3.14845592904966 3.47255558813386
C -6.68597807647384 0.03509322511003 -6.32363637330799
C -5.03606769554601 -8.22151620375348 1.88096791458288
H -7.15847939347442 2.45999252150866 3.71239591601607
H -6.53340633561792 3.64306099167468 2.51059036447133
H -6.24349446792233 3.90785252774600 4.25541508584548
H -5.74212281819985 0.59320772175241 -6.41983472384037
H -7.39651783945124 0.61948199010677 -5.71949965146475
H -7.10966055658541 -0.14132516553215 -7.31734342076295
H -5.59457714278324 -8.99623037547398 2.41582487774405
H -4.93193191407939 -8.50790474804462 0.82320379605725
H -4.03685426523026 -8.11276728092828 2.32985906078380

RR_0070_TS
Frequencies, energies and thermodynamic properties:
Lowest Vibrational Mode (1/cm) = -319.56
2nd Lowest Vibrational Mode (1/cm) = -42.44
3rd Lowest Vibrational Mode (1/cm) = -2.76

4th Lowest Vibrational Mode (1/cm) = 10.9
CREST Electronic Energy (a.u.) = -286.55654458
xTB Electronic Energy (a.u.) = -286.56034446
DFT//xTB Electronic Energy (a.u.) = -5585.42362309
DFT Electronic Energy (a.u.) = -5585.471067184581
DFT//DFT Electronic Energy (a.u.) = -5590.868818045473
Gibbs Free Energy (a.u.) = -5589.611587
Substrate Energy (a.u.) = -2466.96287753694
BIMP Energy (a.u.) = -3123.683471442237

DFT optimised cartesian coordinates:

O	1.73705985779880	0.37479059044054	-1.11381544580123
C	2.45397623662803	0.92877874501372	-0.27990832159795
N	3.57162279312050	1.73528147026349	-0.58062372906983
C	3.60445858049781	2.48918300552199	-1.86490420050722
C	4.04289781777842	2.29679182237189	0.64106169032009
C	5.06784900137515	3.20748802533167	0.90405804906207
C	5.31288719278689	3.58728277631260	2.23217682928644
C	4.55376648529273	3.08465873589330	3.29049779644035
C	3.51456172108647	2.18854359340732	3.03557132013124
C	3.26543880255418	1.80223891847837	1.71924077123731
C	2.24395145465412	0.94215434948751	1.16030648398899
O	1.16422123639415	0.52145583269810	1.70657954202497
C	1.36851662111102	-1.35618040479887	2.46816428982424
C	2.34427582383159	-1.79972100751228	1.58832035988356
C	3.59626018328876	-1.19199151400010	1.45872352304258
C	4.22087031324291	-1.69053273810635	0.26412855852202
C	3.29552411298857	-2.54113274581125	-0.38472613329078
C	3.61057888314510	-3.17972261686403	-1.58411851239422
C	4.88511083036062	-2.96646486140292	-2.11770187216691
C	5.81282560634221	-2.12595218212505	-1.48634355110035
C	5.48685536264157	-1.47636767676472	-0.29802115657419
H	5.67448856104933	3.64592623288291	0.11661650929894
H	6.11464355710554	4.30063875728439	2.42994915428214
H	4.76285026703942	3.40566485417990	4.31243706718621
H	2.88409016498758	1.80530807831134	3.84072806864363
H	2.89185167544250	-3.82435932671986	-2.08635332623053
H	5.15547856040479	-3.46191954096926	-3.05169921206666
H	6.79611769498441	-1.97528557294138	-1.93527424797097
H	6.20263907727102	-0.82104202240580	0.20231279373981
H	1.70967770716102	-0.93871062351566	3.41368672976377
H	0.34577903070342	-1.72272306027145	2.41918493209494
H	4.08308209891170	-0.60852399328901	2.23563703643803
N	2.11158092243850	-2.59516736431745	0.40225953706750
S	1.30715581667507	-4.06568016387893	0.57848941575986
O	1.19070528607647	-4.64429584661737	-0.74987177606322
O	0.09298613022034	-3.78367582630276	1.33803396577554
C	3.35515540741374	1.59297910902245	-3.09757392065982
C	3.70071954518897	0.23815202130207	-3.10916964731473
C	2.94785758894124	2.18930074862289	-4.29637302574336
C	3.61404000836248	-0.50459381327575	-4.28567325222015
H	4.02180425276385	-0.24686289436398	-2.18679419016276
C	2.85865786800872	1.44623402293714	-5.47462956729083
H	2.70072373231578	3.25228884496052	-4.31885226321627
C	3.18886813009372	0.09236264982180	-5.47440072294932
H	3.87719664928574	-1.56358328184579	-4.26720197393091
H	2.53175175659856	1.93416366402140	-6.39467340892450
H	3.11944293263367	-0.49412096434906	-6.39229855974307
C	5.03451953992085	2.98287240412422	-2.14956341095820
C	6.11453845717981	2.13943725750766	-1.85408300683737
C	5.28299652234574	4.17636582851225	-2.83152296890239
C	7.41533191689543	2.50365140311472	-2.18919076057719
H	5.92641106171717	1.19300727058874	-1.34271625578557
C	6.58897049528205	4.54328045717474	-3.16863785574044
H	4.45608315658374	4.83063968230490	-3.11018861305464
C	7.66036876864695	3.71463646921916	-2.84241491831315
H	8.24352313334152	1.83824354292351	-1.93913292713792
H	6.76329118220855	5.48448017896663	-3.69288956143625
H	8.67985385359751	4.00382630153468	-3.10274857304524
C	2.53890194229012	3.59494160590309	-1.71159383677027
C	1.20526131110871	3.33398665551745	-2.06348976208960
C	2.82485305237174	4.81307611994825	-1.07910734050715
C	0.20054823516056	4.27063808754379	-1.82699107793558
H	0.94625871437223	2.37957016133658	-2.52030892696790
C	1.81763978134177	5.75096138694181	-0.83785357694034
H	3.84470278008091	5.04229418792956	-0.76888544049804
C	0.50190670352359	5.48804208870070	-1.21586264501111
H	-0.82907184489747	4.02559268587815	-2.09928939689081
H	2.06916465432223	6.68955659889098	-0.34105594646736
H	-0.28832706663844	6.21601393580168	-1.01769678374348
C	2.36603980147646	-5.07390464109313	1.56531710153971
C	2.26510051575211	-5.01086067816094	2.95629359735930
C	3.31894403423899	-5.87342666418135	0.93273856767227
C	3.14294222785007	-5.77115848055562	3.72247852266620
H	1.50715401082476	-4.38166735597455	3.42491721715466
C	4.18694057182262	-6.62453704480467	1.72036926908084
H	3.37163383368752	-5.91301740742043	-0.15634455302934
C	4.11187809930241	-6.58718908972328	3.11995480586283
H	3.07487776648612	-5.73241280265871	4.81151246407713
H	4.93672001090027	-7.25522086926367	1.23866319226147
C	5.03051134668719	-7.43047374905426	3.96079162438218
H	5.25097738942910	-6.94085811081169	4.91836139002052
H	5.97327834434299	-7.63341389724222	3.43665517790805
H	4.55654902870495	-8.39901332338721	4.18426506305381
H	-0.11861141740161	1.22631414687374	0.61911797086195
N	-0.89071480903132	1.75204526055117	0.17937527136242

C -1.55538168504833 1.07300174350383 -0.80023775781315
C -1.08180824671250 3.00924773630404 0.75513137101241
N -0.98678938465945 -0.11536538945385 -1.07150898571396
S -2.94118495663806 1.64276761869442 -1.60052158785406
C -0.02760220342903 3.49859884019697 1.54156248555654
C -2.24853211921056 3.78531810609126 0.64868410044318
C -1.52832448307161 -1.13461652239673 -1.94542193726025
H -0.00719441872119 -0.22639075665254 -0.77475069007144
C -0.12776878674843 4.73124990556041 2.17832562592005
H 0.88148925175779 2.90671985173381 1.65486817022226
C -2.32109305634073 5.01181259173402 1.29761034138005
H -3.09305224309909 3.42049765256837 0.07294297678665
C -1.56815700244270 -2.47296597181292 -1.19728485848191
H -2.55281663039660 -0.81603950772341 -2.17594288126677
C -0.78125836604127 -1.27606910844114 -3.30813799782188
C 1.04586999133120 5.19564064141316 2.99818841462925
C -1.27025710574390 5.51469200327269 2.06455587529673
C -3.57129104057076 5.83883650011699 1.18747233899521
N -2.21450430646197 -2.44782253925106 0.10993970380248
H -0.54346800828134 -2.81092318093030 -1.00612994947690
H -2.03681636270837 -3.23138359619650 -1.84356772041474
C -1.74931229400070 -1.91431901858159 -4.31328299047819
C -0.36917118338620 0.10906538045586 -3.81730578059841
C 0.48641619362497 -2.13248341357412 -3.17688347360690
F 0.84248943435204 6.40572484680655 3.52450663965195
F 1.29646682190356 4.36203391459787 4.01442736821607
F 2.16449174635283 5.26210954040819 2.27208145569476
H -1.34503494127960 6.48115832630423 2.56387987475849
F -4.08061009029640 6.12589142888776 2.39240156827347
F -3.33026478156510 7.01296209220349 0.59080946674865
F -4.53160624451422 5.23262953042848 0.48762667408827
H -1.60001968609757 -2.66746585555833 0.89459167778055
P -3.81901718799733 -2.24268378653959 0.41111701077978
H -2.61226862538001 -1.25262298031967 -4.49107232186456
H -2.13129673571476 -2.88589425896785 -3.96232184381196
H -1.24081941409710 -2.08136661008039 -5.27493277769304
H -1.21283371931070 0.81495894308225 -3.78663498022488
H -0.00675212869188 0.03006536759851 -4.85414376552757
H 0.45033256710984 0.50651319170587 -3.19950109446926
H 0.26508202630846 -3.19177043214380 -2.97850398670602
H 1.13230625884478 -1.75160672490223 -2.36733623994681
H 1.06368285660930 -2.07708110900943 -4.11284793306968
C -4.20425365338994 -0.59661562476507 1.01797756629729
C -4.71074525428470 -2.57092889557077 -1.11041116109250
C -4.26004463114355 -3.44361897877948 1.67472483758638
C -3.21404484452778 0.08970168292452 1.74178272048689
C -5.46541217958788 -0.01519999594521 0.85755439831175
C -5.01017335446542 -1.54570499327989 -2.01510744477658
C -5.00345356352665 -3.90374974001251 -1.45340455459495
C -3.48124589353230 -4.58981408434596 1.86137315189267
C -5.42282759771279 -3.26293391953620 2.44459776433562
C -3.48120502454464 1.33648020274405 2.27838090989099
H -2.21954772626218 -0.34639199304275 1.86650549761360
C -5.73762959575052 1.24766634692554 1.38101515446983
H -6.25276335597328 -0.53795347169393 0.30925814563675
C -5.58990100199735 -1.83377863737921 -3.24830830066080
H -4.76984953507209 -0.50634010726755 -1.77434378610912
C -5.57811759086006 -4.19760238497978 -2.67716280505041
H -4.77929344555275 -4.71621829567285 -0.75809531050772
C -3.84826439847745 -5.55288562658495 2.80013667598714
H -2.56929013502233 -4.73700803372216 1.27868609975322
C -5.79716695866652 -4.21724536050347 3.37310788415395
H -6.03655023816507 -2.36743117677681 2.32174941360564
C -4.73962289493850 1.93521513830981 2.08748524368284
H -2.72429338440329 1.88385754667171 2.84135165296285
H -6.72251391270351 1.68605004692257 1.22988634627789
C -5.87191191563198 -3.16532858792901 -3.58824086729853
H -5.80839895949767 -1.01793803206690 -3.93542582923405
H -5.81263258089202 -5.22389810590196 -2.96085324967257
C -5.01405760914001 -5.37245829591313 3.55783724971428
H -3.22181062382673 -6.43362821158004 2.93122464459812
H -6.69445578025761 -4.09530987810224 3.98051943685506
O -4.90014960473875 3.16081137014750 2.60783011846842
O -6.41858786897540 -3.54034343077108 -4.75061183095466
O -5.45206727639517 -6.24158993916592 4.47786268474052
C -6.17948015006421 3.76285606178017 2.53807422797389
C -6.70344211658088 -2.54958449151974 -5.72022243953494
C -4.70431773183402 -7.41894980703654 4.71325106195507
H -6.92772639908104 3.14753122238064 3.06106443585449
H -6.48940408751270 3.91298717325118 1.49343821633412
H -6.08728979798918 4.73642795162971 3.02888538874892
H -5.78677308950159 -2.01971642450939 -6.02149883919153
H -7.43707323661592 -1.82335350715319 -5.33854086952849
H -7.12373345076300 -3.07411941336588 -6.58406618125925
H -5.23721864333063 -7.97341900722529 5.49219912431070
H -4.64037284316395 -8.03437676406512 3.80280324097929
H -3.68893618800943 -7.17780489823158 5.06330810667516

RR_0081_TS
Frequencies, energies and thermodynamic properties:
Lowest Vibrational Mode (1/cm) = -312.14
2nd Lowest Vibrational Mode (1/cm) = -41.15
3rd Lowest Vibrational Mode (1/cm) = -9.46
4th Lowest Vibrational Mode (1/cm) = 11.92
CREST Electronic Energy (a.u.) = -286.55601704

xTB Electronic Energy (a.u.) = -286.55903389
DFT//xTB Electronic Energy (a.u.) = -5585.41905635
DFT Electronic Energy (a.u.) = -5585.470378064324
DFT//DFT Electronic Energy (a.u.) = -5590.870005138936
Gibbs Free Energy (a.u.) = -5589.610432
Substrate Energy (a.u.) = -2466.963875977195
BIMP Energy (a.u.) = -3123.678182229858

DFT optimised cartesian coordinates:

O	1.25027318081645	0.05545760439590	0.35952477468748
C	1.95020605335779	-0.73731716920164	-0.28665754601348
N	3.16492015098307	-1.30119622254913	0.16198418001698
C	3.46135476324461	-1.43116498736685	1.61486101591163
C	3.58610184590653	-2.26361200824143	-0.79831027825908
C	4.67305766727621	-3.14124747358153	-0.82188250146308
C	4.84823753211599	-3.97662519400978	-1.93449996390197
C	3.96369884140528	-3.95266773942917	-3.01548584108242
C	2.86321573193817	-3.09540544039526	-2.99270836021910
C	2.67733599260781	-2.26342627377803	-1.88729083390349
C	1.63224218465568	-1.31410962512254	-1.58491752498319
O	0.54866130756057	-1.09006552214218	-2.22579513525834
C	0.72872220948698	0.41284513340478	-3.61937963393454
C	1.77520898826967	1.11686167837097	-3.03471341941686
C	3.04600468088057	0.59296475498166	-2.81097205826779
C	3.77967513551326	1.51784459125478	-1.99605670013601
C	2.90898872805030	2.58585631913957	-1.67061158383782
C	3.32954632492059	3.65009966161090	-0.87372814716239
C	4.64903173688267	3.63590883424340	-0.41515912024675
C	5.52643799762077	2.58923062421952	-0.73538687099773
C	5.10011462923511	1.52416407741891	-1.52444188352881
H	5.38525415705696	-3.21306961675792	-0.00507484406936
H	5.69922597917947	-4.65950310684347	-1.94382699342872
H	4.12770784267709	-4.61279236282638	-3.86898199527145
H	2.14372489027993	-3.07235629799911	-3.81398954368677
H	2.65227002949622	4.46030795194225	-0.61749301241693
H	4.99504750899832	4.45860483499813	0.21323712796474
H	6.55019766251281	2.60903071672840	-0.35742794627812
H	5.77585177718817	0.70681876142097	-1.78411539353961
H	0.98873520124746	-0.33049624894375	-4.37061595062921
H	-0.28129944752582	0.81870329733511	-3.63345026354781
H	3.44649020934411	-0.28853283281540	-3.30329559003706
N	1.65268814623699	2.33529984546251	-2.27593201096147
S	0.76385932420993	3.60882893265353	-2.96080227526241
O	0.94546731815542	4.76311980210503	-2.09836771142466
O	-0.56866858493670	3.09159843277744	-3.22429445924203
C	3.31319717905123	-0.10701682326262	2.39212174155160
C	3.44266238317207	1.14032028471438	1.77742173806651
C	3.22836975106761	-0.15861114349362	3.78948765030260
C	3.44528859274096	2.30669672210854	2.54249167850887
H	3.53039512972726	1.20637557756205	0.69263451605210
C	3.22445922180766	1.00699060367312	4.55482705824693
H	3.16839441645697	-1.12436775951390	4.29504216414909
C	3.32627552927375	2.24970668347304	3.93155918551545
H	3.53759789452007	3.26952886742719	2.04065650123850
H	3.14702838650114	0.93794109127380	5.64135069939338
H	3.31491976167853	3.16894041338573	4.51909113405937
C	4.95801306872491	-1.72623330527901	1.82863611204235
C	5.89333463077383	-0.98878830452575	1.09017941643709
C	5.41926090677004	-2.59388994040165	2.82024582613520
C	7.25818927315505	-1.15367499786749	1.29991994135168
H	5.53557963648181	-0.28737622599138	0.33468937832011
C	6.79172130175695	-2.76017606692220	3.03345026576293
H	4.71376440287301	-3.14936352030737	3.43874808755258
C	7.71557462250884	-2.05100251496668	2.26988048680142
H	7.96943230061416	-0.57864643140562	0.70406506919724
H	7.13398840923440	-3.45038032247005	3.80645275355513
H	8.78575490121906	-2.18580481221735	2.43492244341709
C	2.50677465841711	-2.52295823413832	2.13022499238326
C	1.27600787528466	-2.17154276222492	2.69749693947179
C	2.77349717208335	-3.88379447156693	1.92050646442459
C	0.36206626290728	-3.14621810969243	3.09381711153474
H	1.02984116469789	-1.12018834137268	2.83686117926282
C	1.85208708923478	-4.86136733879102	2.30058007331435
H	3.71376361174306	-4.19219554764343	1.46281478206669
C	0.64613221488515	-4.49775861818603	2.90071711535960
H	-0.57588918746431	-2.83908212003931	3.56082338058394
H	2.08615653755339	-5.91406645696725	2.13327503826201
H	-0.07063563753927	-5.25935229807501	3.21458112792083
C	1.57129297463973	3.91932186034143	-4.50364036256365
C	1.08513318962113	3.32193335949014	-5.66494185721949
C	2.70807506430187	4.73104826864840	-4.51574125861013
C	1.76188545683104	3.54122077250827	-6.86314965709262
H	0.18603316948136	2.70544426334200	-5.62851839877251
C	3.36946386572810	4.93337512701371	-5.72218844942137
H	3.05865657988033	5.20441963333986	-3.59696787218074
C	2.91081825491886	4.34120843833073	-6.90948359178479
H	1.38722606601022	3.08514282133064	-7.78155891782340
H	4.25597362815383	5.57048139643099	-5.74750775708521
C	3.65265989519817	4.55791190414463	-8.20005302560562
H	4.59144563389665	3.98316521493100	-8.20075036211072
H	3.91733674184547	5.61673933208244	-8.32687760108864
H	3.05523286042816	4.23559273287248	-9.06203093640343
H	-0.02500497896207	1.22551554251812	-0.31183469008154
N	-0.89497831150025	1.78030475209076	-0.35281759225106
C	-2.04367175079250	1.08646467973469	-0.63446022505656
C	-0.80186811799298	3.12457401571011	0.00681019637831

N -1.82530646860343 -0.05290530262669 -1.30114310066911
S -3.57271050846340 1.59358929631855 -0.09604923070985
C -1.68473796813508 4.08223987193431 -0.52789990411392
C 0.22769425393322 3.54926701640818 0.85211232119210
C -2.84731538927980 -1.00395499072723 -1.69538000518534
H -0.85341734064257 -0.29139622074183 -1.57304063650621
C -1.54948717035767 5.41501676211831 -0.18782173866283
H -2.45559155503665 3.77097579012471 -1.22727520304593
C 0.33665648170894 4.90671152996477 1.18632621614088
H 0.93770789163985 2.81778285630419 1.25326156585949
C -2.33378720015411 -2.42828728275555 -1.45514486973406
H -3.71411789340800 -0.80780588838712 -1.05130722040091
C -3.33988718998621 -0.83512516929548 -3.17040738468448
C -2.46753686982539 6.44939211587428 -0.77617330979199
C -0.54502511523112 5.84935628664480 0.68082401346726
C 1.40874574049597 5.35603577734597 2.14152393647704
N -1.99205882111733 -2.75286638350853 -0.07036332771239
H -1.40995862142424 -2.57652882385301 -2.02832235100274
H -3.07789303977685 -3.14480534054388 -1.83259436727458
C -3.47480796355533 0.64956254638172 -3.52694841070863
C -2.37091752584563 -1.49482519667271 -4.16197287484689
C -4.72761073411162 -1.48250956665845 -3.28089226170658
F -3.12754598123089 7.11842794074719 0.17706515401186
F -1.78685660638267 7.36339111789443 -1.47728676164071
F -3.37821193355331 5.92132677833530 -1.59290541501291
H -0.45845872635397 6.90128643175955 0.95421021211823
F 2.63100250160125 5.33836882140991 1.58693386150322
F 1.20704046591704 6.59957610641036 2.57607719546319
F 1.46970348558162 4.56649356048302 3.22109317689316
H -1.01003484519913 -2.94473705259469 0.12142832096656
P -3.00242409764067 -2.71725063486041 1.23372045039444
H -4.14519695372102 1.16486883873890 -2.82599803875108
H -3.88520422231613 0.74280619043766 -4.54395053500454
H -2.50291208423761 1.16569953743977 -3.50212273250372
H -2.63356688935443 -1.20308478348768 -5.18986192558357
H -2.41018382026005 -2.59297481710460 -4.10868697974767
H -1.33382168154684 -1.18222071174057 -3.96702620751314
H -5.09532252302989 -1.41514195944870 -4.31603653611616
H -5.44735887885116 -0.96447357087483 -2.62649659490916
H -4.71633036415089 -2.54674418647797 -2.99883794556785
C -4.69369129236061 -2.67083732453597 0.62899996475266
C -2.74285799952978 -4.25558863880629 2.13755036593630
C -2.67458301731673 -1.29020818199891 2.26969764268633
C -5.45146476265350 -1.49654239969472 0.68257577393455
C -5.24320353333515 -3.82154293598248 0.03329696229081
C -2.40685049594870 -5.41758069284844 1.42130794979436
C -2.93658704653850 -4.33646254616614 3.51944854826530
C -3.52534527171058 -0.91567138935756 3.32760111341768
C -1.52871464437945 -0.53353825098886 2.01046153906430
C -6.73816497069266 -1.45539783042131 0.15037473128725
H -5.02805710635363 -0.58488242957895 1.10881951139322
C -6.51708721728812 -3.78669544054096 -0.50459165986604
H -4.67262267672036 -4.75220127285872 -0.01223923540366
C -2.28736471543635 -6.63286270855171 2.07480765133130
H -2.22888655956575 -5.36563009902804 0.34511446988842
C -2.80771574594387 -5.55210175807378 4.18781715479239
H -3.17898491233471 -3.44463418188827 4.10132410374581
C -3.22579375933289 0.19415277875788 4.09723026725398
H -4.43609849942382 -1.48180965358606 3.53975890875125
C -1.21549883649670 0.57974419095491 2.78596505645331
H -0.85853831240973 -0.78767747259687 1.18629602935913
C -7.27614322598615 -2.60161774541125 -0.45240737170343
H -7.30252721771533 -0.52557989230848 0.19956144852513
H -6.95791468619688 -4.66657921626328 -0.97394232707316
C -2.48891352018693 -6.71205409853762 3.46425094417949
H -2.02527262772913 -7.54264276864583 1.53379097027408
H -2.95485349897353 -5.58480832069505 5.26600671962006
C -2.06825464367707 0.95377034986295 3.83301845217248
H -3.87720803204390 0.50899714204114 4.91296324054531
H -0.31194718139794 1.14335465909708 2.55049587106606
O -8.49594786922074 -2.65720624903166 -0.99958017348476
O -2.34789762933394 -7.92333362588556 4.01777178469466
O -1.86400515199690 2.01511687966694 4.62277368601759
C -9.29612552148679 -1.49000850312622 -1.00103039827821
C -2.53100723537181 -8.06017042657071 5.41386324316702
C -0.77654657117077 2.87232513455848 4.32865995454472
H -9.50839639980246 -1.15607249654235 0.02593801071287
H -10.23304752019361 -1.75825592272079 -1.49932961591697
H -8.80375288365388 -0.67663585276938 -1.55583483835327
H -1.80134998160096 -7.45088460062361 5.96880452634851
H -2.37343731059604 -9.11858398349203 5.64418485580655
H -3.55051607885957 -7.76787808375934 5.70833841623344
H 0.18568069321739 2.34165163614998 4.40593568247281
H -0.87795310723180 3.29268537028293 3.31634408543808
H -0.80729564156250 3.68079993818250 5.06629902884077

RS_0015_TS

Frequencies, energies and thermodynamic properties:

Lowest Vibrational Mode (1/cm) = -350.36
2nd Lowest Vibrational Mode (1/cm) = -34.09
3rd Lowest Vibrational Mode (1/cm) = -1.39
4th Lowest Vibrational Mode (1/cm) = 10.09
CREST Electronic Energy (a.u.) = -286.56009797
xTB Electronic Energy (a.u.) = -286.55850257
DFT//xTB Electronic Energy (a.u.) = -5585.42893365

DFT Electronic Energy (a.u.) = -5585.475583633167
DFT//DFT Electronic Energy (a.u.) = -5590.874325616264
Gibbs Free Energy (a.u.) = -5589.61582
Substrate Energy (a.u.) = -2466.962519765415
BIMP Energy (a.u.) = -3123.679896028004

DFT optimised cartesian coordinates:

O	1.10654477346122	-0.17739415034703	2.38950788924495
C	1.67447562226267	-0.05122008697523	1.29288421583019
N	2.95366560990605	-0.52216770731786	0.97002776893670
C	3.84599800053534	-1.25331407604415	1.88889408659584
C	3.31342753599983	-0.02318388801904	-0.29951828024783
C	4.53023659136978	-0.05749083609616	-0.97578546180902
C	4.60042944501438	0.51826585696622	-2.25191353190250
C	3.47964782808477	1.10836788651193	-2.84396166072946
C	2.26265777537993	1.15562010215459	-2.16315027576780
C	2.18253972886335	0.60659544618591	-0.88190837070139
C	1.11799300357487	0.54924627601805	0.09107158553585
O	-0.12138979972650	0.87681244127071	-0.02089337869823
C	-0.46737730306490	2.80802950716970	0.56089150123913
C	0.83125758503419	3.28051359837832	0.38862231992005
C	1.90986763082740	2.90800883611903	1.19039284219403
C	3.11927758004835	3.34297060951421	0.55980520314843
C	2.77558232154077	3.93790890565972	-0.68005834739654
C	3.75426925203784	4.47457152151606	-1.52352337398558
C	5.08377974709569	4.37414023554632	-1.11768184054777
C	5.44247859987055	3.76425718470247	0.09654715759243
C	4.46720508854910	3.24974200718077	0.94011160312250
H	5.41425343231002	-0.51895086170170	-0.53981138649937
H	5.55001310191363	0.49504806735381	-2.78901369193944
H	3.55887695311989	1.54245497320418	-3.84245474524640
H	1.38394616553520	1.63020658689668	-2.60600680740150
H	3.49522853575666	4.93483080081186	-2.47280447569966
H	5.86191185469957	4.77951065680297	-1.76677562323583
H	6.49476407690625	3.69665116113754	0.37859789645896
H	4.73297567758088	2.77503593190252	1.88484986785947
H	-1.27636755237087	3.04227675010179	-0.12794634157097
H	-0.74098363444812	2.53676461797751	1.58068725541387
N	1.37555276135447	3.86812655828448	-0.79447642607796
H	1.81251018143279	2.46206203384588	2.17817782696716
S	0.45571431699220	4.36166241055276	-2.10500806011977
O	-0.57637898418928	3.36287753410172	-2.33881474240872
O	1.38829503087840	4.67654831276220	-3.17145992407489
C	4.63176211882476	-2.34926635803685	1.13459366531333
C	4.11758435697896	-2.93893409681415	-0.02897261968695
C	5.77795454438985	-2.91044755052610	1.70992253600059
C	4.75761051193466	-4.02546576418080	-0.62382107259549
H	3.20813832540058	-2.54839141219948	-0.48798525856002
C	6.42225053948034	-3.99542742511521	1.11398108792395
H	6.16701292749066	-2.51043377421533	2.64799054899952
C	5.91917902702603	-4.55435260630801	-0.06036963517162
H	4.33815002343021	-4.45900460936594	-1.53399570867876
H	7.31799685254078	-4.40953431200165	1.57988760954952
H	6.42178840278538	-5.40300090051763	-0.52699316164089
C	4.70373727202691	-0.18845916963066	2.59750023522493
C	4.08014223108542	0.61162576147513	3.57032243816432
C	6.02864896950147	0.09621317975179	2.25547951968748
C	4.78256434796098	1.61728819303811	4.22931544009320
H	3.03036078801370	0.42808418172312	3.80992647244939
C	6.73300296089253	1.11337581927165	2.90930875917263
H	6.53609781127160	-0.48064682725343	1.48208916460683
C	6.12081987627423	1.86441630936689	3.90953066650523
H	4.28123429283234	2.21486513997115	4.99274399140432
H	7.76968850492145	1.31081779994162	2.63120815936497
H	6.67507544011423	2.64951940036978	4.42653056989986
C	3.02939225206939	-2.12126933784962	2.88166837766054
C	1.96878584042100	-2.89345188061846	2.39003793156722
C	3.40900651393016	-2.28833108033396	4.21350307589289
C	1.26825164394089	-3.76496599587964	3.21337208135565
H	1.68789476692929	-2.79666474270396	1.34177582603889
C	2.71111317929806	-3.17207597809045	5.04658921942216
H	4.25400904235475	-1.73358374752496	4.62187179030702
C	1.63424833269551	-3.90485386811448	4.55733560804224
H	0.43031902450755	-4.33697164987679	2.80498838558957
H	3.02131534870320	-3.28174971618207	6.08728338928895
H	1.08561654537223	-4.58593681446039	5.21032483930999
C	-0.31990072300076	5.84881046582111	-1.55042802377489
C	-1.68162100877266	5.85198882551526	-1.26540512528634
C	0.46301864322060	6.99872650902518	-1.42407087243692
C	-2.27108182274180	7.04171356314310	-0.83619735854280
H	-2.26926871298494	4.94016663847431	-1.39148797076300
C	-0.14284048010665	8.17206079759807	-0.99290637957999
H	1.52788189893291	6.97379601011859	-1.66564116531711
C	-1.51474033809293	8.21113491083090	-0.69230560287108
H	-3.34148730753789	7.06450064906281	-0.62091578467440
H	0.45520906154877	9.08022592881792	-0.89336266224694
C	-2.14930066912168	9.48753888520705	-0.21224380370235
H	-3.24332636814000	9.43865112440524	-0.28283887293259
H	-1.79238535810969	10.34705024793141	-0.79598694660421
H	-1.88357015429997	9.67266219823498	0.83989982373657
H	-0.24576355379159	-1.20656105917504	0.14882898104050
N	-0.47219763239936	-2.19969942914437	0.23141816148675
C	-1.38534277470490	-2.49393944780483	1.21320828104377
C	0.15453700207375	-3.01249113596569	-0.70846542928085
N	-1.34416703222310	-1.60932285315423	2.22073437105727
S	-2.45630711740509	-3.79544531256334	1.11987691877947

C	0.74601925033398	-2.37362968398110	-1.80869553769038
C	0.30925328284066	-4.39838128783694	-0.55831426456392
C	-2.47120343676744	-1.20822721158688	3.04020499552697
H	-0.50882065052839	-0.99910296956948	2.26346818759112
C	1.45676954283681	-3.11113073164385	-2.74704590482875
H	0.64154487555263	-1.29394523039068	-1.92548484272568
C	1.03287786458336	-5.11055356315233	-1.51009418255341
H	-0.12480687633749	-4.91062682311112	0.29768356071042
C	-2.85971751097712	0.24069099003242	2.64473234825459
H	-3.30325320725386	-1.87503431060729	2.77877877445918
C	-2.19859447286044	-1.39754620019913	4.55162657933367
C	2.10040868034133	-2.41847130547049	-3.91795509249531
C	1.61106978677798	-4.48909797240952	-2.61586596757808
C	1.20264408087930	-6.59945977920893	-1.37092410343788
N	-2.68504132291538	0.48828804981780	1.21916735299648
H	-2.23398136093816	0.96181197222701	3.19478393019130
H	-3.90303594123529	0.43474308974531	2.92949715151310
C	-3.35121332901530	-0.77879120689289	5.35077103112442
C	-2.13938879798153	-2.90238152515413	4.83410414074311
C	-0.86677747098958	-0.75996922182835	4.96425561766181
F	1.90372065900181	-3.09970819822834	-5.05398103205162
F	1.63530374511094	-1.18734804730253	-4.10424364166175
F	3.42589453667174	-2.32535889248933	-3.75513082553077
H	2.16626280265529	-5.06601428265090	-3.35837379386417
F	2.49252132176795	-6.94868010287011	-1.41469122231953
F	0.70200028654502	-7.06685267762419	-0.22877133451211
F	0.59523474414021	-7.25249875732651	-2.36789992180793
H	-1.70908093014867	0.55413678407735	0.89053807029920
P	-3.79599575470484	0.61170411154362	0.01140483986629
H	-4.32353864148441	-1.16014441630473	4.99863273439345
H	-3.36124526383149	0.31878602703364	5.27586559761832
H	-3.24894273931123	-1.03917234384876	6.41478543365331
H	-1.88450150092475	-3.08314992958448	5.88974325164776
H	-1.37635525441156	-3.38331794451192	4.20436907884874
H	-3.10761135119028	-3.38142735441326	4.62129284829518
H	-0.83314981751617	0.31575722303548	4.73239716034061
H	-0.01929561846237	-1.24195864361754	4.45308375202643
H	-0.72353358690266	-0.87554184928523	6.04934040594649
C	-3.12236529522153	-0.22154435315821	-1.42949144327625
C	-5.34246202982787	-0.10602546050665	0.58191075998675
C	-4.13831070458252	2.32702064815747	-0.40694109414549
C	-2.13706569679102	0.42398825861784	-2.20151957031038
C	-3.43774675560368	-1.55794674615335	-1.69625083167395
C	-5.36992716328477	-1.41985474707754	1.06906617922497
C	-6.53334585880056	0.64248260540494	0.57026033389925
C	-4.10709011902320	3.28485025173867	0.62094626067767
C	-4.60739841310577	2.68883418710668	-1.67298943486499
C	-1.48644835707478	-0.26550891820355	-3.21196258575799
H	-1.85248462769639	1.46031720178964	-2.00302739150910
C	-2.77583310182270	-2.26073652598701	-2.69896666675551
H	-4.20241621939302	-2.08034847018459	-1.12104494481578
C	-6.55066702644577	-1.98693229243566	1.53803035091171
H	-4.46345578339222	-2.03111096548964	1.08088274268413
C	-7.71322276929438	0.08512818744900	1.03446702168280
H	-6.54228815352988	1.66954793991099	0.20126035406692
C	-4.56112068434487	4.57223702158123	0.38832588107775
H	-3.73045935132038	3.01374380643519	1.61077478814582
C	-5.05593017877711	3.98564630335858	-1.92084486377991
H	-4.63869729627236	1.95435350642034	-2.48129362052628
C	-1.78438932382869	-1.61924536457933	-3.45418627937641
H	-0.70971201704044	0.21352613549351	-3.80994603236524
H	-3.03012383511673	-3.30519681933471	-2.87000044225646
C	-7.73428421484325	-1.23247239904376	1.52434263863474
H	-6.53578217129008	-3.01147371702681	1.90689861169537
H	-8.64336816843109	0.65399575719480	1.03323240697300
C	-5.05292768430119	4.92722450207162	-0.88145431820887
H	-4.55152323361460	5.32746524045782	1.17520063015108
H	-5.41408455436532	4.24682817457517	-2.91520631798377
O	-1.06632233596824	-2.23157848376437	-4.40715289880685
O	-8.91626444037639	-1.68813689887885	1.95523809840114
O	-5.48479830125471	6.19084475195209	-1.01620575715123
C	-1.27219808922354	-3.61482996064533	-4.62265708586022
C	-8.99620437444919	-3.00907149967199	2.45681053022546
C	-5.92421110096325	6.62930331814442	-2.28865525431267
H	-2.29617223753565	-3.81151657085861	-4.97556875782995
H	-1.08687225247318	-4.18645159799440	-3.69805828573768
H	-0.55158679594057	-3.91808722830134	-5.38885578889142
H	-8.34622574191478	-3.13677465846997	3.33577774910010
H	-8.71532838124600	-3.74140279552251	1.68463425270318
H	-10.03959805523042	-3.16657628664638	2.74765470147941
H	-5.11523732437447	6.54814885380635	-3.03118465680585
H	-6.79403658920962	6.04636428395475	-2.62704284763929
H	-6.21139408663281	7.67909390228539	-2.17131642213170

RS_0043_TS

Frequencies, energies and thermodynamic properties:

Lowest Vibrational Mode (1/cm) = -340.29
2nd Lowest Vibrational Mode (1/cm) = -30.14
3rd Lowest Vibrational Mode (1/cm) = 2.3
4th Lowest Vibrational Mode (1/cm) = 12.29
CREST Electronic Energy (a.u.) = -286.55850941
xTB Electronic Energy (a.u.) = -286.55954028
DFT//xTB Electronic Energy (a.u.) = -5585.42973479
DFT Electronic Energy (a.u.) = -5585.47365211572
DFT//DFT Electronic Energy (a.u.) = -5590.873232674765

Gibbs Free Energy (a.u.) = -5589.614658
Substrate Energy (a.u.) = -2466.962082100482
BIMP Energy (a.u.) = -3123.681057816492

DFT optimised cartesian coordinates:

O	1.06201668940192	-0.33885979953437	2.40197241828684
C	1.64174657212424	-0.21246921071342	1.31422025063977
N	2.91293288888583	-0.69640442174761	0.98273722846485
C	3.81851709227422	-1.39578514458173	1.91490240130265
C	3.28291306834299	-0.16294208070058	-0.27248697820089
C	4.49622386204744	-0.21423235470085	-0.95228087539781
C	4.58943717685812	0.41796952738411	-2.20133283114879
C	3.49715920540837	1.08878826759676	-2.75763197114887
C	2.27983733965372	1.14867403917633	-2.07532132633840
C	2.17265695790759	0.52903217326329	-0.82912642818737
C	1.10003459263001	0.44145842859381	0.13227595002532
O	-0.13676705310813	0.77349496875486	0.04719212429545
C	-0.47569771707348	2.59861464840835	0.95410486094113
C	0.76850652992298	3.13834892691977	0.65728378398290
C	1.95700637549579	2.74567757448659	1.27476310486143
C	3.05741813568950	3.28553798606277	0.53410011692821
C	2.53018393736439	3.97810891034254	-0.58366211516983
C	3.36026212758848	4.64638935599184	-1.48819352192921
C	4.73620152715999	4.57016580714917	-1.27670092857738
C	5.27793004442584	3.85359367539983	-0.19619954664127
C	4.44705145924319	3.21199497259683	0.71411031401956
H	5.36140889360608	-0.72967010334914	-0.53997936650755
H	5.53719031187135	0.38156722145177	-2.74082833102113
H	3.59835727225251	1.57592860982581	-3.72925028472722
H	1.42805979150395	1.69494063827281	-2.48827923729454
H	2.95472415486629	5.18950083120326	-2.33829144368372
H	5.40427795753824	5.07715374273601	-1.97501452443163
H	6.36083950509734	3.80455684097759	-0.06991705530940
H	4.85707010349409	2.65426564993849	1.55744381291159
H	-1.37579751324734	2.87389081561012	0.41471223608421
H	-0.57721830804897	2.17062244458424	1.95205086537171
N	1.13123390973292	3.84671641669272	-0.53411174885611
H	2.00446438186340	2.22808328846250	2.23008112592939
S	0.06665446027591	4.49831532730778	-1.65184696917687
O	-1.14151937367383	3.69227391895684	-1.62174618710198
O	0.78411906175443	4.64038878208331	-2.90674306088863
C	4.59636270905072	-2.51934333047257	1.19814407629927
C	4.10643184896794	-3.11624636114266	0.02990082180715
C	5.71600096838575	-3.09075474178544	1.81625243498371
C	4.74331734797532	-4.22508487780857	-0.52830573891714
H	3.21871899563808	-2.71433902516661	-0.45922007615508
C	6.35624814981272	-4.19688397135193	1.25803884765303
H	6.08552851718138	-2.68007507179694	2.75797797258155
C	5.87622220382763	-4.76644215498234	0.07829179476231
H	4.34481058289164	-4.66509641535278	-1.44447501747716
H	7.23023841230787	-4.61974322378785	1.75626022556849
H	6.37563811779442	-5.63233604346026	-0.35916677962924
C	4.69086993857617	-0.30294953908893	2.56151653436038
C	4.07401958495823	0.57211696428859	3.47207535043952
C	6.02227975723208	-0.06238732992521	2.21085650243186
C	4.78429891861210	1.61688082734679	4.05743714081326
H	3.02217192555141	0.41658481280106	3.72334191039610
C	6.73565135971993	0.99185617869898	2.79196546340888
H	6.52669692855252	-0.69976009893293	1.48454536068940
C	6.12665014559809	1.82451465259191	3.72724014720032
H	4.28615721405493	2.27511626395800	4.77150779936649
H	7.77614191596690	1.15543138204690	2.50633989236485
H	6.68693356289133	2.64104455608372	4.18570469687511
C	2.99977014174389	-2.21023148804761	2.94944259872835
C	1.94647264701398	-3.01128451433650	2.48746996742101
C	3.34496237201853	-2.27934609094509	4.29818927204685
C	1.21154992790941	-3.80686792317699	3.35604251660910
H	1.69280152861163	-2.99000389928850	1.42737927951565
C	2.61379162013027	-3.08908402267661	5.17739799361155
H	4.18335433716867	-1.69981980385168	4.68543645675593
C	1.53841931641008	-3.84254399802798	4.71725519224230
H	0.37854707499924	-4.40123751287862	2.97066320159705
H	2.89475823346148	-3.12162105096484	6.23157585257686
H	0.96253671052548	-4.46275887201621	5.40644354449765
C	-0.29533267612100	6.11322793111357	-1.01651142520445
C	-0.79693498606753	6.24574881707480	0.28181414746576
C	-0.08307852116485	7.21998632952885	-1.83271455314578
C	-1.07378637489815	7.51943997594504	0.76372507035749
H	-0.95980767024038	5.36812253420312	0.91187300044858
C	-0.38286695369301	8.48859425311190	-1.33461776238947
H	0.31536556353621	7.08856044645538	-2.83947881213622
C	-0.87856760076195	8.65780302755144	-0.03698694855388
H	-1.45517531582489	7.63669375748027	1.78028309381005
H	-0.22067203650893	9.36346370714929	-1.96729193393843
C	-1.22530761500506	10.02321780590331	0.48942836126389
H	-2.31689239071989	10.16761504547222	0.47807922615853
H	-0.77258307406082	10.81305762410771	-0.12288558952380
H	-0.88991378598650	10.14091975085809	1.52886030473409
H	-0.28025015894619	-1.28936067334140	0.16599236959139
N	-0.52178194120352	-2.27652475347759	0.27257534725275
C	-1.50325614966702	-2.51480545610404	1.20021279375158
C	0.13212134162737	-3.12860994512062	-0.61191077204668
N	-1.52350806117450	-1.57617322364897	2.16011321781808
S	-2.58038246317811	-3.81032699910686	1.10291767555297
C	0.79647778341145	-2.52962859759143	-1.69262237417844
C	0.24214889787480	-4.51458082473702	-0.42665840923306

C -2.69408970462666 -1.16482992008356 2.91025857815449
H -0.68701405853762 -0.97265436562406 2.22442445813524
C 1.52364191310263 -3.30511854913259 -2.58597278636207
H 0.73189099154480 -1.44942794916550 -1.83198525467890
C 0.98912675622847 -5.26510904620056 -1.33000916866227
H -0.24368084002816 -4.99684933070661 0.41882700230096
C -3.10736481517050 0.25736810475898 2.44421767975509
H -3.49675818200970 -1.86376638287575 2.63943271291031
C -2.47815730014119 -1.28839497131203 4.43761867070893
C 2.23507218571396 -2.65484535081570 -3.74160945885053
C 1.63058484993887 -4.68395031314676 -2.42267682762326
C 1.11239035049978 -6.75453024072866 -1.15417816670924
N -2.83187796626726 0.47230574281794 1.03114505415753
H -2.55948974832757 1.01897200430796 3.02196853745664
H -4.17691625683055 0.41326231684132 2.64137217063477
C -3.68682521467467 -0.68872723325616 5.16471819257526
C -2.36687636513651 -2.77770149032438 4.78050918256908
C -1.19009267761301 -0.58169791405793 4.87576590782510
F 1.85939705113811 -1.39346360539664 -3.92909686019296
F 3.56148479727930 -2.65220724678641 -3.55353937102437
F 2.01650573373595 -3.31675626755965 -4.88409228155237
H 2.20045822763667 -5.29151606851884 -3.12879453887491
F 0.58990221649454 -7.17950382381582 -0.00532844440130
F 0.49275801489253 -7.41276819958018 -2.14050479180228
F 2.39156085753500 -7.14247634607561 -1.17973407263158
H -1.83451227196763 0.50320865731467 0.77637179985843
P -3.83216004578796 0.56288463278279 -0.27312697436607
H -4.62684394403957 -1.12654376131211 4.79157795014059
H -3.74035865072135 0.40327313350074 5.04202558406967
H -3.61700630439376 -0.89961662266727 6.24222936674616
H -2.16412980863827 -2.90725676609383 5.85474161837517
H -1.54713024221462 -3.24449863510795 4.21360229689999
H -3.29815299904470 -3.31027528742791 4.53370926198064
H -1.19217069578998 0.48684503980934 4.60938326929896
H -0.30599231433961 -1.04571846058700 4.41257919826974
H -1.08188170630001 -0.65442008256328 5.96847283710429
C -3.04920960374300 -0.35376469223967 -1.60838929135860
C -5.43353482629313 -0.10285626786502 0.18525534074837
C -4.03106209079587 2.25672092923395 -0.83975398515543
C -1.99353313312570 0.24814036727069 -2.32199409015810
C -3.37539684372641 -1.68918466157463 -1.86100226203339
C -5.53044855161977 -1.38759082797880 0.73722414169059
C -6.60093397305502 0.66110544365216 0.01445426617811
C -3.88238303171665 3.31286465861102 0.06229055341049
C -4.34155373922117 2.53221127580510 -2.18303422112386
C -1.28985454973385 -0.48249707456519 -3.26520267257607
H -1.71050212777264 1.28623209432748 -2.12675326882911
C -2.66162013514814 -2.43327978463060 -2.79766453398422
H -4.19207593930597 -2.17742799980586 -1.32821714057593
C -6.76341093168943 -1.91171415531944 1.11244357058354
H -4.63874287445774 -2.00686082102926 0.87333175818945
C -7.83216186880458 0.14586178770107 0.38482536127704
H -6.54628820744028 1.66667557088008 -0.40707176166764
C -4.00568645999879 4.63515703919314 -0.36048577393925
H -3.64510137206894 3.10649797788310 1.10928921015843
C -4.47054981950823 3.84042359278000 -2.61158786840531
H -4.47053965225407 1.71779823878510 -2.89966121798182
C -1.60755342941073 -1.83326755242212 -3.49919480453779
H -0.46047346418788 -0.03911011117632 -3.81779541287669
H -2.92858814072172 -3.47579212441567 -2.96123370579276
C -7.92578986499236 -1.14364787372557 0.93719414277106
H -6.80665216527058 -2.91392019728763 1.53600606781727
H -8.74705407684904 0.72576983184297 0.26025869563157
C -4.28524451511899 4.90249151486778 -1.70833299774075
H -3.86521165436443 5.44383040994185 0.35567397537695
H -4.69790080674874 4.07724310738635 -3.65140297887667
O -0.84288414552966 -2.48206136460831 -4.39082039047442
O -9.15373453461344 -1.56143474108327 1.26756306447542
O -4.39743543766205 6.13479465103486 -2.22377022586183
C -1.08415704336942 -3.85915679477925 -4.60884758080502
C -9.30752982931548 -2.85092150931832 1.82996208001998
C -4.16034153145600 7.24794536075111 -1.38584888961143
H -2.09291210493653 -4.02271964292396 -5.01804104682663
H -0.97129836343045 -4.43000404954828 -3.67218940948639
H -0.33244880293711 -4.19305970778815 -5.33088542258327
H -8.75332300939920 -2.93661337024460 2.77700434299036
H -8.96209184089951 -3.62900209615592 1.13237711007422
H -10.37780587206850 -2.97942347362757 2.01988887355863
H -4.88226544137603 7.28144415452822 -0.55503402156714
H -3.13728858472278 7.22127579314298 -0.97983548610928
H -4.28249648017637 8.13898931900364 -2.01058207179448

RS_0103_TS
Frequencies, energies and thermodynamic properties:
Lowest Vibrational Mode (1/cm) = -327.1
2nd Lowest Vibrational Mode (1/cm) = 4.64
3rd Lowest Vibrational Mode (1/cm) = 9.44
4th Lowest Vibrational Mode (1/cm) = 15.51
CREST Electronic Energy (a.u.) = -286.55533831
xTB Electronic Energy (a.u.) = -286.56070165
DFT//xTB Electronic Energy (a.u.) = -5585.43099404
DFT Electronic Energy (a.u.) = -5585.475055818527
DFT//DFT Electronic Energy (a.u.) = -5590.872118305303
Gibbs Free Energy (a.u.) = -5589.613354
Substrate Energy (a.u.) = -2466.959478189898

BIMP Energy (a.u.) = -3123.681970126724

DFT optimised cartesian coordinates:

O	1.23526706839655	-0.39330320934786	2.27761900728259
C	1.84749156118150	-0.12311781951189	1.23482951784566
N	3.14388140328534	-0.53384692618387	0.89603902707727
C	4.01810891910552	-1.34534071020841	1.76571998869321
C	3.53919425616751	0.14892864495511	-0.27515730944387
C	4.77777025420784	0.21958229345265	-0.90616372402800
C	4.88763877692897	0.98188703965764	-2.07861967897501
C	3.78691376029455	1.66200497057490	-2.60857017698600
C	2.54613984336082	1.60530683991105	-1.97025004306629
C	2.42450739026533	0.86014034814315	-0.79620204287028
C	1.32508312848098	0.64557876482446	0.11421729104615
O	0.09228044638592	0.98066946882526	0.03133825870600
C	-0.27239030124607	2.73595498079827	1.09524610749162
C	0.99675374105415	3.27228522769482	0.93399686325683
C	2.13889267333593	2.79001964134382	1.57632960324668
C	3.29030675367830	3.40588088322189	0.98791339863491
C	2.84427814771404	4.24207529039572	-0.06417700188167
C	3.73473977716791	5.01562163344091	-0.81314653718747
C	5.09289785149737	4.89687702290074	-0.52192084768768
C	5.55697496900188	4.04207743053427	0.49246365757266
C	4.66400589041079	3.29869808518708	1.25430480998150
H	5.64919531586987	-0.30085678735924	-0.51283815375706
H	5.85559410351892	1.04253139197656	-2.57867211087385
H	3.90073674078646	2.25031091487490	-3.52107672858434
H	1.68471857772428	2.15227138288127	-2.36146612867201
H	3.38732830770784	5.67115915620447	-1.60809767716655
H	5.80846385975346	5.48389550387983	-1.10018454790615
H	6.62838130426855	3.96718892484615	0.68647068891322
H	5.01291689290916	2.63635688684725	2.04735196066619
H	-1.13146401325769	3.08766260216784	0.53338767103219
H	-0.44149665971442	2.19970390840810	2.02921615349754
N	1.44302260813467	4.13320845502421	-0.12238549886498
H	2.11712859940926	2.16390153047472	2.46550293104489
S	0.50166725473864	4.70645624079245	-1.38736216899173
O	-0.62644048848818	3.80152078221511	-1.53537955074256
O	1.37371998082829	4.91977106600295	-2.53063684562207
C	4.86639296087456	-2.34086879939295	0.94748718442812
C	4.46694391638007	-2.77820502618150	-0.32058245211600
C	5.95997561853849	-2.97322451027191	1.55367945017079
C	5.16625794891669	-3.78802288842169	-0.98257400566279
H	3.60332899735414	-2.32661329996567	-0.80857839005826
C	6.66238008744262	-3.98023357960203	0.89306190895915
H	6.25857155738926	-2.69022717303315	2.56493117099780
C	6.27198875651282	-4.38914800300957	-0.38267409359468
H	4.83858758543905	-4.09885020871785	-1.97600558360992
H	7.51419268992051	-4.45280987566145	1.38505647355672
H	6.82061427691581	-5.17714779458725	-0.90107759870267
C	4.82819314851885	-0.34771375265285	2.61576011738531
C	4.14923097235526	0.36570436950383	3.61838254721279
C	6.16950371035199	-0.03617912565833	2.37405747377267
C	4.80922912665484	1.31501564881395	4.39477532644742
H	3.08990416041743	0.15916042915927	3.78762197891640
C	6.83271722562821	0.92103745238409	3.14946568133468
H	6.72113532611946	-0.54489845854055	1.58324219720909
C	6.16171632966520	1.58811553320071	4.17141768962292
H	4.26392824097195	1.84671239048099	5.17655907165108
H	7.88239964537198	1.14048404122988	2.94702117781160
H	6.68233990681946	2.32842774504278	4.78113080128895
C	3.15835470540293	-2.31493856979167	2.61768945828848
C	2.13907342520447	-3.03824460970710	1.98350112605174
C	3.42791513268156	-2.58268165526915	3.95885575134925
C	1.35247978343693	-3.94157342758772	2.68554454663825
H	1.94952750642139	-2.86604540555133	0.92315112900155
C	2.65032371884886	-3.50756613356079	4.66763500790465
H	4.24008870944786	-2.06791433967817	4.47303918732394
C	1.60105709337868	-4.17555782392014	4.04356100097940
H	0.54115928848661	-4.46568343126256	2.17269248773277
H	2.87166610639181	-3.69650785084369	5.71955606293390
H	0.98621587798226	-4.88306807011550	4.60255887278070
C	-0.10199294225747	6.27467040405942	-0.83397784439620
C	-0.66662286161580	6.39385805180892	0.43765642733690
C	-0.07658550308537	7.34358270047168	-1.72528744154197
C	-1.22044102853380	7.61204654438600	0.80903831769219
H	-0.67238504538146	5.54764131649636	1.12897753787581
C	-0.64245779667322	8.55631412571128	-1.33336559081329
H	0.38291652687515	7.22710566639910	-2.70772237019560
C	-1.23158645266193	8.70417790936395	-0.07326635323699
H	-1.66413625831167	7.71857853160438	1.80136199533208
H	-0.62976144306580	9.40136610003754	-2.02494712491638
C	-1.90158300426325	9.99098707366944	0.32092106549160
H	-2.99160572746031	9.89619578692029	0.19109248658827
H	-1.55815468893348	10.82792101756082	-0.30030675817546
H	-1.71597870721722	10.22896106797740	1.37699231817126
H	-0.01453595982236	-1.16877340964183	0.05830737195725
N	-0.38371529784697	-2.11964996951549	0.01508182272524
C	-1.42442167735148	-2.35125533071117	0.87389271151637
C	0.18212169906859	-2.90027778090236	-0.98977172871049
N	-1.41190945823431	-1.50936472593009	1.92238727463814
S	-2.60503451029257	-3.53079790596295	0.61878137985171
C	1.01307123092506	-2.22920886588318	-1.90270911746801
C	0.02920623856344	-4.28760541443675	-1.10671394141531
C	-2.59541806053630	-1.07563093692269	2.64105293056249
H	-0.55160343889414	-0.95201996553076	2.05117588756250

C 1.65122106370363 -2.92783210331707 -2.91707273038376
H 1.13856712187538 -1.14653900321965 -1.82437256590584
C 0.68134170861397 -4.96198661472733 -2.13848238782064
H -0.59870425200286 -4.83465447134357 -0.40777857133769
C -2.96856409963699 0.35836863239623 2.17540484792310
H -3.40511829453864 -1.75232805444151 2.33666199744758
C -2.42477822759334 -1.22286160688135 4.17022005198717
C 2.53243731422125 -2.18542433353426 -3.88421011112722
C 1.50029933635630 -4.30734037638113 -3.05287843042628
C 0.40072371342708 -6.42858619164641 -2.32121473585275
N -2.62122469264869 0.55724804814827 0.77661460913772
H -2.44137129392513 1.10828028899486 2.78775345077897
H -4.04548854517786 0.52339813693066 2.32016286134110
C -3.65145739340627 -0.62719804151930 4.86950510575067
C -2.33089494622628 -2.71865692906231 4.49083613761726
C -1.14818884434590 -0.52804869371528 4.65886189088941
F 2.42570522593452 -2.67777626605635 -5.12138491919967
F 2.24518596124854 -0.88746638126460 -3.93569338548763
F 3.82634358498802 -2.28484125826815 -3.54326496576641
H 1.99073289587350 -4.85155710007547 -3.86089980009464
F 1.40066813407717 -7.05858280625733 -2.93905543436015
F 0.18779755555988 -7.04854703066717 -1.16007579229495
F -0.69636562666590 -6.61635361718032 -3.06811253762281
H -1.61002884950796 0.59222202243270 0.57909736531477
P -3.53229139877026 0.92147833885282 -0.54357346521618
H -4.58189641686597 -1.05949138511226 4.46768333010031
H -3.69888582719308 0.46591304566206 4.75372268787040
H -3.61116032420952 -0.84649474907696 5.94683240285309
H -2.16828997121695 -2.86927173493082 5.56886634239597
H -1.49240846343370 -3.17892121292242 3.94579682760925
H -3.25523182522140 -3.24209193929377 4.20173119910383
H -1.14087488052637 0.54555975308546 4.41362118358834
H -0.25217138925420 -0.98503707113902 4.21313965623721
H -1.07161532160864 -0.62161050237833 5.75254839468572
C -2.70459859490648 0.13636629503688 -1.93249312716655
C -5.18851538720771 0.28879824505581 -0.27955014264426
C -3.61805473384288 2.69289953825554 -0.83681956724485
C -1.56859814343417 0.74603576280069 -2.49905380035936
C -3.08223673343874 -1.14341504974948 -2.35205339979309
C -5.36598619708858 -1.00680444872116 0.22211688084751
C -6.31537617431610 1.07126578930422 -0.58431933707939
C -3.61296101317532 3.54832871372320 0.27890237134017
C -3.67992635556250 3.24110942528785 -2.12032139153379
C -0.83793432888185 0.07937681745135 -3.46961116144486
H -1.24659264033801 1.73690566502076 -2.16871031390528
C -2.34956424150018 -1.81868388947733 -3.32605899595077
H -3.95564437495417 -1.63710066327271 -1.92220211098038
C -6.64208805725353 -1.52570697301863 0.41965992424428
H -4.50091558169120 -1.63585770163052 0.45281732991590
C -7.58850901231012 0.56121818615340 -0.39200791127320
H -6.19522118851677 2.08587564680946 -0.96958865822463
C -3.63353982137438 4.92059018378643 0.10781015953397
H -3.56944419587658 3.13382463656951 1.28966886626325
C -3.69632251544240 4.62246104862880 -2.30378671837396
H -3.69677145349966 2.59147061173550 -2.99833563902929
C -1.21759441988985 -1.20917847033765 -3.88602678103372
H 0.05089460380847 0.52931454631194 -3.91360017349678
H -2.66353442839663 -2.81525408828735 -3.63204472356250
C -7.76413128486644 -0.74043691751955 0.11085737813730
H -6.74937747995948 -2.53671165120146 0.80961871010345
H -8.47492855281556 1.15346201843929 -0.62089055223744
C -3.65151149215796 5.46964066700566 -1.18787188609267
H -3.61719675719237 5.59969023308931 0.96078341095650
H -3.72563210247846 5.02606778690617 -3.31453144303128
O -0.43750577783545 -1.79042297968352 -4.81078589527178
O -9.02891040693135 -1.15139642936015 0.26546464437079
O -3.62674121739757 6.80787439165661 -1.26246211860116
C -0.78519195050903 -3.07887819047325 -5.27931236246014
C -9.26601025103161 -2.45371516790705 0.76519720796742
C -3.60243461700874 7.41825555018621 -2.53642780522493
H -1.76468751762844 -3.06174166063436 -5.78172530302216
H -0.81074914963301 -3.80890510400097 -4.45452341656299
H -0.00675846043810 -3.36670313046160 -5.99351791912726
H -8.84348243843707 -2.57146022836916 1.77472626422672
H -8.83614131995328 -3.21634634500615 0.09809118471403
H -10.35296581446040 -2.57566779421951 0.80728909041613
H -2.72384820008515 7.08956618632435 -3.11339033700799
H -4.52031572530580 7.19134195064365 -3.10117884217219
H -3.53481724078839 8.49766124735099 -2.36134582598372

RS_0111_TS
Frequencies, energies and thermodynamic properties:
Lowest Vibrational Mode (1/cm) = -339.85
2nd Lowest Vibrational Mode (1/cm) = -51.06
3rd Lowest Vibrational Mode (1/cm) = 6.89
4th Lowest Vibrational Mode (1/cm) = 13.11
CREST Electronic Energy (a.u.) = -286.55507852
xTB Electronic Energy (a.u.) = -286.55945684
DFT//xTB Electronic Energy (a.u.) = -5585.42810503
DFT Electronic Energy (a.u.) = -5585.473495590256
DFT//DFT Electronic Energy (a.u.) = -5590.873170889912
Gibbs Free Energy (a.u.) = -5589.614062
Substrate Energy (a.u.) = -2466.962091816921
BIMP Energy (a.u.) = -3123.681682000196
DFT optimised cartesian coordinates:

O	1.00734283151483	-0.23705331334363	1.76703459671889
C	1.79609956452826	0.00269748913030	0.84170608830425
N	3.08176038718476	-0.52733564337000	0.68015923625133
C	3.74338652673196	-1.43971120527002	1.63298167264035
C	3.71628651870028	0.15169012460795	-0.38410343296363
C	5.03545299199402	0.10315897821845	-0.82477551265773
C	5.40191003627094	0.89133358153039	-1.92579247472112
C	4.47257213632749	1.71295826079015	-2.57001978684817
C	3.15072346463330	1.77422030230340	-2.12296357588963
C	2.77251238580919	1.00095054370584	-1.02339246581172
C	1.53093081561635	0.86594877124162	-0.30005990805318
O	0.35771243659738	1.31973870451185	-0.55026148187108
C	-0.03129917734314	3.01910396458822	0.56564633146853
C	1.28246081030412	3.46540949683307	0.61056054940169
C	2.28612752487055	2.86277530219969	1.37087793885266
C	3.55154098673139	3.39379337199537	0.96155460865564
C	3.31806420083509	4.30031383119994	-0.10184457893449
C	4.36327218521660	5.01236276724992	-0.69743790979967
C	5.65615896705501	4.76232583513188	-0.23972232650982
C	5.90978696926789	3.83444215155815	0.78448367293593
C	4.86462952490883	3.14983946195492	1.39182684302792
H	5.77641886738466	-0.52953687489462	-0.33974488606142
H	6.43406763747847	0.85753272074791	-2.27816445374939
H	4.78454883677481	2.31860555172093	-3.42291870373946
H	2.42445922176047	2.43640306830079	-2.60117487712754
H	4.18325655061765	5.71989630614883	-1.50286031532616
H	6.48856211451095	5.30033945688515	-0.69655396897055
H	6.93639825303743	3.65404740803042	1.10803743511639
H	5.04961010384936	2.42939349762347	2.18938199185831
H	-0.77787519890284	3.45865830205927	-0.08796336882278
H	-0.36804900077604	2.46002783266407	1.43934599191365
N	1.93392916046095	4.30615420868160	-0.34806157291687
H	2.09715864034857	2.20379035421845	2.21504059613540
S	1.17493278251484	5.19594448621974	-1.54839693361262
O	-0.04241292319717	4.49114998316143	-1.91173201614915
O	2.16419252300524	5.46549860471855	-2.57685600281979
C	4.58930552702124	-2.50069868123145	0.89788948941731
C	4.31715419070587	-2.86456776277564	-0.42640556825576
C	5.52397738444867	-3.25806851969469	1.61576422941357
C	4.98998176290251	-3.92776810032608	-1.02913006842830
H	3.57471026898409	-2.31348608868679	-1.00370032824703
C	6.20032837879876	-4.31881916740555	1.01427570825036
H	5.71472382013029	-3.03151789122280	2.66660466701308
C	5.94056698623704	-4.65599217929963	-0.31458986984944
H	4.76421247247415	-4.18188065971341	-2.06660044905150
H	6.92756241772721	-4.88994045501924	1.59382007539991
H	6.46854129077258	-5.48630557795176	-0.78621853839444
C	4.52484247857480	-0.54568245418692	2.61456683847163
C	3.78702178215756	0.21677455971634	3.53618438237289
C	5.91106479210860	-0.37148536327846	2.57065149796361
C	4.42256377631228	1.07829855405206	4.42670989033442
H	2.69916558677944	0.11708943368340	3.55341553986082
C	6.55090576185626	0.49934217834432	3.45963184288202
H	6.51465200906910	-0.92138041201740	1.84836793149343
C	5.81351509944515	1.21430756905691	4.40002979527958
H	3.82870026413425	1.64932773319934	5.14255427729268
H	7.63524420134096	0.61242816830816	3.41079887988520
H	6.31511055690370	1.88615509148391	5.09848110066593
C	2.69261919005416	-2.33600531348177	2.33845762524863
C	1.71015372177716	-2.95946554928273	1.55739351617873
C	2.76321413546034	-2.65322141534319	3.69423871240008
C	0.77767224998256	-3.81896240254876	2.12295014085199
H	1.67249684490724	-2.74837110795947	0.48848412309243
C	1.83346359222700	-3.53039551492457	4.26782434954960
H	3.53902728953131	-2.21820689141358	4.32473769346673
C	0.82982675950983	-4.10324632226709	3.49310449757217
H	0.00462545626326	-4.26859784391753	1.49383673030467
H	1.90175265167446	-3.75884367543737	5.33290944557288
H	0.09851923620575	-4.77578446019125	3.94478997976991
C	0.74986056678149	6.72047197522166	-0.75030206215729
C	-0.06064451081129	6.69813735804028	0.38870029929703
C	1.23357404792721	7.91384263402619	-1.27786186749272
C	-0.37306955967442	7.90154006191564	1.00963451809017
H	-0.43325183092279	5.75385981032443	0.79216238554022
C	0.89433361913586	9.11207180756393	-0.64825008954363
H	1.86894095549696	7.90193337780495	-2.16421571679910
C	0.09203875683946	9.12512271201828	0.49835286756099
H	-0.99606401989658	7.89608467428229	1.90656922483139
H	1.26768032516240	10.05411233389705	-1.05459471849516
C	-0.29535915785851	10.41955529147252	1.15872450777049
H	-0.26342066330152	10.32862870494181	2.25288627871273
H	-1.32553623036355	10.69239907462201	0.88110613901794
H	0.36625017356840	11.23820152312842	0.84895362027457
H	0.07540639013254	-0.74714083397736	-0.75778439569275
N	-0.28610962568899	-1.69708930113417	-0.85828565885194
C	-1.45497404766186	-1.92548554910871	-0.17937556745152
C	0.44276458951977	-2.49667533457022	-1.73340127882803
N	-1.58240993209531	-1.11786728387714	0.88664054216040
S	-2.60722619969161	-3.05766623623689	-0.66862608086661
C	1.38541199295839	-1.84363395648856	-2.54255355770011
C	0.35670672482613	-3.89507190857457	-1.78224495702882
C	-2.83965632609165	-0.66860065068140	1.45271463056323
H	-0.72551530578698	-0.62486055207741	1.18921831789806
C	2.19721388219805	-2.57127553240685	-3.40150404515217
H	1.47093559907118	-0.75614676933246	-2.50005772596350

C 1.19320374187556 -4.60021081294884 -2.64448634117409
H -0.35080091761720 -4.42569082602082 -1.14890499270131
C -3.04988387999118 0.82618058434194 1.08512601719883
H -3.62735101338379 -1.25653326263602 0.96272421851752
C -2.92848999274633 -0.95982120646848 2.96972894099878
C 3.20318826883799 -1.85835815243179 -4.26362927670605
C 2.11833583706322 -3.96086223958430 -3.46665016519093
C 1.06642817250515 -6.09661165248667 -2.73887077352529
N -2.47357662248478 1.15251203575434 -0.21041382829321
H -2.58478910293685 1.47299831775784 1.84635134891019
H -4.12418506567767 1.05707443750274 1.08215998457760
C -4.21446586792945 -0.33591682512779 3.52338526794345
C -2.97926980280350 -2.47977977589036 3.15897567031507
C -1.70823387021717 -0.40874657412003 3.71758440894222
F 2.99316086232266 -0.54623425844097 -4.31404551963795
F 4.44944408468987 -2.04365492670169 -3.80638071448069
F 3.18760670302982 -2.31988424441809 -5.51917902914760
H 2.75738040289484 -4.53032248696099 -4.14400202771558
F 0.46358142533698 -6.62310150587872 -1.67325865351570
F 0.35237023142457 -6.45805449262407 -3.81179803310705
F 2.26039039652369 -6.68305364254913 -2.85683507999301
H -1.44521690855751 1.10997631125191 -0.25749133598580
P -3.16817504781115 1.52453708857351 -1.65434216277239
H -5.09146697094221 -0.65876749936485 2.93945494279349
H -4.17606551870266 0.76353740090448 3.51146514164452
H -4.36440145024263 -0.65230097581634 4.56634237860257
H -3.00095418046469 -2.72972483764875 4.23069447725358
H -2.09426220315814 -2.95565711685185 2.70956908167230
H -3.87520262150245 -2.90718169215258 2.68317279693466
H -1.59161546709921 0.67761343691331 3.58002945213613
H -0.78087589912796 -0.89502133094520 3.37946263413848
H -1.82032510402362 -0.59859759253453 4.79569162118677
C -2.19429506477881 0.70637933547634 -2.92606464167680
C -4.87566089575725 0.97820611795962 -1.62246987227550
C -3.11411958448875 3.28870661109734 -1.99509000986282
C -0.96444197357757 1.27461277353756 -3.31371952842647
C -2.56475991394103 -0.54257112838786 -3.43251236218243
C -5.18131533428830 -0.34166496005432 -1.26397150509108
C -5.91952284816314 1.86354499683410 -1.94123431327242
C -3.10966224514774 4.19953121038002 -0.93565933118539
C -3.08860814253440 3.76546498267106 -3.31724782144537
C -0.13478829803116 0.59818817648124 -4.19253544392951
H -0.64945083805805 2.24227888094465 -2.91356419343715
C -1.72979414305532 -1.23230857047469 -4.30895555956280
H -3.51211218407018 -1.00339836012215 -3.14924451061373
C -6.50034278336443 -0.78235850011726 -1.22201663843800
H -4.38642662496336 -1.05362317716211 -1.02246116295264
C -7.23510510983408 1.43221282503773 -1.90221747461382
H -5.70223494009430 2.89733080907096 -2.21697937782171
C -3.05124619568456 5.57129795630722 -1.17423507007219
H -3.13325708014106 3.83754913876119 0.09574377777843
C -3.03307040090127 5.12479933518978 -3.56473623965993
H -3.10070401058414 3.06780913773201 -4.15773503603992
C -0.50502746328508 -0.66601887692003 -4.68745779015862
H 0.82717981293024 1.01470050336426 -4.49411063600412
H -2.03920047563471 -2.20856619586091 -4.67785138846882
C -7.53852567044659 0.10747971269225 -1.54150056002803
H -6.70532784717850 -1.81468048384376 -0.94234700305999
H -8.05746810626246 2.10588964901752 -2.14450671650613
C -2.99837795978488 6.03792690296706 -2.49581053627192
H -3.03014827303666 6.26324490308565 -0.33329082587084
H -3.00024542820642 5.51515177677453 -4.58236552546598
O 0.37616140415567 -1.27122695648499 -5.49864077377067
O -8.83578695413102 -0.22164581260544 -1.52678946190141
O -2.91736615771245 7.33248494258381 -2.83436779929589
C 0.06831845822093 -2.56436629204408 -5.98370611965945
C -9.20126922981698 -1.54174065955717 -1.17180591821522
C -2.82516016175659 8.30071245008645 -1.80904371615117
H -0.83589864422565 -2.54408703608064 -6.61132002916188
H -0.07924504048997 -3.27229292572938 -5.15138836788080
H 0.92841108780043 -2.88150045320437 -6.58187466070049
H -8.87291394168853 -1.78038122313402 -0.14870285809728
H -8.77074974160051 -2.27221724169246 -1.87349438247546
H -10.29374969539938 -1.58473768713564 -1.22433318830410
H -3.71772148589521 8.28333318711004 -1.16434195058755
H -1.92693534179732 8.13581492224936 -1.19438200849241
H -2.75406141925688 9.27383678868619 -2.30608312590588

RS_0238_TS
Frequencies, energies and thermodynamic properties:
Lowest Vibrational Mode (1/cm) = -314.17
2nd Lowest Vibrational Mode (1/cm) = -46.3
3rd Lowest Vibrational Mode (1/cm) = 13.59
4th Lowest Vibrational Mode (1/cm) = 18.44
CREST Electronic Energy (a.u.) = -286.55246582
xTB Electronic Energy (a.u.) = -286.55558362
DFT//xTB Electronic Energy (a.u.) = -5585.42765559
DFT Electronic Energy (a.u.) = -5585.473895265456
DFT//DFT Electronic Energy (a.u.) = -5590.873496416931
Gibbs Free Energy (a.u.) = -5589.610504
Substrate Energy (a.u.) = -2466.973381030197
BIMP Energy (a.u.) = -3123.672028766619
DFT optimised cartesian coordinates:
O 0.97871862972633 -0.74464683053623 1.39541713357423
C 1.44126523418647 -0.08391677001039 0.45613075760288

N	2.51888266012915	-0.46445179924106	-0.36254275428398
C	3.31864645498757	-1.68942145414895	-0.16277840458116
C	2.89271339653765	0.64527238152482	-1.14634707518486
C	3.97906692592445	0.82119126621550	-1.99877167500424
C	4.10248577940609	2.03499370819273	-2.69260531644934
C	3.15917106584528	3.05377168414813	-2.53186794265670
C	2.08495972852127	2.88988030969152	-1.65278961126163
C	1.94945301204446	1.69235267088437	-0.94792577685520
C	0.98943341392894	1.22754807734258	0.02381852711220
O	-0.08605690938447	1.76986654282809	0.48094206241343
C	0.23533037794151	2.63916935204794	2.33154234696703
C	1.54407570701704	3.05188250061428	2.12378634219929
C	2.61594432569952	2.16728763476073	1.99407159110630
C	3.72798162659921	2.88143121154251	1.44491529597339
C	3.31251141602197	4.21477849465178	1.20296830570799
C	4.17556998187676	5.15755938243487	0.63540369688184
C	5.46235914090545	4.73467555725978	0.30360338258037
C	5.88948125122177	3.41631868410118	0.53596527688445
C	5.02999946696146	2.48577376537292	1.10742895902024
H	4.72615652085600	0.04195914854371	-2.13711653075608
H	4.95105384879408	2.17612665221536	-3.36422127796934
H	3.26990137367091	3.99224786512974	-3.07968792196519
H	1.35561165806671	3.68507549289935	-1.51511826181733
H	3.86405865360127	6.18571186252522	0.46536060025465
H	6.15194068600972	5.45236616784753	-0.14417780679921
H	6.90485365817034	3.12259358565539	0.26429707307491
H	5.34631362319415	1.45800649368410	1.28765127951738
H	-0.59253882067148	3.33858373059824	2.37911453031746
H	0.11683161836291	1.68044608189103	2.83716759498211
N	1.98048706095005	4.32271779756340	1.63352614915118
H	2.61491508161128	1.14895436377060	2.37381902589524
S	1.02786759500509	5.69586403524034	1.47487585859088
O	1.91755341742361	6.83992562068871	1.54815203818646
O	-0.06150433598650	5.55959788638354	2.42417925217894
C	3.81173754562237	-2.29178873987544	-1.49683107255175
C	3.15438353117797	-2.04941291228469	-2.70794230558104
C	4.81819087502401	-3.26694124261814	-1.47252691746276
C	3.51969048310801	-2.73499011343872	-3.86860491861650
H	2.34389898452883	-1.31933376064692	-2.74631894626066
C	5.19275344070551	-3.94400105317812	-2.63179902167029
H	5.30674655546267	-3.51914298716084	-0.52968366723360
C	4.54709164738077	-3.67691195238623	-3.83896293527224
H	2.98487358779721	-2.52756155064649	-4.79634292694584
H	5.98493837845036	-4.69306911288737	-2.58456211598281
H	4.83295987772609	-4.20911071066789	-4.74767782982527
C	4.44969470210133	-1.32743096640718	0.81826422730136
C	4.11110288615082	-1.11445353565775	2.16307582671240
C	5.77511121561584	-1.10968190706606	0.42643475566094
C	5.07593267073788	-0.74607912508239	3.09820084864231
H	3.07491361703488	-1.26016300666358	2.47398126746133
C	6.74625630671585	-0.74264731434667	1.36331834341125
H	6.07189092980044	-1.23549424767083	-0.61483441374895
C	6.40428555971660	-0.57064913251143	2.70313188034308
H	4.78712425624526	-0.60429213878508	4.14120165957197
H	7.77563608027245	-0.58822965137238	1.03535011993236
H	7.16444131231160	-0.29016131964223	3.43407223519719
C	2.40960915506726	-2.84011910850969	0.33893604279397
C	1.19750873861007	-3.06944058290447	-0.32845930002041
C	2.82595752503584	-3.76319710678948	1.29852678492139
C	0.41503191093881	-4.17740958808892	-0.02869422731274
H	0.87155878571447	-2.37074471135551	-1.10427515183143
C	2.03000436342582	-4.87226744785880	1.61217232504940
H	3.77657411493618	-3.63204582706340	1.81635570079510
C	0.81981117435796	-5.08249906819301	0.95929780931695
H	-0.50850417512380	-4.34754945425778	-0.58489000588293
H	2.36781010942109	-5.57144451927195	2.37895572149550
H	0.19747249972459	-5.94422958786120	1.20766156927699
C	0.37318929353803	5.59165386867386	-0.16376631412961
C	-0.71115675641619	4.74529434666920	-0.39816958869951
C	0.98503724479776	6.29752890364767	-1.19712667822812
C	-1.17035095761387	4.59091495586130	-1.69975233754000
H	-1.16136808526678	4.18575693360914	0.42112204857516
C	0.50690153111814	6.12999242453359	-2.49717424417080
H	1.82197375431234	6.96497763828372	-0.98688411723751
C	-0.56459396609964	5.26937980076819	-2.76934637621491
H	-2.00538210293012	3.91381922816251	-1.89151908736027
H	0.97949016297174	6.67550637528705	-3.31625366088708
C	-1.04620033038452	5.03970853967195	-4.17519191624307
H	-0.74643934686200	4.03581682705233	-4.51525398281091
H	-0.62299529510462	5.77660032116760	-4.86911115283614
H	-2.14297554062865	5.08884745541642	-4.22801172702253
H	-0.67650086494295	-0.59512167194463	2.16557272237564
N	-1.49354497056005	-1.06138597150925	2.59405418759588
C	-2.71327090120400	-0.45978565418144	2.49557306634798
C	-1.13795753704450	-2.27681220555008	3.20053079259393
N	-2.65959983171570	0.78432393173110	1.95699268441702
S	-4.14964525944839	-1.16399442808411	3.02936043091170
C	-1.95437637700838	-3.41355639330186	3.25209764190542
C	0.15581752496873	-2.35596583253739	3.74498759560463
C	-3.81180192624107	1.55602115686730	1.50869506508573
H	-1.73955704289805	1.10678356478102	1.64311439919608
C	-1.47363491299569	-4.58829623720469	3.83001335321497
H	-2.95898593745582	-3.39080652950413	2.84116875692651
C	0.60549500380988	-3.53516576615584	4.32495088246085
H	0.80613480377219	-1.48211925435272	3.69681776987610

C -3.51940988361613 2.20573767926855 0.13048478993312
H -4.63333357185276 0.83203650899769 1.38906758619688
C -4.30421157337460 2.64478823622009 2.51654872939152
C -2.34837609903634 -5.80976442909714 3.76298409297202
C -0.20070160440360 -4.67203867567599 4.38009405295061
C 1.97023571123678 -3.58005255122495 4.95545024460394
N -2.60662753286372 1.49829214695547 -0.75798168886537
H -3.09206301182923 3.20856533473976 0.27606829338141
H -4.47859097839747 2.35416204728347 -0.38389571430692
C -4.85353494284474 1.99947976592007 3.79246467538328
C -3.17520541653666 3.59317997581944 2.93186208043817
C -5.44115851894303 3.44436037349941 1.86202519703388
F -1.98142799648761 -6.74130336951797 4.64572355017212
F -3.63152120041668 -5.52147183390650 3.99559923745639
F -2.29591905585256 -6.38236848828839 2.55146794571103
H 0.16091819963909 -5.59841057655290 4.82743363146418
F 1.91660640013454 -3.32523427992059 6.26788356534740
F 2.53979407482008 -4.78178385969857 4.82283861935448
F 2.80429944818498 -2.68339397210056 4.42201243391197
H -1.62409140282902 1.50291210711528 -0.43697206984683
P -3.02620273198504 0.25611422124581 -1.76045188441713
H -5.70748812114490 1.34325091155342 3.57504658074674
H -5.18294691280341 2.79181260390727 4.48255332489231
H -4.08820758787013 1.39301802980709 4.29562836095877
H -2.70543953541550 4.11207179717763 2.08266939036253
H -2.39713131047241 3.04891244806768 3.48609417696784
H -3.57831657045724 4.36972225003602 3.59907397065208
H -6.21802145719543 2.77095889031318 1.46545850866968
H -5.08810266382050 4.08834327338935 1.04386558646358
H -5.91061044565569 4.09578765002032 2.61378535333583
C -1.55560129722032 -0.08791012791802 -2.73235793363912
C -3.56988522427142 -1.24957037230821 -0.94964666789594
C -4.33269275952075 0.83972628187439 -2.85223281510405
C -1.48175378043813 -1.28001976663764 -3.47704194602997
C -0.55692191359200 0.87905749410428 -2.88276390998734
C -4.92212621579571 -1.47862399885262 -0.66313950696035
C -2.60455415146463 -2.16864703133384 -0.49455258688365
C -5.13369995881151 -0.06480995112210 -3.55858375650628
C -4.44838727323972 2.21379361270243 -3.12594052279015
C -0.43275301569413 -1.49043050962358 -4.35456941291523
H -2.25258939842433 -2.04770099286433 -3.37337703355023
C 0.50817398121345 0.67065557486226 -3.75822122230632
H -0.58820066999639 1.81251639114481 -2.31565720748783
C -5.32033132531450 -2.59543657122644 0.06524641859406
H -5.68552147309302 -0.77171774148484 -0.99522266102539
C -2.99487517295844 -3.28537960217801 0.21817272491898
H -1.54099206465993 -2.00269226603221 -0.68807664147381
C -6.05717077181587 0.38240914359389 -4.50029343270160
H -5.04365452740836 -1.13932843801534 -3.38529196443110
C -5.36684139254185 2.66861238274907 -4.05604333398358
H -3.81335405120324 2.93305957035981 -2.60639148354832
C 0.56290155830836 -0.50805947246100 -4.51223319823522
H -0.36151846929736 -2.40697561578206 -4.94103249743689
H 1.28724685326957 1.42991711685033 -3.83193810531124
C -4.34934499560780 -3.49035255940374 0.53219176195379
H -6.37488479289478 -2.73779497585631 0.29533836236588
H -2.26796769659934 -4.00868713166547 0.58722068329586
C -6.18316509436179 1.75721799652932 -4.74979989261661
H -6.66935158888804 -0.34397417824474 -5.03197056479456
H -5.47418778212992 3.73229190035559 -4.27059190382737
O 1.53282197837604 -0.78069669871115 -5.39705298404658
O -4.60831057466388 -4.54800287931941 1.31073004969520
O -7.04540534070921 2.28209418760059 -5.62913815054395
C 2.56823950721083 0.16794085133134 -5.58472554284664
C -5.91963537554380 -4.72236151760275 1.81233870064799
C -7.88844351757768 1.41219334888978 -6.36014834498279
H 3.11465860708739 0.34539505300156 -4.64497260671623
H 2.16193880791783 1.12153479235957 -5.95614598284982
H 3.24560018998894 -0.25903555953542 -6.33167754871778
H -6.63159997306860 -4.92133697679922 0.99695127275400
H -6.24006283410460 -3.83048015230093 2.37298863402966
H -5.87768851310584 -5.58454157381043 2.48503078460496
H -8.50213050111335 2.04740467027078 -7.00663323874719
H -7.29835892910608 0.71933843582596 -6.97915800840978
H -8.54014092246588 0.83602424231007 -5.68577730485282

SR_0053_TS
Frequencies, energies and thermodynamic properties:
Lowest Vibrational Mode (1/cm) = -305.39
2nd Lowest Vibrational Mode (1/cm) = -39.55
3rd Lowest Vibrational Mode (1/cm) = 4.48
4th Lowest Vibrational Mode (1/cm) = 8.76
CREST Electronic Energy (a.u.) = -286.56122514
xTB Electronic Energy (a.u.) = -286.56468857
DFT//xTB Electronic Energy (a.u.) = -5585.43335508
DFT Electronic Energy (a.u.) = -5585.474969530929
DFT//DFT Electronic Energy (a.u.) = -5590.875319673003
Gibbs Free Energy (a.u.) = -5589.616515
Substrate Energy (a.u.) = -2466.968798450678
BIMP Energy (a.u.) = -3123.679723792606
DFT optimised cartesian coordinates:
O -1.82532990656325 -0.67669009981393 0.04370697577885
C -1.90852900994148 0.48417873584532 -0.38805425134551
N -3.07244588303667 1.10384798990328 -0.84866996603180
C -4.40875124865342 0.47242852106860 -0.81608918695931

C	-2.79307833112046	2.47245779877738	-1.06640573230851
C	-3.64349454459475	3.53380745290466	-1.36594177540964
C	-3.08442729200993	4.80664377827084	-1.54483006963105
C	-1.70748524286539	5.01991881144660	-1.42203115382728
C	-0.85467851084381	3.96181264913741	-1.09973591684157
C	-1.39769429936776	2.68993627663873	-0.91221495898287
C	-0.81037561697333	1.43152813544794	-0.51524333519572
O	0.41742025221389	1.07263742074017	-0.40239939916032
C	1.06837520120412	1.05027324408681	1.56985976894247
C	0.19320845849271	2.01889342597563	2.03328532745890
C	-1.20022109423504	1.88403291536108	2.02912580083535
C	-1.77678219748923	3.19034136492579	2.18885497790216
C	-0.71962100764440	4.12747976204658	2.22742886311957
C	-0.95314688312065	5.49659120979988	2.35500762126429
C	-2.28184734358934	5.91912878280948	2.42973879536630
C	-3.34425400801443	5.00355088313829	2.37404823073829
C	-3.10214864116363	3.63893485759959	2.24799073529562
H	-4.71872387543216	3.39570416213628	-1.46150498365148
H	-3.74409893194265	5.64328391384430	-1.78038591150205
H	-1.30053988787493	6.02273293759563	-1.56402245651679
H	0.21860101827202	4.11845767986914	-0.96997449997453
H	-0.13066135356457	6.20972567548315	2.37528057527658
H	-2.49348759385401	6.98561107410916	2.52236253820131
H	-4.37235081420153	5.36691472877604	2.42398239504290
H	-3.92189640133938	2.92268177841865	2.19461758110255
H	2.12890590776045	1.25847954877794	1.43032877998004
H	0.75560534115830	0.01226886208489	1.67739818109825
N	0.50433346785102	3.42345515292416	2.11585539013442
H	-1.72750093734326	0.93320707389108	2.05427905578621
S	1.81623629163050	3.94457595403249	3.04352350326819
O	2.87750780255327	2.96612378366370	2.87784049360785
O	2.05121937162581	5.33295596767288	2.67408289504932
C	-5.28028830783531	0.91502399103733	-2.01089549464959
C	-4.70439763237901	1.33811987580530	-3.21310968589706
C	-6.66617333112749	0.71343416891958	-1.97380210857391
C	-5.49404479424682	1.60105911178866	-4.33245556562962
H	-3.62538787001539	1.46438851929926	-3.28098808606023
C	-7.45854208051451	0.97903473012650	-3.09003824661326
H	-7.13694023738770	0.32804538976076	-1.06772464706866
C	-6.87698856099177	1.43234727310153	-4.27444821795772
H	-5.01881897910309	1.93802039643258	-5.25552847581160
H	-8.53676174752055	0.82091716599767	-3.03264481299503
H	-7.49610980926722	1.64027468069399	-5.14855380247969
C	-5.00726762528946	0.80088777103757	0.56538149785742
C	-4.49694217908189	0.13239469109566	1.69046166163186
C	-5.95391354860030	1.81075942187884	0.76738899572269
C	-4.95260395582078	0.43525736446849	2.97176320756111
H	-3.73760368986777	-0.63980707100644	1.55041381691465
C	-6.41639106364463	2.11190347952286	2.05253205666690
H	-6.35134911394983	2.37261721064697	-0.07781329785777
C	-5.92626039203779	1.42009885843314	3.15761592192958
H	-4.54641194675021	-0.10407535958685	3.82925419604937
H	-7.16251220399882	2.89755053898383	2.18284070288873
H	-6.28984274223414	1.65309585724176	4.15973736110586
C	-4.30572640653456	-1.05045122120489	-1.09357016757019
C	-3.47064281923003	-1.51913256944800	-2.12005909624886
C	-5.16214176508883	-1.95829275906393	-0.47035817383680
C	-3.47173217532064	-2.85923179490367	-2.48854631957038
H	-2.79636313355035	-0.82612806337264	-2.62960087586864
C	-5.16982705049567	-3.30737121586151	-0.84402283994257
H	-5.84028569847390	-1.62415677403855	0.31552477162516
C	-4.32522474828871	-3.76557636619593	-1.84915731324392
H	-2.79046521736858	-3.20377965741366	-3.27056598596169
H	-5.83811429577100	-3.99994782387377	-0.33036147550068
H	-4.32151122817340	-4.82022732336469	-2.13000743578325
C	1.24395652784001	3.87801676986212	4.71487147582074
C	1.23793685585213	2.64399901180113	5.36756478725969
C	0.78074188089994	5.03834703027555	5.33443534632161
C	0.74923777458196	2.58067472605832	6.66901876388092
H	1.61637973570438	1.75216667859946	4.86506638653151
C	0.29952055605567	4.95187605556175	6.63795897949826
H	0.80797457841867	5.99146470240549	4.80513110887796
C	0.27647895867773	3.72836029009088	7.32231605058335
H	0.73776793095805	1.62194544264779	7.19128066601071
H	-0.06514351961765	5.85282327255995	7.13542275538084
C	-0.21645114032990	3.65562373971694	8.74162968978001
H	-1.02548484523609	4.37704327070271	8.91633364547761
H	-0.57672489026457	2.64798473229957	8.98544455212015
H	0.60033748045788	3.89750650083996	9.43940946016278
H	-0.82977012803065	-1.76951855704954	-0.90119749064055
N	-0.31673156498411	-2.54711857245640	-1.35129156063425
C	0.38550281576931	-2.23646907251479	-2.47370742129229
C	-0.55926277749149	-3.76119535629849	-0.70677244268259
N	0.39783989964504	-0.90909797876292	-2.72286682015727
S	1.14246348649155	-3.37425345954208	-3.47118133213745
C	-1.68400769490202	-3.78522704552291	0.13545171356185
C	0.25964404989328	-4.89648879018456	-0.77836570498597
C	0.95146504885170	-0.26097013411153	-3.89707319310881
H	0.06891590290161	-0.29699020815884	-1.97401603018870
C	-1.97444399239848	-4.91675364051971	0.88602529229015
H	-2.32354880011136	-2.90163585068274	0.19135635795425
C	-0.05477985481683	-6.01513264119653	-0.01115825299923
H	1.14177763749460	-4.89468873576486	-1.41120100951963
C	1.82145956564217	0.95327282968711	-3.49838077454715
H	1.59047241135729	-1.01157636171465	-4.38411640239352

C -0.14985415551106 0.14419211560035 -4.91980324619864
C -3.21354249163554 -4.94337268135042 1.73860897940538
C -1.16590118072769 -6.05055187018387 0.82789622262672
C 0.83271845806165 -7.22779431177174 -0.05894158138935
N 2.47137101038968 0.88401501174778 -2.20177630870281
H 1.20303265907188 1.86157688233944 -3.46868660684532
H 2.57796089567827 1.11548784549826 -4.27906973793829
C -0.91731150802449 -1.10832826511807 -5.35531763063301
C -1.14319958290174 1.14086142121255 -4.30586704756915
C 0.52126188838014 0.76855391477267 -6.14938848851628
F -3.66628273269788 -3.71908625086327 2.01390032682973
F -4.20997043814748 -5.60423486665479 1.13548665498044
F -2.99746401153335 -5.56149471886911 2.90350031926050
H -1.39273842740295 -6.93517197048001 1.42588115418249
F 0.15783639075635 -8.31409005016129 -0.45200695950967
F 1.85966028998578 -7.07773227136896 -0.89678196793429
F 1.33989988241305 -7.51617480952590 1.14611716274382
H 1.83679328401755 1.10216678158503 -1.41436571950928
P 3.85989310375462 0.09992015098239 -1.79909086738042
H -1.43529339679800 -1.57253974563348 -4.50204780478817
H -0.24038033210066 -1.85953354897136 -5.78707219296673
H -1.67427991870268 -0.83962614757327 -6.10752192435283
H -1.99928448863741 1.27513680476201 -4.98495401756064
H -0.70370049474454 2.13421491616954 -4.13227633198031
H -1.53164116357863 0.77520837264486 -3.34057944552086
H 1.28295026269784 0.09019031908119 -6.56629788690758
H 1.00293162212579 1.72888064865951 -5.91431383295382
H -0.23077213434614 0.95720941517496 -6.92997900493948
C 3.62635447585993 -1.53207287512867 -1.08106709331336
C 4.85220976979201 -0.05763000837781 -3.28082757513250
C 4.59208173197712 1.13295025001164 -0.52746523002338
C 2.53112711375198 -1.72304453340045 -0.22008859991092
C 4.56376407239932 -2.55553717329163 -1.24160331474663
C 4.55058517957267 -1.07065699943483 -4.20090790103457
C 5.86741053012387 0.86754556478745 -3.57269509367727
C 4.35055665187937 2.51359391108957 -0.54556498447759
C 5.33089272451724 0.57612444872506 0.53031396952690
C 2.38376557173180 -2.91903514688673 0.46072608134319
H 1.77977876545116 -0.93551707098298 -0.10153644391845
C 4.41328095701040 -3.76905319788409 -0.57149292705096
H 5.42806682338419 -2.41876487557857 -1.89511637084071
C 5.24682301206362 -1.16550932702595 -5.40260907266560
H 3.76512738102103 -1.80285250844750 -3.98378656996446
C 6.56803632872382 0.77528943730315 -4.76383246649695
H 6.11308571040867 1.66020261494244 -2.86218802236058
C 4.81807327355502 3.33209975405274 0.47545608648547
H 3.76579950345588 2.95172692376782 -1.35614361730265
C 5.80299693234692 1.38594844620530 1.54916080749640
H 5.51766882590800 -0.49938809981130 0.56865131878351
C 3.31980408668260 -3.95556444457702 0.28616006530925
H 1.54232280020546 -3.08677485387633 1.13454439520858
H 5.15107317423369 -4.55534525982596 -0.72146965745855
C 6.26311921244021 -0.23971657711337 -5.68870338223075
H 4.99603789091391 -1.96093226207204 -6.10249435609847
H 7.36479685222658 1.47840064811141 -5.00814832150160
C 5.53260073520330 2.76618982469544 1.54233003200366
H 4.58494962369842 4.39548921287788 0.45789363103485
H 6.36112220555229 0.97070172325193 2.38876268482054
O 3.08906308384675 -5.08825398377010 0.96743606928467
O 6.99087943186566 -0.25442337783739 -6.81229261576212
O 5.97977558873984 3.46270668175580 2.59210050346988
C 4.07420593820232 -6.10480539821465 0.93827897766545
C 6.72805973801205 -1.24998884406203 -7.78263669768707
C 5.66105946566472 4.83911638162415 2.67849234183121
H 5.03613783269715 -5.72419847014495 1.31495125363560
H 4.20597410005756 -6.49905981330109 -0.08007910010733
H 3.70903942232422 -6.90503507005884 1.58874085129065
H 5.69572175870367 -1.17337664189787 -8.15676597802329
H 6.89354777424273 -2.25678132895723 -7.36966893027263
H 7.42856036736009 -1.07278678730764 -8.60486754269273
H 6.10550747198149 5.40009473470382 1.84180715490700
H 4.57101914712473 4.98464923463957 2.68518193109451
H 6.08775587791181 5.19572469006116 3.62163394465961

SR_0070_TS
Frequencies, energies and thermodynamic properties:
Lowest Vibrational Mode (1/cm) = -333.88
2nd Lowest Vibrational Mode (1/cm) = 8.57
3rd Lowest Vibrational Mode (1/cm) = 12.72
4th Lowest Vibrational Mode (1/cm) = 13.27
CREST Electronic Energy (a.u.) = -286.56037946
xTB Electronic Energy (a.u.) = -286.56302579
DFT//xTB Electronic Energy (a.u.) = -5585.42863779
DFT Electronic Energy (a.u.) = -5585.476016103249
DFT//DFT Electronic Energy (a.u.) = -5590.877579547006
Gibbs Free Energy (a.u.) = -5589.618724
Substrate Energy (a.u.) = -2466.969677993755
BIMP Energy (a.u.) = -3123.676367451109
DFT optimised cartesian coordinates:
O -2.12914199104783 -0.46264286988362 0.39364716455000
C -2.11209977357617 0.64482615922908 -0.17722678265857
N -3.20334793187129 1.24339338825998 -0.80289690893147
C -4.54815956589018 0.64024197582771 -0.87231268715878
C -2.86682481326657 2.58126719870522 -1.09740797101049
C -3.65785040145389 3.62620523810557 -1.57037402564717

C	-3.05978961772469	4.87544677323229	-1.77884434996920
C	-1.70024711790095	5.07771420756296	-1.52058108752988
C	-0.90767972795090	4.03432747518833	-1.03708919519524
C	-1.48810870275085	2.78284618987974	-0.81392628564852
C	-0.97479606955238	1.53571473783204	-0.29477419194198
O	0.22741152916680	1.14469209157136	-0.05492376865432
C	0.59150396442817	1.04985866997112	1.97409476370234
C	-0.20726840481158	2.12682812184570	2.33177287221719
C	-1.60067205280674	2.13136822059836	2.21641876809007
C	-2.05700186419301	3.49016779902280	2.25750949392489
C	-0.91872629020198	4.32646661913268	2.35172305042974
C	-1.02822884786333	5.71888283689996	2.36171171945090
C	-2.30973433129856	6.26226935335079	2.27150816045684
C	-3.45049033496476	5.44776611947986	2.17426962343407
C	-3.33361879802543	4.06185254460576	2.16485960930122
H	-4.71705232849351	3.49233020800834	-1.77969385819602
H	-3.67281043836379	5.69985599128064	-2.14639923008566
H	-1.25716422306227	6.06117358272859	-1.68757414997543
H	0.14893234873992	4.19334700817947	-0.81722808435214
H	-0.15101066852612	6.35865883593187	2.43772802481241
H	-2.42284497123278	7.34771102538018	2.27296911539055
H	-4.43686208253710	5.90891700418490	2.10076256809343
H	-4.21221137931867	3.41965177706005	2.07850772127732
H	1.67921612125366	1.10866670218448	1.97922111309532
H	0.13574690381499	0.06231776619326	2.04190435281265
N	0.21191073362041	3.49464262769863	2.40030599657088
H	-2.21753495512089	1.23666098749550	2.23615611554671
S	1.75936858078351	4.02628797236965	2.75614257002352
O	1.64772320592079	5.21200067932707	3.58873416488287
O	2.49050803626687	2.87560205883772	3.26529501713892
C	-5.24667728640801	0.99499700243528	-2.20142013713485
C	-4.50332576670419	1.26531427354341	-3.35674901912911
C	-6.63614036712899	0.86841599799512	-2.31818499053412
C	-5.13669524607890	1.45275153479036	-4.58485175068800
H	-3.41646437615523	1.33151850953209	-3.30147374048861
C	-7.27147551992254	1.05825315285342	-3.54540807381526
H	-7.23403125795545	0.60300365836844	-1.44455689655500
C	-6.52495357657081	1.36015427691402	-4.68417827750596
H	-4.53621668253555	1.67089216129060	-5.46968587871846
H	-8.35656484892883	0.96141604652838	-3.60919177918225
H	-7.02057068754418	1.51013745515971	-5.64465554485805
C	-5.28692137702674	1.10862562877632	0.39622730014417
C	-4.94496265334738	0.51891747338959	1.62449276699965
C	-6.18384180710238	2.18201265375200	0.40407583767832
C	-5.51379944081147	0.96322372912400	2.81604502008640
H	-4.22305464904245	-0.30020939659063	1.63750883267491
C	-6.75720026018614	2.62771377479416	1.59922469653764
H	-6.45687881377378	2.68070231130538	-0.52578614912999
C	-6.43244495331897	2.01584613923745	2.80758307321439
H	-5.23805443071912	0.48263458731775	3.75636690120158
H	-7.46064161761973	3.46177609917088	1.57729916052979
H	-6.88291583277694	2.36157720569207	3.73939691354583
C	-4.45750635911969	-0.90319053846233	-0.99757991945445
C	-3.49824898551943	-1.48502515806662	-1.84134393217679
C	-5.42119153215880	-1.73087562231393	-0.42057754920579
C	-3.48001187748924	-2.85678537335379	-2.06659390510547
H	-2.73883842202592	-0.85752528407056	-2.31473853720411
C	-5.41289234806844	-3.11111626881869	-0.65501887219460
H	-6.19456329411995	-1.30822020487324	0.22195411044508
C	-4.44202510822608	-3.68123064416825	-1.47174923509617
H	-2.70097111707331	-3.28831251710226	-2.70047584844613
H	-6.16909159482752	-3.73804811881969	-0.18027703426745
H	-4.42600911476898	-4.75925504202430	-1.64156770333777
C	2.45554528164503	4.49701081128768	1.20061234440617
C	2.43461995663496	3.60102551482969	0.13112083177184
C	3.14051144904561	5.70839169315778	1.13250136134068
C	3.13098615334588	3.93799667362003	-1.02635395367059
H	1.86897302856112	2.66410690081758	0.18380808614675
C	3.83955078554954	6.01783450396945	-0.03128833852549
H	3.13395567281349	6.38800496976803	1.98595877666172
C	3.85754427562339	5.13385473842594	-1.11791442336588
H	3.12827216537895	3.24891968479638	-1.87419221645591
H	4.39434608312748	6.95697020530933	-0.09184172914493
C	4.67981782992422	5.45413653010723	-2.33446171810129
H	5.74596980781908	5.48211279286763	-2.05998180757149
H	4.54250080634875	4.70228759650143	-3.12187440916818
H	4.41901235795232	6.44276643394009	-2.73879555261786
H	-1.11189155827307	-1.66876884423747	-0.05231860762143
N	-0.61931003647632	-2.54405210726841	-0.33514760558570
C	0.11028599828783	-2.47609284397736	-1.47743485960427
C	-1.02853261932391	-3.64378032177865	0.42170351017607
N	0.24508512525202	-1.20810373376378	-1.92339809161582
S	0.72932981510326	-3.82743259236238	-2.28599286587259
C	-0.23873547527427	-4.76855754755961	0.67435094862375
C	-2.28876998294880	-3.53492585887714	1.03543861252193
C	1.22237910774110	-0.74964088647642	-2.89375837749702
H	-0.08914610422467	-0.47747353174550	-1.29098846860091
C	-0.71833366756349	-5.76192987368220	1.53194435528615
H	0.74834255596180	-4.85538637004171	0.22188908564619
C	-2.74035696627208	-4.53689898665946	1.88087184023751
H	-2.89856453692576	-2.65083478294696	0.83326303792324
C	2.28231304438942	0.12702989571834	-2.17843890645188
H	1.71933978368277	-1.65107625679106	-3.27896926963099
C	0.55062901199310	-0.03937183102447	-4.09543537991139
C	0.17963975432407	-6.93767572871321	1.80437111589257

C -1.96300713713800 -5.66774076329127 2.14365670698583
C -4.11208098926292 -4.43003379974015 2.48963807380952
N 2.51870185981679 -0.33843644727081 -0.81445958045960
H 1.94499847111825 1.17442561961885 -2.14011476774591
H 3.21989708634731 0.10020387395698 -2.75276486674645
C 1.64019160182675 0.49742889945839 -5.03043786453833
C -0.29884356728822 -1.07042898786147 -4.84591445056186
C -0.35435774307131 1.10918130637423 -3.63396137383733
F -0.34317373895723 -7.78156964964473 2.69282737753202
F 0.43684183833305 -7.63465964453718 0.69500165171557
F 1.36688512982478 -6.53971625381847 2.28540407930734
H -2.31705840461511 -6.44884264791260 2.81684190112321
F -5.00721885232675 -5.16272997113835 1.81561139904655
F -4.12718011858797 -4.87213658991034 3.75030005823048
F -4.56211571853650 -3.17434810930710 2.50007842337416
H 1.77731710358957 -0.02182603233114 -0.18073752143077
P 3.99320974842356 -0.55073879517589 -0.10345650540459
H 2.34628321096462 -0.30099314891970 -5.30942434864500
H 2.20906792825273 1.31884732021437 -4.56936252862921
H 1.18348664048040 0.88624345334080 -5.95261353568844
H -0.81273658842364 -0.59441408992602 -5.69473297083709
H -1.06022502748982 -1.50688752736016 -4.18097433978289
H 0.32542060589721 -1.89058648298660 -5.23211800021669
H -0.80140027184055 1.60553109125179 -4.50911956935424
H 0.18827368955309 1.87262564941627 -3.05558814190466
H -1.17726217350073 0.73881854772697 -3.00142114712105
C 4.94925065145519 -1.62940668111220 -1.17370369183674
C 4.91888643826283 0.96331648885559 0.18284875212497
C 3.62349814306640 -1.37744120822171 1.44780907193495
C 4.26723496269778 -2.60666713004923 -1.90685082974384
C 6.35493193025247 -1.60879836867034 -1.19174459492471
C 4.78310920119130 1.67518368758461 1.38064320332867
C 5.67021540670686 1.52810732825294 -0.86728241086376
C 4.58432246787878 -1.44218920730150 2.47263125535797
C 2.42270743055000 -2.08347404722234 1.58767038553378
C 4.96071266818044 -3.53013220093491 -2.68351701044032
H 3.17655085472776 -2.67137931718304 -1.86798006400164
C 7.05232833877284 -2.52533843882816 -1.96029657418868
H 6.91053471764697 -0.87639102727601 -0.60282594521538
C 5.40321924366866 2.91076952504262 1.55366359106406
H 4.17149122871769 1.28799539647058 2.19871641519368
C 6.28932969041943 2.75380845296568 -0.70354872313807
H 5.77367961447864 1.00629771041562 -1.82141374291557
C 4.33023817946424 -2.17474712564753 3.61895211185885
H 5.53770654457447 -0.91743534722119 2.37428560311021
C 2.15902636775103 -2.82157384774483 2.73917375508887
H 1.67223529044028 -2.06711061926038 0.79432358585718
C 6.36282399153667 -3.48866356976666 -2.71961724530532
H 4.39925333063183 -4.27471010893159 -3.24650351913809
H 8.14220146148795 -2.52156621564095 -1.99288525275235
C 6.16252169205649 3.45609731160359 0.50978702642356
H 5.25806676054082 3.44570920291403 2.49121140109894
H 6.88106738639911 3.19824759386961 -1.50460713045613
C 3.11416586228893 -2.86904700037087 3.76505557597264
H 5.06092639592354 -2.23189362707703 4.42618816770832
H 1.21377489392030 -3.35780362962397 2.82071854283436
O 7.11850452769535 -4.32723987090780 -3.43943700429951
O 6.78831003331583 4.63956447979108 0.57472286345705
O 2.95225630966831 -3.55004919773089 4.90542016481407
C 6.47978458086576 -5.33112441767673 -4.20523369911173
C 6.78651858904810 5.34044113801348 1.80425248489499
C 1.75139207413944 -4.27535862564580 5.09902027951451
H 5.81581399717717 -4.88695870657532 -4.96243746993042
H 5.89783410065775 -6.00758122044943 -3.56094463015356
H 7.27590371428996 -5.89433218723720 -4.70236342960892
H 7.38522238513304 6.24342300324845 1.64690719456185
H 7.23896833972750 4.73037580052961 2.60101777092442
H 5.76451669303589 5.62436539028814 2.09693436833595
H 1.62673126007806 -5.04385999512589 4.32153346464063
H 1.83498104375077 -4.75446611184788 6.07957263234575
H 0.88031433398137 -3.60235710658606 5.09184426912709

SR_0101_TS
Frequencies, energies and thermodynamic properties:
Lowest Vibrational Mode (1/cm) = -314.12
2nd Lowest Vibrational Mode (1/cm) = -35.09
3rd Lowest Vibrational Mode (1/cm) = -2.84
4th Lowest Vibrational Mode (1/cm) = 14.45
CREST Electronic Energy (a.u.) = -286.55978278
xTB Electronic Energy (a.u.) = -286.56372065
DFT//xTB Electronic Energy (a.u.) = -5585.4339117
DFT Electronic Energy (a.u.) = -5585.476394008769
DFT//DFT Electronic Energy (a.u.) = -5590.875756689211
Gibbs Free Energy (a.u.) = -5589.616011
Substrate Energy (a.u.) = -2466.969790723523
BIMP Energy (a.u.) = -3123.677622788045
DFT optimised cartesian coordinates:
O -1.79445520963906 -0.80459873610848 0.13385689502151
C -1.99920030498810 0.42296719925057 0.16510225554066
N -3.19404175492336 1.05658467954502 -0.17984787430465
C -4.42989873789645 0.34798768363236 -0.56890240058622
C -3.10384731087857 2.41329212585327 0.20086961833665
C -4.07898216046976 3.40843775732432 0.22093760424677
C -3.71654849036277 4.69477032378280 0.63921400905930
C -2.40696009545250 4.98097271294577 1.03459406446189

C -1.43323393131997 3.98012987733204 1.03759078391706
C -1.77689894412350 2.68692343262891 0.63246191175660
C -1.04416365005773 1.44185487402049 0.56636967540566
O 0.20524166289359 1.17355253185447 0.72047265171427
C 0.57636222996865 0.27426192415271 2.56193597246460
C -0.38344619480012 0.96517574130480 3.28567648695718
C -1.75918272900081 0.82395866345910 3.07945783548334
C -2.42246586174267 1.94008447595321 3.68786183591519
C -1.43068269427508 2.78929181464190 4.23677754256670
C -1.75702515484073 3.99390066404042 4.86326310926719
C -3.10734403999290 4.33649445354905 4.92994060587934
C -4.10563552263337 3.50599526667837 4.39290017764985
C -3.77295298223424 2.30804284168319 3.77026209814077
H -5.10452565251810 3.21044492688695 -0.08233200431147
H -4.47544422830283 5.47856407018961 0.65600846353614
H -2.14518025720293 5.98995270581344 1.35842226743847
H -0.41856185542697 4.20138658969315 1.36796640148435
H -0.98990182349618 4.64017376920554 5.28559624768635
H -3.38985000089441 5.27458925703649 5.41070800535478
H -5.15184219891437 3.80851796125677 4.46223144836813
H -4.54037225483044 1.66043189308896 3.34187397910398
H 1.64456249518857 0.46872164239480 2.66250825194922
H 0.26449802938261 -0.69470331970040 2.17542495208866
N -0.18240537911402 2.19021290834089 3.99718629075846
H -2.22653887504014 -0.05737887936388 2.64659956399457
S 1.25716515928918 2.66183726771358 4.71881084793251
O 0.94165479753473 3.34823917205880 5.95989994157651
O 2.10731070412555 1.48251897959227 4.76773380038424
C -5.24107958875509 1.16697306177408 -1.59656705539634
C -4.60307320470094 2.05214090462583 -2.47379396751122
C -6.59997957138318 0.89713096684418 -1.79655068918001
C -5.31419618248697 2.68846320183194 -3.48997469123987
H -3.53695627699228 2.24988405996530 -2.36389079092840
C -7.31427208122917 1.53493324609353 -2.81133693126705
H -7.10997156863408 0.16801839058312 -1.16475539575554
C -6.67683524476232 2.44090205167947 -3.65846203009570
H -4.79474337076518 3.38094708902538 -4.15472546267655
H -8.37464160814487 1.31290479944201 -2.94234017326990
H -7.23480846416688 2.94070461026857 -4.45186457068971
C -5.17209321428745 0.03752979995788 0.74471537730941
C -4.68195934512042 -0.99699374178427 1.55825105266312
C -6.22784527381506 0.81763263001364 1.22857664929507
C -5.25170593684167 -1.26177139047468 2.80195508396537
H -3.84093450449966 -1.59690694087209 1.20450492387240
C -6.80617756228751 0.54741426790678 2.47260299279564
H -6.62037245182940 1.64450461188978 0.63714561115712
C -6.32537471148513 -0.49511339636204 3.26144156496837
H -4.85612060320913 -2.07447971026609 3.41368678984758
H -7.63444223796876 1.16522573060371 2.82371617970265
H -6.77718491406785 -0.70564121219200 4.23217708879648
C -4.11607281332869 -0.91351632537669 -1.41234701538572
C -3.12699900457110 -0.86270595662435 -2.40608424487923
C -4.93804598015441 -2.04012988318421 -1.35208156024782
C -2.95722871380161 -1.91113035322029 -3.30304018299767
H -2.47050571516629 0.00845960205938 -2.47092722937928
C -4.77354833820768 -3.09466260429404 -2.25765462807227
H -5.72695041090845 -2.10405859116072 -0.60147857602258
C -3.78686038087703 -3.03571813719637 -3.23682439287085
H -2.15935098259750 -1.85903103120934 -4.04859916235576
H -5.42295749201284 -3.96836564350326 -2.18416564439176
H -3.65282819573909 -3.86218139757774 -3.93689611384032
C 1.94948530117410 3.84002369517947 3.59325238815722
C 1.87000242073415 3.62371778766316 2.21868901061735
C 2.63301243212308 4.93119231332861 4.12639223673496
C 2.47698415306916 4.53920153762571 1.36455843014929
H 1.32400263904147 2.76690206211762 1.81131072134869
C 3.24968462447730 5.82468479406372 3.25273462461804
H 2.67328391373684 5.08129445391674 5.20615553508455
C 3.18269436997048 5.64323823926387 1.86437459462545
H 2.40654317168467 4.38760122553365 0.28441510274729
H 3.78537873184085 6.68572894002923 3.65749162210162
C 3.87197038500511 6.59427832301069 0.92460120455413
H 4.09633921565044 7.54912751782726 1.41656414465368
H 4.82324937026342 6.16065489139916 0.57784867405389
H 3.25549238117433 6.78657703995067 0.03613099308253
H -0.73631946986435 -1.49475164717259 -0.99572901768682
N -0.14850863736544 -2.01964743827575 -1.67109727887222
C 0.61988518937247 -1.27251113643238 -2.50348837453212
C -0.36211131066376 -3.39522300358015 -1.60506058238280
N 0.63753421653966 0.03516020421788 -2.16492679418953
S 1.43635937843339 -1.89799688213350 -3.84924004918094
C -1.56345905812115 -3.80375271078163 -1.00417599223064
C 0.56788392469133 -4.36718814963360 -1.99758461258088
C 1.30553875668384 1.09407697249734 -2.89825315178943
H 0.21667817452168 0.29138957984777 -1.26819466622283
C -1.83381369643255 -5.15602601817796 -0.83267204049337
H -2.27732887204007 -3.04439539627594 -0.67557502188441
C 0.27561415964967 -5.71482083775240 -1.80646694524142
H 1.52431558255617 -4.06694657563246 -2.41574965552253
C 2.20085789746495 1.94943966501075 -1.96332714350776
H 1.94885535086870 0.58844038974978 -3.63138950459660
C 0.30941418280137 1.97826440510377 -3.70340712444436
C -3.12997342740168 -5.58635778935370 -0.20159357692182
C -0.92666622502792 -6.13374567160911 -1.24005384343297
C 1.29718165790515 -6.75496081926505 -2.18041367099325

N 2.63721690590101 1.33173372443503 -0.72141916327724
H 1.66131984517165 2.85791391328076 -1.66205176369454
H 3.08111032900031 2.28577220643813 -2.52951627473699
C -0.73735343020045 2.62339950836162 -2.78657660225288
C 1.10120020597301 3.06662754835213 -4.43988366949515
C -0.41182833047640 1.10971644418556 -4.73917040438688
F -2.93466072554771 -6.55682362507828 0.69756904352595
F -3.74877480312301 -4.58464869891531 0.42407138910824
F -3.98565385168352 -6.07298408137895 -1.10819138690935
H -1.13550018506869 -7.19383743191114 -1.08320748175875
F 2.49120901741472 -6.22260970651502 -2.44070430879897
F 1.46143839643746 -7.65202804253726 -1.20186103753482
F 0.92708883087671 -7.44470847743677 -3.26596485395169
H 1.90251609773810 1.31597944223762 -0.00028552900334
P 3.89929104593582 0.29779023811548 -0.45654056945810
H -0.29572367274540 3.29623634678655 -2.03620714091387
H -1.32039367650305 1.86088365762167 -2.24493489618463
H -1.44099175488629 3.21827603411805 -3.38937977825512
H 1.55032477919811 3.79480899908940 -3.74894271017075
H 0.43243973495777 3.62019760875028 -5.11559987195444
H 1.90579982186868 2.62121077789871 -5.04658115772491
H -0.97084670675933 0.29417436161198 -4.25570462131102
H 0.30140419828682 0.65670327671762 -5.44335785074287
H -1.12696268808558 1.72266851073367 -5.30856629195480
C 4.51061313493778 0.77771825195392 1.16210587877830
C 3.42157558185478 -1.42966964693945 -0.34554132645496
C 5.09017173300156 0.49742609732738 -1.77676940767151
C 4.76600387956612 2.14115848922065 1.40011166950388
C 4.56412359256353 -0.12421045818919 2.23158317072284
C 2.21460879850519 -1.71671608924538 0.29898041744526
C 4.28466977505148 -2.48036074693497 -0.69776469618969
C 6.28644945594280 1.19602197599212 -1.59389193447717
C 4.79309707634784 -0.05420420541651 -3.03884588014506
C 5.04442508196297 2.58986126851757 2.67732077593636
H 4.70563099341104 2.86297794800491 0.58202516443716
C 4.82303300285864 0.32297940480383 3.52451909584019
H 4.36914040500438 -1.18630863445283 2.06991620574028
C 1.87543821894509 -3.02416567266258 0.62923725057132
H 1.51346357449544 -0.91105960205931 0.53497491267861
C 3.93508920967749 -3.79113437353540 -0.40939042987106
H 5.23514709753102 -2.27487353182440 -1.19539431494266
C 7.17932935648602 1.36417532782336 -2.65063136977808
H 6.54380526168889 1.61193157557520 -0.61796149233240
C 5.67231886050575 0.11425375533633 -4.09308900516952
H 3.87344340813700 -0.62945951135105 -3.19499118623520
C 5.03185866663566 1.69056337296067 3.75845615688450
H 5.21059635655894 3.64929502571142 2.88145968120677
H 4.81893663091658 -0.39136692162019 4.34582136538783
C 2.73437621752423 -4.07221008049847 0.26719814559559
H 0.92645810904975 -3.21795473818138 1.12838953570939
H 4.58002289544168 -4.62436840990441 -0.69012944142483
C 6.87262614453238 0.82733431137693 -3.90960684915700
H 8.10721966694955 1.90774061685638 -2.48170146436206
H 5.45965986937767 -0.30553235136616 -5.07686532585480
O 5.21662285303388 2.22391612459801 4.97090429654670
O 2.47126771341525 -5.36484744578030 0.50937678743306
O 7.66489340708626 0.93824304353980 -4.98264873581716
C 5.10703878039651 1.38335762844195 6.10519389694110
C 1.38186452670378 -5.68609895033719 1.35447183747177
C 8.89338508864570 1.62860159190140 -4.85577160868890
H 5.25061514053075 2.02632363443956 6.97973797042757
H 5.88341594030744 0.60302571262578 6.09513773477716
H 4.10954732612519 0.92236551181089 6.14246157534659
H 0.42690154701141 -5.34361881885440 0.92766716643701
H 1.51837622868410 -5.23467440937213 2.34946186175087
H 1.36762618274992 -6.77758306921923 1.43832385587051
H 8.72819047450710 2.67695752109856 -4.56377652695496
H 9.54464274084459 1.14064314571934 -4.11454491061728
H 9.37048496808609 1.59432937488897 -5.84027256943957

SR_0121_TS
Frequencies, energies and thermodynamic properties:
Lowest Vibrational Mode (1/cm) = -315.13
2nd Lowest Vibrational Mode (1/cm) = -40.09
3rd Lowest Vibrational Mode (1/cm) = 2.82
4th Lowest Vibrational Mode (1/cm) = 14.3
CREST Electronic Energy (a.u.) = -286.55930764
xTB Electronic Energy (a.u.) = -286.56372174
DFT//xTB Electronic Energy (a.u.) = -5585.4338662400005
DFT Electronic Energy (a.u.) = -5585.476377355271
DFT//DFT Electronic Energy (a.u.) = -5590.875696404535
Gibbs Free Energy (a.u.) = -5589.616142
Substrate Energy (a.u.) = -2466.969673573146
BIMP Energy (a.u.) = -3123.677814011319
DFT optimised cartesian coordinates:
O -1.72689146225330 -0.69540323307233 0.18034354437921
C -1.97286613101243 0.52496563283469 0.19125486633171
N -3.18449185155640 1.11315612790877 -0.17559239759326
C -4.39132154397813 0.35907597506547 -0.57066266744630
C -3.14499050222976 2.47620965627536 0.19032382459837
C -4.15272403848410 3.43852407826012 0.18366249254077
C -3.83916539536188 4.74080484761353 0.59187485812490
C -2.54541560787092 5.07548517101213 1.00163799856041
C -1.53850691568424 4.10829045146228 1.02907157524117
C -1.83423586528522 2.79934654780964 0.63708718176953

C	-1.05869724072421	1.57970546722139	0.59480711044941
O	0.19592890604911	1.35547466054288	0.77320716547524
C	0.55240353506810	0.44769718305986	2.61440261258529
C	-0.43455095588505	1.10984661816937	3.32807925467591
C	-1.80299633596058	0.94513175973748	3.09053181446446
C	-2.49989189816814	2.04564223332936	3.69063440685997
C	-1.53604521636459	2.90533879681912	4.27181534745959
C	-1.89557985497311	4.09971149159183	4.89947791747698
C	-3.25197383818388	4.42303700415112	4.93237518668582
C	-4.22302118879041	3.58309435400165	4.36136935546080
C	-3.85700367003830	2.39430888713121	3.73927063563012
H	-5.16622965000222	3.20324296549406	-0.13315072005319
H	-4.62361391927566	5.49920548249538	0.58845122354817
H	-2.32213769062164	6.09692367804859	1.31508989136826
H	-0.53468167177089	4.36800722425127	1.36473394890968
H	-1.14912825770861	4.75295698033078	5.34787532235929
H	-3.56082288546996	5.35311971967722	5.41245343326411
H	-5.27486262099445	3.87065744513320	4.40388069853554
H	-4.60366941455117	1.73997929277258	3.28535196920304
H	1.61418294646392	0.66345442847297	2.73559800515242
H	0.26524781127785	-0.52121392615061	2.20892713431881
N	-0.27297519978404	2.32889267795462	4.05933686609594
H	-2.24400474780579	0.05694963632707	2.64519049146666
S	1.13824860017118	2.82309394342232	4.81857810412915
O	0.78464020586040	3.45042601022194	6.08073593851473
O	2.02886699823325	1.67301242431601	4.83458375323708
C	-5.21204969636601	1.13295709395233	-1.62509529064344
C	-4.59214929247477	2.02897061824294	-2.50400580856081
C	-6.55598637548709	0.80843189405928	-1.84485026976748
C	-5.30833430709236	2.62210864449407	-3.54272806323095
H	-3.53648384071984	2.26861121212447	-2.37825254250757
C	-7.27520990725941	1.40270150183864	-2.88204164873540
H	-7.04913642858207	0.06979803556772	-1.21066514150386
C	-6.65700217204825	2.31963109570233	-3.73182743184825
H	-4.80358725017553	3.32403858001677	-4.20891533231328
H	-8.32371519036914	1.13812604267763	-3.02860240365193
H	-7.21873220649450	2.78549505419802	-4.54303038098866
C	-5.14535329965359	0.04926812023619	0.73646631850004
C	-4.63567546902827	-0.95319167819718	1.57759610321000
C	-6.23477995062302	0.80213109920687	1.18753812262022
C	-5.21654455065738	-1.21263882669610	2.81720988662521
H	-3.77033080408369	-1.53266388021970	1.24882975348814
C	-6.82425942326288	0.53692170594111	2.42742531024633
H	-6.64519913616454	1.60315744848550	0.57311811533059
C	-6.32198136011534	-0.47311852717500	3.24456916912571
H	-4.80436766034277	-1.99974584011761	3.45107752267753
H	-7.67837377975092	1.13322915457827	2.75283283199625
H	-6.78216171583328	-0.67940898250959	4.21227029711414
C	-4.02442065773809	-0.90767320904663	-1.38469756992043
C	-3.02550484587136	-0.84362940195701	-2.36783599180586
C	-4.80675600908222	-2.06118699823215	-1.30888217402053
C	-2.80632538407901	-1.90611339506651	-3.23723089620897
H	-2.39979575590671	0.04892338904236	-2.44588183914364
C	-4.59279336348240	-3.12990381888852	-2.18699732166827
H	-5.60270774515144	-2.13669189066619	-0.56698633125121
C	-3.59532548153930	-3.05846958588740	-3.15423403209487
H	-2.00102248647057	-1.84260584319391	-3.97388791330930
H	-5.21186667827715	-4.02417844856062	-2.10124511147591
H	-3.42157052805615	-3.89596824083295	-3.83215287902872
C	1.79387000998552	4.06777685839309	3.74391827690092
C	1.74004536248301	3.89448879036069	2.36132671001841
C	2.42415623708070	5.16730982968093	4.32226907770289
C	2.31555410733110	4.86443385402474	1.54692888397847
H	1.24350131115041	3.02399924543946	1.91983621409315
C	3.01145153422705	6.11647082216989	3.48733224380984
H	2.44997300559505	5.27897028552332	5.40711551467090
C	2.96278887438022	5.98312522456814	2.09319611507757
H	2.26840322395653	4.74642225054913	0.46127117355746
H	3.51108035568588	6.98164580686111	3.92764722171181
C	3.57471462064435	7.02015829313963	1.19142383969757
H	2.78773027744109	7.60972111168098	0.69706938817650
H	4.21540858517647	7.71019306718323	1.75458815338088
H	4.17297531958211	6.54575106399528	0.40085038301766
H	-0.62863522267702	-1.37890439067363	-0.91047938972516
N	-0.01419327330570	-1.89501838253961	-1.56891636811351
C	0.73656262979423	-1.13423577771213	-2.40520619650149
C	-0.18284322488711	-3.27547788221476	-1.48408347688509
N	0.70367618799152	0.17799003568526	-2.08660682619511
S	1.58861227671842	-1.75015889763397	-3.73331394416633
C	-1.37444290179934	-3.71382876925071	-0.88506051924700
C	0.77999091075992	-4.22288298045577	-1.85745142644934
C	1.35029060820822	1.24775786875332	-2.82349226296417
H	0.26722702248573	0.43147351452496	-1.19644241354933
C	-1.60281447010181	-5.07148417750593	-0.69533363831932
H	-2.11465114699765	-2.97277021964899	-0.57312445647743
C	0.52982197798451	-5.57610089252378	-1.64762336121253
H	1.72821444282805	-3.89862033079007	-2.27624536087183
C	2.19153946626805	2.14710810377789	-1.88142324838702
H	2.02877440869822	0.75392610543243	-3.53281063971195
C	0.34329090049536	2.08368825770691	-3.66519831422416
C	-2.89274721968109	-5.53128939286123	-0.07236148025064
C	-0.66185681004235	-6.02547858783194	-1.08187521351997
C	1.58509672805412	-6.58873448110617	-2.00273271974690
N	2.63868633716538	1.55202508172950	-0.63271415745838
H	1.60668182053326	3.03032496920565	-1.58924325499731

H 3.06275940424860 2.52152814452816 -2.43761734854631
C -0.74862453419076 2.70219625141975 -2.78348633654721
C 1.11262101896231 3.19090104077906 -4.39730723492161
C -0.31995049765560 1.17510395934735 -4.70549646147292
F -3.73130425790903 -6.03785369300925 -0.98429003175976
F -2.68233901688660 -6.49620104771645 0.82902744038555
F -3.53774560126194 -4.54225114257256 0.54730081839006
H -0.83732009204363 -7.08947631309922 -0.91145885118389
F 2.76135687196335 -6.02312913925432 -2.27328303933090
F 1.77836573355059 -7.46240803394844 -1.00839440952798
F 1.23719671986754 -7.30901680093062 -3.07563356406875
H 1.90087881105387 1.52100580381167 0.08466955474426
P 3.93713878292407 0.57028184973068 -0.35102689182361
H -0.35068992011657 3.40374633528749 -2.03496732130171
H -1.31397739048953 1.92576466298570 -2.24296535733945
H -1.46023268941906 3.25938477305743 -3.41236709954632
H 1.51651481149579 3.94421755174093 -3.70535288672890
H 0.44122766123273 3.71024522960370 -5.09718633152889
H 1.94856215729592 2.76830411632492 -4.97742835706821
H -0.86388035940347 0.34834518437383 -4.22375035835893
H 0.42707617453499 0.73586531505604 -5.38284166114004
H -1.03971000980203 1.75466216820384 -5.30336103615158
C 4.50438096750257 1.07992161697111 1.27485056085273
C 3.52984223282237 -1.17486337760593 -0.23605772973179
C 5.12940020367884 0.81720370774536 -1.66192944114944
C 4.65743250782871 2.45642529926627 1.52425921194288
C 4.61744696850122 0.17601127683701 2.33817767399618
C 2.33566050875250 -1.50254542306583 0.41307234961004
C 4.42992882806900 -2.19510352504543 -0.58416414757400
C 6.27185431475417 1.60094185392315 -1.47997616992938
C 4.88145712421250 0.22967931175068 -2.91830125975622
C 4.89239621211858 2.91472218447910 2.80674048798490
H 4.54802463082595 3.17758309907361 0.71084112074713
C 4.83317629346173 0.63148402410411 3.63620980163161
H 4.50154621714412 -0.89623565591865 2.16762351917862
C 2.04514763971131 -2.81894304040642 0.75231916036342
H 1.60747231577229 -0.72104508856385 0.64697712793410
C 4.12906337454056 -3.51626099262311 -0.28698169476495
H 5.37166268681904 -1.95826231768605 -1.08436405533472
C 7.16043950236717 1.81588339572858 -2.53191629229884
H 6.48912084657605 2.04920708698576 -0.50846863538499
C 5.75694761373173 0.44339088249764 -3.96768465430490
H 4.00318478702061 -0.40687114829064 -3.07414443325844
C 4.93781577404260 2.00910284595851 3.88130631177600
H 4.97525477519110 3.98228235548736 3.01976844436117
H 4.87658275027139 -0.08739866657066 4.45245607954608
C 2.94141190385847 -3.83692808556329 0.39483056651242
H 1.10588699444751 -3.04475159687136 1.25613534640530
H 4.80386565013432 -4.32683096698793 -0.56384017645824
C 6.90313757572483 1.24051925229589 -3.78504242502935
H 8.04629973025622 2.42588243448016 -2.36397898934951
H 5.58256834077713 -0.00289786553794 -4.94730540503454
O 5.06994448786122 2.54636525838199 5.09891184920712
O 2.72597828237270 -5.13644848061897 0.64726854089792
O 7.69534409876612 1.39020599027725 -4.85348437379131
C 4.99432829889700 1.69491686709988 6.22759023968878
C 1.65459738736388 -5.48928041117748 1.50301159851823
C 8.86547848556918 2.17617547904637 -4.73109278568234
H 4.03042425673326 1.16606129089217 6.24105670193289
H 5.07368120544149 2.34203301094155 7.10725332813336
H 5.82284398697431 0.97012560398427 6.23242643216861
H 0.68497347398417 -5.18561853261926 1.07980579712330
H 1.78146527025595 -5.02378450734879 2.49277455615292
H 1.68088267967589 -6.57970208263059 1.59668590357965
H 9.54754026607465 1.75280968454123 -3.97798691314173
H 9.35079417238571 2.16306725793411 -5.71208412775304
H 8.61697640547001 3.21308954428244 -4.45775311354514

SR_0180_TS
Frequencies, energies and thermodynamic properties:
Lowest Vibrational Mode (1/cm) = -363.92
2nd Lowest Vibrational Mode (1/cm) = -10.19
3rd Lowest Vibrational Mode (1/cm) = 7.02
4th Lowest Vibrational Mode (1/cm) = 11.1
CREST Electronic Energy (a.u.) = -286.55814294
xTB Electronic Energy (a.u.) = -286.56310389
DFT//xTB Electronic Energy (a.u.) = -5585.43310691
DFT Electronic Energy (a.u.) = -5585.468757850615
DFT//DFT Electronic Energy (a.u.) = -5590.870080164937
Gibbs Free Energy (a.u.) = -5589.612348
Substrate Energy (a.u.) = -2466.969142937232
BIMP Energy (a.u.) = -3123.675372177386
DFT optimised cartesian coordinates:
O -0.56296403807490 -0.63243292030778 2.49009350322342
C -1.16136801087946 -1.38210843904848 1.69441912466856
N -2.24407896306102 -2.20689545696975 2.01743628102503
C -2.88313092449554 -2.29319243770113 3.34615163477163
C -2.78406347768301 -2.72484664191089 0.82069662324457
C -3.94994242345547 -3.45195197478964 0.59488352899583
C -4.25104686095773 -3.84111266678571 -0.71743524868705
C -3.40753363269886 -3.50970346377962 -1.78289653776268
C -2.24644100100383 -2.76625247985326 -1.56038350872594
C -1.93665119236333 -2.36317191721663 -0.26028312619052
C -0.87930220280763 -1.54426161967870 0.28196413485590
O 0.14552543335024 -1.01648302577959 -0.29174398726694

C	-0.15364263262564	0.89110564478125	-0.81377582482536
C	-1.53582333813111	0.87051409095923	-0.99549736989940
C	-2.47129016136429	0.61504740324496	-0.00219125278699
C	-3.72307092214919	0.30824300307613	-0.63831360591143
C	-3.52594179602278	0.37570464513983	-2.03616210341178
C	-4.55695794066720	0.09607291391549	-2.93532858720626
C	-5.79687443080672	-0.26015166199865	-2.40423786573460
C	-6.00359131359329	-0.34561267143881	-1.01780007404241
C	-4.97182784105818	-0.06887613398734	-0.12790401758438
H	-4.62392491445680	-3.71832602945528	1.40699480338457
H	-5.16335819584410	-4.41011145812017	-0.90333819561214
H	-3.66402333880976	-3.82605503728535	-2.79579976000709
H	-1.58705253530423	-2.49066498950845	-2.38514330858247
H	-4.39766171844128	0.14420992055173	-4.01010055306715
H	-6.61774212742312	-0.48856596984764	-3.08604797109607
H	-6.98250718719435	-0.64117664433110	-0.63608506526229
H	-5.11755475452481	-0.14977231479484	0.94870108510465
H	0.49427678166842	1.01188288812807	-1.68180246829430
H	0.22556176126630	1.25203203663741	0.14068879027301
N	-2.16901365781152	0.70484153892963	-2.27903889593629
H	-2.30197478491828	0.73080688914496	1.06465797574622
S	-1.79028210727859	1.86269453274317	-3.46350265853284
O	-0.35578292879637	1.80308632227804	-3.68189465653074
O	-2.70046698883673	1.63592063747799	-4.57478191283220
C	-3.33413239398132	-3.73833076114277	3.65160560005025
C	-2.71898998009146	-4.83738122232397	3.03990534573922
C	-4.23865286511563	-3.97932971609317	4.69373815533613
C	-3.04108430199052	-6.13984746395760	3.42187300444489
H	-1.97903929553668	-4.68130915766816	2.25438388419404
C	-4.56504784066204	-5.28066818774542	5.07385693589135
H	-4.68416363965843	-3.14112291234796	5.23227924982004
C	-3.97392064937540	-6.36929189390815	4.43262067829724
H	-2.55424871512469	-6.97989689167476	2.92359622206826
H	-5.27914527867005	-5.44049094280228	5.88340628835300
H	-4.22817214259198	-7.38841291549957	4.72826112714642
C	-4.00834079725884	-1.23914224908296	3.36117010421103
C	-3.63242069688009	0.11470664928554	3.39092242365664
C	-5.36802036146870	-1.54541184515678	3.25433303137677
C	-4.58704608503949	1.12806634530556	3.35861889726781
H	-2.57079522420056	0.36683844921063	3.44879412673674
C	-6.33006808931397	-0.52924158753660	3.22998807054048
H	-5.69916848876696	-2.58209137401808	3.19540675630613
C	-5.94613662722794	0.80787324547533	3.29196265816195
H	-4.26640438615222	2.17088866677185	3.38625203983893
H	-7.38613725013491	-0.79415913478538	3.15570859441929
H	-6.69788736391995	1.59861023682826	3.27438082977275
C	-1.84816734735687	-2.07180427931709	4.47651419120326
C	-0.61249401399197	-2.72946930418522	4.40801891386728
C	-2.16974378320667	-1.37700531186904	5.64221812456963
C	0.29641946562188	-2.65825853862454	5.45600005531211
H	-0.36534451129899	-3.30500204904690	3.51416343797294
C	-1.25880875099811	-1.30846717240484	6.70340825738148
H	-3.13638016313549	-0.88224109743373	5.74091034988349
C	-0.02212775153928	-1.93890741633077	6.61355694138158
H	1.25764089190755	-3.16940845192096	5.37247595246081
H	-1.52863840352178	-0.75522080010788	7.60468733911116
H	0.68849327370677	-1.88130647182754	7.43962272320160
C	-2.17044374426654	3.39846109965682	-2.67297997214248
C	-3.47674053738556	3.88677439284871	-2.70825013576346
C	-1.16453682859556	4.04659392034538	-1.95510168970550
C	-3.76880916537296	5.05287050647950	-2.00592368863665
H	-4.24580051159108	3.36676549521041	-3.28240765267819
C	-1.47780654136798	5.21221998528535	-1.26099257750910
H	-0.15325765785445	3.63651632707646	-1.94455959852981
C	-2.78141095097197	5.72829549186529	-1.27185845123937
H	-4.78485377376669	5.45216431264176	-2.03083691827186
H	-0.69304600984715	5.72656141131330	-0.70088443827398
C	-3.12872804169901	6.97102107827594	-0.49967140792695
H	-2.22839553647544	7.54196508922117	-0.23946356734701
H	-3.80780374374681	7.61445617001130	-1.07543148970861
H	-3.63828985961823	6.70232298852211	0.43800164905249
H	0.86396590447576	0.28162547522719	2.26257721678590
N	1.75048283313563	0.82590541498404	2.27561073658236
C	2.86012396372809	0.09710169639991	1.96171611393461
C	1.54157886035492	2.19354937342415	2.44422610718166
N	2.54067582423840	-1.16124972762990	1.58330006926849
S	4.45063517624958	0.65907164321702	2.04280944768255
C	0.19637619312337	2.60570156026137	2.34721622649868
C	2.52002143312149	3.16905269149324	2.67690833338478
C	3.42436128855140	-2.07159573755438	0.87500291732013
H	1.54310233665601	-1.33524521605552	1.44380703847556
C	-0.14440936342220	3.94714425875396	2.41864252152183
H	-0.57816028786402	1.84748660843349	2.20564426353419
C	2.14446019679517	4.51309974005695	2.75178132548178
H	3.56304813497129	2.88246970532664	2.78927493920162
C	3.10147977299453	-2.02697490020430	-0.63459602080602
H	4.44144072050049	-1.67933810329303	1.00876052751083
C	3.37047275557264	-3.49336377046508	1.48241029960241
C	-1.59807814854787	4.32761012640322	2.32670184682716
C	0.82702261512218	4.93083540950612	2.61212506349300
C	3.24044700435324	5.52627739333619	2.94485531814117
N	2.84551212852339	-0.65081393754404	-1.04728208553471
H	2.22607930403340	-2.65619507096148	-0.86570143063293
H	3.95862430764977	-2.42196736681144	-1.19771116773836
C	3.95965422684017	-3.42411522180863	2.89544304696080

C 1.92895115440729 -4.01134279394092 1.56254759669677
C 4.21612546885700 -4.44175814195881 0.62542002634169
F -2.21711907561209 4.19136794176604 3.50583836692685
F -1.75563694510762 5.59934895420137 1.94909247502126
F -2.26084114361030 3.56416002674064 1.45520462338204
H 0.56021427564321 5.98599863206342 2.65915625239658
F 4.02211745718875 5.21812869361442 3.98155884286274
F 4.03758587194738 5.58708665761921 1.86879805310736
F 2.76534476240246 6.75387839975986 3.15094394491024
H 1.95713553915433 -0.30873875213221 -0.67322920561327
P 3.26503532097052 -0.01070647154418 -2.50992921566298
H 3.39956882215392 -2.70896164801542 3.51627325632147
H 5.01162328749704 -3.10123457878111 2.86511532324753
H 3.91415687949681 -4.41196375034903 3.37823237471436
H 1.92051184160193 -5.02825800088144 1.98290097856970
H 1.44386878813923 -4.05315300181326 0.57522622297805
H 1.31962722933505 -3.37480045346090 2.22226119053401
H 5.23131799146953 -4.04007806695495 0.47852932188375
H 3.76393758042444 -4.61374789871367 -0.36261142786500
H 4.30440514334302 -5.41750265808675 1.12570466231424
C 2.23401681317553 -0.58715119954326 -3.86110555003535
C 2.97537119466031 1.75563290886146 -2.31707616241785
C 4.98776461269531 -0.40257924058391 -2.80150116641913
C 2.58175342286802 -0.38867356621360 -5.21079162072207
C 0.94990909404031 -1.04215473819734 -3.53898579740468
C 2.55868763645886 2.52376757921803 -3.40820777657235
C 3.09697425722318 2.36349888551056 -1.05252993459985
C 5.96888240402937 0.30801491292468 -2.08515959490298
C 5.37153808920770 -1.45496997154686 -3.63817793745491
C 1.64925230857171 -0.61366523603249 -6.20849571598263
H 3.57908748421568 -0.03285739891315 -5.48193926340638
C 0.00552638940521 -1.25891283916247 -4.53846875258226
H 0.66027754787506 -1.19931683027499 -2.49401261108112
C 2.25904704229584 3.87531936939192 -3.25848163503108
H 2.42196645783028 2.06843870705672 -4.38983898151780
C 2.78241781762296 3.70270138165693 -0.89269703963432
H 3.43207480075420 1.78662500420842 -0.18592067230803
C 7.30407464841792 -0.02462550850008 -2.22007779085110
H 5.68467026265267 1.12451806541863 -1.41659363041044
C 6.71563387548257 -1.79457175400298 -3.78182399116555
H 4.62152427313552 -2.02938797407221 -4.18611699651392
C 0.34282504939474 -1.02118311116665 -5.87683033171044
H 1.89356950821950 -0.45247777717877 -7.25891971972247
H -1.00077891535780 -1.57221818414004 -4.26440164189055
C 2.34695583106360 4.46625516899745 -1.99092326857947
H 1.91762684155785 4.44175314535946 -4.12315507499867
H 2.86633314161389 4.18955712156662 0.07938641403059
C 7.68990241166732 -1.07729547845502 -3.07227688033236
H 8.07983333920548 0.51498127345360 -1.67613402252597
H 6.99016569296175 -2.61611474760190 -4.44128113178346
O -0.51901602132072 -1.14911984463791 -6.89411765423709
O 2.01255720057720 5.74009903524081 -1.73477974143423
O 9.00288591037761 -1.32633235788290 -3.14486140291387
C -1.88534188291174 -1.36724998757516 -6.58895386584186
C 1.59379535623553 6.56049079911289 -2.80939188790962
C 9.45319294865776 -2.37759326531906 -3.97758124074755
H -2.26305769470422 -0.56729300445635 -5.93250043016197
H -2.03333788888137 -2.34477207997047 -6.10496459068355
H -2.42196917228645 -1.34932664106852 -7.54290368023607
H 0.67826668237755 6.16337911012175 -3.27545323169631
H 2.38496474110918 6.64545153849621 -3.56994758461226
H 1.38692864121114 7.54739410213874 -2.38301846908445
H 9.03512878545519 -3.34311695722898 -3.65413210807547
H 9.17956265160909 -2.19361581282667 -5.02770921401445
H 10.54340981547913 -2.40164925104881 -3.88293216836801

SS_0000_TS

Frequencies, energies and thermodynamic properties:

Lowest Vibrational Mode (1/cm) = -326.3
2nd Lowest Vibrational Mode (1/cm) = -21.93
3rd Lowest Vibrational Mode (1/cm) = 7.23
4th Lowest Vibrational Mode (1/cm) = 11.39
CREST Electronic Energy (a.u.) = -286.56688714
xTB Electronic Energy (a.u.) = -286.56797485
DFT//xTB Electronic Energy (a.u.) = -5585.42851304
DFT Electronic Energy (a.u.) = -5585.476911237387
DFT//DFT Electronic Energy (a.u.) = -5590.871328948195
Gibbs Free Energy (a.u.) = -5589.613369
Substrate Energy (a.u.) = -2466.952483983898
BIMP Energy (a.u.) = -3123.68635455105

DFT optimised cartesian coordinates:

O 2.33485345892373 -0.58298293285838 -0.06425192580650
C 2.79852272473204 -1.11132602311954 -1.07838760852951
N 4.17850583567562 -1.19184672716448 -1.39887478494138
C 5.13989874152913 -0.12406179452271 -1.01396265117629
C 4.29586868882899 -1.82949851303287 -2.66092710345083
C 5.42053555348213 -2.09738896062144 -3.44020023845491
C 5.25107377806159 -2.76355872308156 -4.66298328747362
C 3.98643065716029 -3.15195035441791 -5.11291509726051
C 2.85294237679163 -2.85734691084077 -4.35231098621437
C 3.01122018931485 -2.19083465079837 -3.13641572284731
C 2.04399945566531 -1.68999552041900 -2.18403354347187
O 0.77565634950529 -1.63812457440567 -2.29188501345639
C -0.15196686669523 -3.26755611267546 -1.41721703412044
C 0.78442386414225 -3.46830149247385 -0.41328165146328

C	2.08097865559932	-3.93370385391376	-0.63692085184706
C	2.82581344751220	-3.82426884555320	0.58121746201422
C	1.99948828028013	-3.18890319971858	1.54139622656389
C	2.43724796432154	-2.98185446303444	2.85129169658858
C	3.72645085723896	-3.40392548044708	3.18098109911126
C	4.56255606762747	-4.02259313617517	2.23879833401028
C	4.11832366963671	-4.23907808415200	0.93870277803560
H	6.41998108021166	-1.79996070119150	-3.13259646679398
H	6.13221879514649	-2.97682151143018	-5.27033611629754
H	3.88519735111275	-3.67295227020390	-6.06661287933408
H	1.85238715234147	-3.12486117307634	-4.69934032459227
H	1.80441102973122	-2.50406741094394	3.59483655084574
H	4.08559410277750	-3.24840608881451	4.19981949305556
H	5.56482922979322	-4.33990467135676	2.53163958804121
H	4.75095735331301	-4.74041381232539	0.20343657428855
H	-0.03423589771341	-3.87328286637820	-2.31394452351483
H	-1.12356705066814	-2.81566449443541	-1.24340107118049
H	2.39662899976254	-4.44454069379391	-1.54361362055942
N	0.74803503695255	-2.96121301270088	0.92802235415375
S	-0.38402545771424	-1.85349155206192	1.45865997723040
O	0.17397558910438	-1.21542264852827	2.64239134148398
O	-0.77999768439893	-1.02624341159278	0.32398525306804
C	4.89043692816440	0.43618016704056	0.40369371434959
C	4.69098950991181	-0.44858406266212	1.47264419544317
C	5.06865773059606	1.79347473993422	0.68124436887883
C	4.67301484184485	0.01517663948289	2.78246596169216
H	4.53547367547939	-1.51082461892862	1.27069349544330
C	5.05865091789733	2.25899165628854	2.00146613079954
H	5.23107396659435	2.50809422418774	-0.12653126794077
C	4.86627712299941	1.37392334628027	3.05627012845409
H	4.50270271564261	-0.69021966097631	3.59772004961261
H	5.19587497246684	3.32513405635083	2.19175380358503
H	4.85516341855442	1.73468131381142	4.08614788917248
C	6.56131095528641	-0.69754991659356	-0.86255754161418
C	6.76365894199409	-2.05659087528908	-0.60295291712954
C	7.66523856106116	0.16325187532810	-0.81602097984675
C	8.04183512686252	-2.55058926757419	-0.33964737897751
H	5.91021568616548	-2.73466755473666	-0.61158763060026
C	8.94448259043997	-0.32911246866191	-0.56049392443361
H	7.52448820976531	1.23574047189541	-0.96567440291855
C	9.13995177736958	-1.69102408093892	-0.32510163448332
H	8.17635700224432	-3.61631273832758	-0.14525588528394
H	9.79103999657127	0.35922358575182	-0.53662824781991
H	10.14043449797274	-2.07680342486269	-0.12265239446013
C	4.98231546151286	0.95928632901645	-2.09975590610882
C	3.77155789258186	1.67212651122597	-2.12370614179819
C	5.89517435817300	1.17225532783707	-3.13513012532698
C	3.49183273899585	2.58301627237651	-3.13800085328777
H	3.04191662461952	1.50827443364601	-1.32819293027258
C	5.61808141001870	2.08970952866910	-4.15485482466268
H	6.83296771180938	0.61780747460082	-3.16687231976774
C	4.42089497754096	2.79976477881672	-4.16020385511797
H	2.54271838710961	3.12420331457209	-3.13132842434709
H	6.34779025221162	2.23942602296465	-4.95245528290781
H	4.20697600018507	3.51418696055635	-4.95687425444396
C	-1.78200596157853	-2.81453181569606	1.96127472791352
C	-1.81999437678377	-3.28229791347988	3.27662220942976
C	-2.81212205296171	-3.08014423588218	1.06241846874898
C	-2.91797346234015	-4.02799523043130	3.68870641297118
H	-1.00652783811125	-3.05270783970377	3.96740769237532
C	-3.89758705709088	-3.84060283633008	1.49279182621553
H	-2.79046067705531	-2.67083493526423	0.05137792938302
C	-3.97142990328772	-4.31693956285862	2.80670925980231
H	-2.96371761226124	-4.39049434325828	4.71774208655891
H	-4.71409472900847	-4.04971136996524	0.79780484670112
C	-5.17293654599509	-5.08608233894065	3.28229182379683
H	-4.88130430638061	-5.88706778224290	3.97470352805869
H	-5.85889989561649	-4.41560312840126	3.82381844462761
H	-5.72465052342066	-5.52381344544179	2.44064944948324
H	-0.16364816236185	-0.09835345482541	-2.22563020320380
N	-0.72805191643770	0.76685150541943	-2.28476308395587
C	-0.43838289286238	1.75318099815591	-1.42203204877017
C	-1.86491565005651	0.75615516948799	-3.18677279381455
N	0.57319970750267	1.44724957832369	-0.56859434602459
S	-1.26930281494489	3.23348211673575	-1.35412678827588
C	-2.74007725781211	-0.44967417076014	-2.81361426709499
H	-2.42822147190593	1.67575993084647	-2.97384540814741
C	-1.45365158169256	0.80324991229520	-4.68216012664959
H	0.86415933480333	0.46029444375671	-0.48328155610485
C	1.09585380105406	2.27286257883180	0.43902417318411
N	-3.08301917777979	-0.46016032537740	-1.39442788153812
H	-2.19658645898038	-1.38401016721882	-3.02412702776606
H	-3.65955268021943	-0.47245121763574	-3.41674557553656
C	-2.69331015361414	1.14587757659010	-5.51755516548244
C	-0.41117239815268	1.91278313771702	-4.86423104753901
C	-0.85720767235532	-0.52985857497533	-5.15040357055546
C	1.60546253668531	3.54721901367357	0.16271126358910
C	1.16275846123200	1.77244312357374	1.74049152671924
H	-2.27275451814950	-0.52374297216941	-0.76973437888070
P	-4.41822864120088	0.23072043011036	-0.70054991452409
H	-2.42560927821666	1.20045035530493	-6.58351368233022
H	-3.10856480979030	2.12170389884663	-5.21904953501977
H	-3.48418173927943	0.38755928085043	-5.41274282707183
H	-0.21266981755385	2.06961487662404	-5.93521212052408
H	0.53670966279385	1.64488579592753	-4.37465062215603

H -0.76423205573065 2.85967963930541 -4.42741193319109
H -1.62249241187364 -1.31856995439675 -5.21466008018499
H -0.06698628754105 -0.87428160216592 -4.46473519936656
H -0.41847075080843 -0.41104855555108 -6.15278600391998
C 2.14686898905676 4.30790612215424 1.19318562885099
H 1.57574110455776 3.93234214660002 -0.85609530938944
C 1.69747313531864 2.55891582876517 2.75986459477327
H 0.79240620451709 0.76836214927526 1.95230473823962
C -4.05929635931888 0.15922651387954 1.05183588431430
C -4.76403092891310 1.91294245546580 -1.22666956957123
C -5.84343554551227 -0.77139898965105 -1.12345768841089
C 2.79187866207042 5.62803756145875 0.87696111298576
C 2.18590830596635 3.83515127147407 2.50596376642652
C 1.67690028591516 2.02212283980553 4.16392199090209
C -2.89280022859764 0.77217439365908 1.53260841115231
C -4.84278948467375 -0.62112903029224 1.91699856325942
C -5.06215822294618 2.15654072100176 -2.58122863100020
C -4.68927234234804 2.98887030788271 -0.33942269873742
C -5.67152844126896 -2.11583898780854 -1.46742895091301
C -7.14165191325168 -0.23621139272624 -1.04513185838204
F 4.12018818636381 5.49941479802167 0.74485299280479
F 2.33801743077812 6.15144858487197 -0.26204257071847
F 2.59056968104409 6.52820336423342 1.84242236228295
H 2.61143501826936 4.44162944691501 3.30662817194943
F 2.55252574817887 2.64269314708871 4.95726515867325
F 0.46655806994492 2.18460604736171 4.72679668133372
F 1.94071945476952 0.71612547497830 4.21219183463967
C -2.48619451522979 0.58155626987713 2.84880172062612
H -2.27770760329852 1.39550565003529 0.87321378591072
C -4.44402962209815 -0.81393399383610 3.22866285862177
H -5.75224817448135 -1.10578241479309 1.55439529297578
C -5.23855796255894 3.45049483529502 -3.03488091995682
H -5.15288635182001 1.33034317899872 -3.29193296452576
C -4.88691650227959 4.29520890313570 -0.78274274026422
H -4.47100303949332 2.82079977225050 0.71667048598271
C -6.77130866281382 -2.92884957455017 -1.73345555600649
H -4.66515364772716 -2.53491328013818 -1.54020604643806
C -8.23986465405343 -1.03787573949202 -1.30156816947516
H -7.29063017627488 0.81408239730144 -0.78342597476521
C -3.24712945677210 -0.24288864011483 3.69489499152530
H -1.56621503352420 1.04911875984666 3.19769139758456
H -5.02416906413036 -1.43664250490562 3.91078215047249
C -5.14624476918499 4.53326621581763 -2.13863584457441
H -5.45775590405522 3.65921693395896 -4.08259258020423
H -4.82313770318010 5.11505492393159 -0.06940302979490
C -8.06471785910550 -2.39109732880814 -1.64873250718425
H -6.61171112176223 -3.97001150430617 -2.00928270966109
H -9.25487919225169 -0.64319229425931 -1.24759693619987
O -2.89785262057300 -0.54143284255909 4.95165253661252
O -5.32965097068019 5.75110417674946 -2.66433004404998
O -9.18195188629197 -3.08879218543878 -1.88642713654399
C -1.66550337015990 -0.04541861515758 5.44654903931888
C -5.23485484108118 6.87879000096690 -1.81565328408002
C -9.07261502169022 -4.45326583510986 -2.24443509918912
H -1.54535166906311 -0.46572665537205 6.45034824630133
H -1.67967510492626 1.05286249128777 5.50729129101649
H -0.83090967602865 -0.36432366653828 4.80348450810869
H -4.23613206926633 6.94370065362750 -1.35746172139781
H -5.99850824909724 6.84203909041810 -1.02367589520216
H -5.40550740873231 7.75662173784412 -2.44694336608393
H -8.50540714536750 -4.57117682663320 -3.18031573442947
H -8.58486364733396 -5.03255680822047 -1.44562628294112
H -10.09457714612139 -4.81791190375027 -2.38828482906920

SS_0001_TS
Frequencies, energies and thermodynamic properties:
Lowest Vibrational Mode (1/cm) = -344.42
2nd Lowest Vibrational Mode (1/cm) = -21.38
3rd Lowest Vibrational Mode (1/cm) = 5.34
4th Lowest Vibrational Mode (1/cm) = 12.78
CREST Electronic Energy (a.u.) = -286.56684389
xTB Electronic Energy (a.u.) = -286.56790454
DFT//xTB Electronic Energy (a.u.) = -5585.42832521
DFT Electronic Energy (a.u.) = -5585.477155782126
DFT//DFT Electronic Energy (a.u.) = -5590.872386902685
Gibbs Free Energy (a.u.) = -5589.61469
Substrate Energy (a.u.) = -2466.954615571391
BIMP Energy (a.u.) = -3123.686931126141
DFT optimised cartesian coordinates:
O 2.13420327656602 -0.76081867278836 0.86789338474876
C 2.90144096059201 -1.13725590897480 -0.02204075230260
N 4.25670119468773 -1.50457607999060 0.16175893440172
C 5.10723210821231 -0.91944568181349 1.22939542728435
C 4.80141545000295 -1.81230102737523 -1.10973465823555
C 6.09939818005159 -2.16451984648132 -1.47482742598713
C 6.36943003371174 -2.43819386196478 -2.82382522365585
C 5.37054816177576 -2.35332573409342 -3.79778459095160
C 4.07549248544231 -1.96934907695757 -3.44262761370456
C 3.79637258164204 -1.69354193838592 -2.10296635507083
C 2.60180112914811 -1.21268640073903 -1.44558570813942
O 1.50106394026609 -0.81886270716999 -1.95776975065926
C 0.07366451648714 -2.28641973317883 -1.99829734149317
C 0.51132159204059 -3.01469014169328 -0.89556495461158
C 1.70629375195916 -3.73165305935318 -0.87198755922395
C 1.94261065747545 -4.19621164377179 0.46219943231353

C	0.91402998994530	-3.67995002566667	1.28837632720341
C	0.83904081288165	-4.00483613206093	2.64478536978683
C	1.83210844471931	-4.83233652466817	3.17085432773960
C	2.86844379190278	-5.33808254432562	2.37052584812880
C	2.92620594007126	-5.03077807915551	1.01524849643347
H	6.90412807367007	-2.22722581425174	-0.74591750065063
H	7.38432435714552	-2.71884980759658	-3.11009250708108
H	5.60830247198226	-2.57480364579748	-4.83978114276465
H	3.28864509330187	-1.86899781885031	-4.19363718564113
H	0.04379350246508	-3.62199758138571	3.27978213368541
H	1.79438386405813	-5.09093991895593	4.23048988729731
H	3.62794348157914	-5.98364340154361	2.81488335821798
H	3.71246533974545	-5.44115362479993	0.37822296076223
H	0.40943553476766	-2.64544233265634	-2.96945634386418
H	-0.81266004634914	-1.65897777358421	-1.97709464775904
H	2.26614823067910	-4.00933818458654	-1.76182699132927
N	0.04934783445942	-2.93705586652400	0.45978564441888
S	-1.09021782530253	-1.85220180692579	1.01565987573287
O	-0.85195281584563	-1.66818426224441	2.43896061569339
O	-1.05681018096591	-0.69005441150986	0.13389888957679
C	4.32820348445370	-0.67617167259971	2.54193250545169
C	3.54551321024552	-1.71082618989697	3.07511775155055
C	4.53026576911484	0.47422151704501	3.30585208144153
C	2.96749293393230	-1.58713454603786	4.33165958252599
H	3.37849459753519	-2.61580974891164	2.48554774893697
C	3.96066257570304	0.59241357204743	4.58007415265469
H	5.14065009954049	1.29315140500310	2.92426783944641
C	3.17881975156920	-0.43375594571779	5.09735975471396
H	2.34624751342954	-2.39772970344510	4.71763824955798
H	4.13155732097780	1.50283011620996	5.15809549654396
H	2.72803404073390	-0.34004172152034	6.08671467244188
C	6.18322502988809	-1.92028946022837	1.69183441218852
C	6.04900078140399	-3.29001257452068	1.45101396266417
C	7.23571654003681	-1.48589927510219	2.50912198790979
C	6.96060020120538	-4.20289758627276	1.98458761789373
H	5.22046949373363	-3.64544223095895	0.83960582818708
C	8.15234207958727	-2.39385745504786	3.03601372966148
H	7.33211386928376	-0.42546307211810	2.75194085035600
C	8.02103263230973	-3.75907503026738	2.77306555168558
H	6.83579327810150	-5.26803865818880	1.77974662224471
H	8.96802445397052	-2.03202245693243	3.66435952029042
H	8.73612729593599	-4.47107891971854	3.18842485520834
C	5.65621071487312	0.39198805863321	0.63200524047016
C	4.72835640099697	1.41251949754930	0.36052469997495
C	6.98116144985350	0.57871799692735	0.23280807800937
C	5.12184700676274	2.59273329526272	-0.26282896369822
H	3.68387316733899	1.27563570038788	0.65246077914183
C	7.37870970930587	1.76434834419158	-0.39527117045788
H	7.72272043518561	-0.20286499532113	0.39699918072650
C	6.45552337729922	2.77625836895422	-0.64016931590650
H	4.38442986218809	3.37385656621720	-0.45785746299875
H	8.42016452123601	1.88661849536309	-0.69768544384408
H	6.76708373193902	3.70102286629898	-1.12854473204119
C	-2.64646910670293	-2.66644465586525	0.81445114409887
C	-3.32791556545564	-2.57006409371470	-0.39737055651270
C	-3.15391394893520	-3.40373395966812	1.88538009904200
C	-4.54635959397689	-3.23148966126914	-0.53266852365777
H	-2.92935720746040	-1.96668976943365	-1.21498772151068
C	-4.37675563605755	-4.04733891233027	1.73169602787300
H	-2.60574175491229	-3.45645677431890	2.82781293971146
C	-5.09121555179174	-3.96841516584035	0.52664702527492
H	-5.09377640814778	-3.15974327526496	-1.47523378835389
H	-4.79052155470680	-4.61755442916289	2.56595507030472
C	-6.43633985445982	-4.62778239481728	0.39503636247032
H	-6.46777047986138	-5.57742906047724	0.94547775079667
H	-7.21731667424135	-3.97540877654900	0.81711028544890
H	-6.68778831687712	-4.81498035268854	-0.65670712774159
H	0.46774713385853	0.67393185540746	-1.88627319402987
N	-0.04891567716401	1.56427952991656	-1.80636888629868
C	0.08469485654052	2.22927035892167	-0.64770029093396
C	-0.87230355393784	1.98689594974576	-2.92404165506190
N	0.84547451600577	1.58193171563251	0.27124680865484
S	-0.63595407139126	3.73357251859184	-0.31970313197852
C	-1.95585652383966	0.92311347157470	-3.15177601640513
H	-1.36391236970266	2.91543697890113	-2.60077089962611
C	-0.03557872883567	2.31918105081815	-4.18641432631041
H	1.02243113366818	0.57248524659096	0.14967723598952
C	1.07179769369759	2.03613629277151	1.58680070661133
N	-2.68735327185191	0.61505018978968	-1.92649588121535
H	-1.49696723361249	-0.01189089188435	-3.50924375986162
H	-2.66190706454978	1.24279492311121	-3.93165130092059
C	-0.94567561282854	2.99579836965561	-5.21872448812182
C	1.06956382501174	3.30318040861061	-3.78378449940439
C	0.60095998862047	1.06180004854795	-4.79226978395486
C	1.87451579263439	3.15369655701510	1.83535943423990
C	0.52799932719578	1.32454329688527	2.65599373778907
H	-2.09772330754306	0.28048382158359	-1.15723294266077
P	-4.10523862010110	1.31391306258847	-1.43367557938063
H	-1.74450597243099	2.32565775833907	-5.57035756539001
H	-0.35633387605724	3.29605341927714	-6.09813655605218
H	-1.41190675588474	3.90013561632351	-4.79575722065287
H	1.60310353656461	3.65677082894301	-4.67882020326155
H	1.80032322401277	2.82437410216461	-3.11549993298857
H	0.64710678383240	4.17342355154002	-3.25814048910660
H	-0.15293807115206	0.40088008927754	-5.24647822900491

H 1.15209732407730 0.48571510913729 -4.03206718547251
H 1.30975356849260 1.34787598793765 -5.58419057585960
C 2.11873862177503 3.54752617717983 3.14677138179700
H 2.30576166915541 3.70282438475601 0.99783681792303
C 0.75941062957534 1.75449855730254 3.96266081448777
H -0.07330755800635 0.43530117534253 2.46177532569577
C -4.29902235716557 0.73731757014287 0.24952078752627
C -4.13270577530896 3.10950850887439 -1.50785545791858
C -5.42072548214787 0.70711314614786 -2.48913061086399
C 3.08149089098767 4.66884740499112 3.42209801501392
C 1.55128898471978 2.86642864703844 4.22458943087632
C 0.07880147306959 1.03269347366815 5.09221864203507
C -3.30285680708876 1.03619374551818 1.18979666101335
C -5.35413484569071 -0.12001386151901 0.60162745651544
C -3.97360485877299 3.74380879349361 -2.75491988685878
C -4.25463048211491 3.89053296086925 -0.35628868795224
C -5.27576801163952 -0.51532460393083 -3.15212127166519
C -6.63297412442465 1.41276744583304 -2.59146465777609
F 4.30935816706101 4.19917438634356 3.68625569282390
F 3.19933754992368 5.50048363107699 2.38718335814530
F 2.71321036458088 5.39251339587072 4.48200288245428
H 1.74057000469933 3.18827952810086 5.24946148507314
F 0.65541512900701 1.27630818436089 6.27030431128524
F -1.20582377805224 1.42012687426822 5.20538523625091
F 0.05612259737839 -0.28798351872982 4.91601415430799
C -3.33180307102348 0.46048709492160 2.45566447862691
H -2.47919707371854 1.71262164104187 0.93309096734197
C -5.38854199161693 -0.69590891956253 1.85993600097391
H -6.13656796317602 -0.36073782997826 -0.12177507294591
C -3.89548669870834 5.12195578822491 -2.83303857639365
H -3.90618011394299 3.15870908790663 -3.67591040754244
C -4.19795528014416 5.28131203882130 -0.42680119307707
H -4.39433753011043 3.42074736902563 0.61872951925337
C -6.31914578846259 -1.04042825930518 -3.91169705050592
H -4.33302867769062 -1.06320099270187 -3.08398028605764
C -7.67619764418673 0.89610952229853 -3.33924893503705
H -6.75766554289370 2.37148061091760 -2.08245760511274
C -4.36041558564190 -0.43789390370949 2.78340626009595
H -2.54341631738804 0.69551143522118 3.16969319196275
H -6.18487664894731 -1.38422270106648 2.14569560103479
C -4.00098883204190 5.90385951493936 -1.66636959281378
H -3.76033590744834 5.62874593259155 -3.78918542732458
H -4.29651958059380 5.86490983111690 0.48688462057391
C -7.52887800226655 -0.33530276101103 -4.00556570303853
H -6.18070501953701 -1.99009287728061 -4.42597929020968
H -8.62418428892211 1.42673015794164 -3.43264372462734
O -4.42602733530165 -1.09895467644339 3.94506313280470
O -3.91353874105841 7.22953859839322 -1.83446702351110
O -8.58698467349694 -0.75657310666123 -4.70870530779224
C -3.35172017977413 -0.96198712551974 4.86055528552537
C -4.00638194253163 8.06638127661207 -0.69792448299305
C -8.50283057329121 -1.99066574780164 -5.39563731423506
H -3.55988839088403 -1.65254777884297 5.68432133570337
H -3.29132163009888 0.06540651141584 5.24875197386189
H -2.39672550485557 -1.22603575166288 4.38028968951327
H -3.20232759824549 7.84539019009811 0.02058023243781
H -4.98198296236255 7.95083413140756 -0.20139188373551
H -3.90060045540416 9.09379304092855 -1.06052820767656
H -7.70359897847192 -1.96748088208040 -6.15204013393050
H -8.31957226906942 -2.81976395900479 -4.69519401061266
H -9.46884425259907 -2.13621854996935 -5.88929362053387

SS_0016_TS
Frequencies, energies and thermodynamic properties:
Lowest Vibrational Mode (1/cm) = -350.71
2nd Lowest Vibrational Mode (1/cm) = 6.02
3rd Lowest Vibrational Mode (1/cm) = 10.3
4th Lowest Vibrational Mode (1/cm) = 17.06
CREST Electronic Energy (a.u.) = -286.56409282
xTB Electronic Energy (a.u.) = -286.56455822
DFT//xTB Electronic Energy (a.u.) = -5585.42669022
DFT Electronic Energy (a.u.) = -5585.478209283921
DFT//DFT Electronic Energy (a.u.) = -5590.873516256622
Gibbs Free Energy (a.u.) = -5589.612787
Substrate Energy (a.u.) = -2466.952408945087
BIMP Energy (a.u.) = -3123.679423294298
DFT optimised cartesian coordinates:
O 1.75700233005543 -0.68116216335393 -0.08178026146234
C 2.19316640069274 -1.07513866277572 -1.17227993364992
N 3.56320782412508 -1.16298294491932 -1.51419189197052
C 4.55966110161148 -0.20481341003926 -0.95624273491230
C 3.64333371434956 -1.58766910339617 -2.86826106341724
C 4.74546584770786 -1.78725323277135 -3.70001134263777
C 4.53134935974866 -2.23689580551291 -5.01125195624471
C 3.24523227699819 -2.48399889509756 -5.49804145240075
C 2.13581675948625 -2.26284270068600 -4.68001423334729
C 2.33820129862621 -1.80880991080019 -3.37592423170745
C 1.40177642392400 -1.44377448338084 -2.33586879023087
O 0.12999510068619 -1.34969282891475 -2.39230638325412
C -0.77414067828502 -3.13567508515293 -1.87758612978816
C 0.18376012441382 -3.53902275675410 -0.95636390731004
C 1.47100325526538 -3.95733890133576 -1.28647919811101
C 2.23059204942358 -4.11860296775896 -0.08204848018709
C 1.42203715881248 -3.69461491361794 1.00038181646173
C 1.86391278371325 -3.77428668103698 2.32127988622865

C	3.14698837599442	-4.27868002489072	2.54190820782244
C	3.96766823641640	-4.69468499501153	1.48182794170653
C	3.51711775915934	-4.62036206851977	0.16756546990171
H	5.76353348460306	-1.59990733869545	-3.36928872273151
H	5.39600926405117	-2.39338934463239	-5.65822998443697
H	3.10953744033780	-2.83836804279495	-6.52138525612306
H	1.11978742270101	-2.42619183105400	-5.04622555733691
H	1.23858570420406	-3.45246594410988	3.15111822052455
H	3.51452195223225	-4.35111433621523	3.56696050087023
H	4.96459605651555	-5.08482074266777	1.69343494153780
H	4.13907637411223	-4.96200798349606	-0.66220604068287
H	-0.66585675102613	-3.53720178429407	-2.88360459476099
H	-1.75271208451500	-2.77086874184944	-1.57968312743528
H	1.77128365412204	-4.26667224073183	-2.28462818350974
N	0.17546646602348	-3.31451905226691	0.46165690489386
S	-0.98344798385390	-2.45181413260507	1.27823731542615
O	-0.39928436467874	-1.91041469824054	2.49555701123866
O	-1.58780315715327	-1.50305823821716	0.34844322851213
C	4.40352930716527	0.01211523730352	0.56644100317985
C	4.11492279325369	-1.06607521897207	1.41187857446515
C	4.72871711325968	1.24396484629808	1.14312708488398
C	4.12555079475524	-0.90534414975360	2.79395628368701
H	3.85557369708861	-2.03494417200353	0.98108247737744
C	4.72793442239663	1.40968570073324	2.53176900629576
H	4.98722609805886	2.09472126784339	0.51175454230670
C	4.42510538045368	0.33575838643004	3.36331477096810
H	3.87992388901639	-1.75492921541824	3.43227404524203
H	4.95792522320574	2.38965235638249	2.95526410621384
H	4.41290439369020	0.46151568067377	4.44801173337911
C	5.97883705135735	-0.79339227394150	-1.03321178104930
C	6.17196420787945	-2.17608854441202	-0.93268213843470
C	7.10082194703326	0.04171384575856	-1.04115250686203
C	7.45650434980669	-2.71441494674036	-0.88559450557340
H	5.30276805302501	-2.83443047196065	-0.90260478877714
C	8.38839398574009	-0.49600008257643	-0.99752790939698
H	6.97366973307578	1.12534199688993	-1.07286853093295
C	8.57258297349425	-1.87655920395935	-0.92616286023448
H	7.58459439327068	-3.79632040146634	-0.81719340564895
H	9.25083133151734	0.17251874567196	-1.01409771115627
H	9.57907617654114	-2.29686036800438	-0.89342739962383
C	4.34290084688467	1.09175454722990	-1.76020144464901
C	3.26216668135257	1.91353436149867	-1.40729487656750
C	5.06104122200800	1.40858572080971	-2.91817838221732
C	2.92535681968640	3.03362295201541	-2.16176284917044
H	2.68198888391398	1.66622997538034	-0.52027692117474
C	4.71983013708704	2.52714113916260	-3.68545514512265
H	5.89636787138693	0.78473707525969	-3.23612884738270
C	3.65750774594445	3.34701481049667	-3.30946679590642
H	2.08680552895692	3.66087644005991	-1.84648963416363
H	5.29587098709771	2.75495542497713	-4.58400180022087
H	3.39686664304513	4.22194132148555	-3.90792650084457
C	-2.21085950710108	-3.64391873773085	1.73136015465937
C	-3.15672328036445	-3.23015353403985	2.67379186227648
C	-2.24143886896536	-4.91986444145041	1.17669708894425
C	-4.14231317537890	-4.12540020530948	3.06859022828320
H	-3.11452205948301	-2.22481190842790	3.09858564783532
C	-3.24253961589212	-5.80118362720399	1.58723151701793
H	-1.49347781686731	-5.23059883980388	0.44535286719508
C	-4.19675803578516	-5.42465966526452	2.53991900104651
H	-4.88663837234490	-3.81290266695402	3.80446993004648
H	-3.27153374302222	-6.80828036372755	1.16577240202772
C	-5.24785827409776	-6.38830434977779	3.01713094333479
H	-5.02463414232950	-6.71805199281466	4.04331841415135
H	-6.23334275792500	-5.90216634843463	3.03046809788523
H	-5.29659122945979	-7.27774314853225	2.37583063699367
H	0.11877165423037	0.65498377933566	-1.98099673691024
N	-0.30700908581076	1.58662437446237	-2.00621843367431
C	-0.26474277561754	2.30217002500209	-0.86665342321007
C	-1.27204946332099	1.75629657410651	-3.07816506428154
N	0.37790521161932	1.64574630349664	0.13671591423007
S	-0.95585113373145	3.83865383834844	-0.70244152707054
C	-2.18235002358697	0.51342701776378	-3.10604647396628
H	-1.88263610566688	2.63035979504089	-2.81259017285581
C	-0.59148090434994	2.05782364608905	-4.43757850695661
H	0.62641721905905	0.65377079322466	-0.02875476045045
C	0.83037338956903	2.10402840073308	1.37486119812609
N	-2.57122842297655	0.04614291441619	-1.77829669292157
H	-1.65453878508816	-0.32354464145006	-3.58312235534015
H	-3.08113336794128	0.72640607832976	-3.69829496693723
C	-1.63088342962519	1.96633579237933	-5.56136190823282
C	-0.03816106290223	3.48503794630345	-4.38687645264340
C	0.55702775005421	1.07751309748530	-4.70506130992998
C	1.28327876232098	3.41138361374679	1.60573348537240
C	0.88762287698012	1.16286852280014	2.41036549303683
H	-1.86662079281591	-0.51870696260484	-1.29485862253015
P	-3.88568699680656	0.51678853412656	-0.89437468883835
H	-1.95569278852321	0.93038557892838	-5.73827434311203
H	-1.19790326031583	2.34490538259683	-6.49930104491908
H	-2.52024351608849	2.57444631908528	-5.32718011503580
H	0.52454528598946	3.71085506693219	-5.30606633556984
H	0.63615251758172	3.60655239797330	-3.52770614812330
H	-0.85279594185552	4.21908547064700	-4.28626828729420
H	0.22665848444306	0.02870877304256	-4.65275094369514
H	1.36776552109912	1.20735201975480	-3.96940924559351
H	0.97736307876848	1.25746135428461	-5.70653370621503

C 1.74017158844845 3.75877207209185 2.87213967418927
H 1.27820749754765 4.14184786756871 0.79979582930203
C 1.32452184463036 1.54773285863743 3.67533075035048
H 0.56384376613765 0.13452919041348 2.22517395693564
C -3.58786292494931 1.57288382478672 0.53987632115263
C -4.93790730828143 1.42386876598331 -2.03156565407072
C -4.70065562828318 -0.97794448188965 -0.31678196557302
C 2.25581983796344 5.14663416424303 3.13600823965828
C 1.75157371419925 2.84657503283567 3.92841232260717
C 1.22526648928992 0.55931317450338 4.80473032142721
C -2.54700004894254 1.27892382364279 1.42915004555697
C -4.43599003334924 2.65974302240814 0.82001209025129
C -6.02150695844038 0.79563403527404 -2.66459274384156
C -4.60719412917806 2.74183796898139 -2.37461098305527
C -4.53857262219090 -2.16866388313192 -1.03012074194139
C -5.53611816335804 -0.95696689536913 0.81270530223875
F 1.57552092712178 5.74378273284648 4.12168947820283
F 3.53796554399315 5.12475544476045 3.51996836700670
F 2.17684599585829 5.93399004371733 2.06487450494424
H 2.09674663095754 3.14061722123382 4.92096427117596
F -0.03270041648651 0.49861513769084 5.27300061339538
F 1.55407340952381 -0.67734432983231 4.43656167971370
F 2.00379839473651 0.89343412689153 5.83647289514161
C -2.32889523631815 2.06573429492169 2.55641443599328
H -1.88897426670400 0.42869434219914 1.24686410374689
C -4.21541731632700 3.45596441191552 1.93246793265078
H -5.27549224499106 2.89499754332311 0.16435829835178
C -6.75596201672130 1.47478046098846 -3.62371843380498
H -6.29355153629523 -0.23004461796329 -2.40557422145922
C -5.33715996699422 3.42930623583263 -3.33952428882234
H -3.76952127170757 3.24744775040745 -1.88295559932791
C -5.19590001576075 -3.33213708875319 -0.63657309857093
H -3.89420391405669 -2.18932122198416 -1.91103918490825
C -6.20079096673859 -2.10662359356693 1.20635369696102
H -5.66523536892081 -0.03712892957319 1.38798500705260
C -3.14812103121116 3.17603877930245 2.80159055486082
H -1.51690372305687 1.81054294156454 3.23438913539034
H -4.85637377515946 4.31083400230260 2.15010230156723
C -6.41714521597264 2.79353085733289 -3.97389584859271
H -7.60349583498525 1.00567079445478 -4.12419079945176
H -5.06060165950730 4.45267263754208 -3.58816762239312
C -6.03795674236859 -3.30277818357387 0.48415155923076
H -5.04812861581096 -4.24809750103081 -1.20658800942585
H -6.85420150279887 -2.11118472099262 2.07957586075195
O -2.98367288263675 4.00812325897136 3.83979658861046
O -7.17513570258011 3.37153514916710 -4.91422962290398
O -6.72093169894287 -4.36816781978492 0.93050006413909
C -1.85009135402574 3.83800135384498 4.66930417673892
C -6.87630778587948 4.69587974854188 -5.31273801710933
C -6.69413856441548 -5.55589073760980 0.16268580431240
H -1.88883941316682 4.63860316206364 5.41510462964728
H -0.91946614418613 3.92615505592054 4.08527707480677
H -1.86677052756153 2.86224510611114 5.17871246584327
H -7.60687734792354 4.95877357376947 -6.08429782367954
H -5.86022014019560 4.76177420365064 -5.73086616745477
H -6.96912598300827 5.39310432906401 -4.46615560213042
H -5.67488045698285 -5.96954918170036 0.10728312981831
H -7.34983546308943 -6.26991134395030 0.67156001220175
H -7.06791829867157 -5.37041209345937 -0.85615181392808

SS_0210_TS
Frequencies, energies and thermodynamic properties:
Lowest Vibrational Mode (1/cm) = -289.61
2nd Lowest Vibrational Mode (1/cm) = -13.83
3rd Lowest Vibrational Mode (1/cm) = 8.7
4th Lowest Vibrational Mode (1/cm) = 15.57
CREST Electronic Energy (a.u.) = -286.55762438
xTB Electronic Energy (a.u.) = -286.56182221
DFT//xTB Electronic Energy (a.u.) = -5585.42509339
DFT Electronic Energy (a.u.) = -5585.474414030299
DFT//DFT Electronic Energy (a.u.) = -5590.869268030378
Gibbs Free Energy (a.u.) = -5589.610894
Substrate Energy (a.u.) = -2466.966817244524
BIMP Energy (a.u.) = -3123.681915840053
DFT optimised cartesian coordinates:
O 2.03455931128442 -1.73028313488967 1.00219066518649
C 1.88306177411628 -1.12010558578644 2.06076477303238
N 2.87258648077984 -0.92036839816408 3.04028123297167
C 4.19737311315901 -1.56316038779497 2.93252795623913
C 2.25659677223731 -0.44313162005207 4.21882428503502
C 2.75094001738687 -0.23835863209467 5.50703292305456
C 1.88895410712739 0.27660086959248 6.48728261967745
C 0.55496166742664 0.58140453439098 6.20491826682970
C 0.04223294852856 0.34656924610251 4.92756649269297
C 0.88655408359210 -0.17541938892648 3.94583165259306
C 0.62677736921872 -0.59165553709753 2.58966465752262
O -0.47295957555209 -0.64433380657662 1.96045410800481
C -1.02226436848743 1.22611309317637 1.13496815572039
C 0.20703815643685 1.72543008023985 0.71774373747808
C 1.24423806696501 2.12557043722261 1.56500170324402
C 2.31272143086155 2.65605979367508 0.76570572856259
C 1.93876792074310 2.51794594785196 -0.59331910134923
C 2.73834865724143 2.97837523067590 -1.63915220109782
C 3.94899706077214 3.58837328521585 -1.30389389729710
C 4.34399635703711 3.73121204713803 0.03590895392752

C	3.53657096198377	3.27382855942259	1.07363220179089
H	3.77883174271936	-0.46551606585953	5.77791386162727
H	2.28021472064398	0.43645176030494	7.49332206099670
H	-0.08888716228700	0.98451038903790	6.98829642497730
H	-1.00655485183632	0.54204944771122	4.69307163045036
H	2.42575608893446	2.88557972988315	-2.67916867570227
H	4.59296736948951	3.96381110867009	-2.10045250962125
H	5.29527367508019	4.21396402922395	0.26553842444538
H	3.84202749625748	3.39647581553038	2.11466171676230
H	-1.31985070575707	1.48113878979408	2.15011763160006
H	-1.79988256976557	0.97673083165979	0.42241717660396
H	1.14045556311281	2.20648469856505	2.64464121741668
N	0.68055660539269	1.89466769633597	-0.62838989726051
S	0.04760641346300	1.27629052524499	-2.05381748035497
O	-1.11110729660906	0.47810787122329	-1.66655417757083
O	-0.13269548445670	2.36232297613928	-3.00952734745770
C	4.79484254922443	-1.36390249067037	1.52174520911153
C	4.52869501424099	-0.19164585662273	0.80358284552858
C	5.77137892911844	-2.23695903045842	1.03501475776784
C	5.22091918676404	0.10237448130058	-0.36736895574551
H	3.77020847443830	0.50417441369926	1.16548094656679
C	6.45419067877638	-1.95339909663607	-0.15174860027977
H	6.02045408806612	-3.14366874146946	1.58920820758801
C	6.18725746371521	-0.78094772736119	-0.85558116794181
H	4.98818963466657	1.02431160021277	-0.90529936380599
H	7.20770451362624	-2.65409301385818	-0.51619053123162
H	6.72596034136474	-0.55552381295433	-1.77781783835240
C	5.23691903292160	-0.81278598085863	3.78918244167343
C	5.18825329756917	0.58606631306447	3.86494698423401
C	6.32109175385363	-1.47684629028625	4.36837804588423
C	6.17259161752314	1.29792041444901	4.54419849522491
H	4.35979093573779	1.12060117659452	3.39613638884155
C	7.31144793802202	-0.76342201391544	5.04930267226632
H	6.40402340258669	-2.56161834698934	4.28901015113881
C	7.23753463147045	0.62424302759573	5.14799995319542
H	6.10744421447368	2.38587442270489	4.60262522728604
H	8.14571396350133	-1.30231877944251	5.50155414326609
H	8.00849161157505	1.18058857268686	5.68355664579089
C	3.97729317289579	-3.03324646647659	3.33172948401693
C	3.62596199799512	-3.99475323049263	2.37369190893209
C	3.95106043338617	-3.40370988974852	4.68382802817175
C	3.28838399362214	-5.29397977188827	2.75592759595766
H	3.60519821955518	-3.71994234520771	1.31952491544649
C	3.61067398021756	-4.70138380943097	5.06687052724630
H	4.20273061922694	-2.67575006514118	5.45546571529681
C	3.28295585755840	-5.65443599685202	4.10321371223155
H	3.02763325675822	-6.02520423274807	1.98987495185754
H	3.60362121478835	-4.96501711086517	6.12585129099996
H	3.02117070454426	-6.67137768933667	4.40040608817676
C	1.34189395268677	0.25051408563391	-2.67180721928493
C	2.04626054194152	-0.54876353169728	-1.77916764317157
C	1.60616282278560	0.24816394994419	-4.04127538012478
C	3.05110631947924	-1.37101936770938	-2.27487409345775
H	1.82607125694584	-0.55145406223323	-0.70887390332619
C	2.60914302155887	-0.59198532257990	-4.51651500323353
H	1.03912928715815	0.89173371589485	-4.71573477859399
C	3.34509072925220	-1.40861757105181	-3.64365002158816
H	3.60996257798171	-1.99924198114758	-1.57767857630506
H	2.82038047627881	-0.62148728766036	-5.58752352387446
C	4.42942054541517	-2.31080171049883	-4.16397508856673
H	4.13582635085885	-2.76209611258306	-5.12192055659495
H	5.35717709648263	-1.74144035725195	-4.33261710419287
H	4.65090520136131	-3.11060119315523	-3.44369380651982
H	-1.98197465962116	-1.19057048543857	1.15235560196871
N	-2.65207355196378	-1.53928952569808	0.45893760270513
C	-2.13543111316146	-2.23688873543383	-0.56709110963306
C	-4.06419037072221	-1.32237570757292	0.72646615704840
N	-0.78095603927524	-2.36335262599251	-0.51605615898980
S	-3.09079634216344	-2.92689406191767	-1.78909705099809
C	-4.40963174706438	0.14528672999861	0.44900597901464
H	-4.61528656208127	-1.93685046700807	0.00183430049197
C	-4.44375315056815	-1.80514232411801	2.14954616715697
H	-0.29140959643374	-1.96382848262186	0.29557901894246
C	0.03132423767480	-2.99369883514917	-1.47225674658596
N	-4.02304691551552	0.46664812163071	-0.92630158938088
H	-3.89175394539996	0.79685337907395	1.17576255369580
H	-5.49130104400180	0.30458321129677	0.56341181804426
C	-5.96721014387678	-1.74386303546730	2.31050386781992
C	-3.98922659837861	-3.26380389855497	2.28851979988500
C	-3.77487897707317	-0.95771301166778	3.24031452591666
C	-0.13143511152668	-2.75957710609755	-2.84537527455814
C	1.06457725145050	-3.82133907074417	-1.03539124198803
H	-3.09729883833883	0.09740896063078	-1.16719097742866
P	-4.24487981657534	1.98781753428041	-1.55729649524012
H	-6.46915613730192	-2.32229188909304	1.51943960144552
H	-6.34480694728610	-0.71147516224306	2.27931364339482
H	-6.25528700768558	-2.17222788655031	3.28190716625787
H	-4.38244741014439	-3.87703998355898	1.46251314041214
H	-4.35403370593076	-3.68307856732377	3.23780385149203
H	-2.89232552760532	-3.34148771354659	2.27929917642270
H	-2.68212093021536	-0.90799121836017	3.10848448550561
H	-3.97175233197352	-1.40461071090094	4.22657476547192
H	-4.16856685424862	0.06991346156679	3.25820732824706
C	0.69538007251151	-3.40296204831830	-3.75663229093531
H	-0.89147264760154	-2.05712061787940	-3.18227358303919

C 1.90705650241969 -4.42828216005268 -1.96910514653200
H 1.22213160646676 -3.96397811273832 0.03488639447756
C -3.58775509082259 1.90892878049208 -3.22260908038664
C -6.00883471110101 2.30379558036158 -1.52958488982890
C -3.38927128829453 3.25232712496825 -0.61025898351876
C 0.47113430525171 -3.19588071907069 -5.22919279304350
C 1.71994997171128 -4.25415531930562 -3.33457330639306
C 3.07843073573548 -5.22053614588287 -1.46329349809791
C -3.17481098079507 0.69211461225952 -3.77322643765059
C -3.45664250708711 3.09063494888089 -3.97330151650435
C -6.51472081049693 3.59741886878034 -1.37671295004819
C -6.89252096312141 1.23476460257706 -1.76808833801073
C -3.83992223287046 3.56682984318060 0.68811077490616
C -2.15125806728549 3.74103834177739 -1.03869911308400
F 1.61640401118759 -3.21921038191167 -5.91799234330508
F -0.30807464512728 -4.14928273039967 -5.75106014760961
F -0.12610159437325 -2.02703521736381 -5.48909653053427
H 2.37610556154768 -4.74331921465280 -4.05559759670240
F 3.60342304072265 -6.00936257965391 -2.39950311374948
F 4.06565182731547 -4.41239050083284 -1.03845415317105
F 2.75000915864215 -5.99072135419381 -0.42192900113080
C -2.58311638615872 0.65021184046614 -5.03339123775068
H -3.29275950192240 -0.24430140171688 -3.22247155569464
C -2.87259432637173 3.05634817912146 -5.22656009793577
H -3.79688724935060 4.04741849234283 -3.56786168330852
C -7.88622782349821 3.83604860780204 -1.44622431415332
H -5.84006099419335 4.43803244664408 -1.19922585206696
C -8.25468251761634 1.46307416960877 -1.83389847264962
H -6.50285814865696 0.22210493373393 -1.89814595523215
C -3.05096560819029 4.32799499712667 1.53290034165033
H -4.80799210543860 3.20435137114309 1.04396163255670
C -1.35513376456009 4.51288287354075 -0.19765214828426
H -1.77508078487879 3.49921485068028 -2.03243484460565
C -2.40973918653396 1.83764046563980 -5.75747411589510
H -2.25431485583284 -0.30797917592919 -5.43286608749854
H -2.74811727388920 3.96329052268167 -5.81880538716834
C -8.76389840239648 2.76630401988409 -1.67399822710026
H -8.25802245888318 4.85148543800875 -1.32075237307062
H -8.95789332615761 0.64869808340484 -2.01098061766775
C -1.78946324780725 4.78658699249041 1.10591816897809
H -3.37635185680537 4.56948750928619 2.54529391893015
H -0.38546262162281 4.85741209389981 -0.55657570101117
O -1.82117643967219 1.89994283785368 -6.95968960142927
O -10.09419230544932 2.89442209314042 -1.75376229063314
O -1.06954954030954 5.46866511220936 2.00521407488739
C -1.32957231615059 0.70551197525793 -7.53718573301865
C -10.66617885300632 4.17958595058318 -1.60494908080468
C 0.23422282853344 5.89116955632213 1.64966820256246
H -0.87978169395456 0.98720784445253 -8.49459020163737
H -2.14475646235279 -0.01342597057917 -7.71186041939906
H -0.56672905364100 0.23949824875734 -6.89481324175550
H -10.30274639714230 4.86563528085707 -2.38519701832502
H -10.43855492380290 4.59991417360454 -0.61340520334721
H -11.74823553228579 4.05011564966116 -1.70731832066396
H 0.20329293468480 6.60362028888904 0.81119337490953
H 0.65047868303438 6.38527285181470 2.53339623409423
H 0.86507847354084 5.02985527958865 1.37671147265560

SS_0388_TS
Frequencies, energies and thermodynamic properties:
Lowest Vibrational Mode (1/cm) = -368.12
2nd Lowest Vibrational Mode (1/cm) = -5.3
3rd Lowest Vibrational Mode (1/cm) = 8.16
4th Lowest Vibrational Mode (1/cm) = 10.48
CREST Electronic Energy (a.u.) = -286.55468239
xTB Electronic Energy (a.u.) = -286.56052064
DFT//xTB Electronic Energy (a.u.) = -5585.42923771
DFT Electronic Energy (a.u.) = -5585.471336714649
DFT//DFT Electronic Energy (a.u.) = -5590.870076283137
Gibbs Free Energy (a.u.) = -5589.610643
Substrate Energy (a.u.) = -2466.955940259991
BIMP Energy (a.u.) = -3123.684894005718
DFT optimised cartesian coordinates:
O 2.35928028568207 -0.42178593355589 -1.29230518173971
C 2.26786630029363 -1.47332690036853 -1.92518478985503
N 3.25362358957188 -1.99683171536075 -2.79661127585279
C 4.30026139654379 -1.12257786374250 -3.37832695197365
C 2.70686104534363 -3.12522942457733 -3.46081174646566
C 3.21151946428843 -3.93133954368034 -4.48250353599524
C 2.43780344817816 -5.00834401075181 -4.94256004724942
C 1.17350343186605 -5.27985175027198 -4.41158543948365
C 0.65081523583191 -4.46477913802351 -3.40699399874615
C 1.41254567007162 -3.39345700574031 -2.93797716761012
C 1.10751899115870 -2.35544335132924 -1.98682952119444
O -0.00618699629401 -2.06912197095840 -1.41940645524279
C -0.18740144302436 -2.81908940479266 0.43282434058827
C 1.16080439438522 -3.04754339724891 0.71817455194576
C 1.94088553275497 -3.93996848794620 -0.01000283872300
C 3.31281128848356 -3.79310749140552 0.37724729989572
C 3.37889449323766 -2.74337429001039 1.32482828709799
C 4.57298950846449 -2.40652680577594 1.96304678878745
C 5.72391279415826 -3.10025008428475 1.58420234284954
C 5.68695626633626 -4.11041730376709 0.61116268255852
C 4.48411731656013 -4.47175867782614 0.01052823921302
H 4.18142637906060 -3.75175177038425 -4.93875279104979

H	2.84212959691842	-5.64059112857179	-5.73526227672585
H	0.59065490699650	-6.11966680372233	-4.79558095173022
H	-0.34833406097411	-4.63644145125616	-2.99933362528522
H	4.61935381681720	-1.61875650286770	2.71232192542643
H	6.67287445249255	-2.84374647145120	2.05810331632271
H	6.60857395211880	-4.62549960355097	0.33495794699519
H	4.44083703328968	-5.27513756257464	-0.72762991531984
H	-0.73520889423972	-3.68069998023236	0.05578436919650
H	-0.76621997307487	-2.04146436691283	0.92603339999268
H	1.53428339448642	-4.71786826630786	-0.65021342479173
N	2.07308101790443	-2.24306706265128	1.48265125574830
S	1.71248501335212	-0.90907789584934	2.40372532140275
O	2.87714870052609	-0.04664714062631	2.43394833872503
O	0.45613139365576	-0.37483400001354	1.87781993829874
C	4.99388006783303	-0.24440531976070	-2.31326749517875
C	5.17161413676319	-0.70020843862858	-1.00289222344985
C	5.61724359836930	0.94750060441409	-2.69678234451630
C	5.90899744375182	0.04713652708250	-0.08719419272894
H	4.71630701226938	-1.64259764978332	-0.69385330139440
C	6.35813049644038	1.69708237019915	-1.78052907996330
H	5.53027607757868	1.30324658276015	-3.72474401018291
C	6.49936633982821	1.25478257560558	-0.46727537829497
H	6.02005849368272	-0.31856904037186	0.93444875198015
H	6.82662347620802	2.62888563211559	-2.10226820234543
H	7.07275222531794	1.83912543740089	0.25427813875308
C	5.49215707930952	-1.95757957900144	-3.88404192031865
C	5.89129959653751	-3.09097038471541	-3.16404425370349
C	6.28708896120963	-1.52683471472982	-4.94992669844012
C	7.02945965752511	-3.80411680835790	-3.53086911226066
H	5.29254238807249	-3.41951568532989	-2.31305413066938
C	7.42901867927516	-2.24188307025777	-5.32001735446982
H	6.02397297998076	-0.62201322927534	-5.49959114480645
C	7.80105672059298	-3.38709616293627	-4.61843984011449
H	7.31522863225597	-4.69121499944405	-2.96236863802147
H	8.03088029836840	-1.89394892844189	-6.16131065012079
H	8.69143231837374	-3.94676329138662	-4.90961527860298
C	3.58645335280228	-0.29173464887157	-4.46414882066095
C	2.94723306669199	0.91055113295394	-4.12332728679722
C	3.43276311506908	-0.77109880589864	-5.77224963758907
C	2.21406442710914	1.62904344077905	-5.06763395974985
H	3.03229661680324	1.28610614973822	-3.10394096270488
C	2.69722949204004	-0.05409845422696	-6.71901746860970
H	3.89899898630837	-1.71124864527179	-6.06824338915305
C	2.09046686996546	1.15243417766164	-6.37390150157956
H	1.73927840602132	2.57015018806591	-4.78078511493275
H	2.59969791744570	-0.44785576514514	-7.73255781108371
H	1.52201646519436	1.71740543050725	-7.11550082880162
C	1.39846031134196	-1.52784496978111	4.02717514534219
C	2.16064451540433	-1.05402520971954	5.08916553502992
C	0.35752517696226	-2.44376881083239	4.21397523185023
C	1.86275811653302	-1.50343902213900	6.37666681256010
H	2.96522420612463	-0.33937368808912	4.91157833173553
C	0.08412483594493	-2.88170540782523	5.50202064891795
H	-0.23011164805396	-2.80859633484731	3.36670522692476
C	0.82350679467307	-2.41157619758910	6.60167071438705
H	2.44675848060648	-1.13294963563116	7.22121889744987
H	-0.73007670002358	-3.58957846747781	5.66513092957238
C	0.47917980739981	-2.88196791812163	7.98740384584700
H	1.11051273724213	-2.39891236412945	8.74320276575747
H	-0.57432318240399	-2.65835685872604	8.21131898217306
H	0.60609400785034	-3.97149632897921	8.06857019082342
H	-0.20689607109646	0.86226290334998	0.43463399530258
N	-0.86876707990565	1.56395027259238	0.09632181057670
C	-2.10521648145118	1.54651705652638	0.62395862979838
C	-0.34819601199630	2.52674191677265	-0.85535174250732
N	-2.23063514135102	0.59712856925601	1.60433828564283
S	-3.33487042973182	2.60431953754639	0.15642876862011
C	0.29894643547807	1.80510594379454	-2.05476075310638
H	-1.21127272392875	3.10650049229985	-1.21015726887746
C	0.64446476072231	3.51131309065509	-0.17466680764441
H	-1.34706494676475	0.16965109596796	1.88509992556222
C	-3.32018572426103	0.11450944206490	2.32600679499444
N	-0.30955457262362	0.54498738018403	-2.44440046311481
H	1.34066993551598	1.55644363336232	-1.81908652247909
H	0.30486643155408	2.49027135871362	-2.91365063185389
C	1.30096842965652	4.39008486178250	-1.24747711165496
C	-0.13182321909347	4.40756491342710	0.79587953335313
C	1.72317358475589	2.74652272791046	0.60556120840654
C	-4.67146650490677	0.36834105887758	2.03466262677999
C	-3.01533829680943	-0.74423057731519	3.39289959744029
H	-0.02721459385574	-0.27814965538067	-1.89176786491929
P	-1.66289444671118	0.33288998149079	-3.35382816457756
H	1.99525516144308	3.81798062276211	-1.88090026799226
H	1.87668345633908	5.19431837190446	-0.76558002469071
H	0.54141747752988	4.85610873515537	-1.89600500576058
H	0.55795029723441	5.09686442318598	1.30650049064905
H	-0.64734738109012	3.81032626595336	1.56312331759868
H	-0.88920824275847	5.00319709564585	0.26392794645771
H	2.24769149292604	2.00301005368337	-0.01545964061399
H	1.29174834962864	2.21476259335444	1.46677786826553
H	2.47336251834160	3.45257435704693	0.99241031815920
C	-5.66210358493514	-0.23656042072062	2.79800722841323
H	-4.93745339152550	1.02056055053719	1.20883635612089
C	-4.02960629951491	-1.34057536226298	4.13708696376159
H	-1.96890055919876	-0.95081359007993	3.63461648793112

C	-3.13599119021651	-0.06849158181834	-2.40054032071917
C	-1.92516753391002	1.85490678535464	-4.26710867625760
C	-1.36530713574839	-1.03504042860117	-4.47916838470690
C	-7.10967198736328	0.04068142148715	2.49461320991097
C	-5.36919367222299	-1.09828983528229	3.85527410809403
C	-3.63637725949209	-2.28366125523458	5.24209897199842
C	-2.98440009100219	-0.89014172432201	-1.26975818762356
C	-4.41699899607505	0.30212436070386	-2.81566805316601
C	-1.37964837039073	2.00403361052884	-5.55304356153944
C	-2.56581812921433	2.94104258923015	-3.65843834998485
C	-0.07651670158038	-1.27151688504130	-4.97167941749089
C	-2.43431277940134	-1.82836880298961	-4.93119333925497
F	-7.70568258275433	0.68418974777259	3.50487951964234
F	-7.27005774724999	0.79161063018769	1.40420676122905
F	-7.79900514563498	-1.09090569945263	2.31154467109179
H	-6.16163326099460	-1.56645829734456	4.43993227005285
F	-4.68536075730394	-2.69491840354466	5.95153927631484
F	-3.03232047718010	-3.37720683203260	4.75641379327312
F	-2.77282005836357	-1.71964624733833	6.09323933933189
C	-4.09839065012639	-1.32141086532960	-0.57045496942702
H	-1.98646458491092	-1.19723224110183	-0.94698844403846
C	-5.54399368753861	-0.12605801667730	-2.11386916810600
H	-4.55417506336000	0.92981722159517	-3.69920449027467
C	-1.46974589762074	3.21949448115614	-6.21096989110603
H	-0.87886071003675	1.16485382016202	-6.04136894701276
C	-2.66239617888996	4.16581922398757	-4.31500579717200
H	-2.99361589023444	2.84051987132863	-2.65536242205887
C	0.14738879258214	-2.26419618222366	-5.92225939454206
H	0.77029803636286	-0.68617575885856	-4.60265148485736
C	-2.21653969704105	-2.82224801002004	-5.87038669180529
H	-3.44346365278599	-1.66955478810828	-4.54524328437251
C	-5.38836430182524	-0.94526788038140	-0.98716016454325
H	-4.00211015978493	-1.96478034314119	0.30576334525793
H	-6.53236074943315	0.17740048275272	-2.45487456584079
C	-2.10991882269093	4.31118485284870	-5.59721274927827
H	-1.05453386152460	3.35411581689349	-7.21045914785921
H	-3.16653744474613	4.99548380644694	-3.82207320283118
C	-0.92448464958536	-3.04098713816195	-6.38077136081974
H	1.16427598052877	-2.44808241902741	-6.26675829236240
H	-3.03295589244099	-3.44827083626076	-6.23176329230825
O	-6.41229917460172	-1.41484329539155	-0.25629973746115
O	-2.15318542785963	5.44745743340833	-6.30524157034261
O	-0.80447627918884	-4.01418653024737	-7.29687814804662
C	-7.72820049821869	-1.22597050852792	-0.74291749135334
C	-2.78989976618172	6.57785897713385	-5.74143978694255
C	0.43993128692674	-4.17989822091709	-7.94897442706009
H	-8.39261397606212	-1.73539692541538	-0.03844358288607
H	-7.83668927642824	-1.66589966899736	-1.74628173443622
H	-7.98917138987079	-0.15817803491748	-0.77743054841351
H	-2.29241565371629	6.88752982882299	-4.80964609546651
H	-3.85175612925699	6.37188163329328	-5.53733278973281
H	-2.70940427866966	7.37979411598153	-6.48212563407369
H	1.22314184841607	-4.47775530228725	-7.23648932536880
H	0.29743296415983	-4.97190086538949	-8.69154308769161
H	0.74204728631917	-3.24975764190538	-8.45590726747211

1-2_product

Frequencies, energies and thermodynamic properties:

Lowest Vibrational Mode (1/cm) = -25.08

2nd Lowest Vibrational Mode (1/cm) = 10.46

3rd Lowest Vibrational Mode (1/cm) = 17.08

4th Lowest Vibrational Mode (1/cm) = 22.3

DFT Electronic Energy (a.u.) = -2465.14248572131

DFT//DFT Electronic Energy (a.u.) = -2467.599791694003

Gibbs Free Energy (a.u.) = -2466.992848

DFT optimised cartesian coordinates:

O	-2.13810162316884	1.19359610408474	-2.42450535709084
C	-1.59946629496546	1.12251506774376	-1.34514597945328
N	-2.03224868864427	0.47895907304023	-0.20097224726574
C	-3.30047730914919	-0.27376267449190	-0.07164352711344
C	-1.31324134823421	1.00905529237479	0.90935034660173
C	-1.53690912269364	0.83893175872992	2.27177511817281
C	-0.67853480382629	1.49506076016249	3.16417437804301
C	0.36403533003431	2.30480662352203	2.71696705092905
C	0.57728331540294	2.47275898734381	1.34355340836503
C	-0.26099758196093	1.81696407870301	0.45817224899447
C	-0.24950625385636	1.78948876066976	-1.04024474013458
O	-0.18645609936371	3.06753096879898	-1.59758772469764
C	0.84649017147663	0.85873252599398	-1.63215346766769
C	2.21704057591479	1.30603128054299	-1.24262014053865
C	2.95718736268383	2.25692535676204	-1.87957052966199
C	4.14772652710025	2.50368619433724	-1.11086225185977
C	4.08912054285910	1.66670441788262	0.02597313218383
C	5.10946396250305	1.65151262742773	0.98603893709120
C	6.19146669252509	2.50031404110374	0.77979896099936
C	6.26328899970369	3.34583213115533	-0.34311934739216
C	5.24834027159817	3.35729793454407	-1.28928293065450
H	-2.34461613794136	0.21771992300661	2.65027494725037
H	-0.84174080116821	1.36741403951167	4.23553518303330
H	1.01302053525369	2.80603107536009	3.43585987313874
H	1.38608280048450	3.10523577825422	0.97002082089540
H	5.05470215972278	1.02253349487324	1.87225324801048
H	6.99819950376053	2.51405369022985	1.51425400559353
H	7.12687034072569	4.00121621719110	-0.46574620526197
H	5.29855271528050	4.01310683367224	-2.16004982126537

H 0.76084541993413 0.93991240326564 -2.72589419651533
N 2.89302481386701 0.93809063530539 -0.05580821094188
S 2.59066766795886 -0.38275796671999 0.96435521082846
O 2.91566705431070 0.04716974026613 2.30977270509231
O 1.25957069469204 -0.86907337205702 0.65224875897386
C -3.67817873315433 -0.92938228044642 -1.41888818306014
C -2.69970265614860 -1.63432993072253 -2.13434601149259
C -4.99901451703663 -0.97494427254250 -1.86485808281536
C -3.02963063443053 -2.33603264637890 -3.28782603853734
H -1.66524259390079 -1.63105829448228 -1.78133871367602
C -5.33378226118771 -1.68814657918965 -3.02127890042484
H -5.78446777765799 -0.45854310107657 -1.31253750275250
C -4.35303527831753 -2.36436549766662 -3.73997676418399
H -2.25120776095102 -2.86844131842161 -3.83695588722582
H -6.37265352715755 -1.70981367569985 -3.35486688444109
H -4.61397246589914 -2.91756031868805 -4.64362679212110
C -4.35039634384616 0.74148982101293 0.42053029022470
C -4.71534660393114 1.77423964513381 -0.45909837535946
C -4.87130639513844 0.75838045562184 1.71680225996109
C -5.59091623222904 2.77926194332816 -0.05940450470963
H -4.30582875064269 1.78152262121825 -1.47128396607335
C -5.75062451127961 1.76838708290421 2.12024260338821
H -4.59763554733976 -0.01434606709139 2.43500797039316
C -6.11695790440853 2.77813944274087 1.23540322844073
H -5.86381147375180 3.56830098301943 -0.76209955963784
H -6.14405774153158 1.75997023269337 3.13800892981597
H -6.80437589751386 3.56469376249334 1.55103431893691
C -3.10436728980121 -1.50164256027359 0.83988697562559
C -1.83403108138056 -2.02066715001592 1.10959086159417
C -4.22811737260823 -2.21417787134646 1.27968490933551
C -1.69147222146606 -3.20264319623549 1.83951547862993
H -0.93764032476927 -1.50821931572043 0.75918549775170
C -4.08537748155658 -3.39197530400308 2.01045069355192
H -5.22982473896246 -1.85358499790888 1.03642511948079
C -2.81322468260975 -3.88925118968402 2.30001045491006
H -0.68920128960939 -3.58282669709252 2.04522767233310
H -4.97453192517643 -3.92696261196470 2.34810541476447
H -2.69972738117704 -4.81140153209330 2.87229324142404
C 3.78146880638212 -1.57951172866494 0.44013496943800
C 4.84933306309149 -1.89166249653645 1.27674708346251
C 3.61376875411533 -2.19586136518549 -0.80205581298561
C 5.77881009271506 -2.83785224021181 0.84771912409915
H 4.94375224348329 -1.41112943568949 2.25135537152819
C 4.55404731479358 -3.13341084664440 -1.21118133196958
H 2.76018046696805 -1.94887037003709 -1.43749560587310
C 5.65065289535111 -3.46457465946817 -0.39754161738983
H 6.61788718251614 -3.09600710965985 1.49652743887415
H 4.43538247067795 -3.62521007552069 -2.17877944709347
C 6.67496743914530 -4.45878604084990 -0.87043546323221
H 6.20107380781996 -5.28007463360845 -1.42437968249285
H 7.38919117332210 -3.97026257948470 -1.55133445494653
H 7.24282165493458 -4.87536411985916 -0.02896045714803
H -0.49390342398117 2.99364857067565 -2.51488300959950
H 0.64572638237750 -0.18015996567982 -1.34924230604445
H 2.65662137243923 2.75279383271284 -2.79943875205998

2-3_product

Frequencies, energies and thermodynamic properties:

Lowest Vibrational Mode (1/cm) = -38.69
2nd Lowest Vibrational Mode (1/cm) = 15.72
3rd Lowest Vibrational Mode (1/cm) = 18.87
4th Lowest Vibrational Mode (1/cm) = 21.68
DFT Electronic Energy (a.u.) = -2465.127937930392
DFT//DFT Electronic Energy (a.u.) = -2467.583903843287
Gibbs Free Energy (a.u.) = -2466.976658

DFT optimised cartesian coordinates:

O -1.14202406594498 2.08832479092167 -0.79548166063187
C -1.12408662942837 1.45905599829576 0.23632175305818
N -1.73028208696269 0.25080752511289 0.52164690513773
C -2.63221569881109 -0.48031384243216 -0.40731136793972
C -1.79113612557068 0.10335037800172 1.93784740144773
C -2.44465712130988 -0.86050879486723 2.70253155411800
C -2.35765092954716 -0.76417128252758 4.09719354769617
C -1.64352363245478 0.25891950649702 4.72042823485067
C -0.98800765082586 1.22106539749086 3.94396961303410
C -1.07326006871414 1.13056018243255 2.56479861756371
C -0.44431300813535 1.99082834217422 1.51334920983087
O -0.73574351863170 3.34713112121407 1.66906431471469
C 1.49046476843603 -0.66387139759661 1.98523250223202
C 1.50901012260673 0.35699961424657 1.12313185281086
C 1.11184750464839 1.78607115686704 1.44833944620293
C 1.70973346218269 2.56702007526370 0.31158645257513
C 2.10912530345334 1.68638691623803 -0.70207745061061
C 2.59650695248714 2.16391435218124 -1.91736421477876
C 2.70568753659358 3.55051517494699 -2.07489323985352
C 2.33701332503775 4.43436082963565 -1.06175240251961
C 1.82704539640183 3.94058975888115 0.14342267203354
H -3.00855145864242 -1.67214080346366 2.25068584818636
H -2.86801171737195 -1.51339820004852 4.70454749626330
H -1.59930916795752 0.30974736215442 5.80888888380346
H -0.42211871927764 2.03158942843314 4.40807429639874
H 2.88572906285492 1.49193960233934 -2.72078750989255
H 3.09293643002744 3.93956630758040 -3.01796885919481
H 2.44238698877195 5.50970269944334 -1.20963544842286
H 1.52628457586053 4.61727127542707 0.94485378053429

H 1.73920500345493 -1.68514508799462 1.70875315281853
H 1.18296824061213 -0.45850634434725 3.01083553303412
N 1.90047523484351 0.34573915895442 -0.25488108203107
H 1.49003516223499 2.09511722935847 2.43363060253428
S 2.63859668789730 -0.99691046958151 -0.95938737627247
O 1.87296436957197 -2.15546544274271 -0.53683464734565
O 2.76451073598450 -0.69346787048879 -2.37402833163281
C -2.19172839707502 -0.27388426048536 -1.87389930779029
C -3.11386027449654 -0.07436314688298 -2.90101193219608
C -0.84148251786308 -0.44966453310490 -2.20538893233580
C -2.68746581438655 -0.01410302468319 -4.23299909385063
H -4.17572728777451 0.03149556006252 -2.67807496215937
C -0.41390944371633 -0.37994164126841 -3.52565876858881
H -0.11843569022317 -0.65267312974168 -1.41357972087394
C -1.34010313258476 -0.15585030537381 -4.55007625361191
H -3.42347503212987 0.14471321118436 -5.02303317140504
H 0.64665673459330 -0.51292467251558 -3.74651241113356
H -1.01006825273504 -0.10457911232376 -5.58896249957770
C -4.04639639604158 0.05807311425846 -0.11738168555553
C -5.02265363315989 -0.65668742274113 0.58189213708975
C -4.32074711266426 1.39020978131858 -0.47005048534397
C -6.24909925071071 -0.06607500456336 0.90261584585794
H -4.84132046690164 -1.68588496835431 0.89051304459518
C -5.54075678512243 1.97918389331587 -0.15163986314468
H -3.56439757538035 1.96703554997254 -1.00559162570726
C -6.51479741083211 1.24972991423950 0.53512698946116
H -6.99536355616744 -0.64580545910944 1.44823450241297
H -5.73199736488120 3.01373580763181 -0.44139180015315
H -7.47267324779998 1.70897938867676 0.78489737680480
C -2.45673385205325 -1.99950917065579 -0.23022123522728
C -3.39795385930357 -2.87564041009454 -0.78614339929886
C -1.28805031998798 -2.53230159220773 0.32209568755659
C -3.19852028472065 -4.25435496922947 -0.74400590084842
H -4.29070754428323 -2.47812892185145 -1.27317325362028
C -1.08383293440252 -3.91239818267493 0.35636981041555
H -0.51576003543417 -1.86988603724481 0.71578003517262
C -2.04160877527044 -4.78002062023275 -0.16565092476536
H -3.94756662676431 -4.91975627910642 -1.17642955707956
H -0.16140181196133 -4.30458948466476 0.78821658887769
H -1.88284661846836 -5.85920512970710 -0.13607146902589
C 4.24854971110208 -1.06374739109877 -0.23752443440889
C 5.21866633200301 -0.15787245904670 -0.67881880851972
C 4.51780411292398 -1.99456198020466 0.76260912546737
C 6.47317190352303 -0.18380548524875 -0.08335105599361
H 4.99684154334170 0.54878939796723 -1.48071895322014
C 5.78619568250197 -2.00608274526514 1.34201947197445
H 3.75438233426757 -2.70986430962340 1.07025298205713
C 6.77500008246432 -1.10292338448347 0.93587648215593
H 7.24052703778094 0.51698344015339 -0.41870658756112
H 6.01092731304385 -2.73478004122109 2.12308895544422
C 8.14026253962662 -1.10800449508366 1.56649045828508
H 8.33351967540068 -0.14976118788535 2.07094768527543
H 8.23583021060754 -1.91450846787513 2.30379606812937
H 8.91933705309186 -1.23629990359291 0.80128889438736
H -0.71788614715159 3.74589958926455 0.78346004342318

BIMP
Frequencies, energies and thermodynamic properties:
Lowest Vibrational Mode (1/cm) = 10.77
2nd Lowest Vibrational Mode (1/cm) = 18.0
3rd Lowest Vibrational Mode (1/cm) = 21.48
4th Lowest Vibrational Mode (1/cm) = 23.87
DFT Electronic Energy (a.u.) = -3120.345161061669
DFT//DFT Electronic Energy (a.u.) = -3123.301400370568
Gibbs Free Energy (a.u.) = -3122.677561
DFT optimised cartesian coordinates:
H -1.26958713075950 -3.32215865742959 0.09189219077029
N -1.25566032689036 -2.46627927547970 -0.45509128666953
C -0.34798020925840 -2.49470481976358 -1.48824407382812
C -2.27485259260624 -1.54244278456614 -0.18608071253590
N -0.08433833739655 -1.32809911907247 -2.08588811113123
S 0.37668041454051 -3.96357375587535 -1.88041085966081
C -2.77220038864420 -1.46725120181382 1.12077725008508
C -2.86582323722608 -0.75486760312565 -1.18592324885774
C 0.88238848957986 -1.12584809986068 -3.16152441376079
H -0.38895130068268 -0.47998555655056 -1.60928893495877
C -3.85728583288320 -0.64403760247606 1.40985488975522
H -2.30672539655479 -2.06427462382074 1.90778030448833
C -3.92529556914185 0.08443238101451 -0.86400612093829
H -2.51981680981219 -0.81311952203935 -2.21614350216222
C 2.24331730919876 -0.75053786210383 -2.54896593895808
H 1.01952192520855 -2.11010185742969 -3.63212835503944
C 0.29760086203045 -0.19225264489454 -4.25532267792151
C -4.39707816539202 -0.62890356965593 2.81593280758560
C -4.45236247803443 0.13716809279112 0.42583535679194
C -4.51206723333675 0.98842221569548 -1.91508347281242
N 2.25527049247631 0.45220210157142 -1.75018722072823
H 2.96362224979416 -0.66220561972525 -3.37968900864260
H 2.55818069228306 -1.64749164994191 -1.97693189867981
C -0.23298703662175 1.13607154250992 -3.70083370356058
C 1.39175708646393 0.11024164681132 -5.28486666353318
C -0.84790059573747 -0.93638662652126 -4.95476523712060
F -5.15239846063197 -1.70809988360867 3.05523273218611
F -3.41698082953901 -0.64146025541957 3.72089461124879
F -5.16022427947693 0.43850100281046 3.04976663396156

H -5.30310821561729 0.77882086523521 0.65862852365558
F -4.09530612807239 0.67978278561036 -3.14227405834450
F -5.84503847796584 0.94589841066944 -1.91636586920485
F -4.16849888300858 2.26610113230056 -1.69310345133807
P 2.30945656558311 0.45500146416091 -0.16871643567654
H 0.55842965769986 1.67488584765270 -3.16373401422496
H -1.08889034781308 0.98996061220131 -3.02035424734786
H -0.59288965363841 1.76158599242408 -4.53220065420015
H 0.95385277800211 0.62782028764555 -6.15146201076499
H 1.86696238524862 -0.81638523056010 -5.64380130799658
H 2.17227453022650 0.75864476616358 -4.86090067519109
H -0.48372550017299 -1.85808191686029 -5.43398862482186
H -1.30116939027033 -0.30001975721477 -5.72984109478480
H -1.63886742801878 -1.21550810323094 -4.24175594359590
C 0.71929476791637 0.80118843984016 0.67211934390144
C 2.95022036443791 -1.06217671576598 0.59881632949475
C 3.39541795740297 1.80251981161401 0.38449500347345
C 0.41322402709841 0.40383088056947 1.98487303578369
C -0.19407426506478 1.60944955088934 -0.01129094472819
C 2.10824878140275 -2.14820843485855 0.85305422044659
C 4.33394387181798 -1.22375972454105 0.78687083018741
C 3.49280123053442 2.16136906616385 1.73936134559723
C 4.17964192016708 2.47343320210011 -0.55212726958933
C -0.76904186044844 0.80899329698881 2.58781949405043
H 1.10764783645254 -0.22310245138539 2.54886734922890
C -1.38403351207981 2.03068502485256 0.58356000993968
H 0.03345748218210 1.92773633913932 -1.03151132480349
C 2.61387477329818 -3.37000557673831 1.29747763928537
H 1.02899043726680 -2.04873157860645 0.71421377702105
C 4.85020801784971 -2.43021321358069 1.23287352598570
H 5.01404879121186 -0.39246132630837 0.58473039120489
C 4.36191771543404 3.16242286523955 2.14047559316761
H 2.88453366028128 1.65203336032835 2.49138764297687
C 5.05459168983213 3.49062439121917 -0.16300248901676
H 4.09712515934680 2.19258391315212 -1.60350640801508
C -1.67184145662600 1.63184898426135 1.89421938331798
H -1.02075245623584 0.50512395603803 3.60437587603572
H -2.07453033397391 2.65677993120566 0.01949454105850
C 3.99348134137598 -3.51498016528954 1.49043672862829
H 1.92807191925122 -4.19719315601474 1.47410861256835
H 5.92096852784070 -2.56611992169090 1.38963061494861
C 5.15095535924342 3.83634845757915 1.19023194196879
H 4.45240132727989 3.45048599844988 3.18856340832170
H 5.65267926206946 4.00016148801604 -0.91708585486624
O -2.79166775672194 1.98086745712486 2.55613550657835
O 4.57779528354601 -4.64866475901707 1.91619428387312
O 5.96649750882200 4.79440778942776 1.66774572194509
C -3.61163495216436 2.99159469097899 2.00569586695022
C 3.76159230329195 -5.77262452972015 2.17209604833094
C 6.78264565001387 5.49985011513087 0.75679423149076
H -4.43280126736471 3.14487108642613 2.71363126992716
H -4.02383271401177 2.69271228890030 1.03018524531252
H -3.04420317808377 3.92734189209497 1.88199396644051
H 4.43109327996840 -6.57646077648542 2.49538109032661
H 3.22335742001087 -6.08700968648570 1.26433464140389
H 3.03295969869545 -5.56058404363169 2.97012875969468
H 6.17472971948345 6.03999677074528 0.01427455897756
H 7.35923820575025 6.21940189680259 1.34734728326253
H 7.47267814096731 4.82008064031388 0.23284982127565
----
associated_product_HBIMP
Frequencies, energies and thermodynamic properties:
Lowest Vibrational Mode (1/cm) = 7.88
2nd Lowest Vibrational Mode (1/cm) = 10.87
3rd Lowest Vibrational Mode (1/cm) = 13.48
4th Lowest Vibrational Mode (1/cm) = 16.79
DFT Electronic Energy (a.u.) = -5585.50470905794
DFT//DFT Electronic Energy (a.u.) = -5590.906476604847
Gibbs Free Energy (a.u.) = -5589.646914
DFT optimised cartesian coordinates:
O -2.57625585792286 -0.41515564088964 0.82679823742313
C -2.38087117563310 0.61849561213361 0.21376513080837
N -3.20906559259468 1.15684990148526 -0.74016654034383
C -4.56063797218613 0.58793941271474 -0.97871318140221
C -2.74722422091700 2.47151444620183 -1.05640617775317
C -3.36976927060588 3.47967349867765 -1.79096602388381
C -2.69242983245670 4.69421905719314 -1.95958617150850
C -1.43054200414502 4.90175990754594 -1.40859656830624
C -0.83074201646419 3.88912381034054 -0.65089416059412
C -1.49000909330644 2.68473390544585 -0.46751040237220
C -1.10237981369256 1.47702895879838 0.37787508780959
O -0.03160682732269 0.76391376117161 -0.04127980981373
C 1.24657698871730 1.00940064006816 2.67403168302264
C 0.37822561549319 2.00088551901180 2.45505350991642
C -1.02894482233489 1.80656785240848 1.94490249463403
C -1.70009910230244 3.09869069335371 2.30497498154197
C -0.73326014886024 4.05067606103950 2.65559978190399
C -1.07796266819252 5.38144320462793 2.88110842446720
C -2.42883881594054 5.73104227469628 2.75967009104341
C -3.40362255536008 4.78682126590626 2.43452209422187
C -3.03732576340279 3.45676013525763 2.20309472806865
H -4.35462632958206 3.35559348230207 -2.23240728276430
H -3.17460886834368 5.48746970191053 -2.53327204596942
H -0.91886263486179 5.85499870820539 -1.54893154250096
H 0.13607913503916 4.06042844553906 -0.17886915337588

H	-0.33167072532402	6.12463288568766	3.14826708945031
H	-2.71820837999165	6.76864461992795	2.93464553588885
H	-4.45091027688692	5.08362237087417	2.35646066265831
H	-3.78929011340764	2.71124494715824	1.92610896362828
H	2.23789679012753	1.15222969784013	3.09629147671307
H	0.91041626170658	0.00428918890585	2.42314163523284
N	0.53922332998746	3.40457186208450	2.71334323553307
H	-1.46630092521394	0.92940022583504	2.44043744636283
S	2.00950041979550	4.18464637097915	2.81965449511240
O	1.79031799969906	5.44978684392888	3.49977040016980
O	2.95558936189328	3.23954302276391	3.39360264897347
C	-5.10476857296275	0.98418932362568	-2.36274042292021
C	-4.23607587537794	1.06371316642228	-3.45852732463094
C	-6.47884854021392	1.10689171966444	-2.58577914922339
C	-4.72454526933119	1.30757098465742	-4.73911646584061
H	-3.16254785564245	0.94683786123180	-3.30751461590614
C	-6.97110431635153	1.34759694533558	-3.87099350409555
H	-7.17925565224356	1.00942208658909	-1.75505024578979
C	-6.09719813230042	1.45712934454146	-4.95073103707545
H	-4.02726416576405	1.37867287842895	-5.57588178434567
H	-8.04711033614706	1.44644888737887	-4.02325632378319
H	-6.48197675575575	1.64923814493695	-5.95354378783682
C	-5.40036574059595	1.09119915095892	0.21019623660156
C	-5.43159903082503	0.38272177433888	1.42063099608639
C	-5.98456847257370	2.36595734495778	0.17232521146862
C	-6.04176563575756	0.92828299224808	2.55108762544599
H	-4.96385071167290	-0.59858447235552	1.48571005363574
C	-6.59381588363198	2.91130590972839	1.30302169150059
H	-5.97061016498756	2.94870546297190	-0.74854898171489
C	-6.62545595076513	2.19406871210179	2.49856065170249
H	-6.05494026147953	0.35642849024926	3.48041352129419
H	-7.04309013076015	3.90419109788527	1.24494353373880
H	-7.10070225416785	2.61913686067761	3.38415645603083
C	-4.47546524660254	-0.94919442368565	-1.11783676194445
C	-3.37266188169921	-1.54122134339690	-1.74896175006417
C	-5.56632281466942	-1.75550638033627	-0.78598216457646
C	-3.34998319452960	-2.90731602649159	-2.01587741661758
H	-2.50741681499285	-0.93199475919696	-2.02293327837841
C	-5.54082119068247	-3.12969151848923	-1.04243467905940
H	-6.45270659997889	-1.31605521612451	-0.32574531956925
C	-4.43437276624825	-3.71218889604414	-1.65534828920477
H	-2.46554273144333	-3.34833537127386	-2.48394022901238
H	-6.39535094951423	-3.74404668874917	-0.75451156629853
H	-4.41161349024097	-4.78654439083739	-1.84575752383039
C	2.51792450978931	4.52562471580323	1.15601117154458
C	2.47791943640618	3.53012864754696	0.17707784766869
C	3.10374828083624	5.76680146879539	0.91161188645108
C	3.04878924652288	3.79852728972244	-1.06375751217047
H	2.00064977213176	2.56120330629910	0.36265587310266
C	3.68496272505652	6.00511660019591	-0.33088080763015
H	3.11447580996291	6.52738669590921	1.69358133660032
C	3.68025345736213	5.02154731959036	-1.32833849550244
H	3.03139058947943	3.03038319877317	-1.83974670802695
H	4.16260693243986	6.96820996024005	-0.52501091508121
C	4.37796083624376	5.27001403404630	-2.63602186492431
H	5.46010141737178	5.37653399423159	-2.46142821540715
H	4.21979319747928	4.44253591704616	-3.33929677283229
H	4.02726428480954	6.20283394419551	-3.10033741103454
H	-1.02562326769396	-1.52524039137658	0.19541488549701
N	-0.64065466550818	-2.41193278837138	-0.14087154848874
C	0.03668555520007	-2.35930135765021	-1.33165183773328
C	-1.10717808236320	-3.53699692354876	0.55457151274327
N	0.17963668514682	-1.11373879601768	-1.78614580831150
S	0.58268826599224	-3.75690830886031	-2.13335595815835
C	-0.29064884699829	-4.63280626424331	0.84605811624450
C	-2.42715438903201	-3.51702524658766	1.02883511375498
C	1.13231720976527	-0.69311506528132	-2.79271866296207
H	-0.08178308768810	-0.32095451452904	-1.10144420332532
C	-0.80081747704594	-5.69194389594591	1.59853867471326
H	0.74246987894570	-4.64401273351461	0.49901994249176
C	-2.91561834299721	-4.58905547694800	1.76546712773496
H	-3.05941777368901	-2.65515166038305	0.80806823803647
C	2.17286931305923	0.23499937681927	-2.11457462403267
H	1.64338828084797	-1.59280064894587	-3.16550692751670
C	0.43275937829444	-0.02450726867398	-4.00402530721553
C	0.12213818578529	-6.83669207974309	1.91523169076272
C	-2.11276739607570	-5.69146155548204	2.06190800066616
C	-4.35645904277291	-4.59284250344397	2.19797480923612
N	2.46759378294177	-0.18602743375767	-0.74547368922999
H	1.77654402870877	1.26031149859261	-2.07695749944954
H	3.09604443529690	0.25163395260703	-2.71298656958780
C	1.49314031511627	0.42895497080422	-5.01325047361198
C	-0.47912988331906	-1.06629932336305	-4.66125662617390
C	-0.41775134432979	1.17346910031257	-3.56403364665837
F	-0.40898477778858	-7.68834997691389	2.79158416455874
F	0.44232329858818	-7.53709793573835	0.82418665379038
F	1.27808247990157	-6.39821823569623	2.43497374732908
H	-2.49862873140480	-6.52391949486270	2.65099727897845
F	-5.11959050385514	-5.30548702647725	1.36013681618092
F	-4.50734510535768	-5.13845305139039	3.40774042605049
F	-4.87296450547380	-3.36310698343102	2.24518084722388
H	1.73679805755100	0.12661255890706	-0.08290927519944
P	3.97411655464983	-0.42725394250545	-0.12468035009276
H	2.15137497711918	-0.40851249895239	-5.29460310493114
H	2.11769604248304	1.24191182697517	-4.61304756992180

H 1.00763315991192 0.80281471855837 -5.92717975825021
H -0.98260362062023 -0.63212884258899 -5.53862037869775
H -1.25039815440599 -1.41200616295055 -3.95568356027225
H 0.09672257367951 -1.94582271481248 -4.98725711723045
H -1.02169305458332 1.53737142322350 -4.40993941292325
H 0.19188752384716 2.01673079228231 -3.20511675249393
H -1.10045227424111 0.88944230474399 -2.74764415722441
C 4.84594364771193 -1.54767280095586 -1.22733879426484
C 4.95521895646795 1.06679929037268 0.07674463695818
C 3.69027940270107 -1.23560041578322 1.45374834511177
C 4.10340522038795 -2.52483599137798 -1.89807029818188
C 6.24878197533695 -1.56548572295891 -1.32040890862769
C 4.86845946821064 1.82610347655460 1.24890772717786
C 5.66913476902047 1.58723650941878 -1.02099396207029
C 4.65628238904314 -1.21399962955199 2.47442537165639
C 2.53818122945247 -2.01440914480308 1.61565671049865
C 4.73011541687952 -3.48176081108095 -2.69125425428356
H 3.01602695633289 -2.56736302780150 -1.79563669058158
C 6.88087519866408 -2.51557618382879 -2.10488297929830
H 6.85398986969461 -0.83729576352946 -0.77703747684655
C 5.49558568735567 3.06558557395069 1.35081729379818
H 4.27646874417465 1.47999375868319 2.09831986453871
C 6.29301522362866 2.81868311866275 -0.93031749663292
H 5.73394068570426 1.02990576604989 -1.95819065836332
C 4.44957903436864 -1.92689615507065 3.64337799555701
H 5.57603628438747 -0.63657793883456 2.35787973877343
C 2.32462580570298 -2.73692365452830 2.78670164318958
H 1.78317177218679 -2.05705321155723 0.82657159243952
C 6.12819955375502 -3.47548197432898 -2.80633344260771
H 4.11910695189817 -4.22420813612290 -3.20336180066756
H 7.96724665157787 -2.54092002060594 -2.19502171399570
C 6.20755037686869 3.57164833167556 0.25571985130156
H 5.37631152538466 3.63739412265921 2.27002318196126
H 6.85080317193188 3.23153634689713 -1.77164529015578
C 3.27990126610826 -2.69094136082412 3.81303663008920
H 5.18349752608165 -1.91428335998471 4.44960600789427
H 1.41687579443559 -3.33123944015976 2.88694570757843
O 6.82343832197503 -4.34637723001034 -3.54949872703755
O 6.81899453906321 4.76477086685981 0.24405097345781
O 3.16023892533111 -3.34491827442920 4.97478714305341
C 6.11930836295778 -5.34395282872323 -4.26410152896043
C 6.82476426079201 5.53251988860280 1.43266109163407
C 1.99928963102975 -4.12364557431528 5.19938497711822
H 5.42516324828517 -4.89357595427075 -4.99002641590123
H 5.55738899122454 -5.99724866535586 -3.57935456528282
H 6.87260485566904 -5.93361573062368 -4.79614143787708
H 7.39746261986041 6.43940072374435 1.21330084453734
H 7.30753737259632 4.97974675105256 2.25311165399740
H 5.80120927625355 5.80702618096488 1.72975525192540
H 1.90996313531312 -4.92536003954885 4.45126528188132
H 2.10981701679885 -4.56237214686780 6.19607943101618
H 1.09466680061856 -3.49689881463012 5.17185843565136
----
associated_substrate_BIMP
Frequencies, energies and thermodynamic properties:
Lowest Vibrational Mode (1/cm) = -33.84
2nd Lowest Vibrational Mode (1/cm) = -8.82
3rd Lowest Vibrational Mode (1/cm) = 8.14
4th Lowest Vibrational Mode (1/cm) = 14.35
DFT Electronic Energy (a.u.) = -5585.505093298697
DFT//DFT Electronic Energy (a.u.) = -5590.904619633416
Gibbs Free Energy (a.u.) = -5589.641762
DFT optimised cartesian coordinates:
O -1.33846531004056 -0.51660097572565 0.39302916653897
C -1.52001567304602 0.55353450138318 -0.15336120718595
N -2.68587999272136 0.98572840760837 -0.73531107458671
C -3.95924871406398 0.23001224832215 -0.57356386445886
C -2.58091118307114 2.39055369755037 -1.00583296214868
C -3.55226476951421 3.31004087588027 -1.40273024210840
C -3.16537038949575 4.64328639800294 -1.58808445036848
C -1.84769489256855 5.05915943450153 -1.40375648277451
C -0.88383391490592 4.13672627922001 -0.98995136985823
C -1.26670914733093 2.82114912294310 -0.77219655168269
C -0.47342397555729 1.67895860487963 -0.20521725023517
O 0.10341809883638 1.87061056033829 1.05936320099862
C -0.76201087270198 2.18600467892334 2.13827843924473
C -1.07268851113230 3.64890952155442 2.22048942950750
C -2.29832470641317 4.23088452019108 2.11938904806165
C -2.12138920262493 5.65675712294355 2.15876370676943
C -0.73588147157807 5.90600591616438 2.30077340947821
C -0.23142083287718 7.21333657033694 2.36158316380444
C -1.14204894485044 8.25797962742051 2.25511178332465
C -2.52227441674010 8.02583535109274 2.10327983356350
C -3.01967871786564 6.73200291278497 2.06068785520727
H -4.58905814921790 3.03257194951427 -1.56906903222787
H -3.92420479936440 5.36643333983316 -1.89145228918651
H -1.57347281709742 6.10307842180620 -1.56218324358638
H 0.14590155529025 4.44890601310257 -0.80777903108563
H 0.82731952350346 7.41220866809262 2.51002326043655
H -0.77237702652339 9.28371003215231 2.29788492670652
H -3.20383438939811 8.87407537389770 2.02453494909001
H -4.08874357089642 6.54275872198607 1.94822029371881
H -0.24443749730228 1.83842804128437 3.04025011506422
H -1.72409375983284 1.64954893813794 2.06167127936351
N -0.09707240776298 4.65741207107407 2.35438004107021

H	-3.23070221580108	3.68433822426939	1.98819263433338
S	1.58650043188497	4.43557564301915	2.48488504777890
O	2.06659259272316	5.59386418909107	3.22007661362209
O	1.78182846010274	3.10380595674847	3.02047862536359
C	-4.94584911451116	0.61710524884682	-1.68831192538345
C	-4.46304466822385	0.83606783380198	-2.98569882880219
C	-6.32589768756702	0.60594780749614	-1.47388618873175
C	-5.34137551925168	1.08839443653760	-4.03550746580003
H	-3.38664966516515	0.81890171852084	-3.17403581323870
C	-7.20765461341428	0.85753698957285	-2.52818887630083
H	-6.72531824725804	0.39512254792111	-0.48089936280690
C	-6.71998597820089	1.10831096290464	-3.80904067614368
H	-4.94690685562812	1.26909666552337	-5.03669319285341
H	-8.28264974913126	0.85286960621323	-2.34106472763190
H	-7.40926950122210	1.30844542031951	-4.63080764090925
C	-4.41432391328765	0.54760271940443	0.86237911365706
C	-3.95803094507783	-0.23731789574352	1.93241099083923
C	-5.14000566427589	1.71052578917791	1.15373681580736
C	-4.22791032234215	0.12467493964181	3.25177932810145
H	-3.38415668295123	-1.14110624492246	1.73360343488620
C	-5.41306676192147	2.07114275740356	2.47471814228640
H	-5.50756235102144	2.34707814560201	0.34908369208965
C	-4.95596361861999	1.28195756811159	3.52943969244676
H	-3.86646210069001	-0.50781897420538	4.06469197453157
H	-5.98400075215689	2.97925979155340	2.67578868221535
H	-5.16933494343321	1.56496514647541	4.56145461698079
C	-3.77976085788573	-1.27902374984974	-0.84577960806995
C	-2.83702461027560	-1.75190379264481	-1.76411771346066
C	-4.71187367140040	-2.18088398460621	-0.32063584081716
C	-2.81429807168450	-3.09518292914059	-2.13589098899484
H	-2.10148626471866	-1.06810416184022	-2.19274808827813
C	-4.69177017301949	-3.52615879384805	-0.69194079328140
H	-5.47406554943192	-1.83290467400381	0.37885781137329
C	-3.74198928089492	-3.99000892276986	-1.60039809201167
H	-2.04972098976306	-3.45016101645815	-2.83170798049965
H	-5.42306124955006	-4.21172595084938	-0.26192356820104
H	-3.71756562227747	-5.04330771175757	-1.88485388622571
C	2.18404369455448	4.53357872623846	0.82679859559310
C	2.42483217398318	3.36288506724103	0.10894419152407
C	2.34819857887919	5.79454152832918	0.24840539487156
C	2.80360274125705	3.46383834798652	-1.22572662347234
H	2.30479394188898	2.38836163945407	0.57900540067981
C	2.72874397797714	5.87160566534481	-1.08911366571492
H	2.18231991415068	6.70300090920683	0.82815885241376
C	2.95397599140788	4.71204549451600	-1.84578354531164
H	2.98432175083409	2.53427286216543	-1.77158317617459
H	2.85435835712944	6.85157301541435	-1.55356148411073
C	3.38080105827026	4.79230520707076	-3.28548172933541
H	4.45115709495157	4.55070390427601	-3.37686708301757
H	2.82847850357695	4.06462887184147	-3.89674896156861
H	3.22209311078102	5.79714006054848	-3.69668310685158
H	-0.29886256048974	-1.99297977950630	-0.22488617189956
N	0.04171457422929	-2.86791465388535	-0.63197939268415
C	0.60824081106458	-2.75074922354049	-1.86746521083304
C	-0.38488151422997	-3.99542204549986	0.07199962657736
N	0.86566832579026	-1.46768767556338	-2.17990540256122
S	0.91471993160465	-4.05989789209600	-2.89161112731066
C	0.31698152970754	-5.20351293977773	0.10837253433944
C	-1.54131401083499	-3.84667531925979	0.85049590825413
C	1.55355073725504	-0.95089802654733	-3.34924397812036
H	0.95672901426288	-0.82538010259210	-1.38499059445849
C	-0.14426380788241	-6.23546005758381	0.92375035989840
H	1.22763055278971	-5.32183689783897	-0.47593811718725
C	-1.98393381515464	-4.89398487442431	1.64784519331243
H	-2.09021855797881	-2.90198116724407	0.80779814267481
C	2.71806093225038	-0.01353921967433	-2.84976552732840
H	1.98269818557398	-1.81455454230098	-3.87967619472513
C	0.57956374172996	-0.25012070429455	-4.33712308997605
C	0.68237065645427	-7.48715938532582	1.03395880570394
C	-1.29272983914855	-6.10320675115414	1.69984628891071
C	-3.25377020077943	-4.75160023224259	2.44003861091167
N	2.73051646922152	0.19932486840428	-1.42526264993385
H	2.60839293286869	0.96264785254064	-3.35062089163485
H	3.66665014753029	-0.43289768426852	-3.23139476249359
C	1.36773109349133	0.22972505922917	-5.56282188366535
C	-0.47508006499823	-1.25413785004126	-4.81297986520681
C	-0.12220967965602	0.93544683032311	-3.66472120546555
F	-0.04801483812703	-8.53920358072778	1.40718137388809
F	1.27751105104009	-7.79734446173302	-0.11896849021860
F	1.65494674523063	-7.34898666773722	1.94570986029219
H	-1.63746755910939	-6.92190722342742	2.33288086513809
F	-4.27375095318456	-5.39303982224251	1.86069290515743
F	-3.12848048557430	-5.26529469010490	3.66768004989441
F	-3.62357485692524	-3.47391165261690	2.58114980207125
P	3.72972055886014	-0.48184158460831	-0.38957972131598
H	1.92650558586192	-0.60437645735210	-6.01573262606305
H	2.08001768208360	1.03065593617079	-5.31928070940558
H	0.67304654426796	0.62261954914623	-6.32045576590688
H	-1.19241128931314	-0.76062280741609	-5.48713452972233
H	-1.03470932249236	-1.67588448688341	-3.96585632582284
H	-0.00680745937137	-2.09069422871223	-5.35270017838015
H	-0.76957598246334	1.45653366654580	-4.38703069544137
H	0.59472565055145	1.67012193057046	-3.26256850601404
H	-0.76323358268295	0.59011702666594	-2.83670045494464
C	4.93979581715872	-1.61840947373141	-1.11954786800769

C 4.63336898739731 0.81263135662791 0.50710331006490
C 2.84233446320557 -1.46587781048076 0.85825396503999
C 4.45113486869690 -2.63676582690547 -1.94783978638857
C 6.31881044096544 -1.55477753140749 -0.87036702474704
C 4.39498922688555 1.11785193825895 1.84697504773369
C 5.43056932915654 1.68827996385104 -0.25493066577193
C 3.20072004532089 -2.78286179249128 1.19324981858443
C 1.70193863544268 -0.91032375652534 1.44559932917597
C 5.30442008669579 -3.57365513900240 -2.52561010607683
H 3.37778347237139 -2.72772872451234 -2.14239094968775
C 7.18100958365297 -2.48098621706404 -1.44131391187596
H 6.72529163658152 -0.77911670740314 -0.21809898926459
C 4.89330773113937 2.29163986302912 2.41847947394723
H 3.77745946762496 0.45907073957823 2.46060854430929
C 5.95728229595427 2.83749257347996 0.30667569492247
H 5.61487103529560 1.47753564125782 -1.31272494119438
C 2.42352830017709 -3.52200767464275 2.07382759949720
H 4.08766519880457 -3.24355478137948 0.75389438389981
C 0.92304378092886 -1.63733750087721 2.34587599090070
H 1.38517060804986 0.10521741306502 1.19303237139011
C 6.68261312788477 -3.49591974346487 -2.27450891861857
H 4.88468672765232 -4.35355210216644 -3.15972521900728
H 8.25492434604487 -2.44383601587787 -1.25463376454520
C 5.66077485144737 3.16608896395799 1.64269695099575
H 4.64942844295306 2.52415872228421 3.45343252109590
H 6.56619865830146 3.52966998973203 -0.27662796601032
C 1.27379103760687 -2.95852597707414 2.64993580423427
H 2.66945698904341 -4.55721686624833 2.31560245312849
H 0.02992267277973 -1.17596764104019 2.76559009102585
O 7.58747472819576 -4.34852519078748 -2.78424172009042
O 6.14389651927500 4.33907220138161 2.08954343998619
O 0.55379389048875 -3.76115815830160 3.45635728310665
C 7.13628070133799 -5.38838849041446 -3.62781270646417
C 5.71989717351149 4.79575993285376 3.35819569430601
C -0.53372407136559 -3.20308776832999 4.16245279093740
H 6.63640572360718 -4.98463106120692 -4.52189073736924
H 6.44255183455258 -6.05766265343107 -3.09584174272998
H 8.02529396846504 -5.95132552274828 -3.93039310637061
H 6.16588537997112 5.78624015609351 3.49728355808874
H 6.06470465780826 4.12602398732388 4.16114692523136
H 4.62163790119057 4.87874783071639 3.39615364342414
H -1.29231904136358 -2.79314225571608 3.47708875467674
H -0.97948034210816 -4.01687128631247 4.74456675324097
H -0.19405161375600 -2.40285437692185 4.83924970308875
H 0.36095518821261 1.36680329925305 -0.86050852796705

----

associated_substrate_HBIMP

Frequencies, energies and thermodynamic properties:

Lowest Vibrational Mode (1/cm) = -7.55
2nd Lowest Vibrational Mode (1/cm) = 9.44
3rd Lowest Vibrational Mode (1/cm) = 13.17
4th Lowest Vibrational Mode (1/cm) = 13.99
DFT Electronic Energy (a.u.) = -5585.510557509193
DFT//DFT Electronic Energy (a.u.) = -5590.908979121035
Gibbs Free Energy (a.u.) = -5589.646851

DFT optimised cartesian coordinates:

O -2.05207160073451 -0.70011893242057 0.43570421997399
C -1.97224617973101 0.40639293749451 -0.17981726454899
N -3.01770119375912 0.93558363066428 -0.97297188242649
C -4.36344145394108 0.33198719809681 -0.91926705931003
C -2.69841343560492 2.26698045250685 -1.27978478894859
C -3.44212666692427 3.29731393239480 -1.86500707908598
C -2.86467792376929 4.56503211661341 -1.99207307412054
C -1.56260340778045 4.81569142724786 -1.54269231501755
C -0.81379656997699 3.80125258329016 -0.95042737426706
C -1.37304907383746 2.52353558640632 -0.80903577211736
C -0.91985559109965 1.32179241704192 -0.18888004260044
O 0.19662733740235 1.15855158438717 0.61711968442200
C -0.12275288831733 1.17814366572789 2.02472618174539
C -0.67155635047308 2.51983939188263 2.38223242267182
C -1.98991363474949 2.86227771667792 2.34223212353835
C -2.08776712388418 4.29818431876270 2.35764961072161
C -0.77086257357442 4.80456511513146 2.42899614606954
C -0.50264874754367 6.17850915397575 2.43179572940329
C -1.59135075054997 7.03824888564632 2.33751961116614
C -2.91050066949274 6.55298763155817 2.25381397417901
C -3.16883306789715 5.18932537766206 2.26731607270741
H -4.46443489428311 3.14760795054504 -2.20425292239711
H -3.45099605490630 5.36803270240777 -2.44151364356710
H -1.13629394096981 5.81610559141138 -1.64077627164896
H 0.18727449717415 4.00390286968055 -0.57030250333648
H 0.51215499780554 6.56757357043365 2.50138395379995
H -1.41475235328721 8.11494446613808 2.32670157969000
H -3.73691589546386 7.26106716625882 2.17746334681738
H -4.18824614709373 4.80650019297841 2.19873099061130
H 0.79669428762884 0.91639072576202 2.55334842713301
H -0.89879384252482 0.42513439963318 2.22033428850396
N 0.09688432267571 3.70648642183587 2.44893898257446
H -2.80929119181839 2.15144675069053 2.22604528437180
S 1.74304924526926 3.85380576783495 2.80594572305617
O 1.87440901615638 4.87765120328280 3.82583225205310
O 2.22554122233403 2.50870575571108 3.07157276708430
C -5.20813266939110 0.76562802686851 -2.13222658476075
C -4.60291336288675 0.87175116507834 -3.39205231486499
C -6.59483732094326 0.91005186809979 -2.04067225450339

C	-5.36061736476571	1.16017586398756	-4.52349766154818
H	-3.52260865287619	0.74319779238144	-3.48124415200737
C	-7.35698175397772	1.19773447437599	-3.17617897057813
H	-7.09408594414100	0.79611921280757	-1.07748084814088
C	-6.74350784478199	1.33178521292056	-4.41974354487675
H	-4.86769886769268	1.25200824767681	-5.49286606969025
H	-8.43790739238162	1.31460669310994	-3.08170440559886
H	-7.33796912549921	1.56070592469514	-5.30572482656155
C	-4.93997251520943	0.75848960183440	0.44559420089246
C	-4.76316920050603	-0.02996607045543	1.59135418750562
C	-5.49531513407943	2.03714953038278	0.60248258734624
C	-5.14934103268578	0.43723931928855	2.84887318693702
H	-4.30234933752651	-1.01215279987226	1.50237000390221
C	-5.88433087997678	2.50350019209925	1.85888820046138
H	-5.63194957530571	2.68261324583480	-0.26556859488031
C	-5.71369144822998	1.70483003699926	2.98982514149101
H	-5.00407562653449	-0.19878817734190	3.72390873774895
H	-6.32623202229586	3.49759662769604	1.94996835902370
H	-6.01787744943363	2.06805367809629	3.97293455718651
C	-4.29854577054669	-1.19759375414591	-1.13330774397869
C	-3.30125092031007	-1.77306642600428	-1.93108861897345
C	-5.33741712579358	-2.01518136193330	-0.67920844065089
C	-3.32078582455294	-3.13203901824130	-2.23451937398949
H	-2.48353558956315	-1.15354955437150	-2.30470128692694
C	-5.35951577162315	-3.37993062292266	-0.97955494217437
H	-6.14613468402538	-1.58829260653102	-0.08305425295395
C	-4.35002394428399	-3.94667042831508	-1.75443894395838
H	-2.51054498791056	-3.56053746662499	-2.83032644320712
H	-6.17028480293634	-4.00050886696870	-0.59476692034445
H	-4.35931577332577	-5.01493151947805	-1.97764013088335
C	2.47795178851292	4.44822794993102	1.31682471299457
C	2.49526250795253	3.61875067637053	0.19571114076048
C	3.12566185778923	5.68154490163199	1.33229140792328
C	3.17449744379715	4.04946607477323	-0.93827043497075
H	1.98198540407961	2.65344285737744	0.21333633136950
C	3.81266181029423	6.08595533374776	0.19056013997340
H	3.10605872076220	6.30031657245215	2.23083452037844
C	3.85620107379148	5.27524503425135	-0.95162844299545
H	3.20021903548382	3.41066175771187	-1.82387587922780
H	4.33933755013998	7.04292724806566	0.19084508108440
C	4.67054330513676	5.69737340405340	-2.14144448974822
H	5.73770982075126	5.69941263487721	-1.86847957787573
H	4.52870884668632	5.01480313682355	-2.98874508726753
H	4.40987463802890	6.71749972346162	-2.45736672164769
H	-1.13402311189619	-1.84080544845453	-0.07846604339501
N	-0.59373254245160	-2.69611578740462	-0.40522187568885
C	0.15872771479284	-2.56328271138122	-1.52192220474986
C	-0.96645760512587	-3.84431886821240	0.29434087574964
N	0.28767329426755	-1.27546188658628	-1.91560406253482
S	0.81867374167543	-3.87228846883035	-2.37506532822003
C	-0.10943224215873	-4.91601327759843	0.56263070636591
C	-2.26213298464023	-3.84698694593565	0.83935513276416
C	1.20250161647602	-0.75208399814065	-2.91261214988564
H	-0.18270294070685	-0.56984367084963	-1.34323096258909
C	-0.55922155671487	-5.97012487326405	1.36061069846034
H	0.90606213742353	-4.91151797125694	0.16815677596384
C	-2.68753959730002	-4.91305042823303	1.61945442417391
H	-2.92009523405200	-2.99816349398213	0.63687040738155
C	2.15751811677282	0.28933526118733	-2.26128783936592
H	1.79901049953058	-1.60823958992246	-3.25761581092761
C	0.45934197201485	-0.18626541138454	-4.15269540116387
C	0.40943006172731	-7.08088435709239	1.66191997760972
C	-1.84402487200663	-5.99170787153977	1.89320485345898
C	-4.10228770694157	-4.93713694023952	2.13006114888813
N	2.41906401490215	0.01620761466079	-0.85333173929292
H	1.72410094497033	1.29913930087021	-2.33952573219604
H	3.10327664465074	0.30331900312572	-2.82253827403043
C	1.49303120856074	0.37190425833532	-5.13807944810626
C	-0.30361343263582	-1.33357067222438	-4.82366981487472
C	-0.53770459220194	0.91303875755834	-3.76498720752638
F	-0.09410800813189	-7.97554916055043	2.51155303168799
F	0.77176883035394	-7.73996544065551	0.55775229223236
F	1.53779272899856	-6.60594875637950	2.20681072027680
H	-2.17807248330587	-6.82073056556486	2.51719741399607
F	-4.90399820792716	-5.64207413698717	1.32203328389350
F	-4.18299482499463	-5.50394872161662	3.33711909573520
F	-4.62525515681479	-3.71344338509890	2.22600229075593
H	1.68630261850868	0.35734840836237	-0.21606488547804
P	3.85794961184792	-0.29756387793741	-0.11306842266505
H	2.25620297997908	-0.38591386473315	-5.37717179162089
H	2.00067138300413	1.26399082897437	-4.74191637283408
H	0.99627475873044	0.66237180466689	-6.07569938913409
H	-0.84054861582446	-0.96179119074226	-5.70950117960842
H	-1.03926038726422	-1.77479349667109	-4.13347103076579
H	0.38277207943160	-2.13352380931153	-5.13910520725786
H	-1.08922831375661	1.24232168248648	-4.65931740387374
H	-0.04914556641701	1.79897760439393	-3.33238487206361
H	-1.27490232197754	0.55232691673743	-3.02931068354592
C	4.77631044391784	-1.45574152342512	-1.12975386994988
C	4.88873552062060	1.14559623526572	0.17724719870925
C	3.38555809762425	-1.08089838539786	1.43423813969780
C	4.05772229177644	-2.48241902647339	-1.74910192031397
C	6.17968538114062	-1.45167199186101	-1.21306472458155
C	4.86514160937265	1.82174217133207	1.40082163167274
C	5.60497091709203	1.70288207665655	-0.90132647705510

C 4.34525030255091 -1.32181555031089 2.43417803910670
C 2.09943297612348 -1.61431455794455 1.57655633641263
C 4.70352108963552 -3.47157890934872 -2.48252982506672
H 2.97132719826126 -2.53849859344651 -1.65468595170698
C 6.83323481431083 -2.43514580660374 -1.93721551245227
H 6.76777491533747 -0.68282700660463 -0.70912019755391
C 5.54115064539002 3.02990731262911 1.56324341790130
H 4.28707285906778 1.43379021707067 2.24106852568104
C 6.28132880196118 2.89885676884854 -0.74805245912180
H 5.62812432963621 1.20231568252755 -1.87246712769052
C 4.01402235907699 -2.06864740897246 3.55114300196371
H 5.36147735745112 -0.93204219529356 2.33417686231144
C 1.75900510726598 -2.36931761409572 2.69665993647577
H 1.33918978241627 -1.45717258842903 0.80986850717032
C 6.10303726922111 -3.44789167433321 -2.58639408497828
H 4.10600132628383 -4.25032037500735 -2.95580346690312
H 7.92051536963585 -2.44640992384517 -2.01859949839897
C 6.24261432029957 3.58111558278553 0.48315402850241
H 5.48650643971911 3.54231326731627 2.52297321702903
H 6.84033901710025 3.34151454910873 -1.57330941852365
C 2.72012319497366 -2.60762627899443 3.68907774044900
H 4.74539884194998 -2.26750312143243 4.33500070393823
H 0.74883542629548 -2.76992088280694 2.77694937066342
O 6.81824853731387 -4.34945794630428 -3.27009956853512
O 6.88920039957525 4.75447853800036 0.52761170625565
O 2.49305507242914 -3.33835050362647 4.78709839979872
C 6.13612532196970 -5.39728134301797 -3.93322048700830
C 6.94546752207163 5.45147568452208 1.75817297609550
C 1.23596391690842 -3.97469251166539 4.92770615983056
H 5.44356567667968 -4.99953683531636 -4.69059331187621
H 5.57673334646517 -6.01832243485527 -3.21728926170593
H 6.90381992049935 -6.00472411536205 -4.42287440349020
H 7.53181677085664 6.35752383412180 1.57448047242668
H 7.43930743941400 4.84149060216665 2.52997118386434
H 5.93716035111244 5.72933441473683 2.10110719121934
H 1.05308348161955 -4.66448178650159 4.08924250179555
H 1.27815721990576 -4.53865698957170 5.86483469866216
H 0.42189019462578 -3.23594267072682 4.97992191718562
----
ether_substrate
Frequencies, energies and thermodynamic properties:
Lowest Vibrational Mode (1/cm) = -22.22
2nd Lowest Vibrational Mode (1/cm) = 21.07
3rd Lowest Vibrational Mode (1/cm) = 25.32
4th Lowest Vibrational Mode (1/cm) = 28.3
DFT Electronic Energy (a.u.) = -2465.119994342233
DFT//DFT Electronic Energy (a.u.) = -2467.57206674992
Gibbs Free Energy (a.u.) = -2466.962623
DFT optimised cartesian coordinates:
O -0.69050926878312 0.58454421027061 2.17170721943674
C -0.65515267089936 -0.41747336527310 1.49808066749481
N -1.50376345988916 -0.75533774256358 0.45565426552584
C -2.69491382648498 0.04907829162089 0.08854975908371
C -1.34070088707886 -2.13561743621732 0.15410123400138
C -2.07402766947092 -2.96500936398369 -0.69426480622521
C -1.67331138589818 -4.30126698359786 -0.82418938678262
C -0.57660680856848 -4.81166259280255 -0.13186001914533
C 0.13759908010223 -3.98044666432865 0.73837783624252
C -0.26188626760005 -2.66206151153372 0.87975746425891
C 0.31264508681565 -1.59058954606365 1.74986698151015
O 1.65052684964392 -1.35526613966956 1.40042019416973
C 2.34027704448811 -0.31914906768317 2.05586924093393
C 2.25576237388230 1.02157184489226 1.38129367653783
C 2.11776793333471 2.22440647062313 1.99953080992251
C 2.23571170306821 3.25610965414277 1.00697093997163
C 2.44599423116220 2.62452176494049 -0.24208560077903
C 2.57470845492638 3.37378167766553 -1.42181015668268
C 2.49555278354381 4.75803967163984 -1.31524081956339
C 2.28989417010272 5.39917220262275 -0.07918240977881
C 2.16008194249781 4.65631796438164 1.08442264059318
H -2.92859725466574 -2.60722872213930 -1.26108285709139
H -2.24147889307918 -4.95179958661451 -1.49114872763186
H -0.28618177530506 -5.85515528851444 -0.25851328212480
H 0.99064121066573 -4.35779973466403 1.30638486318884
H 2.72718876209706 2.89853242253476 -2.38544834516190
H 2.59468873968303 5.35893188205957 -2.22070147184367
H 2.23187590325647 6.48795630655873 -0.04047231966052
H 1.99569787141287 5.14146226744933 2.04806711656510
H 3.39420604823387 -0.64101185810686 2.08735757928445
H 1.98933903967678 -0.18728068315841 3.09189000191978
N 2.47540380365589 1.23743869266908 0.00398033523503
H 1.94651786164200 2.35772472784217 3.06518615948134
S 2.44475498642061 0.04438907995563 -1.21355242518529
O 1.13737566580819 -0.59041503004153 -1.23315576651161
O 2.91306431917110 0.71288743968406 -2.41481879428071
C -2.39819959411183 1.56255815507828 0.17873696670138
C -1.16834816778065 2.06929033483440 -0.26452889454091
C -3.41222842581155 2.46074816278612 0.51804683436501
C -0.95470985764819 3.44294650885572 -0.33612780511448
H -0.37094694777775 1.37946628956564 -0.55715171920336
C -3.19576266085926 3.84021410375267 0.45021637347501
H -4.38856157126957 2.09151062711271 0.83407867722509
C -1.96630058141123 4.33667484983510 0.02736156914851
H 0.00809077752012 3.82190011529183 -0.68188131634948
H -4.00067158435400 4.52352178260591 0.72653132853597

H -1.79283796185176 5.41276304878733 -0.02805719535789
C -3.80824209331955 -0.42133353080408 1.04422008710321
C -3.80226845825621 0.04097148678827 2.37061693797064
C -4.73536588611733 -1.40862648782381 0.69180726112431
C -4.70867521017988 -0.45368401078573 3.30533936404043
H -3.07376189042895 0.79507649805165 2.66972593437645
C -5.64464698498867 -1.90579556202923 1.62919240005286
H -4.76669842945900 -1.79934104441776 -0.32471213054119
C -5.63817365470679 -1.42872863068429 2.93745612740896
H -4.68787604762435 -0.07380908755817 4.32820980822648
H -6.36036400681655 -2.67201474657170 1.32679754867821
H -6.35070710712308 -1.81451836180727 3.66841619771339
C -3.01238746403510 -0.10235122287285 -1.41152093830638
C -4.29464595292875 0.18383493211821 -1.89270713559797
C -1.98857747346749 -0.36087312226508 -2.33088856678091
C -4.56135961287386 0.16323386700887 -3.26217657461840
H -5.09561541866319 0.43725328279219 -1.19596540100158
C -2.25631113791287 -0.38065585788986 -3.69929685785569
H -0.97310842516033 -0.54723237024984 -1.97637409938618
C -3.54478942330089 -0.12764187232202 -4.17152483009865
H -5.56994105555277 0.38231468168222 -3.61656992908947
H -1.44726800405729 -0.59276677697331 -4.40034855651539
H -3.75265109761495 -0.14573001227874 -5.24260336714421
C 3.68286738010220 -1.09933187721418 -0.69297221467864
C 3.36028096465856 -2.44743492333137 -0.57252460060013
C 4.98131526583881 -0.62652674217257 -0.49899386788282
C 4.37086012389410 -3.34212545178560 -0.23014479731422
H 2.33105825956376 -2.77606503420105 -0.72010371367593
C 5.97247566289824 -1.53548222257123 -0.14653728034779
H 5.20946076613561 0.43612325183492 -0.60844066004096
C 5.68365883464028 -2.90292857134536 -0.00997639239456
H 4.13335872780583 -4.40251347513703 -0.12269810395262
H 6.99065364709328 -1.18086230606379 0.02522795752925
C 6.77201797841729 -3.88015523407723 0.33977473946383
H 7.26856457209874 -4.23610032659910 -0.57640872496749
H 7.53788074847494 -3.41067050065835 0.97086166969190
H 6.36609396784926 -4.75689167185772 0.86060989306430
H 0.23810546820403 -1.82892661043106 2.82768638365353
----
rearomatised_2-3_product
Frequencies, energies and thermodynamic properties:
Lowest Vibrational Mode (1/cm) = -38.77
2nd Lowest Vibrational Mode (1/cm) = 7.36
3rd Lowest Vibrational Mode (1/cm) = 13.49
4th Lowest Vibrational Mode (1/cm) = 17.58
DFT Electronic Energy (a.u.) = -2465.134708109368
DFT//DFT Electronic Energy (a.u.) = -2467.593239163541
Gibbs Free Energy (a.u.) = -2466.987774
DFT optimised cartesian coordinates:
O -1.49161069759633 1.30607517748101 1.77255967418088
C -1.23989656328119 0.32785720121378 1.11468696783454
N -2.04615786741478 -0.35712564212430 0.22076777162893
C -3.49563455176906 -0.14450364759148 -0.02951466360909
C -1.38848586092392 -1.55541597143250 -0.17900079664673
C -1.85192535012717 -2.57170658419646 -1.01349700466726
C -1.00022549847403 -3.65197284650024 -1.26697128323939
C 0.27936035270954 -3.72335135154522 -0.71232778244197
C 0.73135990900576 -2.70097700763807 0.12719235011065
C -0.11517058414942 -1.63530974495051 0.39113035077903
C 0.09363391828688 -0.44947084066806 1.29103392553878
O 0.08845327584536 -0.87915327041939 2.63548662693999
C 3.06845710169230 -0.55507423506205 2.57257479206573
C 2.54264465331597 0.33641176808557 1.49272646670182
C 1.29429399880873 0.39406709474406 0.93150567287090
C 1.32851778027988 1.37435768070612 -0.13325574105683
C 2.63020955317130 1.92105966570884 -0.16949817084381
C 2.98795332606635 2.90621802146507 -1.10066136042033
C 2.01623440638122 3.32408476615095 -2.00287069749724
C 0.71703905135074 2.78917661863358 -1.98394326358027
C 0.36540011970155 1.82120262486355 -1.05410080491712
H -2.84240503292110 -2.53826069724636 -1.46322821352660
H -1.35123033272607 -4.45347209024577 -1.91899851928148
H 0.92250781739032 -4.57600928848728 -0.93264066735788
H 1.72784465011989 -2.73383440923859 0.57370986003223
H 3.98673652352857 3.33343310633684 -1.11908519988947
H 2.27421147323308 4.08745298762213 -2.73844383711797
H -0.02339747928662 3.13713284145517 -2.70591167563143
H -0.64529799004948 1.41284413740474 -1.05436946651702
H 2.28607526075340 -1.25799374959611 2.86587951352435
H 3.38745930855846 0.02781529321470 3.44623430614503
N 3.36188339698312 1.28947376475343 0.85013906197319
S 5.04771611456787 1.38876546338142 1.04190398850166
O 5.44445661335345 2.62340170632027 0.39517667849738
O 5.33594528743374 1.17866320193962 2.44544124989747
C -4.21481771309109 -1.37694020792359 0.56987164050934
C -3.79639065275861 -1.82658969049812 1.83247929877508
C -5.24463512362527 -2.07085699080749 -0.06968014456256
C -4.40416605967922 -2.91832318055438 2.44559280178316
H -2.97449015793777 -1.32280823782540 2.34720283764365
C -5.85416897156304 -3.17027772932319 0.54312577380634
H -5.57675706011173 -1.78188242961725 -1.06523657204582
C -5.44247809226651 -3.59592258914580 1.80256223836267
H -4.06003735077977 -3.24444620595935 3.42837711243936
H -6.65267667426325 -3.69802722271661 0.01903877572101
H -5.91822608767551 -4.45495389093078 2.27828405952258

C	-3.75642202519959	0.05586170415147	-1.53769780905661
C	-2.74348070926744	0.03686980214700	-2.49990248689552
C	-5.05708702865278	0.38448144710003	-1.95258055866081
C	-3.02617011204301	0.30331773505560	-3.84376020692535
H	-1.71336275714509	-0.17923771188117	-2.22083621514922
C	-5.34339013881037	0.63892575886864	-3.29030293214963
H	-5.85441552629320	0.46586254511808	-1.21078196838979
C	-4.32559475212721	0.59548205270317	-4.24664238374611
H	-2.21490983117485	0.28357266070753	-4.57333263881131
H	-6.36419431412595	0.88763484580476	-3.58491232114965
H	-4.54534744281672	0.80224419989280	-5.29522062047441
C	-3.98518191234557	1.16218514393110	0.64476970364259
C	-4.86383261648519	1.17208980675170	1.72710137124610
C	-3.57206073593145	2.38940587537994	0.10296966743053
C	-5.30260870636556	2.38414039546852	2.27484603324561
H	-5.22456564724990	0.23961407017991	2.15904222678869
C	-3.99883826803057	3.59194160794741	0.65008054703990
H	-2.89768859956243	2.40024579355087	-0.75655144320865
C	-4.86911464190872	3.59439171466438	1.74589692899004
H	-5.99168857663206	2.36979556707783	3.12094855284907
H	-3.65501431751649	4.53412060643339	0.22006763901809
H	-5.21105894448567	4.53787345170845	2.17464872555879
C	5.66277235694620	0.01837847155628	0.10980984567542
C	5.51631022823483	0.02995524403653	-1.28039247518325
C	6.30448323340125	-1.02371153403284	0.77199290813657
C	6.00911504701024	-1.04491812941450	-2.00841632194150
H	5.02674131512348	0.86625073007313	-1.78461743682262
C	6.79751381980127	-2.08986745642007	0.01984914878506
H	6.41883862155059	-0.99669849809567	1.85667461499933
C	6.65447213445752	-2.11925595174881	-1.37178473759621
H	5.89915232400331	-1.05224879863163	-3.09478393815990
H	7.30557840214477	-2.91251919320735	0.52648675091967
C	7.17258199054840	-3.27446391996788	-2.18321885711215
H	6.33785609586881	-3.81979639268672	-2.64839419424551
H	7.82201257647009	-2.91773641305056	-2.99527983234189
H	7.74009483118745	-3.97654284213875	-1.56014777053409
H	0.04968445239127	-0.08019986318935	3.18635291669675
H	3.94274505336388	-1.12129793257968	2.22106965809648
